# Worldwide trends in blood pressure from 1975 to 2015: a pooled analysis of 1479 population-based measurement studies with 19·1 million participants

**DOI:** 10.1016/S0140-6736(16)31919-5

**Published:** 2017-01-07

**Authors:** NCD Risk Factor Collaboration (NCD-RisC)

## Abstract

**Background:**

Raised blood pressure is an important risk factor for cardiovascular diseases and chronic kidney disease. We estimated worldwide trends in mean systolic and mean diastolic blood pressure, and the prevalence of, and number of people with, raised blood pressure, defined as systolic blood pressure of 140 mm Hg or higher or diastolic blood pressure of 90 mm Hg or higher.

**Methods:**

For this analysis, we pooled national, subnational, or community population-based studies that had measured blood pressure in adults aged 18 years and older. We used a Bayesian hierarchical model to estimate trends from 1975 to 2015 in mean systolic and mean diastolic blood pressure, and the prevalence of raised blood pressure for 200 countries. We calculated the contributions of changes in prevalence versus population growth and ageing to the increase in the number of adults with raised blood pressure.

**Findings:**

We pooled 1479 studies that had measured the blood pressures of 19·1 million adults. Global age-standardised mean systolic blood pressure in 2015 was 127·0 mm Hg (95% credible interval 125·7–128·3) in men and 122·3 mm Hg (121·0–123·6) in women; age-standardised mean diastolic blood pressure was 78·7 mm Hg (77·9–79·5) for men and 76·7 mm Hg (75·9–77·6) for women. Global age-standardised prevalence of raised blood pressure was 24·1% (21·4–27·1) in men and 20·1% (17·8–22·5) in women in 2015. Mean systolic and mean diastolic blood pressure decreased substantially from 1975 to 2015 in high-income western and Asia Pacific countries, moving these countries from having some of the highest worldwide blood pressure in 1975 to the lowest in 2015. Mean blood pressure also decreased in women in central and eastern Europe, Latin America and the Caribbean, and, more recently, central Asia, Middle East, and north Africa, but the estimated trends in these super-regions had larger uncertainty than in high-income super-regions. By contrast, mean blood pressure might have increased in east and southeast Asia, south Asia, Oceania, and sub-Saharan Africa. In 2015, central and eastern Europe, sub-Saharan Africa, and south Asia had the highest blood pressure levels. Prevalence of raised blood pressure decreased in high-income and some middle-income countries; it remained unchanged elsewhere. The number of adults with raised blood pressure increased from 594 million in 1975 to 1·13 billion in 2015, with the increase largely in low-income and middle-income countries. The global increase in the number of adults with raised blood pressure is a net effect of increase due to population growth and ageing, and decrease due to declining age-specific prevalence.

**Interpretation:**

During the past four decades, the highest worldwide blood pressure levels have shifted from high-income countries to low-income countries in south Asia and sub-Saharan Africa due to opposite trends, while blood pressure has been persistently high in central and eastern Europe.

**Funding:**

Wellcome Trust.

## Introduction

Raised blood pressure is the leading global risk factor for cardiovascular diseases and chronic kidney disease.^1^ One of the global non-communicable disease (NCD) targets adopted by the World Health Assembly in 2013 is to lower the prevalence of raised blood pressure, defined as systolic blood pressure of 140 mm Hg or higher or diastolic blood pressure of 90 mm Hg or higher, by 25% compared with its 2010 level by 2025.^2^ Consistent global information is needed to understand how countries compare on blood pressure levels and trends, and where interventions to curtail the rise in blood pressure are most needed.

The prevalence of raised blood pressure measures the number of high-risk people irrespective of treatment status, and is the indicator used in the global NCD target. However, blood pressure has a log-linear association with cardiovascular diseases and chronic kidney disease that continues well below the threshold for raised blood pressure, and treatment provides similar proportional risk reductions irrespective of pretreatment blood pressure.^3^, ^4^ Trends in mean population blood pressure measure how blood pressure distribution has shifted over time.

We pooled population-based data to estimate national, regional, and global trends from 1975 to 2015 in mean systolic and mean diastolic blood pressure, and in the prevalence of raised blood pressure, for adults aged 18 years and older in 200 countries and territories. We also estimated trends in the number of adults with raised blood pressure, and calculated how much these trends are attributable to changes in prevalence versus changes in population size and age structure.

**Research in context**

**Evidence before this study**

We searched MEDLINE (via PubMed) for articles published in English, Spanish, Portuguese, Chinese, Italian, French, or Farsi between Jan 1, 1950, and Feb 19, 2014, using the search terms (“blood pressure”[Mesh:NoExp] OR “hypertension”[Mesh:NoExp]) AND (“Humans”[Mesh]). We screened articles according to the inclusion and exclusion criteria described in the appendix.

Some studies, including the MONICA Project, have reported on blood pressure change or trends in one or more countries. Two previous global analyses, done more than a decade ago, pooled data from different countries and reported mean systolic blood pressure or prevalence of hypertension in the year 2000 for the world and its major regions. A more recent analysis published in 2016 pooled 135 studies to estimate global and regional hypertension prevalence in 2000 and 2010, but did not report changes in mean blood pressure, which reflect shifts in the population distribution of blood pressure. None of these studies provided consistent estimates for all countries or accounted for the fact that the data used were collected in different years. The only analysis of trends at the country level reported mean systolic blood pressure from 1980 to 2008 but did not report mean diastolic blood pressure or prevalence of raised blood pressure, which is of clinical relevance and needed for monitoring progress towards the global target.

**Added value of this study**

This study provides the most complete picture of trends in adult blood pressure for all countries in the world with the longest observation period of any global blood pressure study to our knowledge, and includes trends in mean diastolic blood pressure and prevalence of raised blood pressure, which were not included in previous studies and are of clinical, public health, and health systems significance. We also estimated trends in the number of adults with raised blood pressure, and how much these trends are driven by changes in prevalence versus population size and age structure.

**Implications of all the available evidence**

During the past four decades, the highest levels of blood pressure worldwide have shifted from high-income countries to low-income and middle-income countries in south Asia and sub-Saharan Africa, while blood pressure has been persistently high in central and eastern Europe. The global target of reducing raised blood pressure prevalence by 25% by 2025 is unlikely to be achieved in these regions. The number of people with raised blood pressure has risen worldwide, with the increase happening mainly in low-income and middle-income countries. Population-based interventions throughout the life-course and pharmacological treatment for people with high absolute risk or people with substantially raised blood pressure should be a part of any effort to address the global burden of non-communicable diseases, especially in the poorest countries.

## Methods

### Study design and data sources

For this pooled analysis, we included data collected from samples of a national, subnational (ie, covering one or more subnational regions), or community (one or a small number of communities) population in which participants' blood pressure had been measured. Our methods for identifying and accessing data sources are described in the appendix (pp 2–6). When a study measured blood pressure more than once in participants (1053 [86%] of 1220 studies for which information about number of measurements was available), we discarded the first measurement, and used the average of the remainder.

292 (20%) of the 1479 data sources we analysed (2298 [16%] of 14 391 age-sex-study-specific data points) that were from a previous global pooling^5^ or extracted from publications did not have data on one or more of our primary outcomes. We used regressions to convert available data in these sources to the missing primary outcomes because the various blood pressure outcomes are correlated.^6^ Details of conversion (or so-called cross-walking) regressions and their coefficients are presented in the appendix (pp 7, 8, 44–152).

### Statistical analysis

The statistical model used to estimate means and prevalence by country, year, and age is described in detail in a statistical paper and related substantive papers.^5^, ^7^, ^8^ In summary, we organised countries into 21 regions, mainly on the basis of geography and national income, which we further aggregated into nine “super-regions” (appendix pp 14, 15). The model had a hierarchical structure in which estimates for each country and year were informed by its own data, if available, and by data from other years in the same country and from other countries, especially countries in the same region with data for similar time periods. The hierarchical structure shares information to a greater extent when data are non-existent or weakly informative (eg, have a small sample size or are not national), and to a lesser extent for data-rich countries and regions.

The model incorporated non-linear time trends and age patterns. It allowed the age association of blood pressure to vary across populations, and the rise in means and prevalence over age to be steeper where blood pressure is higher.^9^, ^10^ The model accounted for the possibility that blood pressure in subnational and community studies might systematically differ from nationally representative ones, and might also have larger variation than in national studies; the model also accounted for rural–urban differences in blood pressure, and used it to adjust rural-only and urban-only studies. The statistical model included covariates that help predict blood pressure, including mean number of years of education, proportion of national population living in urban areas, and a summary measure of availability of different food types for human consumption (appendix pp 9, 10).

We fitted the statistical model with the Markov chain Monte Carlo algorithm, and obtained 5000 post-burn-in samples from the posterior distribution of model parameters, which were in turn used to obtain the posterior distributions of primary outcomes. The reported credible intervals (CrI) represent the 2·5th to 97·5th percentiles of the posterior distributions. Each primary outcome was analysed separately, and all analyses were done separately by sex to allow blood pressure, its trends, and age associations to differ among outcomes and between men and women.

We calculated mean change in mean blood pressure and the prevalence of raised blood pressure across the 41 years of analysis (reported as change per decade). We also report the posterior probability (PP) that an estimated trend represents a true increase or decrease. We generated age-standardised estimates using the WHO standard population,^11^ by taking weighted means of age–sex-specific estimates, with use of age weights from the standard population. We tested how our statistical model predicted mean blood pressure and the prevalence of raised blood pressure when a country-year did not have data (appendix pp 11–13), which showed that the model performed well in its predictive validity.

We calculated the contribution of population growth and ageing to the change in the number of adults with raised blood pressure by fixing age-specific prevalence at its 1975 levels while allowing age-specific population to change as it did. We calculated the contribution of change in prevalence by fixing age-specific population at its 1975 level while allowing age-specific prevalence to change as it did. The interaction between the two contributions is the residual change in the number of adults with raised blood pressure after accounting for the two forementioned components.

### Role of funding source

The funder of the study had no role in study design, data collection, data analysis, data interpretation, or writing of the report. Country and Regional Data Group members and BZ had full access to the data in the study. The corresponding author had final responsibility for the decision to submit for publication.

## Results

We included 1479 population-based measurement surveys and studies, with 19·1 million participants aged 18 years and older for whom blood pressure was measured. We had at least one data source for 174 (87%) of the 200 countries we made estimates for, covering 97·5% of the world's population in 2015 (appendix pp 193, 194), and at least two data sources for 122 (61%) countries. Of these 1479 sources, 517 (35%) were from national samples, 249 (17%) covered one or more subnational regions, and the remaining 713 (48%) were from one or a small number of communities. Regionally, data availability ranged from 0·83 data sources per country in central Africa to 37 sources per country in high-income Asia Pacific. 543 (37%) data sources were from years before 1995 and another 936 (63%) were from 1995 and later.

**Figure 1:**
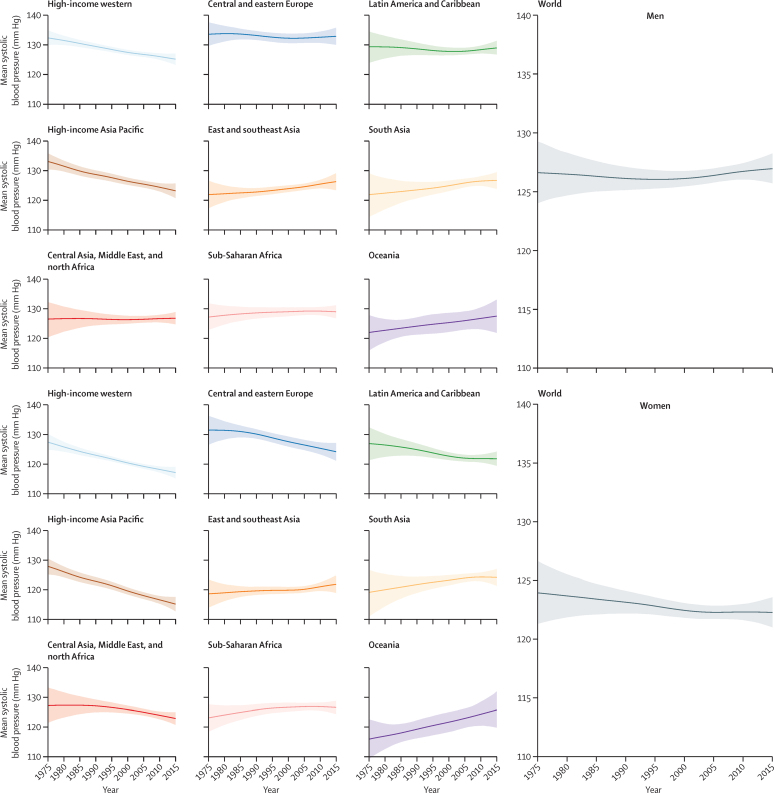
Trends in age-standardised mean systolic blood pressure by sex and super-region in people aged 18 years and older The lines show the posterior mean estimates and the shaded areas show the 95% CrI. See appendix (pp 199–266) for trends by country.

**Figure 2:**
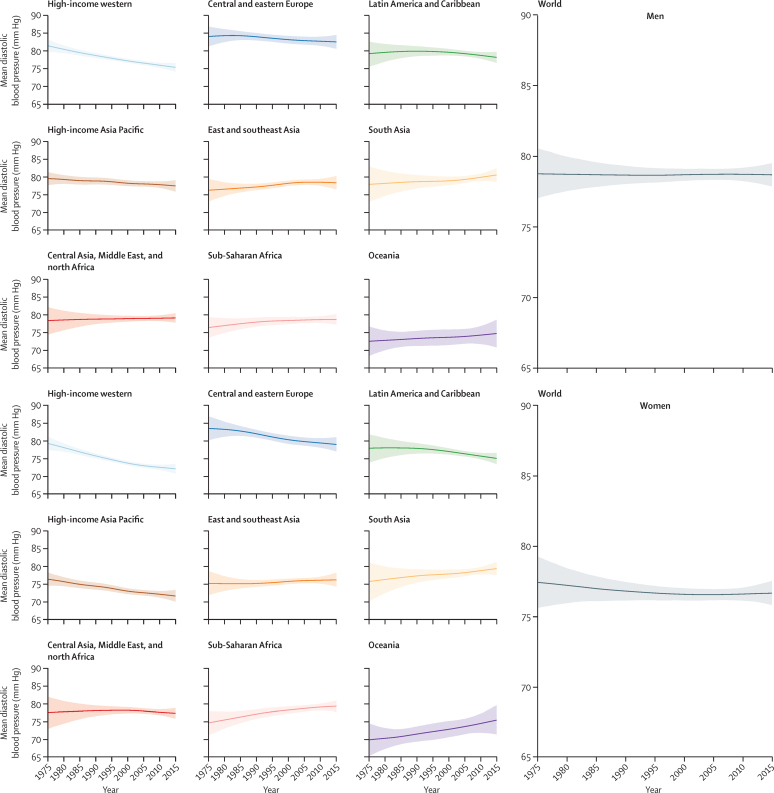
Trends in age-standardised mean diastolic blood pressure by sex and super-region in people aged 18 years and older The lines show the posterior mean estimates and the shaded areas show the 95% CrI. See appendix (pp 199–266) for trends by country.

Globally, age-standardised adult mean systolic blood pressure remained virtually unchanged from 1975 to 2015 in men (126·6 mm Hg [95% CrI 124·0 to 129·3] in 1975 and 127·0 mm Hg [125·7 to 128·3] in 2015; an increase of 0·07 mm Hg per decade [–0·59 to 0·74]; PP of being a true increasing trend is 0·5808) and decreased slightly in women (123·9 mm Hg [121·3 to 126·6] in 1975 and 122·3 mm Hg [121·0 to 123·6] in 2015; a decrease of 0·47 mm Hg per decade [–0·20 to 1·15]; PP=0·9210; figure 1). Trends in age-standardised mean diastolic blood pressure, which was 78·7 mm Hg (77·9 to 79·5) for men and 76·7 mm Hg (75·9 to 77·6) for women in 2015, were similar (figure 2).

Mean systolic and mean diastolic blood pressure decreased substantially during these four decades in high-income western and high-income Asia Pacific super-regions, moving these two super-regions from being among those with the highest blood pressure in 1975 to the lowest in 2015 (Figure 1, Figure 2). The largest decrease in mean systolic blood pressure, which occurred in high-income Asia Pacific, was 3·2 mm Hg per decade (95% CrI 2·4–3·9) for women and 2·4 mm Hg per decade (1·6–3·1) for men (PP>0·9999). The largest decrease in mean diastolic blood pressure, which was in the high-income western super-region, was 1·8 mm Hg per decade (1·4–2·3) for women and 1·5 mm Hg per decade (1·0–1·9) for men (PP>0·9999). Mean systolic blood pressure also seems to have decreased in women in central and eastern Europe, Latin America and the Caribbean, and, more recently, central Asia, Middle East, and north Africa, but the estimated trends in these super-regions had larger uncertainty than those in high-income super-regions; mean diastolic blood pressure showed a similar, but less pronounced, decrease in these super-regions (Figure 1, Figure 2). Little or no change in mean systolic or mean diastolic blood pressure occurred in men in these super-regions.

By contrast with these decreases, mean systolic blood pressure might have increased in men and women in east and southeast Asia, south Asia, Oceania, and sub-Saharan Africa, with a similar trend in mean diastolic blood pressure (Figure 1, Figure 2). Central and eastern Europe, sub-Saharan Africa, and south Asia had the highest mean blood pressures in 2015.

**Figure 3:**
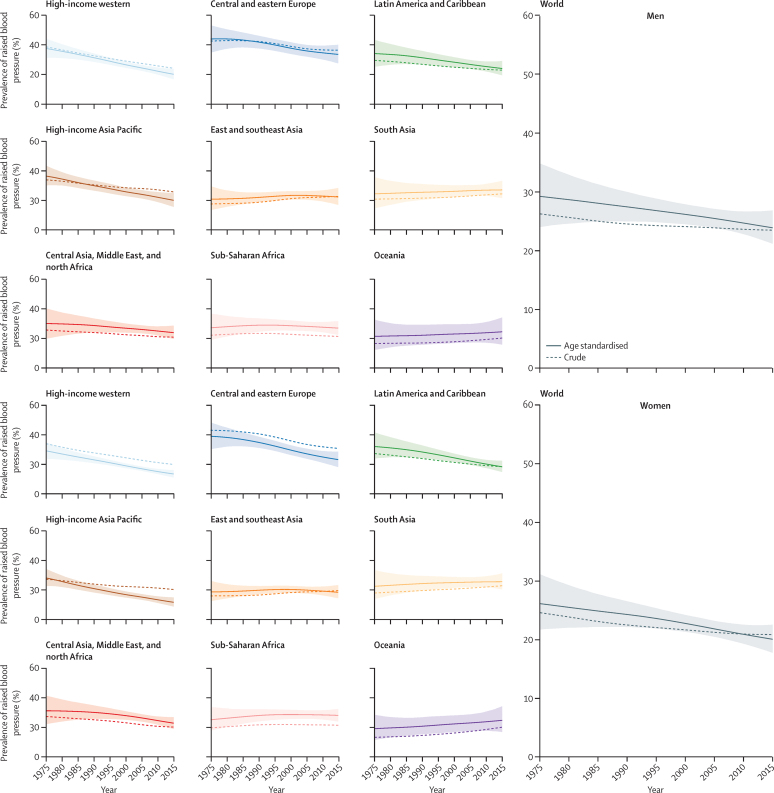
Trends in age-standardised and crude prevalence of raised blood pressure by sex and super-region in people aged 18 years and older The lines show the posterior mean estimates and the shaded area shows the 95% CrI for age-standardised prevalence. See appendix (pp 267–334) for trends by country.

Age-standardised prevalence of raised blood pressure decreased globally from 1975 to 2015, from 29·5% (95% CrI 24·2–35·0) to 24·1% (21·4–27·1) in men (PP=0·9482) and from 26·1% (21·7–31·1) to 20·1% (17·8–22·5) in women (PP=0·9884). The largest decrease was seen in high-income super-regions, followed by Latin America and the Caribbean, central and eastern Europe, and central Asia, Middle East, and north Africa (figure 3). Elsewhere, age-standardised prevalence of raised blood pressure remained unchanged. Crude prevalence decreased more slowly than age-standardised prevalence, especially where there has been substantial ageing (eg, in high-income super-regions and Latin America and the Caribbean).

**Figure 4:**
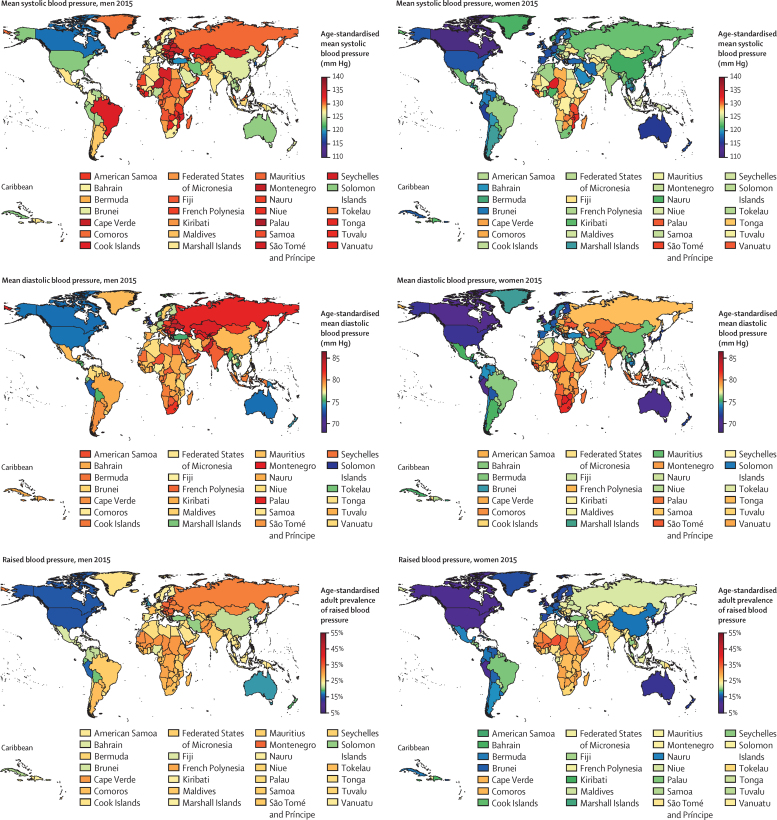
Age-standardised mean systolic blood pressure, mean diastolic blood pressure, and prevalence of raised blood pressure by sex and country in 2015 in people aged 18 years and older Interactive versions of these maps and downloadable numerical results are available online.

**Figure 5:**
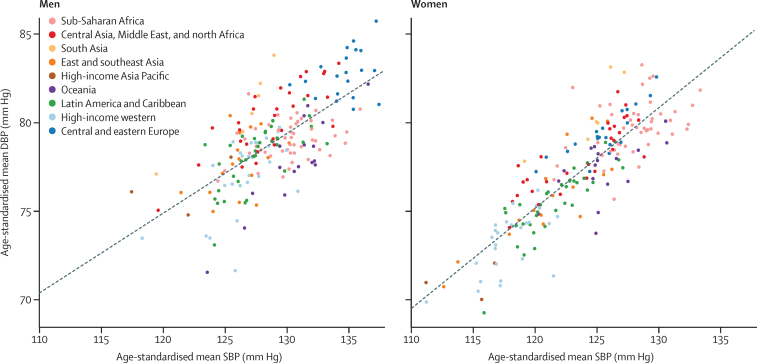
Relation between age-standardised mean systolic and mean diastolic blood pressure in men and women aged 18 years and older in 2015 The dotted line shows the linear association between the two outcomes. DBP=diastolic blood pressure. SBP=systolic blood pressure.

South Korea and Canada had the lowest age-standardised mean systolic blood pressure in 2015 for both men (117–118 mm Hg) and women (about 111 mm Hg; figure 4). The highest mean systolic blood pressures in men were seen in some countries in central and eastern Europe (eg, Slovenia, Lithuania, and Croatia), Oceania, central Asia, and sub-Saharan Africa, with age-standardised mean systolic blood pressure reaching 137·5 mm Hg (95% CrI 131·2–143·8) in Slovenia. Women in a few countries in sub-Saharan Africa (eg, Niger, Guinea, Malawi, and Mozambique) had the highest levels of mean systolic blood pressure, surpassing 132 mm Hg. Countries with the lowest mean diastolic blood pressure were Peru and several high-income countries including Canada, Australia, the UK, New Zealand, and Singapore. Diastolic blood pressure was high throughout central and eastern Europe, south Asia, and sub-Saharan Africa, with age-standardised mean surpassing 85 mm Hg in Lithuanian men. Mean systolic and mean diastolic blood pressure were correlated across countries (correlation coefficients of 0·69 for men and 0·86 for women in 2015). However, men and women in countries in south Asia, central and eastern Europe, and central Asia, Middle East, and north Africa had higher diastolic blood pressure than expected on the basis of their systolic blood pressure and the systolic blood pressure–diastolic blood pressure association (figure 5); the opposite was seen for men and women in Oceania.

South Korea, Canada, the USA, Peru, the UK, Singapore and Australia had the lowest prevalence of raised blood pressure in 2015 for both sexes, with an age-standardised prevalence of less than 13% in women and less than 19% in men (figure 4). At the other extreme, age-standardised prevalence surpassed 35% in men in some countries in central and eastern Europe including Croatia, Latvia, Lithuania, Hungary, and Slovenia; prevalence was more than 33% in women in a few countries in west Africa.

**Figure 6:**
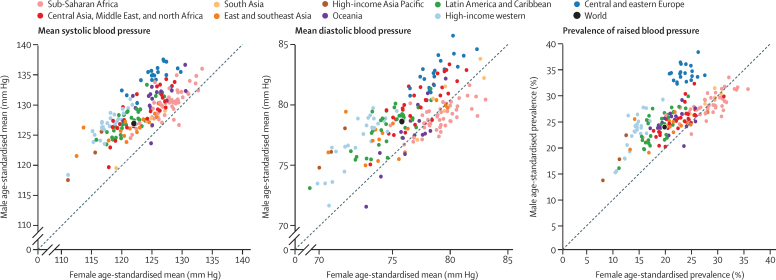
Comparison of age-standardised mean systolic blood pressure, mean diastolic blood pressure, and prevalence of raised blood pressure in men and women aged 18 years and older in 2015

In 2015, men had higher age-standardised mean systolic blood pressure than women in most countries (figure 6). Men also had higher diastolic blood pressure and prevalence of raised blood pressure than women in most countries, except in sub-Saharan Africa, where the sex pattern was reversed in most countries, and a few countries in Oceania and Asia. The male–female differences in age-standardised means and prevalence were virtually all due to differences in people younger than 50 years; among people aged 50 years and older, on average men and women had similar mean systolic and diastolic blood pressure and prevalence of raised blood pressure, with countries divided into some with lower and others with higher male blood pressure (results not shown). The male–female difference in blood pressure in 2015 was largest in high-income countries and countries in central and eastern Europe. Compared with 1975, the male excess in mean blood pressure increased in high-income super-regions, central and eastern Europe, Latin America and the Caribbean, and central Asia, Middle East, and north Africa but decreased (and in the case of diastolic blood pressure reversed) in sub-Saharan Africa, Oceania, and south Asia (results not shown).

**Figure 7:**
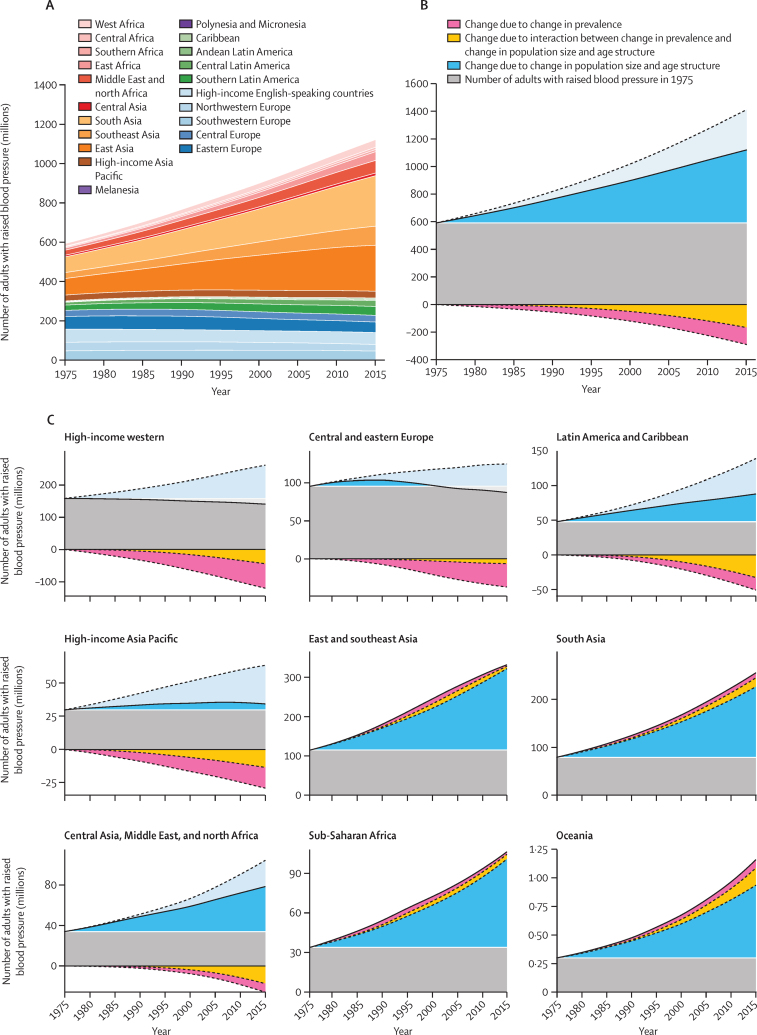
Trends in the number of adults aged 18 years and older with raised blood pressure Trends are (A) by region; (B) decomposed into the contributions of population growth and ageing, change in prevalence, and interaction of the two for the world; and (C) decomposed into the contributions of population growth and ageing, change in prevalence, and interaction of the two by super-region. (B, C) The solid black lines show the trends in the number of adults with raised blood pressure, and the light blue sections show how much of the rise in numbers due to population growth and ageing has been offset by the decrease in prevalence.

The estimated number of adults with raised blood pressure increased from 594 million in 1975 to 1·13 billion in 2015 (figure 7), comprising 597 million men and 529 million women. At the global level, this increase was attributable to population growth and ageing, offset partly by falling age-specific prevalence. In the high-income western super-region, the absolute number of people with raised blood pressure has decreased steadily since 1975 because the steep decrease in prevalence outweighed the effect of population growth and ageing. Nonetheless, 141 million adults in the constituent countries had raised blood pressure in 2015. Similarly, in central and eastern Europe, the number of people with raised blood pressure peaked in 1988 and went below its 1975 levels in 2002, driven by decreasing prevalence. In high-income Asia Pacific, the number of people with raised blood pressure has decreased since 2007 but is still higher than it was in 1975. In other low-income and middle-income super-regions, the number of people with raised blood pressure is still increasing. In Latin America and the Caribbean and central Asia, the Middle East, and north Africa, this rise is a net effect of increase due to population growth and ageing and decrease due to lower age-specific prevalence. In Oceania, south Asia, east and southeast Asia, and sub-Saharan Africa, three quarters or more of the rise is attributable to population growth and ageing, and the remainder is due to an increase in prevalence (figure 7). In 2015, 258 million (23%) of the 1·13 billion adults with raised blood pressure lived in south Asia (199 million of whom in India) and another 235 million (21%) lived in east Asia (226 million of whom in China).

## Discussion

Raised blood pressure has transitioned from a risk factor largely affecting high-income countries to one that is now most prevalent in low-income countries in south Asia and sub-Saharan Africa, while being a persistent health issue in central and eastern Europe. Although favourable trends continue in high-income countries, and might also be happening in some middle-income regions, other low-income and middle-income regions are affected by rising, or at best stable but high, blood pressure. The number of people with raised blood pressure in the world has increased by 90% during these four decades, with the majority of the increase occurring in low-income and middle-income countries, and largely driven by the growth and ageing of the population.

At the global level, we estimated lower mean systolic blood pressure in the 1980s, and hence a smaller reduction over time, than reported by Danaei and colleagues,^5^ possibly because we had more data than their earlier analysis. At the regional level, the additional data from low-income and middle-income countries included in our analysis gave more confidence to our finding of a rise in mean systolic blood pressure in Asia and sub-Saharan Africa than the trends estimated by Danaei and colleagues.^5^ Our results cannot be directly compared with the studies by Kearney and colleagues^12^ and Mills and colleagues^13^ because these studies included people who used antihypertensive medicines when calculating prevalence. Despite this difference in the reported metric, the reports are broadly consistent in identifying central and eastern Europe, central Asia, and sub-Saharan Africa as regions at the highest risk. Lawes and colleagues^14^ also reported the highest mean systolic blood pressure in central and eastern Europe and central Asia, as we did, but unlike our study they found lower mean systolic blood pressure in south Asia than in most regions. This difference is largely because blood pressure in south Asia has increased since 2000, the reporting year of Lawes and colleagues' study; the difference might also be attributable to us having substantially more data from south Asia than Lawes and colleagues.

The estimated decrease in blood pressure in high-income countries in our analysis is consistent with findings of country studies and the MONICA Project.^15^, ^16^, ^17^, ^18^, ^19^, ^20^, ^21^, ^22^, ^23^, ^24^, ^25^, ^26^, ^27^, ^28^, ^29^, ^30^, ^31^, ^32^, ^33^, ^34^ Fewer studies have analysed blood pressure trends in low-income and middle-income countries than in high-income countries. The available studies suggest reductions in blood pressure in central and possibly eastern Europe,^35^, ^36^, ^37^, ^38^ the Middle East and north Africa,^39^ and Latin America,^40^ and increases in south Asia and sub-Saharan Africa,^41^, ^42^, ^43^ and possibly in east and southeast Asia.^44^, ^45^

We also found that the prevalence of raised blood pressure decreased in some regions where mean blood pressure did not change, and remained unchanged where the mean increased. Some other studies^32^, ^46^ have also found a larger decrease in the upper tail of blood pressure distribution than in its mean. In the MONICA Project,^16^ the upper percentiles of blood pressure distribution decreased more than the mean in some communities but not in others. Although the changing shape of the distribution is partly due to antihypertensive drugs, it has also occurred in younger adult ages when medication use is uncommon.^32^, ^46^ To investigate the drivers of the changing distribution would require historical data on multiple determinants of blood pressure throughout the life course. Finally, our finding of a higher mean blood pressure in men than in women, especially in premenopause ages, is consistent with previous studies.^47^

The strengths of our study include its scope in making consistent and comparable estimates of trends in both mean and raised blood pressure over four decades for all the countries in the world. We used a large amount of population-based data covering countries in which more than 97% of the global adult population lives. We used only data from studies that had measured blood pressure to avoid bias in self-reported data. We analysed data according to a consistent protocol, and NCD Risk Factor Collaboration members verified the characteristics of data from each country through repeated checks. We pooled data using a statistical model that took into account the epidemiological features of blood pressure, including non-linear time trends and age associations. Our statistical model used all available data while giving more weight to national data than to subnational and community sources.

Similar to all global analyses, our study is affected by some limitations. First, some countries had no or few data sources, especially those in sub-Saharan Africa and the Caribbean. Estimates for these countries relied mostly or entirely on the statistical model. The absence or scarcity of data is reflected in wider uncertainty intervals of our estimates for these countries and regions, emphasising the importance of national NCD-oriented surveillance. Second, we had fewer data sources for the years before 1990 in most regions, which was reflected in the larger uncertainty for these years. In a sensitivity analysis, we analysed trends starting in 1990 with an identical model, and compared the post-1990 estimates with estimates from the main analysis (which included data from 1975 onwards). The estimates were very similar with correlation coefficients between the estimates from the main and sensitivity analyses being 0·94 or higher in 1990 and 0·98 or higher in 2015 (appendix pp 197, 198). Third, only 53% of sources included people older than 70 years, necessitating the use of data in these older ages elsewhere to infer an age pattern and make estimates in older ages. In view of the ageing trends throughout the world, inclusion of older people in health surveys should be emphasised. Fourth, our model accounted and adjusted for systematic and random errors in subnational and community data. However, the adjustments are not country-specific because estimation of country-specific adjustments would require national and subnational or community data in the same country and year. Therefore, the correction for each single country remains uncertain. Fifth, although data held by NCD Risk Factor Collaboration members were analysed to provide all the primary outcomes, individual participant data could not be accessed for 20% of data sources. To overcome this issue, we systematically used the reported metrics to estimate all of our primary outcomes; the cross-walking regressions used for this purpose had good predictive accuracy but increased the uncertainty of our estimates. Sixth, over time, standard mercury sphygmomanometers have been replaced by random-zero sphygmomanometers and more recently digital oscillometric devices in health surveys. Similarly, studies differed on whether they used multiple cuff sizes or one cuff size. We note that the effect of measurement device and protocol on population mean and prevalence depends on the circumstances of each survey. For example, an automated digital device with a standard cuff, although not the traditional gold-standard in a clinical setting, avoids observer bias and increases compliance, and possibly even response rate, compared with a standard mercury sphygmomanometer with multiple cuffs.^48^ Nonetheless, measurements from different devices are not fully comparable,^49^, ^50^, ^51^ which might have affected the estimated trends. When we included device type as study-level covariate in our statistical model, studies using random-zero sphygmomanometers, which were used commonly in the late 1980s and 1990s, had lower mean blood pressure (by about 4·5 mm Hg for systolic blood pressure and by about 3 mm Hg for diastolic blood pressure) and prevalence of raised blood pressure than studies using standard mercury sphygmomanometers. The mean difference between studies using digital devices and mercury sphygmomanometers was about 2 mm Hg for systolic blood pressure and about 0·2 mm Hg for diastolic blood pressure. Finally, blood pressure had been measured only once in some of our data sources. In those sources with multiple measurements, the median difference between the first measurement and the average of subsequent ones was 1·5 mm Hg for systolic blood pressure and 0·0 mm Hg for diastolic blood pressure, suggesting that mean blood pressure and prevalence of raised blood pressure might be slightly overestimated in some of our sources.

Blood pressure is a multifaceted trait, affected by nutrition, environment, and behaviour throughout the life course, including fetal and early childhood nutrition and growth,^52^ adiposity,^53^, ^54^ specific components of diet, especially sodium and potassium intakes,^53^ alcohol use,^54^, ^55^ smoking,^56^ physical activity,^54^ air pollution,^57^ lead,^58^ noise,^59^ psychosocial stress, and the use of blood pressure lowering drugs. Changes in risk factors and improvements in detection and treatment of raised blood pressure have, at least partly, resulted in the decrease in blood pressure in high-income countries, although the decrease seems to have begun before or in the absence of specific interventions for risk factors and scale-up of treatment, and is only partly accounted for by the measured risk factors and treatment.^17^, ^19^, ^20^, ^21^, ^34^, ^35^, ^46^, ^60^, ^61^, ^62^, ^63^, ^64^, ^65^, ^66^, ^67^, ^68^, ^69^, ^70^ In particular, the decrease in high-income and some middle-income countries has happened despite increasing body-mass index.^71^

The partly unexplained nature of these favourable trends necessitates speculation about their drivers, which might include unmeasured improvements in early childhood nutrition and year-round availability of fruits and vegetables, which might increase the amount and regularity of their consumption. Our results show that similar decreasing trends in mean blood pressure and prevalence of raised blood pressure might have begun in some middle-income regions, although at a slower rate than trends in high-income regions, but not in the poorest populations, including those in south Asia and sub-Saharan Africa, and in populations affected by major social and economic changes in central and eastern Europe. These populations have low consumption of fresh fruits^72^ and, in many cases, high consumption of salt.^73^ South Asia and sub-Saharan Africa have the highest prevalence of maternal undernutrition,^71^, ^74^ preterm and small-for-gestational age births, and child undernutrition;^75^, ^76^ they have also had some of the smallest gains in adult height,^74^ which is associated with lower risk of cardiovascular diseases. Many cases of raised blood pressure go untreated in these regions.^13^, ^77^ The absence of these favourable determinants of low blood pressure, coupled with rising body-mass index,^71^ might be causing the increase in mean blood pressure in these regions. Therefore, if governments and multinational organisations are to address the large and inequitable burden of cardiovascular diseases and kidney disease associated with high blood pressure, they need to take a multifaceted approach using both population-based strategies throughout the life course and individual lifestyle management and treatment through primary care systems.^78^

Correspondence to: Prof Majid Ezzati, Imperial College London, London W2 1PG, UK **majid.ezzati@imperial.ac.uk**

## References

[1] Global Burden of Metabolic Risk Factors for Chronic Diseases Collaboration (2014). Cardiovascular disease, chronic kidney disease, and diabetes mortality burden of cardiometabolic risk factors from 1980 to 2010: a comparative risk assessment. Lancet Diabetes Endocrinol.

[2] WHO (2013). Global action plan for the prevention and control of noncommunicable diseases 2013–2020. http://apps.who.int/iris/bitstream/10665/94384/1/9789241506236_eng.pdf?ua=1.

[3] Lewington S, Clarke R, Qizilbash N, Peto R, Collins R, Prospective Studies Collaboration (2002). Age-specific relevance of usual blood pressure to vascular mortality: a meta-analysis of individual data for one million adults in 61 prospective studies. Lancet.

[4] Czernichow S, Zanchetti A, Turnbull F (2011). The effects of blood pressure reduction and of different blood pressure-lowering regimens on major cardiovascular events according to baseline blood pressure: meta-analysis of randomized trials. J Hypertens.

[5] Danaei G, Finucane MM, Lin JK (2011). National, regional, and global trends in systolic blood pressure since 1980: systematic analysis of health examination surveys and epidemiological studies with 786 country-years and 5·4 million participants. Lancet.

[6] Rose G, Day S (1990). The population mean predicts the number of deviant individuals. BMJ.

[7] Finucane MM, Paciorek CJ, Danaei G, Ezzati M (2014). Bayesian estimation of population-level trends in measures of health status. Stat Sci.

[8] NCD Risk Factor Collaboration (2016). Worldwide trends in diabetes since 1980: a pooled analysis of 751 population-based studies with 4.4 million participants. Lancet.

[9] Singh GM, Danaei G, Pelizzari PM (2012). The age associations of blood pressure, cholesterol, and glucose: analysis of health examination surveys from international populations. Circulation.

[10] Rodriguez BL, Labarthe DR, Huang B, Lopez-Gomez J (1994). Rise of blood pressure with age. New evidence of population differences. Hypertension.

[11] Ahmad OB, Boschi-Pinto C, Lopez AD, Murray CJ, Lozano R, Inoue M (2001). Age standardization of rates: a new WHO standard.

[12] Kearney PM, Whelton M, Reynolds K, Muntner P, Whelton PK, He J (2005). Global burden of hypertension: analysis of worldwide data. Lancet.

[13] Mills KT, Bundy JD, Kelly TN (2016). Global disparities of hypertension prevalence and control: a systematic analysis of population-based studies from 90 countries. Circulation.

[14] Lawes CM, Vander Hoorn S, Law MR, Elliott P, MacMahon S, Rodgers A (2006). Blood pressure and the global burden of disease 2000. Part 1: estimates of blood pressure levels. J Hypertens.

[15] Evans A, Tolonen H, Hense HW (2001). Trends in coronary risk factors in the WHO MONICA project. Int J Epidemiol.

[16] Tunstall-Pedoe H, Connaghan J, Woodward M, Tolonen H, Kuulasmaa K (2006). Pattern of declining blood pressure across replicate population surveys of the WHO MONICA project, mid-1980s to mid-1990s, and the role of medication. BMJ.

[17] McCarron P, Smith GD, Okasha M (2002). Secular changes in blood pressure in childhood, adolescence and young adulthood: systematic review of trends from 1948 to 1998. J Hum Hypertens.

[18] Bennett SA, Magnus P (1994). Trends in cardiovascular risk factors in Australia. Results from the National Heart Foundation's Risk Factor Prevalence Study, 1980–1989. Med J Aust.

[19] Joossens JV, Kesteloot H (1991). Trends in systolic blood pressure, 24-hour sodium excretion, and stroke mortality in the elderly in Belgium. Am J Med.

[20] Ikeda N, Gakidou E, Hasegawa T, Murray CJ (2008). Understanding the decline of mean systolic blood pressure in Japan: an analysis of pooled data from the National Nutrition Survey, 1986–2002. Bull World Health Organ.

[21] Ueshima H, Tatara K, Asakura S, Okamoto M (1987). Declining trends in blood pressure level and the prevalence of hypertension, and changes in related factors in Japan, 1956–1980. J Chronic Dis.

[22] Wietlisbach V, Paccaud F, Rickenbach M, Gutzwiller F (1997). Trends in cardiovascular risk factors (1984–1993) in a Swiss region: results of three population surveys. Prev Med.

[23] Tverdal A (2001). Significant decline in blood pressure levels after 1996—fact or artefact?. Tidsskr Nor Laegeforen.

[24] Kastarinen MJ, Nissinen AM, Vartiainen EA (2000). Blood pressure levels and obesity trends in hypertensive and normotensive Finnish population from 1982 to 1997. J Hypertens.

[25] Juonala M, Viikari JS, Hutri-Kahonen N (2004). The 21-year follow-up of the Cardiovascular Risk in Young Finns Study: risk factor levels, secular trends and east-west difference. J Intern Med.

[26] Nuotio J, Oikonen M, Magnussen CG (2014). Cardiovascular risk factors in 2011 and secular trends since 2007: the Cardiovascular Risk in Young Finns Study. Scand J Public Health.

[27] Borodulin K, Vartiainen E, Peltonen M (2015). Forty-year trends in cardiovascular risk factors in Finland. Eur J Public Health.

[28] Pereira M, Carreira H, Vales C, Rocha V, Azevedo A, Lunet N (2012). Trends in hypertension prevalence (1990–2005) and mean blood pressure (1975–2005) in Portugal: a systematic review. Blood Press.

[29] Giampaoli S, Palmieri L, Donfrancesco C, Lo Noce C, Pilotto L, Vanuzzo D (2015). Cardiovascular health in Italy. Ten-year surveillance of cardiovascular diseases and risk factors: Osservatorio Epidemiologico Cardiovascolare/Health Examination Survey 1998–2012. Eur J Prev Cardiol.

[30] Hoffmeister H, Mensink GB, Stolzenberg H (1996). Reduction of coronary heart disease risk factors in the German cardiovascular prevention study. Prev Med.

[31] Ezzati M, Oza S, Danaei G, Murray CJ (2008). Trends and cardiovascular mortality effects of state-level blood pressure and uncontrolled hypertension in the United States. Circulation.

[32] Burt VL, Cutler JA, Higgins M (1995). Trends in the prevalence, awareness, treatment, and control of hypertension in the adult US population. Data from the health examination surveys, 1960 to 1991. Hypertension.

[33] Guo F, He D, Zhang W, Walton RG (2012). Trends in prevalence, awareness, management, and control of hypertension among United States adults, 1999 to 2010. J Am Coll Cardiol.

[34] Falaschetti E, Mindell J, Knott C, Poulter N (2014). Hypertension management in England: a serial cross-sectional study from 1994 to 2011. Lancet.

[35] Cifkova R, Skodova Z, Bruthans J (2010). Longitudinal trends in major cardiovascular risk factors in the Czech population between 1985 and 2007/8. Czech MONICA and Czech post-MONICA. Atherosclerosis.

[36] Vlasoff T, Laatikainen T, Korpelainen V (2008). Ten year trends in chronic disease risk factors in the Republic of Karelia, Russia. Eur J Public Health.

[37] Abina J, Volozh O, Solodkaya E, Saava M (2003). Blood pressure and contributing factors in inhabitants of Estonia: 15-year trends. Blood Press.

[38] Dorobantu M, Darabont R, Ghiorghe S (2014). Hypertension prevalence and control in Romania at a seven-year interval. Comparison of SEPHAR I and II surveys. J Hypertens.

[39] Sozmen K, Unal B, Saidi O (2015). Cardiovascular risk factor trends in the Eastern Mediterranean region: evidence from four countries is alarming. Int J Public Health.

[40] Picon RV, Fuchs FD, Moreira LB, Riegel G, Fuchs SC (2012). Trends in prevalence of hypertension in Brazil: a systematic review with meta-analysis. PLoS One.

[41] Fezeu L, Kengne AP, Balkau B, Awah PK, Mbanya JC (2010). Ten-year change in blood pressure levels and prevalence of hypertension in urban and rural Cameroon. J Epidemiol Community Health.

[42] Gupta R, al-Odat NA, Gupta VP (1996). Hypertension epidemiology in India: meta-analysis of 50 year prevalence rates and blood pressure trends. J Hum Hypertens.

[43] Gupta R (2004). Trends in hypertension epidemiology in India. J Hum Hypertens.

[44] Nguyen QN, Pham ST, Nguyen VL (2012). Time trends in blood pressure, body mass index and smoking in the Vietnamese population: a meta-analysis from multiple cross-sectional surveys. PLoS One.

[45] Guo J, Zhu YC, Chen YP, Hu Y, Tang XW, Zhang B (2015). The dynamics of hypertension prevalence, awareness, treatment, control and associated factors in Chinese adults: results from CHNS 1991–2011. J Hypertens.

[46] Goff DC, Howard G, Russell GB, Labarthe DR (2001). Birth cohort evidence of population influences on blood pressure in the United States, 1887–1994. Ann Epidemiol.

[47] Reckelhoff JF (2001). Gender differences in the regulation of blood pressure. Hypertension.

[48] Myers MG, Godwin M, Dawes M (2011). Conventional versus automated measurement of blood pressure in primary care patients with systolic hypertension: randomised parallel design controlled trial. BMJ.

[49] Pickering TG, Hall JE, Appel LJ (2005). Recommendations for blood pressure measurement in humans and experimental animals: part 1: blood pressure measurement in humans: a statement for professionals from the Subcommittee of Professional and Public Education of the American Heart Association Council on High Blood Pressure Research. Circulation.

[50] Ogedegbe G, Pickering T (2010). Principles and techniques of blood pressure measurement. Cardiol Clin.

[51] O'Brien E, Waeber B, Parati G, Staessen J, Myers MG (2001). Blood pressure measuring devices: recommendations of the European Society of Hypertension. BMJ.

[52] Victora CG, Adair L, Fall C (2008). Maternal and child undernutrition: consequences for adult health and human capital. Lancet.

[53] Sacks FM, Campos H (2010). Dietary therapy in hypertension. N Engl J Med.

[54] Institute of Medicine (2010). A population-based policy and systems change approach to prevent and control hypertension.

[55] Taylor B, Irving HM, Baliunas D (2009). Alcohol and hypertension: gender differences in dose-response relationships determined through systematic review and meta-analysis. Addiction.

[56] Virdis A, Giannarelli C, Neves MF, Taddei S, Ghiadoni L (2010). Cigarette smoking and hypertension. Curr Pharm Des.

[57] Liang R, Zhang B, Zhao X, Ruan Y, Lian H, Fan Z (2014). Effect of exposure to PM2.5 on blood pressure: a systematic review and meta-analysis. J Hypertens.

[58] Navas-Acien A, Schwartz BS, Rothenberg SJ, Hu H, Silbergeld EK, Guallar E (2008). Bone lead levels and blood pressure endpoints: a meta-analysis. Epidemiology.

[59] van Kempen E, Babisch W (2012). The quantitative relationship between road traffic noise and hypertension: a meta-analysis. J Hypertens.

[60] Ezzati M, Obermeyer Z, Tzoulaki I, Mayosi BM, Elliott P, Leon DA (2015). Contributions of risk factors and medical care to cardiovascular mortality trends. Nat Rev Cardiol.

[61] Sakata K, Labarthe DR (1996). Changes in cardiovascular disease risk factors in three Japanese national surveys 1971–1990. J Epidemiol.

[62] Bernstein AM, Willett WC (2010). Trends in 24-h urinary sodium excretion in the United States, 1957–2003: a systematic review. Am J Clin Nutr.

[63] Schiffrin EL, Campbell NR, Feldman RD (2016). Hypertension in Canada: past, present, and future. Ann Glob Health.

[64] Walker RL, Chen G, Campbell NR (2011). Canadian provincial trends in antihypertensive drug prescriptions between 1996 and 2006. Can J Cardiol.

[65] He FJ, Pombo-Rodrigues S, Macgregor GA (2014). Salt reduction in England from 2003 to 2011: its relationship to blood pressure, stroke and ischaemic heart disease mortality. BMJ Open.

[66] Du S, Batis C, Wang H, Zhang B, Zhang J, Popkin BM (2014). Understanding the patterns and trends of sodium intake, potassium intake, and sodium to potassium ratio and their effect on hypertension in China. Am J Clin Nutr.

[67] Banegas JR, Navarro-Vidal B, Ruilope LM (2015). Trends in hypertension control among the older population of Spain from 2000 to 2001 to 2008 to 2010: role of frequency and intensity of drug treatment. Circ Cardiovasc Qual Outcomes.

[68] Blix HS, Landmark K, Selmer R, Reikvam A (2012). Patterns in the prescription of antihypertensive drugs in Norway, 1975–2010. Tidsskr Nor Laegeforen.

[69] Andersen UO, Jensen GB (2010). Trends and determinant factors for population blood pressure with 25 years of follow-up: results from the Copenhagen City Heart Study. Eur J Cardiovasc Prev Rehabil.

[70] Laatikainen T, Nissinen A, Kastarinen M, Jula A, Tuomilehto J (2016). Blood pressure, sodium intake, and hypertension control: lessons from the North Karelia Project. Glob Heart.

[71] NCD Risk Factor Collaboration (2016). Trends in adult body-mass index in 200 countries from 1975 to 2014: a pooled analysis of 1698 population-based measurement studies with 19·2 million participants. Lancet.

[72] Micha R, Khatibzadeh S, Shi P (2015). Global, regional and national consumption of major food groups in 1990 and 2010: a systematic analysis including 266 country-specific nutrition surveys worldwide. BMJ Open.

[73] Powles J, Fahimi S, Micha R (2013). Global, regional and national sodium intakes in 1990 and 2010: a systematic analysis of 24 h urinary sodium excretion and dietary surveys worldwide. BMJ Open.

[74] NCD Risk Factor Collaboration (2016). A century of trends in adult human height. eLife.

[75] Lee AC, Katz J, Blencowe H (2013). National and regional estimates of term and preterm babies born small for gestational age in 138 low-income and middle-income countries in 2010. Lancet Glob Health.

[76] Stevens GA, Finucane MM, Paciorek CJ (2012). Trends in mild, moderate, and severe stunting and underweight, and progress towards MDG 1 in 141 developing countries: a systematic analysis of population representative data. Lancet.

[77] Chow CK, Teo KK, Rangarajan S (2013). Prevalence, awareness, treatment, and control of hypertension in rural and urban communities in high-, middle-, and low-income countries. JAMA.

[78] Olsen MH, Angell SY, Asma S (2016). A call to action and a lifecourse strategy to address the global burden of raised blood pressure on current and future generations: the *Lancet* Commission on hypertension. Lancet.

