# Department of Error

**DOI:** 10.1016/S0140-6736(20)31972-3

**Published:** 2020-09-26

*NCD Risk Factor Collaboration (NCD-RisC). Worldwide trends in blood pressure from 1975 to 2015: a pooled analysis of 1479 population-based measurement studies with 19·1 million participants.* Lancet *2017; **389:** 37–55—*In this Article, spelling of author Niels Wedderkopp's name was incorrect. This correction has been made to the online version as of Sept 24, 2020.

