# Supplementary material for: Worldwide trends in blood pressure from 1975 to 2015: a pooled analysis of 1479 population-based measurement studies with 19·1 million participants
Source: Lancet. 2017 Jan 7;389(10064):37–55. doi: 10.1016/S0140-6736(16)31919-5 (PMC5220163; doi:10.1016/S0140-6736(16)31919-5)
Supplement: Supplementary appendix [file mmc1.pdf]

THE LANCET

Supplementary appendix

This appendix formed part of the original submission and has been peer reviewed. We post it as supplied by the authors.

Supplement to: NCD Risk Factor Collaboration (NCD-RisC). Worldwide trends in blood pressure from 1975 to 2015: a pooled analysis of 1479 population-based measurement studies with 19·1 million participants. *Lancet* 2016; published online Nov 15. http://dx.doi.org/10.1016/S0140-6736(16)31919-5.

Appendix

Worldwide trends in blood pressure from 1975 to 2015: a pooled analysis of 1,479 population-based measurement studies with 19.1 million participants

NCD Risk Factor Collaboration (NCD-RisC)

Table of Contents

Appendix 1: Data sources 2

Appendix 2: Converting to primary blood pressure metrics 7

Appendix 3: Model covariates 9

Appendix 4: Validation of statistical model 11

Appendix Table 1: List of analysis regions and "super-regions", and countries in each region 14

Appendix Table 2: Data sources used in the analysis. 16

Appendix Table 3: Model specifications and regression coefficients to estimate the primary outcomes (mean SBP, mean DBP, and prevalence of raised blood pressure defined by SBP ≥140 mmHg or DBP ≥90 mmHg) from other metrics. 44

Appendix Table 4: Results of model validation. 153

Appendix Table 5: Mean systolic blood pressure, mean diastolic blood pressure and prevalence of raised blood pressure by sex and age group for the world and nine super-regions in 1975, 1985, 1995, 2005 and 2015. 166

Appendix Figure 1: Flowchart of secondary search for data sources. 191

Appendix Figure 2: Number of data sources by country. 193

Appendix Figure 3: Number of data sources by region and year. 195

Appendix Figure 4: Comparison of age-standardised mean systolic blood pressure, mean diastolic blood pressure and prevalence of raised blood pressure in the main analysis, which used data from 1975 to 2015, with a sensitivity analysis using data from 1990 to 2015. 197

Appendix Figure 5: Trends in age-standardised mean systolic and diastolic blood pressure by sex and country in people aged 18 years and older. 199

Appendix Figure 6: Trends in age-standardised and crude prevalence of raised blood pressure by sex and country in people aged 18 years and older. 267

References 335

Appendix 1: Data sources

Data inclusion and exclusion

Data sources were included in NCD-RisC database if:

- measured data on systolic blood pressure (SBP) and/or diastolic blood pressure (DBP) were available;
- study participants were ten years of age and older;
- data were collected using a probabilistic sampling method with a defined sampling frame;
- data were from population samples at the national, sub-national (i.e. covering one or more sub-national regions), or community (one or a small number of communities) level;
- data were collected in or after 1950;
- data were from the countries and territories listed in Appendix Table 1.

We excluded all data sources that included only hypertension diagnosis history or medication status without measurement of blood pressure. We also excluded data sources on population subgroups whose blood pressure levels may differ systematically from the general population, including:

- studies that had included or excluded people based on their health status or cardiovascular risk;
- ethnic minorities;
- specific educational, occupational, or socioeconomic subgroups of the population; and
- those recruited through health facilities, with the exception noted below.

We used school-based data in countries where secondary school enrolment was 70% or higher, and used data whose sampling frame was health insurance schemes in countries where at least 80% of the population were insured. In addition, we used data collected through general practice and primary care system in high-income countries with universal insurance, because contact with the primary care systems tends to be at least as good as response rates for population-based surveys.

In this paper, we used data from the NCD-RisC database for years 1975 through 2015, and among subjects aged 18 years and older.

Primary routes for data access

We used a database of population-based data on cardiometabolic risk factors collated by the NCD Risk Factor Collaboration (NCD-RisC), a worldwide network of health researchers and practitioners whose aim is to understand the worldwide trends and variations in NCD risk factors. The initial database was collated through multiple routes for identifying and accessing data. We accessed publicly available population-based multi-country and national measurement surveys (e.g., Demographic and Health Surveys, and surveys identified via the Inter-University Consortium for Political and Social Research and European Health Interview & Health Examination Surveys Database) as well as the World Health Organization (WHO) STEPwise approach to Surveillance (STEPS) surveys. We requested, via WHO and its regional offices, from ministries of health and other national health and statistical agencies to identify and access population-based surveys. Requests were also sent via the World Heart Federation to its national partners. We made a similar request to the co-authors of an earlier pooled analysis of cardiometabolic risk factors,[1-4] and invited them to reanalyse data from their studies and join NCD-RisC. NCD-RisC database is continuously updated through the

above routes and through periodic requests to NCD-RisC members to help identify new data sources.

Anonymised individual record data from sources included in NCD-RisC were reanalysed by the Pooled Analysis and Writing Group or by data holders according to a common protocol. Within each survey, we included participants aged 18 years and older who were not pregnant. We dropped participants with implausible SBP (defined as SBP <70 mmHg or SBP >270 mmHg; 0.09% of all SBP data) or DBP (DBP <50 mmHg or DBP >150 mmHg; 0.07% of all DBP data).

We calculated mean SBP, mean DBP and prevalence of raised blood pressure defined as systolic blood pressure (SBP) ≥140 mmHg or diastolic blood pressure (DBP) ≥90 mmHg, and associated standard errors and sample sizes, by sex and age group (18-19 years, 20-29 years, followed by 10-year age groups and 80+ years). All analyses incorporated appropriate sample weights and complex survey design in calculating age-sex-specific means and prevalences when applicable. To ensure summaries were prepared according to the study protocol, the Pooled Analysis and Writing Group provided computer code to NCD-RisC members who requested assistance. All submitted data were checked by at least two independent members of the Pooled Analysis and Writing Group. Questions and clarifications were discussed with NCD-RisC members and resolved before data were incorporated in the database.

Finally, we obtained data not accessed through the above routes by extracting from published reports of all additional national health surveys identified through the above-described strategies, four sub-national STEPS surveys, 12 CINDI surveys, eight MONICA surveys

which were not deposited in MONICA Data Centre. We restricted the extractions to those reports that had reported data by sex and in age groups no wider than 20 years. We also used data from a previous pooling study[2] when such data did not overlap with those accessed through the above routes.

Secondary search for additional data sources

To identify any major sources not accessed through the above routes, we searched Medline (via PubMed) for articles published between 1st January 1950 and 19th February 2014 using the search terms ("blood pressure"[Mesh:NoExp] OR "hypertension"[Mesh:NoExp]) AND ("Humans"[Mesh]) (search for adults for articles published between 1980 and 2009 was done in a previous analysis;[2] those for children for all years and for adults for years prior to 1980 and after 2009 were done here). We excluded the USA from the search because multiple nationally representative health examination surveys with individual records were publicly accessible and accessed before the search had begun. Articles identified through this search were screened according to the inclusion and exclusion criteria described above. The number of articles identified and retained is summarised in Appendix Figure 1. We contacted the corresponding authors of all eligible studies and invited them to join NCD-RisC. We did similar searches for other cardiometabolic risk factors including body mass index (BMI),[5] height,[6] diabetes,[7] and serum cholesterol. All eligible studies were invited to analyse data on all cardiometabolic risk factors and join NCD-RisC.

Data management

For each data source accessed through the primary and secondary data access process, we recorded the available information about the study population, period of measurement, sampling approach, and measurement methods. The information about study population was

used to establish that each data source was population-based, and to assess whether it covered the whole country, multiple sub-national regions, or one or a small number of communities, and whether it was rural, urban, or combined.

We carefully checked all data sources in terms of how they met our inclusion and exclusion criteria. We identified duplicate data sources by comparing studies from the same country and year. Additionally, NCD-RisC members received the list of all data sources in the database and were asked to ensure that the included data from their country met the inclusion criteria and that there were no duplicates. Data sources used in the analysis are listed in Appendix Table 2.

Appendix 2: Converting to primary blood pressure metrics

As described in Methods, our primary outcomes were mean SBP, mean DBP and prevalence of raised blood pressure defined as SBP ≥140 mmHg or DBP ≥90 mmHg, which were all available for 80% of data sources (84% of age-sex specific data points). Of the other 16% data points, from a previous global pooling[2] or extracted from published studies, 6% had mean SBP but not the other two primary outcomes; 6% had both mean SBP and mean DBP but not prevalence of raised blood pressure; 1% had only prevalence of raised blood pressure as defined above, and the remaining 3% had only a prevalence that differed from raised blood pressure defined above, e.g. SBP ≥160 mmHg or DBP ≥95 mmHg, or one that included use of antihypertensive medicine in the definition. In order to use these data, we developed conversion regressions to estimate the missing or all three primary outcomes from the data available.

The dependent variable in each regression was one of our primary outcomes, and the main independent variable was one or more of the above-mentioned metrics present in at least one study that did not report all three primary outcomes. The coefficients of these regressions were estimated from data sources with individual-level data which could be used to calculate all included terms, excluding data points for which there were fewer than 25 subjects. All regressions included terms for age, sex, country's income (natural logarithm of per-capita gross domestic product in 2011 international dollars), and the year of study, and regional random effects. Finally, we included interaction terms in the regressions if the interaction terms provided a better fit to the data as determined by the Bayesian Information Criteria (BIC).[8] In all cases, all sources of uncertainty in the conversion – including the sampling uncertainty of the original data, the uncertainty of the regression coefficients and random effects, and residuals – were carried forward by using repeated draws from their respective

distributions while accounting for the correlation among the uncertainties of regression coefficients and random effects. The regression coefficients and number of data points used to estimate the coefficients are shown in Appendix Table 3.

Appendix 3: Model covariates

As stated in the main paper, our statistical model included covariates that help predict blood pressure, including average number of years of education, proportion of national population living in urban areas, and a summary measure of availability of different food types for human consumption.

Data on proportion of national population living in urban areas were from the United Nations Population Division for all countries in our analysis. Data on average number of years of education were from the Institute of Health Metrics and Evaluation Global Educational Attainment for 190 countries. We used regional averages for American Samoa, Cook Islands, Greenland, St Kitts & Nevis, Niue, Nauru, Palau, French Polynesia, Tokelau and Tuvalu. The covariate on food availability was constructed from the food balance sheets of the Food and Agriculture Organization of the United Nations, using principal component analysis of 19 food items (alcoholic beverages, animal fats, cereals, eggs, fish & seafood, fruits (excluding wine), meat, milk (excluding butter), offals, oilcrops, other aquatic products, pulses, spices, starchy roots, stimulants, sugar & sweeteners, treenuts, vegetable oils and vegetables) as described elsewhere.[2,9,10]

Before the principal component analysis, the data for all country-item pairs were examined manually for outliers and implausible values. We removed six country-item-year data points as outliers, and 322 as implausible, mostly from the Maldives and Namibia. In addition, we removed all data for Occupied Palestinian Territory because the pre-1996 data were implausibly low. After data cleaning, a total of 2,283 country-item-year data points (~1% of all country-item-year data points for 179 countries with data) were missing. 38% of these were for non-fish aquatic products (e.g., aquatic mammals) and were set to zero as this item

was zero in most countries with data. Another 49% were situations in which a part of the time series was missing in such a way that missing years can be filled through linear interpolation or linear extrapolation using the regional linear trends. For the remaining 13%, regional means were used. Finally, we used regional means for 21 countries without data (American Samoa, Andorra, Bahrain, Bhutan, Cook Islands, DR Congo, Equatorial Guinea, Federated States of Micronesia, Greenland, Marshall Islands, Nauru, Niue, Oman, Palau, Papua New Guinea, Puerto Rico, Qatar, Singapore, Tokelau, Tonga and Tuvalu).

Appendix 4: Validation of statistical model

We tested how well our statistical model, which is described in the main text, predicts mean blood pressure and prevalence of raised blood pressure in countries and years without data in two different tests. In Test 1, we held out all data from 10% of countries with data (i.e., created the appearance of countries with no data where we actually had data). The countries whose data were withheld were randomly selected from the following three groups: data-rich (6 or more data sources, with at least one year of data after 2000), data-poor (1 data source), and average data availability (2 to 5 data sources, or 6 or more data sources with no data after 2000). We fitted the model to the data from the remaining 90% of countries and made estimates of the held-out observations. In Test 2, we assessed other patterns of missing data by holding out 10% of our data sources, again from a mix of data-rich, data-poor, and average-data countries, as defined above. For a given country, we either held out a random one third of the country's data or all of the country's 2000-2015 data to determine, respectively, how well we filled in the gaps for countries with intermittent data and how well we estimated in countries without recent data. We fitted the model to the remaining 90% of the dataset and made estimates of the held-out observations. We repeated each test five times, holding out a different subset of data in each repetition. In both tests, we calculated the differences between the held-out data and the estimates. We also checked the 95% credible intervals of the estimates; in a model with good external predictive validity, 95% of held-out values would be included in the 95% credible intervals.

Our statistical model performed well in the validation tests. The estimates of raised blood pressure prevalence were unbiased as evidenced with median errors that were very close to zero globally (ranging from -0.6 percentage points to 0.9 percentage points for the two sexes in the two tests), and less than or equal to ±4 percentage points in every subset of withheld

data except the -10.0 and -4.7 percentage points differences in Test 1 in South Asia for men and women respectively, and 4.9 percentage points difference in Test 1 for women in Central and Eastern Europe (Appendix Table 4). The 95% credible intervals of estimated prevalence covered 96-98% of true data, which implies good estimation of uncertainty; among all subgroups of data, coverages were ≥90% for both sexes. Median absolute errors ranged from 5.1 to 7.2 percentage points overall, or 2.2 to 10.2 percentage points in all subsets of withheld data.

Median errors for mean SBP and DBP were also small, and ranged from 0.10 to 0.99 mmHg for mean SBP and -0.07 to 0.50 mmHg for mean DBP globally suggesting that our estimates of mean SBP and DBP are also on average unbiased (Appendix Table 4). They were small in all subsets of withheld data: less than ±3 mmHg for mean SBP, with two exceptions (women in high-income Asia Pacific, a super-region with only three countries one of which was held out, and in Central and Eastern Europe) in Test 1; less than or equal to ±2 mmHg for mean DBP, also with two exceptions in Test 1 (men in South Asia and women in Central and Eastern Europe). Median absolute errors ranged from 3.5 to 4.4 mmHg for mean SBP and 2.2 to 2.8 mmHg for mean DBP overall, or less than 6 mmHg for mean SBP (except 8.3 mmHg in high-income Asia Pacific for women in Test 1) and less than or equal to 3.6 mmHg for mean DBP (except 4.3 mmHg for men in South Asia and 4.0 mmHg for women in Central and Eastern Europe in Test 1) in all subsets. Coverage globally for SBP was 86-88% in Test 1 and 74-77% in Test 2. In subsets, coverage ranged from 66% to 96%, but was mostly >75%, with coverage generally lower in Test 2 than Test 1. For DBP, global coverage was 87-88% in Test 1 and 78-79% in Test 2. In subsets, coverage ranged from 72% to 98%, but was mostly >80%, with coverage again generally lower in Test 2 than Test 1.

For comparison, median absolute differences between pairs of nationally representative surveys done in the same country and in the same year were 2.1 mmHg for SBP, 1.5 mmHg for DBP and 3.2 percentage points for prevalence of raised blood pressure, indicating that our estimates perform as well as running two parallel surveys in the same country and year.

Nonetheless the prediction errors were slightly larger and coverage lower than similar analyses for BMI and height (but not compared to diabetes),[5-7] likely because blood pressure varies more across countries in the same region that BMI and height, and because its trends over time are more complex and may include periods when blood pressure is unchanged preceding or following rises or declines in the same country. This heterogeneity makes prediction in countries and years without data harder than in the case of BMI and height, where there has been more steady change in most countries and where within-region variation is smaller.

Appendix Table 1: List of analysis regions and "super-regions", and countries in each region. The hierarchical structure of the statistical model consisted of country, region, super-region, and world.

Super-region	Region
**Sub-Saharan Africa (48)**	**Central Africa (6)**: Angola, Central African Republic, Congo, DR Congo, Equatorial Guinea, Gabon
	**East Africa (17)**: Burundi, Comoros, Djibouti, Eritrea, Ethiopia, Kenya, Madagascar, Malawi, Mauritius, Mozambique, Rwanda, Seychelles, Somalia, Sudan, Tanzania, Uganda, Zambia
	**Southern Africa (6)**: Botswana, Lesotho, Namibia, South Africa, Swaziland, Zimbabwe
	**West Africa (19)**: Benin, Burkina Faso, Cabo Verde, Cameroon, Chad, Cote d'Ivoire, Gambia, Ghana, Guinea, Guinea Bissau, Liberia, Mali, Mauritania, Niger, Nigeria, Sao Tome and Principe, Senegal, Sierra Leone, Togo
**Central Asia, Middle East and North Africa (28)**	**Central Asia (9)**: Armenia, Azerbaijan, Georgia, Kazakhstan, Kyrgyzstan, Mongolia, Tajikistan, Turkmenistan, Uzbekistan
	**Middle East and North Africa (19)**: Algeria, Bahrain, Egypt, Iran, Iraq, Jordan, Kuwait, Lebanon, Libya, Morocco, Occupied Palestinian Territory, Oman, Qatar, Saudi Arabia, Syrian Arab Republic, Tunisia, Turkey, United Arab Emirates, Yemen
**South Asia (6)**	**South Asia (6)**: Afghanistan, Bangladesh, Bhutan, India, Nepal, Pakistan
**East and Southeast Asia (16)**	**East Asia (4)**: China, China (Hong Kong SAR), North Korea, Taiwan
	**Southeast Asia (12)**: Brunei Darussalam, Cambodia, Indonesia, Lao PDR, Malaysia, Maldives, Myanmar, Philippines, Sri Lanka, Thailand, Timor-Leste, Viet Nam
**Oceania (17)**	**Polynesia and Micronesia (13)**: American Samoa, Cook Islands, French Polynesia, Kiribati, Marshall Islands, Micronesia (Federated States of), Nauru, Niue, Palau, Samoa, Tokelau, Tonga, Tuvalu
	**Melanesia (4):** Fiji, Papua New Guinea, Solomon Islands, Vanuatu
**High-income Asia Pacific (3)**	**High-income Asia Pacific (3)**: Japan, Singapore, South Korea
**Latin America and Caribbean (35)**	**Andean Latin America (3)**: Bolivia, Ecuador, Peru
	**Caribbean (18)**: Antigua and Barbuda, Bahamas, Barbados, Belize, Bermuda, Cuba, Dominica, Dominican Republic, Grenada, Guyana, Haiti, Jamaica, Puerto Rico, Saint Kitts and Nevis, Saint Lucia, Saint Vincent and the Grenadines, Suriname, Trinidad and Tobago
	**Central Latin America (9)**: Colombia, Costa Rica, El Salvador, Guatemala, Honduras, Mexico, Nicaragua, Panama, Venezuela
	**Southern Latin America (5)**: Argentina, Brazil, Chile, Paraguay, Uruguay
**High-income Western countries (27)**	**High-income English-speaking countries* (6)**: Australia, Canada, Ireland, New Zealand, United Kingdom, United States of America
	**North Western Europe (12)**: Austria, Belgium, Denmark, Finland, Germany, Greenland, Iceland, Luxembourg, Netherlands, Norway, Sweden, Switzerland
	**South Western Europe (9)**: Andorra, Cyprus, France, Greece, Israel, Italy, Malta, Portugal, Spain

**Central and Eastern Europe (20)**	**Central Europe (13)**: Albania, Bosnia and Herzegovina, Bulgaria, Croatia, Czech Republic, Hungary, Macedonia (TFYR), Montenegro, Poland, Romania, Serbia, Slovakia, Slovenia
	**Eastern Europe (7)**: Belarus, Estonia, Latvia, Lithuania, Moldova, Russian Federation, Ukraine

* Although high-income English-speaking countries are geographically separated, they exhibit remarkably similar trends in cardiometabolic risk factors and outcomes.[5-7] They were therefore grouped together so that the statistical model shares information amongst them more than it does with other countries that are geographically closer but epidemiologically more distinct.

We did not have data on population by age group for American Samoa, Bermuda, French Polynesia, Greenland, and Tokelau. Country-specific estimates were made but were not used in calculation of regional and global prevalences because the latter requires weighting by age-specific population.

Appendix Table 2: Data sources used in the analysis.

	Country	Data years	Survey/Study name/Citation	Level of representative-ness	Rural, urban, or both	Age range as used for global analysis		Sample size		Type of blood pressure measurement device[1]	Note
						Male	Female	Male	Female		
1	Albania	2001	Shapo et al., J Epidemiol Community Health 2003; 57: 734-9	Community	urban	25+	25+	535	585	Unknown	
2	Albania	2008-2009	DHS	National	both	18-49	18-49	2,445	3,086	Digital	
3	Algeria	2003	STEPS	Community	both	25-64	25-64	1,612	2,450	Digital	
4	Algeria	2007-2009	The ISOR (InSulino-resistance in ORan) study	Community	urban	30-64	30-64	377	405	Standard	
5	American Samoa	1976-1978	McGarvey, Am J Clin Nutr 1991;53: 1586S-94S	National	both	18+	18+	859	1,220	Standard	
6	American Samoa	1994	McGarvey, Pac Health Dialog 2001; 8: 157-62	National	both	25+	25-79	167	242	Standard	
7	American Samoa	2004	STEPS	National	both	25-64	25-64	950	1,062	Digital	
8	Argentina	1985-1986	INTERSALT	Community	urban	20-59	20-59	100	100	Random-zero	
9	Argentina	1997	de Sereday et al., Diabetes Metab 2004; 30: 335-9	Subnational	urban	20-59	20-59	834	1,097	Unknown	
10	Argentina	2004-2005	CArdiovascular Risk factors Multiple Evaluation in Latin America (CARMELA)	Community	urban	25-64	25-64	734	748	Standard	
11	Argentina	2005	Encuensta Nacional de Nutricion y Salud 2005	National	both		18-49		4,778	Standard	
12	Argentina	2008-2011	The VELA Project	Community	rural	20+	20+	134	252	Digital	
13	Argentina	2011-2012	Detection and follow-up of cardiovascular disease and risk factors in the Southern Cone of Latin America. The CESCAS I Study	Community	urban	35-74	35-74	1,589	2,397	Standard	
14	Armenia	2005	DHS	National	both	18-49	18-49	1,011	5,510	Digital	
15	Australia	1975	Busselton Health Study	Community	urban	18-84	18+	373	420	Standard	
16	Australia	1978	Busselton Health Study	Community	urban	18-84	18+	462	554	Standard	
17	Australia	1980	Risk Factor Prevalence Surveys	National	urban	25-65	25-65	2,764	2,839	Standard	
18	Australia	1981	Busselton Health Study	Community	urban	18-84	18+	420	482	Standard	
19	Australia	1983	MONICA, Newcastle	Subnational	urban	35-64	35-64	1,216	1,244	Random-zero	
20	Australia	1983	MONICA, Perth inner	Community	urban	25-64	25-64	861	921	Standard	
21	Australia	1983	Risk Factor Prevalence Surveys	National	urban	25-64	25-64	3,733	3,862	Standard	
22	Australia	1988-1989	Dubbo Study of Australian Elderly	Community	urban	59+	59+	878	1,222	Standard	
23	Australia	1988-1989	MONICA, Newcastle	Subnational	urban	35-64	35-64	672	661	Random-zero	
24	Australia	1988-1989	MONICA, Newcastle	Community	urban	25-34	25-34	70	83	Random-zero	
25	Australia	1988-1989	MONICA, Perth inner	Community	urban	25-64	25-64	404	405	Standard	
26	Australia	1988-1989	MONICA, Perth outer	Community	urban	25-64	25-64	412	419	Standard	
27	Australia	1989	Risk Factor Prevalence Surveys 1989	National	urban	20-69	20-69	4,551	4,727	Standard	
28	Australia	1990-1991	Canberra-Queanbeyan Longitudinal Study of the Elderly	Community	urban	70+	70+	454	380	Standard	
29	Australia	1992-1993	Australia Longitudinal Study of Ageing	Community	urban	65+	65+	842	771	Standard	
30	Australia	1993	Blue Mountain Eyes Study II; Simons et al., Med J Aust 2005; 182: 219-22	Community	urban	50+	50+	659	986	Unknown	
31	Australia	1994	MONICA, Newcastle	Subnational	urban	35-64	35-64	637	688	Random-zero	
32	Australia	1994	MONICA, Perth inner	Community	urban	25-64	25-64	363	350	Standard	
33	Australia	1994	MONICA, Perth outer	Community	urban	25-64	25-64	375	387	Standard	
34	Australia	1995-1996	National Nutrition Study	National	both	16+	16+	5,227	5,700	Standard	2
35	Australia	1996-1998	Western Australian AAA Screening Program	Community	urban	65-84		12,202		Standard	
36	Australia	1998	Blue Mountain Eyes Study II; Simons et al., Med J Aust 2005; 182: 219-22	Community	urban	50+	50+	736	1,089	Unknown	
37	Australia	1999-2000	The Australian Diabetes, Obesity and Lifestyle Study 1999-2000	National	both	25+	25+	5,019	6,112	Unknown	
38	Australia	1999-2003	North West Adelaide Health Study	Community	urban	18+	18+	1,932	2,123	Standard	
39	Australia	2004-2005	The Australian Diabetes, Obesity and Lifestyle Study 2004-2005	National	both	30+	30+	2,885	3,478	Digital	
40	Australia	2004-2006	North West Adelaide Health Study	Community	urban	20+	20+	1,515	1,669	Standard	
41	Australia	2008-2010	North West Adelaide Health Study	Community	urban	24+	24+	1,169	1,317	Standard	
42	Australia	2011-2012	Australian Health Survey	National	both	20+	20+	9,749	10,779	Digital	
43	Australia	2012	The Australian Diabetes, Obesity and Lifestyle Study 2012	National	both	37+	37+	2,047	2,531	Digital	
44	Austria	1986	CINDI	Community	both	25-64	25-64	647	700	Unknown	
45	Austria	1991	CINDI	Subnational	both	25-64	25-64	701	744	Standard	
46	Austria	1992	Vorarlberg Health Monitoring and Promotion Programme	Subnational	rural	18+	18+	14,161	18,826	Standard	
47	Austria	1998	Vorarlberg Health Monitoring and Promotion Programme	Subnational	rural	18+	18+	16,154	20,917	Standard	
48	Austria	2004	Vorarlberg Health Monitoring and Promotion Programme	Subnational	rural	18+	18+	20,161	23,893	Standard	
49	Azerbaijan	2006	DHS	National	both	18-59	18-49	2,259	7,254	Digital	
50	Bahrain	1995-1996	National Population Register in Bahrain; Al-Mahroos et al., Int J Epidmiol 2000; 29:71-6	National	both	40-59	50-69	1,218	872	Standard	
51	Bangladesh	2011	DHS	National	both	30+	30+	3,905	3,952	Digital	
52	Bangladesh	2015	An assessment of BRAC Health Nutrition and Population Programme and benchmark survey of Sustainable Development Goal	National	rural	35+	35+	5,254	5,694	Standard	
53	Barbados	1990	Barbados Eye Studies; Hennis et al., Ophthalmology 2003; 110: 908-14	National	both	40-84	40-84	1,988	2,635	Unknown	
54	Barbados	2011-2013	Health of the Nation (HotN)	National	both	25+	25+	470	739	Digital	
55	Belarus	1985	CINDI	Community	both	35-64		4,154		Unknown	

	Country	Data years	Survey/Study name/Citation	Level of representative-ness	Rural, urban, or both	Age range as used for global analysis		Sample size		Type of blood pressure measurement device[1]	Note
						Male	Female	Male	Female		
56	Belgium	1983-1985	MONICA, Luxembourg	Community	urban	35-64	35-64	975	946	Random-zero	
57	Belgium	1984-1985	Belgian Interuniversity Research on Nutrition and Health	National	both	25-74	25-74	5,899	5,288	Random-zero	
58	Belgium	1985-1987	INTERSALT, Charleroi	Community	urban	20-59	20-59	82	75	Random-zero	
59	Belgium	1985-1986	INTERSALT, Ghent	Community	urban	20-59	20-59	100	100	Random-zero	
60	Belgium	1985-1987	MONICA, Charleroi	Community	urban	25-64	25-64	346	326	Random-zero	
61	Belgium	1985-1987	MONICA, Ghent	Community	urban	25-64	25-64	550	459	Random-zero	
62	Belgium	1985-1990	Flemish Study on Environment, Genes and Health Outcomes	Community	rural	20-90	20-90	659	696	Standard	
63	Belgium	1987-1990	MONICA, Charleroi	Community	urban	25-64	25-64	325	301	Random-zero	
64	Belgium	1988-1990	MONICA, Ghent	Community	urban	25-64	25-64	456	449	Random-zero	
65	Belgium	1990-1993	MONICA, Charleroi	Community	urban	25-64	25-64	337	332	Random-zero	
66	Belgium	1990-1992	MONICA, Ghent	Community	urban	25-64	25-64	509	475	Random-zero	
67	Belgium	1991-1994	Flemish Study on Environment, Genes and Health Outcomes	Community	rural	26-88	26-88	394	416	Standard	
68	Belgium	1992-1995	Flemish Study on Environment, Genes and Health Outcomes	Community	rural	27-89	27-89	298	312	Standard	
69	Belgium	1994-1996	BIRNH Elderly: Belgian Interuniversity Research on Nutrition and Health in the Elderly	National	both	65-89	65-89	1,159	980	Random-zero	
70	Belgium	1996-1998	Flemish Study on Environment, Genes and Health Outcomes	Community	rural	18-84	18-84	354	347	Standard	
71	Belgium	1998	Flemish Study on Environment, Genes and Health Outcomes	Community	rural	32-86	32-86	320	359	Standard	
72	Belgium	1998-2000	Flemish Study on Environment, Genes and Health Outcomes	Community	rural	18-79	18-79	199	196	Standard	
73	Belgium	1999-2001	Flemish Study on Environment, Genes and Health Outcomes	Community	rural	18-81	18-79	213	227	Standard	
74	Belgium	2001	Flemish Study on Environment, Genes and Health Outcomes	Community	rural	18-78	18-78	230	208	Standard	
75	Belgium	2002-2003	Flemish Study on Environment, Genes and Health Outcomes	Community	rural	18-81	18-81	169	181	Standard	
76	Belgium	2003	The European Male Ageing Study (EMAS)	Community	both	40+		447		Digital	
77	Belgium	2002-2005	Flemish Study on Environment, Genes and Health Outcomes	Community	rural	18-88	18-88	391	404	Standard	
78	Belgium	2005-2008	Flemish Study on Environment, Genes and Health Outcomes	Community	rural	18-89	18-89	449	456	Standard	
79	Belgium	2008	The European Male Ageing Study (EMAS)	Community	both	40+		385		Digital	
80	Belgium	2009-2013	Flemish Study on Environment, Genes and Health Outcomes	Community	rural	20-88	20-88	330	335	Standard	
81	Belgium	2010-2015	Flemish Study on Environment, Genes and Health Outcomes	Community	rural	18-87	18-87	389	410	Standard	
82	Belize	2004-2005	CAMDI	National	both	20+	20+	999	1,434	Digital	
83	Benin	2007	STEPS	Community	urban	25-64	25-64	954	1,468	Digital	
84	Benin	2008	STEPS	National	both	25-64	25-64	3,445	3,383	Digital	
85	Benin	2011-2012	DHS	National	both	30-64	18-49	2,681	4,200	Digital	
86	Bhutan	2007	STEPS	Community	urban	25-74	25-74	1,131	1,328	Digital	
87	Bhutan	2014	STEPS	National	both	18-69	18-69	1,071	1,680	Digital	
88	Bolivia	2005-2007	Cardiovascular and metabolic syndrome risk assessment of Bolivian school children and adolescents - Relationships to obesity, diabetes, income, food intake and physical activity	National	both	18-18	18-18	136	134	Digital	
89	Bosnia and Herzegovina	2002	Non-communicable disease risk factor survey, Federation of B&H	Subnational	both	25-64	25-64	1,119	1,621	Standard	
90	Bosnia and Herzegovina	2012	Non-communicable disease risk factor survey, Federation of B&H	Subnational	rural	18+	18+	1,177	1,287	Standard	
91	Bosnia and Herzegovina	2012	Non-communicable disease risk factor survey, Federation of B&H	Subnational	urban	18+	18+	589	706	Standard	
92	Botswana	2007	STEPS	National	both	25-64	25-64	1,266	2,600	Digital	
93	Botswana	2014	STEPS	National	both	18-69	18-69	1,238	2,516	Digital	
94	Brazil	1987	de Lolio, Arq Bras Cardiol 1990; 55: 167-73	Community	urban	25-74	25-74	608	620	Unknown	
95	Brazil	1991	Fornes et al., Rev Saude Publica 2002; 36: 12-8	Community	urban	25+	25+	388	546	Unknown	
96	Brazil	1992	EPIDOSO; Ramos et al., Rev Saude Publica 1998; 32: 397-407	Community	urban	65+	65+	271	474	Unknown	
97	Brazil	1995-1996	Cohort study from Porto Alegre	Community	both	18+	18+	490	596	Standard	
98	Brazil	1996-1997	The Bambui Cohort Study of Ageing	Community	urban	18+	18+	947	1,367	Standard	
99	Brazil	1998	Frietas et al., Arq Bras Cardiol 2001; 77: 9-21	Subnational	urban	18-99	18-99	286	400	Unknown	
100	Brazil	1999	Lessa et al., Arq Bras Cardiol 2006; 87: 747-56	Community	urban	20+	20+	609	830	Unknown	
101	Brazil	2000	1982 Pelotas Birth Cohort	Community	urban	18-18		2,228		Standard	
102	Brazil	1999-2000	Pelotas cross-sectional survey	Community	urban	20-69	20-69	844	1,116	Standard	
103	Brazil	2002	Marcopito et al., Rev Saude Publica 2005; 39: 738-45	Community	urban	15-59	15-59	978	1,125	Unknown	2
104	Brazil	2003	Pereira et al., Cad Saude Publica 2007; 23: 2363-74	Community	urban	18-59	18-59	243	322	Unknown	
105	Brazil	2003	Hartmann et al., Cad Saude Publica 2007; 23: 1857-66	Community	urban		20-60		1,020	Unknown	
106	Brazil	2004	Marquezine et al., Int J Cardiol 2008; 129: 259-65	Community	urban	24-64	24-64	710	851	Unknown	
107	Brazil	2004	de Castro et al., Arq Bras Cardiol 2007; 88: 334-9	Community	urban	18+	18+	131	154	Unknown	
108	Brazil	2003-2005	São Paulo Ageing & Health Study	Community	urban	65+	65+	782	1,200	Digital	
109	Brazil	2004-2006	Hearts of Brazil	National	urban	18-79	18+	571	650	Standard	
110	Brazil	2004-2005	1982 Pelotas Birth Cohort	Community	urban	23-23	23-23	2,209	2,083	Digital	
111	Brazil	2006	ATITUDE	Subnational	both	18-21	18-21	1,195	1,553	Standard	

	Country	Data years	Survey/Study name/Citation	Level of representative-ness	Rural, urban, or both	Age range as used for global analysis		Sample size		Type of blood pressure measurement device[1]	Note
						Male	Female	Male	Female		
112	Brazil	2006-2007	SOFT study	Community	urban	20+	20+	665	1,038	Digital	
113	Brazil	2008	The Bambui Cohort Study of Ageing	Community	urban	71+	71+	254	475	Standard	
114	Brazil	2009	EPIFLORIPA Adults	Community	urban	25-64	25-64	633	796	Unknown	
115	Brazil	2009-2010	Condições de Saúde de Idosos de Florianópolis - EpiFloripa Idoso	Community	urban	60+	60+	610	1,070	Digital	
116	Brazil	2011	ATITUDE	Subnational	both	18-19	18-19	685	840	Digital	
117	Brazil	2010-2013	Baependi Heart Study	Community	rural	18+	18+	609	885	Digital	
118	Brazil	2011-2012	The 1993 Pelotas (Brazil) Birth Cohort: 18 years follow-up	Community	urban	18-19	18-19	1,962	1,997	Digital	
119	Brazil	2012-2013	Pelotas 1982 Birth Cohort	Community	urban	30-30	30-30	1,772	1,848	Digital	
120	Brazil	2012-2013	Prevalence of Leptin Polymorphism Gln223Arg	Community	urban	18+	18+	282	526	Digital	
121	Bulgaria	1983	Pavlov et al., Vutr Boles 1988; 27: 55-60	Community	rural	31+	31+	225	450	Unknown	
122	Bulgaria	1985	CINDI	Community	both	25-64	25-64	1,716	3,200	Unknown	
123	Burkina Faso	2013	STEPS	National	both	25-64	25-64	2,231	2,255	Digital	
124	Cabo Verde	2007	STEPS	National	both	25-64	25-64	663	1,076	Digital	
125	Cambodia	2010	STEPS	National	both	25-64	25-64	1,884	3,346	Digital	
126	Cameroon	1993	Cooper et al., Am J Public Health 1997; 87: 160-8	Subnational	both	25-74	25-74	1,088	1,125	Unknown	
127	Cameroon	1998-1999	ENHIP	Community	rural	18+	18+	486	701	Standard	
128	Cameroon	1998-1999	ENHIP	Community	urban	18+	18-79	430	538	Standard	
129	Cameroon	2000		Community	urban	35-54	35-54	448	567	Unknown	
130	Cameroon	2003	STEPS	Subnational	urban	18+	18+	3,379	5,048	Digital	
131	Cameroon	2007	Cameroon Burden of Diabetes - Second Survey	Subnational	urban	18+	18+	3,118	4,107	Digital	
132	Canada	1981	Canada Fitness Survey	National	both	20-65	20-65	4,699	5,310	Standard	
133	Canada	1983	McIntyre and Shah, CMAJ 1986; 134: 345-9	Community	rural	15+	15+	328	340	Unknown	2
134	Canada	1985	CINDI	Community	both	25-64	25-64	633	683	Unknown	
135	Canada	1985-1986	INTERSALT, StJohns	Community	urban	20-59	20-59	100	100	Random-zero	
136	Canada	1985-1988	MONICA, Halifax	Community	both	25-64	25-64	438	421	Standard	
137	Canada	1986-1992	Canada Heart Health Survey	National	both	18-74	18-74	11,353	11,737	Standard	
138	Canada	1995	CINDI	Community	urban	25-64	25-64	940	964	Unknown	
139	Canada	1995	MONICA, Halifax	Community	both	25-64	25-64	274	287	Standard	
140	Canada	2006	Ontario Survey of the Prevalence and Control of Hypertension; Leenen et al., CMAJ 2008; 178: 1441-9	Community	urban	20-79	20-79	1,452	1,540	Unknown	
141	Canada	2007-2011	Canadian Health Measures Survey	National	both	20-79	20-79	3,332	3,772	Digital	
142	Central African Republic	2010	STEPS	Community	both	25-64	25-64	1,863	1,992	Digital	
143	Chad	2008	STEPS	Community	urban	25-64	25-64	982	822	Digital	
144	Chile	1992-1993	Miquel et al., Gastroenterology 1998; 115: 937-946	Community	urban	18+	18+	660	1,032	Standard	
145	Chile	2000	Nervi F et al., J Hepatol 2006; 45: 299 - 305	Community	urban	20+	20+	335	625	Standard	
146	Chile	2003	Encuesta Nacional de Salud	National	both	18+	18+	1,551	1,848	Digital	
147	Chile	2004-2005	CArdiovascular Risk factors Multiple Evaluation in Latin America (CARMELA)	Community	urban	25-64	25-64	783	872	Standard	
148	Chile	2009-2010	Encuesta Nacional de Salud	National	both	18+	18+	1,890	2,876	Digital	
149	Chile	2011-2012	Detection and follow-up of cardiovascular disease and risk factors in the Southern Cone of Latin America. The CESCAS I Study	Community	urban	35-74	35-74	923	1,027	Standard	
150	China	1979-1982	East Beijing Study 1	Community	urban	20-79	20-79	382	399	Standard	
151	China	1979-1980	China National Hypertension Survey	National	both	15+	15+	1,902,339	2,109,789	Standard	2
152	China	1982	China National Nutrition Survey	National	both	20+	20+	6,222	7,145	Standard	
153	China	1983	Sino-MONICA Shanghai	Subnational	both	30-64	30-64	624	630	Standard	
154	China	1985	Chinese National Surveys on Students Constitution and Health	National	both	18-18	18-18	16,251	15,823	Standard	
155	China	1984-1985	Sino-MONICA Beijing	Subnational	both	25-64	25-64	816	857	Standard	
156	China	1986	INTERSALT, Beijing	Community	urban	20-59	20-59	100	100	Random-zero	
157	China	1986	INTERSALT, Nanning	Community	both	20-59	20-59	100	100	Random-zero	
158	China	1986	INTERSALT, Tianjin	Community	urban	20-59	20-59	100	100	Random-zero	
159	China	1988	Sino-MONICA Hebei	Subnational	both	25-64		800		Standard	
160	China	1988	Sino-MONICA Heilongjiang	Subnational	both	25-64	25-64	800	800	Standard	
161	China	1988	Sino-MONICA Henan	Subnational	both	25-64	25-64	345	427	Standard	
162	China	1988	Sino-MONICA Neimenggu	Subnational	both	25-64	25-64	397	400	Standard	
163	China	1988	Sino-MONICA Sichuan	Subnational	both	25-64	25-64	313	334	Standard	
164	China	1988	Sino-MONICA Shandong	Subnational	both	25-64	25-64	211	225	Standard	
165	China	1986-1989	Sino-MONICA Shanghai	Subnational	both	25-64	25-64	675	753	Standard	
166	China	1988-1990	East Beijing Study 2	Community	urban	20-84	20-84	138	150	Standard	
167	China	1989	China Health and Nutrition Study	National	both	20-45	20-45	2,383	2,570	Standard	3

	Country	Data years	Survey/Study name/Citation	Level of representative-ness	Rural, urban, or both	Age range as used for global analysis		Sample size		Type of blood pressure measurement device[1]	Note
						Male	Female	Male	Female		
168	China	1988-1989	Sino-MONICA Beijing	Subnational	both	25-64	25-64	703	863	Standard	
169	China	1989	Sino-MONICA Fujian	Subnational	both	25-64	25-64	179	191	Standard	
170	China	1988-1989	Sino-MONICA Jilin	Subnational	both	25-64	25-64	394	408	Standard	
171	China	1989	Sino-MONICA Jiangsu	Subnational	both	25-64	25-64	398	399	Standard	
172	China	1988-1989	Sino-MONICA Jiangxi	Subnational	both	25-64	25-64	379	386	Standard	
173	China	1988-1989	Sino-MONICA Liaoning	Subnational	both	25-64	25-64	728	734	Standard	
174	China	1991	China Health and Nutrition Study	National	both	18+	18+	4,092	4,512	Standard	3
175	China	1991	China National Hypertension Survey	National	both	15+	15+	448,929	500,441	Standard	2
176	China	1990-1991	China Prospective Study	National	both	40-79		221,080		Standard	
177	China	1991	Sino-MONICA Shanghai	Subnational	both	30-64	30-64	564	624	Standard	
178	China	1992-1993	Anzhen 02 Cohort Study	Community	urban	34-65	34-65	2,032	2,120	Standard	
179	China	1991-1992	Fangshan Cohort Study	Community	urban	34-86	34-86	879	1,746	Standard	
180	China	1992	Huashan Study	Community	urban	35-75	35-75	896	970	Standard	
181	China	1992	Sino-MONICA Sichuan	Subnational	both	25-64	25-64	628	536	Standard	
182	China	1993	China Health and Nutrition Study	National	both	18+	18+	3,901	4,280	Standard	3
183	China	1993	Sino-MONICA Anhui	Subnational	both	25-64	25-64	193	195	Standard	
184	China	1993	Sino-MONICA Beijing	Subnational	both	25-64	25-64	613	816	Standard	
185	China	1993	Sino-MONICA Jiangsu	Subnational	both	25-64	25-64	862	365	Standard	
186	China	1993	Sino-MONICA Liaoning	Subnational	both	25-64	25-64	493	500	Standard	
187	China	1995	Chinese National Surveys on Students Constitution and Health	National	both	18-18	18-18	8,562	8,392	Standard	
188	China	1997	China Health and Nutrition Study	National	both	18+	18+	4,184	4,424	Standard	3
189	China	1997	INTERMAP, Beijing	Community	rural	40-59	40-59	133	139	Random-zero	
190	China	1997	INTERMAP, Guangxi	Community	rural	40-59	40-59	140	138	Random-zero	
191	China	1997	INTERMAP, Shanxi	Community	rural	40-59	40-59	143	146	Random-zero	
192	China	1998	Guangdong Provincial Diabetic Survey	Subnational	rural	25-74	25-74	5,066	5,845	Unknown	
193	China	1998	Chinese Longitudinal Healthy Longevity Survey	National	both	80+	80+	3,447	5,244	Standard	
194	China	2000	Xu et al., Zhonghua Liu Xing Bing Xue Za Zhi 2004; 25: 33-5	Community	rural	35+	35+	389	442	Unknown	
195	China	2000	Jia et al., Obes Rev 2002; 3: 157-65	Community	urban	20+	20+	1,106	1,670	Unknown	
196	China	2000	Zhang et al., Wei Sheng Yan Jiu 2001; 30: 100-2	Community	rural	18-59	18-59	1,259	1,647	Unknown	
197	China	2000	China Health and Nutrition Study	National	both	18+	18+	4,527	4,937	Standard	3
198	China	2000	Chinese Longitudinal Healthy Longevity Survey	National	both	80+	80+	1,883	2,719	Standard	
199	China	2000	Chinese National Surveys on Students Constitution and Health	National	both	18-18	18-18	8,970	9,052	Standard	
200	China	2001	Shi et al., Zhonghua Liu Xing Bing Xue Za Zhi 2003; 24: 547-50	Community	rural	20+	20+	1,825	3,022	Unknown	
201	China	2000-2001	The International Collaborative Study of Cardiovascular Disease in Asia (InterASIA)	National	both	35-74	35-74	7,515	8,008	Standard	
202	China	2002	Chinese Longitudinal Healthy Longevity Survey	National	both	80+	80+	1,082	1,460	Standard	
203	China	2002	China National Nutrition and Health Survey	National	both	20+	20+	63,471	76,623	Standard	
204	China	2003	Fan et al., World J Gastroenterol 2008; 14: 2418-24	Subnational	both	25-84	25-74	5,518	7,768	Unknown	
205	China	2004	Beijing Child and Adolescent Metabolic Syndrome study	Community	both	18-18	18-18	256	260	Standard	
206	China	2004	China Health and Nutrition Study	National	both	18+	18+	4,402	4,802	Standard	3
207	China	2005	Le et al., BMC Public Health 2007; 7: 72	Subnational	rural	45+	45+	2,905	3,101	Unknown	
208	China	2005	Pang et al., Intern Med 2008; 47: 893-7	Community	rural	35+	35+	22,962	22,963	Unknown	
209	China	2005	Chinese Longitudinal Healthy Longevity Survey	National	both	80+	80+	405	582	Standard	
210	China	2005	Chinese National Surveys on Students Constitution and Health	National	both	18-18	18-18	10,091	10,155	Standard	
211	China	2006	CHEAPS Study; Dong et al., Indian J Med Res 2008; 128: 122-7	Subnational	rural	35+	35+	22,747	23,178	Unknown	
212	China	2006	Beijing Eye Study	Community	both	45+	45+	1,396	1,826	Standard	
213	China	2006	China Health and Nutrition Study	National	both	18+	18+	4,315	4,843	Standard	3
214	China	2007	Beijing Child and Adolescent Metabolic Syndrome study	Community	urban	18-18	18-18	20	18	Standard	
215	China	2008-2009	Chinese Longitudinal Healthy Longevity Survey	National	both	65+	65+	6,743	8,965	Standard	
216	China	2009	Study in Shenyang/Jinzhou/Anshan, Liaoning	Subnational	urban	25-84	25-84	5,757	6,877	Unknown	
217	China	2009	Study in Mianning, Sichuan	Subnational	rural	25-64	25-64	522	959	Unknown	
218	China	2009	China Health and Nutrition Study	National	both	18+	18+	4,529	4,964	Standard	3
219	China	2008-2010	Fangshan Family-based Ischemic Stroke Study in China (FISSIC) program	Community	rural	40+	40+	20,241	37,772	Standard	
220	China	2007-2010	SAGE	National	both	50+	50+	5,820	6,699	Digital	
221	China	2010	China Noncommunicable Disease Surveillance	National	both	18+	18+	45,078	53,460	Digital	
222	China	2010	Chinese National Surveys on Students Constitution and Health	National	both	18-18	18-18	8,952	8,913	Standard	
223	China	2011	Beijing Eye Study	Community	both	50+	50+	1,499	1,949	Standard	
224	China	2011	China Health and Nutrition Study	National	both	18+	18+	5,892	6,654	Standard	3

	Country	Data years	Survey/Study name/Citation	Level of representative-ness	Rural, urban, or both	Age range as used for global analysis		Sample size		Type of blood pressure measurement device[1]	Note
						Male	Female	Male	Female		
225	China	2013	Gobi Desert Children Eye Study	Community	both	18-21	18-19	28	25	Digital	
226	China	2012-2013	The Kailuan Study	Community	urban	18+	18+	81,496	21,590	Standard	
227	China (Hong Kong SAR)	1985-1986	Shatin New Town Study	Community	urban	70+	70+	285	698	Standard	
228	China (Hong Kong SAR)	1991	The Hong Kong study on Health, health risk and quality of life in the Chinese elderly cohort	Community	both	70+	70+	992	1,027	Standard	
229	China (Hong Kong SAR)	1995-1996	Hong Kong Cardiovascular Risk Factor Prevalence Study 1995-1996	National	both	25-74	25-74	1,412	1,483	Standard	
230	Colombia	1986	INTERSALT	Community	rural	20-59	20-59	96	95	Random-zero	
231	Colombia	2001	CINDI/CARMEN - Bucaramaga; Bautista et al., Eur J Cardiovasc Prev Rehabil 2006; 13: 769-75	Community	urban	25-64	25-64	623	1,227	Unknown	
232	Colombia	2002	CINDI/CARMEN - Bogota; Gomez et al., Boletin Epidemiológico Distrital 2004; 6: 11-29	Community	urban	25-74	25-74	319	568	Unknown	
233	Colombia	2004-2005	CArdiovascular Risk factors Multiple Evaluation in Latin America (CARMELA)	Community	urban	25-64	25-64	738	815	Standard	
234	Colombia	2007	Encuesta Nacional de Salud	National	both	18-69	18-69	5,510	7,744	Digital	
235	Colombia	2010	STEPS	Subnational	urban	18-64	18-64	881	1,188	Digital	
236	Comoros	2011	STEPS	National	both	25-64	25-64	1,568	3,591	Digital	
237	Congo	2004	STEPS	Community	urban	25-64	25-64	1,014	914	Digital	
238	Cook Islands	2003	STEPS	National	both	25-64	25-64	925	959	Digital	
239	Costa Rica	2004	CAMDI	Community	urban	20+	20+	522	896	Digital	
240	Costa Rica	2004-2006	Costa Rican Longevity and Healthy Aging Study Pre-1945 Cohort Wave 1	National	both	60+	60+	1,278	1,515	Digital	
241	Costa Rica	2006-2008	Costa Rican Longevity and Healthy Aging Study Pre-1945 Cohort Wave 2	National	both	62+	62+	1,060	1,261	Digital	
242	Costa Rica	2009-2010	Costa Rican Longevity and Healthy Aging Study Pre-1945 Cohort Wave 3	National	both	64+	64+	808	1,008	Digital	
243	Costa Rica	2010	Costa Rican National Cardiovascular Risk Factors Survey	National	both	20+	20+	1,016	2,618	Standard	
244	Costa Rica	2010-2011	Costa Rican Longevity and Healthy Aging Study 1945-1955 Cohort Wave 1	National	both	54-66	54-66	1,084	1,692	Digital	
245	Costa Rica	2012-2014	Costa Rican Longevity and Healthy Aging Study 1945-1955 Cohort Wave 2	National	both	56-68	56-68	898	1,508	Digital	
246	Cote d'Ivoire	2005	STEPS	National	rural	18-64	18-64	848	961	Digital	
247	Cote d'Ivoire	2005	STEPS	National	urban	18-64	18-64	986	1,293	Digital	
248	Croatia	2003	2003 CAHS - Croatian Health Survey project of the Ministry of Health and Social Welfare	National	both	18+	18+	2,878	6,162	Standard	
249	Croatia	2003-2004	School Health Survey	National	both	18-19	18-19	71	21	Standard	
250	Croatia	2005	Endemic Nephropathy and Arterial hypertension (ENAH)	Subnational	rural	18+	18+	266	401	Digital	
251	Croatia	2006-2008	The Cardiovascular risk factors in school age – intervention model development	National	both	18-20	18-19	190	202	Standard	
252	Croatia	2008	Endemic Nephropathy and Arterial hypertension (ENAH)	Subnational	rural	18+	18+	263	465	Digital	
253	Croatia	2010	Endemic Nephropathy and Arterial hypertension (ENAH)	Subnational	rural	18+	18+	258	406	Digital	
254	Croatia	2015	Endemic Nephropathy and Arterial hypertension (ENAH) Follow-up Study	Subnational	rural	20+	20+	225	462	Digital	
255	Cuba	1999-2000	The Survey on Health, Well-Being, and Aging in Latin America and the Caribbean (SABE)	Community	urban	60+	60+	645	1,087	Unknown	
256	Cuba	2010	National Risk Factor Survey	National	both	15+	15+	3,350	3,884	Standard	2
257	Cuba	2011	Non communicable disease risk factor in Cienfuegos	Community	urban	15-74	15-74	616	880	Standard	2
258	Czech Republic	1985	MONICA	National	both	25-64	25-64	1,245	1,309	Standard	
259	Czech Republic	1988	MONICA	National	both	25-64	25-64	1,357	1,411	Standard	
260	Czech Republic	1992	MONICA	National	both	25-64	25-64	1,134	1,209	Standard	
261	Czech Republic	1997-1998	post-Czech-MONICA	National	both	25-64	25-64	1,529	1,666	Standard	
262	Czech Republic	2000-2001	post-Czech-MONICA	National	both	25-64	25-64	1,631	1,690	Standard	
263	Czech Republic	2002-2005	Health, Alcohol and Psychosocial factors In Eastern Europe	Subnational	urban	45-69	45-69	3,240	3,888	Digital	
264	Czech Republic	2005	HELEN; Kubinova	National	both	45-54	45-54	762	1,065	Unknown	
265	Czech Republic	2007-2008	post-Czech-MONICA	National	both	25-64	25-64	1,718	1,861	Standard	
266	Denmark	1982	CCHS; Grubman and Mason, Rev Sci Tech 2002; 21: 589-600	Community	urban	20+	20+	5,677	7,014	Unknown	
267	Denmark	1981-1983	Obesity Research Group-Copenhagen City Heart Study 2	Subnational	both	22-62		1,138		Standard	
268	Denmark	1982-1984	MONICA, Glostrup	Community	urban	30-61	30-61	1,940	1,845	Standard	
269	Denmark	1985	Jennum et al., Eur J Epidemiol 1996; 12: 285-90	Community	urban	70-70	70-70	392	412	Unknown	
270	Denmark	1985	INTERSALT	Community	urban	20-59	20-59	99	100	Random-zero	
271	Denmark	1986-1987	MONICA, Glostrup	Community	urban	29-61	29-61	747	755	Standard	
272	Denmark	1991-1992	MONICA, Glostrup	Community	urban	29-61	29-61	808	816	Standard	
273	Denmark	1993	CCHS; Grubman and Mason, Rev Sci Tech 2002; 21: 589-600	Community	urban	20+	20+	4,220	5,439	Unknown	
274	Denmark	1992-1994	Obesity Research Group-Copenhagen City Heart Study 3	Subnational	both	32-72		922		Standard	
275	Denmark	1994	Hansen et al., J Hypertens 2006; 24: 2247-53	Community	urban	41-71	41-71	1,333	1,323	Unknown	
276	Denmark	1993-1997	EPIC Aarhus	Community	urban	50-65	50-65	8,424	8,712	Digital	
277	Denmark	1993-1997	EPIC Copenhagen	Community	urban	50-65	50-65	18,717	21,114	Digital	
278	Denmark	1998	Talleruphuus et al., Blood Press 2006; 15: 347-53	Community	both	70-80	70-80	1,336	1,410	Unknown	
279	Denmark	2006-2008	The Health2006 Cohort	Community	urban	18-71	18-71	1,553	1,918	Standard	
280	Denmark	2007-2008	The Danish Health Examination Survey	National	both	18+	18+	7,358	10,655	Digital	
281	Denmark	2009-2010	The European Youth Heart Study (EYHS)	Community	both	20-28	20-28	305	333	Digital	

	Country	Data years	Survey/Study name/Citation	Level of representative-ness	Rural, urban, or both	Age range as used for global analysis		Sample size		Type of blood pressure measurement device[1]	Note
						Male	Female	Male	Female		
282	Denmark	2012-2015	Danish study of Functional Disorders (DanFunD)	Subnational	urban	18-72	18-72	3,455	4,036	Standard	
283	Dominica	2007	STEPS	National	both	18-64	18-64	417	515	Digital	
284	Dominican Republic	1993	Aono et al., J Epidemiol 1997; 7: 238-43	National	both	20-69	20-69	737	1,149	Standard	
285	Dominican Republic	1997	EFRICARD	National	urban	25-84	25-84	2,042	3,964	Unknown	
286	DR Congo	1983	M'Buyamba-Kabangu et al., Am J Epidemiol 1986; 124: 957-68	Community	urban	20-59	20-59	179	223	Unknown	
287	DR Congo	2005	STEPS	Community	urban	18+	18+	677	1,031	Digital	
288	Ecuador	2004-2005	CArdiovascular Risk factors Multiple Evaluation in Latin America (CARMELA)	Community	urban	25-64	25-64	813	825	Standard	
289	Ecuador	2011-2013	Encuesta Nacional de Salud y Nutricion	National	both	18-59	18-59	13,237	18,621	Digital	
290	Egypt	1991-1993	The Egyptian National Hyperetension Study	National	both	25+	30+	2,930	3,317	Standard	
291	Egypt	2002	National Survey of Smoking, Obesity, Blood Pressure and Blood Glucose	National	both	20+	20+	1,958	2,519	Standard	
292	Egypt	2003-2004	Marzouk et al., Gut 2007; 56: 1105-10	Community	rural	25+	25+	316	446	Standard	
293	Egypt	2005	STEPS	National	both	18-65	18-65	4,255	4,022	Digital	
294	Egypt	2007-2009	Mostafa et al., Gut 2010; 59: 1135-40	Community	rural	35+	35+	638	840	Digital	
295	Egypt	2008	DHS	National	both	18-59	18-59	4,775	5,519	Digital	
296	Egypt	2011	STEPS	National	both	18-65	18-65	1,674	2,875	Digital	
297	Egypt	2015	DHS	National	both	18-59	18-59	6,644	7,801	Digital	
298	El Salvador	2004	CAMDI	Community	urban	20+	20+	397	811	Digital	
299	Eritrea	2004	STEPS	National	both	18-64	18-64	1,074	1,043	Digital	
300	Eritrea	2010	STEPS	National	both	25-74	25-74	1,713	4,290	Digital	
301	Estonia	1985	Abina et al., Blood Press 2003; 12: 111-21	Community	urban	20-54	30-54	2,477	851	Unknown	
302	Estonia	1992	CINDI	Community	both	25-64	25-64	1,193	862	Standard	
303	Estonia	1993	Abina et al., Blood Press 2003; 12: 111-21	Community	urban	20-54	20-54	921	678	Unknown	
304	Estonia	2000	Abina et al., Blood Press 2003; 12: 111-21	Community	urban	20-54	20-54	635	692	Unknown	
305	Estonia	2002	Estonian Biobank	National	both	18-79	18+	89	215	Digital	
306	Estonia	2003	Estonian Biobank	National	both	18+	18+	2,686	5,668	Digital	
307	Estonia	2003	The European Male Ageing Study (EMAS)	Community	both	40+		418		Digital	
308	Estonia	2004	Estonian Biobank	National	both	18+	18+	527	944	Digital	
309	Estonia	2007	Estonian Biobank	National	both	18+	18+	1,000	2,185	Digital	
310	Estonia	2008	Estonian Biobank	National	both	18+	18+	5,148	10,967	Digital	
311	Estonia	2008	The European Male Ageing Study (EMAS)	Community	both	40+		306		Digital	
312	Estonia	2009	Estonian Biobank	National	both	18+	18+	3,963	6,489	Digital	
313	Estonia	2010	Estonian Biobank	National	both	18+	18+	4,054	7,042	Digital	
314	Estonia	2011	Estonian Biobank	National	both	18+	18+	111	174	Digital	
315	Estonia	2012	Estonian Biobank	National	both	18+	18+	89	130	Digital	
316	Estonia	2013	Estonian Biobank	National	both	18+	18+	110	146	Digital	
317	Ethiopia	1983	Zein and Assefa, Ethiop Med J 1986; 24: 169-78	Community	rural	15-49	15-49	213	209	Unknown	2
318	Ethiopia	2004	STEPS; Tesfaye et al., Hum Hypertens 2007; 21: 28-37	Subnational	both	25-64	25-64	1,763	2,284	Unknown	
319	Ethiopia	2006	Surveillance of NCD Risk Factors in Addis Ababa (STEPS_Addis2006)	Subnational	urban	25-64	25-64	1,646	2,317	Digital	
320	Fiji	2002	STEPS	National	both	18-64	18-64	2,500	3,604	Digital	
321	Fiji	2011	STEPS	National	both	25-64	25-64	1,118	1,413	Digital	
322	Finland	1980	Young Finns Study 1980 rural	National	rural	18-18	18-18	121	149	Standard	
323	Finland	1980	Young Finns Study 1980 urban	National	urban	18-18	18-18	131	130	Standard	
324	Finland	1982	MONICA, North Karelia/Kuopio/Turku/Loimaa	Subnational	both	25-64	25-64	4,550	4,654	Standard	
325	Finland	1983	Young Finns Study 1983 rural	National	rural	18-21	18-21	166	198	Standard	
326	Finland	1983	Young Finns Study 1983 urban	National	urban	18-21	18-21	184	204	Standard	
327	Finland	1984	Finnish cohort of the FINE study	Community	rural	65-84		716		Standard	
328	Finland	1985-1986	INTERSALT, Joensuu	Community	urban	20-59	20-59	100	100	Random-zero	
329	Finland	1985	INTERSALT, Turku	Community	urban	20-59	20-59	100	100	Random-zero	
330	Finland	1986	Young Finns Study 1986 rural	National	rural	18-24	18-24	205	236	Random-zero	
331	Finland	1986	Young Finns Study 1986 urban	National	urban	18-24	18-24	262	331	Random-zero	
332	Finland	1987	Kuusisto et al., Stroke 1994; 25: 1157-64	Community	urban	65-74	65-74	470	828	Unknown	
333	Finland	1984-1989	Kuopio Ischaemic Heart Disease Risk factor Study	Subnational	both	42-61		2,628		Standard	
334	Finland	1987	MONICA, North Karelia/Kuopio/Turku/Loimaa	Subnational	both	25-64	25-64	2,898	3,152	Standard	
335	Finland	1989	Helsinki Ageing Study; Lindroos et al., Eur Heart J 1994; 15: 865-70	Community	urban	75-85	75-85	178	465	Unknown	
336	Finland	1989	Finnish cohort of the FINE study	Community	rural	70-89		462		Standard	
337	Finland	1990-1992	Oulu 35 Study	Community	urban	56-56	56-56	231	327	Standard	
338	Finland	1992	The National FINRISK Study	Subnational	both	25-64	25-64	2,847	3,201	Standard	

	Country	Data years	Survey/Study name/Citation	Level of representative-ness	Rural, urban, or both	Age range as used for global analysis		Sample size		Type of blood pressure measurement device[1]	Note
						Male	Female	Male	Female		
339	Finland	1991-1993	Kuopio Ischaemic Heart Disease Risk factor Study	Subnational	both	46-65		1,028		Standard	
340	Finland	1994	Finnish cohort of the FINE study	Community	rural	75-94		266		Standard	
341	Finland	1997	The National FINRISK Study	National	both	25-74	25-74	4,248	4,186	Standard	
342	Finland	1996-1998	Oulu 35 Study	Community	urban	60-63	60-63	233	338	Standard	
343	Finland	1996-1998	Savitaipale Study, Baseline	Community	rural	40-66	40-66	573	573	Standard	
344	Finland	2000	Finnish cohort of the FINE study	Community	rural	81-96		95		Standard	
345	Finland	1998-2001	Kuopio Ischaemic Heart Disease Risk factor Study	Subnational	both	53-73	53-73	854	920	Standard	
346	Finland	2000-2001	Health 2000 Survey	National	both	30+	30+	2,871	3,463	Standard	
347	Finland	2001	Young Finns Study 2001 rural	National	rural	24-39	24-39	344	391	Random-zero	
348	Finland	2001	Young Finns Study 2001 urban	National	urban	24-39	24-39	657	764	Random-zero	
349	Finland	2002	The National FINRISK Study	National	both	25-74	25-74	3,267	3,814	Standard	
350	Finland	2005	Mantyselka et al., Rheumatology (Oxford) 2008; 47: 1235-8	Community	rural	30-65	30-65	230	243	Unknown	
351	Finland	2007	The National FINRISK Study	National	both	25-74	25-74	2,926	3,310	Standard	
352	Finland	2005-2008	Kuopio Ischaemic Heart Disease Risk factor Study	Subnational	both	60-81	60-81	1,239	634	Standard	
353	Finland	2007	Oulu 35 Study	Community	urban	72-72	72-72	184	272	Standard	
354	Finland	2007	Young Finns Study 2007 rural	National	rural	30-45	30-45	379	438	Random-zero	
355	Finland	2007	Young Finns Study 2007 urban	National	urban	30-45	30-45	602	720	Random-zero	
356	Finland	2008	Control group for Finnish male former elite athletes	National	both	61+		207		Standard	
357	Finland	2007-2008	Savitaipale Study, Follow-up	Community	rural	51-75	51-75	433	484	Standard	
358	Finland	2011	Young Finns Study 2011 rural	National	rural	34-49	34-49	364	423	Random-zero	
359	Finland	2011	Young Finns Study 2011 urban	National	urban	34-49	34-49	501	634	Random-zero	
360	Finland	2012	The National FINRISK Study	National	both	25-74	25-74	2,771	3,042	Standard	
361	Finland	2011-2012	Health 2011 Survey	National	both	30+	30+	2,061	2,563	Standard	
362	France	1985-1987	MONICA, Strasbourg	Subnational	both	35-64	35-64	667	714	Standard	
363	France	1985-1987	MONICA, Strasbourg	Community	both	25-34	25-34	65	78	Standard	
364	France	1985-1987	MONICA, Toulouse	Subnational	both	35-64	35-64	678	645	Standard	
365	France	1986-1989	MONICA, Lille	Community	urban	25-64	25-64	882	737	Standard	
366	France	1988-1991	MONICA, Toulouse	Subnational	both	35-64		586		Random-zero	
367	France	1994-1996	MONICA, Toulouse	Subnational	both	35-64	35-64	609	566	Random-zero	
368	France	1995-1997	MONICA, Lille	Community	urban	36-67	36-66	598	594	Standard	
369	France	1995-1997	MONICA, Strasbourg	Subnational	both	35-64	35-64	527	533	Digital	
370	France	1999-2001	The Three city Study	Community	urban	65+	65+	3,511	5,412	Digital	
371	France	2001-2003	The Three city Study	Community	urban	66+	66+	3,087	4,870	Digital	
372	France	2003-2005	The Three city Study	Community	urban	68+	68+	2,692	4,371	Digital	
373	France	2004-2006	Monitoring National du Risque Artériel; National Monitoring of Arterial Risk (MONA LISA) Lille	Community	urban	35-75	35-75	797	796	Digital	
374	France	2005-2007	National Monitoring of Arterial Risk (MONA LISA) Bas-Rhin	Subnational	both	35-74	35-74	781	785	Digital	
375	France	2006-2008	The Three city Study	Community	urban	72+	72+	800	1,295	Digital	
376	France	2006-2007	Etude Nationale Nutrition Santé	National	both	18-74	18-74	792	1,330	Digital	
377	France	2008-2010	The Three city Study	Community	urban	73+	73+	801	1,404	Digital	
378	France	2010-2012	The Three city Study	Community	urban	76+	76+	561	993	Digital	
379	France	2011-2013	Enquête LIttorale Souffle Air Biologie EnvironnemenT (ELISABET) Dunkerque	Community	urban	40-64	40-64	749	798	Digital	
380	France	2011-2013	Enquête LIttorale Souffle Air Biologie EnvironnemenT (ELISABET) Lille	Community	urban	40-64	40-64	753	850	Digital	
381	French Polynesia	2010	STEPS	National	both	18-64	18-64	993	1,308	Digital	
382	Gabon	2009	STEPS	Community	urban	18-64	18-64	1,018	1,465	Digital	
383	Gambia	1997	van der Sande et al., Hum Hypertens 2000; 14: 489-96	Community	both	25-64	25-64	1,291	1,922	Unknown	
384	Gambia	1996-1997	Van der Sande et al., Trop Med Int Health 1997; 2: 1039-48	National	both	16+	16+	2,705	3,336	Digital	2
385	Gambia	2010	STEPS	National	both	25-64	25-64	1,643	1,953	Digital	
386	Georgia	1998	Grim et al., J Hum Hypertens 1999; 13: 243-7	Community	urban	40-69	40-69	92	249	Unknown	
387	Georgia	2010	STEPS	National	both	18-64	18-64	1,849	4,476	Digital	
388	Germany	1981	Hense et al., Int J Epidemiol 1986; 15: 513-8	Community	urban	30-69	30-69	1,032	1,163	Unknown	
389	Germany	1982	MBS; Koenig et al., Klin Wochenschr 1986; 64: 1229-36	Community	urban	30-69	30-69	861	966	Unknown	
390	Germany	1982	MONICA, Erfurt	Community	urban	25-64	25-64	106	103	Standard	
391	Germany	1982-1984	MONICA, Chemnitz	Community	urban	25-64	25-64	267	294	Standard	
392	Germany	1982-1984	MONICA, Zwickau	Community	urban	25-64	25-64	247	279	Standard	
393	Germany	1984	Herbold et al., Soz Praventivmed 1989; 34: 19-23	Community	urban	30-39		298		Unknown	
394	Germany	1984	German Cardiovascular Prevention Study (GCP) - National Health Survey 1984	Subnational	both	25-69	25-69	2,409	2,368	Standard	
395	Germany	1984-1985	MONICA, Berlin-Lichtenberg	Community	urban	25-64	25-64	597	639	Standard	

	Country	Data years	Survey/Study name/Citation	Level of representative-ness	Rural, urban, or both	Age range as used for global analysis		Sample size		Type of blood pressure measurement device[1]	Note
						Male	Female	Male	Female		
396	Germany	1984	MONICA, Bremen North/West	Community	urban	25-64	25-64	814	853	Random-zero	
397	Germany	1983-1984	MONICA, Halle County	Subnational	urban	25-64	25-64	1,111	1,172	Standard	
398	Germany	1982-1985	MONICA, Rest of Karl-Marx-Stadt County	Subnational	urban	25-64	25-64	591	656	Standard	
399	Germany	1982-1985	MONICA, Rest of DDR-MONICA	Subnational	urban	25-64	25-64	235	232	Standard	
400	Germany	1985-1986	INTERSALT, Cottbus	Community	urban	20-59	20-59	99	99	Random-zero	
401	Germany	1985-1986	INTERSALT, Heidelberg	Community	urban	20-59	20-59	97	99	Random-zero	
402	Germany	1984-1985	MONICA, Augsburg	Community	both	25-64	25-64	2,017	1,991	Random-zero	
403	Germany	1984-1986	MONICA, Cottbus County	Community	urban	25-64	25-64	657	739	Standard	
404	Germany	1983-1987	MONICA, Rhein-Neckar Region	Community	urban	25-64	25-64	1,486	1,608	Standard	
405	Germany	1986	CINDI	Community	urban	25-64	25-64	1,878	1,990	Unknown	
406	Germany	1985-1986	INTERSALT, Bernried	Community	urban	20-59	20-59	99	98	Random-zero	
407	Germany	1987-1988	MONICA, Erfurt	Community	urban	25-64	25-64	879	915	Standard	
408	Germany	1988	German Cardiovascular Prevention Study (GCP) - National Health Survey 1988	Subnational	both	25-69	25-69	2,647	2,684	Standard	
409	Germany	1988	MONICA, Berlin-Lichtenberg	Community	urban	25-64	25-64	689	727	Standard	
410	Germany	1988	MONICA, Bremen North/West	Community	urban	25-69	25-69	621	632	Random-zero	
411	Germany	1988	MONICA, Bremen Center/South/East	Community	urban	25-69	25-69	502	582	Random-zero	
412	Germany	1988	MONICA, Chemnitz	Community	urban	25-64	25-64	288	382	Standard	
413	Germany	1988	MONICA, Zwickau	Community	urban	25-64	25-64	193	250	Standard	
414	Germany	1988-1989	CINDI	Subnational	both	25-64	25-64	1,355	1,432	Standard	
415	Germany	1989-1990	MONICA, Cottbus County	Community	urban	25-64	25-64	542	529	Standard	
416	Germany	1988-1989	MONICA, Halle County	Subnational	urban	25-64	25-64	963	1,202	Standard	
417	Germany	1988-1989	MONICA, Rest of Karl-Marx-Stadt County	Subnational	urban	25-64	25-64	543	626	Standard	
418	Germany	1989-1990	MONICA, Augsburg	Community	both	25-64	25-64	1,952	1,997	Random-zero	
419	Germany	1991-1992	MONICA, Bremen North/West	Community	urban	25-69	25-69	598	672	Random-zero	
420	Germany	1991-1992	MONICA, Bremen Center/South/East	Community	urban	25-69	25-69	524	547	Random-zero	
421	Germany	1991-1992	CINDI	Subnational	both	25-64	25-64	1,338	1,430	Standard	
422	Germany	1991-1992	German Cardiovascular Prevention Study (GCP) - National Health Survey 1991	Subnational	both	25-69	25-69	2,622	2,686	Standard	
423	Germany	1991-1992	First National Examination of life conditions, Environment and Health in East Germany 1991/92	Subnational	both	25-69	25-69	1,051	1,160	Standard	
424	Germany	1991-1992	MONICA, Erfurt	Community	urban	25-64	25-64	588	574	Standard	
425	Germany	1993	STEPHY I; Trenkwalder et al., J Hypertens 1994; 12: 709-16	Community	rural	65+	65+	394	588	Unknown	
426	Germany	1993-1994	MONICA, Chemnitz	Community	urban	25-64	25-64	407	426	Standard	
427	Germany	1993-1994	MONICA, Zwickau	Community	urban	25-64	25-64	139	186	Standard	
428	Germany	1995	Bobak et al., Int J Epidemiol 1999; 28: 437-44	Community	urban	45-64		501		Unknown	
429	Germany	1994-1995	MONICA, Augsburg	Community	both	25-64	25-64	1,905	1,994	Random-zero	
430	Germany	1994-1998	EPIC Heidelberg	Community	urban	40-64	35-64	4,396	5,369	Digital	
431	Germany	1994-1998	EPIC Potsdam	Community	urban	40-64	35-64	9,541	15,239	Digital	
432	Germany	1997-1999	German National Health Interview and Examination Survey (GNHIES98)	National	both	18-79	18-79	3,445	3,616	Standard	
433	Germany	1997-2001	Study of Health in Pomerania (SHIP-0) baseline study	Subnational	both	20-80	20-80	2,112	2,187	Digital	
434	Germany	1999-2001	KORA S4 Study: Kooperative Research in the Region of Augsburg Survey 4	Community	both	24-75	24-75	2,082	2,161	Random-zero	
435	Germany	2000-2002	Epidemiological study of the chances of prevention, early recognition and optimal treatment of chronic diseases in an elderly population (ESTHER)	Subnational	both	50-75	50-75	4,315	5,306	Unknown	
436	Germany	2002-2006	Study of Health in Pomerania (SHIP-1) 5-year follow-up	Subnational	both	20-85	20-85	1,584	1,707	Digital	
437	Germany	2006-2008	KORA F4 Study: Kooperative Research in the Region of Augsburg Follow-Up of Survey 4	Community	both	32-82	31-82	1,482	1,591	Random-zero	
438	Germany	2008-2011	Epidemiological study of the chances of prevention, early recognition and optimal treatment of chronic diseases in an elderly population (ESTHER)	Subnational	both	58-84	58-84	1,297	1,443	Standard	
439	Germany	2008-2011	German Health Interview and Examination Survey for adults 2008-11 (DEGS1)	National	both	18-79	18-79	3,405	3,665	Digital	
440	Ghana	1998	Amoah et al., Diabetes Res Clin Pract 2002; 56: 197-205	Subnational	urban	25+	25+	1,857	2,875	Unknown	
441	Ghana	2001	Cappuccio et al., Hypertension 2004; 43: 1017-22	Community	both	40-75	40-75	385	628	Unknown	
442	Ghana	2006	STEPS	Community	urban	25-69	25-69	887	1,698	Digital	
443	Ghana	2007-2008	SAGE	National	both	50+	50+	2,215	2,034	Digital	
444	Ghana	2012	The Burden and Correlates of Hypertension in Rural Ghana: A Cross-Sectional Study	Subnational	rural	35+	35+	150	271	Standard	
445	Ghana	2013	Silent Crisis: Epidemic Hypertension in Rural West Africa	Subnational	rural	35+	35+	151	278	Digital	
446	Ghana	2012-2014	Research on Obesity and Diabetes among African Migrants (RODAM), control group	Subnational	rural	25+	25+	431	679	Digital	
447	Ghana	2012-2014	Research on Obesity and Diabetes among African Migrants (RODAM), control group	Subnational	urban	25-79	25-79	419	1,033	Digital	
448	Ghana	2014	DHS	National	both	18-59	18-49	3,841	7,916	Digital	
449	Greece	1986	Lindholm et al., Eur Heart J 1992; 13: 291-8	Community	rural	45-79	45-79	92	111	Unknown	
450	Greece	1991-1999	EPIC	National	both	19-86	20-86	11,119	15,885	Standard	

	Country	Data years	Survey/Study name/Citation	Level of representative-ness	Rural, urban, or both	Age range as used for global analysis		Sample size		Type of blood pressure measurement device[1]	Note
						Male	Female	Male	Female		
451	Greece	1997	Stergiou et al., Am J Hypertens 1999; 12: 959-65	Community	rural	25+	25+	266	362	Unknown	
452	Greece	2001	Karalis et al., BMC Public Health 2007; 7: 351	Community	rural	25+	25+	91	103	Unknown	
453	Greenland	2005-2010	Population Health Survey in Greenland	National	both	18+	18+	1,351	1,725	Digital	
454	Grenada	2011	STEPS	National	both	25-64	25-64	440	645	Digital	
455	Guatemala	1998	Stein et al., J Nutr 2002; 132: 2208-14	Community	rural	25-44	25-44	488	548	Unknown	
456	Guatemala	1998	Stein et al., J Nutr 2002; 132: 2208-14	Community	urban	25-44	25-44	154	230	Unknown	
457	Guatemala	2001-2002	CAMDI	Community	urban	20+	20+	454	940	Digital	
458	Guatemala	2003-2005	The Institute of Nutrition of Central America and Panama Nutrition Supplementation Trial Cohort	Community	both	25-41	25-41	265	304	Digital	
459	Guinea	2009	STEPS	Subnational	both	18-64	18-64	1,057	1,133	Digital	
460	Honduras	2003-2004	CAMDI	Community	urban	20+	20+	437	792	Digital	
461	Hungary	1982-1984	MONICA, Budapest	Community	urban	25-64	25-64	775	737	Standard	
462	Hungary	1982-1983	MONICA, Pecs	Community	urban	25-64	25-64	821	861	Standard	
463	Hungary	1985	CINDI	Community	urban	25-64	25-64	1,202	1,198	Unknown	
464	Hungary	1985	INTERSALT	Community	rural	20-59	20-59	100	100	Random-zero	
465	Hungary	1987	Biro, Acta Cardiol 1990; 45: 3-14	Community	both	18+	18+	308	491	Unknown	
466	Hungary	1987-1988	MONICA, Budapest	Community	urban	25-64	25-64	1,415	1,603	Standard	
467	Hungary	1987-1988	MONICA, Pecs	Community	urban	25-64	25-64	1,584	1,519	Standard	
468	Hungary	1994-1996	CINDI	Community	urban	25-64	25-64	693	1,150	Standard	
469	Hungary	2003	The European Male Ageing Study (EMAS)	Community	both	40+		429		Digital	
470	Hungary	2008	The European Male Ageing Study (EMAS)	Community	both	40+		349		Digital	
471	Iceland	1983	MONICA, Arnes County	Community	rural	25-64	25-64	388	450	Standard	
472	Iceland	1983	MONICA, Reykjavik	Subnational	urban	25-64	25-64	435	461	Standard	
473	Iceland	1985-1986	INTERSALT	Community	urban	20-59	20-59	100	100	Random-zero	
474	Iceland	1988-1989	MONICA, Arnes County	Community	rural	25-64	25-64	388	442	Standard	
475	Iceland	1988-1989	MONICA, Reykjavik	Subnational	urban	25-64	25-64	413	445	Standard	
476	Iceland	1993-1994	MONICA, Arnes County	Community	rural	25-64	25-64	422	484	Standard	
477	Iceland	1993-1994	MONICA, Reykjavik	Subnational	urban	25-64	25-64	443	449	Standard	
478	Iceland	2004	AGES Reykjavik Study; Mitchell et al., Hypertension 2008; 51: 1123-8	Community	urban	69+	69+	184	224	Unknown	
479	India	1986	INTERSALT	Community	urban	20-59	20-59	100	99	Random-zero	
480	India	1988-1989	Ramachandran et al., Diabetes Res Clin Pract 2002; 58:55-60	Community	urban	20-74	20-74	455	435	Standard	
481	India	1990	Beegom et al., Int J Cardiol 1995; 51: 183-91	Community	urban	25-64	25-64	737	760	Unknown	
482	India	1991-1997	Mumbai Cohort Study	Community	urban	35+	35+	88,658	59,515	Standard	
483	India	1995	Gupta et al., J Hum Hypertens 2003; 17: 535-40	Community	urban	20+	20+	1,406	797	Unknown	
484	India	1995	Malhotra et al., J Hum Hypertens 1999; 13: 467-72	Community	both	16-70	16-70	1,230	1,329	Unknown	2
485	India	1995	Ramachandran et al., Diabetes Res Clin Pract 1998; 42:181-6	Community	urban	20-74	20-74	1,061	1,093	Standard	
486	India	1995-1997	Aravind Comprehensice Eye Survey	Community	rural	40+	40+	2,200	2,672	Standard	
487	India	1995-1996	Epidemiology of blood pressure across cross-cultural populations of Visakhapatnam district, Andhra Pradesh, India	Community	rural	19-76	19-76	210	228	Standard	
488	India	1997	Ramachandran et al., Diabetes Res Clin Pract 1999; 44: 207-13	Community	rural	20-74	20-74	738	879	Standard	
489	India	1996-1999	Chennai Urban Population Study	Community	urban	20+	20+	557	705	Standard	
490	India	1999	Swami et al., Public Health 2002; 116: 45-9	Community	both	65+	65+	153	209	Unknown	
491	India	2000	Barucha and Kuruvilla, BMC Public Health 2003; 3: 1	Community	urban	20+	20+	1,099	1,316	Unknown	
492	India	2001	Gupta et al., BMC Cardiovasc Disord 2009; 9: 28	Community	urban	15-39	15-39	977	1,493	Unknown	2
493	India	1999-2002	New Delhi Birth Cohort	Community	urban	26-33	26-33	880	631	Digital	
494	India	2002	Gupta et al., J Hum Hypertens 2003; 17: 535-40	Community	urban	20+	20+	550	573	Unknown	
495	India	2001-2004	Chennai Urban Rural Epidemiology Study	Community	urban	20+	20+	1,095	1,252	Standard	
496	India	2002-2003	Blood Pressure epidemiology in tribal, rural and urban communities of Orissa with special reference to physical and social parameters	Community	rural	18-80	18-80	201	187	Standard	
497	India	2003-2005	WHO-ICMR NCD risk factor surveillance study	National	rural	15-69	15-69	6,432	6,842	Digital	2
498	India	2003-2005	WHO-ICMR NCD risk factor surveillance study	National	urban	15-69	15-69	6,553	6,727	Digital	2
499	India	2006	Ramachandran et al., Diabetes Care 2008; 31:893-8	Community	both	20+	20+	3,321	3,745	Standard	
500	India	2005-2006	Diet and Nutritional status of Rural population and Prevalnce of Hypertension	National	rural	20+	20+	11,920	13,702	Standard	
501	India	2005-2007	Prevalence of cardiovascular risk factors in rural Tamil Nadu	Community	rural	25-64	25-64	4,900	5,563	Digital	
502	India	2007	Kusuma et al., Asia Pac J Public Health 2009; 21: 497-507	Community	urban	25-64	25-64	179	192	Unknown	
503	India	2006-2008	Central India Eye and Medical Study	Community	rural	30+	30+	2,191	2,520	Digital	
504	India	2007-2008	Integrated Disease Surveillance Project Non-communicable Disease Risk Factors Survey	Subnational	both	15-64	15-64	2,719	3,499	Digital	2
505	India	2007-2008	Integrated Disease Surveillance Project Non-communicable Disease Risk Factors Survey	Subnational	both	15-64	15-64	1,720	3,136	Digital	2
506	India	2007-2008	Integrated Disease Surveillance Project Non-communicable Disease Risk Factors Survey	Subnational	both	15-64	15-64	2,857	2,996	Digital	2

	Country	Data years	Survey/Study name/Citation	Level of representative-ness	Rural, urban, or both	Age range as used for global analysis		Sample size		Type of blood pressure measurement device[1]	Note
						Male	Female	Male	Female		
507	India	2007-2008	Integrated Disease Surveillance Project Non-communicable Disease Risk Factors Survey	Subnational	both	15-64	15-64	3,084	3,007	Digital	2
508	India	2007-2008	Integrated Disease Surveillance Project Non-communicable Disease Risk Factors Survey	Subnational	both	15-64	15-64	2,282	2,185	Digital	2
509	India	2007-2008	Integrated Disease Surveillance Project Non-communicable Disease Risk Factors Survey	Subnational	both	15-64	15-64	2,060	3,017	Digital	2
510	India	2007-2008	Integrated Disease Surveillance Project Non-communicable Disease Risk Factors Survey	Subnational	both	15-64	15-64	2,144	3,283	Digital	2
511	India	2007-2009	Prevalence of NCD risk factor in people above 15 year in Rural area Nagpur using WHO STEP approach	Community	rural	15+	15+	1,971	1,811	Digital	2
512	India	2006-2009	New Delhi Birth Cohort	Community	urban	33-38	33-38	650	447	Digital	
513	India	2007-2008	SAGE	National	both	50+	50+	3,257	3,201	Digital	
514	India	2008-2010	ICMR India Diabetes Study	National	both	20+	20+	6,965	7,024	Digital	
515	India	2009-2010	Baseline Survey for the assessment of prevalence of risk factors of NCDs in Gandhinagar District	Community	rural	15-64	15-64	887	797	Digital	2
516	India	2009-2010	Baseline Survey for the assessment of prevalence of risk factors of NCDs in Gandhinagar District	Community	urban	15-64	15-64	898	907	Digital	2
517	India	2011-2012	Diet and Nutritional status of Rural population and Prevalnce of Hypertension	National	rural	18+	18+	22,075	27,244	Digital	
518	India	2012-2014	District Level Household and Facility Survey (DLHS) 4	National	both	18+	18+	402,994	476,021	Digital	
519	India	2013-2014	Annual Health Survey (AHS) - Clinical, Anthropometric & Bio-Chemical (CAB) Component	Subnational	both	18+	18+	84,281	99,883	Digital	
520	Indonesia	1997-1998	Indonesian Family Life Surveys	National	both	18+	18+	7,729	9,489	Digital	
521	Indonesia	2001	STEPS; Ng et al., Bull World Health Organ 2006; 84: 305-13	Community	both	15-74	15-74	1,502	1,461	Unknown	2
522	Indonesia	2001	STEPS/SURKESNAS	National	both	25+	25+	4,686	5,345	Unknown	
523	Indonesia	2000-2001	Indonesian Family Life Surveys	National	both	18+	18+	10,609	11,505	Digital	
524	Indonesia	2003	STEPS; Tesfaye et al., J Hum Hypertens 2007; 21: 28-37	Subnational	both	25-64	25-64	984	960	Unknown	
525	Indonesia	2006	NCD RFS; Soebardi et al., Acta Med Indones 2009; 41: 186-90	Community	urban	25-64	25-64	641	950	Unknown	
526	Indonesia	2007-2008	Indonesian Family Life Surveys	National	both	18+	18+	12,839	14,342	Digital	
527	Indonesia	2013	Population Health Basic Health Research 2013 (Riskesdas)	National	both	15+	15+	344,856	372,158	Digital	2
528	Iran	1990-1991	National Health Survey I	National	both	20+	20+	8,233	10,976	Standard	
529	Iran	1993	SarrafZadegan and AminiNik, J Hum Hypertens 1997; 11: 425-8	Subnational	urban	41-70	41-70	1,712	2,362	Unknown	
530	Iran	1993	SarrafZadegan and AminiNik, J Hum Hypertens 1997; 11: 425-8	Subnational	rural	41-70	41-70	67	115	Unknown	
531	Iran	1999-2000	National Health Survey II	National	both	20+	20+	12,429	15,175	Standard	
532	Iran	1999-2001	Tehran Lipid and Glucose Study	Community	both	18-80	18-80	6,238	6,538	Standard	
533	Iran	2001	Isfahan Healthy Heart Program, Arak rural	Community	rural	19+	19+	1,028	1,091	Standard	
534	Iran	2001	Isfahan Healthy Heart Program, Arak urban	Community	urban	19+	19+	2,085	2,127	Standard	
535	Iran	2001	Isfahan Healthy Heart Program, Isfahan rural	Community	rural	19+	19+	234	239	Standard	
536	Iran	2001	Isfahan Healthy Heart Program, Isfahan urban	Community	urban	19+	19+	1,779	1,927	Standard	
537	Iran	2001	Isfahan Healthy Heart Program, Najaf Abad rural	Community	rural	19+	19+	408	419	Standard	
538	Iran	2001	Isfahan Healthy Heart Program, Najaf Abad urban	Community	urban	19+	19-79	580	576	Standard	
539	Iran	2004	Persian Gulf Healthy Heart Study	Subnational	urban	25-64	25-64	1,615	1,528	Unknown	
540	Iran	2003-2004	Childhood and Adolescence Surveillance and Prevention of Adult Noncommunicable Disease (CASPIAN) R1	National	both	18-18	18-18	60	77	Standard	
541	Iran	2002-2005	Tehran Lipid and Glucose Study	Community	both	18-80	18-80	2,188	2,919	Standard	
542	Iran	2005	Provincial Non-Communicable Disease Surveillance Survey	National	both	18-64	18-64	38,081	37,564	Digital	
543	Iran	2004-2008	Golestan Cohort Study	Subnational	both	40-79	40-79	21,225	28,800	Standard	
544	Iran	2006	Provincial Non-Communicable Disease Surveillance Survey	National	both	18-65	18-65	14,180	14,029	Digital	
545	Iran	2007	Isfahan Healthy Heart Program, Arak rural	Community	rural	19+	19+	1,030	1,028	Standard	
546	Iran	2007	Isfahan Healthy Heart Program, Arak urban	Community	urban	19+	19+	1,429	1,366	Standard	
547	Iran	2007	Isfahan Healthy Heart Program, Isfahan rural	Community	rural	19+	19+	157	152	Standard	
548	Iran	2007	Isfahan Healthy Heart Program, Isfahan urban	Community	urban	19+	19+	1,340	1,339	Standard	
549	Iran	2007	Isfahan Healthy Heart Program, Najaf Abad rural	Community	rural	19-79	19+	255	254	Standard	
550	Iran	2007	Isfahan Healthy Heart Program, Najaf Abad urban	Community	urban	19+	19+	495	544	Standard	
551	Iran	2007	National Non-Communicable Disease Surveillance Survey	National	both	18-64	18-64	2,228	2,234	Digital	
552	Iran	2007	Provincial Non-Communicable Disease Surveillance Survey	National	both	18-64	18-64	13,859	13,826	Digital	
553	Iran	2005-2008	Tehran Lipid and Glucose Study	Community	both	18-80	18-80	2,496	3,269	Standard	
554	Iran	2008	Provincial Non-Communicable Disease Surveillance Survey	National	both	18-64	18-64	13,778	13,674	Digital	
555	Iran	2009	Provincial Non-Communicable Disease Surveillance Survey	National	both	18-64	18-64	13,985	13,766	Digital	
556	Iran	2009-2010	Childhood and Adolescence Surveillance and Prevention of Adult Noncommunicable Disease (CASPIAN) R3	National	both	18-18	18-18	484	502	Standard	
557	Iran	2008-2011	Tehran Lipid and Glucose Study	Community	urban	20+	20+	4,714	6,025	Standard	
558	Iran	2011	Provincial Non-Communicable Disease Surveillance Survey	National	both	18-69	18-69	4,106	5,771	Digital	
559	Iran	2011-2012	Childhood and Adolescence Surveillance and Prevention of Adult Noncommunicable Disease (CASPIAN) R4	National	both	18-18	18-18	287	270	Standard	
560	Iraq	2006	STEPS	National	both	25-64	25-64	1,946	2,557	Standard	
561	Ireland	1998	Survey of Lifestye, Attitudes and Nutritional in Ireland (SLAN)	National	both	18+	18+	124	296	Unknown	
562	Ireland	2002	Survey of Lifestye, Attitudes and Nutritional in Ireland (SLAN)	National	both	18+	18+	168	221	Unknown	
563	Ireland	2006-2007	Survey of Lifestye, Attitudes and Nutritional in Ireland (SLAN)	National	both	45-79	45-79	526	679	Digital	

	Country	Data years	Survey/Study name/Citation	Level of representative-ness	Rural, urban, or both	Age range as used for global analysis		Sample size		Type of blood pressure measurement device[1]	Note
						Male	Female	Male	Female		
564	Ireland	2008-2010	National Adult Nutrition Survey	National	both	18+	18+	644	671	Digital	
565	Ireland	2009-2011	The Irish Longitudinal Study on Ageing	National	both	50+	50+	2,696	3,173	Digital	
566	Israel	1981	GOH; Gerber et al., Prev Med 2005; 41: 85-91	National	both	35-69	35-69	1,760	1,907	Unknown	
567	Israel	1986	Gofin et al., Eur Heart J 1995; 16: 1988-94	Community	urban	50-84	50-84	847	1,011	Unknown	
568	Israel	1985-1986	MONICA, Tel Aviv	Community	urban	25-64	25-64	390	361	Standard	
569	Israel	1990-1991	The Jerusalem Longitudinal Cohort Study	Community	urban	69-70	69-70	248	205	Standard	
570	Israel	1996	Bobak et al., Int J Epidemiol 1999; 28: 437-44	Community	urban	45-64		160		Unknown	
571	Israel	1997-1998	The Jerusalem Longitudinal Cohort Study	Community	urban	76-77	76-77	437	436	Standard	
572	Israel	1999-2005	The Israel Glucose Intolerance, Obesity and Hypertention Study	National	urban	58+	58+	513	506	Standard	
573	Israel	2002-2007	Hadera District Study	Subnational	urban	25-79	25-79	388	377	Standard	
574	Israel	2005-2006	The Jerusalem Longitudinal Cohort Study	Community	urban	83-85	83-85	502	589	Digital	
575	Israel	2005-2006	Mabat Zahav National Health and Nutrition Survey ages 65 and over 2005-6	National	both	65+	65+	793	890	Digital	
576	Italy	1981	[No Authors], Am J Epidemiol 1981; 113: 338-46	Subnational	both	20-59	20-59	3,189	3,510	Unknown	
577	Italy	1984	Laurenzi et al., J Hypertens Suppl 1990; 8: S7-12	Community	urban	25+	25+	1,473	1,721	Unknown	
578	Italy	1982-1987	MONICA, Latina	Community	rural	24-66	24-66	852	871	Standard	
579	Italy	1985	Finland, Italy, Netherlands, Elderly (Fine-Italy)	Community	rural	65-84		680		Standard	
580	Italy	1985	INTERSALT, Naples	Community	urban	20-59	20-59	100	100	Random-zero	
581	Italy	1986	Vincenzi et al., G Ital Cardiol 1992; 22: 427-40	Community	urban	35-64	35-64	1,289	1,565	Unknown	
582	Italy	1986	INTERSALT, Bassiano	Community	urban	20-59	20-59	99	100	Random-zero	
583	Italy	1986	INTERSALT, Gubbio	Community	urban	20-59	20-59	99	100	Random-zero	
584	Italy	1986	INTERSALT, Mirano	Community	urban	20-59	20-59	100	100	Random-zero	
585	Italy	1986	MONICA, Friuli	Subnational	urban	25-64	25-64	925	919	Standard	
586	Italy	1986-1987	MONICA, Brianza	Subnational	urban	25-64	25-64	809	830	Standard	
587	Italy	1989	European Fat Study; Seidell et al., Atherosclerosis 1991; 86: 251-60	Subnational	urban	38-38		100		Unknown	
588	Italy	1989	MONICA, Friuli	Subnational	urban	25-64	25-64	906	906	Standard	
589	Italy	1989	Ventimiglia Heart Study	Community	rural	20+	20+	488	593	Unknown	
590	Italy	1990	Bruneck Study	Community	rural	40-79	40-79	469	450	Standard	
591	Italy	1983-1996	Malattie cardiovascolari ATerosclerotiche Istituto Superiore di Sanità	Community	rural	18-77	18-77	3,968	4,519	Standard	
592	Italy	1989-1990	MONICA, Brianza	Subnational	urban	25-64	25-64	764	762	Standard	
593	Italy	1991	Pasini et al., G Ital Cardiol 1999; 29: 891-7	Community	urban	40-59	40-59	747	750	Unknown	
594	Italy	1991	FINEStudy; Bijnen et al., Am J Epidemiol 1996; 143: 553-61; Site 1	Community	rural	69-90		188		Unknown	
595	Italy	1991	FINEStudy; Bijnen et al., Am J Epidemiol 1996; 143: 553-61; Site 2	Community	rural	69-90		230		Unknown	
596	Italy	1992	PAMELA; Mancia et al., Lancet 1997; 349: 454-7	Community	urban	25-74	25-74	1,032	1,013	Unknown	
597	Italy	1992-1993	Italian Longitudinal Study on Aging	National	both	65-84	65-84	1,828	1,669	Standard	
598	Italy	1994	MONICA, Friuli	Subnational	urban	25-64	25-64	883	888	Standard	
599	Italy	1993-1994	MONICA, Brianza	Subnational	urban	25-64	25-64	796	848	Standard	
600	Italy	1995	Vobarno Study; Muiesan et al., Blood Press 2006; 15: 14-9	Community	both	35-64	35-64	265	319	Unknown	
601	Italy	1995	Bruneck Study	Community	rural	45-84	45-84	414	412	Standard	
602	Italy	1996	CASTEL; Casiglia et al., J Hum Hypertens 2002; 16: 611-20	Community	urban	65+	65+	1,281	2,001	Unknown	
603	Italy	1993-1998	EPIC Florence	Community	urban	24-72	30-72	3,326	9,485	Standard	
604	Italy	1995-1996	Italian Longitudinal Study on Aging	National	both	69-90	69-90	1,091	972	Standard	
605	Italy	1995-1999	PROgetto Veneto Anziani	Subnational	both	65+	65+	1,243	1,851	Standard	
606	Italy	1998	Olivieri et al., J Clin Endocrinol Metab 2004; 89: 4221-6	Community	both	35-74	35-74	572	641	Unknown	
607	Italy	1998-1999	progetto VIP	Community	both	25-74	25-74	598	599	Standard	
608	Italy	2000	Muntoni et al., Eur J Intern Med 2009; 20: 373-7	Community	urban	20+	20+	2,250	2,487	Unknown	
609	Italy	2000	Bruneck Study	Community	rural	50-89	50-89	331	361	Standard	
610	Italy	1998-2002	Osservatorio Epidemiologico Cardiovascolare	National	both	35-74	35-74	4,878	4,772	Standard	
611	Italy	2000-2001	Italian Longitudinal Study on Aging	National	both	73-93	73-93	683	671	Standard	
612	Italy	2002	Bo et al., Diab Metab Research Rev 2005; 21: 515-24	Community	both	45-64	45-64	780	878	Unknown	
613	Italy	2002	Vobarno Study; Muiesan et al., Hypertens 2010; 28: 1935-43	Community	both	35-74	35-74	169	216	Unknown	
614	Italy	2001-2003	The Study of Asti	Community	both	45-64	45-64	780	878	Standard	
615	Italy	2000-2003	PROgetto Veneto Anziani	Subnational	both	67+	67+	804	1,364	Standard	
616	Italy	2003	The European Male Ageing Study (EMAS)	Community	both	40+		433		Digital	
617	Italy	2004	Menotti et al., J Hypertens 2009; 27: 266-74	Community	urban	20-79	20-79	1,761	2,055	Unknown	
618	Italy	2002-2005	PROgetto Veneto Anziani	Subnational	both	70+	70+	626	1,147	Standard	
619	Italy	2005	Bruneck Study	Community	rural	55-93	55-93	264	307	Standard	
620	Italy	2004-2005	Italian Project on the Epidemiolofy of Alzheimer's disease	National	both	65-84	65-84	1,578	1,449	Standard	

	Country	Data years	Survey/Study name/Citation	Level of representative-ness	Rural, urban, or both	Age range as used for global analysis		Sample size		Type of blood pressure measurement device[1]	Note
						Male	Female	Male	Female		
621	Italy	2004-2005	Vobarno study	Community	both	55-74	55-74	99	113	Standard	
622	Italy	2008	The European Male Ageing Study (EMAS)	Community	both	40+		352		Digital	
623	Italy	2005-2010	Moli-sani Study	Subnational	both	35+	35+	11,699	12,617	Digital	
624	Italy	2008-2009	progetto VIP	Community	both	25-74	25-74	600	598	Standard	
625	Italy	2010	Bruneck Study	Community	rural	60-98	60-98	224	256	Standard	
626	Italy	2008-2012	Osservatorio Epidemiologico Cardiovascolare/Health Examination Survey	National	both	35-80	35-80	4,370	4,337	Standard	
627	Italy	2010-2012	CArdiovascular risk MEtabolic syndrome LIver and Autoimmunity diseases (CA.ME.LI.A)	Community	both	18-75	18-75	477	515	Standard	
628	Italy	2011-2012	Vobarno study	Community	both	49-62	49-62	104	143	Standard	
629	Jamaica	2000-2001	Jamaica Health and Lifestyle Survey	National	both	18-74	18-74	611	1,240	Standard	
630	Jamaica	2006-2007	Jamaica Youth Risk and Resiliency Behaviour Survey 2006	National	both	18-19	18-19	149	185	Standard	
631	Jamaica	2007-2008	Jamaica Health and Lifestyle Survey	National	both	18-74	18-74	826	1,845	Standard	
632	Japan	1973	National Nutrition Survey	National	both	18+	18+	5,791	7,306	Standard	4
633	Japan	1975	National Nutrition Survey	National	both	18+	18+	4,619	6,640	Standard	
634	Japan	1976	National Nutrition Survey	National	both	18+	18+	5,555	7,824	Standard	
635	Japan	1977	National Nutrition Survey	National	both	18+	18+	4,441	6,261	Standard	
636	Japan	1978	National Nutrition Survey	National	both	18+	18+	4,767	6,595	Standard	
637	Japan	1979	National Nutrition Survey	National	both	18+	18+	5,056	6,927	Standard	
638	Japan	1980	National Nutrition Survey	National	both	18+	18+	5,790	7,317	Standard	
639	Japan	1980-1983	Aito Town Study	Community	rural	20-77	20-77	744	973	Standard	
640	Japan	1981	National Nutrition Survey	National	both	18+	18+	4,123	6,035	Standard	
641	Japan	1982	National Nutrition Survey	National	both	18+	18+	4,740	6,705	Standard	
642	Japan	1983	National Nutrition Survey	National	both	18+	18+	4,567	6,540	Standard	
643	Japan	1984	National Nutrition Survey	National	both	18+	18+	4,497	6,265	Standard	
644	Japan	1985	INTERSALT, Osaka	Community	urban	20-59	20-59	100	97	Random-zero	
645	Japan	1985	INTERSALT, Tochigi	Community	urban	20-59	20-59	95	99	Random-zero	
646	Japan	1985	INTERSALT, Toyama	Community	urban	20-59	20-59	100	100	Random-zero	
647	Japan	1985	National Nutrition Survey	National	both	18+	18+	4,864	6,608	Standard	
648	Japan	1985-1986	Akabane Study	Community	urban	40-69	40-69	813	1,023	Standard	
649	Japan	1986	National Nutrition Survey	National	both	18+	18+	4,952	6,575	Standard	
650	Japan	1987	Konan Town Study	Community	rural	20-79	20-79	70	89	Standard	
651	Japan	1987	National Nutrition Survey	National	both	18+	18+	4,083	6,092	Standard	
652	Japan	1988	Konan Town Study	Community	rural	20-79	20-79	76	85	Standard	
653	Japan	1988	National Nutrition Survey	National	both	18+	18+	4,150	5,833	Standard	
654	Japan	1989	Aito Town Study	Community	rural	25-74	25-74	530	526	Standard	
655	Japan	1989	Konan Town Study	Community	rural	20-79	20-79	59	63	Standard	
656	Japan	1988-1990	Miyama Cohort Study	Community	rural	40-80	40-80	477	601	Standard	
657	Japan	1989	National Nutrition Survey	National	both	18+	18+	3,700	5,217	Standard	
658	Japan	1990	Konan Town Study	Community	rural	20-79	20-79	30	58	Standard	
659	Japan	1990	National Nutrition Survey	National	both	18+	18+	4,091	5,632	Standard	
660	Japan	1991	Konan Town Study	Community	rural	20-79	20-79	93	117	Standard	
661	Japan	1991	Shigaraki Town Study	Community	rural	30-89	30-89	234	330	Standard	
662	Japan	1991	National Nutrition Survey	National	both	18+	18+	3,871	5,332	Standard	
663	Japan	1992	Konan Town Study	Community	rural	20-79	20-79	55	54	Standard	
664	Japan	1992	Shigaraki Town Study	Community	rural	30-89	30-89	288	387	Standard	
665	Japan	1992	National Nutrition Survey	National	both	18+	18+	3,589	5,016	Standard	
666	Japan	1993	Konan Town Study	Community	rural	20-79	20-79	54	65	Standard	
667	Japan	1993	Shigaraki Town Study	Community	rural	30-89	30-89	301	454	Standard	
668	Japan	1993	National Nutrition Survey	National	both	18+	18+	3,233	4,664	Standard	
669	Japan	1994	Konan Town Study	Community	rural	20-79	20-79	43	59	Standard	
670	Japan	1994	Shigaraki Town Study	Community	rural	30-89	30-89	252	336	Standard	
671	Japan	1994	National Nutrition Survey	National	both	18+	18+	3,138	4,537	Standard	
672	Japan	1995	Konan Town Study	Community	rural	20-79	20-79	45	61	Standard	
673	Japan	1995	Shigaraki Town Study	Community	rural	30-89	30-89	300	470	Standard	
674	Japan	1995	National Nutrition Survey	National	both	18+	18+	3,018	4,444	Standard	
675	Japan	1996	Shigaraki Town Study	Community	rural	30-79	30-89	86	152	Standard	
676	Japan	1996	National Nutrition Survey	National	both	18+	18+	2,954	4,281	Standard	
677	Japan	1997	Shigaraki Town Study	Community	rural	30-79	30-89	61	100	Standard	

	Country	Data years	Survey/Study name/Citation	Level of representative-ness	Rural, urban, or both	Age range as used for global analysis		Sample size		Type of blood pressure measurement device[1]	Note
						Male	Female	Male	Female		
678	Japan	1996-1997	INTERMAP, AitoTown	Community	both	40-59	40-59	130	129	Random-zero	
679	Japan	1997	National Nutrition Survey	National	both	18+	18+	2,892	4,220	Standard	
680	Japan	1997-1998	INTERMAP, Sapporo	Community	urban	40-59	40-59	149	148	Random-zero	
681	Japan	1997-1998	INTERMAP, Toyama	Community	urban	40-59	40-59	149	150	Random-zero	
682	Japan	1997-1998	INTERMAP, Wakayama	Community	urban	40-59	40-59	146	144	Random-zero	
683	Japan	1998	Niigata Study	Community	urban	70-70	70-70	305	294	Standard	
684	Japan	1998	National Nutrition Survey	National	both	18+	18+	3,115	4,472	Standard	
685	Japan	1999	Niigata Study	Community	urban	71-71	71-71	245	217	Standard	
686	Japan	1999	National Nutrition Survey	National	both	18+	18+	2,415	3,672	Standard	
687	Japan	2000	Niigata Study	Community	urban	72-72	72-72	234	201	Standard	
688	Japan	2000	National Nutrition Survey	National	both	18+	18+	2,570	3,645	Standard	
689	Japan	2001	The Japan Association of Health Service Database	Subnational	both	20+	20+	1,318,133	1,061,237	Digital	
690	Japan	2001	Niigata Study	Community	urban	73-73	73-73	236	199	Standard	
691	Japan	2001	National Nutrition Survey	National	both	18+	18+	2,327	3,616	Standard	
692	Japan	2002	Niigata Study	Community	urban	74-74	74-74	228	203	Standard	
693	Japan	2002	National Nutrition Survey	National	both	18+	18+	2,280	3,358	Standard	
694	Japan	2002-2003	The Hisayama Study	Community	rural	40+	40+	1,414	1,883	Digital	
695	Japan	2003	National Health and Nutrition Survey	National	both	18+	18+	2,259	3,389	Standard	
696	Japan	2003	Niigata Study	Community	urban	75-75	75-75	216	192	Standard	
697	Japan	2004	National Health and Nutrition Survey	National	both	18+	18+	1,666	2,521	Standard	
698	Japan	2004	Niigata Study	Community	urban	76-76	76-76	216	188	Standard	
699	Japan	2005	National Health and Nutrition Survey	National	both	18+	18+	1,666	2,427	Standard	
700	Japan	2005	Niigata Study	Community	urban	77-77	77-77	204	189	Standard	
701	Japan	2006	National Health and Nutrition Survey	National	both	18+	18+	1,858	2,715	Standard	
702	Japan	2006	Niigata Study	Community	urban	78-78	78-78	200	197	Standard	
703	Japan	2007	National Health and Nutrition Survey	National	both	18+	18+	1,754	2,562	Standard	
704	Japan	2007	Niigata Study	Community	urban	79-79	79-79	185	194	Standard	
705	Japan	2008	Study of residents in Kanazawa city	Community	urban	35+	35+	6,562	11,944	Unknown	
706	Japan	2008	National Health and Nutrition Survey	National	both	18+	18+	1,933	2,753	Standard	
707	Japan	2008	Niigata Study	Community	urban	80-80	80-80	177	180	Standard	
708	Japan	2009	National Health and Nutrition Survey	National	both	18+	18+	1,820	2,664	Standard	
709	Japan	2010	National Health and Nutrition Survey	National	both	18+	18+	1,711	2,416	Standard	
710	Japan	2011	National Health and Nutrition Survey	National	both	18+	18+	1,597	2,266	Standard	
711	Japan	2011	The Tokyo Health Service Association Database	Community	urban	20+	20+	82,375	53,738	Digital	
712	Japan	2012	National Health and Nutrition Survey	National	both	20+	20+	6,163	8,875	Standard	
713	Japan	2013	National Health and Nutrition Survey	National	both	20+	20+	1,487	2,061	Standard	
714	Japan	2014	National Health and Nutrition Survey	National	both	20+	20+	1,555	2,141	Standard	
715	Jordan	1995	Jaddou et al., J Hum Hypertens 2000; 14: 497-501	Subnational	urban	25+	25+	841	1,435	Unknown	
716	Jordan	2004	Behavioural Risk Factor Surveillence Survey	National	both	18+	18+	237	474	Standard	
717	Jordan	2007	Behavioural Risk Factor Surveillence Survey	National	both	18+	18+	332	433	Standard	
718	Jordan	2009	Metablic abnomalities and vitamin D study	National	both	18+	18+	1,142	3,347	Standard	
719	Kenya	1980	Poulter et al, Trans R Soc Trop Med Hyg 1985; 79: 389-92; Site 1	Community	rural	15-54	15-54	182	483	Unknown	2
720	Kenya	1980	Poulter et al, Trans R Soc Trop Med Hyg 1985; 79: 389-92; Site 2	Community	rural	15-54	15-54	52	178	Unknown	2
721	Kenya	1980	Poulter et al, Trans R Soc Trop Med Hyg 1985; 79: 389-92; Site 3	Community	urban	15-54	15-54	204	87	Unknown	2
722	Kenya	1980-1981	Poulter et al., J Epidemiol Community Health 1984; 38: 181-5	Community	rural		20+		597	Random-zero	
723	Kenya	1980-1981	Poulter et al., Hypertension 1984; 6: 810-3	Community	rural	17+		1,737		Random-zero	2
724	Kenya	1985	INTERSALT	Community	rural	20-59	20-59	90	86	Random-zero	
725	Kiribati	1981	Epidemiological survey of Kiribati	Subnational	rural	20+	20+	474	534	Random-zero	
726	Kiribati	1981	Epidemiological survey of Kiribati	Subnational	urban	20+	20+	939	906	Random-zero	
727	Kiribati	2004	STEPS	National	both	18-64	18-64	734	900	Digital	
728	Kuwait	2006	STEPS	National	both	20-65	20-65	918	1,297	Standard	
729	Kuwait	2008-2010	World Health Survey	National	both	18+	18+	1,873	1,691	Digital	
730	Kuwait	2014	STEPS	National	both	18-69	18-69	1,380	2,216	Standard	
731	Kyrgyzstan	2012	DHS	National	both	18-49	18-49	2,122	6,559	Digital	
732	Kyrgyzstan	2013	STEPS	National	both	25-64	25-64	943	1,601	Digital	
733	Lao PDR	2008	STEPS	Community	urban	25-64	25-64	1,657	2,446	Digital	
734	Lao PDR	2013	STEPS	National	both	18-64	18-64	989	1,470	Digital	

	Country	Data years	Survey/Study name/Citation	Level of representative-ness	Rural, urban, or both	Age range as used for global analysis		Sample size		Type of blood pressure measurement device[1]	Note
						Male	Female	Male	Female		
735	Latvia	2008-2009	Cardiovascular risk factor study	National	both	25-74	25-74	1,358	2,409	Digital	
736	Lebanon	2008-2009	STEPS	National	both	18+	18+	1,319	1,480	Standard	
737	Lesotho	2009-2010	DHS	National	both	18-59	18-49	2,665	3,160	Digital	
738	Lesotho	2012	STEPS	National	both	25-64	25-64	743	1,457	Digital	
739	Lesotho	2014	DHS	National	both	18-59	18-49	2,386	2,694	Digital	
740	Liberia	2011	STEPS	National	both	25-64	25-64	1,017	1,270	Digital	
741	Libya	1999	Kadiki and Roaeid, Diabetes Metab 2001; 27: 647-54	Community	both	25-84	25-74	229	378	Unknown	
742	Libya	2009	STEPS	National	both	25-64	25-64	1,684	1,569	Digital	
743	Lithuania	1977-1980	Multifactorial Prevention of Ishaemic Heart Disease, Kaunas	Community	urban	40-59		5,721		Standard	
744	Lithuania	1983	CINDI	Community	both	25-64	25-64	1,823	1,955	Unknown	
745	Lithuania	1983-1985	MONICA, Kaunas	Community	urban	35-64	35-64	728	734	Standard	
746	Lithuania	1987	Countrywide Integrated Noncommunicable Diseases Intervention Programme survey	Subnational	rural	25-64	25-64	1,227	1,445	Standard	
747	Lithuania	1986-1987	MONICA, Kaunas	Community	urban	35-64	35-64	894	868	Standard	
748	Lithuania	1992-1993	MONICA, Kaunas	Community	urban	35-64	35-64	611	628	Standard	
749	Lithuania	1992-1993	Countrywide Integrated Noncommunicable Diseases Intervention Programme survey	Subnational	rural	25-64	25-64	677	864	Standard	
750	Lithuania	1998-1999	Countrywide Integrated Noncommunicable Diseases Intervention Programme survey	Subnational	rural	25-64	25-64	816	1,019	Standard	
751	Lithuania	2001-2002	MONICA4	Community	urban	35-64	35-64	626	775	Standard	
752	Lithuania	2006-2007	Countrywide Integrated Noncommunicable Diseases Intervention Programme survey	Subnational	rural	25-64	25-64	731	982	Standard	
753	Lithuania	2006-2008	Health, Alcohol and Psychosocial factors In Eastern Europe	Community	urban	45-75	45-75	3,221	3,863	Digital	
754	Luxembourg	2007-2009	Observation des Risques et de la Santé Cardio-Vasculaire au Luxembourg (ORISCAV-LUX)	National	both	18-69	18-69	657	694	Digital	
755	Madagascar	2005	STEPS	Community	urban	25-64	25-64	2,745	2,682	Digital	
756	Malawi	2009	STEPS	National	both	25-64	25-64	1,183	2,527	Digital	
757	Malaysia	1996	National Health and Morbidity Survey	National	both	30+	30+	9,990	11,379	Digital	
758	Malaysia	2004	Rampal et al., Public Health 2008; 122: 11-8	National	both	15+	15+	7,301	9,899	Standard	2
759	Malaysia	2005	STEPS	National	both	25-64	25-64	1,043	1,526	Standard	
760	Malaysia	2006	The Third National Health and Morbidity Survey (NHMS III)	National	both	18+	18+	15,205	18,770	Digital	
761	Malaysia	2008	Metabolic Syndrome Study in Malaysia	National	both	18+	18+	1,523	2,817	Digital	
762	Malaysia	2011	National Health and Morbidity Survey (NHMS)	National	both	18+	18+	8,228	9,443	Digital	
763	Malaysia	2015	National Health and Morbidity Survey (NHMS)	National	both	20+	18+	8,667	10,100	Digital	
764	Maldives	2011	STEPS	National	both	18-64	18-64	589	1,006	Digital	
765	Mali	2007	STEPS	Community	both	18-64	18-64	954	1,356	Digital	
766	Malta	1984	MONICA, Malta	Community	urban	25-64	25-64	963	939	Standard	
767	Malta	1985	CINDI	Community	both	25-64	25-64	947	930	Standard	
768	Malta	1986	INTERSALT	Community	rural	20-59	20-59	100	100	Random-zero	
769	Marshall Islands	2002	STEPS	National	both	18-64	18-64	700	1,058	Digital	
770	Mauritius	1987	Mauritius Noncommunicable Disease Survey	National	both	25-74	25-74	2,351	2,659	Standard	
771	Mauritius	1992	Mauritius Noncommunicable Disease Survey	National	both	25-74	25-74	2,984	3,475	Standard	
772	Mauritius	1998	Mauritius Noncommunicable Disease Survey	National	both	25-74	25-74	2,560	3,243	Standard	
773	Mauritius	2009	Mauritius Noncommunicable Disease Survey	National	both	20-79	20-79	2,901	3,430	Digital	
774	Mexico	1992	Yamamoto-Kimura et al., Arch Med Res 1998; 29: 341-9	Community	urban	20-90	20-90	72	104	Unknown	
775	Mexico	1992-1993	Encuesta Nacional de Enfermedades Cronicas	National	urban	20-69	20-69	6,115	8,351	Standard	
776	Mexico	1995	Guerrero-Romero et al., Salud Publica Mex 1998; 40: 339-46	Community	rural	20+	20+	1,350	4,452	Unknown	
777	Mexico	2000	Encuesta Nacional de Salud	National	both	20+	20+	13,689	28,706	Standard	
778	Mexico	1999-2000	The Survey on Health, Well-Being, and Aging in Latin America and the Caribbean (SABE)	Community	urban	60+	60+	367	563	Unknown	
779	Mexico	2002	Encuesta Nacional Sobre Niveles de vida de los Hogares	National	both	18+	18+	7,344	9,131	Digital	
780	Mexico	2004-2005	CArdiovascular Risk factors Multiple Evaluation in Latin America (CARMELA)	Community	urban	25-64	25-64	833	889	Standard	
781	Mexico	2005	Encuesta Nacional Sobre Niveles de vida de los Hogares	National	both	18+	18+	7,542	9,106	Digital	
782	Mexico	2006	Encuesta Nacional de Salud Y Nutricion	National	both	20+	20+	13,358	20,008	Standard	
783	Mexico	2006	PREVENIMSS National Coverage Surveys	Subnational	both	20+	20+	8,722	11,333	Standard	
784	Mexico	2009-2010	SAGE	National	both	50+	50+	834	1,298	Digital	
785	Mexico	2011-2012	Encuesta Nacional de Salud Y Nutricion	National	both	20+	20+	4,563	6,629	Standard	
786	Mexico	2012	The Mexican Health and Aging Study	National	both	50+	50+	803	1,126	Digital	
787	Micronesia (Federated Stat	2002	STEPS	Subnational	both	25-64	25-64	637	948	Digital	
788	Micronesia (Federated Stat	2006	STEPS	Subnational	both	25-64	25-64	635	1,164	Digital	
789	Micronesia (Federated Stat	2008	STEPS	Subnational	both	25-64	25-64	897	1,292	Digital	
790	Micronesia (Federated Stat	2009	STEPS	Subnational	both	20-64	20-64	213	429	Digital	
791	Micronesia (Federated Stat	2009	STEPS	Subnational	both	18-64	18-64	421	545	Digital	

	Country	Data years	Survey/Study name/Citation	Level of representative-ness	Rural, urban, or both	Age range as used for global analysis		Sample size		Type of blood pressure measurement device[1]	Note
						Male	Female	Male	Female		
792	Moldova	2013	STEPS	National	both	18-69	18-69	1,729	2,809	Digital	
793	Mongolia	1999	National Nutrition Survey; Suvd et al., Diabet Med 2002; 19: 502-8	National	both	35+	35+	1,009	1,438	Standard	
794	Mongolia	2005	STEPS	National	both	18-64	18-64	1,508	1,586	Digital	
795	Mongolia	2009	STEPS	National	both	18-64	18-64	2,094	2,964	Digital	
796	Mongolia	2013	STEPS	National	both	18-64	18-64	2,330	2,808	Digital	
797	Mozambique	2005	STEPS	National	both	25-64	25-64	1,280	1,687	Digital	
798	Myanmar	2003-2004	STEPS	Subnational	both	25-74	25-74	1,989	2,448	Digital	
799	Namibia	2005	STEPS	National	both	24-64	24-64	1,403	1,795	Digital	
800	Namibia	2009	Okambilimbili Survey	Community	urban	18+	18+	761	969	Digital	
801	Namibia	2013	DHS	National	both	30-64	30-64	1,539	2,042	Digital	
802	Nauru	1975-1976	Trends in the prevalence and incidence of non-insulin-dependent diabetes mellitus and impaired glucose tolerance	Subnational	both	18-79	18-79	235	241	Unknown	
803	Nauru	1982	Trends in the prevalence and incidence of non-insulin-dependent diabetes mellitus and impaired glucose tolerance	National	both	20+	20+	706	780	Unknown	
804	Nauru	1987	Trends in the prevalence and incidence of non-insulin-dependent diabetes mellitus and impaired glucose tolerance	National	both	20+	20+	553	665	Standard	
805	Nauru	1994	Trends in the prevalence and incidence of non-insulin-dependent diabetes mellitus and impaired glucose tolerance	National	both	25+	25+	647	726	Standard	
806	Nauru	2004	STEPS	National	both	18-64	18-64	1,014	1,077	Digital	
807	Nauru	2006	STEPS	National	both	18-65	18-65	245	231	Digital	
808	Nepal	2006-2011	Early detection and management of Kidney disease, Hypertension, Diabetes and Cardiovascular disease (KHDC Nepal), Tarahara	Community	rural	18+	18+	1,176	2,351	Standard	
809	Nepal	2006-2011	Early detection and management of Kidney disease, Hypertension, Diabetes and Cardiovascular disease (KHDC Nepal), Damak	Community	urban	18+	18+	1,095	1,577	Standard	
810	Nepal	2006-2011	Early detection and management of Kidney disease, Hypertension, Diabetes and Cardiovascular disease (KHDC Nepal), Dharan	Community	urban	18+	18+	4,130	6,127	Standard	
811	Nepal	2013	STEPS	National	both	18-69	18-69	1,276	2,703	Digital	
812	Netherlands	1983	Van Loo et al., Ned Tijdschr Geneeskd 1987; 131: 624-7	Community	urban	20-49	20-49	2,876	3,093	Unknown	
813	Netherlands	1985	INTERSALT	Community	urban	20-59	20-59	100	99	Random-zero	
814	Netherlands	1985	Zutphen Elderly Study	Community	urban	65-85		886		Random-zero	
815	Netherlands	1986	European Fat Distribution Study; Seidell et al., Clin Epidemiol 1990; 43: 21-34	Subnational	urban		38-38		140	Unknown	
816	Netherlands	1986	van Poppel et al., Int J Vitam Nutr Res 1989; 59: 381-7	Community	urban	35-35		60		Unknown	
817	Netherlands	1990	Zutphen Elderly Study	Community	urban	69-90		554		Random-zero	
818	Netherlands	1989-1993	the Rotterdam Study, first subcohort	Community	urban	55+	55+	2,797	4,212	Random-zero	
819	Netherlands	1993-1995	the Rotterdam Study, first subcohort	Community	urban	56+	56+	2,305	3,278	Random-zero	
820	Netherlands	1993-1997	EPIC Bilthoven	Community	urban	20-59	20-59	9,662	11,653	Unknown	
821	Netherlands	1993-1997	EPIC Utrecht	Community	both		49-70		16,991	Digital	
822	Netherlands	1997	AGHLS; Twisk et al., Int J Sports Med 2002; 23 Suppl 1: S8-14	Community	urban	32-32	32-32	132	145	Unknown	
823	Netherlands	1997-1999	the Rotterdam Study, first subcohort	Community	urban	61+	61+	1,741	2,428	Random-zero	
824	Netherlands	2000	MPCDRF; Schelleman et al., J Hum Hypertens 2004; 18: 317-24	Community	urban	30-59	30-59	5,004	5,816	Unknown	
825	Netherlands	1998-2001	Regenboog Project	National	both	18-89	18-89	2,580	2,483	Digital	
826	Netherlands	2000-2001	the Rotterdam Study, second subcohort	Community	urban	55+	55+	1,212	1,472	Random-zero	
827	Netherlands	2002	Utrecht Health Project; Scheltens et al., J Hum Hypertens 2007; 21: 99-106	Community	urban	20-59	20-59	1,924	2,418	Unknown	
828	Netherlands	2001-2003	Surinamese in the Netherlands: Study on Ethnicity and Health	Community	urban	35-60	35-60	251	257	Digital	
829	Netherlands	2002-2004	the Rotterdam Study, first subcohort	Community	urban	65+	65+	1,288	1,852	Random-zero	
830	Netherlands	2004-2005	the Rotterdam Study, second subcohort	Community	urban	58+	58+	980	1,267	Random-zero	
831	Netherlands	2006-2008	the Rotterdam Study, third subcohort	Community	urban	45+	45+	1,574	2,068	Digital	
832	Netherlands	2009-2010	Measuring the Netherlands (NL de Maat)	Subnational	both	30-70	30-70	1,779	2,019	Digital	
833	Netherlands	2009-2011	the Rotterdam Study, first subcohort	Community	urban	72+	72+	668	960	Digital	
834	Netherlands	2011-2012	the Rotterdam Study, second subcohort	Community	urban	65+	65+	720	913	Digital	
835	Netherlands	2011-2015	Healthy Life in an Urban Setting	Community	urban	18-71	18-71	2,085	2,470	Digital	
836	Netherlands	2012-2014	the Rotterdam Study, third subcohort	Community	urban	51+	51+	1,254	1,635	Digital	
837	New Zealand	1978	Simpson et al., N Z Med J 1982; 95: 873-6	Community	urban	16+	16+	579	612	Unknown	2
838	New Zealand	1981	Simpson et al., N Z Med J 1982; 95: 873-6	Community	urban	16+	16+	546	590	Unknown	2
839	New Zealand	1982	MONICA, Auckland	Community	urban	35-64	35-64	1,019	568	Random-zero	
840	New Zealand	1989	LINZ; Nye et al., N Z Med J 1992; 105: 1-3	Subnational	both	15-59	15-59	1,110	1,301	Unknown	2
841	New Zealand	1993-1994	MONICA, Auckland	Community	urban	35-64	35-64	745	726	Random-zero	
842	New Zealand	2008-2009	The Adult Nutrition Survey	National	both	15+	15+	1,949	2,396	Digital	2
843	New Zealand	2012-2013	2012/13 New Zealand Health Survey	National	both	15+	15+	4,952	6,692	Digital	2
844	Nicaragua	2003-2004	CAMDI	Community	urban	20+	20+	774	916	Digital	
845	Niger	1983	Bretagne et al., Bull Soc Pathol Exot Filiales 1985; 78: 79-88; Site 1	Community	rural	15-34		30		Unknown	2

	Country	Data years	Survey/Study name/Citation	Level of representative-ness	Rural, urban, or both	Age range as used for global analysis		Sample size		Type of blood pressure measurement device[1]	Note
						Male	Female	Male	Female		
846	Niger	1983	Bretagne et al., Bull Soc Pathol Exot Filiales 1985; 78: 79-88; Site 2	Community	rural	15-34		32		Unknown	2
847	Niger	2007	STEPS	National	both	18-64	18-64	1,372	1,140	Digital	
848	Nigeria	1990	Non-communicable Diseases National Survey	National	both	15-99	15-99	8,359	7,655	Unknown	2
849	Nigeria	1995	Okesina et al., East Afr Med J 1999; 76: 212-6	Community	rural	21-50	21-50	106	205	Unknown	
850	Nigeria	2007	Ibadan Study of Ageing	Subnational	both	60+	60+	670	928	Digital	
851	Nigeria	2008	Ibadan Study of Ageing	Subnational	both	61+	61+	475	659	Digital	
852	Nigeria	2009	Ibadan Study of Ageing	Subnational	both	62+	62+	445	615	Digital	
853	Nigeria	2009	Community Health Plan - Kwara Central Survey	Community	rural	18+	18+	1,251	1,437	Digital	
854	Nigeria	2011	Community Health Plan - Kwara Central Survey	Community	rural	18+	18+	399	504	Digital	
855	Nigeria	2013	Community Health Plan - Kwara Central Survey	Community	rural	18+	18+	359	428	Digital	
856	Niue	2011	STEPS	National	both	18-100	18-100	379	455	Digital	
857	North Korea	2005	STEPS	Community	urban	25-64	25-64	1,044	1,081	Digital	
858	North Korea	2007	STEPS	Community	rural	25-64	25-64	1,155	1,176	Digital	
859	North Korea	2008	STEPS	Subnational	both	25-64	25-64	2,818	2,924	Digital	
860	Norway	1980	Norwegian Counties Study; Graff-Iversen et al., Heart 2008; 94: 482-6	Community	rural	40-49	40-49	16,616	16,265	Unknown	
861	Norway	1979-1980	The Tromsø Study: Tromsø 2	Community	both	20-54	20-49	8,448	7,887	Standard	
862	Norway	1984-1986	HUNT1 Study	Subnational	rural	20+	20+	36,655	38,145	Standard	
863	Norway	1987	Oslo Risk Factor Screening Program; Jenum et al., Int J Epidemiol 2001; 30 Suppl 1: S59-65	Community	urban	40-40	40-40	9,100	9,100	Unknown	
864	Norway	1986-1987	The Tromsø Study: Tromsø 3	Community	both	20-61	20-56	10,372	9,820	Digital	
865	Norway	1994-1995	The Tromsø Study: Tromsø 4	Community	both	25+	25+	12,775	13,864	Digital	
866	Norway	1995-1997	HUNT2 study	Subnational	rural	20+	20+	30,297	33,836	Digital	
867	Norway	1995-1997	Young-HUNT1 Study	Subnational	rural	20-21	20-21	9	12	Digital	
868	Norway	1998	HUSK; Brekke et al., BMC Geriatr 2006; 6: 16	Community	urban	70-74	70-74	1,473	1,886	Unknown	
869	Norway	2000-2003	the Oslo cohort (HUBBRO), the Oppland and Hedmark cohort (OPPHED), and the Troms and Finnmark cohort (TROFINN) of COHORT NORWAY	Subnational	both	30-76	30-76	16,888	20,660	Digital	
870	Norway	2001-2002	The Tromsø Study: Tromsø 5, Tromsø Study Panel	Community	both	30-89	30-89	2,542	3,594	Digital	
871	Norway	2006-2008	HUNT3 Study	Subnational	rural	20+	20+	20,021	24,072	Digital	
872	Norway	2006-2008	Young-HUNT3 Study	Subnational	rural	20-21	20-21	14	8	Digital	
873	Norway	2007-2008	The Tromsø Study: Tromsø 6	Community	both	30-87	30-87	6,014	6,868	Digital	
874	Occupied Palestinian Territ	1996-1998	Abdul-Rahim et al., Diabetes Care 2001; 24: 275-9	Community	rural	30-64	18-64	206	443	Standard	
875	Occupied Palestinian Territ	1996-1998	Abdul-Rahim et al., Diabetes Care 2001; 24: 275-9	Community	urban	30-64	18-64	182	458	Standard	
876	Occupied Palestinian Territ	1999-2000	The First National Health and Nutrition Survey	National	both	20-59	20-59	1,600	1,704	Random-zero	
877	Occupied Palestinian Territ	2010	STEPS	National	both	20-64	20-64	1,847	3,401	Digital	
878	Oman	2000	Al-Riyami and Afifi, Saudi Med J 2003; 24: 641-6	National	both	20-59	20-59	3,075	3,125	Unknown	
879	Oman	2006	STEPS	Community	urban	20-59	20-59	540	732	Digital	
880	Oman	2008	The Oman World Health Survey	National	both	18+	18+	2,275	2,343	Digital	
881	Pakistan	1990-1994	National Health Survey Of Pakistan 1990-1994	National	both	18+	18+	3,936	4,489	Standard	
882	Pakistan	1995	Shah et la., J Hum Hypertens 2001; 15: 107-12	Community	rural	18-59	18-59	1,112	2,529	Unknown	
883	Pakistan	2004	COBRA-1	Community	urban	40+	40+	1,502	1,639	Unknown	
884	Pakistan	2005	STEPS	National	both	25-65	25-65	800	1,100	Digital	
885	Pakistan	2014	STEPS	Subnational	both	18-69	18-69	2,989	3,703	Digital	
886	Palau	2013	STEPS	National	both	25-64	25-64	1,046	1,133	Digital	
887	Panama	2010-2011	Prevalencia de factores de riesgo asociados a enfermedad cardiovascular 2010-2011	Subnational	both	18+	18+	1,073	2,474	Digital	
888	Papua New Guinea	1983	King et al., J Epidemiol Community Health 1985; 39: 215-9; Site 1	Community	rural	20-44	20-44	59	58	Unknown	
889	Papua New Guinea	1983	King et al., J Epidemiol Community Health 1985; 39: 215-9; Site 2	Community	rural	20-44	20-44	23	49	Unknown	
890	Papua New Guinea	1986	King et al., P N G Med J 1994; 37: 100-9; Site 1	Community	rural	20-54	20-54	75	76	Unknown	
891	Papua New Guinea	1986	King et al., P N G Med J 1994; 37: 100-9; Site 2	Community	rural	20-54	20-54	100	118	Unknown	
892	Papua New Guinea	1986	King et al., P N G Med J 1994; 37: 100-9; Site 3	Community	rural	20-54	20-54	66	91	Unknown	
893	Papua New Guinea	1986	King et al., P N G Med J 1994; 37: 100-9; Site 4	Community	rural	20-54	20-54	106	115	Unknown	
894	Papua New Guinea	1986	King et al., P N G Med J 1994; 37: 100-9; Site 5	Community	rural	20-54	20-54	92	121	Unknown	
895	Papua New Guinea	1986	King et al., P N G Med J 1994; 37: 100-9; Site 6	Community	rural	20-54	20-54	97	112	Unknown	
896	Papua New Guinea	1985-1986	INTERSALT	Community	rural	20-59	20-59	88	74	Random-zero	
897	Papua New Guinea	2007	STEPS	National	both	18-64	18-64	1,291	1,325	Digital	
898	Peru	2005	FRENT	Subnational	urban	25+	25+	1,052	2,147	Unknown	
899	Peru	2004-2005	CArdiovascular Risk factors Multiple Evaluation in Latin America (CARMELA)	Community	urban	25-64	25-64	769	883	Standard	
900	Peru	2004-2005	Encuesta Nacional de Indicadores Nutricionales, Bioquímicos, Socioeconómicos y Culturales Relacionados con las Enfermedades Crónicas Degenerativas (ENIN)	National	both	20+	20+	2,091	2,101	Standard	

	Country	Data years	Survey/Study name/Citation	Level of representative-ness	Rural, urban, or both	Age range as used for global analysis		Sample size		Type of blood pressure measurement device[1]	Note
						Male	Female	Male	Female		
901	Peru	2007-2008	PERU MIGRANT Study	Community	both	35+	35+	406	442	Digital	
902	Peru	2010	DHS	National	both	50+	18+	7,736	28,604	Digital	
903	Peru	2009-2012	CRONICAS Cohort Study	Subnational	both	35+	35+	1,557	1,659	Digital	
904	Peru	2011	DHS	National	both	40+	40+	12,894	14,866	Digital	
905	Peru	2012	DHS	National	both	40+	40+	14,278	15,928	Digital	
906	Peru	2013	DHS	National	both	18+	18+	2,759	15,967	Digital	
907	Peru	2014	DHS	National	both	18+	18+	11,882	13,933	Digital	
908	Peru	2015	DHS	National	both	18+	18+	13,826	17,479	Digital	
909	Philippines	1998-1999	Cebu Longitudinal Health and Nutrition Survey 1998-1999 Mother Followup	Community	both		20-59		1,913	Standard	
910	Philippines	2002	Cebu Longitudinal Health and Nutrition Survey 2002 Child Followup	Community	both	18-19	18-19	1,085	896	Standard	
911	Philippines	2002	Cebu Longitudinal Health and Nutrition Survey 2002 Mother Followup	Community	both		32-66		2,080	Standard	
912	Philippines	2003-2004	National Nutrition and Health Survey	National	both	20+	20+	2,222	2,183	Standard	
913	Philippines	2005	Cebu Longitudinal Health and Nutrition Survey 2005 Child Followup	Community	both	20-22	20-22	1,005	831	Standard	
914	Philippines	2005	Cebu Longitudinal Health and Nutrition Survey 2005 Mother Followup	Community	both		35-69		2,001	Standard	
915	Philippines	2007	Cebu Longitudinal Health and Nutrition Survey 2007 Child Followup	Community	both	23-24	23-24	992	776	Standard	
916	Philippines	2007	Cebu Longitudinal Health and Nutrition Survey 2007 Mother Followup	Community	both		38-71		1,974	Standard	
917	Philippines	2008	Philippines LIFECARE Cohort	National	both	20-49	20-49	1,329	1,743	Digital	
918	Philippines	2009	Cebu Longitudinal Health and Nutrition Survey 2009 Child Followup	Community	both	24-26	24-26	907	742	Standard	
919	Philippines	2009	Life Course Study in Cardiovascular Disease Epidemiology	Subnational	both	20-50	20-50	1,329	1,741	Digital	
920	Philippines	2013	8th National Nutrition Survey Philippine	National	both	18+	18+	69,401	67,644	Standard	
921	Poland	1980	Proniewska et al., Rocz Akad Med Im Juliana Marchlewskiego Bialymst 1982; 26: 89-95	Community	urban	40-59		2,108		Unknown	
922	Poland	1980	Proniewska et al., Rocz Akad Med Im Juliana Marchlewskiego Bialymst 1982; 26: 89-95	Community	rural	40-59		746		Unknown	
923	Poland	1983	Kocemba et al., J Hypertens Suppl 1991; 9: S286-7	Community	urban	70-93	70-93	153	359	Unknown	
924	Poland	1983-1984	MONICA, Tarnobrzeg Voivodship	Community	rural	35-64	35-64	1,237	1,441	Standard	
925	Poland	1983-1985	MONICA, Warsaw	Community	urban	35-64	35-64	1,297	1,327	Standard	
926	Poland	1986	Kocemba et al., Folia Med Cracov 1988; 29: 141-52	Community	urban	70-93	70-93	359	153	Unknown	
927	Poland	1986	INTERSALT, Krakow	Community	urban	20-59	20-59	100	100	Random-zero	
928	Poland	1986	INTERSALT, Warsaw	Community	urban	20-59	20-59	100	100	Random-zero	
929	Poland	1987-1988	MONICA, Tarnobrzeg Voivodship	Community	rural	35-64	35-64	616	672	Standard	
930	Poland	1988-1989	MONICA, Warsaw	Community	urban	35-64	35-64	705	713	Standard	
931	Poland	1989-1990	CINDI Łodz	Community	urban	25-64	25-64	822	948	Digital	
932	Poland	1992-1993	MONICA, Tarnobrzeg Voivodship	Community	rural	35-64	35-64	621	696	Standard	
933	Poland	1993	MONICA, Warsaw	Community	urban	35-64	35-64	751	763	Standard	
934	Poland	1995-1996	CINDI Łodz	Community	urban	18-64	18-64	954	1,417	Digital	
935	Poland	1997	Wilmanska et al., Przegl Lek 2002; 59: 252-5	Community	rural	75+	75+	83	147	Unknown	
936	Poland	1997	Wilmanska et al., Przegl Lek 2002; 59: 252-5	Community	urban	75+	75+	91	142	Unknown	
937	Poland	2000	CINDI Torun	Community	urban	18-83	18-79	930	1,020	Digital	
938	Poland	2001-2002	CINDI Łodz	Community	urban	18-64	18-64	997	838	Digital	
939	Poland	2002	CINDI Łodz senior	Community	urban	65+	65+	291	538	Digital	
940	Poland	2003	The European Male Ageing Study (EMAS)	Community	both	40+		407		Digital	
941	Poland	2002-2005	Health, Alcohol and Psychosocial factors In Eastern Europe	Community	urban	45-69	45-69	4,454	4,715	Digital	
942	Poland	2003-2005	National Multicenter Health Survey in Poland. Project WOBASZ	National	both	20-74	20-74	6,311	6,967	Digital	
943	Poland	2006	CINDI Torun	Community	urban	18-65	18-65	749	1,114	Digital	
944	Poland	2008	The European Male Ageing Study (EMAS)	Community	both	40+		311		Digital	
945	Poland	2003-2013	Mogielica Human Ecology Study	Community	rural	21+	21+	349	893	Digital	
946	Poland	2007-2011	Medical, psychological and socioeconomic aspects of aging in Poland (PolSenior)	National	both	55+	55+	2,877	2,756	Digital	
947	Poland	2011	NATPOL	National	both	20-79	20-79	1,128	1,215	Digital	
948	Portugal	1985	CINDI	Community	both	25-64	25-64	601	711	Unknown	
949	Portugal	1986	INTERSALT	Community	both	20-59	20-59	99	99	Random-zero	
950	Portugal	1999-2003	EPIPorto Study	Community	urban	18+	18+	888	1,424	Standard	
951	Portugal	2003-2004	The PAP Study	National	both	18-90	18-90	2,286	2,736	Digital	
952	Portugal	2010-2012	Exercise for Elderly	Community	urban	60-84	60-84	47	104	Digital	
953	Portugal	2011-2013	EPITeen - Epidemiological Health Investigation of Teenagers in Porto	Community	urban	20-23	20-23	853	895	Digital	
954	Puerto Rico	1974-1977	Puerto Rico Heart Health Program (PRHHP)	Subnational	both	43-88		7,910		Standard	
955	Puerto Rico	2006	Pérez et al., Ethn Dis 2008; 18: 434-41	Community	urban	25-84	25-84	276	532	Unknown	
956	Qatar	2006	GCC	National	both	18+	18+	1,884	2,042	Unknown	
957	Qatar	2012	STEPS	National	both	18-64	18-64	1,039	1,355	Digital	

	Country	Data years	Survey/Study name/Citation	Level of representative-ness	Rural, urban, or both	Age range as used for global analysis		Sample size		Type of blood pressure measurement device[1]	Note
						Male	Female	Male	Female		
958	Romania	1986-1987	MONICA, Bucharest	Community	urban	25-64	25-64	702	873	Standard	
959	Romania	1997	Valorile medii si limitele normalitatii unor constante biologice; Infobase 101221a1	National	both	15-84	15-84	3,964	5,050	Unknown	2
960	Romania	2005	Study for the Evaluation of Prevalence of Hypertension and Cardiovascular Risk in Adult Population in Romania (SEPHAR)	National	both	18+	18+	847	1,170	Digital	
961	Romania	2014	Timis County Study	Community	urban	18-19	18-19	9	6	Standard	
962	Russian Federation	1980	Mirer et al., Ter Arkh 1985; 57: 52-5	Community	urban	40-59	40-59	350	329	Unknown	
963	Russian Federation	1980	Mirer et al., Ter Arkh 1985; 57: 52-5	Community	rural	40-59	40-59	307	259	Unknown	
964	Russian Federation	1984	Konstantinov et al., Kardiologiia 1989; 29: 38-43	Community	urban	20-69	20-69	1,238	1,247	Unknown	
965	Russian Federation	1985	CINDI	Community	both	25-64	25-64	799	800	Standard	
966	Russian Federation	1985	CINDI	Community	both	25-64	25-64	2,327	2,407	Unknown	
967	Russian Federation	1985	CINDI	Community	both	25-64	25-64	839	851	Unknown	
968	Russian Federation	1984-1986	MONICA, Moscow (control)	Community	urban	35-64	35-64	774	642	Standard	
969	Russian Federation	1984-1986	MONICA, Moscow, Leninsky district	Community	urban	35-64	35-64	553	622	Standard	
970	Russian Federation	1984-1986	MONICA, Moscow, Cheremushkinsky district	Community	urban	35-64	35-64	600	600	Standard	
971	Russian Federation	1985	MONICA, Novosibirsk (intervention)	Community	urban	25-64	25-64	797	818	Standard	
972	Russian Federation	1986	INTERSALT	Community	urban	20-59	20-59	97	97	Random-zero	
973	Russian Federation	1985-1986	MONICA, Novosibirsk, Kirowsky district	Community	urban	25-64	25-64	758	774	Standard	
974	Russian Federation	1985-1986	MONICA, Novosibirsk, Leninsky district	Community	urban	25-64	25-64	624	628	Standard	
975	Russian Federation	1988	CINDI	Community	both	25-64	25-64	839	851	Standard	
976	Russian Federation	1988	MONICA, Novosibirsk (intervention)	Community	urban	25-64	25-64	840	853	Standard	
977	Russian Federation	1988-1989	MONICA, Moscow (control)	Community	urban	35-64	35-64	621	581	Standard	
978	Russian Federation	1988-1989	MONICA, Moscow, Leninsky district	Community	urban	35-64	35-64	597	613	Standard	
979	Russian Federation	1988-1989	MONICA, Novosibirsk, Kirowsky district	Community	urban	25-64	25-64	876	805	Standard	
980	Russian Federation	1992	CINDI	Community	rural	25-64	25-64	377	453	Standard	
981	Russian Federation	1992	Puska et al., Int J Epidemiol 1993; 22: 1048-55	Community	both	25-64	25-64	379	458	Unknown	
982	Russian Federation	1992-1995	MONICA, Moscow (control)	Community	urban	35-64	35-64	557	527	Standard	
983	Russian Federation	1992-1995	MONICA, Moscow, Leninsky district	Community	urban	35-64	35-64	538	858	Standard	
984	Russian Federation	1992-1993	Russia Longitudinal Monitoring Survey-Higher School of Economics Round II	National	both	18+	18+	4,395	5,999	Unknown	
985	Russian Federation	1993	Russia Longitudinal Monitoring Survey-Higher School of Economics Round III	National	both	18+	18+	4,559	6,400	Unknown	
986	Russian Federation	1993-1994	Russia Longitudinal Monitoring Survey-Higher School of Economics Round IV	National	both	18+	18+	4,129	5,782	Unknown	
987	Russian Federation	1994	CINDI	Community	both	25-64	25-64	824	868	Standard	
988	Russian Federation	1994-1995	MONICA, Novosibirsk (intervention)	Community	urban	25-64	25-64	822	865	Standard	
989	Russian Federation	1995	MONICA, Novosibirsk, Kirowsky district	Community	urban	25-64	25-64	771	787	Standard	
990	Russian Federation	2002-2005	Health, Alcohol and Psychosocial Factors in Eastern Europe	Community	urban	45-69	45-69	4,205	5,039	Digital	
991	Russian Federation	2007-2010	SAGE	National	both	50+	50+	1,350	2,464	Digital	
992	Russian Federation	2015-2016	Ufa Eye and Medical Study (UEMS)	Subnational	urban	40+	40+	683	1,868	Digital	
993	Rwanda	2012	STEPS	National	both	18-64	18-64	2,492	3,998	Digital	
994	Saint Kitts and Nevis	2007	STEPS	Subnational	both	25-64	25-64	454	794	Digital	
995	Saint Lucia	1981	Khaw and Rose, Int J Epidemiol 1982; 11: 372-7	Subnational	rural	15+	15+	168	191	Random-zero	2
996	Saint Lucia	2012	STEPS	National	both	25-64	25-64	573	918	Digital	
997	Samoa	1979-1982	McGarvey, Am J Clin Nutr 1991; 53: 1586S-94S	National	both	18-79	18+	195	195	Standard	
998	Samoa	1995	McGarvey, Pac Health Dialog 2001; 8: 157-62	National	both	25-69	25-59	143	150	Standard	
999	Samoa	2002	STEPS	National	both	25-64	25-64	1,197	1,346	Digital	
1000	Samoa	2010	Samoan Genome-Wide Association Study	National	both	24-65	24-65	1,396	2,043	Digital	
1001	Samoa	2013	STEPS	National	both	18-64	18-64	604	901	Digital	
1002	Sao Tome and Principe	2009	STEPS	National	both	25-64	25-64	1,047	1,322	Digital	
1003	Saudi Arabia	1989-1994	National Nutrition Survey	National	both	18-39	18-39	1,514	2,599	Standard	
1004	Saudi Arabia	1994	FEPHD; Soyannwo et al., Afr J Med Med Sci 1998; 27: 107-16	Community	both	15+	15+	1,158	1,689	Unknown	2
1005	Saudi Arabia	1995-2000	National Epidemiological Health Survey; Al-Nozha et al., Saudi Med J 25:1603-10, 2004	National	both	30-69	30-69	8,224	9,006	Standard	
1006	Saudi Arabia	2004-2005	STEPS	National	both	15-64	15-64	2,259	2,357	Digital	2
1007	Saudi Arabia	2011-2013	Jeddah City Study	Community	urban	20+	20+	514	639	Standard	
1008	Saudi Arabia	2013	Saudi Health Information Survey	National	both	15+	15+	5,194	5,411	Digital	2
1009	Serbia	1984	MONICA, Novi Sad	Community	urban	25-64	25-64	798	777	Standard	
1010	Serbia	1985	CINDI	Community	urban	25-64	25-64	801	781	Unknown	
1011	Serbia	1987	Serbian Cohorts of Seven Countries Study, Velika Krsna; Kromhout et al., Int J Epidemiol 1994; 23: 5-11	Community	rural	65-69		97		Standard	
1012	Serbia	1988-1989	MONICA, Novi Sad	Community	urban	25-64	25-64	778	791	Standard	
1013	Serbia	1994-1995	MONICA, Novi Sad	Community	urban	25-64	25-64	600	670	Standard	

	Country	Data years	Survey/Study name/Citation	Level of representative-ness	Rural, urban, or both	Age range as used for global analysis		Sample size		Type of blood pressure measurement device[1]	Note
						Male	Female	Male	Female		
1014	Serbia	2000	Health Status, Health Needs and Utilization of Health Care of the Population of Serbia	National	both	20+	20+	4,200	5,257	Unknown	
1015	Serbia	2006	National Health Survey Serbia	National	both	20+	20+	6,640	7,513	Standard	
1016	Serbia	2013	National Health Survey	National	both	15+	15+	6,586	7,704	Digital	2
1017	Serbia	2013-2014	Stay Fit for Lifelong Health; The Prevalence of Lifestyle Health Conditions in Serbian Population	National	urban	18-65		1,367		Digital	
1018	Seychelles	1989	Seychelles Heart Survey I	National	both	25-64	25-64	513	568	Standard	
1019	Seychelles	1994	Seychelles Heart Survey II	National	both	25-64	25-64	504	563	Standard	
1020	Seychelles	2003	School Screening Program	National	both		18-19		2	Digital	
1021	Seychelles	2004	Seychelles Heart Survey III	National	both	25-64	25-64	568	687	Standard	
1022	Seychelles	2013-2014	Seychelles Heart Survey IV	National	both	25-64	25-64	531	699	Standard	
1023	Sierra Leone	1995	Lisk et al., Ethn Dis 1999; 9: 254-63	Community	urban	15+	15+	201	397	Unknown	2
1024	Sierra Leone	1995	Lisk et al., Ethn Dis 1999; 9: 254-63	Community	rural	15+	15+	255	351	Unknown	2
1025	Sierra Leone	2009	STEPS	National	both	25-64	25-64	2,214	2,332	Digital	
1026	Singapore	1982-1985	Thyroid Heart Study	National	both	18+	18+	1,036	995	Standard	
1027	Singapore	1992	National Health Survey	National	both	18-64	18-64	1,744	1,704	Standard	
1028	Singapore	1993-1995	NUH Heart Study	National	both	26+	26-79	498	484	Standard	
1029	Singapore	1998	National Health Survey	National	both	18-69	18-69	2,569	2,830	Standard	
1030	Singapore	2004	National Health Survey	National	both	25-74	25-74	1,801	1,852	Standard	
1031	Singapore	2004-2007	Combined follow up of Singapore Cardiovascular Cohort study and Singapore Prospective study	National	both	24+	24+	2,467	2,666	Standard	
1032	Singapore	2009	Social Isolation, Health and Lifestyles Survey (SIHLS) 2009	National	both	60+	60+	2,050	2,438	Digital	
1033	Singapore	2010	National Health Survey	National	both	30-69	30-69	1,573	1,676	Standard	
1034	Singapore	2009-2011	The Singapore Chinese Eye Study	Community	both	40+	40+	1,662	1,691	Digital	
1035	Singapore	2012-2013	Singapore Health Study	National	both	18-79	18-79	958	1,026	Standard	
1036	Slovakia	1993	Countrywide Integrated Noncommunicable Diseases Intervention Programme	National	both	18-64	18-64	763	1,215	Standard	
1037	Slovakia	1998	Countrywide Integrated Noncommunicable Diseases Intervention Programme	National	both	18-64	18-64	857	1,046	Standard	
1038	Slovakia	2003	Countrywide Integrated Noncommunicable Diseases Intervention Programme	National	both	18-64	18-64	622	867	Standard	
1039	Slovakia	2008	Countrywide Integrated Noncommunicable Diseases Intervention Programme	National	both	18-64	18-64	391	561	Standard	
1040	Slovakia	2011-2012	Europan Health Examination Survey	National	both	18-64	18-64	885	1,080	Digital	
1041	Slovenia	2014	Analysis of Children's Development in Slovenia (ACDSi)	National	urban	18-19	18-19	144	126	Digital	
1042	Solomon Islands	2006	STEPS	Subnational	both	18-64	18-64	990	1,308	Digital	
1043	Solomon Islands	2009-2010	Influences of the large-scale disaster and related socioecological changes on people's nutritional status and health in the Western Solomon Islands	Subnational	rural	18+	18+	153	215	Digital	
1044	Solomon Islands	2009-2010	Influences of the large-scale disaster and related socioecological changes on people's nutritional status and health in the Western Solomon Islands	Community	urban	20-69	18-70	22	58	Digital	
1045	South Africa	1981	Seedat and Hackland, Trans R Soc Trop Med Hyg 1984; 78: 785-9	Community	rural	15+	15+	1,472	3,521	Unknown	2
1046	South Africa	1982	Steyn et al., S Afr Med J 1985; 67: 619-25	Community	rural	15-64	15-64	478	498	Unknown	2
1047	South Africa	1983	Ijsselmuiden, S Afr Med J 1985; 67: 773-5	Community	urban	21-60	21-60	40	392	Unknown	
1048	South Africa	1990	Mollentze et al., S Afr Med J 1995; 85: 90-6; Site 1	Community	both	25+	25+	279	574	Unknown	
1049	South Africa	1990	Mollentze et al., S Afr Med J 1995; 85: 90-6; Site 2	Community	both	25+	25+	290	468	Unknown	
1050	South Africa	1993	Charlton et al., S Afr Med J 1997; 87: 1124-30	Community	urban	65+	65+	96	104	Unknown	
1051	South Africa	1993	CORIS; Steyn et al., Int J Epidemiol 1997; 26: 964-71; Site 1	Community	urban	35-44	35-44	55	51	Unknown	
1052	South Africa	1993	CORIS; Steyn et al., Int J Epidemiol 1997; 26: 964-71; Site 2	Community	urban	35-44	35-44	57	59	Unknown	
1053	South Africa	1993	CORIS; Steyn et al., Int J Epidemiol 1997; 26: 964-71; Site 3	Community	urban	35-44	35-44	51	54	Unknown	
1054	South Africa	1995	Charlton et al., S Afr Med J 1997; 87: 1124-30	Community	urban	65+	65+	66	76	Unknown	
1055	South Africa	1998	DHS	National	both	18+	18+	4,996	7,055	Digital	
1056	South Africa	2001	Alberts et al., Eur J Cardiovasc Prev Rehabil 2005; 12: 347-54	Community	rural	30+	30+	498	1,607	Unknown	
1057	South Africa	2003	SASPI; Thorogood et al., BMC Public Health 2007; 7: 326	Community	rural	35+	35+	82	270	Unknown	
1058	South Africa	2003	DHS	National	both	18+	18+	2,888	4,183	Digital	
1059	South Africa	2003-2004	Africa Centre Biomeasure Survey	Community	rural	25-49	25-49	790	1,758	Digital	
1060	South Africa	2008	National Income Dynamics Study Wave I	National	both	18+	18+	4,855	7,426	Digital	
1061	South Africa	2007-2008	SAGE	National	both	50+	50+	1,583	2,137	Digital	
1062	South Africa	2008-2009	Cape Town Bellville South Cohort Study - Baseline evaluation I	Community	urban	18+	18+	218	707	Digital	
1063	South Africa	2008-2009	Cardiovascular Risk in Black South Africans (CRIBSA) study	Community	urban	25-74	25-74	392	707	Digital	
1064	South Africa	2010	Africa Centre Biomeasure Survey	Community	rural	18+	18+	2,755	7,119	Digital	
1065	South Africa	2010-2011	National Income Dynamics Study Wave II	National	both	18+	18+	30,980	49,186	Digital	
1066	South Africa	2012	National Income Dynamics Study Wave III	National	both	18+	18+	33,284	54,384	Digital	
1067	South Korea	1986	INTERSALT	Community	urban	20-59	20-59	100	98	Random-zero	
1068	South Korea	1998	Korea National Health and Nutrition Examination Survey	National	both	18+	18+	3,741	4,488	Standard	

	Country	Data years	Survey/Study name/Citation	Level of representative-ness	Rural, urban, or both	Age range as used for global analysis		Sample size		Type of blood pressure measurement device[1]	Note
						Male	Female	Male	Female		
1069	South Korea	2001	Kim et al., Br J Psychiatry 2004; 185: 102-7	Community	both	65+	65+	300	432	Unknown	
1070	South Korea	2001	Korea National Health and Nutrition Examination Survey	National	both	18+	18+	2,695	3,467	Standard	
1071	South Korea	2002-2003	Korean National Health Insurance	National	both	40+	40+	64,510	53,449	Unknown	
1072	South Korea	2005	Korea National Health and Nutrition Examination Survey	National	both	18+	18+	2,406	3,174	Standard	
1073	South Korea	2004-2005	Korean National Health Insurance	National	both	40+	40+	76,643	69,279	Unknown	
1074	South Korea	2006	KLoSHA	Community	urban	64+	64+	299	415	Unknown	
1075	South Korea	2007	Korea National Health and Nutrition Examination Survey	National	both	18+	18+	1,282	1,767	Standard	
1076	South Korea	2006-2007	Korean National Health Insurance	National	both	40+	40+	93,208	92,125	Unknown	
1077	South Korea	2008	Korea National Health and Nutrition Examination Survey	National	both	18+	18+	2,902	3,980	Standard	
1078	South Korea	2009	Korea National Health and Nutrition Examination Survey	National	both	18+	18+	3,317	4,274	Standard	
1079	South Korea	2008-2009	Korean National Health Insurance	National	both	40+	40+	115,935	121,788	Unknown	
1080	South Korea	2010	Korea National Health and Nutrition Examination Survey	National	both	18+	18+	2,766	3,593	Standard	
1081	South Korea	2011	Korea National Health and Nutrition Examination Survey	National	both	18+	18+	2,685	3,565	Standard	
1082	South Korea	2010-2011	Korean National Health Insurance	National	both	40+	40+	135,057	143,245	Unknown	
1083	South Korea	2012	Korea National Health and Nutrition Examination Survey	National	both	18+	18+	2,522	3,474	Standard	
1084	South Korea	2013	Korea National Health and Nutrition Examination Survey	National	both	18+	18+	2,508	3,303	Standard	
1085	South Korea	2012-2013	Korean National Health Insurance	National	both	40+	40+	143,709	152,991	Unknown	
1086	Spain	1982	Martinez et al., Rev Clin Esp 1987; 180: 25-31	Community	urban	21+	21+	4,755	5,018	Unknown	
1087	Spain	1984	Tormerl-Soler et al., Rev Clin Esp 1989; 185: 253-6	Community	rural	15+	15+	886	956	Unknown	2
1088	Spain	1985	INTERSALT, Manresa	Community	urban	20-59	20-59	100	100	Random-zero	
1089	Spain	1986	Plans et al., Med Clin (Barc) 1992; 98: 369-72	Community	urban	15+	15+	330	374	Unknown	2
1090	Spain	1986	Custodi et al., Aten Primaria 1989; 6: 151-8	Community	both	20+	20+	321	307	Unknown	
1091	Spain	1986	INTERSALT, Torrejo	Community	urban	20-59	20-59	100	100	Random-zero	
1092	Spain	1986-1988	MONICA, Catalonia	Community	urban	25-64	25-64	1,252	1,271	Random-zero	
1093	Spain	1989	Cardiovascular Risk Study in Catalonia	Subnational	both	20+	20+	310	344	Standard	
1094	Spain	1990	Banegas et al., Hypertension 1998; 32: 998-1002	National	both	35-65	35-65	810	1,211	Random-zero	
1095	Spain	1990-1992	MONICA, Catalonia	Community	urban	25-64	25-64	1,720	1,192	Random-zero	
1096	Spain	1992	CINDI	Subnational	both	25-64	25-64	1,202	1,458	Standard	
1097	Spain	1991-1993	Encuesta de Factores de Riesgo Cardiovascular en la Región de Murcia (Cardiovascular Risk Factors Survey)	Subnational	both	18-69	18-69	1,512	1,563	Standard	
1098	Spain	1994-1995	Encuesta de Nutrición y Salud Comunidad Valenciana 1994-95 (ENCV)	Subnational	both	18+	18+	749	900	Digital	
1099	Spain	1994-1996	MONICA, Catalonia	Community	urban	25-64	25-64	1,802	1,631	Random-zero	
1100	Spain	1996	de Pablos-Velasco et al., J Hypertens 2002; 20: 1965-71	Community	rural	30+	30+	305	385	Unknown	
1101	Spain	1998	Rodriguez and Hoffmann, Lakartidningen 2001; 98: 1964	Subnational	both	18-75	18-75	627	738	Unknown	
1102	Spain	1999-2000	Factores de riesgo en las islas Baleares: Estudio CORSAIB	Subnational	both	35-75	35-75	813	870	Digital	
1103	Spain	2000-2001	Regidor et al., J Hum Hypertens 2006; 20: 73-82	National	both	60+	60+	1,408	2,461	Unknown	
1104	Spain	2000-2001	EUREYE Study	Subnational	both	65+	65+	274	324	Digital	
1105	Spain	2001-2002	Catalan Health Interview Survey	Subnational	both	18-74	18-74	606	760	Digital	
1106	Spain	2001-2003	DIabetes, Nutrición y Obesidad en la población adulta de la Región de Murcia (DINO)	Subnational	both	20+	20+	716	832	Digital	
1107	Spain	2003	Vara-Gonzalez et al., Rev Esp Salud Publica 2007; 81: 211-9	Community	both	18+	18+	550	646	Unknown	
1108	Spain	2000-2005	CDC of the Canary Islands	Community	both	20-75	20-75	2,801	3,651	Digital	
1109	Spain	2003	The European Male Ageing Study (EMAS)	Community	both	40-79		406		Digital	
1110	Spain	2004	Perez-Fernandez et al., J Hum Hypertens 2007; 21: 366-73	Community	rural	25-84	25-84	1,171	1,343	Unknown	
1111	Spain	2004	Vioque et al., Obesity 2008; 16: 664-670	Community	urban	24+	24+	85	116	Digital	
1112	Spain	2004	Cardiovascular Risk Study in Castilla y León	Subnational	both	18+	18+	1,852	2,015	Digital	
1113	Spain	2003-2005	Registre Gironi del Cor (REGICOR)	Subnational	both	35-79	35-79	2,964	3,289	Digital	
1114	Spain	2004-2006	PREVICTUS	National	both	60+	60+	3,416	3,915	Digital	
1115	Spain	2008	The European Male Ageing Study (EMAS)	Community	both	40+		267		Digital	
1116	Spain	2007-2009	Harmonizing Equation of Risk in Mediterraneon countries EXtremadura	Subnational	both	25-79	25-79	1,298	1,498	Digital	
1117	Spain	2008-2010	Study on Nutrition and Cardiovascular Risk in Spain (ENRICA)	National	both	18+	18+	5,792	6,414	Digital	
1118	Spain	2015	Study on Nutrition and Cardiovascular Risk in Spain (ENRICA)	National	both	60+	60+	741	799	Digital	
1119	Sri Lanka	2006	STEPS	National	both	18-64	18-64	5,695	5,931	Digital	
1120	Sudan	2005-2006	STEPS	National	both	25-64	25-64	620	872	Digital	
1121	Swaziland	2014	STEPS	National	both	18-69	18-69	997	1,860	Digital	
1122	Sweden	1972	The Swedish Conscription Study	National	both	17-18		50,215		Standard	2,4
1123	Sweden	1973	The Swedish Conscription Study	National	both	17-18		54,774		Standard	2,4
1124	Sweden	1974	The Swedish Conscription Study	National	both	17-18		56,668		Standard	2,4
1125	Sweden	1975	The Swedish Conscription Study	National	both	17-18		53,048		Standard	2

	Country	Data years	Survey/Study name/Citation	Level of representative-ness	Rural, urban, or both	Age range as used for global analysis		Sample size		Type of blood pressure measurement device[1]	Note
						Male	Female	Male	Female		
1126	Sweden	1976	The Swedish Conscription Study	National	both	17-18		51,609		Standard	2
1127	Sweden	1977	The Swedish Conscription Study	National	both	17-18		49,814		Standard	2
1128	Sweden	1978	The Swedish Conscription Study	National	both	17-18		12,882		Standard	2
1129	Sweden	1979	The Swedish Conscription Study	National	both	17-18		38,280		Standard	2
1130	Sweden	1980	Ohlson et al., Diabetologia 1987; 30: 386-93	Community	urban	67-67		575		Unknown	
1131	Sweden	1980	The Swedish Conscription Study	National	both	17-18		50,577		Standard	2
1132	Sweden	1981	The Swedish Conscription Study	National	both	17-18		53,678		Standard	2
1133	Sweden	1982	Lernfelt et al., J Hypertens 1990; 8: 483-90	Community	urban	70-70	70-70	232	215	Unknown	
1134	Sweden	1982	Cederholm, Ups J Med Sci 1985; 90: 201-27	Community	urban	47-54	47-54	371	436	Unknown	
1135	Sweden	1982	The Swedish Conscription Study	National	both	17-18		55,797		Standard	2
1136	Sweden	1983	Wilhelmsen et al., J Intern Med 2008; 263: 636-43	Community	urban	50-50		776		Unknown	
1137	Sweden	1983	Bengtsson et al., Eur J Vasc Surg 1991; 5: 53-7	Community	urban	74-74		338		Unknown	
1138	Sweden	1983	The Swedish Conscription Study	National	both	17-18		54,567		Standard	2
1139	Sweden	1980-1984	Uppsala Longitudinal Study of Adult Men	Community	both	60-60		1,847		Standard	
1140	Sweden	1984	The Swedish Conscription Study	National	both	17-18		37,404		Standard	2
1141	Sweden	1985	MONICA Gothenburg	Community	urban	25-64	25-64	550	595	Random-zero	
1142	Sweden	1985	The Swedish Conscription Study	National	both	17-18		11,174		Standard	2
1143	Sweden	1986	Bjorkelund and Bengtsson, Scand J Soc Med 1991; 19: 218-24	Community	urban		45-64		927	Unknown	
1144	Sweden	1986	MONICA, Northern Sweden	Subnational	urban	25-64	25-64	820	799	Random-zero	
1145	Sweden	1986	The Swedish Conscription Study	National	both	17-18		46,350		Standard	2
1146	Sweden	1987	The Swedish Conscription Study	National	both	17-18		50,489		Standard	2
1147	Sweden	1988	Ellenius et al., Scand J Prim Health Care 1994; 12: 289-94	Community	urban		40-59		300	Unknown	
1148	Sweden	1988	The Swedish Conscription Study	National	both	17-18		48,944		Standard	2
1149	Sweden	1989	The Swedish Conscription Study	National	both	17-18		49,490		Standard	2
1150	Sweden	1990	CRISS; Henriksson et al., Eur J Epidemiol 2001; 17: 521-6	Community	urban	37-37		991		Unknown	
1151	Sweden	1990	Lind et al., Scand J Clin Lab Invest 1994; 54: 177-83	Community	urban	40-40		314		Unknown	
1152	Sweden	1990	MONICA, Northern Sweden	Subnational	urban	25-64	25-64	764	794	Random-zero	
1153	Sweden	1990	MONICA Gothenburg	Community	urban	25-64	25-64	766	766	Random-zero	
1154	Sweden	1990	The Swedish Conscription Study	National	both	17-18		50,321		Standard	2
1155	Sweden	1991	Asplund-Carlson and Carlson, J Intern Med 1994; 236: 57-64	Community	urban	40-50		1,564		Unknown	
1156	Sweden	1991	The Swedish Conscription Study	National	both	17-18		44,554		Standard	2
1157	Sweden	1992	Women in Gothenburg; Bjorkelund et al., Diabetes Care 2005; 28: 2739-44	Community	urban		38-84		1,042	Unknown	
1158	Sweden	1985-1996	EPIC Umea	Subnational	both	24-72	24-72	12,139	12,985	Standard	
1159	Sweden	1992	The Swedish Conscription Study	National	both	17-18		43,239		Standard	2
1160	Sweden	1993	Wilhelmsen et al., J Intern Med 2008; 263: 636-43	Community	urban	50-50		798		Unknown	
1161	Sweden	1993	The Swedish Conscription Study	National	both	17-18		47,494		Standard	2
1162	Sweden	1991-1995	Uppsala Longitudinal Study of Adult Men	Community	both	70-70		1,216		Standard	
1163	Sweden	1994	Andersson et al., J Hypertens 2000; 18: 1753-61	Community	urban		50-62		876	Unknown	
1164	Sweden	1994	Helicobacter Pylori	Community	urban	56-65	56-65	170	217	Standard	
1165	Sweden	1991-1996	Malmö Diet and Cancer	Community	urban	45-73	45-73	12,101	18,291	Standard	
1166	Sweden	1994	MONICA, Northern Sweden	Subnational	urban	25-64	25-64	736	769	Random-zero	
1167	Sweden	1994	The Swedish Conscription Study	National	both	17-18		44,601		Standard	2
1168	Sweden	1995	Rosmond and Bjorntorp, J Hypertens 1998; 16: 1721-6	Community	urban	51-51		284		Unknown	
1169	Sweden	1995	MONICA Gothenburg	Community	urban	25-64	25-64	741	863	Random-zero	
1170	Sweden	1995	The Swedish Conscription Study	National	both	17-18		44,523		Standard	2
1171	Sweden	1996	The Swedish Conscription Study	National	both	17-18		43,819		Standard	2
1172	Sweden	1997	The Swedish Conscription Study	National	both	17-18		35,848		Standard	2
1173	Sweden	1998	WHILA; Lidfeldt et al., Blood Press 2002; 11: 270-8	Community	urban		50-59		6,901	Unknown	
1174	Sweden	1998	The Swedish Conscription Study	National	both	17-18		39,023		Standard	2
1175	Sweden	1999	MONICA Northern Sweden	Subnational	both	25-74	25-74	889	934	Digital	
1176	Sweden	1999	The Swedish Conscription Study	National	both	17-18		30,185		Standard	2
1177	Sweden	1997-2001	Uppsala Longitudinal Study of Adult Men	Community	both	77-77		782		Standard	
1178	Sweden	2000	Women in Gothenburg; Bjorkelund et al., Diabetes Care 2005; 28: 2739-44	Community	urban		70-92		559	Unknown	
1179	Sweden	2000	The Swedish Conscription Study	National	both	17-18		20,703		Standard	2
1180	Sweden	2001	Gause-Nilsson et al., Acta Diabetol 2006; 43: 120-6	Community	urban	70-70	70-70	243	265	Unknown	
1181	Sweden	2001	Lissner et al., Scand J Prim Health Care 2003; 21: 242-7	Community	urban		70+		494	Unknown	
1182	Sweden	2001	Molander et al., J Am Geriatr Soc 2008; 56: 1853-9	Community	both	85+	85+	98	250	Unknown	

	Country	Data years	Survey/Study name/Citation	Level of representative-ness	Rural, urban, or both	Age range as used for global analysis		Sample size		Type of blood pressure measurement device[1]	Note
						Male	Female	Male	Female		
1183	Sweden	2001-2003	Uppsala Longitudinal Study of Adult Men	Community	both	82-82		524		Standard	
1184	Sweden	2003	Wilhelmsen et al., J Intern Med 2008; 263: 636-43	Community	urban	50-50		595		Unknown	
1185	Sweden	2003	Brohall et al., Diabetes Care 2006; 29: 363-7	Community	urban		64-64		2,382	Unknown	
1186	Sweden	2003	The European Male Ageing Study (EMAS)	Community	both	40+		397		Digital	
1187	Sweden	2001-2004	Swedish INTERGENE Cohort Study	Subnational	both	24-76	24-76	1,691	1,905	Digital	
1188	Sweden	2001-2004	Prospective Investigation of the Vasculature in Uppsala Seniors (PIVUS)	Community	both	70-70	70-70	506	506	Standard	
1189	Sweden	2004	Welin et al., BMC Public Health 2008; 8: 403	Community	urban	50-60	50-50	1,250	667	Unknown	
1190	Sweden	2004	MONICA Northern Sweden	Subnational	both	25-74	25-74	910	957	Digital	
1191	Sweden	2005	Women in Gothenburg; Bjorkelund et al., Diabetes Care 2005; 28: 2739-44	Community	urban		75-87		366	Unknown	
1192	Sweden	2004-2005	European Youth Heart Study (EYHS) II	Subnational	both	20-21	20-21	70	111	Digital	
1193	Sweden	2004-2005	Population Study of Women in Gothenburg	Community	urban		38-50		493	Standard	
1194	Sweden	2008	The European Male Ageing Study (EMAS)	Community	both	40+		370		Digital	
1195	Sweden	2006-2009	Prospective Investigation of the Vasculature in Uppsala Seniors (PIVUS)	Community	both	75-75	75-75	407	418	Standard	
1196	Sweden	2009	MONICA Northern Sweden	Subnational	both	25-74	25-74	850	872	Digital	
1197	Sweden	2014	MONICA Northern Sweden	Subnational	both	25-74	25-74	756	806	Digital	
1198	Switzerland	1984-1986	The Swiss MONICA Study Wave I	Subnational	both	25-74	25-74	1,749	1,692	Random-zero	
1199	Switzerland	1988-1989	The Swiss MONICA Study Wave II	Subnational	both	25-74	25-74	1,775	1,683	Random-zero	
1200	Switzerland	1990	Bodenmann and Ackermann-Liebrich, Schweiz Med Wochenschr Suppl 1993; 48: 38-45	Community	urban	25-74	25-74	252	280	Unknown	
1201	Switzerland	1993	Bus Santé Survey	Community	urban	35-74	35-74	410	279	Unknown	
1202	Switzerland	1992-1993	The Swiss MONICA Study Wave III	Subnational	both	25-74	25-74	1,570	1,670	Random-zero	
1203	Switzerland	1994	Bus Santé Survey	Community	urban	35-74	35-74	300	342	Unknown	
1204	Switzerland	1995	Bus Santé Survey	Community	urban	35-74	35-74	446	462	Unknown	
1205	Switzerland	1996	Bus Santé Survey	Community	urban	35-74	35-74	576	581	Unknown	
1206	Switzerland	1997	Bus Santé Survey	Community	urban	35-74	35-74	593	635	Unknown	
1207	Switzerland	1998	Bus Santé Survey	Community	urban	35-74	35-74	602	534	Unknown	
1208	Switzerland	1999	Bus Santé Survey	Community	urban	35-74	35-74	620	576	Unknown	
1209	Switzerland	2000	Bus Santé Survey	Community	urban	35-74	35-74	562	592	Unknown	
1210	Switzerland	2001	Bus Santé Survey	Community	urban	35-74	35-74	650	635	Unknown	
1211	Switzerland	2002	SAPALDIA 2; Nitsch et al., Nephrol Dial Transplant 2006; 21: 935-44	Subnational	both	55+	55+	1,349	1,404	Unknown	
1212	Switzerland	2003-2006	Cohorte Lausannoise	Community	urban	35-75	35-75	3,185	3,531	Digital	
1213	Switzerland	2007-2012	Bus Santé Study	Subnational	both	20-80	20-80	1,886	1,913	Digital	
1214	Switzerland	2009-2012	Cohorte Lausannoise	Community	urban	40-79	40-79	2,199	2,507	Digital	
1215	Syrian Arab Republic	2002	national survey on non-communicable diseases and factors affecting their development	National	both	15-64	15-64	3,664	5,425	Unknown	2
1216	Taiwan	1985	INTERSALT	Community	rural	20-59	20-59	89	92	Random-zero	
1217	Taiwan	1990	Liau et al., Int J Cardiol 1998; 67: 177-81	Subnational	both	65+	65+	1,283	1,235	Unknown	
1218	Taiwan	1993-1994	The Kinmen Neurological Disorders Survey	Community	urban	50+	50+	1,446	1,339	Standard	
1219	Taiwan	1995	Lu et al., Gerontol A Biol Sci Med Sci 2000; 55: M463-8	Community	urban	65+	65+	477	399	Unknown	
1220	Taiwan	1993-1996	Nutrition and Health Survey in Taiwan (NAHSIT)	National	both	18+	18+	2,588	2,612	Standard	
1221	Taiwan	1999-2000	Nutrition and Health Survey in Taiwan (NAHSIT)	National	both	65+	65+	1,277	1,217	Digital	
1222	Taiwan	2002	Taiwanese Survey on Hypertension, Hyperglycemia and Hyperlipidemia (TwSHHH)	National	both	15+	15+	3,335	3,606	Standard	2
1223	Taiwan	2005	TCHS	Community	urban	40+	40+	1,147	1,212	Unknown	
1224	Taiwan	2005-2008	Nutrition and Health Survey in Taiwan (NAHSIT)	National	both	19+	19+	1,342	1,402	Digital	
1225	Taiwan	2007	Taiwanese Survey on Hypertension, Hyperglycemia and Hyperlipidemia (TwSHHH)	National	both	20+	20+	2,160	2,468	Digital	
1226	Tanzania	1987	CARDIAC Study; Njelekela et al., Acta Tropica 2001; 78: 231-9; Site 1	Community	both	47-57	47-57	88	96	Unknown	
1227	Tanzania	1987	CARDIAC Study; Njelekela et al., Acta Tropica 2001; 78: 231-9; Site 2	Community	both	47-57	47-57	58	64	Unknown	
1228	Tanzania	1987	CARDIAC Study; Njelekela et al., Acta Tropica 2001; 78: 231-9; Site 3	Community	both	47-57	47-57	103	83	Unknown	
1229	Tanzania	1989	Swai et al., Int J Epidemiol 1993; 22: 651-9; Site 1	Subnational	rural	15+	15+	1,472	2,271	Unknown	2
1230	Tanzania	1989	Swai et al., Int J Epidemiol 1993; 22: 651-9; Site 2	Subnational	rural	15+	15+	1,239	1,317	Unknown	2
1231	Tanzania	1989	Swai et al., Int J Epidemiol 1993; 22: 651-9; Site 3	Subnational	rural	15+	15+	430	543	Unknown	2
1232	Tanzania	1996	Edwards et al., J Hypertens 2000; 18: 145-52	Community	urban	15-54	15-54	303	408	Unknown	2
1233	Tanzania	1997	Edwards et al., J Hypertens 2000; 18: 145-52	Community	rural	15-54	15-54	288	404	Unknown	2
1234	Tanzania	1998	CARDIAC Study; Njelekela et al., Acta Tropica 2001; 78: 231-9; Site 4	Community	both	47-57	47-57	93	91	Unknown	
1235	Tanzania	1998	CARDIAC Study; Njelekela et al., Acta Tropica 2001; 78: 231-9; Site 5	Community	both	47-57	47-57	41	61	Unknown	
1236	Tanzania	1998	CARDIAC Study; Njelekela et al., Acta Tropica 2001; 78: 231-9; Site 6	Community	both	47-57	47-57	81	79	Unknown	
1237	Tanzania	1998-1999	Bovet et al., Int J Epidemiol 2002; 31: 240-7	Community	urban	25-64	25-64	3,600	5,654	Digital	
1238	Tanzania	2011	STEPS	Subnational	both	25-64	25-64	1,008	1,508	Digital	
1239	Tanzania	2012	STEPS	National	both	25-64	25-64	2,585	2,834	Digital	

	Country	Data years	Survey/Study name/Citation	Level of representative-ness	Rural, urban, or both	Age range as used for global analysis		Sample size		Type of blood pressure measurement device[1]	Note
						Male	Female	Male	Female		
1240	Tanzania	2014	Dar es Salaam Urban Cohort Hypertension Study	Community	urban	40+	40+	971	1,271	Digital	
1241	Thailand	1991	Thailand National Health Examination Survey I	National	both	18+	18+	5,809	8,208	Standard	
1242	Thailand	1997	Thailand National Health Examination Survey II	National	both	18+	18+	2,953	4,398	Digital	
1243	Thailand	2000	The International Collaborative Study of Cardiovascular Disease in Asia (InterASIA)	National	both	35+	35+	2,093	3,212	Standard	
1244	Thailand	2004	Thailand National Health Examination Survey III	National	both	18+	18+	18,524	19,962	Standard	
1245	Thailand	2003-2004	The Fifth National Nutrition Survey of Thailand	National	both	15+	15+	1,999	3,406	Unknown	2
1246	Thailand	2009	Thailand National Health Examination Survey IV	National	both	15+	15+	9,718	10,615	Digital	2
1247	Timor-Leste	2014	STEPS	National	both	18-69	18-69	1,066	1,452	Digital	
1248	Togo	2010	STEPS	National	both	18-64	18-64	1,895	1,952	Digital	
1249	Tokelau	2005	STEPS	National	both	18-64	18-64	241	266	Digital	
1250	Tonga	2004	STEPS	National	both	18-64	18-64	391	548	Digital	
1251	Tonga	2011	STEPS	National	both	20-64	20-64	884	1,412	Digital	
1252	Trinidad and Tobago	1985	INTERSALT	Community	urban	20-59	20-59	84	92	Random-zero	
1253	Trinidad and Tobago	2001	Adult Survey	National	rural	25+	25+	203	267	Digital	
1254	Tunisia	1989	Gharbi et al., Rev Epidemiol Sante Publique 1996; 44: 125-32	Community	rural	35-50	35-50	172	210	Unknown	
1255	Tunisia	1989	Gharbi et al., Rev Epidemiol Sante Publique 1996; 44: 125-32	Community	urban	35-50	35-50	148	157	Unknown	
1256	Tunisia	1996	Ghannem and Fredj, Rev Epidemiol Sante Publique 1997; 45: 286-92	Community	urban	20+	20+	290	667	Unknown	
1257	Tunisia	1996-1997	Ariana Healthy Project 1997	Community	both	35-65	35-65	2,655	2,724	Standard	
1258	Tunisia	1996-1997	Tunisian National Nutrition Survey 1996-1997	National	both	18+	18+	1,397	2,674	Standard	
1259	Tunisia	2000	Laouani et al., Tunis Med 2004; 82: 1001-5	Community	urban	60+	60+	260	340	Unknown	
1260	Tunisia	2001	Ben Romdhane et al., Tunis Med 2005; 83 Suppl 5: 41-6	Community	both	40-69	40-69	917	919	Unknown	
1261	Tunisia	2005	Mode de vie et santé de l'adolescent, Le cas de la Tunisie dans le contexte de la transition épidémiologique	National	both	18-19	18-19	451	583	Standard	
1262	Tunisia	2005	Tunisian National Survey	National	both	35-71	35-71	3,417	4,590	Standard	
1263	Tunisia	2009-2010	ObeMaghreb	Subnational	urban	18-49	18-49	998	696	Standard	
1264	Turkey	1990	Turkish Adult Risk Factor Study	National	both	20+	20+	1,344	1,372	Standard	
1265	Turkey	1995	Turkish Adult Risk Factor Study	National	both	25+	25+	864	894	Standard	
1266	Turkey	1998	Turkish Adult Risk Factor Study	National	both	28+	28+	883	915	Standard	
1267	Turkey	1999	Tugay Aytekin et al., Health Soc Care Community 2002; 10: 394-401	Subnational	urban	30+	30+	952	1,040	Unknown	
1268	Turkey	2000	Turkish Adult Risk Factor Study	National	both	30+	30+	905	952	Standard	
1269	Turkey	2001	The Healthy Nutrition for Healthy Heart Study; Sanisoglu et al., BMC Public Health 2006; 6: 92	National	both	25+	25+	4,733	5,425	Unknown	
1270	Turkey	2002	Soysal et al., Anadolu Kardiyol Derg 2005; 5: 196-201	Subnational	urban	20-39	20-39	318	567	Unknown	
1271	Turkey	2002	Erem et al., Obes Res 2004; 12: 1117-27	Community	urban	25-84	25-84	2,081	2,349	Unknown	
1272	Turkey	2002	Onal et al., Blood Press 2004; 13: 31-6	Subnational	urban	25-74	25-84	62	355	Unknown	
1273	Turkey	2001-2002	Turkish Adult Risk Factor Study	National	both	32+	32+	1,137	1,252	Standard	
1274	Turkey	2003	Prevalence, awareness, treatment and control of hypertension in Turkey in 2003	National	both	18+	18+	2,019	2,891	Random-zero	
1275	Turkey	2004	Trabzon Hypertension Study; Erem	Community	urban	25-84	25-84	1,906	2,219	Unknown	
1276	Turkey	2003-2004	Turkish Adult Risk Factor Study	National	both	34+	34+	1,106	1,150	Standard	
1277	Turkey	2003-2005	Prevalence of diabetes and associated risk factors among adult population in Trabzon city	Subnational	both	20+	20+	2,208	2,601	Standard	
1278	Turkey	2005-2006	Turkish Adult Risk Factor Study	National	both	33+	33+	1,031	1,091	Standard	
1279	Turkey	2007-2008	Turkish Adult Risk Factor Study	National	both	35+	35+	1,135	1,148	Standard	
1280	Turkey	2009-2010	Turkish Adult Risk Factor Study	National	both	37+	37+	1,490	1,555	Standard	
1281	Turkey	2011	Chronic Disease Risk Factor Survey	National	both	15+	15+	7,995	8,906	Digital	2
1282	Turkey	2012-2013	Turkish Adult Risk Factor Study	National	both	37+	40+	1,030	1,110	Standard	
1283	Turkey	2014	Turkish Adult Risk Factor Study	National	both	44+	44+	345	369	Standard	
1284	Turkmenistan	2013	STEPS	National	both	18-64	18-64	1,931	2,887	Digital	
1285	Tuvalu	1976	The Funafuti Survey	Subnational	urban	18+	18-79	280	290	Unknown	
1286	Uganda	2011-2012	The Prevalence and Distribution of Non-communicable Diseases and Their Risk Factors in Kasese District, Uganda	Subnational	both	25-79	25-79	284	234	Digital	
1287	Uganda	2014	STEPS	National	both	18-69	18-69	1,570	2,132	Digital	
1288	Ukraine	1995	CINDI	Community	both	25-64	25-64	788	892	Standard	
1289	Ukraine	2007	DHS	National	both	18-49	18-49	2,327	5,125	Digital	
1290	United Arab Emirates	1990	el Mugamer et al., J Trop Med Hyg 1995; 98: 407-15	Community	both	20-59	20-59	109	178	Unknown	
1291	United Arab Emirates	1999-2000	Emirates National Diabetes and Coronary Artery Disease Risk Factor Study; Malik et al., Diabetes Res Clin Pract 2005; 69: 188-95	National	both	20-79	20-79	2,839	3,773	Standard	
1292	United Kingdom	1980	Islington Diabetes Survey; Forrest et al., Diabet Med 1986; 3: 338-42	Community	urban	40+	40+	465	619	Unknown	
1293	United Kingdom	1982	MRC National Survey of Health and Development	National	both	36-36	36-36	1,639	1,651	Random-zero	
1294	United Kingdom	1983-1984	MONICA, Belfast	Subnational	both	25-64	25-64	1,160	1,184	Random-zero	
1295	United Kingdom	1985-1986	INTERSALT, Belfast	Community	urban	20-59	20-59	99	100	Random-zero	

	Country	Data years	Survey/Study name/Citation	Level of representative-ness	Rural, urban, or both	Age range as used for global analysis		Sample size		Type of blood pressure measurement device[1]	Note
						Male	Female	Male	Female		
1296	United Kingdom	1985	INTERSALT, Birmingham	Community	urban	20-59	20-59	100	100	Random-zero	
1297	United Kingdom	1985	INTERSALT, Wales	Community	urban	20-59	20-59	100	99	Random-zero	
1298	United Kingdom	1984-1986	Scottish Heart Health Survey	Subnational	both	40-59	40-59	4,385	4,471	Standard	
1299	United Kingdom	1986-1987	Dietary and Nutritional Survey of British Adults (DNS)	National	both	18-64	18-64	1,133	1,144	Digital	
1300	United Kingdom	1986-1987	MONICA, Belfast	Subnational	both	25-64	25-64	1,164	1,185	Random-zero	
1301	United Kingdom	1987-1988	Edinburgh Artery Study	Community	urban	54-74	54-74	808	783	Random-zero	
1302	United Kingdom	1989	MRC National Survey of Health and Development	National	both	43-43	43-43	1,596	1,588	Random-zero	
1303	United Kingdom	1991-1992	Health Survey for England	National	both	18+	18+	2,863	3,226	Digital	
1304	United Kingdom	1991-1992	MONICA, Belfast	Subnational	both	25-64	25-64	999	997	Random-zero	
1305	United Kingdom	1992	MONICA, Glasgow	Community	urban	25-64	25-64	704	779	Random-zero	
1306	United Kingdom	1993	Scottish Heart Health Study; Vanderpump et al., Diabet Med 1996; 13: 741-7	Community	urban	35+	35+	724	895	Unknown	
1307	United Kingdom	1992-1994	Edinburgh Artery Study	Community	urban	60+	60+	580	580	Random-zero	
1308	United Kingdom	1993	Health Survey for England	National	both	18+	18+	6,538	7,293	Digital	
1309	United Kingdom	1994	Health Survey for England	National	both	18+	18+	6,053	6,993	Digital	
1310	United Kingdom	1993-1997	EPIC Norfolk	Subnational	both	40-79	40-79	11,551	13,961	Digital	
1311	United Kingdom	1995	Health Survey for England	National	both	18+	18+	6,159	7,107	Digital	
1312	United Kingdom	1995	MONICA, Glasgow	Community	urban	25-64	25-64	857	958	Random-zero	
1313	United Kingdom	1994-1995	National Diet and Nutrition Survey (NDNS)	National	both	65+	65+	743	730	Digital	
1314	United Kingdom	1995	Scottish Health Survey (SHeS)	Subnational	both	18-64	18-64	3,015	3,655	Digital	
1315	United Kingdom	1996	Health Survey for England	National	both	18+	18+	6,359	7,392	Digital	
1316	United Kingdom	1993-2000	EPIC Oxford	Subnational	both	20-98	20-98	2,443	8,922	Digital	
1317	United Kingdom	1997	Health Survey for England	National	both	18+	18+	3,380	3,866	Digital	
1318	United Kingdom	1997	National Diet and Nutrition Survey (NDNS)	National	both	18-18	18-18	49	52	Digital	
1319	United Kingdom	1998	Health Survey for England	National	both	18+	18+	5,947	7,035	Digital	
1320	United Kingdom	1998-1999	INTERMAP, Belfast	Community	urban	40-59	40-59	125	97	Random-zero	
1321	United Kingdom	1997-1999	INTERMAP, WestBromwich	Community	urban	40-59	40-59	141	138	Random-zero	
1322	United Kingdom	1998	Scottish Health Survey (SHeS)	Subnational	both	18-74	18-74	3,184	3,995	Digital	
1323	United Kingdom	1998-2000	The British Regional Heart Study	National	urban	60-79		4,141		Digital	
1324	United Kingdom	1999	MRC National Survey of Health and Development	National	both	53-53	53-53	1,452	1,478	Digital	
1325	United Kingdom	1999-2001	British Women's Heart and Health Study	National	both		60-79		3,801	Digital	
1326	United Kingdom	1999-2001	Edinburgh Artery Study	Community	urban	66-87	66-87	373	404	Random-zero	
1327	United Kingdom	2000	Health Survey for England	National	both	18+	18+	3,199	4,118	Digital	
1328	United Kingdom	1999-2004	Hertfordshire Cohort Study	Subnational	both	59-73	60-73	1,574	1,412	Digital	
1329	United Kingdom	2001	Health Survey for England	National	both	18+	18+	5,402	6,449	Digital	
1330	United Kingdom	2000-2001	National Diet and Nutrition Survey (NDNS)	National	both	19-64	19-64	797	939	Digital	
1331	United Kingdom	2002	Health Survey for England	National	both	18+	18+	3,163	4,021	Digital	
1332	United Kingdom	2003	The European Male Ageing Study (EMAS)	Community	both	40+		395		Digital	
1333	United Kingdom	2003	Health Survey for England	National	both	18+	18+	4,897	5,945	Digital	
1334	United Kingdom	2003	Scottish Health Survey (SHeS)	Subnational	both	18+	18+	2,311	2,901	Digital	
1335	United Kingdom	2004-2005	English Longitudinal Study of Ageing Wave 2	National	both	52+	52+	3,404	4,159	Digital	
1336	United Kingdom	2005	Health Survey for England	National	both	18+	18+	3,214	3,906	Digital	
1337	United Kingdom	2006	Health Survey for England	National	both	18+	18+	4,512	5,434	Digital	
1338	United Kingdom	2007	Health Survey for England	National	both	18+	18+	2,121	2,596	Digital	
1339	United Kingdom	2008	The European Male Ageing Study (EMAS)	Community	both	40+		315		Digital	
1340	United Kingdom	2008	Health Survey for England	National	both	18+	18+	4,572	5,607	Digital	
1341	United Kingdom	2008	Scottish Health Survey (SHeS)	Subnational	both	18+	18+	485	587	Digital	
1342	United Kingdom	2008-2009	English Longitudinal Study of Ageing Wave 4	National	both	50+	50+	3,824	4,622	Digital	
1343	United Kingdom	2009	Health Survey for England	National	both	18+	18+	1,416	1,686	Digital	
1344	United Kingdom	2006-2010	MRC National Survey of Health and Development	National	both	60-64	60-64	1,065	1,151	Digital	
1345	United Kingdom	2009	Scottish Health Survey (SHeS)	Subnational	both	18+	18+	467	614	Digital	
1346	United Kingdom	2010	Health Survey for England	National	both	18+	18+	2,311	2,994	Digital	
1347	United Kingdom	2008-2012	National Diet and Nutrition Survey (NDNS)	National	both	18+	18+	704	918	Digital	
1348	United Kingdom	2010	Scottish Health Survey (SHeS)	Subnational	both	18+	18+	434	575	Digital	
1349	United Kingdom	2011	Health Survey for England	National	both	18+	18+	2,405	3,064	Digital	
1350	United Kingdom	2011	Scottish Health Survey (SHeS)	Subnational	both	18+	18+	415	529	Digital	
1351	United Kingdom	2012	Health Survey for England	National	both	18+	18+	2,297	2,946	Digital	
1352	United Kingdom	2012	Scottish Health Survey (SHeS)	Subnational	both	18+	18+	421	529	Digital	

	Country	Data years	Survey/Study name/Citation	Level of representative-ness	Rural, urban, or both	Age range as used for global analysis		Sample size		Type of blood pressure measurement device[1]	Note
						Male	Female	Male	Female		
1353	United Kingdom	2012-2013	English Longitudinal Study of Ageing Wave 6	National	both	50+	50+	3,527	4,283	Digital	
1354	United Kingdom	2013	Health Survey for England	National	both	18+	18+	2,633	3,281	Digital	
1355	United Kingdom	2013	Scottish Health Survey (SHeS)	Subnational	both	18+	18+	514	646	Digital	
1356	United Kingdom	2014	Health Survey for England	National	both	18+	18+	2,358	2,918	Digital	
1357	United Kingdom	2014	Scottish Health Survey (SHeS)	Subnational	both	18+	18+	536	695	Digital	
1358	United States of America	1971-1975	US NHANES I	National	both	25-74	25-74	3,141	3,657	Standard	4
1359	United States of America	1975-1978	Framingham Heart Study (FHS)	Community	urban	55-86	55-88	1,142	1,608	Standard	
1360	United States of America	1977-1979	Framingham Heart Study (FHS)	Community	urban	57-88	57-89	1,011	1,494	Standard	
1361	United States of America	1976-1980	US NHANES II	National	both	18-74	18-74	5,909	6,565	Standard	
1362	United States of America	1978-1979	Bogalusa Heart Study (BHS)	Community	rural	18-21	18-21	44	35	Standard	
1363	United States of America	1979-1980	MONICA, Stanford	Subnational	urban	25-64	25-64	674	761	Standard	
1364	United States of America	1979-1982	Framingham Heart Study (FHS)	Community	urban	59-90	59-91	897	1,335	Standard	
1365	United States of America	1979-1983	Framingham Heart Study (FHS) - Offspring Cohort	Community	urban	21-68	21-68	1,823	1,950	Standard	
1366	United States of America	1981-1982	Bogalusa Heart Study (BHS)	Community	rural	18-21	18-21	38	26	Standard	
1367	United States of America	1982-1983	Established Populations for Epidemiologic Studies of the Elderly, Baseline, East Boston	Community	urban	65+	65+	1,391	2,266	Standard	
1368	United States of America	1982-1983	Established Populations for Epidemiologic Studies of the Elderly, Baseline, Iowa	Subnational	both	65+	65+	1,153	1,952	Standard	
1369	United States of America	1982-1983	Established Populations for Epidemiologic Studies of the Elderly, Baseline, New Haven	Community	urban	65+	65+	1,112	1,557	Standard	
1370	United States of America	1981-1984	Framingham Heart Study (FHS)	Community	urban	61-92	60-93	800	1,221	Standard	
1371	United States of America	1983-1985	Bogalusa Heart Study (BHS)	Community	rural	18-21	18-21	50	27	Standard	
1372	United States of America	1983-1985	Framingham Heart Study (FHS)	Community	urban	62-93	61-95	676	1,028	Standard	
1373	United States of America	1983-1987	Framingham Heart Study (FHS) - Offspring Cohort	Community	urban	24-72	24-72	1,819	1,965	Standard	
1374	United States of America	1985-1986	Coronary Artery Risk Development in Young Adults (CARDIA)	Subnational	urban	18-30	18-30	2,327	2,785	Random-zero	
1375	United States of America	1985-1986	Established Populations for Epidemiologic Studies of the Elderly, 3rd Follow-up, East Boston	Community	urban	68+	68+	1,002	1,709	Standard	
1376	United States of America	1985-1986	Established Populations for Epidemiologic Studies of the Elderly, 3rd Follow-up, Iowa	Subnational	both	68+	68+	1,020	1,814	Standard	
1377	United States of America	1985-1986	Established Populations for Epidemiologic Studies of the Elderly, 3rd Follow-up, New Haven	Community	urban	68+	68+	784	1,202	Standard	
1378	United States of America	1985-1986	INTERSALT, Chicago	Community	urban	20-59	20-59	97	99	Random-zero	
1379	United States of America	1986	INTERSALT, Goodman	Community	urban	20-59	20-59	192	192	Random-zero	
1380	United States of America	1986	INTERSALT, Jackson	Community	urban	20-59	20-59	184	199	Random-zero	
1381	United States of America	1985-1986	MONICA, Stanford	Subnational	urban	25-64	25-64	703	824	Standard	
1382	United States of America	1986-1987	Established Populations for Epidemiologic Studies of the Elderly, Baseline, Duke	Subnational	both	65+	65+	1,372	2,521	Standard	
1383	United States of America	1985-1988	Framingham Heart Study (FHS)	Community	urban	65-93	65-96	559	881	Standard	
1384	United States of America	1987-1989	Atherosclerosis Risk in Communities Study (ARIC)	Subnational	both	44-66	44-66	8,153	6,784	Random-zero	
1385	United States of America	1987-1988	Bogalusa Heart Study (BHS)	Community	rural	18-21	18-21	40	28	Standard	
1386	United States of America	1987-1988	Coronary Artery Risk Development in Young Adults (CARDIA)	Subnational	urban	20-32	20-32	2,089	2,531	Random-zero	
1387	United States of America	1986-1990	Framingham Heart Study (FHS)	Community	urban	68-95	67-97	499	802	Standard	
1388	United States of America	1988-1989	Established Populations for Epidemiologic Studies of the Elderly, 6th Follow-up, East Boston	Community	urban	71+	71+	617	1,193	Standard	
1389	United States of America	1988-1989	Established Populations for Epidemiologic Studies of the Elderly, 6th Follow-up, Iowa	Subnational	both	71+	71+	757	1,428	Standard	
1390	United States of America	1988-1989	Established Populations for Epidemiologic Studies of the Elderly, 6th Follow-up, New Haven	Community	urban	71+	71+	520	906	Standard	
1391	United States of America	1987-1991	Framingham Heart Study (FHS) - Offspring Cohort	Community	urban	28-76	28-76	1,886	2,039	Standard	
1392	United States of America	1989-1990	Cardiovascular Health Study (CHS), baseline	Subnational	both	65+	65+	2,449	3,299	Random-zero	
1393	United States of America	1989-1990	Established Populations for Epidemiologic Studies of the Elderly, 3rd Follow-up, Duke	Subnational	both	68+	68+	1,018	2,084	Standard	
1394	United States of America	1988-1992	Framingham Heart Study (FHS)	Community	urban	69-97	69-99	430	780	Standard	
1395	United States of America	1989-1990	MONICA, Stanford	Subnational	urban	25-64	25-64	655	754	Standard	
1396	United States of America	1990-1992	Atherosclerosis Risk in Communities Study (ARIC)	Subnational	both	46-70	47-70	7,414	6,121	Random-zero	
1397	United States of America	1990-1991	Coronary Artery Risk Development in Young Adults (CARDIA)	Subnational	urban	23-35	23-35	1,958	2,389	Random-zero	
1398	United States of America	1988-1994	US NHANES III	National	both	18+	18+	8,772	9,644	Standard	
1399	United States of America	1990-1994	Framingham Heart Study (FHS)	Community	urban	72-99	72-101	367	692	Standard	
1400	United States of America	1992-1994	Bogalusa Heart Study (BHS)	Community	rural	18-20	18-20	53	35	Standard	
1401	United States of America	1992-1993	Coronary Artery Risk Development in Young Adults (CARDIA)	Subnational	urban	25-37	25-37	1,836	2,246	Random-zero	
1402	United States of America	1992-1993	Cardiovascular Health Study (CHS),	Subnational	both	67+	67+	2,010	2,637	Random-zero	
1403	United States of America	1992-1993	Established Populations for Epidemiologic Studies of the Elderly, 6th Follow-up, Duke	Subnational	both	71+	71+	755	1,620	Standard	
1404	United States of America	1991-1995	Framingham Heart Study (FHS) - Offspring Cohort	Community	urban	31-78	31-78	1,742	1,969	Standard	
1405	United States of America	1993-1995	Atherosclerosis Risk in Communities Study (ARIC)	Subnational	both	50-73	49-73	6,587	5,528	Random-zero	
1406	United States of America	1993-1994	Cardiovascular Health Study (CHS)	Subnational	both	65+	65+	1,935	2,583	Random-zero	
1407	United States of America	1992-1996	Framingham Heart Study (FHS)	Community	urban	74-97	73-101	305	608	Standard	
1408	United States of America	1994-1995	Cardiovascular Health Study (CHS)	Subnational	both	66-90	66-90	1,787	2,435	Random-zero	
1409	United States of America	1995-1996	Bogalusa Heart Study (BHS)	Community	rural	20-37	20-37	549	843	Standard	

	Country	Data years	Survey/Study name/Citation	Level of representative-ness	Rural, urban, or both	Age range as used for global analysis		Sample size		Type of blood pressure measurement device[1]	Note
						Male	Female	Male	Female		
1410	United States of America	1995-1996	Coronary Artery Risk Development in Young Adults (CARDIA)	Subnational	urban	28-40	28-40	1,754	2,182	Random-zero	
1411	United States of America	1995-1996	Cardiovascular Health Study (CHS)	Subnational	both	66+	66+	1,830	2,603	Random-zero	
1412	United States of America	1993-1998	Women's Health Initiative Study (WHI OS)	National	both		50-79		93,545	Standard	
1413	United States of America	1996-1998	Atherosclerosis Risk in Communities Study (ARIC)	Subnational	both	53-75	52-75	6,020	4,880	Random-zero	
1414	United States of America	1996-1997	Cardiovascular Health Study (CHS)	Subnational	both	67+	67+	1,701	2,523	Random-zero	
1415	United States of America	1995-1998	Framingham Heart Study (FHS)	Community	urban	76-95	76-103	236	475	Standard	
1416	United States of America	1995-1998	Framingham Heart Study (FHS) - Offspring Cohort	Community	urban	37-80	37-80	1,603	1,829	Standard	
1417	United States of America	1996-1997	INTERMAP, Baltimore	Community	urban	40-59	40-59	146	134	Random-zero	
1418	United States of America	1997-1998	INTERMAP, CC	Community	urban	40-59	40-59	271	276	Random-zero	
1419	United States of America	1997-1998	INTERMAP, Chicago	Community	urban	40-59	40-59	156	159	Random-zero	
1420	United States of America	1996-1997	INTERMAP, Jackson	Community	urban	40-59	40-59	132	134	Random-zero	
1421	United States of America	1996-1998	INTERMAP, Minneapolis	Community	urban	40-59	40-59	130	130	Random-zero	
1422	United States of America	1996-1997	INTERMAP, Pittsburgh	Community	urban	40-59	40-59	132	128	Random-zero	
1423	United States of America	1996-1997	Study of Women's Health Across the Nation	National	both		40-55		3,233	Standard	
1424	United States of America	1997-1999	Framingham Heart Study (FHS)	Community	urban	78-98	78-104	215	450	Standard	
1425	United States of America	1997-1999	Study of Women's Health Across the Nation	National	both		40-55		2,840	Standard	
1426	United States of America	1998-1999	Cardiovascular Health Study (CHS)	Subnational	both	69+	69+	1,406	2,158	Random-zero	
1427	United States of America	1998-2000	Study of Women's Health Across the Nation	National	both		40-55		2,661	Standard	
1428	United States of America	1999-2000	Cardiovascular Health Study (CHS)	Subnational	both	70+	70+	1,288	2,024	Random-zero	
1429	United States of America	1999-2001	Framingham Heart Study (FHS)	Community	urban	80-96	80-103	153	335	Standard	
1430	United States of America	1998-2001	Framingham Heart Study (FHS) - Offspring Cohort	Community	urban	37-89	33-90	1,601	1,887	Standard	
1431	United States of America	1999-2000	US NHANES	National	both	18+	18+	2,272	2,251	Standard	
1432	United States of America	1999-2001	Study of Women's Health Across the Nation	National	both		40-56		2,526	Standard	
1433	United States of America	2000-2001	Coronary Artery Risk Development in Young Adults (CARDIA)	Subnational	urban	33-45	33-45	1,614	2,043	Random-zero	
1434	United States of America	2000-2001	Cardiovascular Health Study (CHS)	Subnational	both	71+	71+	1,185	1,900	Random-zero	
1435	United States of America	2000-2002	Multi-Ethnic Study of Atherosclerosis (MESA)	Subnational	both	45-84	45-84	3,210	3,596	Digital	
1436	United States of America	2000-2002	Study of Women's Health Across the Nation	National	both		40-57		2,457	Standard	
1437	United States of America	2001-2002	US NHANES	National	both	18+	18+	2,545	2,415	Standard	
1438	United States of America	2002-2003	Framingham Heart Study (FHS)	Community	urban	83-99	83-106	116	269	Standard	
1439	United States of America	2002-2004	Multi-Ethnic Study of Atherosclerosis (MESA)	Subnational	both	46-87	46-86	2,969	3,261	Digital	
1440	United States of America	2002-2005	Framingham Heart Study (FHS) - Third Generation Cohort	Community	urban	19-72	19-70	1,903	2,173	Standard	
1441	United States of America	2003-2004	US NHANES	National	both	18+	18+	2,388	2,289	Standard	
1442	United States of America	2004-2005	Framingham Heart Study (FHS)	Community	urban	85-101	85-102	91	188	Standard	
1443	United States of America	2004-2005	Multi-Ethnic Study of Atherosclerosis (MESA)	Subnational	both	47-88	47-87	2,809	3,128	Digital	
1444	United States of America	2005-2007	Multi-Ethnic Study of Atherosclerosis (MESA)	Subnational	both	49-90	49-89	2,691	3,008	Digital	
1445	United States of America	2005-2006	US NHANES	National	both	18+	18+	2,418	2,278	Standard	
1446	United States of America	2005-2006	National Social Life Health and Aging Project	National	both	57-85	57-85	1,423	1,511	Digital	
1447	United States of America	2005-2008	Framingham Heart Study (FHS) - Offspring Cohort	Community	urban	43-92	40-93	1,349	1,648	Standard	
1448	United States of America	2007-2008	US NHANES	National	both	18+	18+	2,815	2,828	Standard	
1449	United States of America	2008-2009	National Longitudinal Study of Adolescent Health Wave IV	National	both	24-34	24-34	2,285	2,698	Digital	
1450	United States of America	2009-2010	US NHANES	National	both	18+	18+	2,975	3,029	Standard	
1451	United States of America	2010-2011	National Social Life Health and Aging Project	National	both	50-99	36-99	1,485	1,791	Digital	
1452	United States of America	2011-2012	US NHANES	National	both	18+	18+	2,676	2,627	Standard	
1453	United States of America	2013-2014	US NHANES	National	both	18+	18+	2,737	2,915	Standard	
1454	Uruguay	2006	STEPS	National	both	25-64	25-64	261	644	Digital	
1455	Uruguay	2011-2012	Detection and follow-up of cardiovascular disease and risk factors in the Southern Cone of Latin America. The CESCAS I Study	Community	urban	35-74	35-74	652	932	Standard	
1456	Uruguay	2012-2016	Genotype, Phenotype and Environment of Hypertension in Uruguay (GEFA-HT-UY)	Community	urban	19+	20+	128	190	Standard	
1457	Uzbekistan	2002	DHS	National	both	18-59	18-49	2,060	4,530	Standard	
1458	Uzbekistan	2014	STEPS	National	both	18-64	18-64	1,539	2,170	Digital	
1459	Vanuatu	1998	1998 Vanuatu Non-Comunicable Disease Survey	National	both	20-59	20-59	734	735	Unknown	
1460	Vanuatu	2005	STEPS	Subnational	both	18-60	18-60	588	705	Digital	
1461	Vanuatu	2011	STEPS	National	both	25-64	25-64	2,283	2,205	Digital	
1462	Venezuela	1998	Sulbaran et al., J Hum Hypertens 2000; 14 Suppl 1: S6-9	Community	urban	20-59	20-59	3,272	3,368	Unknown	
1463	Venezuela	2000	Zulia Coronary Heart Disease Risk Factor Study; Florez et al., Diabetes Res Clin Pract 2005; 69: 63-77	Subnational	both	25+	25+	1,281	2,900	Unknown	
1464	Venezuela	2004-2005	CArdiovascular Risk factors Multiple Evaluation in Latin America (CARMELA)	Community	urban	25-64		713		Standard	
1465	Venezuela	2005-2006	Brajkovich et al., Rev Ven Endoc Metab 2006; 4: 31-2	Community	urban	20-65	20-65	200	432	Standard	

	Country	Data years	Survey/Study name/Citation	Level of representative-ness	Rural, urban, or both	Age range as used for global analysis		Sample size		Type of blood pressure measurement device[1]	Note
						Male	Female	Male	Female		
1466	Venezuela	2007-2008	Venezuelan Study of Metabolic Syndrome, Obesity and Lifestyle (VEMSOLS)	Community	urban	20-79	20+	107	231	Standard	
1467	Venezuela	2008-2009	Venezuelan Study of Metabolic Syndrome, Obesity and Lifestyle (VEMSOLS)	Community	rural	20+	20-79	51	87	Standard	
1468	Venezuela	2010-2011	Venezuelan Study of Metabolic Syndrome, Obesity and Lifestyle (VEMSOLS)	Community	urban	20+	20+	66	193	Standard	
1469	Viet Nam	2001-2002	Vietnam National Health Survey	National	both	18+	18+	42,814	49,431	Standard	
1470	Viet Nam	2005	National Adult Overweight Survey	National	both	25-64	25-64	8,474	8,725	Standard	
1471	Viet Nam	2005	Non-communicable disease risk factors in Ho Chi Minh City	Community	both	25-64	25-64	906	1,058	Digital	
1472	Viet Nam	2008-2009	The survey on diabetes and its risk factors in 2 northern provinces of Vietnam (DM-S)	Subnational	both	25+	25+	830	1,446	Digital	
1473	Viet Nam	2009	STEPS	National	both	25-64	25-64	6,740	7,807	Digital	
1474	Zambia	1999	CD4 Survey; Kelly et al., Acta Trop 2002; 83: 151-8	Community	urban	25-74	25-74	129	215	Unknown	
1475	Zambia	2008	STEPS	Community	urban	25+	25+	632	1,220	Digital	
1476	Zimbabwe	1985-1986	INTERSALT	Community	urban	20-59	20-59	100	95	Random-zero	
1477	Zimbabwe	1993	Matenga et al., S Afr Med J 1997; 87: 1371-3	Community	both	60+	60+	121	209	Unknown	
1478	Zimbabwe	1995	Mufunda et al., J Hum Hypertens 2000; 14: 65-73	Community	both	25-54	25-54	284	298	Unknown	
1479	Zimbabwe	2005	STEPS	National	both	25+	25+	574	1,829	Digital	

1. Blood pressure measurement devices include:
   'Standard': standard mercury sphygmomanometer or aneroid sphygmomanometer; 'Random-zero': random-zero mercury sphygmomanometer; 'Digital': digital oscillometric device; 'Unknown': information unavailable or multiple types of devices used.
2. The first age group started from <18 years old, but had a mean age ≥18 years.
3. This research uses data from China Health and Nutrition Survey (CHNS). We thank the National Institute of Nutrition and Food Safety, China Center for Disease Control and Prevention, Carolina Population Center (5 R24 HD050924), the University of North Carolina at Chapel Hill, the NIH (R01-HD30880, DK056350, R24-HD050924, and R01-HD38700) and the Fogarty International Center, NIH for financial support for the CHNS data collection and analysis files from 1989 to 2011 and future surveys, and the China-Japan Friendship Hospital, Ministry of Health for support for CHNS 2009.
4. National study conducted between 1972 and 1974, included in the analysis as a 1975 study.

Appendix Table 3: Model specifications and regression coefficients to estimate the primary outcomes (mean SBP, mean DBP, and prevalence of raised blood pressure defined by SBP ≥140 mmHg or DBP ≥90 mmHg) from other metrics.

The dependent variable in all regressions was inverse of means or prevalence, fitted using a linear (mixed) model for means and a generalized linear (mixed) model with a probit link function for prevalence. Random effects for regions are presented.

* denotes statistical interaction. CI: confidence interval.

Primary outcome: mean SBP	
**Independent variable: prevalence (SBP ≥140 mmHg or DBP ≥90 mmHg)**	
**Variables**	**Coefficients (95% CI)**
Intercept	0.0074 (0.0074, 0.0075)
Probit-transformed prevalence (SBP ≥140 mmHg or DBP ≥90 mmHg)	-0.00095 (-0.00096, -0.00094)
Mean age of age group (centred at 50 years)	-6.2e-06 (-6.6e-06, -5.9e-06)
Male sex	9.4e-06 (-3.1e-08, 1.9e-05)
Study mid-year (per one more recent year since 1975)	-1.8e-06 (-2.3e-06, -1.4e-06)
Natural logarithm of per-capita gross domestic product	-5.8e-06 (-1.3e-05, 1.6e-06)
Probit-transformed prevalence * mean age of age group	-5.5e-06 (-5.8e-06, -5.2e-06)
Probit-transformed prevalence * male sex	0.00016 (0.00015, 0.00017)
Random effects for regions	Yes
**Number of data points used to fit the model = 10,517**	

Traditional R^2 is not clearly defined for mixed-effect models. The conditional R^2 for the model, which describes the proportion of variance explained by both fixed and random factors,[11] was 0.924.

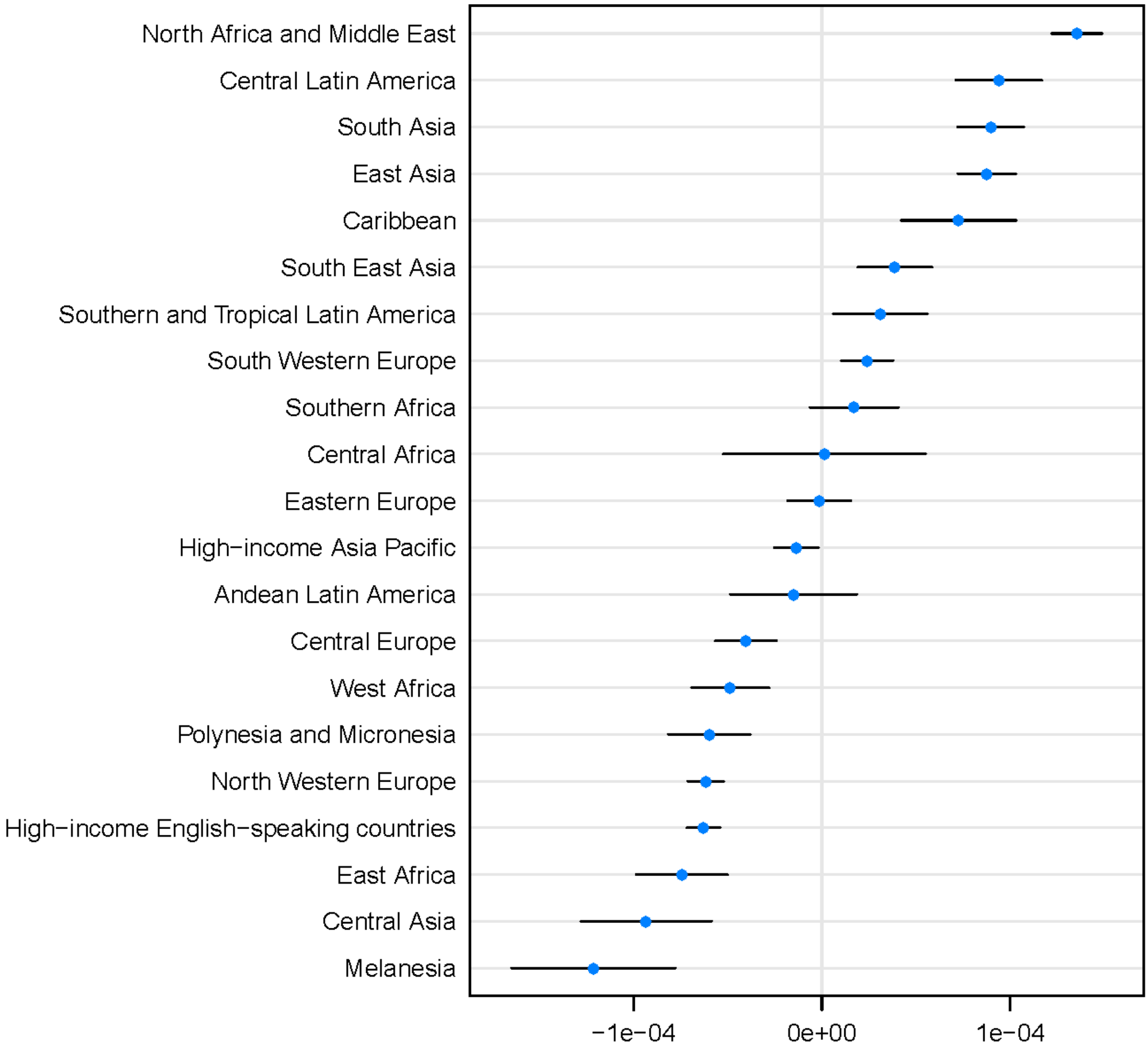
North Africa and Middle East
Central Latin America
South Asia
East Asia
Caribbean
South East Asia
Southern and Tropical Latin America
South Western Europe
Southern Africa
Central Africa
Eastern Europe
High−income Asia Pacific
Andean Latin America
Central Europe
West Africa
Polynesia and Micronesia
North Western Europe
High−income English−speaking countries
East Africa
Central Asia
Melanesia
−1e−04
0e+00
1e−04

Primary outcome: mean SBP	
**Independent variable: prevalence (SBP ≥140 mmHg)**	
**Variables**	**Coefficients (95% CI)**
Intercept	0.0073 (0.0072, 0.0074)
Probit-transformed prevalence (SBP ≥140 mmHg)	-0.00095 (-0.00096, -0.00094)
Mean age of age group (centred at 50 years)	-1.1e-06 (-1.5e-06, -8.0e-07)
Male sex	-2.3e-06 (-1.0e-05, 5.7e-06)
Study mid-year (per one more recent year since 1975)	-1.5e-06 (-1.8e-06, -1.1e-06)
Natural logarithm of per-capita gross domestic product	-1.2e-05 (-1.8e-05, -6.0e-06)
Probit-transformed prevalence * mean age of age group	-4.2e-06 (-4.4e-06, -3.9e-06)
Probit-transformed prevalence * male sex	0.00011 (0.00010, 0.00012)
Random effects for regions	Yes
**Number of data points used to fit the model = 10,558**	

Traditional R^2 is not clearly defined for mixed-effect models. The conditional R^2 for the model, which describes the proportion of variance explained by both fixed and random factors,[11] was 0.953.

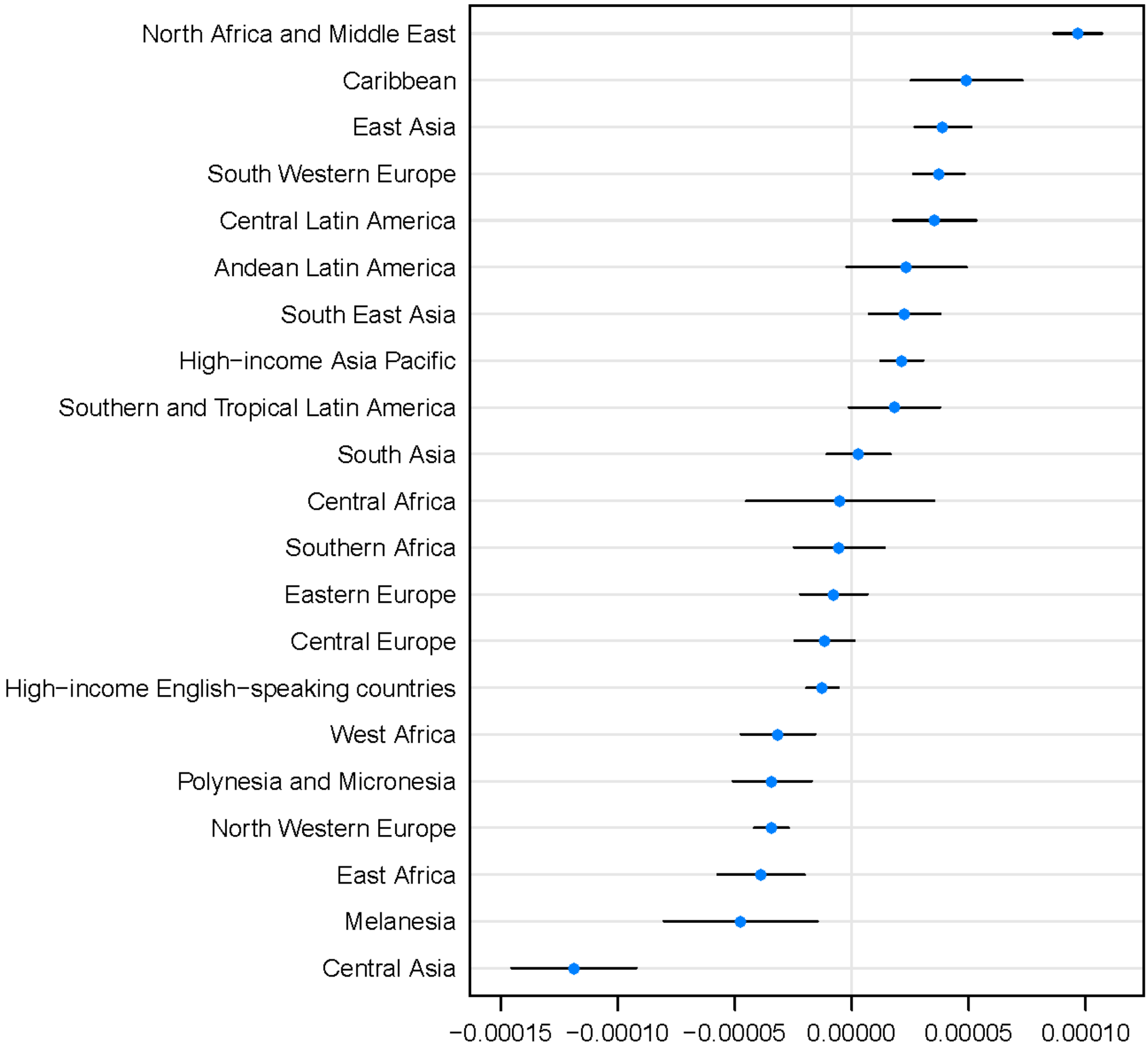
North Africa and Middle East
Caribbean
East Asia
South Western Europe
Central Latin America
Andean Latin America
South East Asia
High−income Asia Pacific
Southern and Tropical Latin America
South Asia
Central Africa
Southern Africa
Eastern Europe
Central Europe
High−income English−speaking countries
West Africa
Polynesia and Micronesia
North Western Europe
East Africa
Melanesia
Central Asia
−0.00015
−0.00010
−0.00005
0.00000
0.00005
0.00010

**Primary outcome: mean SBP**	
**Independent variable: prevalence (SBP ≥140 mmHg or DBP ≥90 mmHg or use of antihypertensive medication)**	
**Variables**	**Coefficients (95% CI)**
Intercept	0.0072 (0.0071, 0.0073)
Probit-transformed prevalence (SBP ≥140 mmHg or DBP ≥90 mmHg or use of antihypertensive medication)	-0.00086 (-0.00087, -0.00084)
Mean age of age group (centred at 50 years)	-1.5e-06 (-2.1e-06, -8.5e-07)
Male sex	-7.5e-05 (-8.7e-05, -6.4e-05)
Study mid-year (per one more recent year since 1975)	3.7e-06 (3.0e-06, 4.3e-06)
Natural logarithm of per-capita gross domestic product	2.6e-05 (1.6e-05, 3.6e-05)
Probit-transformed prevalence * mean age of age group	-2.4e-06 (-2.8e-06, -2.0e-06)
Probit-transformed prevalence * male sex	0.00014 (0.00013, 0.00016)
Random effects for regions	Yes
**Number of data points used to fit the model = 9,141**	

Traditional R^2 is not clearly defined for mixed-effect models. The conditional R^2 for the model, which describes the proportion of variance explained by both fixed and random factors,[11] was 0.873.

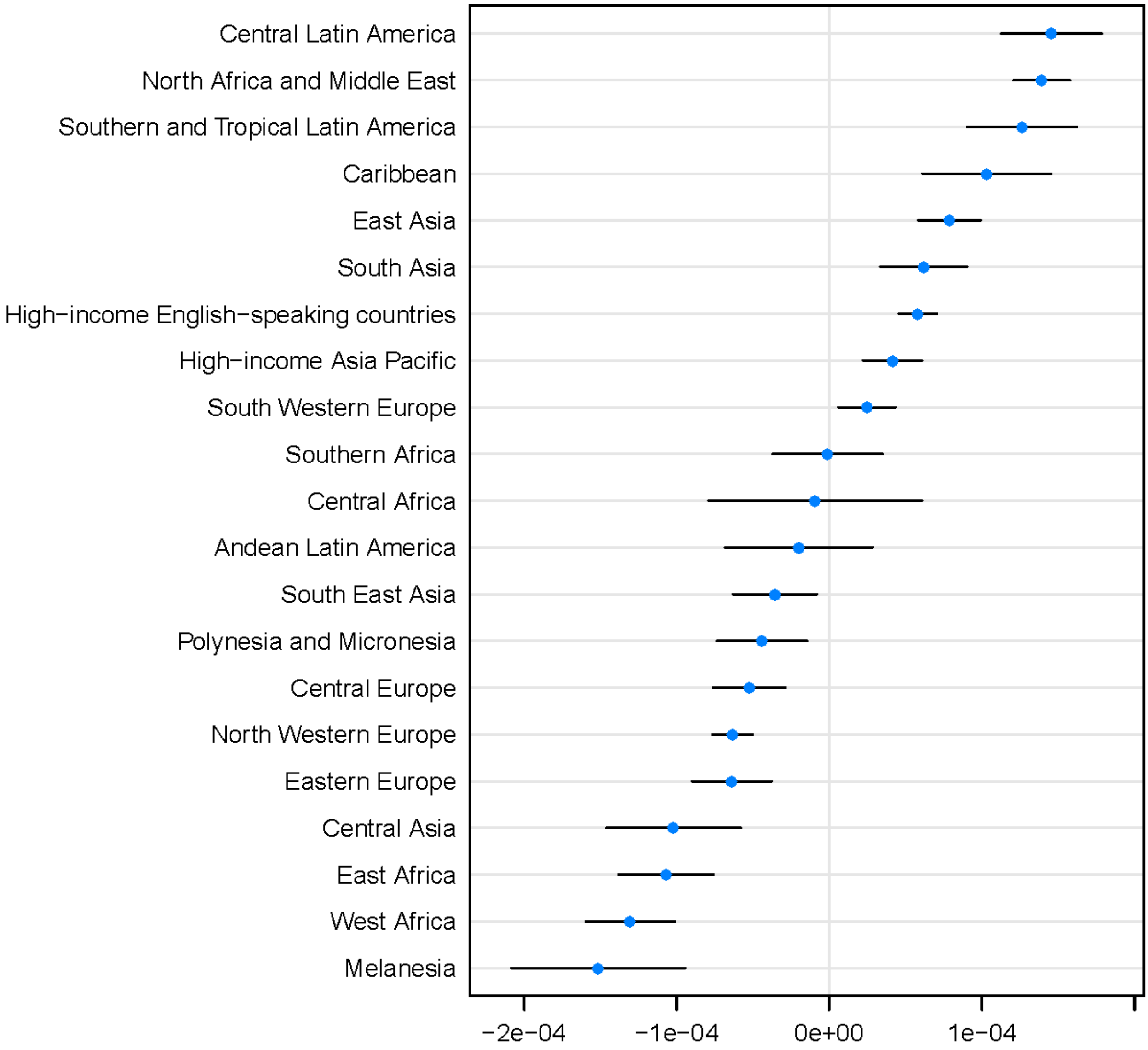
Central Latin America
North Africa and Middle East
Southern and Tropical Latin America
Caribbean
East Asia
South Asia
High−income English−speaking countries
High−income Asia Pacific
South Western Europe
Southern Africa
Central Africa
Andean Latin America
South East Asia
Polynesia and Micronesia
Central Europe
North Western Europe
Eastern Europe
Central Asia
East Africa
West Africa
Melanesia
−2e−04
−1e−04
0e+00
1e−04

Primary outcome: mean SBP	
**Independent variable: prevalence (SBP ≥160 mmHg or DBP ≥95 mmHg or use of antihypertensive medication)**	
**Variables**	**Coefficients (95% CI)**
Intercept	0.0070 (0.0068, 0.0071)
Probit-transformed prevalence (SBP ≥160 mmHg or DBP ≥95 mmHg or use of antihypertensive medication)	-0.00066 (-0.00068, -0.00064)
Mean age of age group (centred at 50 years)	-1.1e-05 (-1.2e-05, -1.0e-05)
Male sex	-2.2e-05 (-4.2e-05, -2.8e-06)
Study mid-year (per one more recent year since 1975)	7.7e-06 (6.9e-06, 8.6e-06)
Natural logarithm of per-capita gross domestic product	1.9e-05 (6.2e-06, 3.2e-05)
Probit-transformed prevalence * mean age of age group	-1.1e-06 (-1.6e-06, -5.6e-07)
Probit-transformed prevalence * male sex	0.00024 (0.00022, 0.00026)
Random effects for regions	Yes
**Number of data points used to fit the model = 8,852**	

Traditional R^2 is not clearly defined for mixed-effect models. The conditional R^2 for the model, which describes the proportion of variance explained by both fixed and random factors,[11] was 0.799.

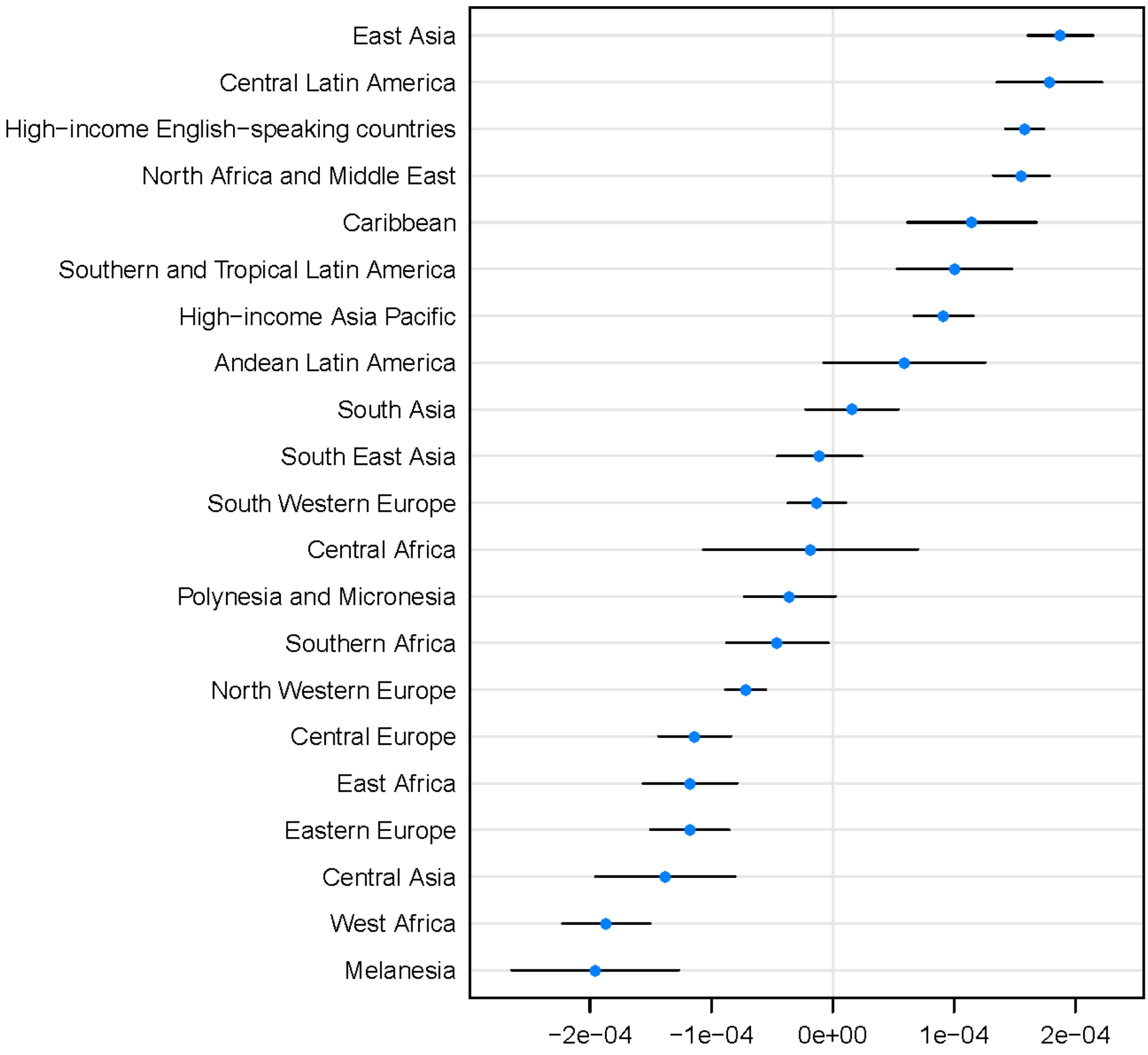
East Asia
Central Latin America
High−income English−speaking countries
North Africa and Middle East
Caribbean
Southern and Tropical Latin America
High−income Asia Pacific
Andean Latin America
South Asia
South East Asia
South Western Europe
Central Africa
Polynesia and Micronesia
Southern Africa
North Western Europe
Central Europe
East Africa
Eastern Europe
Central Asia
West Africa
Melanesia
−2e−04
−1e−04
0e+00
1e−04
2e−04

**Primary outcome: mean SBP**	
**Independent variable: prevalence (SBP ≥130 mmHg or DBP ≥85 mmHg)**	
**Variables**	**Coefficients (95% CI)**
Intercept	0.0078 (0.0077, 0.0078)
Probit-transformed prevalence (SBP ≥130 mmHg or DBP ≥85 mmHg)	-0.00094 (-0.00095, -0.00093)
Mean age of age group (centred at 50 years)	-4.5e-06 (-4.8e-06, -4.1e-06)
Male sex	-2.2e-05 (-3.0e-05, -1.4e-05)
Study mid-year (per one more recent year since 1975)	-1.1e-06 (-1.5e-06, -6.0e-07)
Natural logarithm of per-capita gross domestic product	3.8e-06 (-3.6e-06, 1.1e-05)
Probit-transformed prevalence * mean age of age group	-3.8e-06 (-4.2e-06, -3.5e-06)
Probit-transformed prevalence * male sex	0.00012 (0.00010, 0.00013)
Random effects for regions	Yes
**Number of data points used to fit the model = 7,558**	

Traditional R^2 is not clearly defined for mixed-effect models. The conditional R^2 for the model, which describes the proportion of variance explained by both fixed and random factors,[11] was 0.948.

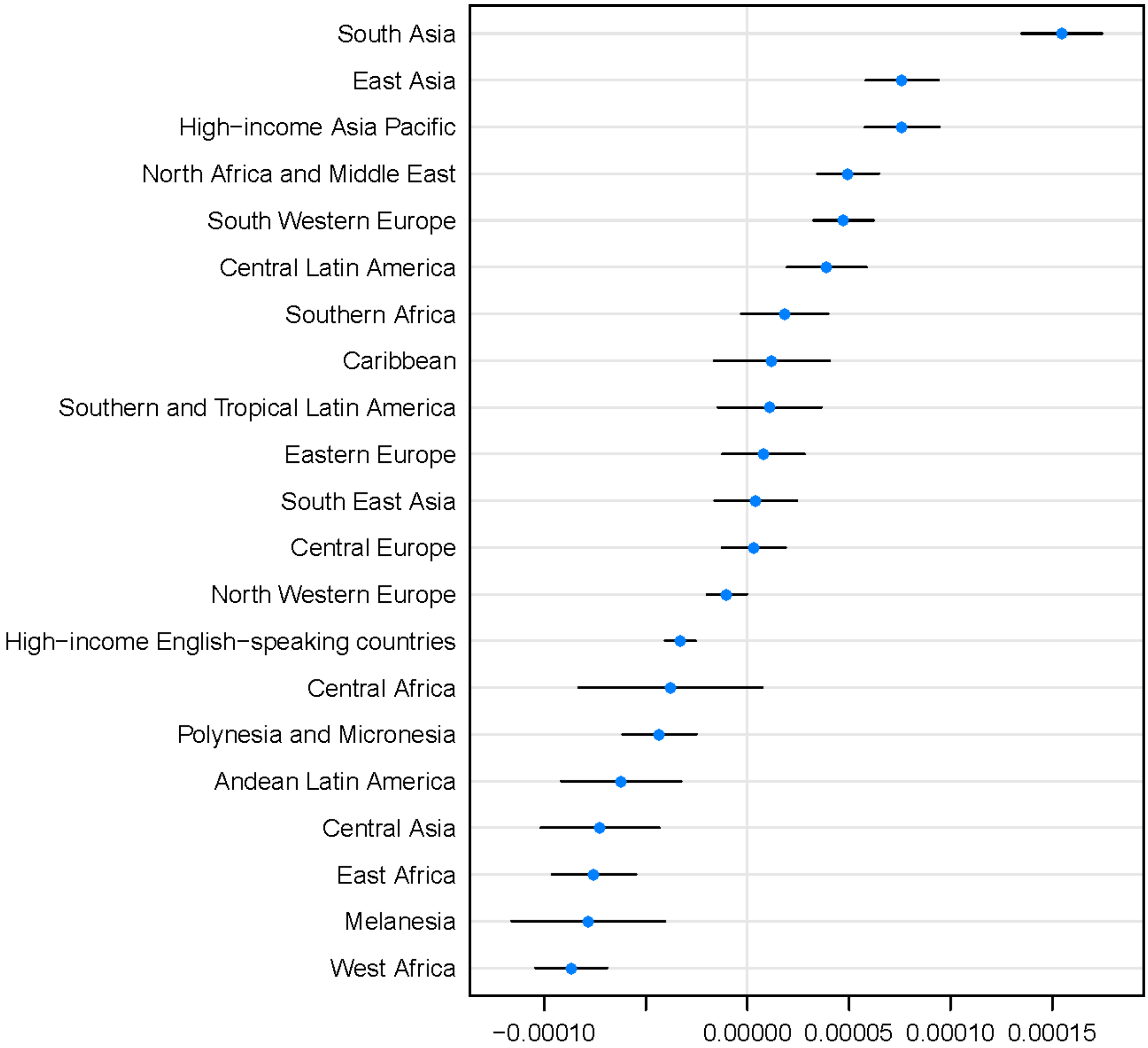
South Asia
East Asia
High−income Asia Pacific
North Africa and Middle East
South Western Europe
Central Latin America
Southern Africa
Caribbean
Southern and Tropical Latin America
Eastern Europe
South East Asia
Central Europe
North Western Europe
High−income English−speaking countries
Central Africa
Polynesia and Micronesia
Andean Latin America
Central Asia
East Africa
Melanesia
West Africa
−0.00010
0.00000
0.00005
0.00010
0.00015

Primary outcome: mean SBP	
**Independent variable: prevalence (SBP ≥150 mmHg or DBP ≥90 mmHg)**	
**Variables**	**Coefficients (95% CI)**
Intercept	0.0073 (0.0072, 0.0074)
Probit-transformed prevalence (SBP ≥150 mmHg or DBP ≥90 mmHg)	-0.00091 (-0.00093, -0.00090)
Mean age of age group (centred at 50 years)	-1.6e-05 (-1.7e-05, -1.5e-05)
Male sex	8.8e-05 (7.1e-05, 0.00011)
Study mid-year (per one more recent year since 1975)	-3.1e-06 (-3.8e-06, -2.4e-06)
Natural logarithm of per-capita gross domestic product	-7.3e-07 (-1.2e-05, 1.0e-05)
Probit-transformed prevalence * mean age of age group	-7.7e-06 (-8.2e-06, -7.2e-06)
Probit-transformed prevalence * male sex	0.00027 (0.00025, 0.00029)
Random effects for regions	Yes
**Number of data points used to fit the model = 7,412**	

Traditional R^2 is not clearly defined for mixed-effect models. The conditional R^2 for the model, which describes the proportion of variance explained by both fixed and random factors,[11] was 0.884.

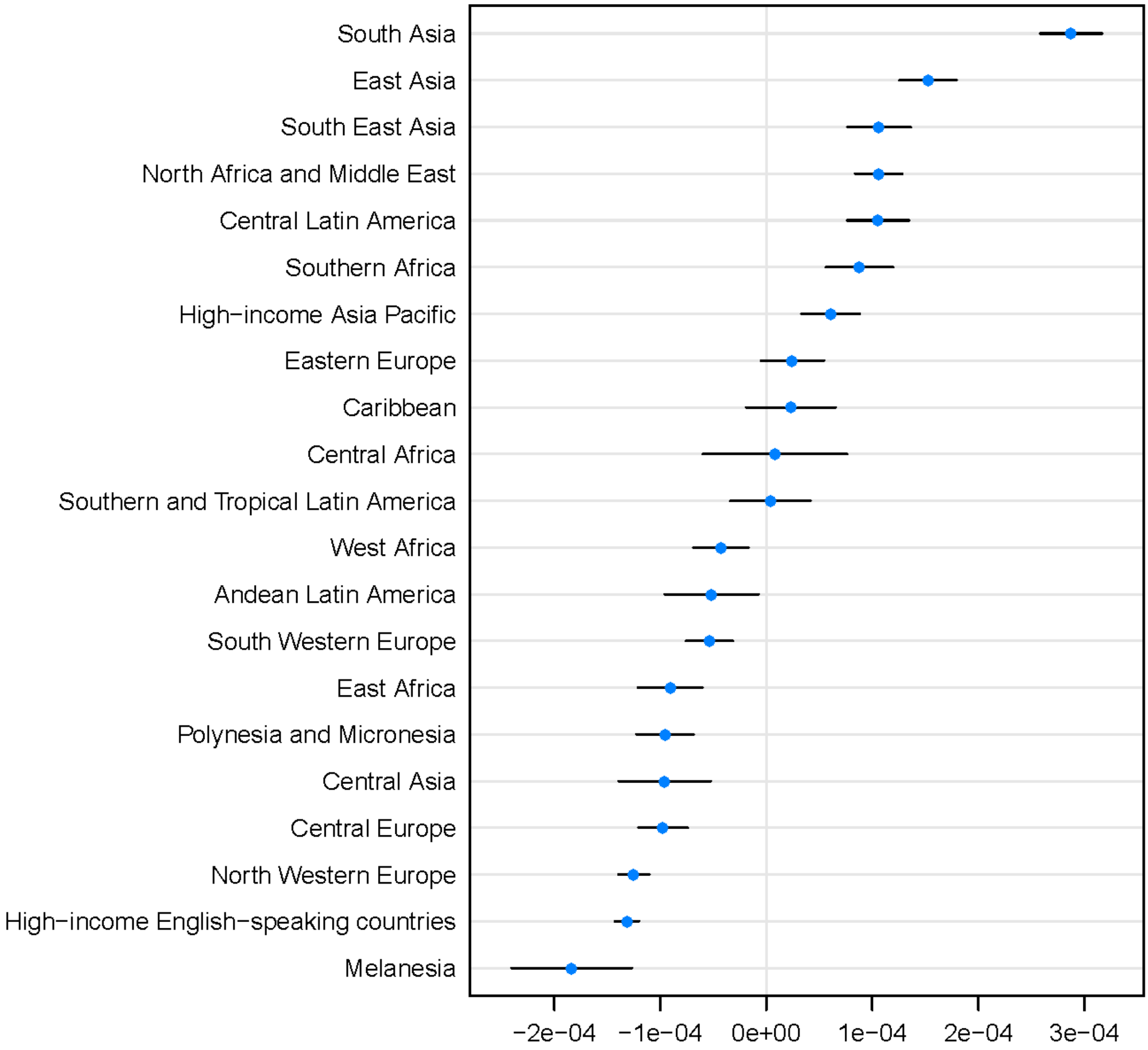
South Asia
East Asia
South East Asia
North Africa and Middle East
Central Latin America
Southern Africa
High−income Asia Pacific
Eastern Europe
Caribbean
Central Africa
Southern and Tropical Latin America
West Africa
Andean Latin America
South Western Europe
East Africa
Polynesia and Micronesia
Central Asia
Central Europe
North Western Europe
High−income English−speaking countries
Melanesia
−2e−04
−1e−04
0e+00
1e−04
2e−04
3e−04

Primary outcome: mean SBP	
**Independent variable: prevalence (SBP ≥160 mmHg or DBP ≥90 mmHg)**	
**Variables**	**Coefficients (95% CI)**
Intercept	0.0073 (0.0072, 0.0074)
Probit-transformed prevalence (SBP ≥160 mmHg or DBP ≥90 mmHg)	-0.00084 (-0.00086, -0.00082)
Mean age of age group (centred at 50 years)	-2.4e-05 (-2.5e-05, -2.3e-05)
Male sex	0.00015 (0.00013, 0.00017)
Study mid-year (per one more recent year since 1975)	-2.6e-06 (-3.4e-06, -1.8e-06)
Natural logarithm of per-capita gross domestic product	-4.9e-06 (-1.7e-05, 7.3e-06)
Probit-transformed prevalence * mean age of age group	-8.6e-06 (-9.2e-06, -7.9e-06)
Probit-transformed prevalence * male sex	0.00032 (0.00030, 0.00034)
Random effects for regions	Yes
**Number of data points used to fit the model = 7,389**	

Traditional R^2 is not clearly defined for mixed-effect models. The conditional R^2 for the model, which describes the proportion of variance explained by both fixed and random factors,[11] was 0.856.

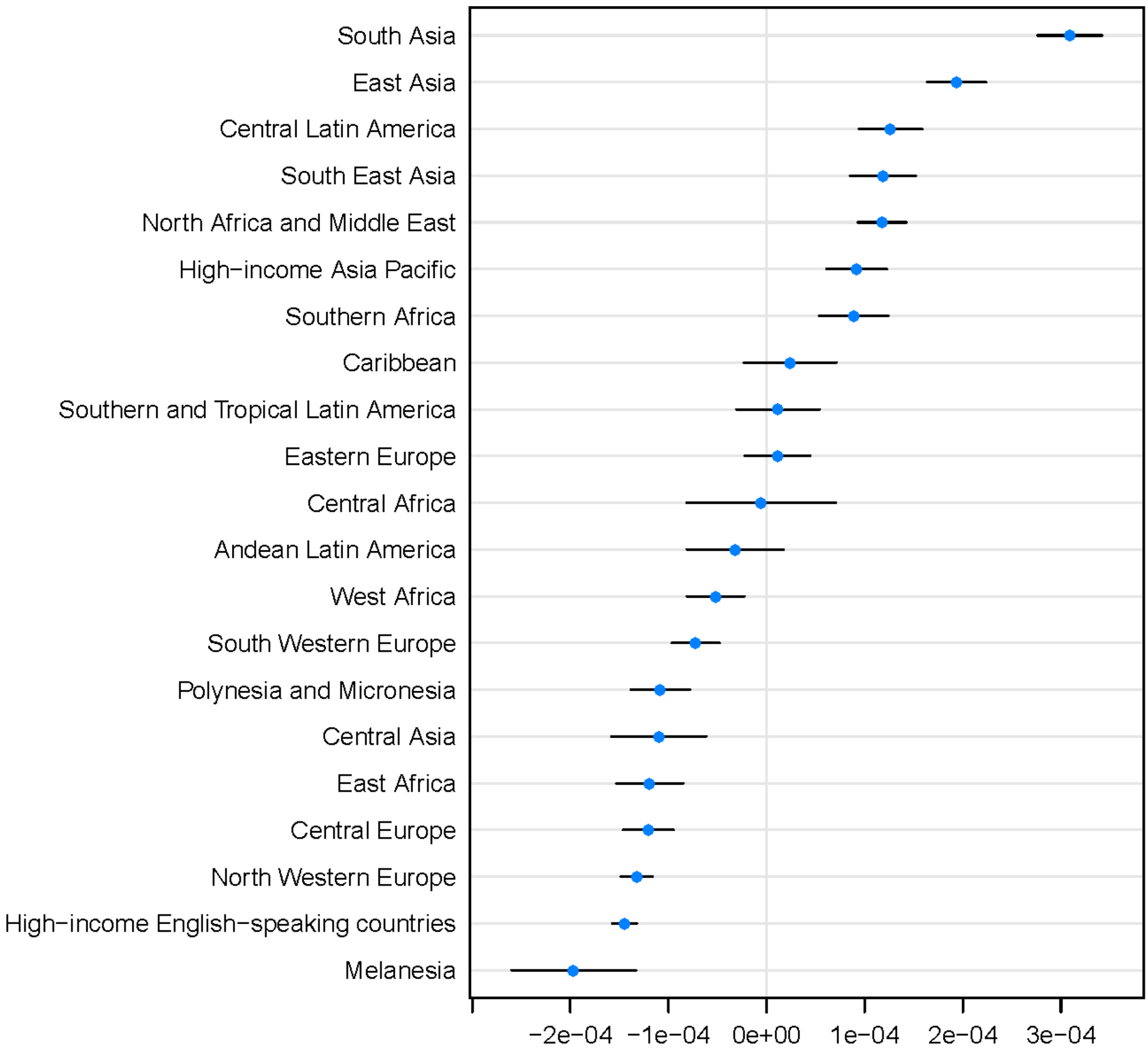
South Asia
East Asia
Central Latin America
South East Asia
North Africa and Middle East
High−income Asia Pacific
Southern Africa
Caribbean
Southern and Tropical Latin America
Eastern Europe
Central Africa
Andean Latin America
West Africa
South Western Europe
Polynesia and Micronesia
Central Asia
East Africa
Central Europe
North Western Europe
High−income English−speaking countries
Melanesia
−2e−04
−1e−04
0e+00
1e−04
2e−04
3e−04

Primary outcome: mean SBP	
**Independent variable: prevalence (SBP ≥160 mmHg or DBP ≥95 mmHg)**	
**Variables**	**Coefficients (95% CI)**
Intercept	0.0069 (0.0068, 0.0070)
Probit-transformed prevalence (SBP ≥160 mmHg or DBP ≥95 mmHg)	-0.00092 (-0.00093, -0.00090)
Mean age of age group (centred at 50 years)	-1.9e-05 (-2.0e-05, -1.8e-05)
Male sex	0.00019 (0.00017, 0.00022)
Study mid-year (per one more recent year since 1975)	-2.0e-06 (-2.7e-06, -1.3e-06)
Natural logarithm of per-capita gross domestic product	5.3e-07 (-1.0e-05, 1.1e-05)
Probit-transformed prevalence * mean age of age group	-7.5e-06 (-8.0e-06, -6.9e-06)
Probit-transformed prevalence * male sex	0.00028 (0.00026, 0.00030)
Random effects for regions	Yes
**Number of data points used to fit the model = 7,477**	

Traditional R^2 is not clearly defined for mixed-effect models. The conditional R^2 for the model, which describes the proportion of variance explained by both fixed and random factors,[11] was 0.876.

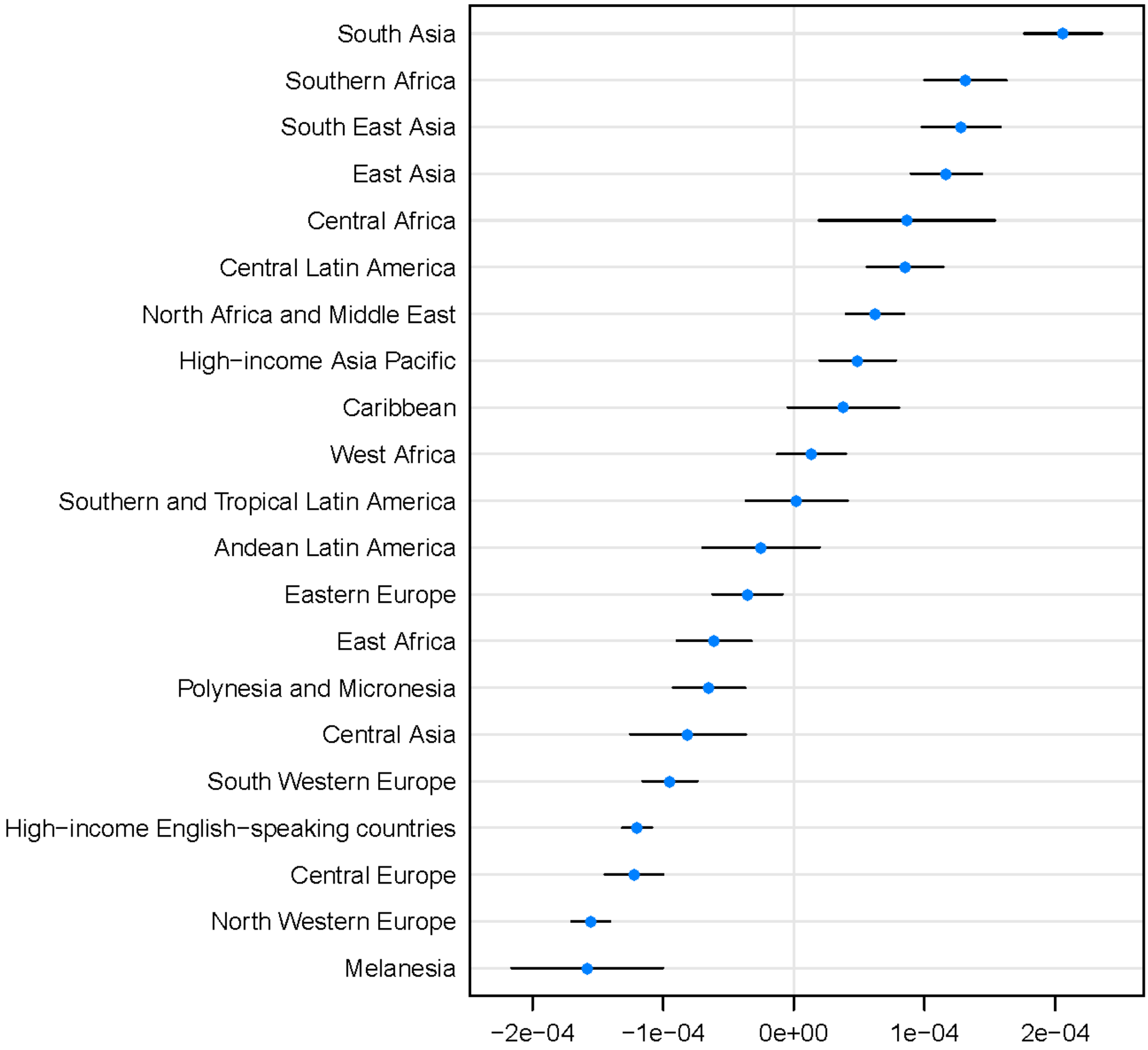
South Asia
Southern Africa
South East Asia
East Asia
Central Africa
Central Latin America
North Africa and Middle East
High−income Asia Pacific
Caribbean
West Africa
Southern and Tropical Latin America
Andean Latin America
Eastern Europe
East Africa
Polynesia and Micronesia
Central Asia
South Western Europe
High−income English−speaking countries
Central Europe
North Western Europe
Melanesia
−2e−04
−1e−04
0e+00
1e−04
2e−04

**Primary outcome: mean SBP**	
**Independent variable: prevalence (SBP ≥130 mmHg or DBP ≥85 mmHg or use of antihypertensive medication)**	
**Variables**	**Coefficients (95% CI)**
Intercept	0.0076 (0.0075, 0.0076)
Probit-transformed prevalence (SBP ≥130 mmHg or DBP ≥85 mmHg or use of antihypertensive medication)	-0.00090 (-0.00091, -0.00088)
Mean age of age group (centred at 50 years)	-5.7e-07 (-1.1e-06, -2.5e-09)
Male sex	-6.0e-05 (-7.1e-05, -4.9e-05)
Study mid-year (per one more recent year since 1975)	2.2e-06 (1.5e-06, 2.8e-06)
Natural logarithm of per-capita gross domestic product	2.9e-05 (1.9e-05, 3.9e-05)
Probit-transformed prevalence * mean age of age group	-1.6e-06 (-2.1e-06, -1.2e-06)
Probit-transformed prevalence * male sex	9.7e-05 (8.2e-05, 0.00011)
Random effects for regions	Yes
**Number of data points used to fit the model = 6,346**	

Traditional R^2 is not clearly defined for mixed-effect models. The conditional R^2 for the model, which describes the proportion of variance explained by both fixed and random factors,[11] was 0.912.

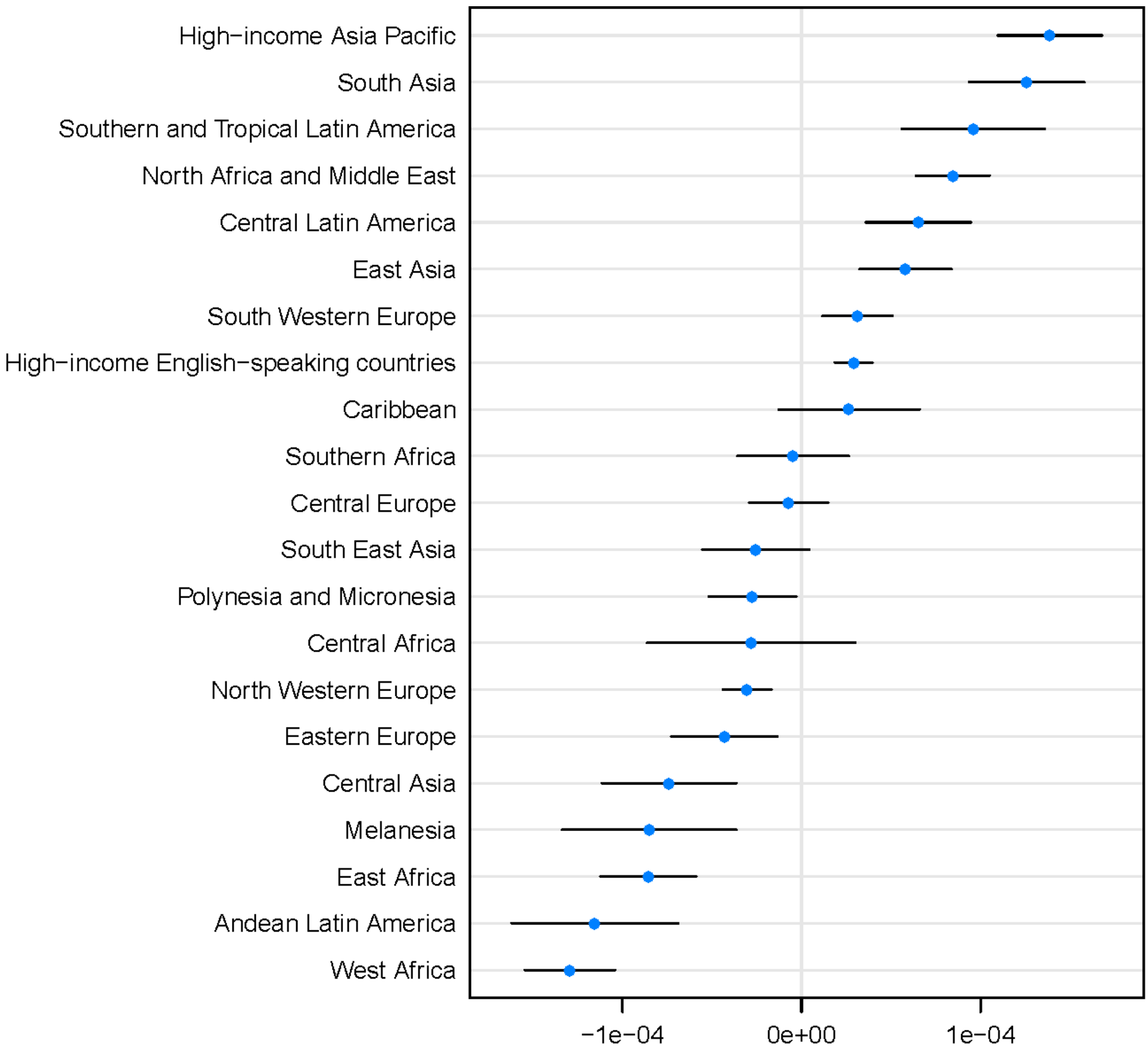

High−income Asia Pacific
South Asia
Southern and Tropical Latin America
North Africa and Middle East
Central Latin America
East Asia
South Western Europe
High−income English−speaking countries
Caribbean
Southern Africa
Central Europe
South East Asia
Polynesia and Micronesia
Central Africa
North Western Europe
Eastern Europe
Central Asia
Melanesia
East Africa
Andean Latin America
West Africa
−1e−04
0e+00
1e−04

**Primary outcome: mean SBP**	
**Independent variable: prevalence (SBP ≥160 mmHg or DBP ≥100 mmHg or use of antihypertensive medication)**	
**Variables**	**Coefficients (95% CI)**
Intercept	0.0068 (0.0066, 0.0070)
Probit-transformed prevalence (SBP ≥160 mmHg or DBP ≥100 mmHg or use of antihypertensive medication)	-0.00061 (-0.00064, -0.00059)
Mean age of age group (centred at 50 years)	-1.2e-05 (-1.3e-05, -1.0e-05)
Male sex	-1.3e-05 (-3.8e-05, 1.3e-05)
Study mid-year (per one more recent year since 1975)	5.2e-06 (4.1e-06, 6.2e-06)
Natural logarithm of per-capita gross domestic product	4.2e-05 (2.6e-05, 5.8e-05)
Probit-transformed prevalence * mean age of age group	-5.9e-07 (-1.2e-06, 3.7e-08)
Probit-transformed prevalence * male sex	0.00026 (0.00024, 0.00028)
Random effects for regions	Yes
**Number of data points used to fit the model = 6,023**	

Traditional R^2 is not clearly defined for mixed-effect models. The conditional R^2 for the model, which describes the proportion of variance explained by both fixed and random factors,[11] was 0.779.

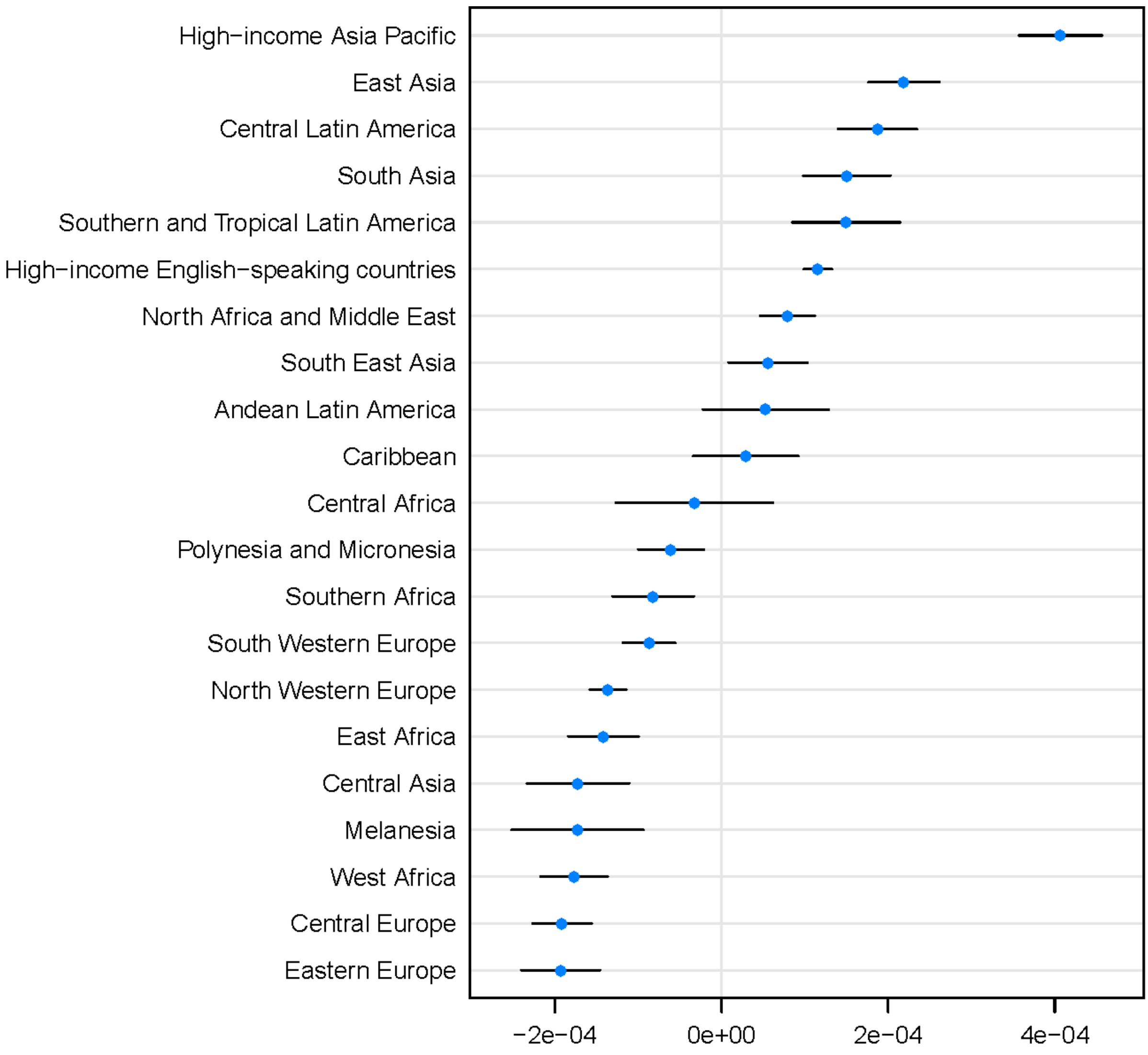
High−income Asia Pacific
East Asia
Central Latin America
South Asia
Southern and Tropical Latin America
High−income English−speaking countries
North Africa and Middle East
South East Asia
Andean Latin America
Caribbean
Central Africa
Polynesia and Micronesia
Southern Africa
South Western Europe
North Western Europe
East Africa
Central Asia
Melanesia
West Africa
Central Europe
Eastern Europe
−2e−04
0e+00
2e−04
4e−04

Primary outcome: mean SBP	
**Independent variables: prevalence (SBP ≥140 mmHg or DBP ≥90 mmHg or use of antihypertensive medication) and prevalence (SBP ≥160 mmHg or DBP ≥95 mmHg or use of antihypertensive medication)**	
**Variables**	**Coefficients (95% CI)**
Intercept	0.0076 (0.0075, 0.0077)
Probit-transformed prevalence (SBP ≥140 mmHg or DBP ≥90 mmHg or use of antihypertensive medication)	-0.0012 (-0.0013, -0.0012)
Probit-transformed prevalence (SBP ≥160 mmHg or DBP ≥95 mmHg or use of antihypertensive medication)	0.00042 (0.00038, 0.00046)
Mean age of age group (centred at 50 years)	3.0e-07 (-6.2e-07, 1.2e-06)
Male sex	-5.8e-05 (-8.4e-05, -3.1e-05)
Study mid-year (per one more recent year since 1975)	2.0e-06 (1.4e-06, 2.7e-06)
Natural logarithm of per-capita gross domestic product	1.1e-05 (1.3e-06, 2.1e-05)
Probit-transformed prevalence (SBP ≥140 mmHg or DBP ≥90 mmHg or use of antihypertensive medication) * mean age of age group	-7.7e-06 (-9.0e-06, -6.4e-06)
Probit-transformed prevalence (SBP ≥160 mmHg or DBP ≥95 mmHg or use of antihypertensive medication) * mean age of age group	6.8e-06 (5.6e-06, 8.0e-06)
Probit-transformed prevalence (SBP ≥140 mmHg or DBP ≥90 mmHg or use of antihypertensive medication) * male sex	0.00017 (0.00012, 0.00022)
Probit-transformed prevalence (SBP ≥160 mmHg or DBP ≥95 mmHg or use of antihypertensive medication) * male sex	-3.3e-05 (-8.3e-05, 1.7e-05)
Random effects for regions	Yes
**Number of data points used to fit the model = 8,724**	

Traditional R^2 is not clearly defined for mixed-effect models. The conditional R^2 for the model, which describes the proportion of variance explained by both fixed and random factors,[11] was 0.886.

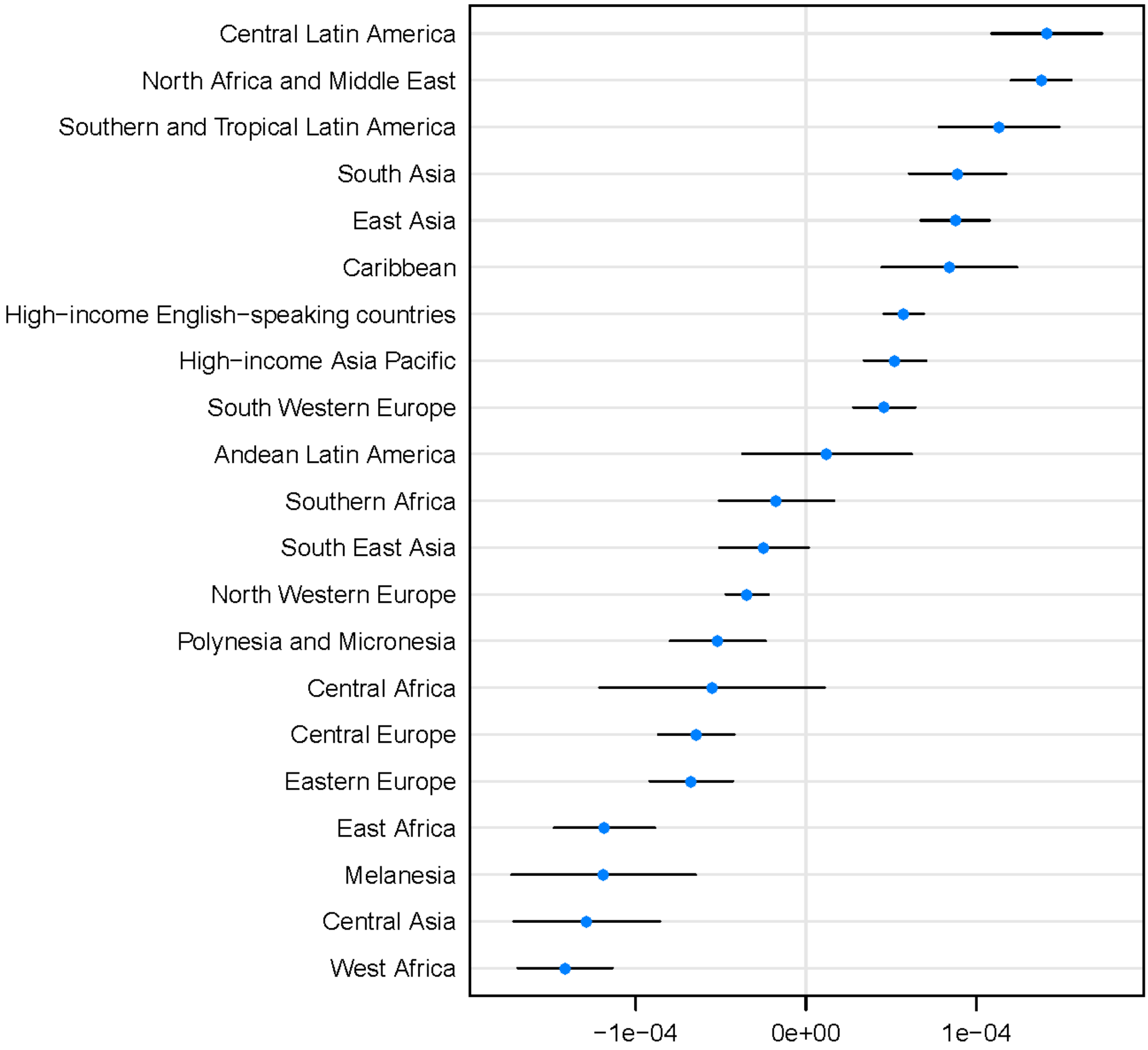

Central Latin America
North Africa and Middle East
Southern and Tropical Latin America
South Asia
East Asia
Caribbean
High−income English−speaking countries
High−income Asia Pacific
South Western Europe
Andean Latin America
Southern Africa
South East Asia
North Western Europe
Polynesia and Micronesia
Central Africa
Central Europe
Eastern Europe
East Africa
Melanesia
Central Asia
West Africa
−1e−04
0e+00
1e−04

Primary outcome: mean SBP	
**Independent variables: prevalence (SBP ≥160 mmHg) and prevalence (DBP ≥95 mmHg)**	
**Variables**	**Coefficients (95% CI)**
Intercept	0.0066 (0.0066, 0.0068)
Probit-transformed prevalence (SBP ≥160 mmHg)	-0.00088 (-0.00090, -0.00086)
Probit-transformed prevalence (DBP ≥95 mmHg)	-0.00010 (-0.00013, -8.2e-05)
Mean age of age group (centred at 50 years)	-1.0e-05 (-1.1e-05, -9.2e-06)
Male sex	0.00014 (0.00011, 0.00016)
Study mid-year (per one more recent year since 1975)	-2.8e-06 (-3.5e-06, -2.2e-06)
Natural logarithm of per-capita gross domestic product	-2.0e-05 (-2.9e-05, -1.0e-05)
Probit-transformed prevalence (SBP ≥160 mmHg) * mean age of age group	-5.6e-06 (-6.2e-06, -5.1e-06)
Probit-transformed prevalence (DBP ≥95 mmHg) * mean age of age group	1.0e-07 (-6.0e-07, 8.1e-07)
Probit-transformed prevalence (SBP ≥160 mmHg) * male sex	0.00023 (0.00021, 0.00024)
Probit-transformed prevalence (DBP ≥95 mmHg) * male sex	-3.2e-05 (-5.5e-05, -8.4e-06)
Random effects for regions	Yes
**Number of data points used to fit the model = 6,257**	

Traditional R^2 is not clearly defined for mixed-effect models. The conditional R^2 for the model, which describes the proportion of variance explained by both fixed and random factors,[11] was 0.905.

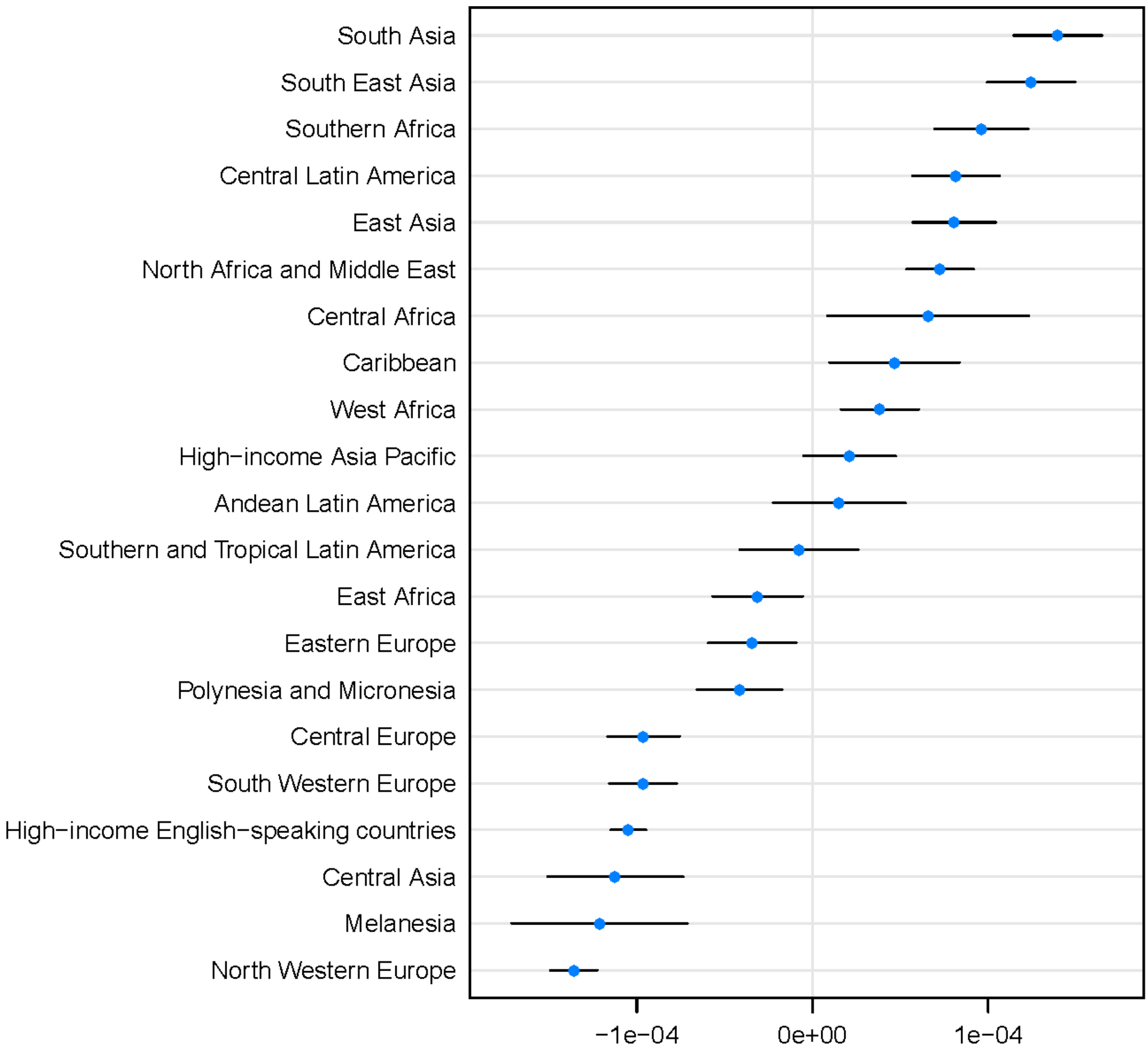
South Asia
South East Asia
Southern Africa
Central Latin America
East Asia
North Africa and Middle East
Central Africa
Caribbean
West Africa
High−income Asia Pacific
Andean Latin America
Southern and Tropical Latin America
East Africa
Eastern Europe
Polynesia and Micronesia
Central Europe
South Western Europe
High−income English−speaking countries
Central Asia
Melanesia
North Western Europe
−1e−04
0e+00
1e−04

Primary outcome: mean DBP	
**Independent variable: mean SBP**	
**Variables**	**Coefficients (95% CI)**
Intercept	0.012 (0.012, 0.012)
Mean SBP (centred at 130 mmHg)	-6.5e-05 (-6.7e-05, -6.3e-05)
Mean age of age group (centred at 50 years)	1.2e-05 (1.1e-05, 1.3e-05)
Male sex	-2.2e-05 (-4.5e-05, 7.3e-07)
Study mid-year (per one more recent year since 1975)	4.0e-06 (2.7e-06, 5.3e-06)
Natural logarithm of per-capita gross domestic product	2.0e-05 (-2.8e-06, 4.2e-05)
Mean SBP * mean age of age group	1.7e-06 (1.7e-06, 1.8e-06)
Mean SBP * male sex	-1.1e-05 (-1.3e-05, -9.2e-06)
Random effects for regions	Yes
**Number of data points used to fit the model = 11,979**	

Traditional R^2 is not clearly defined for mixed-effect models. The conditional R^2 for the model, which describes the proportion of variance explained by both fixed and random factors,[11] was 0.640.

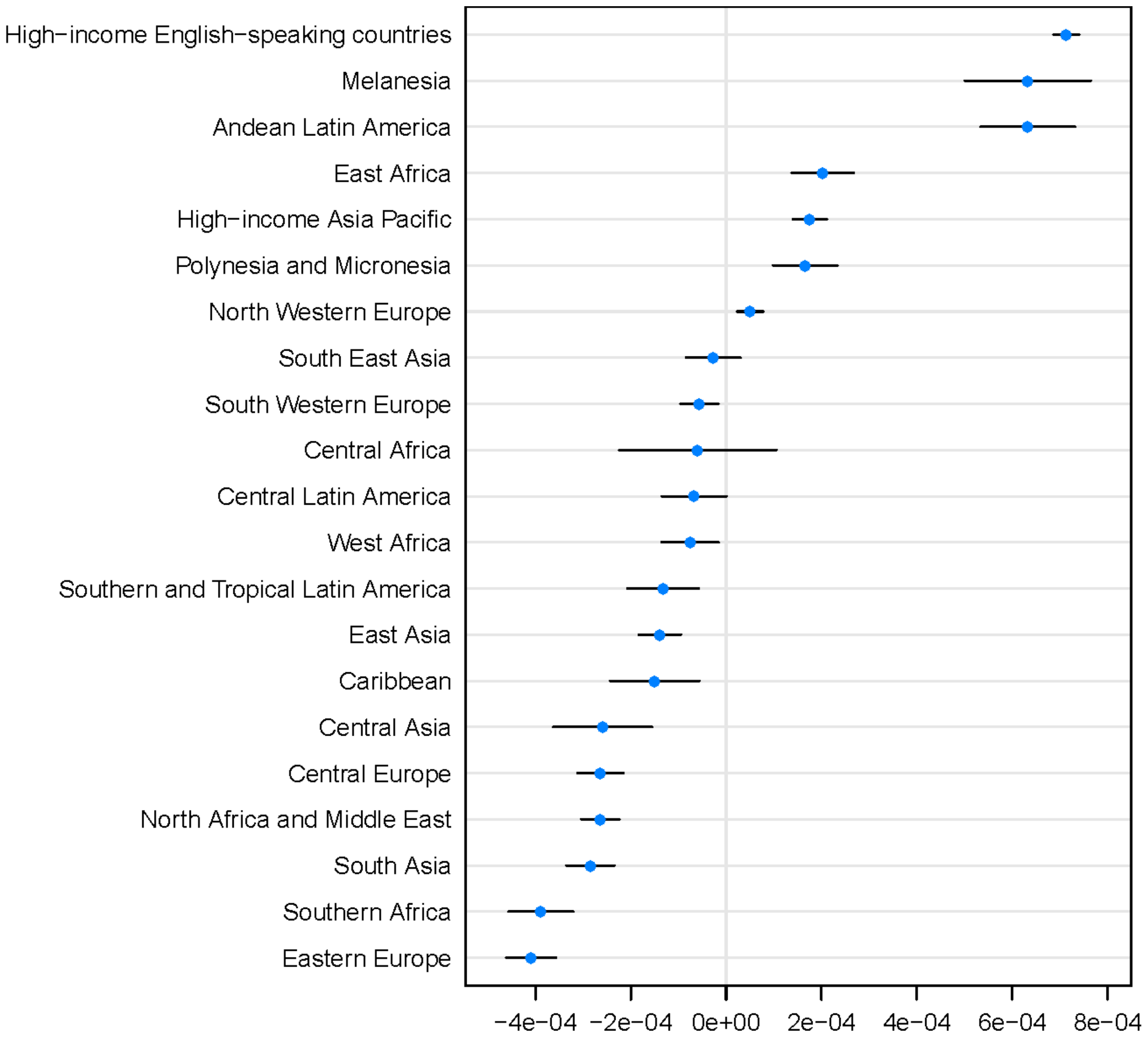
High−income English−speaking countries
Melanesia
Andean Latin America
East Africa
High−income Asia Pacific
Polynesia and Micronesia
North Western Europe
South East Asia
South Western Europe
Central Africa
Central Latin America
West Africa
Southern and Tropical Latin America
East Asia
Caribbean
Central Asia
Central Europe
North Africa and Middle East
South Asia
Southern Africa
Eastern Europe
−4e−04
−2e−04
0e+00
2e−04
4e−04
6e−04
8e−04

**Primary outcome: mean DBP**	
**Independent variable: prevalence (SBP ≥140 mmHg or DBP ≥90 mmHg)**	
**Variables**	**Coefficients (95% CI)**
Intercept	0.012 (0.012, 0.012)
Probit-transformed prevalence (SBP ≥140 mmHg or DBP ≥90 mmHg)	-0.0012 (-0.0013, -0.0012)
Mean age of age group (centred at 50 years)	3.0e-05 (2.9e-05, 3.2e-05)
Male sex	-0.00014 (-0.00016, -0.00011)
Study mid-year (per one more recent year since 1975)	9.1e-08 (-1.1e-06, 1.3e-06)
Natural logarithm of per-capita gross domestic product	1.6e-05 (-3.7e-06, 3.6e-05)
Probit-transformed prevalence * mean age of age group	1.7e-05 (1.6e-05, 1.8e-05)
Probit-transformed prevalence * male sex	-0.00021 (-0.00024, -0.00018)
Random effects for regions	Yes
**Number of data points used to fit the model = 10,965**	

Traditional R^2 is not clearly defined for mixed-effect models. The conditional R^2 for the model, which describes the proportion of variance explained by both fixed and random factors,[11] was 0.699.

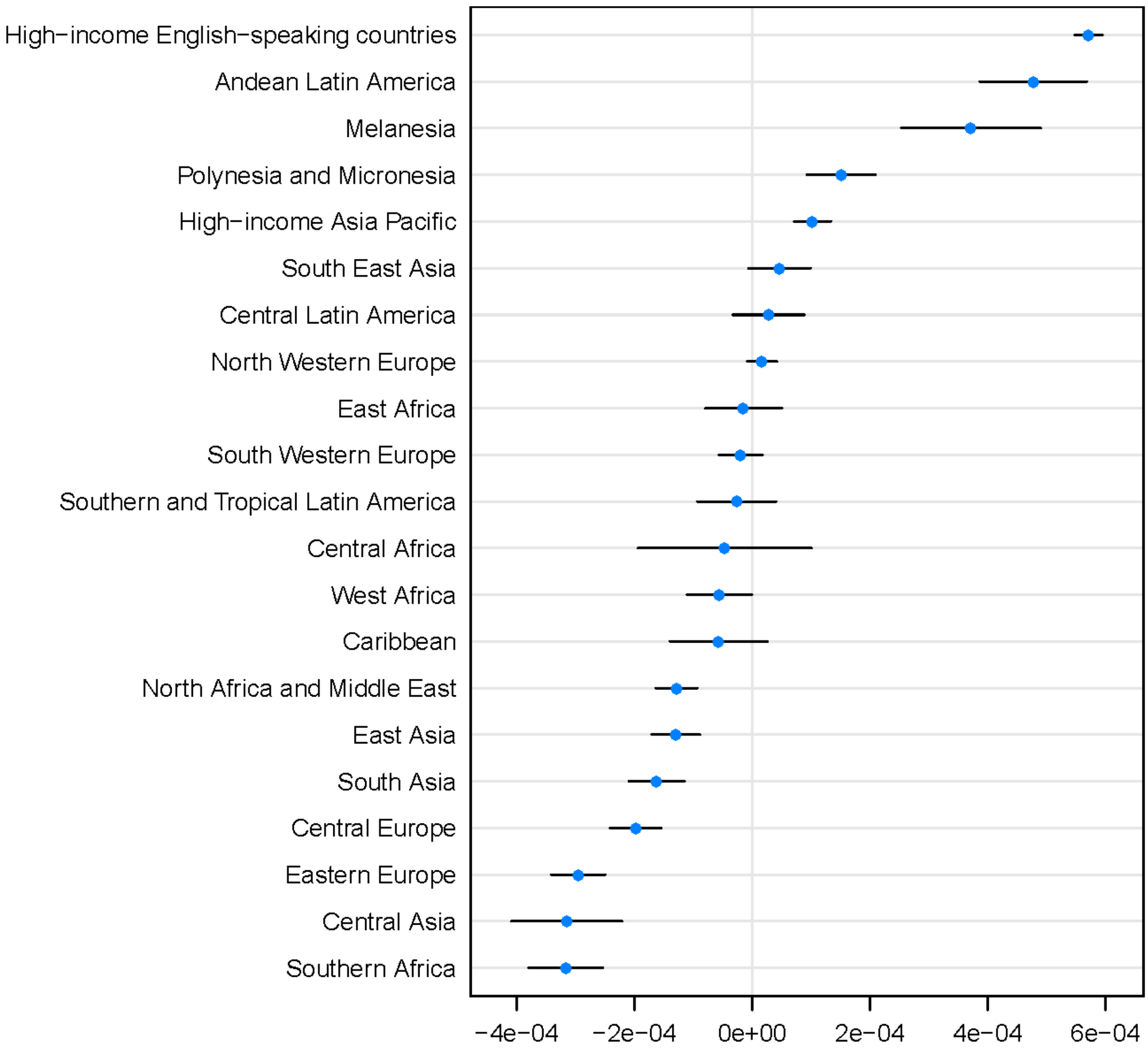
High−income English−speaking countries
Andean Latin America
Melanesia
Polynesia and Micronesia
High−income Asia Pacific
South East Asia
Central Latin America
North Western Europe
East Africa
South Western Europe
Southern and Tropical Latin America
Central Africa
West Africa
Caribbean
North Africa and Middle East
East Asia
South Asia
Central Europe
Eastern Europe
Central Asia
Southern Africa
−4e−04
−2e−04
0e+00
2e−04
4e−04
6e−04

**Primary outcome: mean DBP**	
**Independent variable: prevalence (SBP ≥140 mmHg or DBP ≥90 mmHg or use of antihypertensive medication)**	
**Variables**	**Coefficients (95% CI)**
Intercept	0.012 (0.011, 0.012)
Probit-transformed prevalence (SBP ≥140 mmHg or DBP ≥90 mmHg or use of antihypertensive medication)	-0.0011 (-0.0011, -0.0010)
Mean age of age group (centred at 50 years)	3.2e-05 (3.1e-05, 3.4e-05)
Male sex	-0.00018 (-0.00020, -0.00015)
Study mid-year (per one more recent year since 1975)	9.0e-06 (7.5e-06, 1.0e-05)
Natural logarithm of per-capita gross domestic product	3.4e-05 (1.2e-05, 5.6e-05)
Probit-transformed prevalence * mean age of age group	2.4e-05 (2.3e-05, 2.4e-05)
Probit-transformed prevalence * male sex	-0.00014 (-0.00017, -0.00011)
Random effects for regions	Yes
**Number of data points used to fit the model = 9,124**	

Traditional R^2 is not clearly defined for mixed-effect models. The conditional R^2 for the model, which describes the proportion of variance explained by both fixed and random factors,[11] was 0.672.

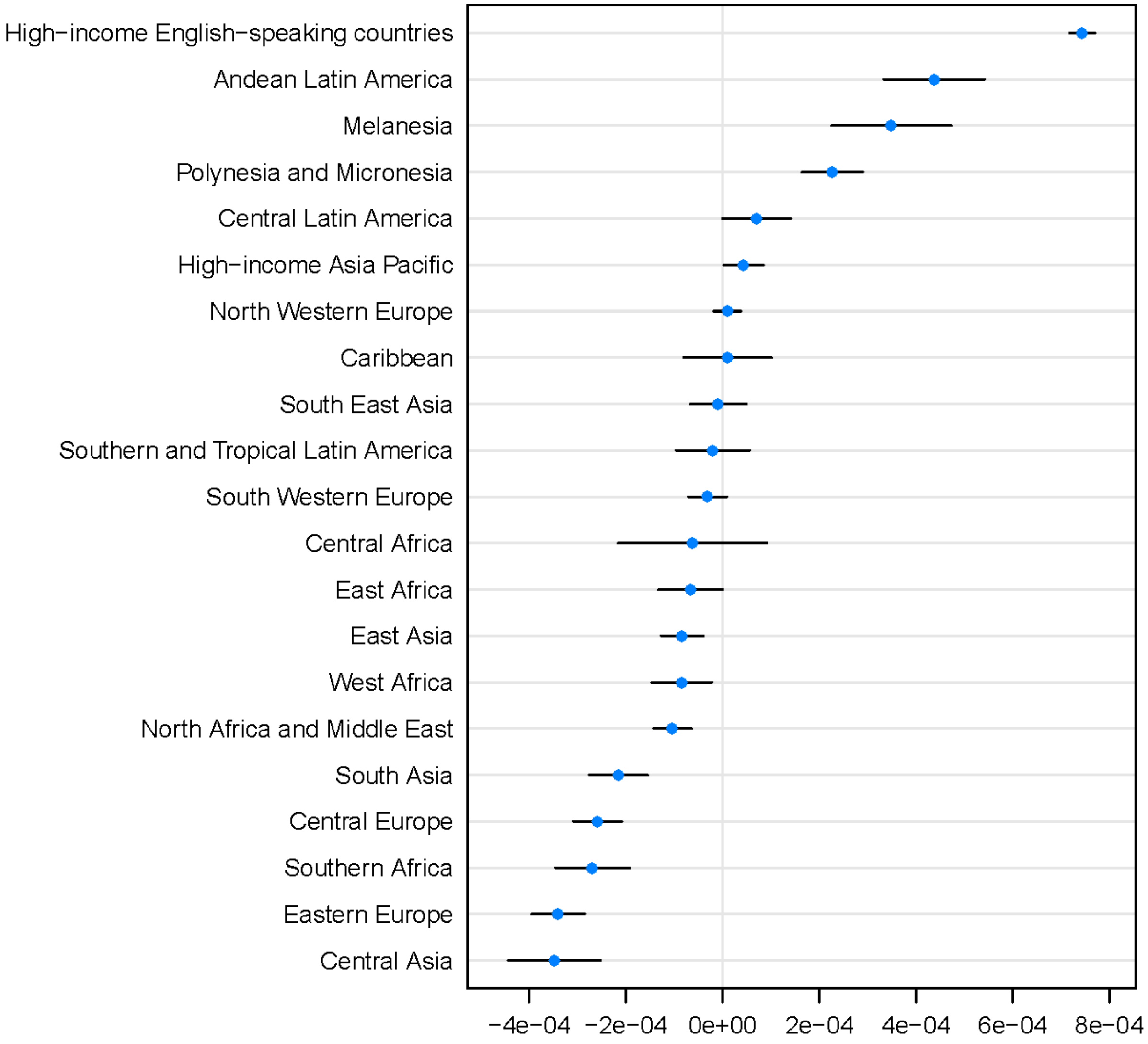
High−income English−speaking countries
Andean Latin America
Melanesia
Polynesia and Micronesia
Central Latin America
High−income Asia Pacific
North Western Europe
Caribbean
South East Asia
Southern and Tropical Latin America
South Western Europe
Central Africa
East Africa
East Asia
West Africa
North Africa and Middle East
South Asia
Central Europe
Southern Africa
Eastern Europe
Central Asia
−4e−04
−2e−04
0e+00
2e−04
4e−04
6e−04
8e−04

Primary outcome: mean DBP	
**Independent variable: prevalence (SBP ≥160 mmHg or DBP ≥95 mmHg or use of antihypertensive medication)**	
**Variables**	**Coefficients (95% CI)**
Intercept	0.011 (0.011, 0.011)
Probit-transformed prevalence (SBP ≥160 mmHg or DBP ≥95 mmHg or use of antihypertensive medication)	-0.00096 (-0.00099, -0.00092)
Mean age of age group (centred at 50 years)	4.0e-05 (3.8e-05, 4.1e-05)
Male sex	-0.00026 (-0.00029, -0.00022)
Study mid-year (per one more recent year since 1975)	1.4e-05 (1.2e-05, 1.5e-05)
Natural logarithm of per-capita gross domestic product	3.1e-05 (8.3e-06, 5.5e-05)
Probit-transformed prevalence * mean age of age group	2.6e-05 (2.5e-05, 2.7e-05)
Probit-transformed prevalence * male sex	4.5e-05 (1.4e-05, 7.5e-05)
Random effects for regions	Yes
**Number of data points used to fit the model = 8,821**	

Traditional R^2 is not clearly defined for mixed-effect models. The conditional R^2 for the model, which describes the proportion of variance explained by both fixed and random factors,[11] was 0.629.

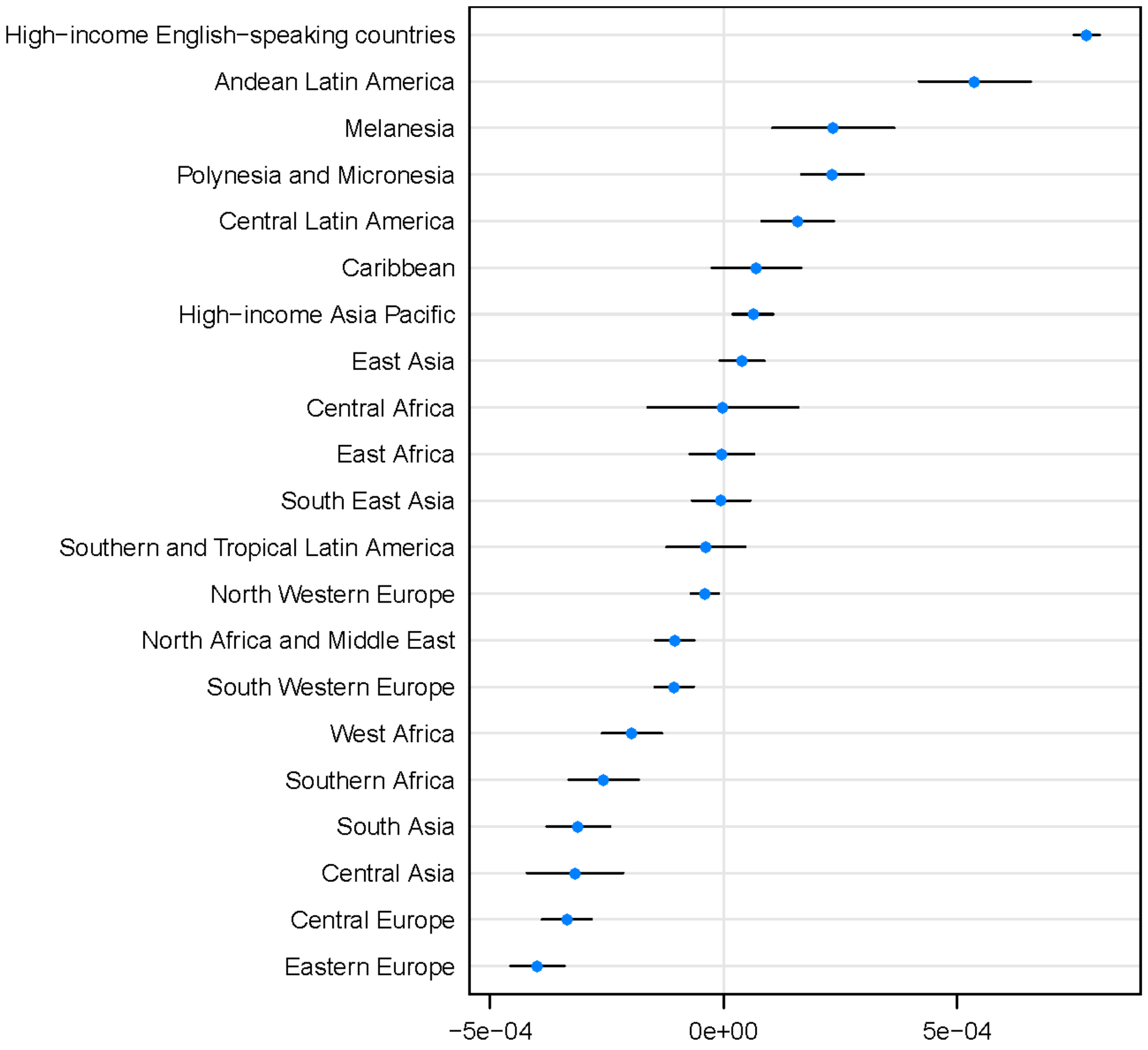
High−income English−speaking countries
Andean Latin America
Melanesia
Polynesia and Micronesia
Central Latin America
Caribbean
High−income Asia Pacific
East Asia
Central Africa
East Africa
South East Asia
Southern and Tropical Latin America
North Western Europe
North Africa and Middle East
South Western Europe
West Africa
Southern Africa
South Asia
Central Asia
Central Europe
Eastern Europe
−5e−04
0e+00
5e−04

Primary outcome: mean DBP	
**Independent variable: prevalence (SBP ≥130 mmHg or DBP ≥85 mmHg)**	
**Variables**	**Coefficients (95% CI)**
Intercept	0.012 (0.012, 0.012)
Probit-transformed prevalence (SBP ≥130 mmHg or DBP ≥85 mmHg)	-0.0012 (-0.0012, -0.0011)
Mean age of age group (centred at 50 years)	1.8e-05 (1.7e-05, 1.9e-05)
Male sex	3.0e-05 (1.8e-06, 5.7e-05)
Study mid-year (per one more recent year since 1975)	6.4e-06 (4.8e-06, 8.0e-06)
Natural logarithm of per-capita gross domestic product	1.7e-05 (-8.5e-06, 4.3e-05)
Probit-transformed prevalence * mean age of age group	1.7e-05 (1.6e-05, 1.8e-05)
Probit-transformed prevalence * male sex	-0.00026 (-0.00030, -0.00022)
Random effects for regions	Yes
**Number of data points used to fit the model = 7,558**	

Traditional R^2 is not clearly defined for mixed-effect models. The conditional R^2 for the model, which describes the proportion of variance explained by both fixed and random factors,[11] was 0.667.

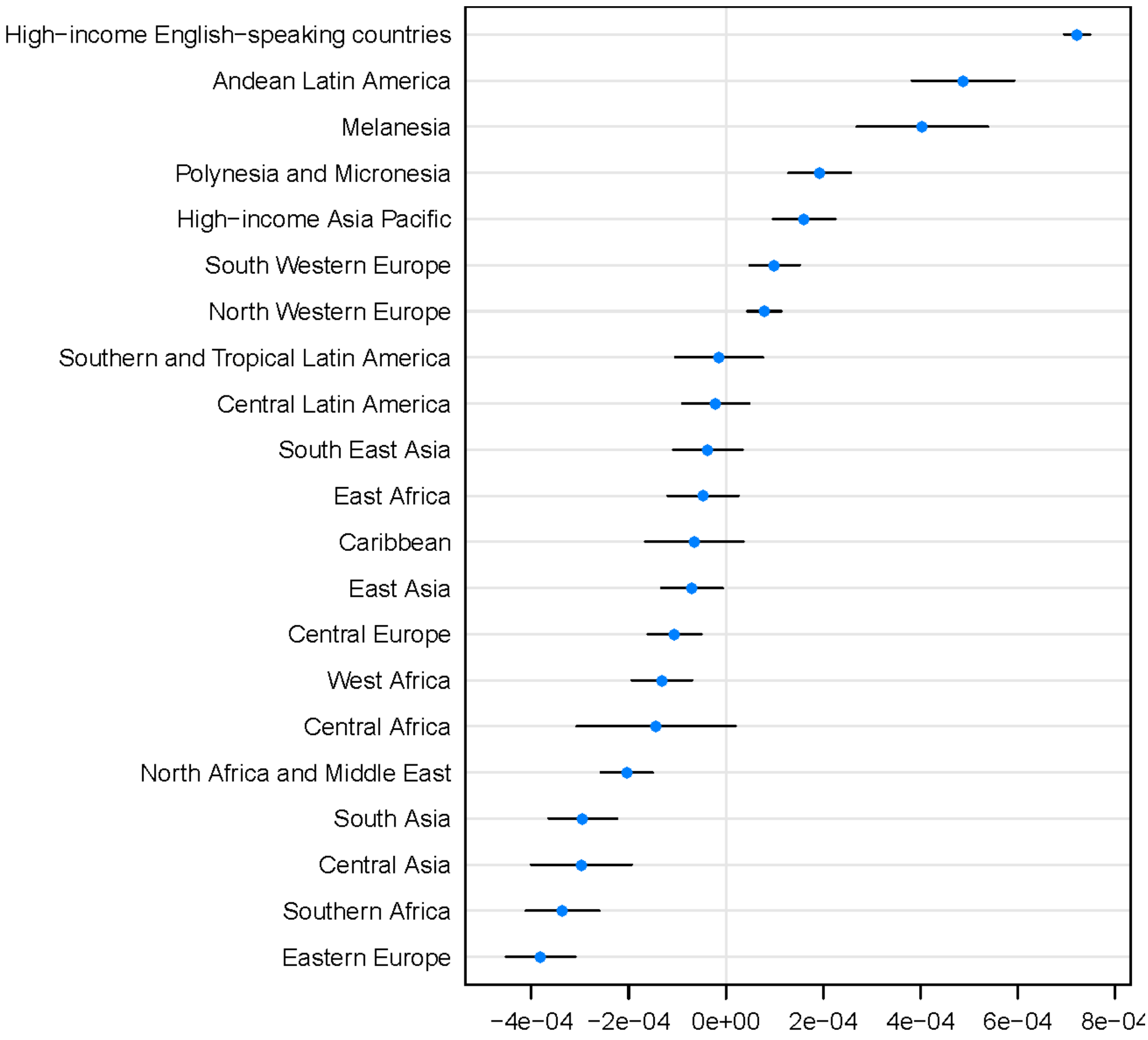

High−income English−speaking countries
Andean Latin America
Melanesia
Polynesia and Micronesia
High−income Asia Pacific
South Western Europe
North Western Europe
Southern and Tropical Latin America
Central Latin America
South East Asia
East Africa
Caribbean
East Asia
Central Europe
West Africa
Central Africa
North Africa and Middle East
South Asia
Central Asia
Southern Africa
Eastern Europe
−4e−04
−2e−04
0e+00
2e−04
4e−04
6e−04
8e−04

Primary outcome: mean DBP	
**Independent variable: prevalence (SBP ≥150 mmHg or DBP ≥90 mmHg)**	
**Variables**	**Coefficients (95% CI)**
Intercept	0.011 (0.011, 0.012)
Probit-transformed prevalence (SBP ≥150 mmHg or DBP ≥90 mmHg)	-0.0015 (-0.0015, -0.0015)
Mean age of age group (centred at 50 years)	3.4e-05 (3.3e-05, 3.5e-05)
Male sex	-0.00019 (-0.00022, -0.00015)
Study mid-year (per one more recent year since 1975)	-1.2e-06 (-2.6e-06, 1.9e-07)
Natural logarithm of per-capita gross domestic product	1.9e-05 (-1.9e-06, 4.1e-05)
Probit-transformed prevalence * mean age of age group	1.3e-05 (1.2e-05, 1.4e-05)
Probit-transformed prevalence * male sex	-0.00015 (-0.00018, -0.00012)
Random effects for regions	Yes
**Number of data points used to fit the model = 7,412**	

Traditional R^2 is not clearly defined for mixed-effect models. The conditional R^2 for the model, which describes the proportion of variance explained by both fixed and random factors,[11] was 0.748.

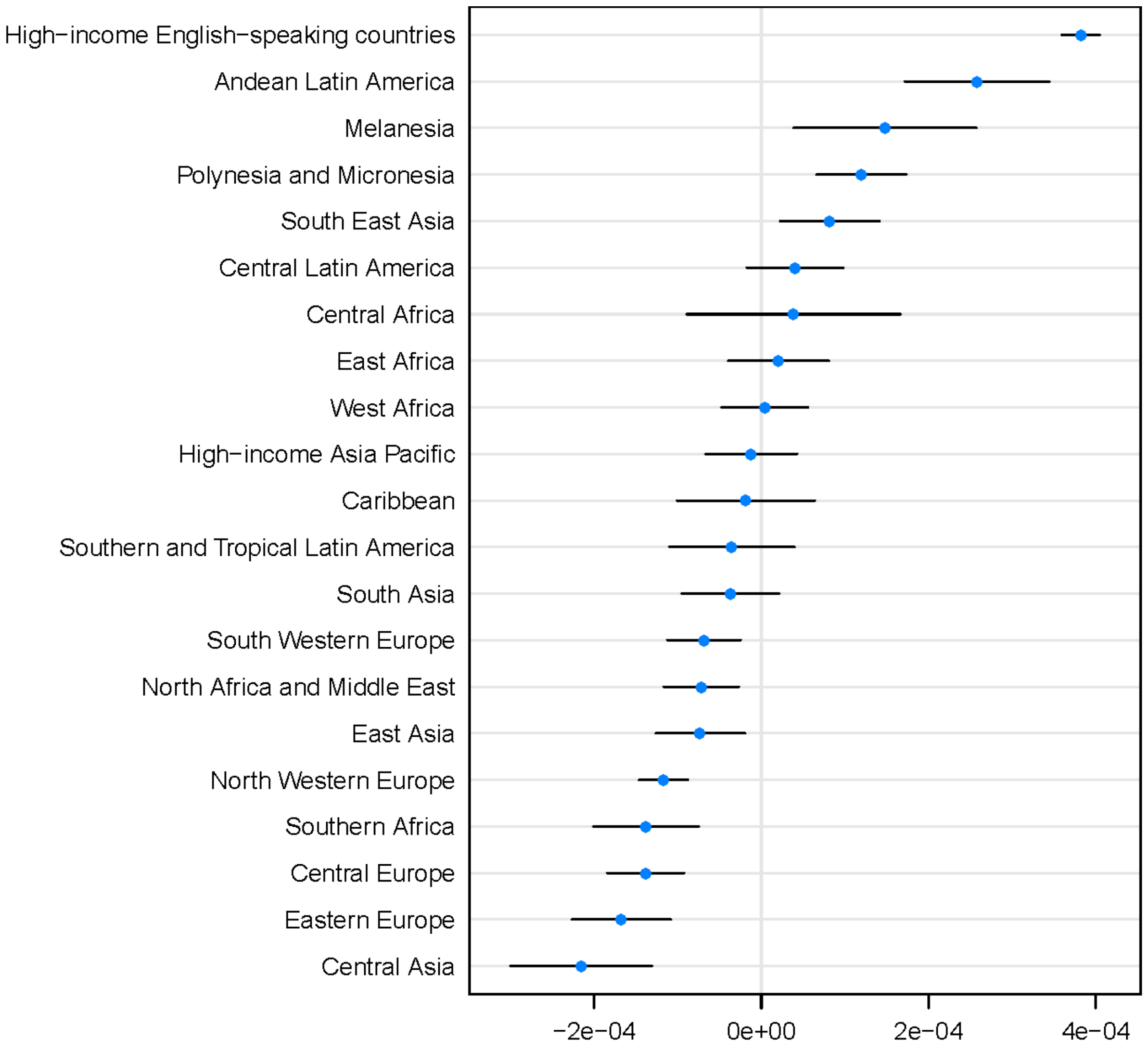

High−income English−speaking countries
Andean Latin America
Melanesia
Polynesia and Micronesia
South East Asia
Central Latin America
Central Africa
East Africa
West Africa
High−income Asia Pacific
Caribbean
Southern and Tropical Latin America
South Asia
South Western Europe
North Africa and Middle East
East Asia
North Western Europe
Southern Africa
Central Europe
Eastern Europe
Central Asia
−2e−04
0e+00
2e−04
4e−04

**Primary outcome: mean DBP**	
**Independent variable: prevalence (SBP ≥160 mmHg or DBP ≥90 mmHg)**	
**Variables**	**Coefficients (95% CI)**
Intercept	0.011 (0.011, 0.012)
Probit-transformed prevalence (SBP ≥160 mmHg or DBP ≥90 mmHg)	-0.0016 (-0.0016, -0.0016)
Mean age of age group (centred at 50 years)	2.4e-05 (2.3e-05, 2.6e-05)
Male sex	-0.00016 (-0.00019, -0.00012)
Study mid-year (per one more recent year since 1975)	-3.0e-06 (-4.3e-06, -1.7e-06)
Natural logarithm of per-capita gross domestic product	1.4e-05 (-4.5e-06, 3.4e-05)
Probit-transformed prevalence * mean age of age group	6.2e-06 (5.2e-06, 7.2e-06)
Probit-transformed prevalence * male sex	-8.2e-05 (-0.00012, -4.8e-05)
Random effects for regions	Yes
**Number of data points used to fit the model = 7,389**	

Traditional R^2 is not clearly defined for mixed-effect models. The conditional R^2 for the model, which describes the proportion of variance explained by both fixed and random factors,[11] was 0.796.

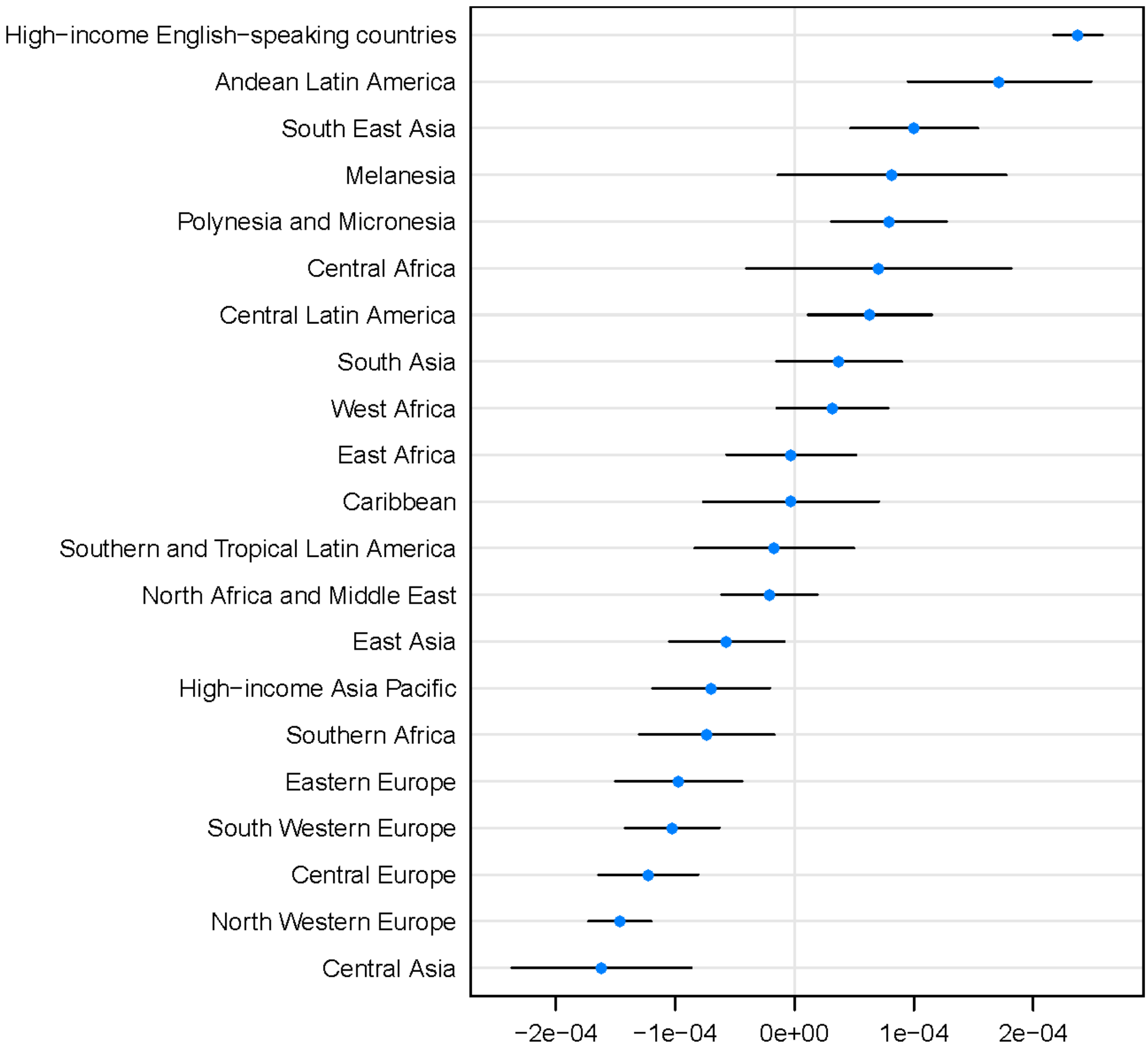
High−income English−speaking countries
Andean Latin America
South East Asia
Melanesia
Polynesia and Micronesia
Central Africa
Central Latin America
South Asia
West Africa
East Africa
Caribbean
Southern and Tropical Latin America
North Africa and Middle East
East Asia
High−income Asia Pacific
Southern Africa
Eastern Europe
South Western Europe
Central Europe
North Western Europe
Central Asia
−2e−04
−1e−04
0e+00
1e−04
2e−04

**Primary outcome: mean DBP**	
**Independent variable: prevalence (SBP ≥160 mmHg or DBP ≥95 mmHg)**	
**Variables**	**Coefficients (95% CI)**
Intercept	0.011 (0.010, 0.011)
Probit-transformed prevalence (SBP ≥160 mmHg or DBP ≥95 mmHg)	-0.0015 (-0.0015, -0.0015)
Mean age of age group (centred at 50 years)	4.0e-05 (3.8e-05, 4.2e-05)
Male sex	-0.00024 (-0.00029, -0.00019)
Study mid-year (per one more recent year since 1975)	1.7e-06 (2.7e-07, 3.1e-06)
Natural logarithm of per-capita gross domestic product	2.8e-05 (6.3e-06, 5.1e-05)
Probit-transformed prevalence * mean age of age group	1.3e-05 (1.2e-05, 1.4e-05)
Probit-transformed prevalence * male sex	-8.2e-05 (-0.00012, -4.4e-05)
Random effects for regions	Yes
**Number of data points used to fit the model = 7,469**	

Traditional R^2 is not clearly defined for mixed-effect models. The conditional R^2 for the model, which describes the proportion of variance explained by both fixed and random factors,[11] was 0.713.

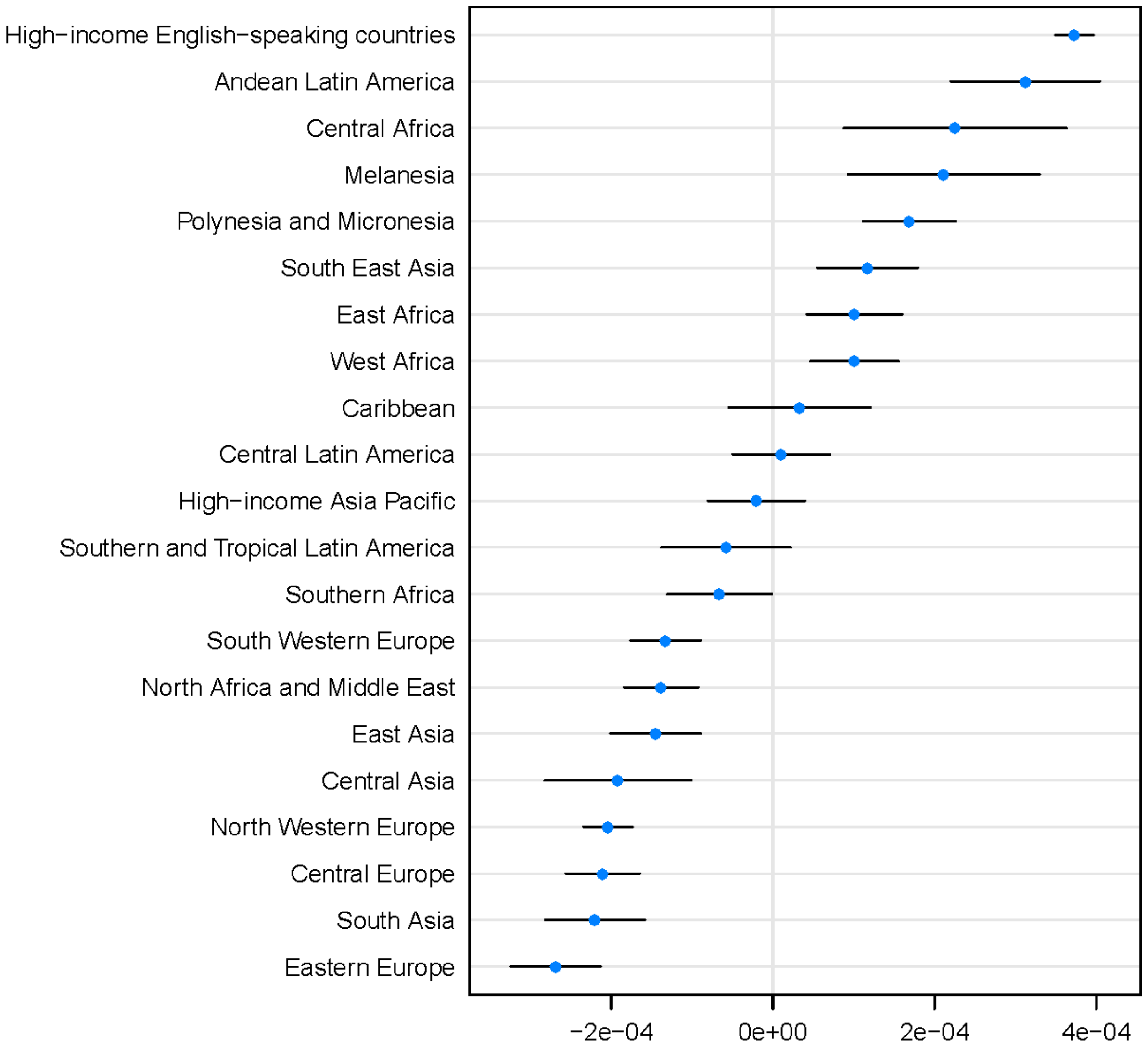

High−income English−speaking countries
Andean Latin America
Central Africa
Melanesia
Polynesia and Micronesia
South East Asia
East Africa
West Africa
Caribbean
Central Latin America
High−income Asia Pacific
Southern and Tropical Latin America
Southern Africa
South Western Europe
North Africa and Middle East
East Asia
Central Asia
North Western Europe
Central Europe
South Asia
Eastern Europe
−2e−04
0e+00
2e−04
4e−04

**Primary outcome: mean DBP**	
**Independent variable: prevalence (SBP ≥130 mmHg or DBP ≥85 mmHg or use of antihypertensive medication)**	
**Variables**	**Coefficients (95% CI)**
Intercept	0.012 (0.012, 0.012)
Probit-transformed prevalence (SBP ≥130 mmHg or DBP ≥85 mmHg or use of antihypertensive medication)	-0.00099 (-0.0010, -0.00095)
Mean age of age group (centred at 50 years)	1.8e-05 (1.6e-05, 2.0e-05)
Male sex	2.3e-05 (-8.1e-06, 5.5e-05)
Study mid-year (per one more recent year since 1975)	1.0e-05 (8.5e-06, 1.2e-05)
Natural logarithm of per-capita gross domestic product	3.3e-05 (4.8e-06, 6.0e-05)
Probit-transformed prevalence * mean age of age group	2.5e-05 (2.4e-05, 2.6e-05)
Probit-transformed prevalence * male sex	-0.00026 (-0.00030, -0.00022)
Random effects for regions	Yes
**Number of data points used to fit the model = 6,346**	

Traditional R^2 is not clearly defined for mixed-effect models. The conditional R^2 for the model, which describes the proportion of variance explained by both fixed and random factors,[11] was 0.652.

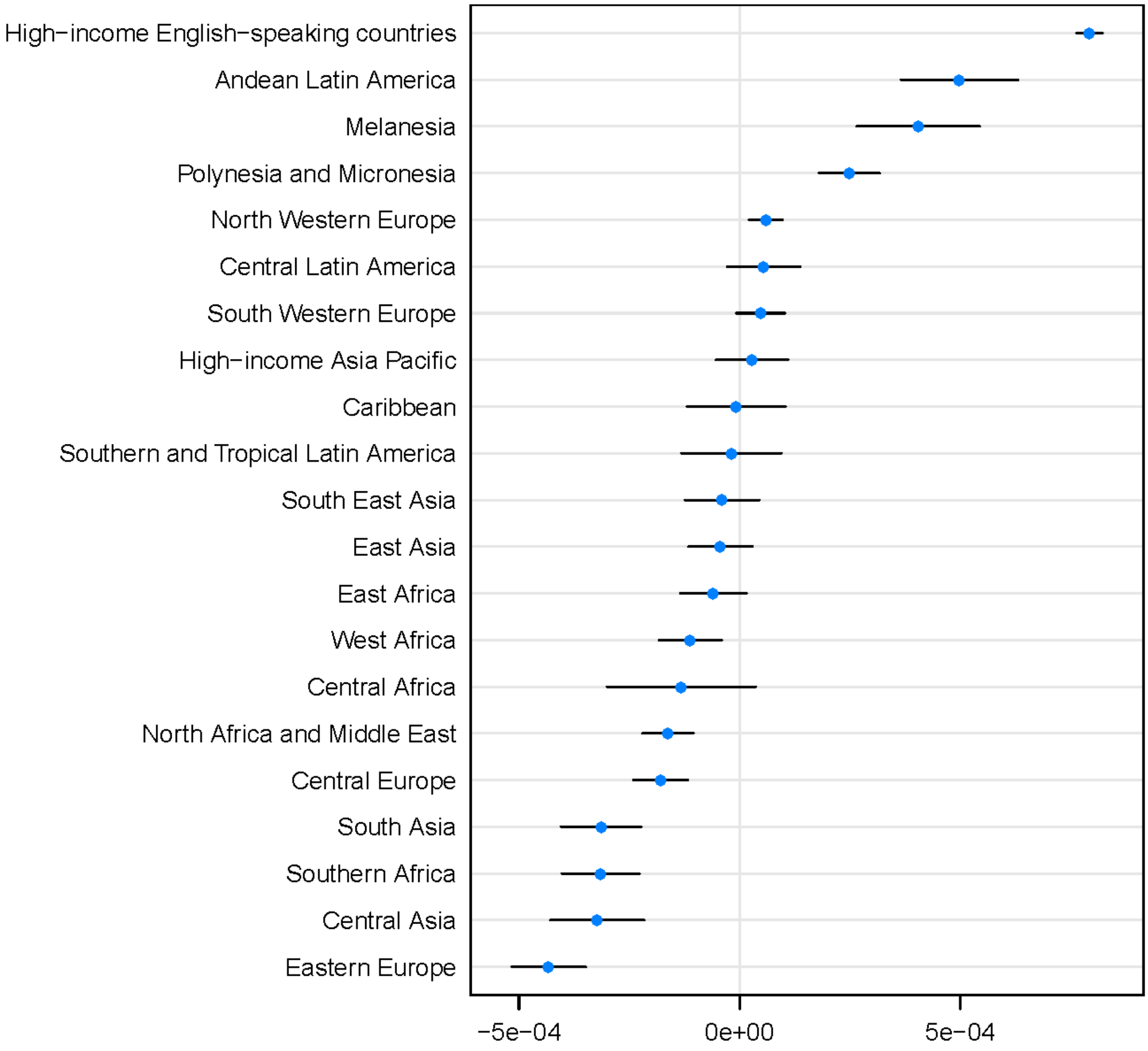
High−income English−speaking countries
Andean Latin America
Melanesia
Polynesia and Micronesia
North Western Europe
Central Latin America
South Western Europe
High−income Asia Pacific
Caribbean
Southern and Tropical Latin America
South East Asia
East Asia
East Africa
West Africa
Central Africa
North Africa and Middle East
Central Europe
South Asia
Southern Africa
Central Asia
Eastern Europe
−5e−04
0e+00
5e−04

Primary outcome: mean DBP	
**Independent variable: prevalence (SBP ≥160 mmHg or DBP ≥100 mmHg or use of antihypertensive medication)**	
**Variables**	**Coefficients (95% CI)**
Intercept	0.011 (0.011, 0.011)
Probit-transformed prevalence (SBP ≥160 mmHg or DBP ≥100 mmHg or use of antihypertensive medication)	-0.00079 (-0.00084, -0.00074)
Mean age of age group (centred at 50 years)	3.9e-05 (3.6e-05, 4.1e-05)
Male sex	-0.00024 (-0.00029, -0.00020)
Study mid-year (per one more recent year since 1975)	1.3e-05 (1.1e-05, 1.5e-05)
Natural logarithm of per-capita gross domestic product	5.4e-05 (2.3e-05, 8.4e-05)
Probit-transformed prevalence * mean age of age group	2.8e-05 (2.7e-05, 2.9e-05)
Probit-transformed prevalence * male sex	0.00011 (6.8e-05, 0.00015)
Random effects for regions	Yes
**Number of data points used to fit the model = 6,023**	

Traditional R^2 is not clearly defined for mixed-effect models. The conditional R^2 for the model, which describes the proportion of variance explained by both fixed and random factors,[11] was 0.572.

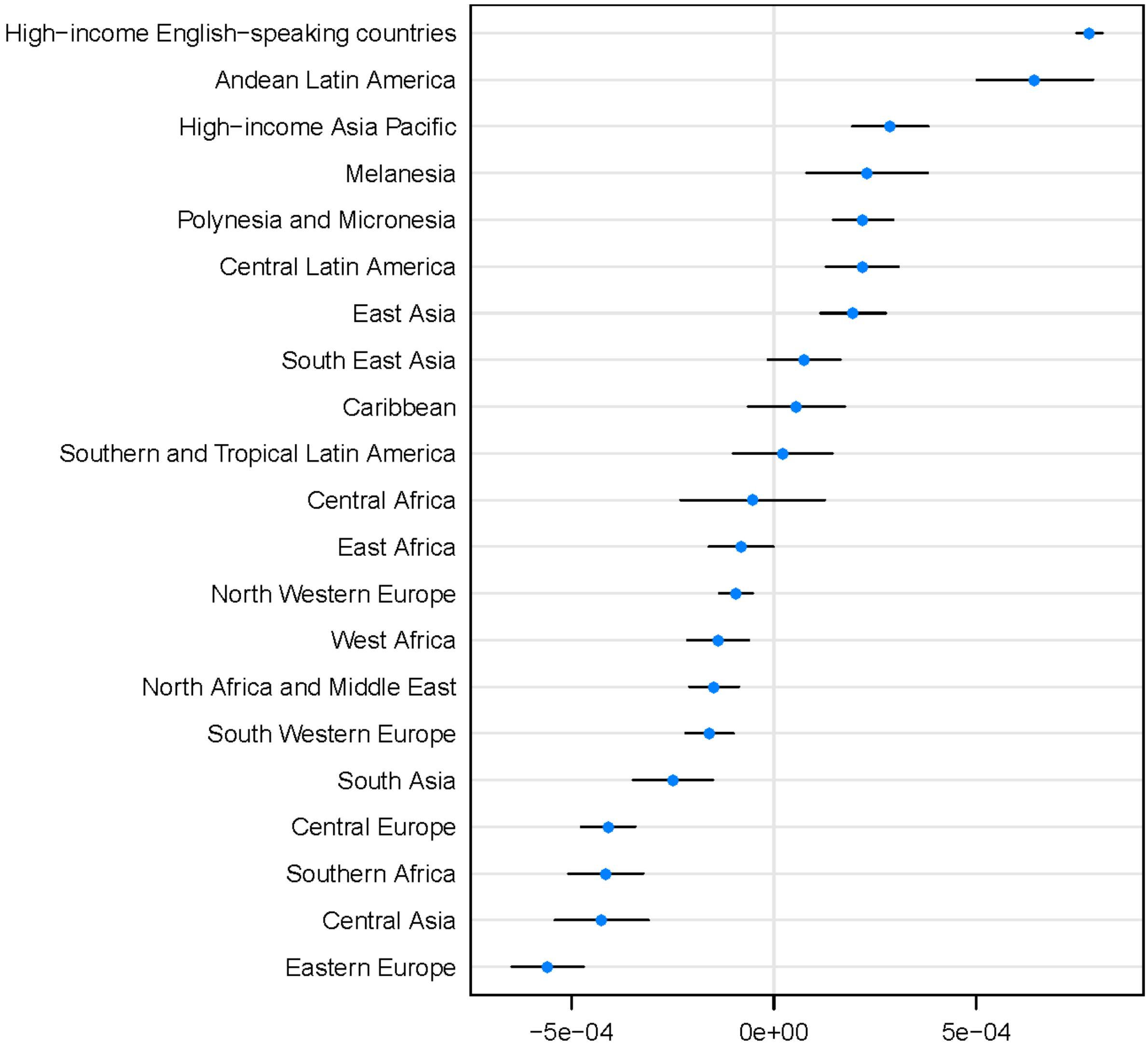
High−income English−speaking countries
Andean Latin America
High−income Asia Pacific
Melanesia
Polynesia and Micronesia
Central Latin America
East Asia
South East Asia
Caribbean
Southern and Tropical Latin America
Central Africa
East Africa
North Western Europe
West Africa
North Africa and Middle East
South Western Europe
South Asia
Central Europe
Southern Africa
Central Asia
Eastern Europe
−5e−04
0e+00
5e−04

**Primary outcome: mean DBP**	
**Independent variables: prevalence (SBP ≥140 mmHg or DBP ≥90 mmHg) and prevalence (SBP ≥140 mmHg)**	
**Variables**	**Coefficients (95% CI)**
Intercept	0.012 (0.012, 0.012)
Probit-transformed prevalence (SBP ≥140 mmHg or DBP ≥90 mmHg)	-0.0027 (-0.0028, -0.0026)
Probit-transformed prevalence (SBP ≥140 mmHg)	0.0015 (0.0014, 0.0016)
Mean age of age group (centred at 50 years)	2.8e-05 (2.7e-05, 2.9e-05)
Male sex	-5.2e-05 (-8.0e-05, -2.4e-05)
Study mid-year (per one more recent year since 1975)	-9.3e-07 (-2.1e-06, 2.1e-07)
Natural logarithm of per-capita gross domestic product	7.2e-06 (-1.1e-05, 2.6e-05)
Probit-transformed prevalence (SBP ≥140 mmHg or DBP ≥90 mmHg) * mean age of age group	-4.5e-05 (-4.8e-05, -4.1e-05)
Probit-transformed prevalence (SBP ≥140 mmHg) * mean age of age group	5.7e-05 (5.3e-05, 6.0e-05)
Probit-transformed prevalence (SBP ≥140 mmHg or DBP ≥90 mmHg) * male sex	-0.00036 (-0.00048, -0.00024)
Probit-transformed prevalence (SBP ≥140 mmHg) * male sex	0.00021 (9.8e-05, 0.00031)
Random effects for regions	Yes
**Number of data points used to fit the model = 10,516**	

Traditional R^2 is not clearly defined for mixed-effect models. The conditional R^2 for the model, which describes the proportion of variance explained by both fixed and random factors,[11] was 0.743.

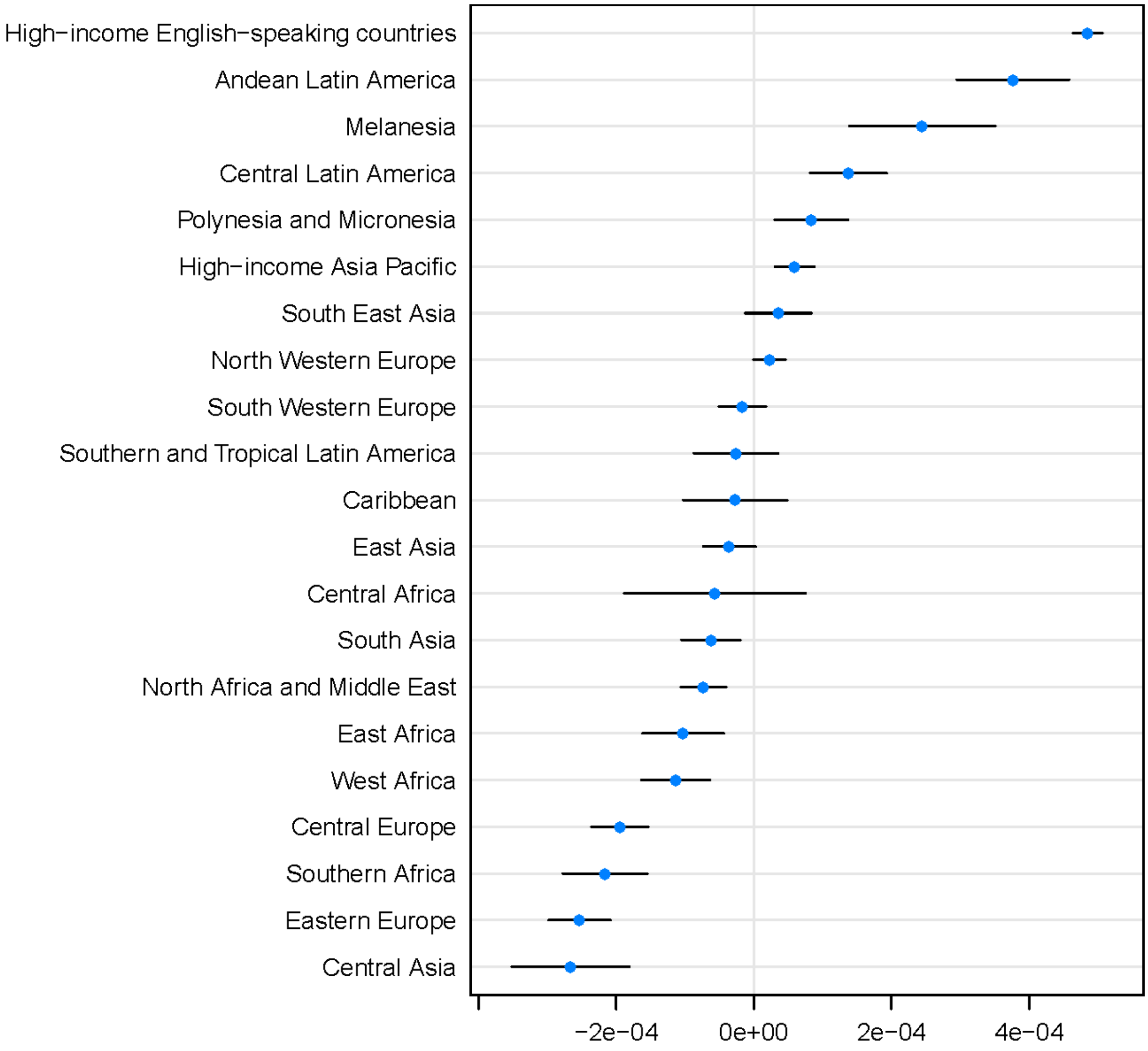
High−income English−speaking countries
Andean Latin America
Melanesia
Central Latin America
Polynesia and Micronesia
High−income Asia Pacific
South East Asia
North Western Europe
South Western Europe
Southern and Tropical Latin America
Caribbean
East Asia
Central Africa
South Asia
North Africa and Middle East
East Africa
West Africa
Central Europe
Southern Africa
Eastern Europe
Central Asia
−2e−04
0e+00
2e−04
4e−04

Primary outcome: mean DBP	
**Independent variables: prevalence (SBP ≥140 mmHg or DBP ≥90 mmHg or use of antihypertensive medication) and prevalence (SBP ≥160 mmHg or DBP ≥95 mmHg or use of antihypertensive medication)**	
**Variables**	**Coefficients (95% CI)**
Intercept	0.012 (0.012, 0.012)
Probit-transformed prevalence (SBP ≥140 mmHg or DBP ≥90 mmHg or use of antihypertensive medication)	-0.0016 (-0.0017, -0.0015)
Probit-transformed prevalence (SBP ≥160 mmHg or DBP ≥95 mmHg or use of antihypertensive medication)	0.00044 (0.00036, 0.00052)
Mean age of age group (centred at 50 years)	5.8e-05 (5.6e-05, 6.0e-05)
Male sex	-0.00038 (-0.00043, -0.00032)
Study mid-year (per one more recent year since 1975)	7.4e-06 (6.0e-06, 8.7e-06)
Natural logarithm of per-capita gross domestic product	2.0e-05 (-6.1e-07, 4.0e-05)
Probit-transformed prevalence (SBP ≥140 mmHg or DBP ≥90 mmHg or use of antihypertensive medication) * mean age of age group	-1.9e-05 (-2.2e-05, -1.6e-05)
Probit-transformed prevalence (SBP ≥160 mmHg or DBP ≥95 mmHg or use of antihypertensive medication) * mean age of age group	4.5e-05 (4.2e-05, 4.7e-05)
Probit-transformed prevalence (SBP ≥140 mmHg or DBP ≥90 mmHg or use of antihypertensive medication) * male sex	0.00039 (0.00028, 0.00049)
Probit-transformed prevalence (SBP ≥160 mmHg or DBP ≥95 mmHg or use of antihypertensive medication) * male sex	-0.00043 (-0.00054, -0.00033)
Random effects for regions	Yes
**Number of data points used to fit the model = 8,723**	

Traditional R^2 is not clearly defined for mixed-effect models. The conditional R^2 for the model, which describes the proportion of variance explained by both fixed and random factors,[11] was 0.716.

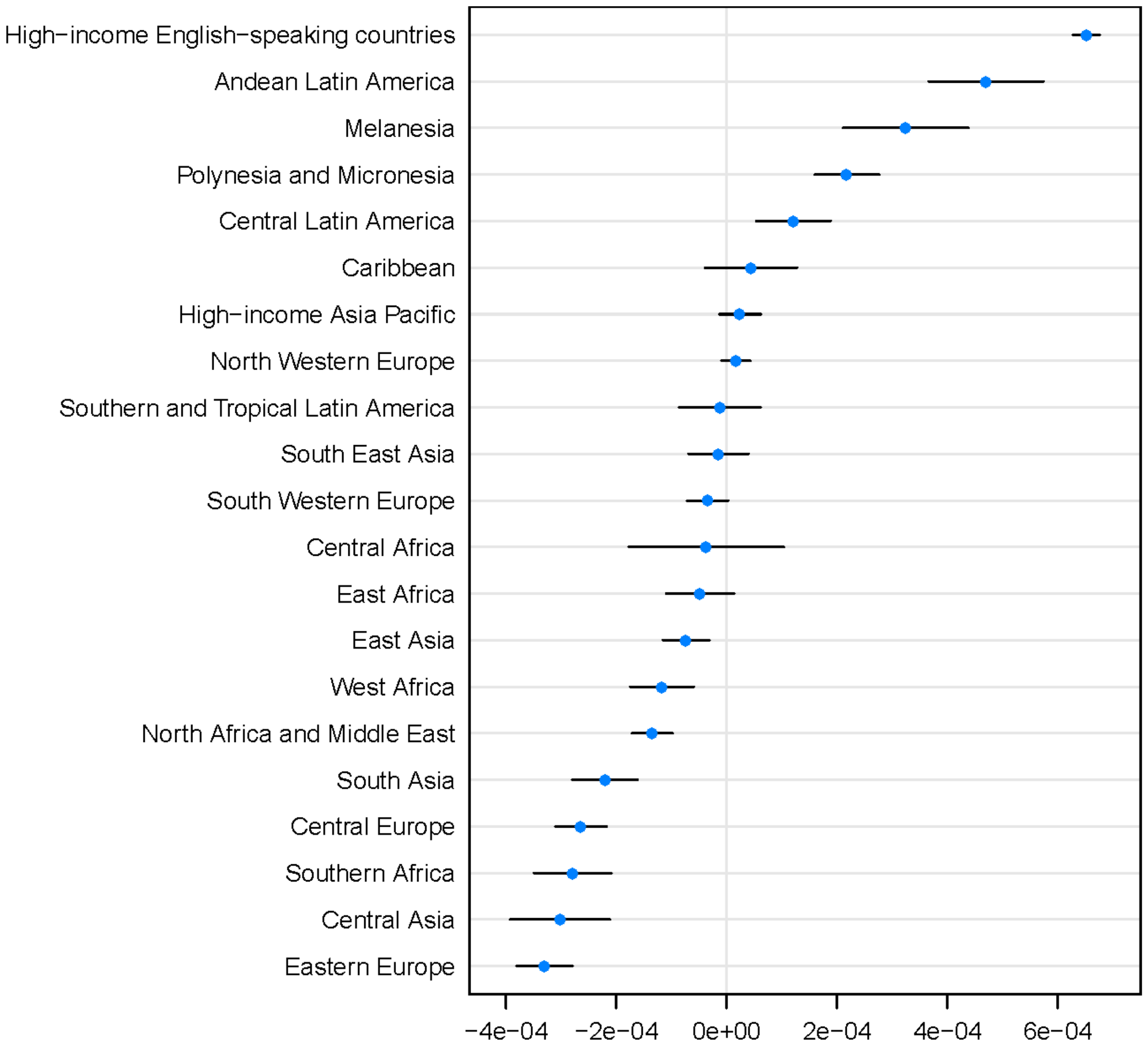

High−income English−speaking countries
Andean Latin America
Melanesia
Polynesia and Micronesia
Central Latin America
Caribbean
High−income Asia Pacific
North Western Europe
Southern and Tropical Latin America
South East Asia
South Western Europe
Central Africa
East Africa
East Asia
West Africa
North Africa and Middle East
South Asia
Central Europe
Southern Africa
Central Asia
Eastern Europe
−4e−04
−2e−04
0e+00
2e−04
4e−04
6e−04

**Primary outcome: mean DBP**	
**Independent variables: prevalence (SBP ≥140 mmHg or DBP ≥90 mmHg or use of antihypertensive medication) and prevalence (SBP ≥140 mmHg or DBP ≥90 mmHg)**	
**Variables**	**Coefficients (95% CI)**
Intercept	0.012 (0.012, 0.012)
Probit-transformed prevalence (SBP ≥140 mmHg or DBP ≥90 mmHg or use of antihypertensive medication)	-7.3e-05 (-0.00015, 4.8e-06)
Probit-transformed prevalence (SBP ≥140 mmHg or DBP ≥90 mmHg)	-0.0011 (-0.0012, -0.0011)
Mean age of age group (centred at 50 years)	2.0e-05 (1.8e-05, 2.1e-05)
Male sex	-8.0e-05 (-0.00011, -4.8e-05)
Study mid-year (per one more recent year since 1975)	-3.0e-07 (-1.6e-06, 9.8e-07)
Natural logarithm of per-capita gross domestic product	-7.0e-06 (-2.6e-05, 1.2e-05)
Probit-transformed prevalence (SBP ≥140 mmHg or DBP ≥90 mmHg or use of antihypertensive medication) * mean age of age group	4.5e-05 (4.3e-05, 4.8e-05)
Probit-transformed prevalence (SBP ≥140 mmHg or DBP ≥90 mmHg) * mean age of age group	-3.0e-05 (-3.2e-05, -2.7e-05)
Probit-transformed prevalence (SBP ≥140 mmHg or DBP ≥90 mmHg or use of antihypertensive medication) * male sex	-0.00022 (-0.00031, -0.00014)
Probit-transformed prevalence (SBP ≥140 mmHg or DBP ≥90 mmHg) * male sex	0.00014 (4.9e-05, 0.00024)
Random effects for regions	Yes
**Number of data points used to fit the model = 8,885**	

Traditional R^2 is not clearly defined for mixed-effect models. The conditional R^2 for the model, which describes the proportion of variance explained by both fixed and random factors,[11] was 0.762.

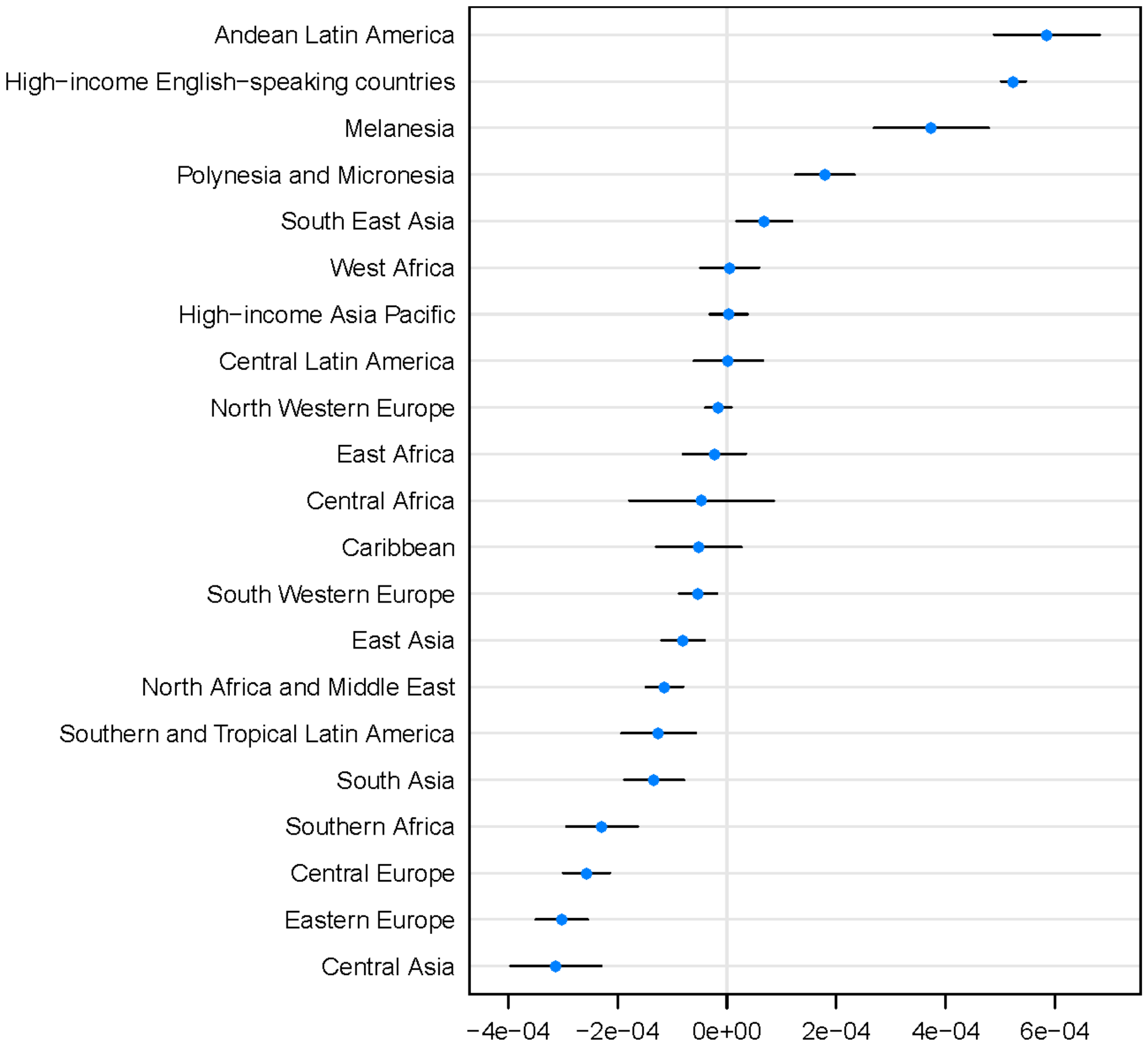

Andean Latin America
High−income English−speaking countries
Melanesia
Polynesia and Micronesia
South East Asia
West Africa
High−income Asia Pacific
Central Latin America
North Western Europe
East Africa
Central Africa
Caribbean
South Western Europe
East Asia
North Africa and Middle East
Southern and Tropical Latin America
South Asia
Southern Africa
Central Europe
Eastern Europe
Central Asia
−4e−04
−2e−04
0e+00
2e−04
4e−04
6e−04

Primary outcome: mean DBP	
**Independent variables: prevalence (SBP ≥160 mmHg) and prevalence (DBP ≥95 mmHg)**	
**Variables**	**Coefficients (95% CI)**
Intercept	0.010 (0.010, 0.011)
Probit-transformed prevalence (SBP ≥160 mmHg)	-0.00010 (-0.00014, -6.5e-05)
Probit-transformed prevalence (DBP ≥95 mmHg)	-0.0014 (-0.0014, -0.0014)
Mean age of age group (centred at 50 years)	4.8e-06 (2.8e-06, 6.8e-06)
Male sex	2.9e-05 (-2.2e-05, 8.0e-05)
Study mid-year (per one more recent year since 1975)	1.9e-06 (7.9e-07, 3.0e-06)
Natural logarithm of per-capita gross domestic product	-5.9e-06 (-2.3e-05, 1.1e-05)
Probit-transformed prevalence (SBP ≥160 mmHg) * mean age of age group	8.5e-06 (7.4e-06, 9.6e-06)
Probit-transformed prevalence (DBP ≥95 mmHg) * mean age of age group	-7.5e-06 (-8.8e-06, -6.3e-06)
Probit-transformed prevalence (SBP ≥160 mmHg) * male sex	7.8e-05 (4.5e-05, 0.00011)
Probit-transformed prevalence (DBP ≥95 mmHg) * male sex	5.9e-06 (-3.6e-05, 4.7e-05)
Random effects for regions	Yes
**Number of data points used to fit the model = 6,257**	

Traditional R^2 is not clearly defined for mixed-effect models. The conditional R^2 for the model, which describes the proportion of variance explained by both fixed and random factors,[11] was 0.850.

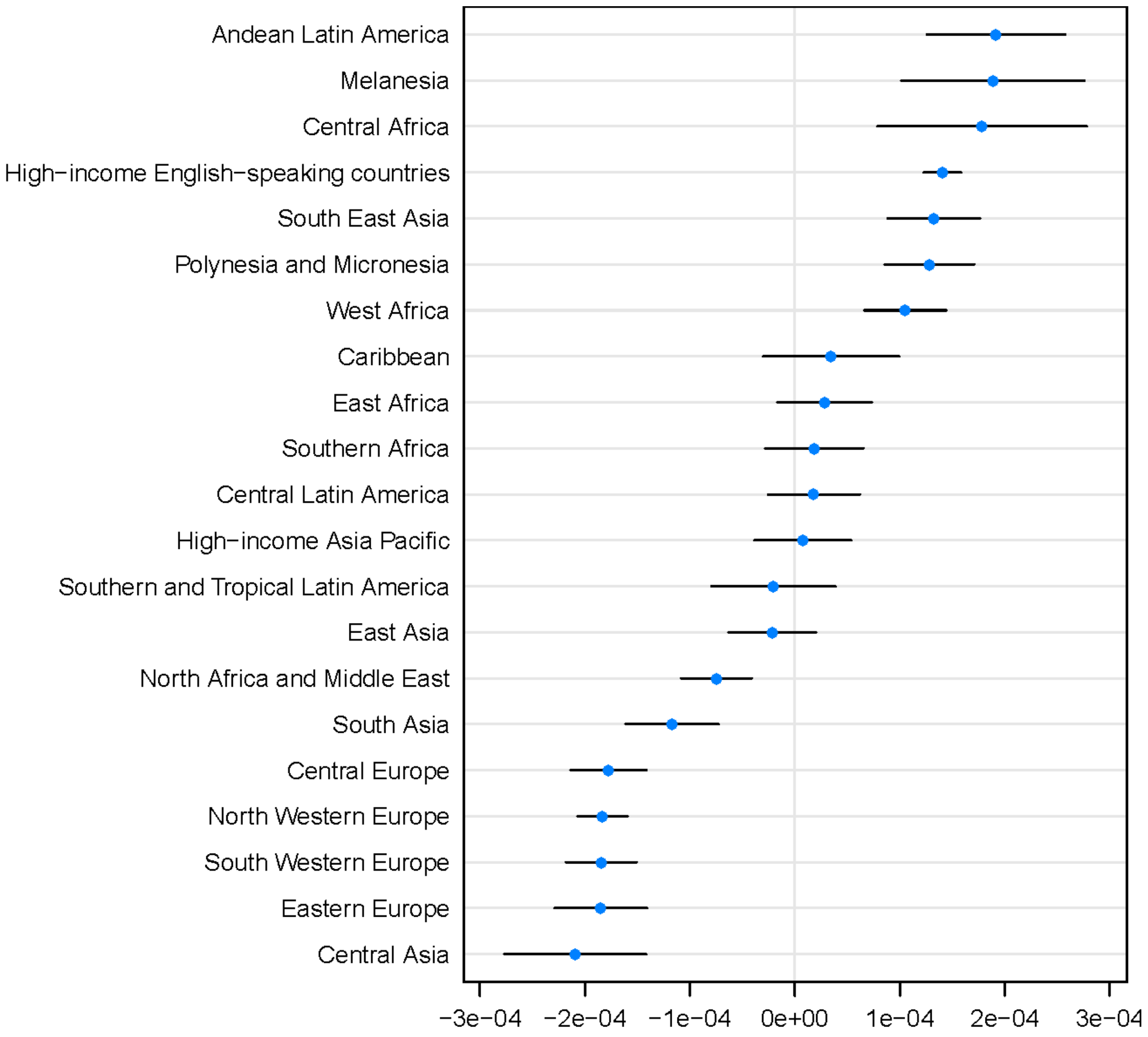

Andean Latin America
Melanesia
Central Africa
High−income English−speaking countries
South East Asia
Polynesia and Micronesia
West Africa
Caribbean
East Africa
Southern Africa
Central Latin America
High−income Asia Pacific
Southern and Tropical Latin America
East Asia
North Africa and Middle East
South Asia
Central Europe
North Western Europe
South Western Europe
Eastern Europe
Central Asia
−3e−04
−2e−04
−1e−04
0e+00
1e−04
2e−04
3e−04

Primary outcome: mean DBP	
**Independent variables: mean SBP and prevalence (SBP ≥140 mmHg or DBP ≥90 mmHg or use of antihypertensive medication)**	
**Variables**	**Coefficients (95% CI)**
Intercept	0.012 (0.012, 0.012)
Mean SBP (centred at 130 mmHg)	-5.3e-05 (-5.6e-05, -5.0e-05)
Probit-transformed prevalence (SBP ≥140 mmHg or DBP ≥90 mmHg or use of antihypertensive medication)	-0.00030 (-0.00035, -0.00024)
Mean age of age group (centred at 50 years)	3.8e-05 (3.6e-05, 3.9e-05)
Male sex	-0.00024 (-0.00027, -0.00021)
Study mid-year (per one more recent year since 1975)	3.6e-06 (2.3e-06, 4.9e-06)
Natural logarithm of per-capita gross domestic product	1.3e-05 (-6.9e-06, 3.3e-05)
Mean SBP * mean age of age group	-8.6e-07 (-9.7e-07, -7.4e-07)
Probit-transformed prevalence * mean age of age group	3.9e-05 (3.7e-05, 4.0e-05)
Mean SBP * male sex	2.1e-05 (1.7e-05, 2.5e-05)
Probit-transformed prevalence * male sex	-0.00046 (-0.00052, -0.00040)
Random effects for regions	Yes
**Number of data points used to fit the model = 9,124**	

Traditional R^2 is not clearly defined for mixed-effect models. The conditional R^2 for the model, which describes the proportion of variance explained by both fixed and random factors,[11] was 0.735.

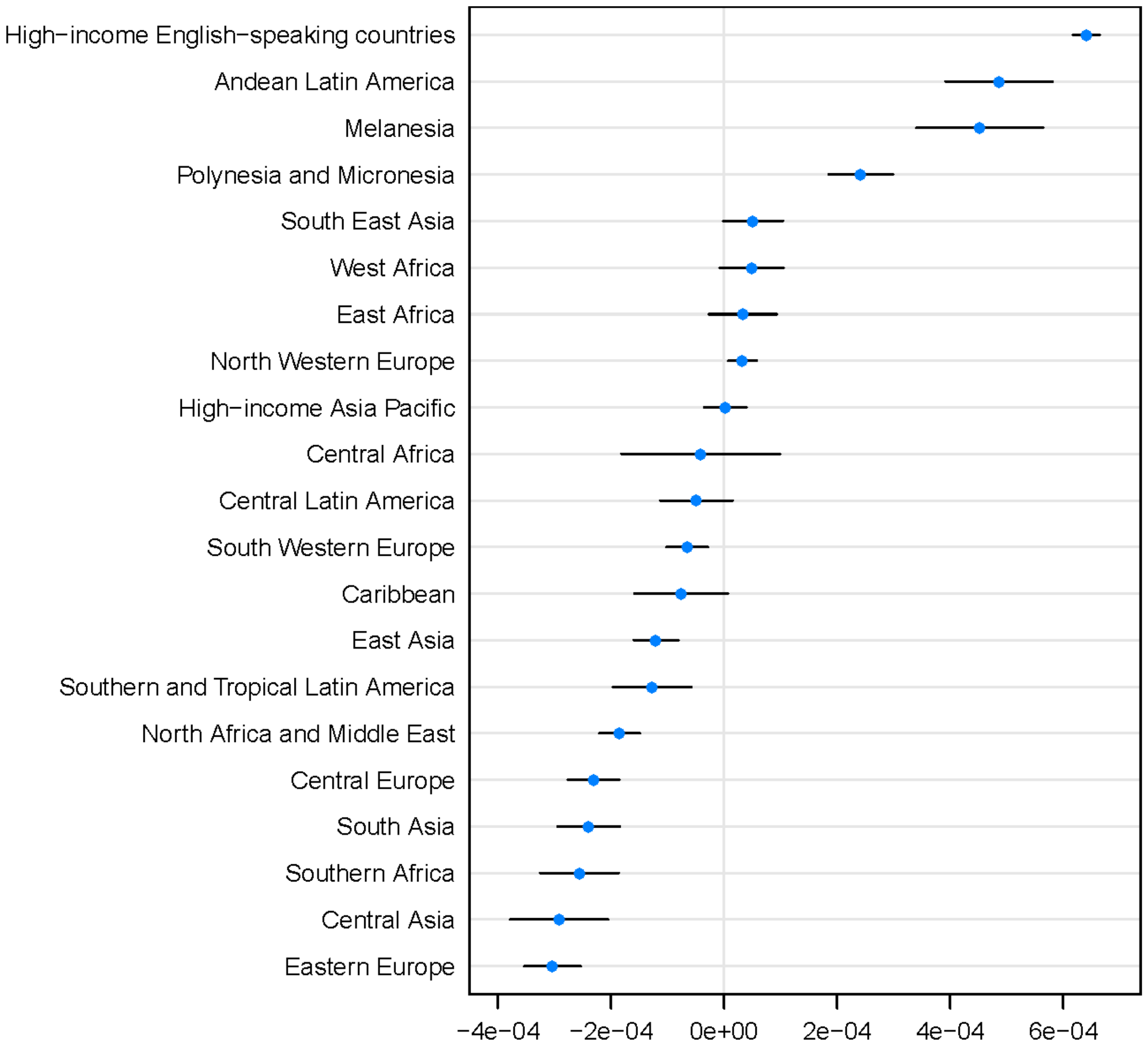
High−income English−speaking countries
Andean Latin America
Melanesia
Polynesia and Micronesia
South East Asia
West Africa
East Africa
North Western Europe
High−income Asia Pacific
Central Africa
Central Latin America
South Western Europe
Caribbean
East Asia
Southern and Tropical Latin America
North Africa and Middle East
Central Europe
South Asia
Southern Africa
Central Asia
Eastern Europe
−4e−04
−2e−04
0e+00
2e−04
4e−04
6e−04

Primary outcome: mean DBP	
**Independent variables: mean SBP and prevalence (SBP ≥160 mmHg or DBP ≥95 mmHg or use of antihypertensive medication)**	
**Variables**	**Coefficients (95% CI)**
Intercept	0.012 (0.012, 0.012)
Mean SBP (centred at 130 mmHg)	-6.0e-05 (-6.3e-05, -5.8e-05)
Probit-transformed prevalence (SBP ≥160 mmHg or DBP ≥95 mmHg or use of antihypertensive medication)	-0.00030 (-0.00034, -0.00026)
Mean age of age group (centred at 50 years)	5.5e-05 (5.3e-05, 5.6e-05)
Male sex	-0.00035 (-0.00039, -0.00031)
Study mid-year (per one more recent year since 1975)	5.0e-06 (3.7e-06, 6.2e-06)
Natural logarithm of per-capita gross domestic product	1.0e-05 (-8.6e-06, 3.0e-05)
Mean SBP * mean age of age group	-2.9e-07 (-3.7e-07, -2.0e-07)
Probit-transformed prevalence * mean age of age group	3.3e-05 (3.2e-05, 3.4e-05)
Mean SBP * male sex	1.2e-05 (8.4e-06, 1.5e-05)
Probit-transformed prevalence * male sex	-0.00026 (-0.00031, -0.00021)
Random effects for regions	Yes
**Number of data points used to fit the model = 8,821**	

Traditional R^2 is not clearly defined for mixed-effect models. The conditional R^2 for the model, which describes the proportion of variance explained by both fixed and random factors,[11] was 0.752.

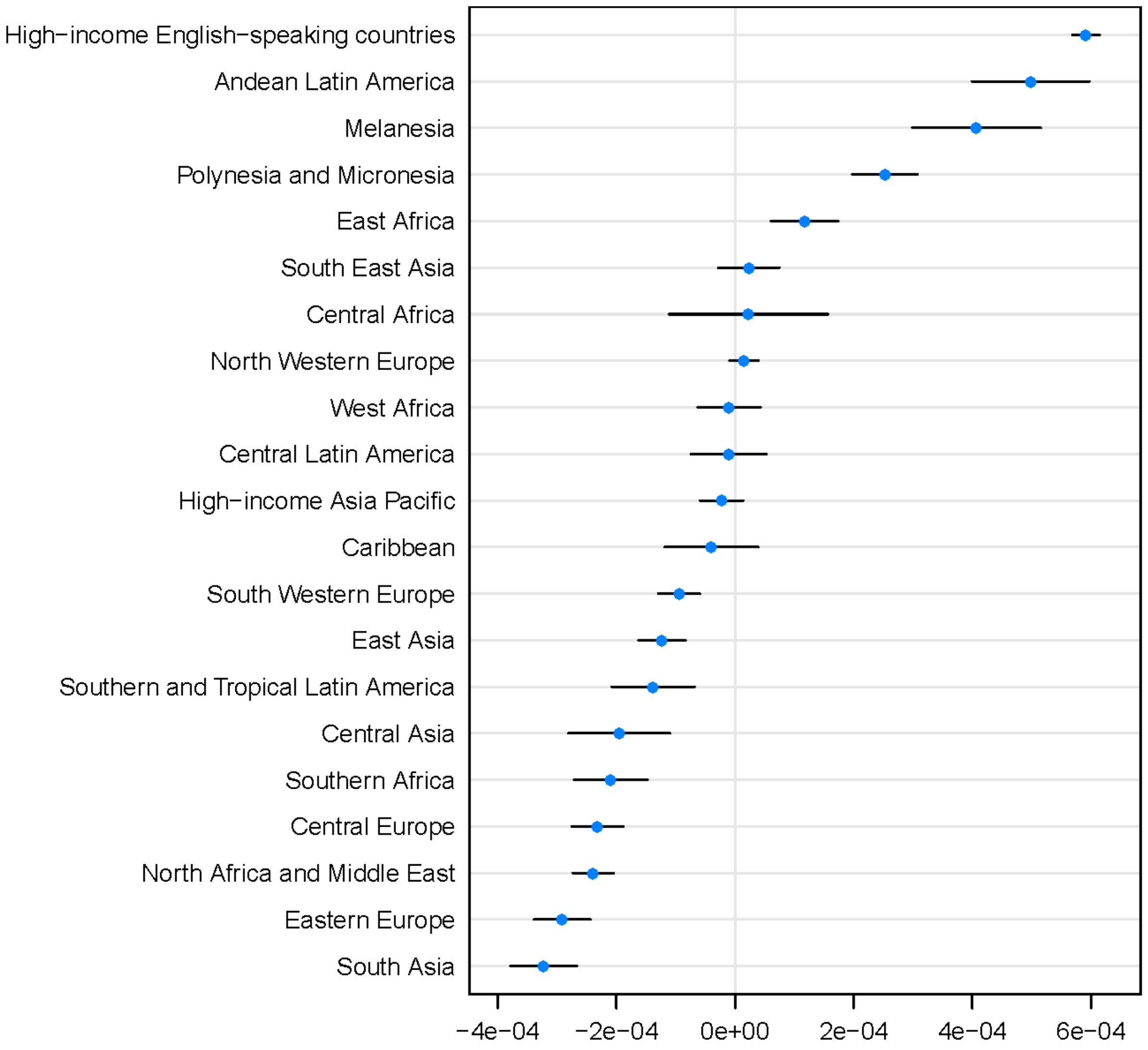
High−income English−speaking countries
Andean Latin America
Melanesia
Polynesia and Micronesia
East Africa
South East Asia
Central Africa
North Western Europe
West Africa
Central Latin America
High−income Asia Pacific
Caribbean
South Western Europe
East Asia
Southern and Tropical Latin America
Central Asia
Southern Africa
Central Europe
North Africa and Middle East
Eastern Europe
South Asia
−4e−04
−2e−04
0e+00
2e−04
4e−04
6e−04

**Primary outcome: mean DBP**	
**Independent variables: mean SBP and prevalence (SBP ≥160 mmHg)**	
**Variables**	**Coefficients (95% CI)**
Intercept	0.012 (0.012, 0.012)
Mean SBP (centred at 130 mmHg)	-7.4e-05 (-7.9e-05, -6.8e-05)
Probit-transformed prevalence (SBP ≥160 mmHg)	5.6e-05 (-4.0e-05, 0.00015)
Mean age of age group (centred at 50 years)	8.0e-05 (7.5e-05, 8.5e-05)
Male sex	-0.00051 (-0.00069, -0.00033)
Study mid-year (per one more recent year since 1975)	9.9e-06 (8.1e-06, 1.2e-05)
Natural logarithm of per-capita gross domestic product	8.9e-06 (-1.8e-05, 3.6e-05)
Mean SBP * mean age of age group	-5.3e-07 (-7.2e-07, -3.3e-07)
Probit-transformed prevalence * mean age of age group	3.7e-05 (3.4e-05, 4.0e-05)
Mean SBP * male sex	7.9e-06 (8.2e-07, 1.5e-05)
Probit-transformed prevalence * male sex	-0.00027 (-0.00039, -0.00016)
Random effects for regions	Yes
**Number of data points used to fit the model = 6,423**	

Traditional R^2 is not clearly defined for mixed-effect models. The conditional R^2 for the model, which describes the proportion of variance explained by both fixed and random factors,[11] was 0.625.

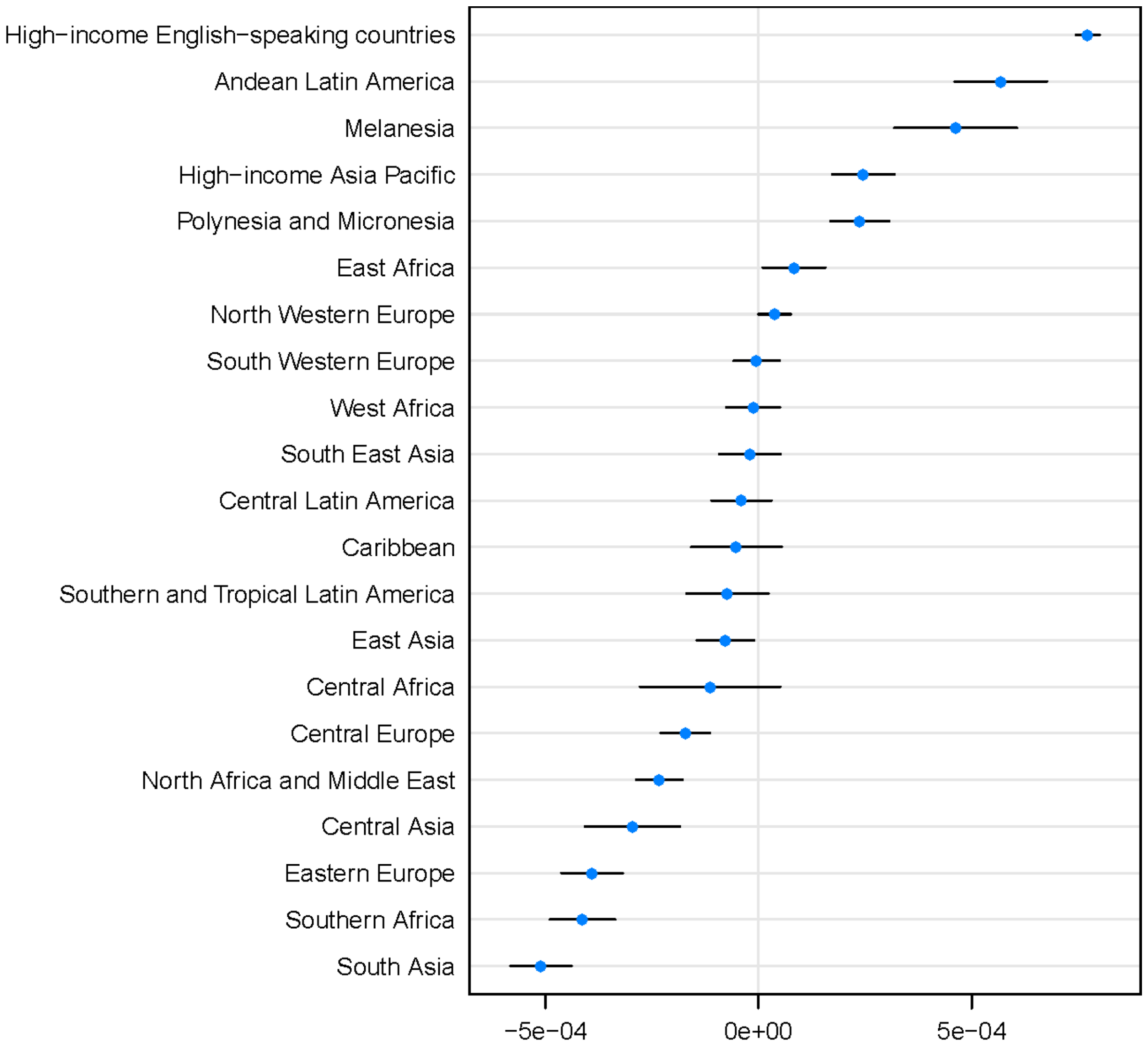
High−income English−speaking countries
Andean Latin America
Melanesia
High−income Asia Pacific
Polynesia and Micronesia
East Africa
North Western Europe
South Western Europe
West Africa
South East Asia
Central Latin America
Caribbean
Southern and Tropical Latin America
East Asia
Central Africa
Central Europe
North Africa and Middle East
Central Asia
Eastern Europe
Southern Africa
South Asia
−5e−04
0e+00
5e−04

Primary outcome: mean DBP	
**Independent variables: mean SBP, prevalence (SBP ≥140 mmHg or DBP ≥90 mmHg), and prevalence (SBP ≥160 mmHg or DBP ≥95 mmHg)**	
**Variables**	**Coefficients (95% CI)**
Intercept	0.011 (0.011, 0.011)
Mean SBP (centred at 130 mmHg)	5.2e-06 (4.7e-07, 9.9e-06)
Probit-transformed prevalence (SBP ≥140 mmHg or DBP ≥90 mmHg)	-0.00065 (-0.00075, -0.00054)
Probit-transformed prevalence (SBP ≥160 mmHg or DBP ≥95 mmHg)	-0.00099 (-0.0011, -0.00091)
Mean age of age group (centred at 50 years)	1.5e-05 (1.1e-05, 1.8e-05)
Male sex	-0.00045 (-0.00055, -0.00035)
Study mid-year (per one more recent year since 1975)	6.3e-07 (-7.7e-07, 2.0e-06)
Natural logarithm of per-capita gross domestic product	1.4e-05 (-7.2e-06, 3.5e-05)
Mean SBP * mean age of age group	1.5e-06 (1.4e-06, 1.7e-06)
Probit-transformed prevalence (SBP ≥140 mmHg or DBP ≥90 mmHg) * mean age of age group	-4.4e-06 (-7.9e-06, -9.4e-07)
Probit-transformed prevalence (SBP ≥160 mmHg or DBP ≥95 mmHg) * mean age of age group	-7.1e-06 (-9.9e-06, -4.3e-06)
Mean SBP * male sex	7.6e-06 (1.9e-06, 1.3e-05)
Probit-transformed prevalence (SBP ≥140 mmHg or DBP ≥90 mmHg) * male sex	1.6e-05 (-0.00012, 0.00015)
Probit-transformed prevalence (SBP ≥160 mmHg or DBP ≥95 mmHg) * male sex	-0.00033 (-0.00044, -0.00023)
Random effects for regions	Yes
**Number of data points used to fit the model = 7,329**	

Traditional R^2 is not clearly defined for mixed-effect models. The conditional R^2 for the model, which describes the proportion of variance explained by both fixed and random factors,[11] was 0.747.

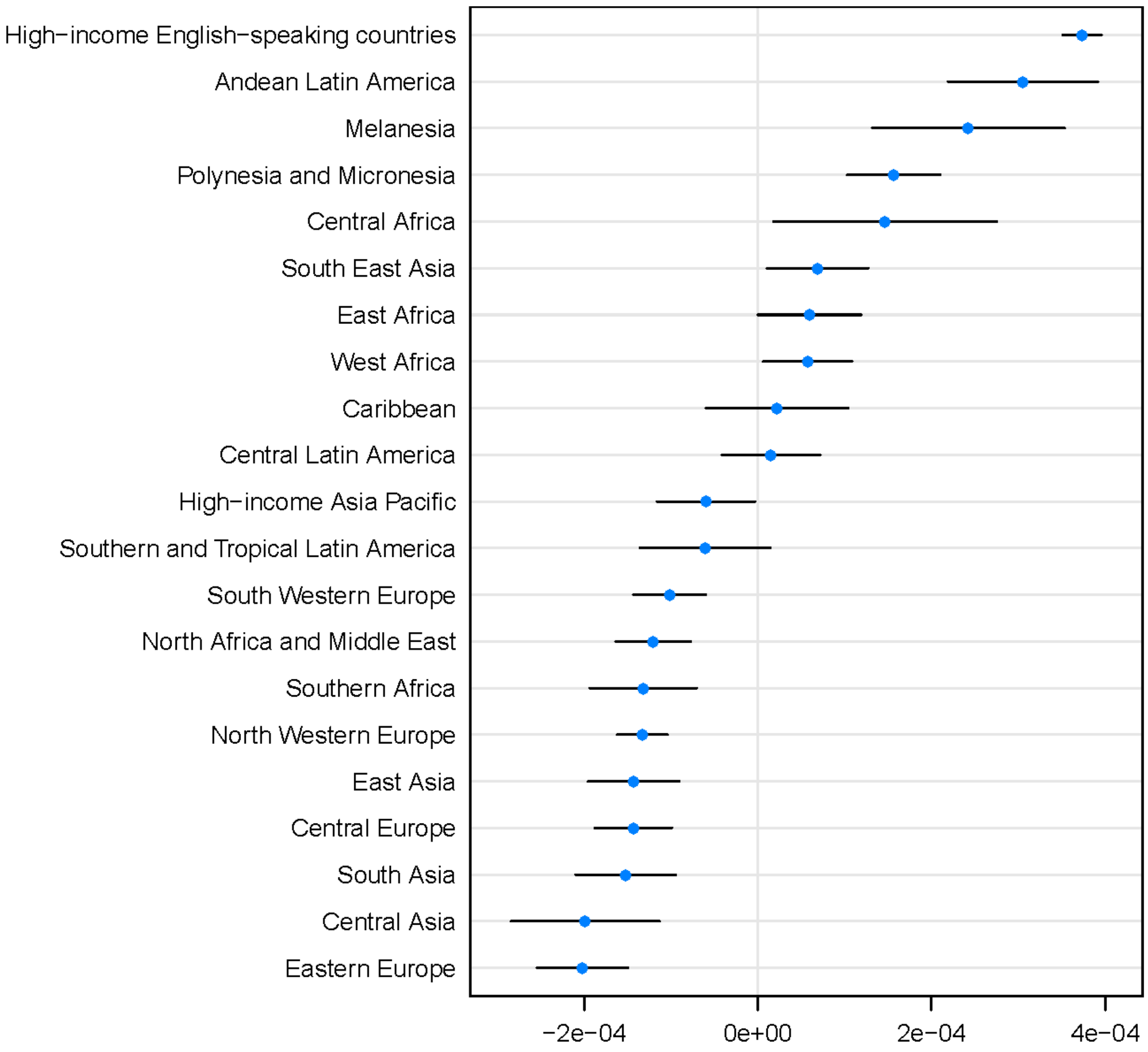
High−income English−speaking countries
Andean Latin America
Melanesia
Polynesia and Micronesia
Central Africa
South East Asia
East Africa
West Africa
Caribbean
Central Latin America
High−income Asia Pacific
Southern and Tropical Latin America
South Western Europe
North Africa and Middle East
Southern Africa
North Western Europe
East Asia
Central Europe
South Asia
Central Asia
Eastern Europe
−2e−04
0e+00
2e−04
4e−04

Primary outcome: prevalence of raised blood pressure (SBP ≥140 mmHg or DBP ≥90 mmHg)	
**Independent variable: mean SBP**	
**Variables**	**Coefficients (95% CI)**
Intercept	-0.52 (-0.58, -0.47)
Mean SBP (centred at 130 mmHg)	0.047 (0.047, 0.047)
Mean age of age group (centred at 50 years)	0.0071 (0.0070, 0.0072)
Male sex	0.014 (0.012, 0.015)
Study mid-year (per one more recent year since 1975)	-0.0030 (-0.0031, -0.0028)
Natural logarithm of per-capita gross domestic product	0.018 (0.015, 0.020)
Mean SBP * mean age of age group	-0.00037 (-0.00038, -0.00037)
Mean SBP * male sex	0.0029 (0.0027, 0.0031)
Random effects for regions	Yes
**Number of data points used to fit the model = 10,974**	

Traditional R^2 is not clearly defined for mixed-effect models. The pseudo-R^2 for the model, which describes the proportion of variance explained by both fixed and random factors,[12] was 0.909.

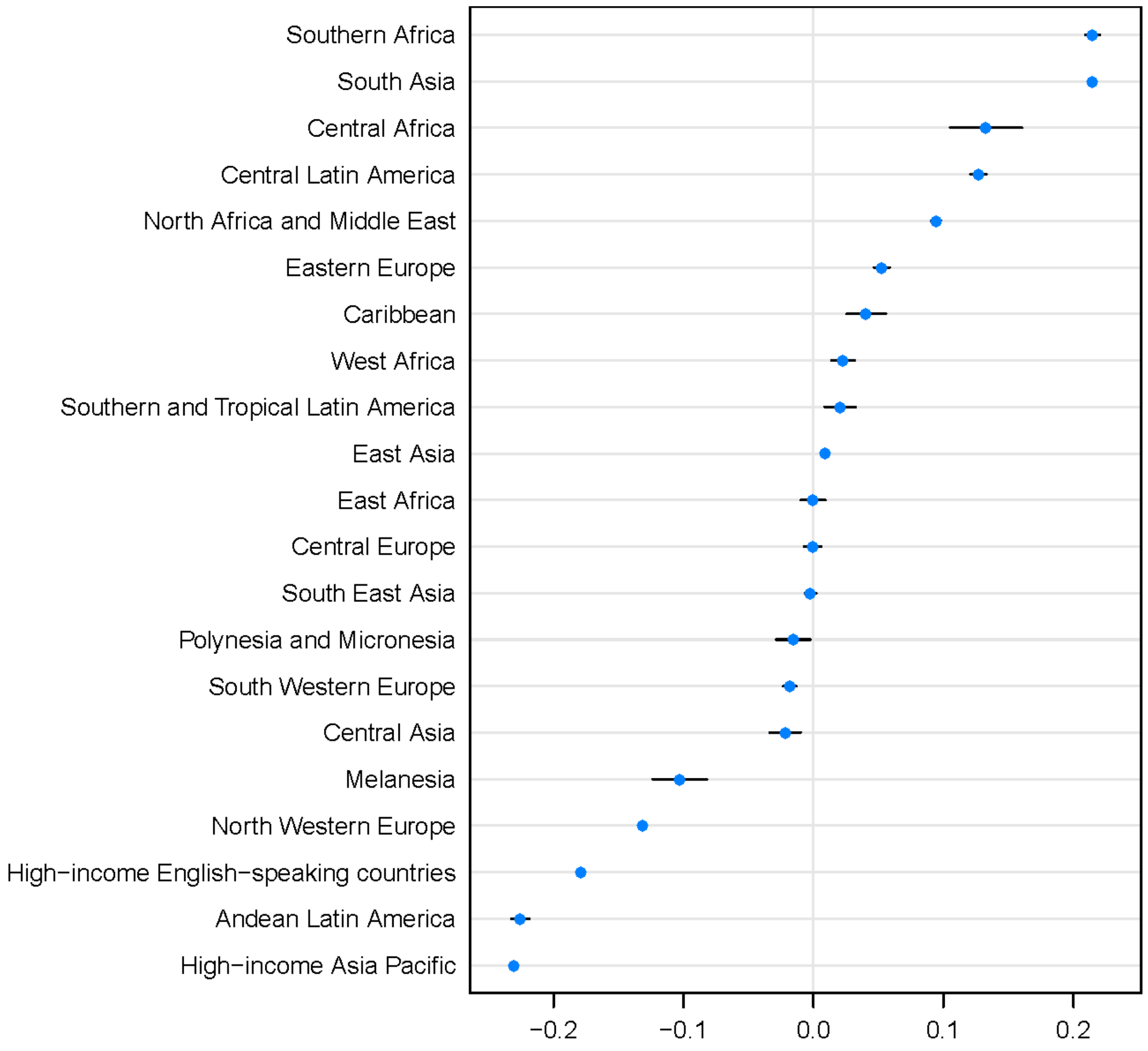
Southern Africa
South Asia
Central Africa
Central Latin America
North Africa and Middle East
Eastern Europe
Caribbean
West Africa
Southern and Tropical Latin America
East Asia
East Africa
Central Europe
South East Asia
Polynesia and Micronesia
South Western Europe
Central Asia
Melanesia
North Western Europe
High−income English−speaking countries
Andean Latin America
High−income Asia Pacific
−0.2
−0.1
0.0
0.1
0.2

**Primary outcome: prevalence of raised blood pressure (SBP ≥140 mmHg or DBP ≥90 mmHg)**	
**Independent variables: mean SBP and mean DBP**	
**Variables**	**Coefficients (95% CI)**
Intercept	-0.33 (-0.36, -0.29)
Mean SBP (centred at 130 mmHg)	0.042 (0.042, 0.042)
Mean DBP (centred at 80 mmHg)	0.024 (0.024, 0.025)
Mean age of age group (centred at 50 years)	0.0040 (0.0039, 0.0041)
Male sex	-0.0097 (-0.012, -0.0078)
Study mid-year (per one more recent year since 1975)	-0.0034 (-0.0035, -0.0032)
Natural logarithm of per-capita gross domestic product	-0.0023 (-0.0047, 0.00014)
Mean SBP * mean age of age group	-0.00018 (-0.00018, -0.00017)
Mean DBP * mean age of age group	-0.00026 (-0.00027, -0.00025)
Mean SBP * male sex	0.0045 (0.0042, 0.0048)
Mean DBP * male sex	-0.0071 (-0.0076, -0.0066)
Random effects for regions	Yes
**Number of data points used to fit the model = 10,965**	

Traditional R^2 is not clearly defined for mixed-effect models. The pseudo-R^2 for the model, which describes the proportion of variance explained by both fixed and random factors,[12] was 0.929.

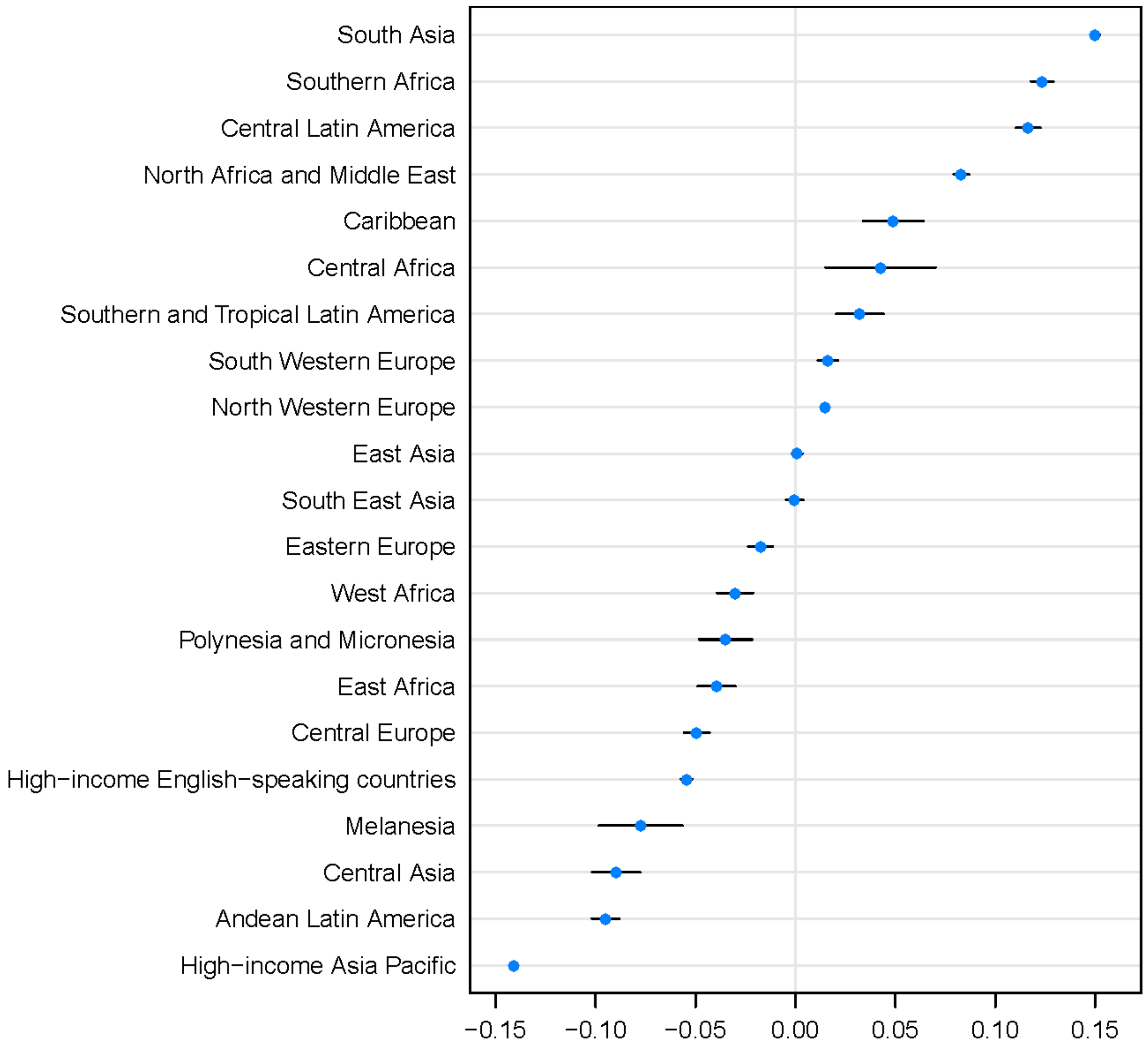
South Asia
Southern Africa
Central Latin America
North Africa and Middle East
Caribbean
Central Africa
Southern and Tropical Latin America
South Western Europe
North Western Europe
East Asia
South East Asia
Eastern Europe
West Africa
Polynesia and Micronesia
East Africa
Central Europe
High−income English−speaking countries
Melanesia
Central Asia
Andean Latin America
High−income Asia Pacific
−0.15
−0.10
−0.05
0.00
0.05
0.10
0.15

Primary outcome: prevalence of raised blood pressure (SBP ≥140 mmHg or DBP ≥90 mmHg)	
**Independent variable: prevalence (SBP ≥140 mmHg or DBP ≥90 mmHg or use of antihypertensive medication)**	
**Variables**	**Coefficients (95% CI)**
Intercept	0.10 (0.062, 0.14)
Probit-transformed prevalence (SBP ≥140 mmHg or DBP ≥90 mmHg or use of antihypertensive medication)	0.97 (0.97, 0.98)
Mean age of age group (centred at 50 years)	-0.0063 (-0.0065, -0.0061)
Male sex	0.050 (0.048, 0.053)
Study mid-year (per one more recent year since 1975)	-0.0070 (-0.0072, -0.0069)
Natural logarithm of per-capita gross domestic product	-0.0085 (-0.011, -0.0057)
Probit-transformed prevalence * mean age of age group	-0.0018 (-0.0019, -0.0017)
Probit-transformed prevalence * male sex	0.031 (0.027, 0.035)
Random effects for regions	Yes
**Number of data points used to fit the model = 8,651**	

Traditional R^2 is not clearly defined for mixed-effect models. The pseudo-R^2 for the model, which describes the proportion of variance explained by both fixed and random factors,[12] was 0.899.

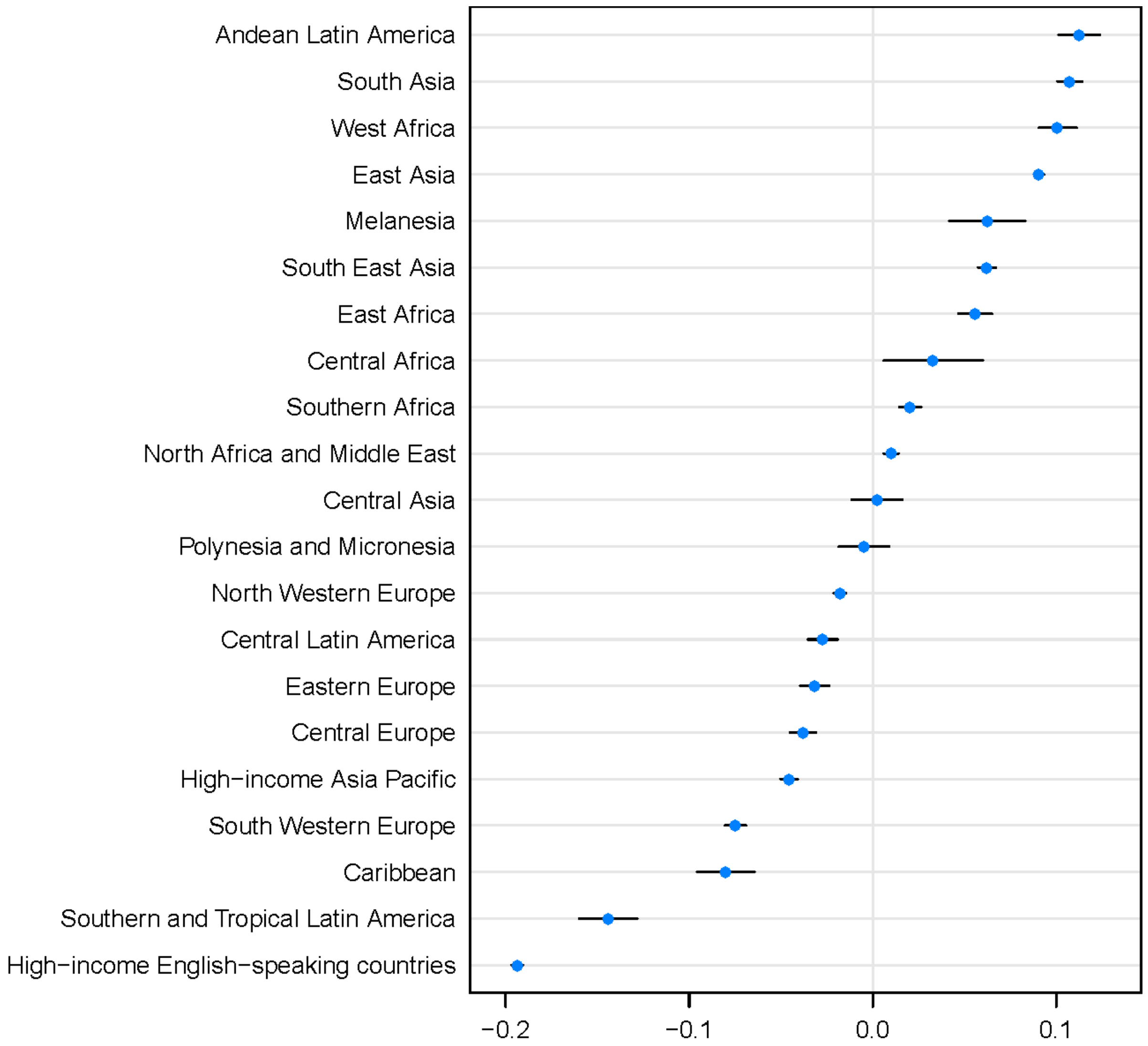
Andean Latin America
South Asia
West Africa
East Asia
Melanesia
South East Asia
East Africa
Central Africa
Southern Africa
North Africa and Middle East
Central Asia
Polynesia and Micronesia
North Western Europe
Central Latin America
Eastern Europe
Central Europe
High−income Asia Pacific
South Western Europe
Caribbean
Southern and Tropical Latin America
High−income English−speaking countries
−0.2
−0.1
0.0
0.1

**Primary outcome: prevalence of raised blood pressure (SBP ≥140 mmHg or DBP ≥90 mmHg)**	
**Independent variable: prevalence (SBP ≥160 mmHg or DBP ≥95 mmHg or use of antihypertensive medication)**	
**Variables**	**Coefficients (95% CI)**
Intercept	0.027 (-0.031, 0.085)
Probit-transformed prevalence (SBP ≥160 mmHg or DBP ≥95 mmHg or use of antihypertensive medication)	0.68 (0.68, 0.69)
Mean age of age group (centred at 50 years)	0.0036 (0.0034, 0.0038)
Male sex	0.068 (0.064, 0.072)
Study mid-year (per one more recent year since 1975)	-0.012 (-0.012, -0.012)
Natural logarithm of per-capita gross domestic product	0.039 (0.036, 0.042)
Probit-transformed prevalence * mean age of age group	-0.0029 (-0.0030, -0.0028)
Probit-transformed prevalence * male sex	-0.079 (-0.083, -0.076)
Random effects for regions	Yes
**Number of data points used to fit the model = 8,681**	

Traditional R^2 is not clearly defined for mixed-effect models. The pseudo-R^2 for the model, which describes the proportion of variance explained by both fixed and random factors,[12] was 0.811.

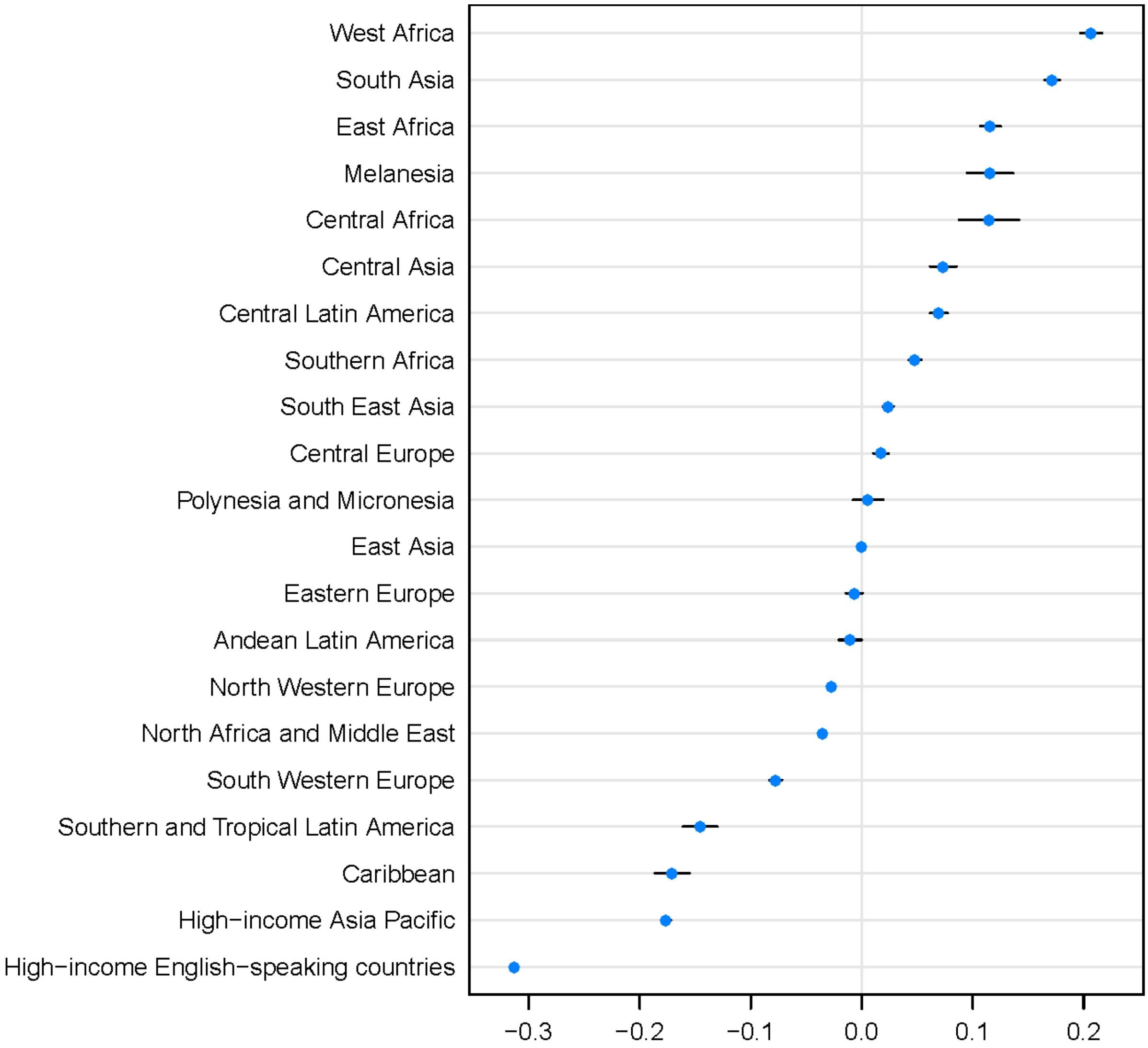
West Africa
South Asia
East Africa
Melanesia
Central Africa
Central Asia
Central Latin America
Southern Africa
South East Asia
Central Europe
Polynesia and Micronesia
East Asia
Eastern Europe
Andean Latin America
North Western Europe
North Africa and Middle East
South Western Europe
Southern and Tropical Latin America
Caribbean
High−income Asia Pacific
High−income English−speaking countries
−0.3
−0.2
−0.1
0.0
0.1
0.2

Primary outcome: prevalence of raised blood pressure (SBP ≥140 mmHg or DBP ≥90 mmHg)	
**Independent variable: prevalence (SBP ≥130 mmHg or DBP ≥85 mmHg)**	
**Variables**	**Coefficients (95% CI)**
Intercept	-0.27 (-0.31, -0.23)
Probit-transformed prevalence (SBP ≥130 mmHg or DBP ≥85 mmHg)	0.89 (0.89, 0.90)
Mean age of age group (centred at 50 years)	0.0040 (0.0039, 0.0042)
Male sex	-0.039 (-0.041, -0.036)
Study mid-year (per one more recent year since 1975)	-0.0033 (-0.0035, -0.0030)
Natural logarithm of per-capita gross domestic product	-0.012 (-0.016, -0.0085)
Probit-transformed prevalence * mean age of age group	-0.00048 (-0.00063, -0.00033)
Probit-transformed prevalence * male sex	0.029 (0.024, 0.034)
Random effects for regions	Yes
**Number of data points used to fit the model = 7,463**	

Traditional R^2 is not clearly defined for mixed-effect models. The pseudo-R^2 for the model, which describes the proportion of variance explained by both fixed and random factors,[12] was 0.930.

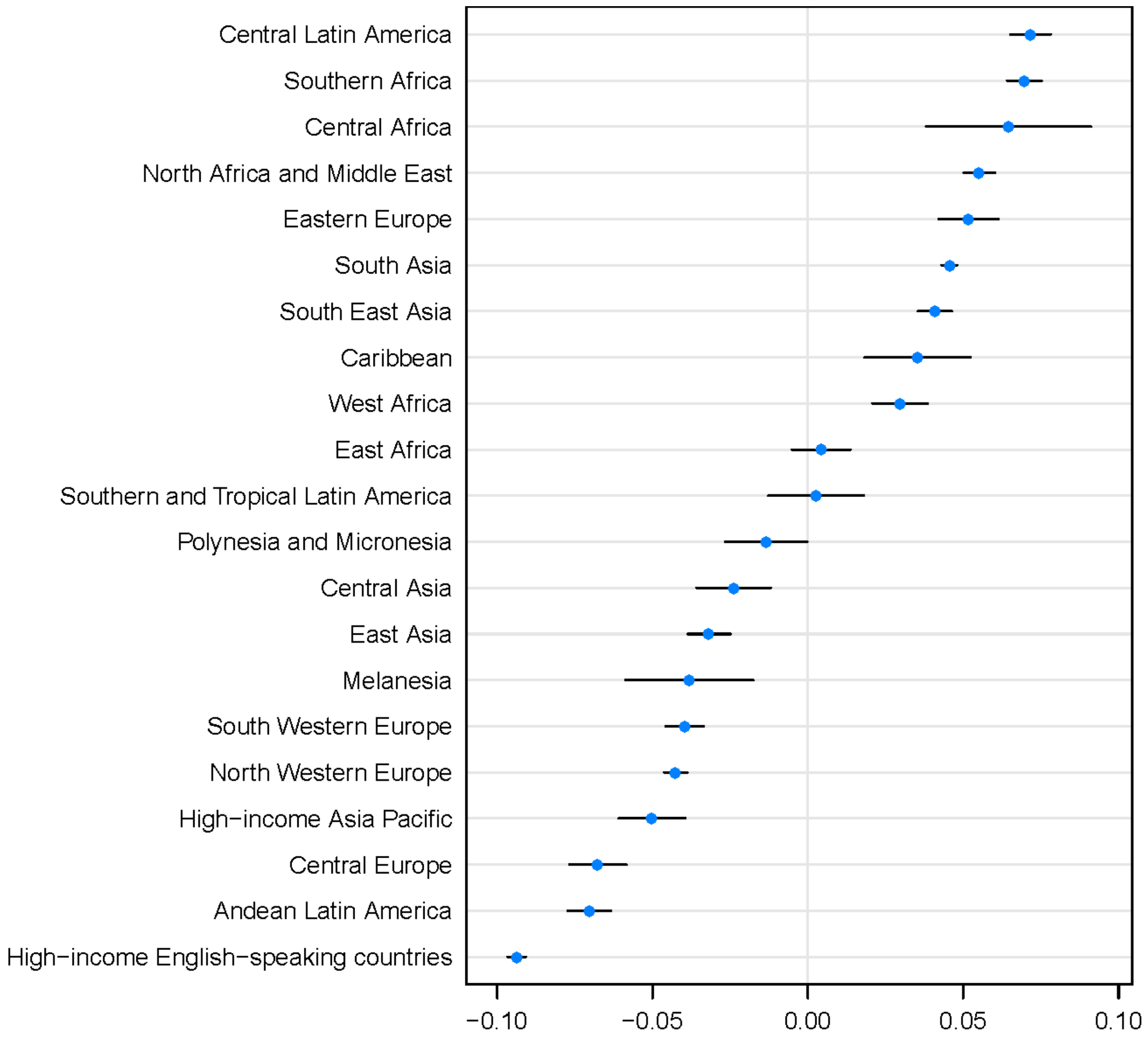

Central Latin America
Southern Africa
Central Africa
North Africa and Middle East
Eastern Europe
South Asia
South East Asia
Caribbean
West Africa
East Africa
Southern and Tropical Latin America
Polynesia and Micronesia
Central Asia
East Asia
Melanesia
South Western Europe
North Western Europe
High−income Asia Pacific
Central Europe
Andean Latin America
High−income English−speaking countries
−0.10
−0.05
0.00
0.05
0.10

Primary outcome: prevalence of raised blood pressure (SBP ≥140 mmHg or DBP ≥90 mmHg)	
**Independent variable: prevalence (SBP ≥150 mmHg or DBP ≥90 mmHg)**	
**Variables**	**Coefficients (95% CI)**
Intercept	0.085 (0.046, 0.12)
Probit-transformed prevalence (SBP ≥150 mmHg or DBP ≥90 mmHg)	1.01 (1.00, 1.01)
Mean age of age group (centred at 50 years)	0.0060 (0.0058, 0.0062)
Male sex	-0.034 (-0.039, -0.030)
Study mid-year (per one more recent year since 1975)	0.0014 (0.0012, 0.0016)
Natural logarithm of per-capita gross domestic product	0.0068 (0.0032, 0.010)
Probit-transformed prevalence * mean age of age group	0.0020 (0.0019, 0.0022)
Probit-transformed prevalence * male sex	-0.088 (-0.094, -0.083)
Random effects for regions	Yes
**Number of data points used to fit the model = 7,412**	

Traditional R^2 is not clearly defined for mixed-effect models. The pseudo-R^2 for the model, which describes the proportion of variance explained by both fixed and random factors,[12] was 0.932.

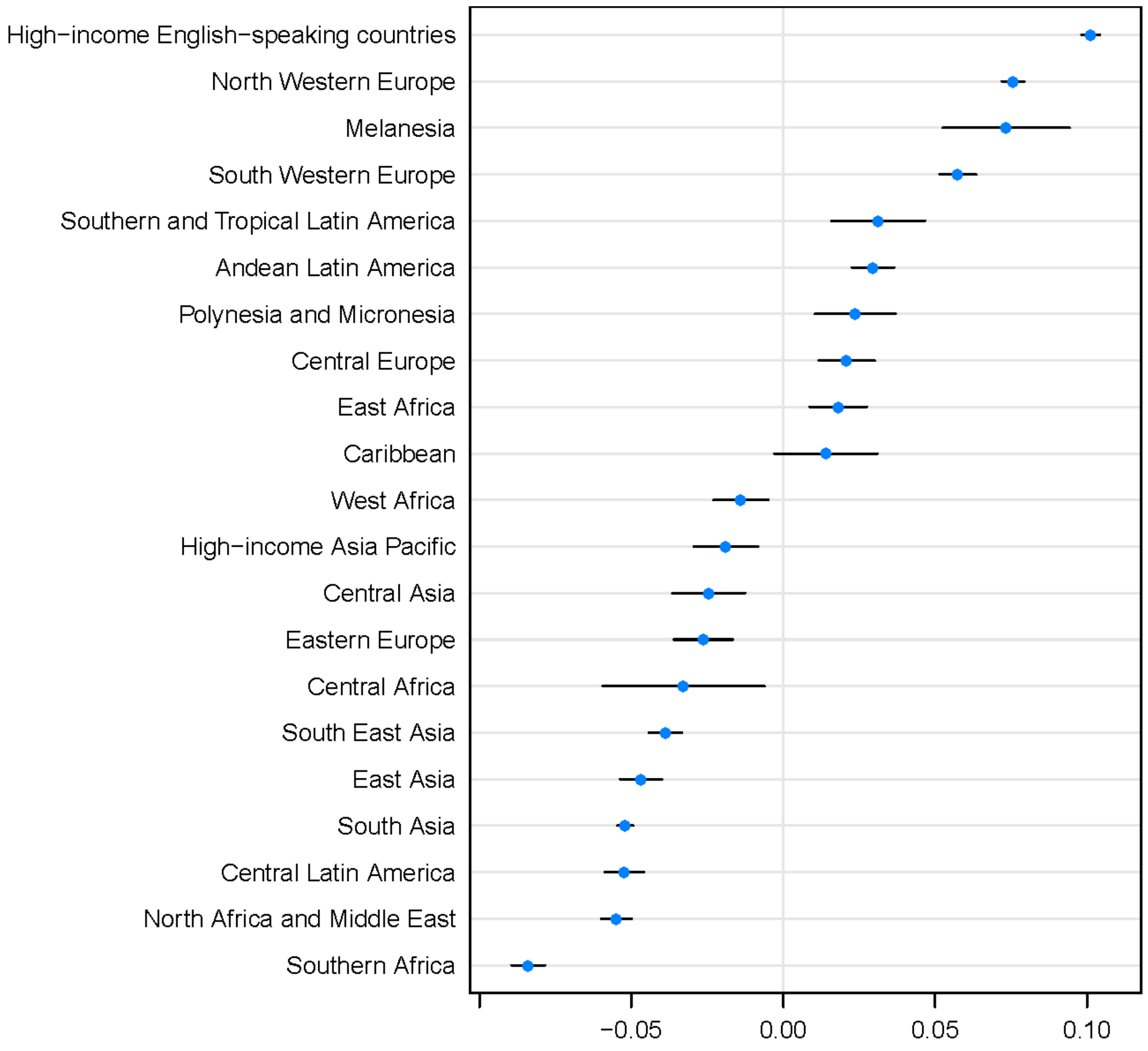
High−income English−speaking countries
North Western Europe
Melanesia
South Western Europe
Southern and Tropical Latin America
Andean Latin America
Polynesia and Micronesia
Central Europe
East Africa
Caribbean
West Africa
High−income Asia Pacific
Central Asia
Eastern Europe
Central Africa
South East Asia
East Asia
South Asia
Central Latin America
North Africa and Middle East
Southern Africa
−0.05
0.00
0.05
0.10

**Primary outcome: prevalence of raised blood pressure (SBP ≥140 mmHg or DBP ≥90 mmHg)**	
**Independent variable: prevalence (SBP ≥160 mmHg or DBP ≥90 mmHg)**	
**Variables**	**Coefficients (95% CI)**
Intercept	0.059 (0.016, 0.10)
Probit-transformed prevalence (SBP ≥160 mmHg or DBP ≥90 mmHg)	0.93 (0.93, 0.94)
Mean age of age group (centred at 50 years)	0.012 (0.012, 0.012)
Male sex	-0.078 (-0.083, -0.072)
Study mid-year (per one more recent year since 1975)	0.0011 (0.00090, 0.0014)
Natural logarithm of per-capita gross domestic product	0.014 (0.011, 0.018)
Probit-transformed prevalence * mean age of age group	0.0025 (0.0023, 0.0026)
Probit-transformed prevalence * male sex	-0.15 (-0.15, -0.14)
Random effects for regions	Yes
**Number of data points used to fit the model = 7,389**	

Traditional R^2 is not clearly defined for mixed-effect models. The pseudo-R^2 for the model, which describes the proportion of variance explained by both fixed and random factors,[12] was 0.905.

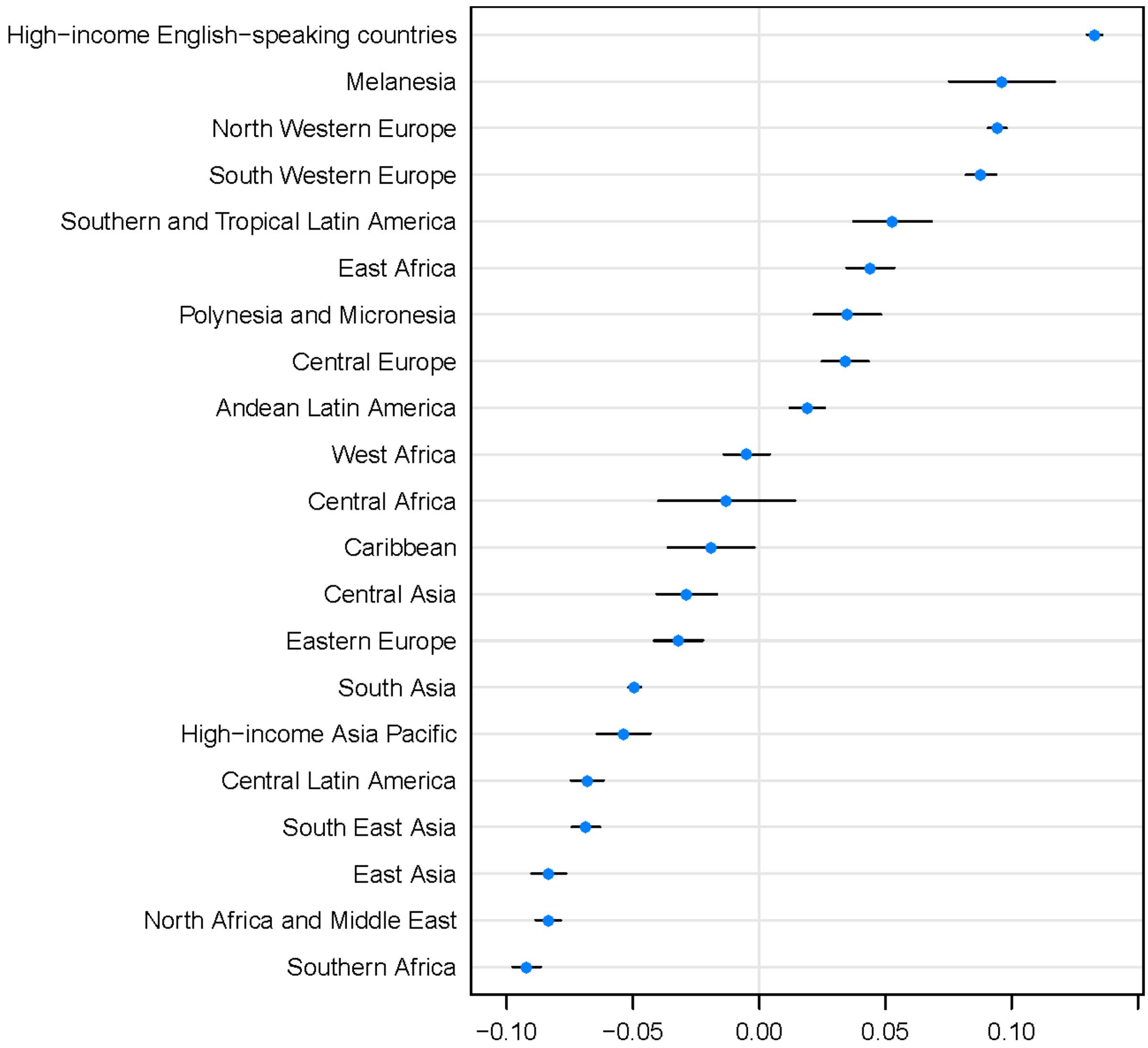
High−income English−speaking countries
Melanesia
North Western Europe
South Western Europe
Southern and Tropical Latin America
East Africa
Polynesia and Micronesia
Central Europe
Andean Latin America
West Africa
Central Africa
Caribbean
Central Asia
Eastern Europe
South Asia
High−income Asia Pacific
Central Latin America
South East Asia
East Asia
North Africa and Middle East
Southern Africa
−0.10
−0.05
0.00
0.05
0.10

Primary outcome: prevalence of raised blood pressure (SBP ≥140 mmHg or DBP ≥90 mmHg)	
**Independent variable: prevalence (SBP ≥160 mmHg or DBP ≥95 mmHg)**	
**Variables**	**Coefficients (95% CI)**
Intercept	-0.29 (-0.33, -0.26)
Probit-transformed prevalence (SBP ≥160 mmHg or DBP ≥95 mmHg)	0.96 (0.95, 0.96)
Mean age of age group (centred at 50 years)	0.0094 (0.0092, 0.0096)
Male sex	-0.048 (-0.054, -0.043)
Study mid-year (per one more recent year since 1975)	0.0020 (0.0018, 0.0022)
Natural logarithm of per-capita gross domestic product	0.086 (0.083, 0.089)
Probit-transformed prevalence * mean age of age group	0.0039 (0.0038, 0.0040)
Probit-transformed prevalence * male sex	-0.081 (-0.085, -0.077)
Random effects for regions	Yes
**Number of data points used to fit the model = 7,363**	

Traditional R^2 is not clearly defined for mixed-effect models. The pseudo-R^2 for the model, which describes the proportion of variance explained by both fixed and random factors,[12] was 0.955.

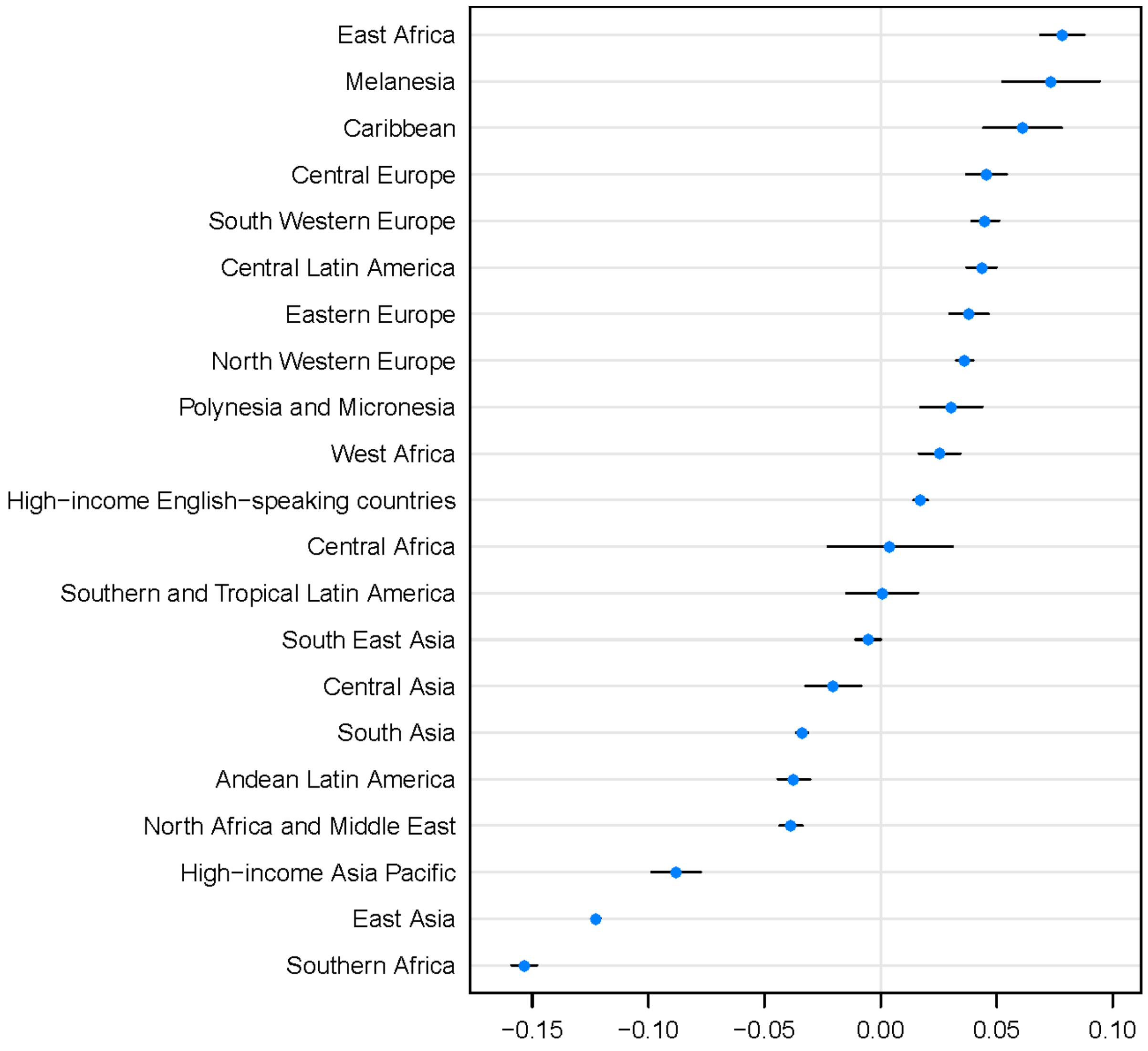
East Africa
Melanesia
Caribbean
Central Europe
South Western Europe
Central Latin America
Eastern Europe
North Western Europe
Polynesia and Micronesia
West Africa
High−income English−speaking countries
Central Africa
Southern and Tropical Latin America
South East Asia
Central Asia
South Asia
Andean Latin America
North Africa and Middle East
High−income Asia Pacific
East Asia
Southern Africa
−0.15
−0.10
−0.05
0.00
0.05
0.10

**Primary outcome: prevalence of raised blood pressure (SBP ≥140 mmHg or DBP ≥90 mmHg)**	
**Independent variable: prevalence (SBP ≥130 mmHg or DBP ≥85 mmHg or use of antihypertensive medication)**	
**Variables**	**Coefficients (95% CI)**
Intercept	0.033 (-0.017, 0.083)
Probit-transformed prevalence (SBP ≥130 mmHg or DBP ≥85 mmHg or use of antihypertensive medication)	0.85 (0.84, 0.85)
Mean age of age group (centred at 50 years)	0.00065 (0.00042, 0.00087)
Male sex	-0.0071 (-0.011, -0.0033)
Study mid-year (per one more recent year since 1975)	-0.0075 (-0.0078, -0.0073)
Natural logarithm of per-capita gross domestic product	-0.043 (-0.047, -0.039)
Probit-transformed prevalence * mean age of age group	-0.0020 (-0.0022, -0.0018)
Probit-transformed prevalence * male sex	0.076 (0.070, 0.082)
Random effects for regions	Yes
**Number of data points used to fit the model = 6,260**	

Traditional R^2 is not clearly defined for mixed-effect models. The pseudo-R^2 for the model, which describes the proportion of variance explained by both fixed and random factors,[12] was 0.867.

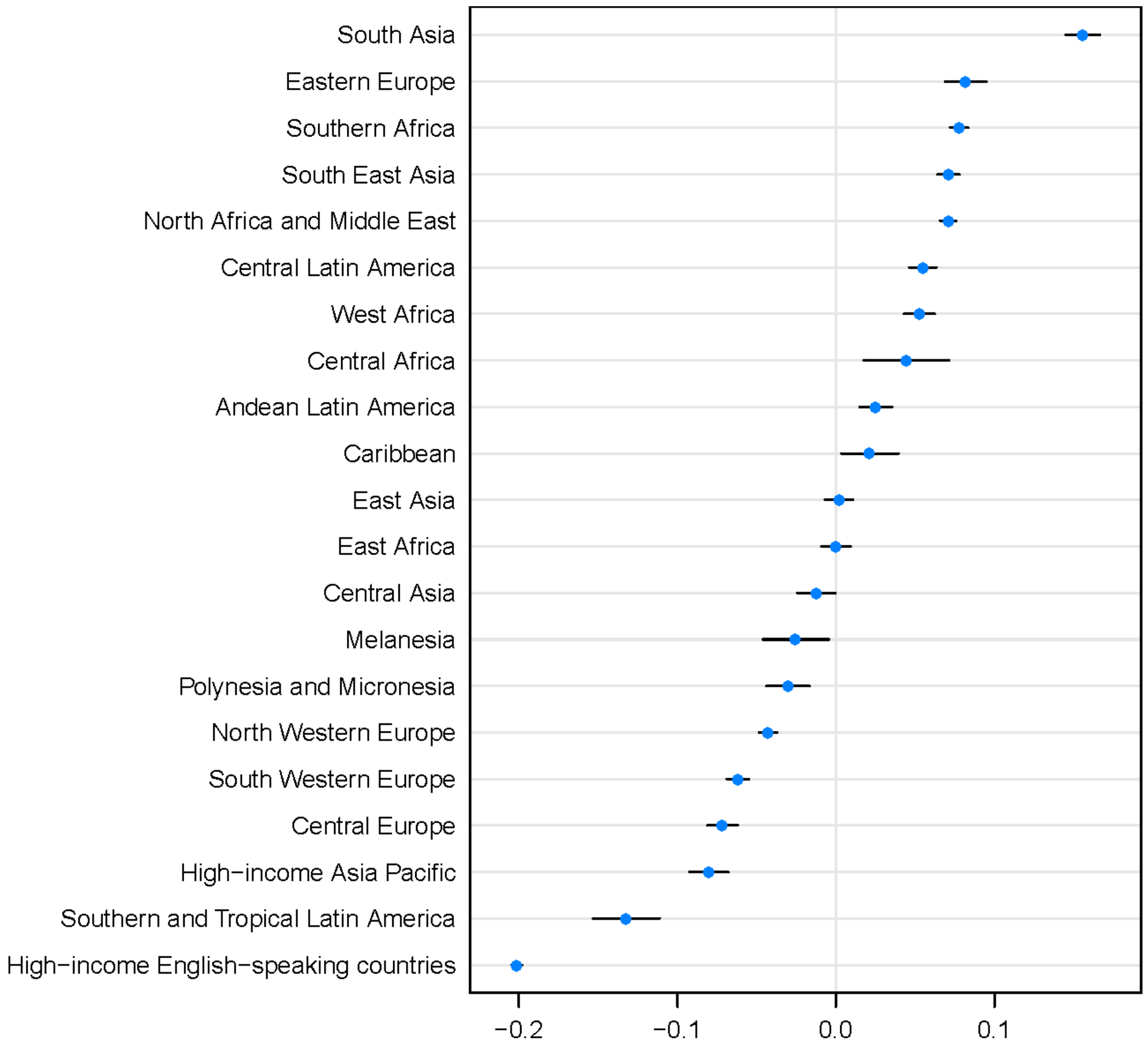
South Asia
Eastern Europe
Southern Africa
South East Asia
North Africa and Middle East
Central Latin America
West Africa
Central Africa
Andean Latin America
Caribbean
East Asia
East Africa
Central Asia
Melanesia
Polynesia and Micronesia
North Western Europe
South Western Europe
Central Europe
High−income Asia Pacific
Southern and Tropical Latin America
High−income English−speaking countries
−0.2
−0.1
0.0
0.1

Primary outcome: prevalence of raised blood pressure (SBP ≥140 mmHg or DBP ≥90 mmHg)	
**Independent variable: prevalence (SBP ≥160 mmHg or DBP ≥100 mmHg or use of antihypertensive medication)**	
**Variables**	**Coefficients (95% CI)**
Intercept	0.94 (0.88, 1.01)
Probit-transformed prevalence (SBP ≥160 mmHg or DBP ≥100 mmHg or use of antihypertensive medication)	0.52 (0.52, 0.53)
Mean age of age group (centred at 50 years)	0.0073 (0.0070, 0.0075)
Male sex	0.063 (0.058, 0.069)
Study mid-year (per one more recent year since 1975)	-0.011 (-0.011, -0.011)
Natural logarithm of per-capita gross domestic product	-0.077 (-0.081, -0.073)
Probit-transformed prevalence * mean age of age group	-0.0034 (-0.0036, -0.0033)
Probit-transformed prevalence * male sex	-0.12 (-0.12, -0.11)
Random effects for regions	Yes
**Number of data points used to fit the model = 5,997**	

Traditional R^2 is not clearly defined for mixed-effect models. The pseudo-R^2 for the model, which describes the proportion of variance explained by both fixed and random factors,[12] was 0.760.

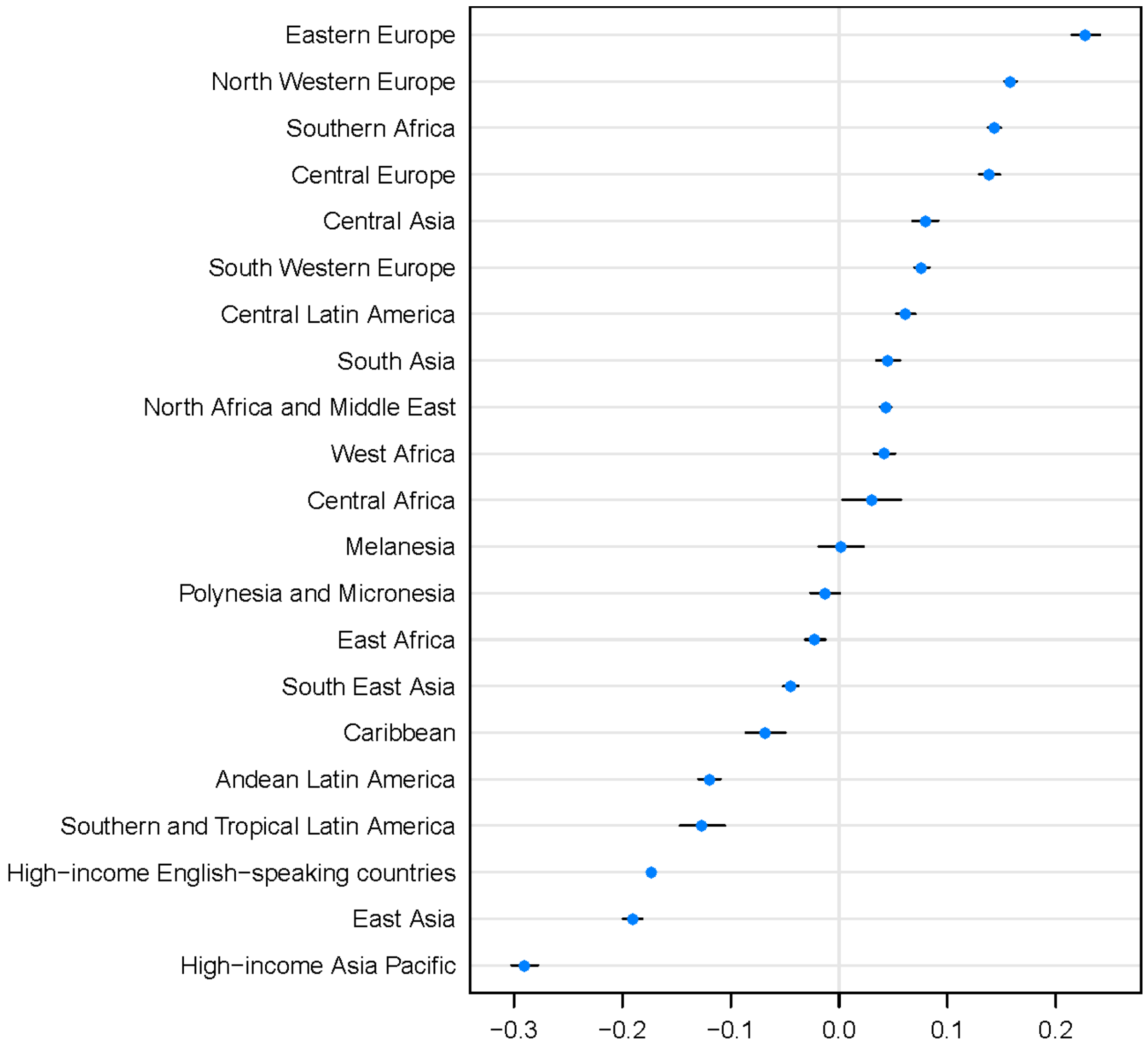
Eastern Europe
North Western Europe
Southern Africa
Central Europe
Central Asia
South Western Europe
Central Latin America
South Asia
North Africa and Middle East
West Africa
Central Africa
Melanesia
Polynesia and Micronesia
East Africa
South East Asia
Caribbean
Andean Latin America
Southern and Tropical Latin America
High−income English−speaking countries
East Asia
High−income Asia Pacific
−0.3
−0.2
−0.1
0.0
0.1
0.2

Primary outcome: prevalence of raised blood pressure (SBP ≥140 mmHg or DBP ≥90 mmHg)	
**Independent variables: mean SBP, mean DBP, and prevalence (SBP ≥140 mmHg or DBP ≥90 mmHg or use of antihypertensive medication)**	
**Variables**	**Coefficients (95% CI)**
Intercept	-0.081 (-0.11, -0.052)
Mean SBP (centred at 130 mmHg)	0.021 (0.021, 0.022)
Mean DBP (centred at 80 mmHg)	0.014 (0.014, 0.015)
Probit-transformed prevalence (SBP ≥140 mmHg or DBP ≥90 mmHg or use of antihypertensive medication)	0.50 (0.49, 0.51)
Mean age of age group (centred at 50 years)	-0.0051 (-0.0053, -0.0048)
Male sex	0.013 (0.0088, 0.017)
Study mid-year (per one more recent year since 1975)	-0.0035 (-0.0037, -0.0033)
Natural logarithm of per-capita gross domestic product	-0.014 (-0.017, -0.011)
Mean SBP * mean age of age group	0.00056 (0.00054, 0.00058)
Mean SBP * male sex	0.0012 (0.00053, 0.0018)
Mean DBP * mean age of age group	-0.00019 (-0.00021, -0.00016)
Mean DBP * male sex	0.0011 (0.00043, 0.0017)
Probit-transformed prevalence * mean age of age group	-0.0095 (-0.0099, -0.0092)
Probit-transformed prevalence * male sex	-0.00038 (-0.010, 0.0096)
Random effects for regions	Yes
**Number of data points used to fit the model = 8,639**	

Traditional R^2 is not clearly defined for mixed-effect models. The pseudo-R^2 for the model, which describes the proportion of variance explained by both fixed and random factors,[12] was 0.935.

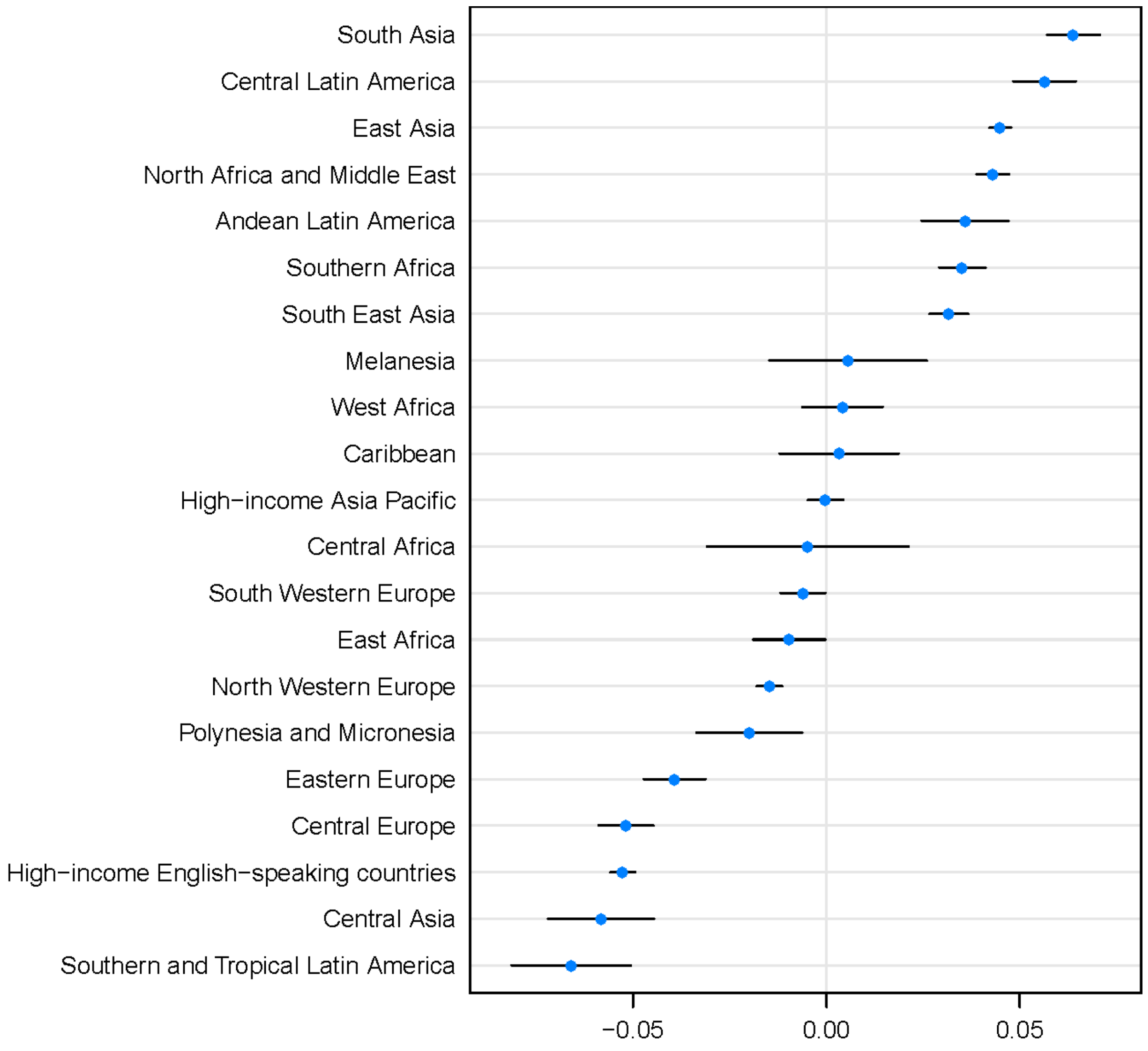
South Asia
Central Latin America
East Asia
North Africa and Middle East
Andean Latin America
Southern Africa
South East Asia
Melanesia
West Africa
Caribbean
High−income Asia Pacific
Central Africa
South Western Europe
East Africa
North Western Europe
Polynesia and Micronesia
Eastern Europe
Central Europe
High−income English−speaking countries
Central Asia
Southern and Tropical Latin America
−0.05
0.00
0.05

Primary outcome: prevalence of raised blood pressure (SBP ≥140 mmHg or DBP ≥90 mmHg)	
**Independent variables: mean SBP, mean DBP, and prevalence (SBP ≥160 mmHg or DBP ≥95 mmHg or use of antihypertensive medication)**	
**Variables**	**Coefficients (95% CI)**
Intercept	-0.17 (-0.20, -0.14)
Mean SBP (centred at 130 mmHg)	0.031 (0.031, 0.032)
Mean DBP (centred at 80 mmHg)	0.025 (0.024, 0.025)
Probit-transformed prevalence (SBP ≥160 mmHg or DBP ≥95 mmHg or use of antihypertensive medication)	0.19 (0.18, 0.19)
Mean age of age group (centred at 50 years)	-0.00090 (-0.0012, -0.00059)
Male sex	-0.044 (-0.050, -0.037)
Study mid-year (per one more recent year since 1975)	-0.0039 (-0.0041, -0.0037)
Natural logarithm of per-capita gross domestic product	-0.0040 (-0.0068, -0.0012)
Mean SBP * mean age of age group	0.00025 (0.00024, 0.00026)
Mean SBP * male sex	0.0047 (0.0042, 0.0052)
Mean DBP * mean age of age group	-0.00046 (-0.00048, -0.00044)
Mean DBP * male sex	0.00044 (-0.00020, 0.0011)
Probit-transformed prevalence * mean age of age group	-0.0041 (-0.0043, -0.0038)
Probit-transformed prevalence * male sex	-0.066 (-0.074, -0.059)
Random effects for regions	Yes
**Number of data points used to fit the model = 8,679**	

Traditional R^2 is not clearly defined for mixed-effect models. The pseudo-R^2 for the model, which describes the proportion of variance explained by both fixed and random factors,[12] was 0.922.

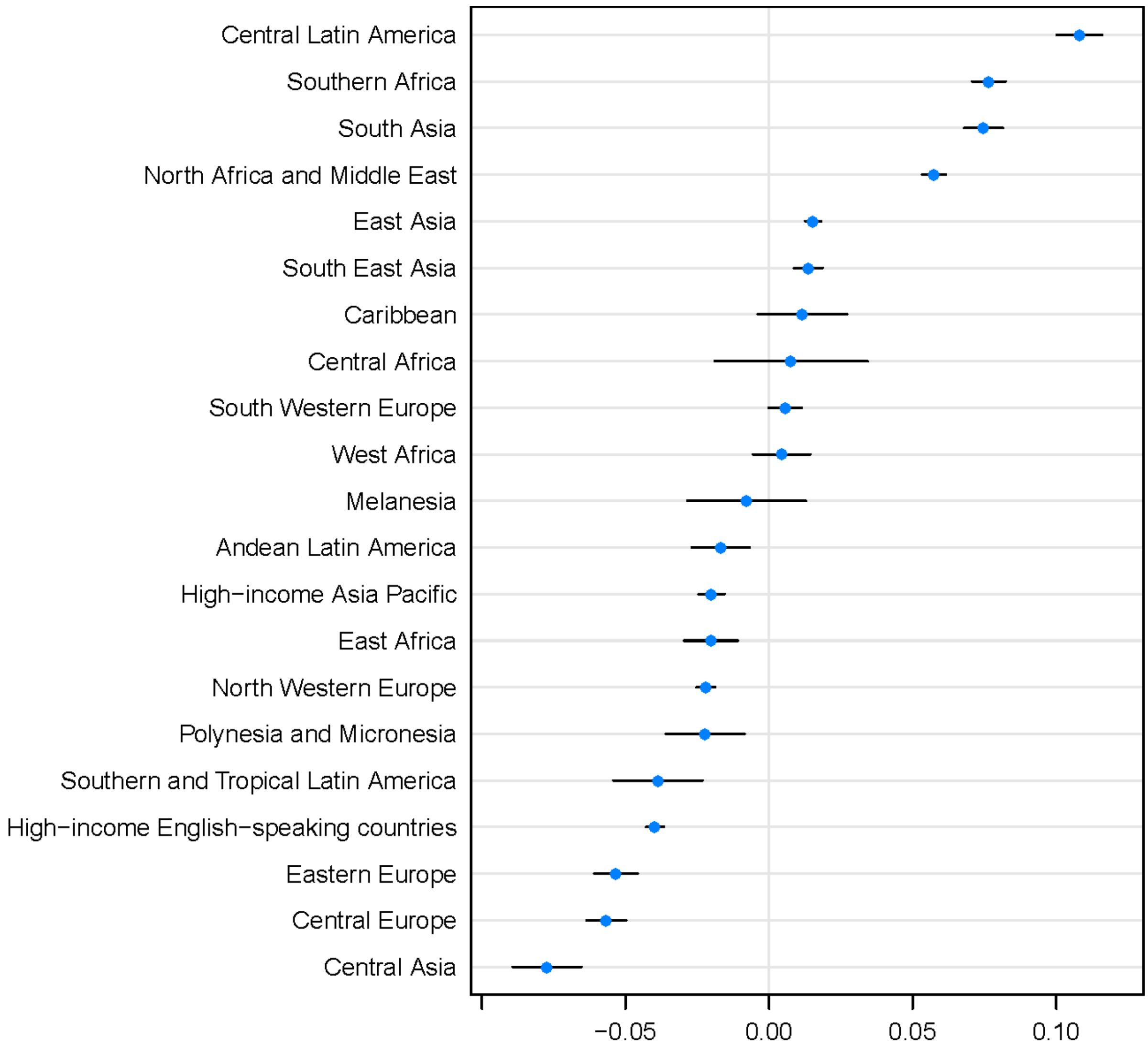
Central Latin America
Southern Africa
South Asia
North Africa and Middle East
East Asia
South East Asia
Caribbean
Central Africa
South Western Europe
West Africa
Melanesia
Andean Latin America
High−income Asia Pacific
East Africa
North Western Europe
Polynesia and Micronesia
Southern and Tropical Latin America
High−income English−speaking countries
Eastern Europe
Central Europe
Central Asia
−0.05
0.00
0.05
0.10

Primary outcome: prevalence of raised blood pressure (SBP ≥140 mmHg or DBP ≥90 mmHg)	
**Independent variables: mean SBP, mean DBP, and prevalence (SBP ≥160 mmHg or DBP ≥95 mmHg)**	
**Variables**	**Coefficients (95% CI)**
Intercept	0.16 (0.12, 0.20)
Mean SBP (centred at 130 mmHg)	0.025 (0.024, 0.025)
Mean DBP (centred at 80 mmHg)	0.0072 (0.0064, 0.0080)
Probit-transformed prevalence (SBP ≥160 mmHg or DBP ≥95 mmHg)	0.46 (0.45, 0.47)
Mean age of age group (centred at 50 years)	-0.0025 (-0.0029, -0.0021)
Male sex	-0.16 (-0.17, -0.14)
Study mid-year (per one more recent year since 1975)	-0.0025 (-0.0027, -0.0023)
Natural logarithm of per-capita gross domestic product	-0.0047 (-0.0083, -0.0011)
Mean SBP * mean age of age group	0.00016 (0.00014, 0.00017)
Mean SBP * male sex	0.0056 (0.0051, 0.0061)
Mean DBP * mean age of age group	-0.00014 (-0.00017, -0.00012)
Mean DBP * male sex	0.0058 (0.0049, 0.0067)
Probit-transformed prevalence * mean age of age group	-0.0046 (-0.0050, -0.0042)
Probit-transformed prevalence * male sex	-0.16 (-0.17, -0.14)
Random effects for regions	Yes
**Number of data points used to fit the model = 7,329**	

Traditional R^2 is not clearly defined for mixed-effect models. The pseudo-R^2 for the model, which describes the proportion of variance explained by both fixed and random factors,[12] was 0.929.

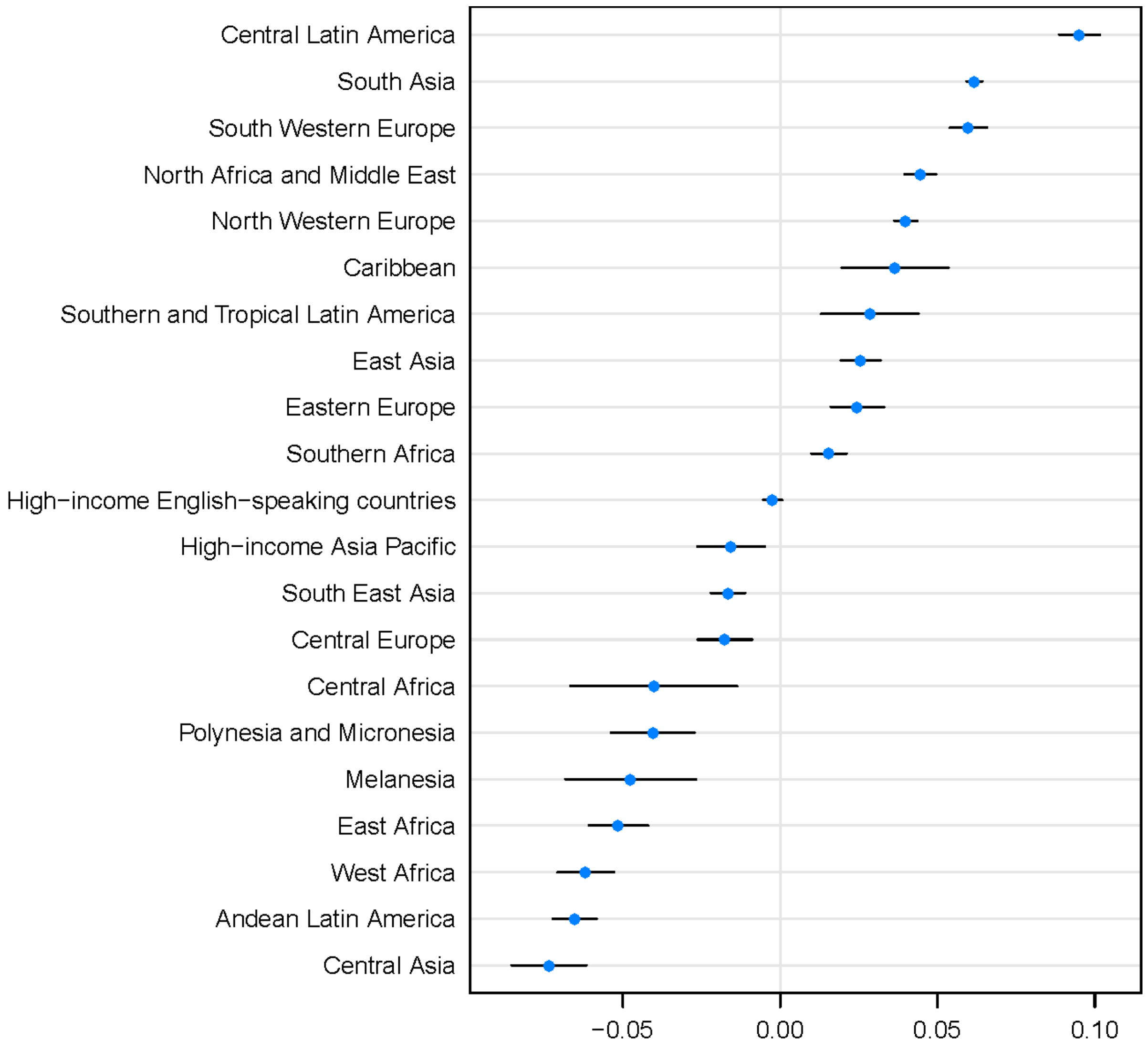
Central Latin America
South Asia
South Western Europe
North Africa and Middle East
North Western Europe
Caribbean
Southern and Tropical Latin America
East Asia
Eastern Europe
Southern Africa
High−income English−speaking countries
High−income Asia Pacific
South East Asia
Central Europe
Central Africa
Polynesia and Micronesia
Melanesia
East Africa
West Africa
Andean Latin America
Central Asia
−0.05
0.00
0.05
0.10

Primary outcome: prevalence of raised blood pressure (SBP ≥140 mmHg or DBP ≥90 mmHg)	
**Independent variables: mean SBP, mean DBP, and prevalence (SBP ≥140 mmHg)**	
**Variables**	**Coefficients (95% CI)**
Intercept	0.18 (0.16, 0.21)
Mean SBP (centred at 130 mmHg)	-0.0081 (-0.0088, -0.0075)
Mean DBP (centred at 80 mmHg)	0.021 (0.020, 0.021)
Probit-transformed prevalence (SBP ≥140 mmHg)	0.91 (0.90, 0.92)
Mean age of age group (centred at 50 years)	-0.0012 (-0.0014, -0.00088)
Male sex	0.078 (0.069, 0.086)
Study mid-year (per one more recent year since 1975)	-0.0010 (-0.0012, -0.00092)
Natural logarithm of per-capita gross domestic product	-0.0038 (-0.0062, -0.0013)
Mean SBP * mean age of age group	0.00027 (0.00025, 0.00030)
Mean SBP * male sex	-0.0059 (-0.0067, -0.0050)
Mean DBP * mean age of age group	-0.00017 (-0.00018, -0.00016)
Mean DBP * male sex	-0.0025 (-0.0031, -0.0020)
Probit-transformed prevalence * mean age of age group	-4.6e-06 (-0.00038, 0.00037)
Probit-transformed prevalence * male sex	0.070 (0.057, 0.083)
Random effects for regions	Yes
**Number of data points used to fit the model = 10,453**	

Traditional R^2 is not clearly defined for mixed-effect models. The pseudo-R^2 for the model, which describes the proportion of variance explained by both fixed and random factors,[12] was 0.956.

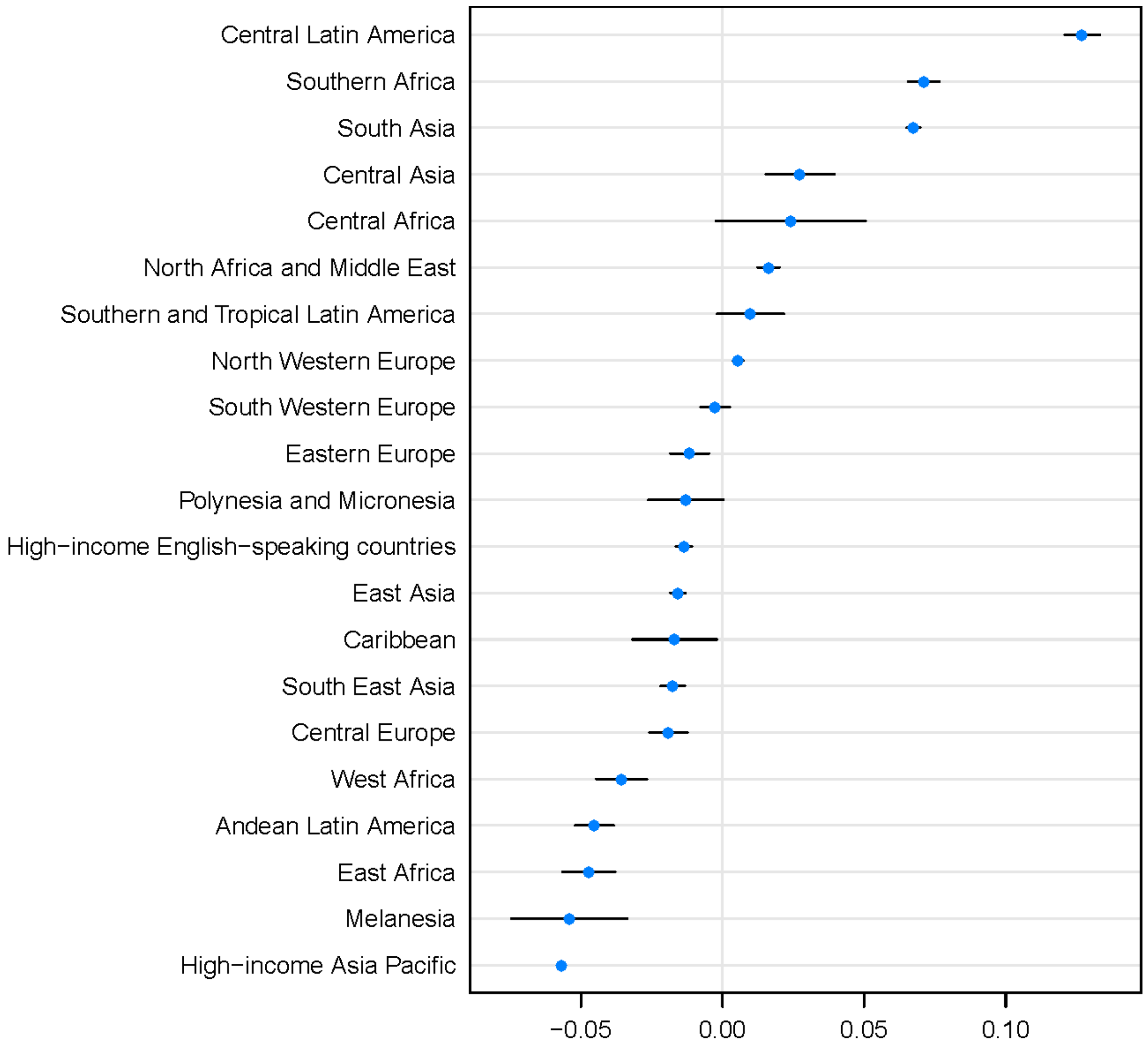
Central Latin America
Southern Africa
South Asia
Central Asia
Central Africa
North Africa and Middle East
Southern and Tropical Latin America
North Western Europe
South Western Europe
Eastern Europe
Polynesia and Micronesia
High−income English−speaking countries
East Asia
Caribbean
South East Asia
Central Europe
West Africa
Andean Latin America
East Africa
Melanesia
High−income Asia Pacific
−0.05
0.00
0.05
0.10

Primary outcome: prevalence of raised blood pressure (SBP ≥140 mmHg or DBP ≥90 mmHg)	
**Independent variables: mean SBP, mean DBP, and prevalence (SBP ≥160 mmHg)**	
**Variables**	**Coefficients (95% CI)**
Intercept	0.013 (-0.028, 0.054)
Mean SBP (centred at 130 mmHg)	0.020 (0.019, 0.021)
Mean DBP (centred at 80 mmHg)	0.027 (0.026, 0.027)
Probit-transformed prevalence (SBP ≥160 mmHg)	0.35 (0.34, 0.36)
Mean age of age group (centred at 50 years)	-0.00084 (-0.0015, -0.00016)
Male sex	-0.13 (-0.15, -0.11)
Study mid-year (per one more recent year since 1975)	-0.0020 (-0.0022, -0.0017)
Natural logarithm of per-capita gross domestic product	0.0090 (0.0054, 0.013)
Mean SBP * mean age of age group	0.00028 (0.00025, 0.00030)
Mean SBP * male sex	0.0058 (0.0050, 0.0066)
Mean DBP * mean age of age group	-0.00060 (-0.00062, -0.00058)
Mean DBP * male sex	0.0018 (0.0011, 0.0024)
Probit-transformed prevalence * mean age of age group	-0.0012 (-0.0017, -0.00075)
Probit-transformed prevalence * male sex	-0.10 (-0.12, -0.091)
Random effects for regions	Yes
**Number of data points used to fit the model = 6,417**	

Traditional R^2 is not clearly defined for mixed-effect models. The pseudo-R^2 for the model, which describes the proportion of variance explained by both fixed and random factors,[12] was 0.922.

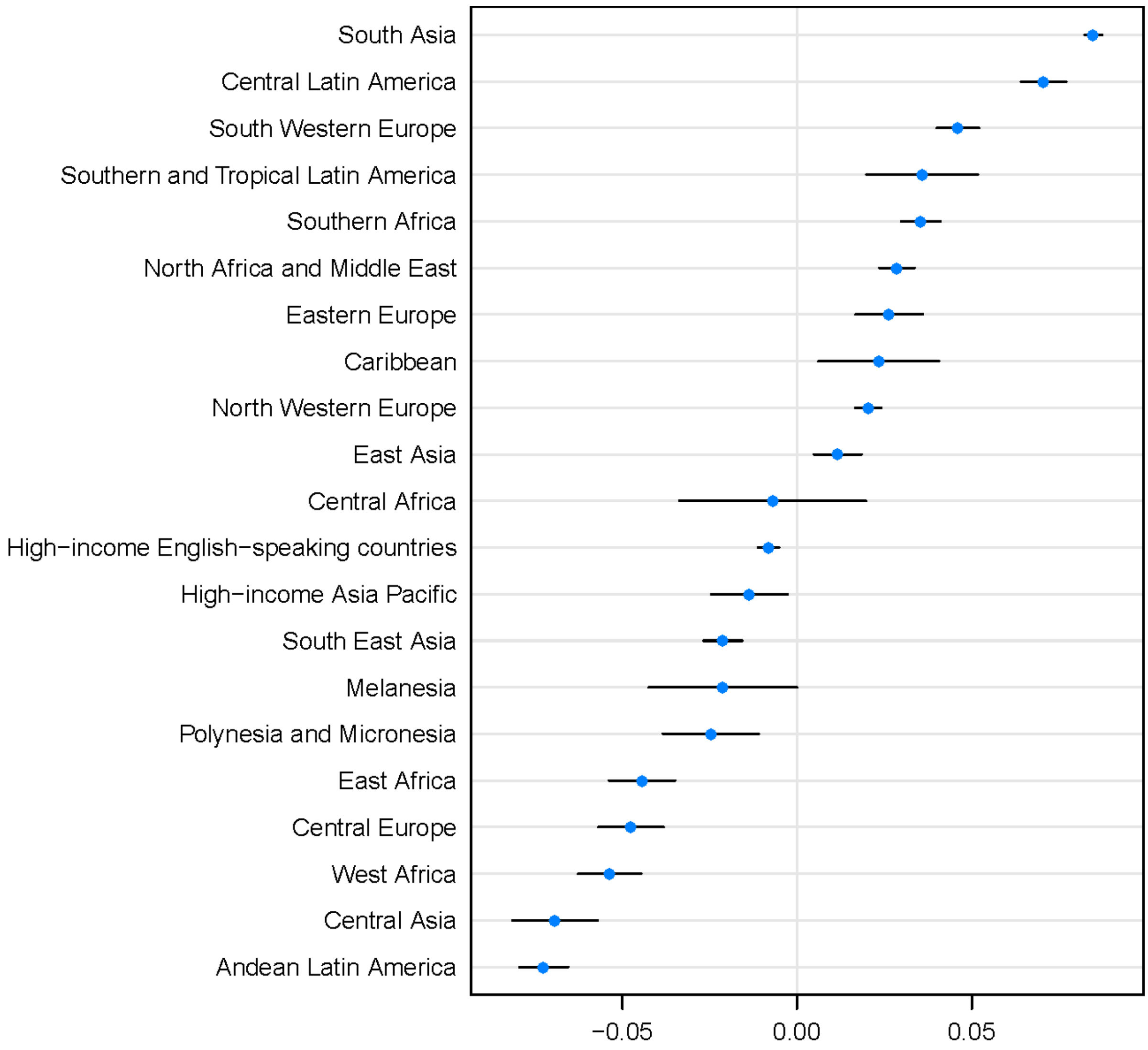
South Asia
Central Latin America
South Western Europe
Southern and Tropical Latin America
Southern Africa
North Africa and Middle East
Eastern Europe
Caribbean
North Western Europe
East Asia
Central Africa
High−income English−speaking countries
High−income Asia Pacific
South East Asia
Melanesia
Polynesia and Micronesia
East Africa
Central Europe
West Africa
Central Asia
Andean Latin America
−0.05
0.00
0.05

Primary outcome: prevalence of raised blood pressure (SBP ≥140 mmHg or DBP ≥90 mmHg)	
**Independent variables: mean SBP, mean DBP, and prevalence (SBP ≥135 mmHg or DBP ≥85 mmHg)**	
**Variables**	**Coefficients (95% CI)**
Intercept	-0.10 (-0.14, -0.065)
Mean SBP (centred at 130 mmHg)	0.0042 (0.0034, 0.0049)
Mean DBP (centred at 80 mmHg)	0.00097 (0.00015, 0.0018)
Probit-transformed prevalence (SBP ≥135 mmHg or DBP ≥85 mmHg)	0.85 (0.83, 0.87)
Mean age of age group (centred at 50 years)	0.0036 (0.0035, 0.0038)
Male sex	-0.018 (-0.022, -0.015)
Study mid-year (per one more recent year since 1975)	-0.0035 (-0.0037, -0.0033)
Natural logarithm of per-capita gross domestic product	-0.013 (-0.017, -0.0098)
Mean SBP * mean age of age group	0.00018 (0.00015, 0.00021)
Mean SBP * male sex	9.5e-05 (-0.00078, 0.00097)
Mean DBP * mean age of age group	7.5e-05 (4.9e-05, 0.00010)
Mean DBP * male sex	0.00058 (-0.00034, 0.0015)
Probit-transformed prevalence * mean age of age group	-0.0039 (-0.0045, -0.0033)
Probit-transformed prevalence * male sex	-0.0083 (-0.029, 0.012)
Random effects for regions	Yes
**Number of data points used to fit the model = 7,466**	

Traditional R^2 is not clearly defined for mixed-effect models. The pseudo-R^2 for the model, which describes the proportion of variance explained by both fixed and random factors,[12] was 0.935.

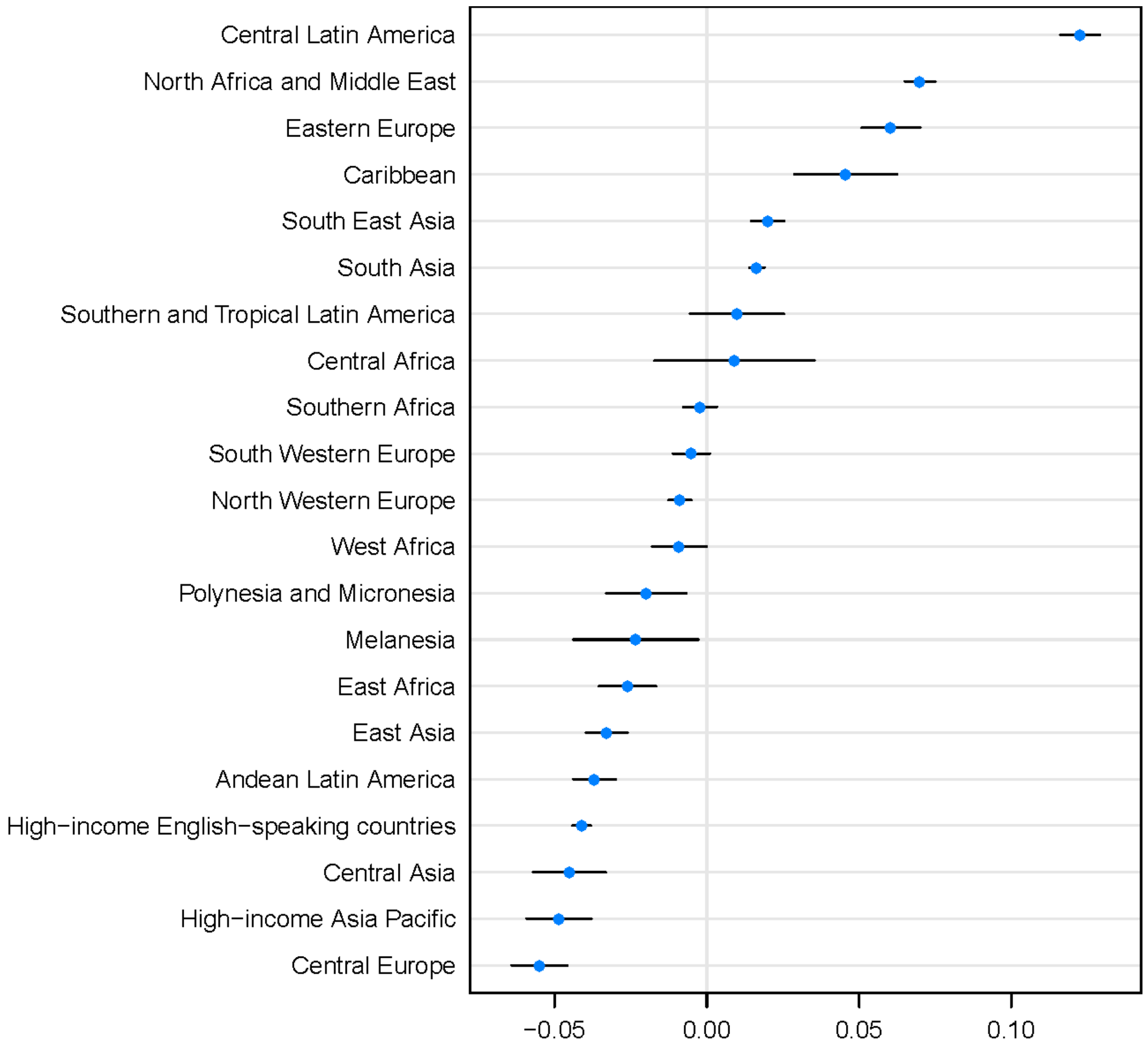
Central Latin America
North Africa and Middle East
Eastern Europe
Caribbean
South East Asia
South Asia
Southern and Tropical Latin America
Central Africa
Southern Africa
South Western Europe
North Western Europe
West Africa
Polynesia and Micronesia
Melanesia
East Africa
East Asia
Andean Latin America
High−income English−speaking countries
Central Asia
High−income Asia Pacific
Central Europe
−0.05
0.00
0.05
0.10

Primary outcome: prevalence of raised blood pressure (SBP ≥140 mmHg or DBP ≥90 mmHg)	
**Independent variables: mean SBP, prevalence (SBP ≥140 mmHg), and prevalence (SBP ≥160 mmHg)**	
**Variables**	**Coefficients (95% CI)**
Intercept	0.14 (0.094, 0.19)
Mean SBP (centred at 130 mmHg)	0.0014 (0.00041, 0.0023)
Probit-transformed prevalence (SBP ≥140 mmHg)	0.85 (0.83, 0.87)
Probit-transformed prevalence (SBP ≥160 mmHg)	0.013 (-0.0022, 0.028)
Mean age of age group (centred at 50 years)	-0.012 (-0.013, -0.011)
Male sex	0.084 (0.064, 0.10)
Study mid-year (per one more recent year since 1975)	-0.0024 (-0.0027, -0.0022)
Natural logarithm of per-capita gross domestic product	0.0055 (0.0018, 0.0092)
Mean SBP * mean age of age group	0.00039 (0.00035, 0.00043)
Mean SBP * male sex	-0.0047 (-0.0060, -0.0035)
Probit-transformed prevalence (SBP ≥140 mmHg) * mean age of age group	0.0019 (0.0011, 0.0026)
Probit-transformed prevalence (SBP ≥140 mmHg) * male sex	0.051 (0.025, 0.078)
Probit-transformed prevalence (SBP ≥160 mmHg) * mean age of age group	-0.0072 (-0.0077, -0.0066)
Probit-transformed prevalence (SBP ≥160 mmHg) * male sex	0.0024 (-0.015, 0.020)
Random effects for regions	Yes
**Number of data points used to fit the model = 6,376**	

Traditional R^2 is not clearly defined for mixed-effect models. The pseudo-R^2 for the model, which describes the proportion of variance explained by both fixed and random factors,[12] was 0.924.

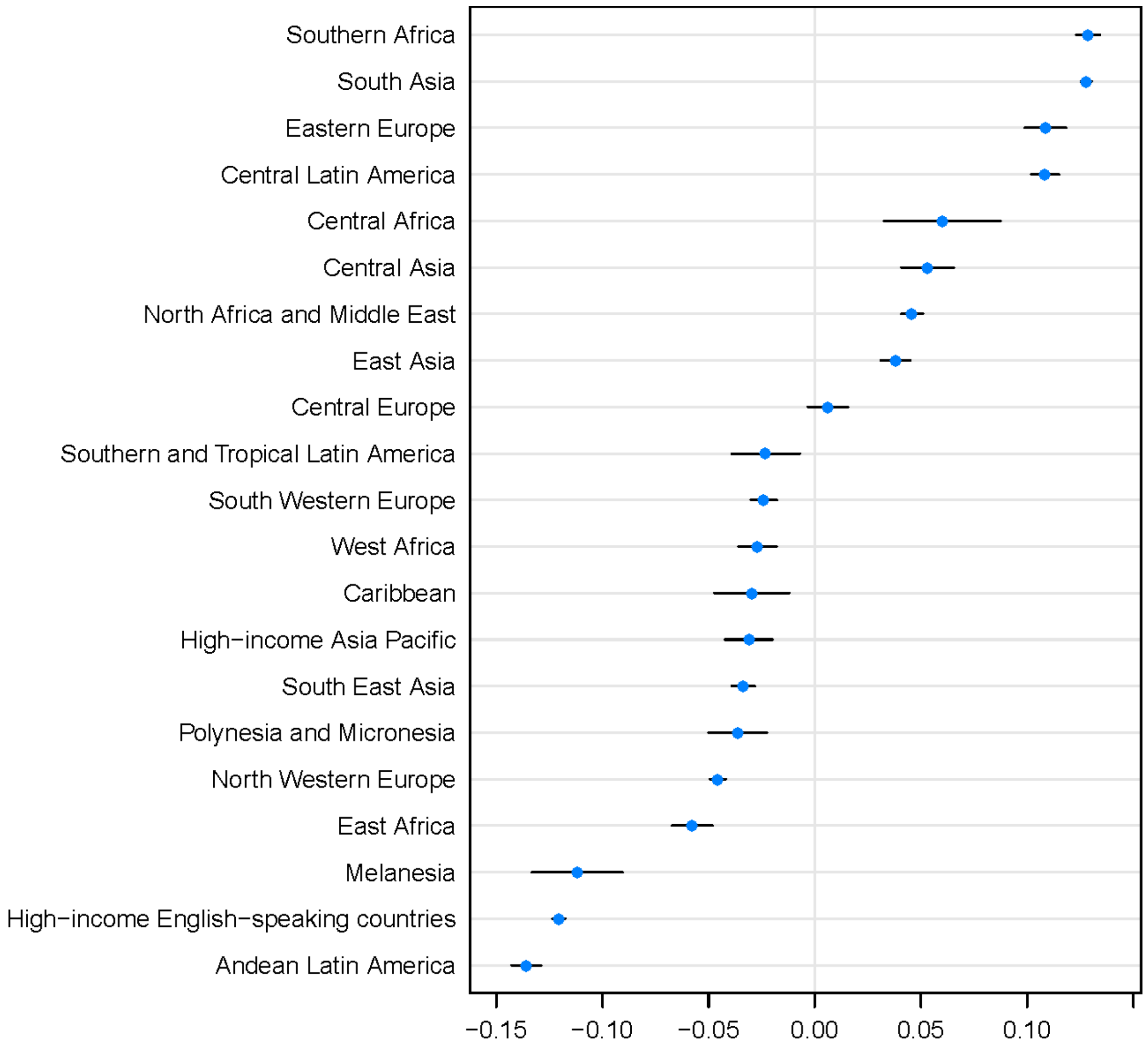

Southern Africa
South Asia
Eastern Europe
Central Latin America
Central Africa
Central Asia
North Africa and Middle East
East Asia
Central Europe
Southern and Tropical Latin America
South Western Europe
West Africa
Caribbean
High−income Asia Pacific
South East Asia
Polynesia and Micronesia
North Western Europe
East Africa
Melanesia
High−income English−speaking countries
Andean Latin America
−0.15
−0.10
−0.05
0.00
0.05
0.10

Primary outcome: prevalence of raised blood pressure (SBP ≥140 mmHg or DBP ≥90 mmHg)	
**Independent variables: mean SBP and prevalence (SBP ≥140 mmHg)**	
**Variables**	**Coefficients (95% CI)**
Intercept	-0.035 (-0.079, 0.0089)
Mean SBP (centred at 130 mmHg)	-0.0020 (-0.0026, -0.0013)
Probit-transformed prevalence (SBP ≥140 mmHg)	0.87 (0.86, 0.88)
Mean age of age group (centred at 50 years)	-0.0053 (-0.0056, -0.0051)
Male sex	0.11 (0.10, 0.12)
Study mid-year (per one more recent year since 1975)	-0.00083 (-0.00096, -0.00069)
Natural logarithm of per-capita gross domestic product	0.017 (0.014, 0.019)
Mean SBP * mean age of age group	0.00072 (0.00070, 0.00074)
Probit-transformed prevalence (SBP ≥140 mmHg) * mean age of age group	-0.0098 (-0.010, -0.0094)
Mean SBP * male sex	-0.0073 (-0.0081, -0.0065)
Probit-transformed prevalence (SBP ≥140 mmHg) * male sex	0.086 (0.074, 0.098)
Random effects for regions	Yes
**Number of data points used to fit the model = 10,454**	

Traditional R^2 is not clearly defined for mixed-effect models. The pseudo-R^2 for the model, which describes the proportion of variance explained by both fixed and random factors,[12] was 0.940.

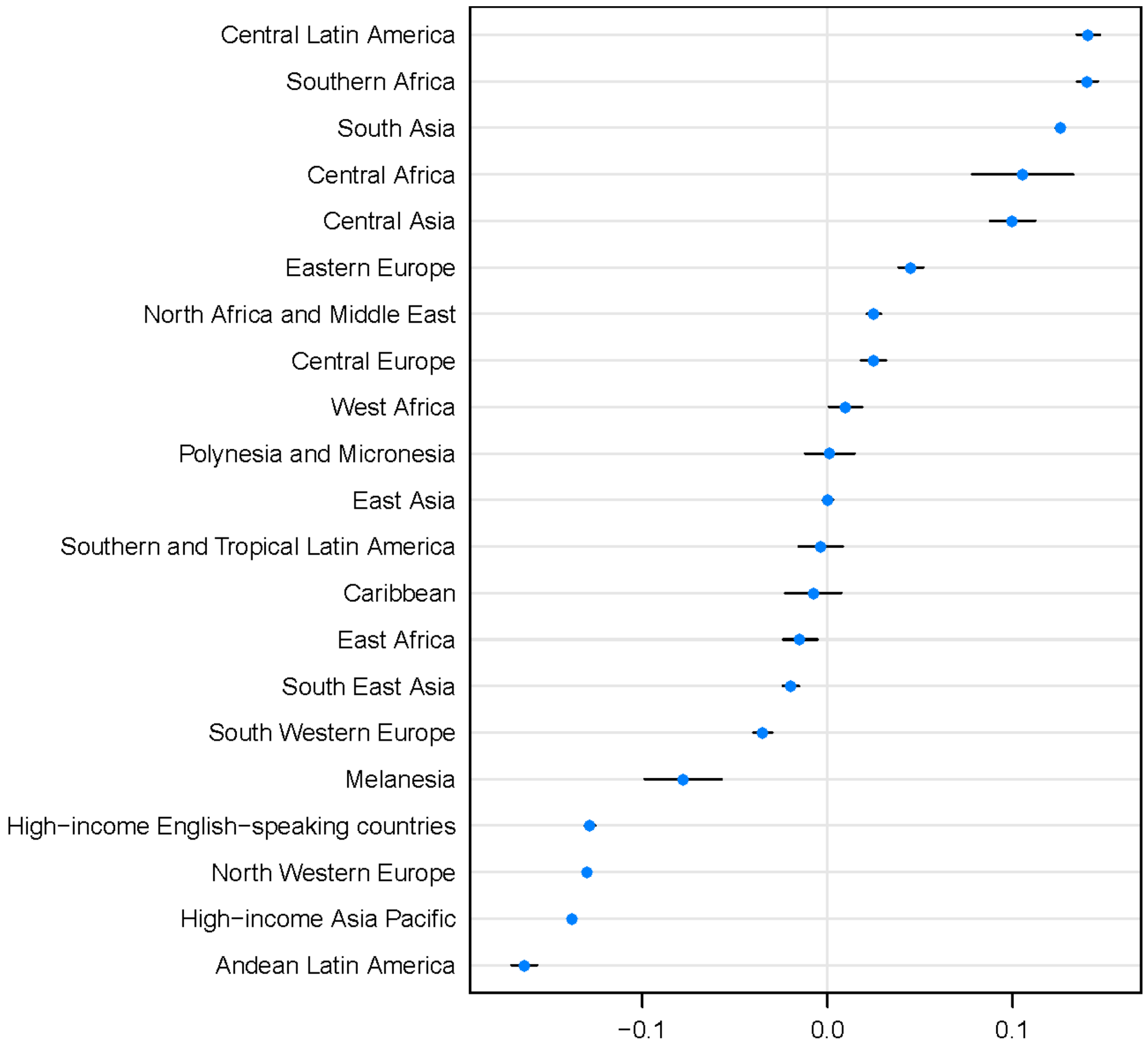
Central Latin America
Southern Africa
South Asia
Central Africa
Central Asia
Eastern Europe
North Africa and Middle East
Central Europe
West Africa
Polynesia and Micronesia
East Asia
Southern and Tropical Latin America
Caribbean
East Africa
South East Asia
South Western Europe
Melanesia
High−income English−speaking countries
North Western Europe
High−income Asia Pacific
Andean Latin America
−0.1
0.0
0.1

**Primary outcome: prevalence of raised blood pressure (SBP ≥140 mmHg or DBP ≥90 mmHg)**	
**Independent variables: mean SBP and prevalence (SBP ≥140 mmHg or DBP ≥90 mmHg or use of antihypertensive medication)**	
**Variables**	**Coefficients (95% CI)**
Intercept	-0.070 (-0.10, -0.038)
Mean SBP (centred at 130 mmHg)	0.024 (0.023, 0.024)
Probit-transformed prevalence (SBP ≥140 mmHg or DBP ≥90 mmHg or use of antihypertensive medication)	0.58 (0.57, 0.59)
Mean age of age group (centred at 50 years)	-0.0086 (-0.0088, -0.0084)
Male sex	0.035 (0.031, 0.039)
Study mid-year (per one more recent year since 1975)	-0.0039 (-0.0041, -0.0037)
Natural logarithm of per-capita gross domestic product	-0.010 (-0.013, -0.0072)
Mean SBP * mean age of age group	0.00064 (0.00062, 0.00066)
Probit-transformed prevalence * mean age of age group	-0.014 (-0.014, -0.013)
Mean SBP * male sex	0.00023 (-0.00039, 0.00084)
Probit-transformed prevalence * male sex	0.031 (0.021, 0.041)
Random effects for regions	Yes
**Number of data points used to fit the model = 8,640**	

Traditional R^2 is not clearly defined for mixed-effect models. The pseudo-R^2 for the model, which describes the proportion of variance explained by both fixed and random factors,[12] was 0.932.

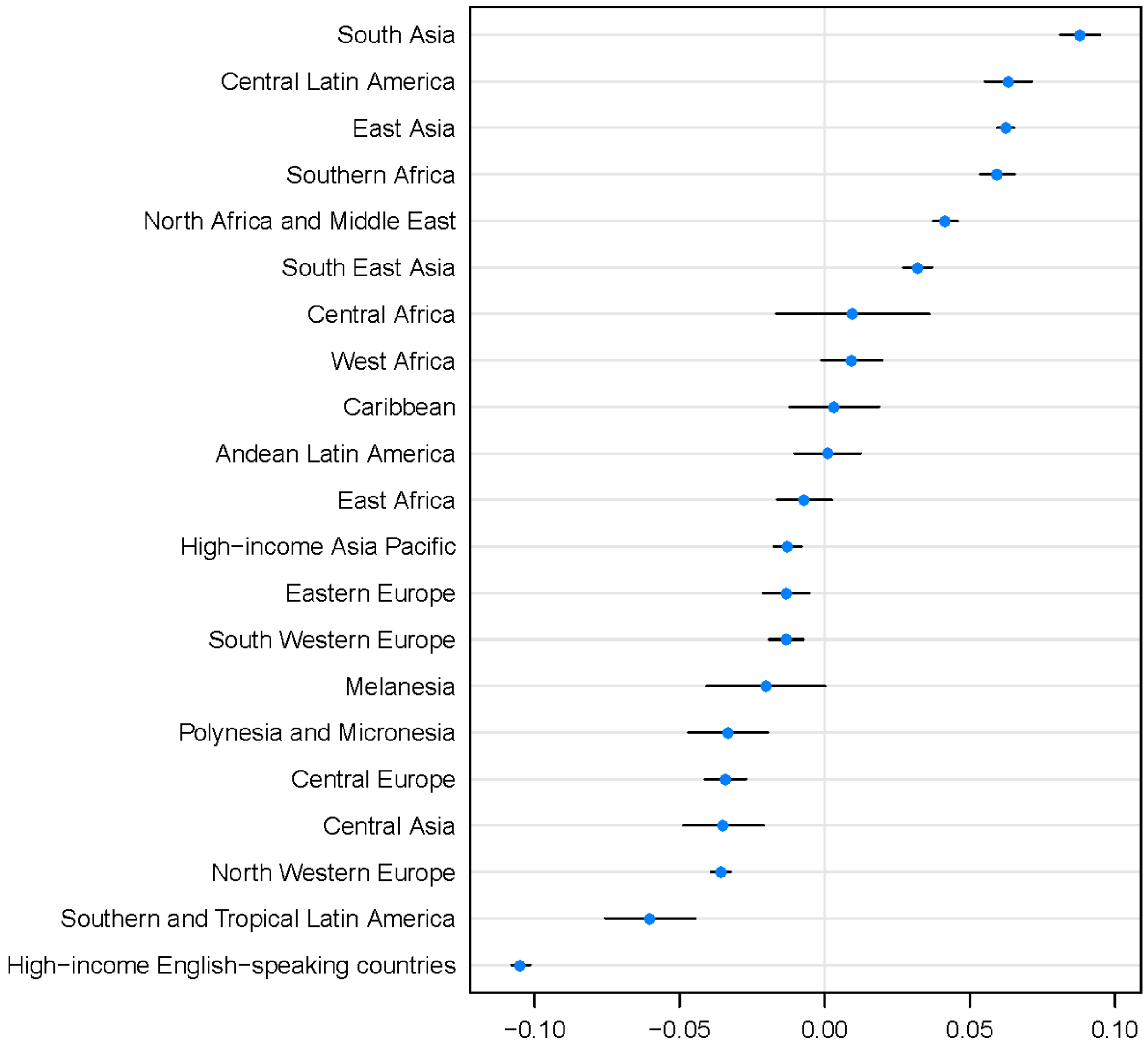
South Asia
Central Latin America
East Asia
Southern Africa
North Africa and Middle East
South East Asia
Central Africa
West Africa
Caribbean
Andean Latin America
East Africa
High−income Asia Pacific
Eastern Europe
South Western Europe
Melanesia
Polynesia and Micronesia
Central Europe
Central Asia
North Western Europe
Southern and Tropical Latin America
High−income English−speaking countries
−0.10
−0.05
0.00
0.05
0.10

Primary outcome: prevalence of raised blood pressure (SBP ≥140 mmHg or DBP ≥90 mmHg)	
**Independent variables: prevalence (SBP ≥140 mmHg or DBP ≥90 mmHg or use of antihypertensive medication) and prevalence (SBP ≥160 mmHg or DBP ≥95 mmHg or use of antihypertensive medication)**	
**Variables**	**Coefficients (95% CI)**
Intercept	-0.12 (-0.16, -0.083)
Probit-transformed prevalence (SBP ≥140 mmHg or DBP ≥90 mmHg or use of antihypertensive medication)	1.18 (1.16, 1.19)
Probit-transformed prevalence (SBP ≥160 mmHg or DBP ≥95 mmHg or use of antihypertensive medication)	-0.21 (-0.22, -0.20)
Mean age of age group (centred at 50 years)	-0.013 (-0.013, -0.013)
Male sex	-0.063 (-0.071, -0.055)
Study mid-year (per one more recent year since 1975)	-0.0045 (-0.0047, -0.0043)
Natural logarithm of per-capita gross domestic product	-0.0027 (-0.0055, 0.00019)
Probit-transformed prevalence (SBP ≥140 mmHg or DBP ≥90 mmHg or use of antihypertensive medication) * mean age of age group	0.011 (0.011, 0.012)
Probit-transformed prevalence (SBP ≥160 mmHg or DBP ≥95 mmHg or use of antihypertensive medication) * mean age of age group	-0.014 (-0.014, -0.013)
Probit-transformed prevalence (SBP ≥140 mmHg or DBP ≥90 mmHg or use of antihypertensive medication) * male sex	0.21 (0.19, 0.22)
Probit-transformed prevalence (SBP ≥160 mmHg or DBP ≥95 mmHg or use of antihypertensive medication) * male sex	-0.19 (-0.21, -0.18)
Random effects for regions	Yes
**Number of data points used to fit the model = 8,432**	

Traditional R^2 is not clearly defined for mixed-effect models. The pseudo-R^2 for the model, which describes the proportion of variance explained by both fixed and random factors,[12] was 0.911.

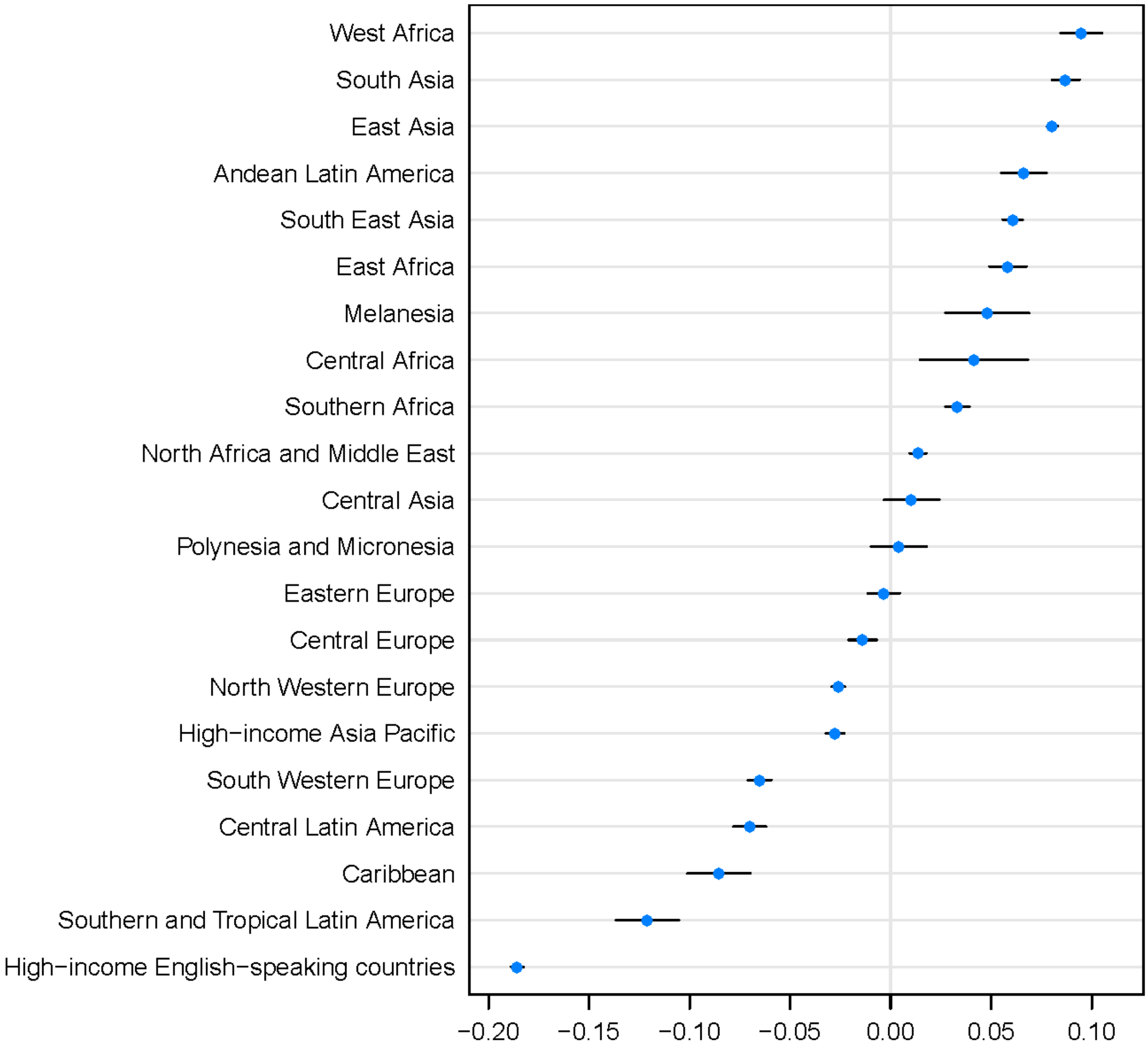

West Africa
South Asia
East Asia
Andean Latin America
South East Asia
East Africa
Melanesia
Central Africa
Southern Africa
North Africa and Middle East
Central Asia
Polynesia and Micronesia
Eastern Europe
Central Europe
North Western Europe
High−income Asia Pacific
South Western Europe
Central Latin America
Caribbean
Southern and Tropical Latin America
High−income English−speaking countries
−0.20
−0.15
−0.10
−0.05
0.00
0.05
0.10

Primary outcome: prevalence of raised blood pressure (SBP ≥140 mmHg or DBP ≥90 mmHg)	
**Independent variables: mean SBP and prevalence (SBP ≥160 mmHg or DBP ≥95 mmHg or use of antihypertensive medication)**	
**Variables**	**Coefficients (95% CI)**
Intercept	-0.089 (-0.13, -0.051)
Mean SBP (centred at 130 mmHg)	0.038 (0.037, 0.038)
Probit-transformed prevalence (SBP ≥160 mmHg or DBP ≥95 mmHg or use of antihypertensive medication)	0.29 (0.29, 0.30)
Mean age of age group (centred at 50 years)	-0.0084 (-0.0086, -0.0081)
Male sex	0.0063 (0.00012, 0.012)
Study mid-year (per one more recent year since 1975)	-0.0052 (-0.0054, -0.0050)
Natural logarithm of per-capita gross domestic product	0.0022 (-0.00062, 0.0049)
Mean SBP * mean age of age group	0.00025 (0.00024, 0.00026)
Probit-transformed prevalence * mean age of age group	-0.0090 (-0.0092, -0.0088)
Mean SBP * male sex	0.0036 (0.0031, 0.0040)
Probit-transformed prevalence * male sex	-0.043 (-0.050, -0.036)
Random effects for regions	Yes
**Number of data points used to fit the model = 8,680**	

Traditional R^2 is not clearly defined for mixed-effect models. The pseudo-R^2 for the model, which describes the proportion of variance explained by both fixed and random factors,[12] was 0.914.

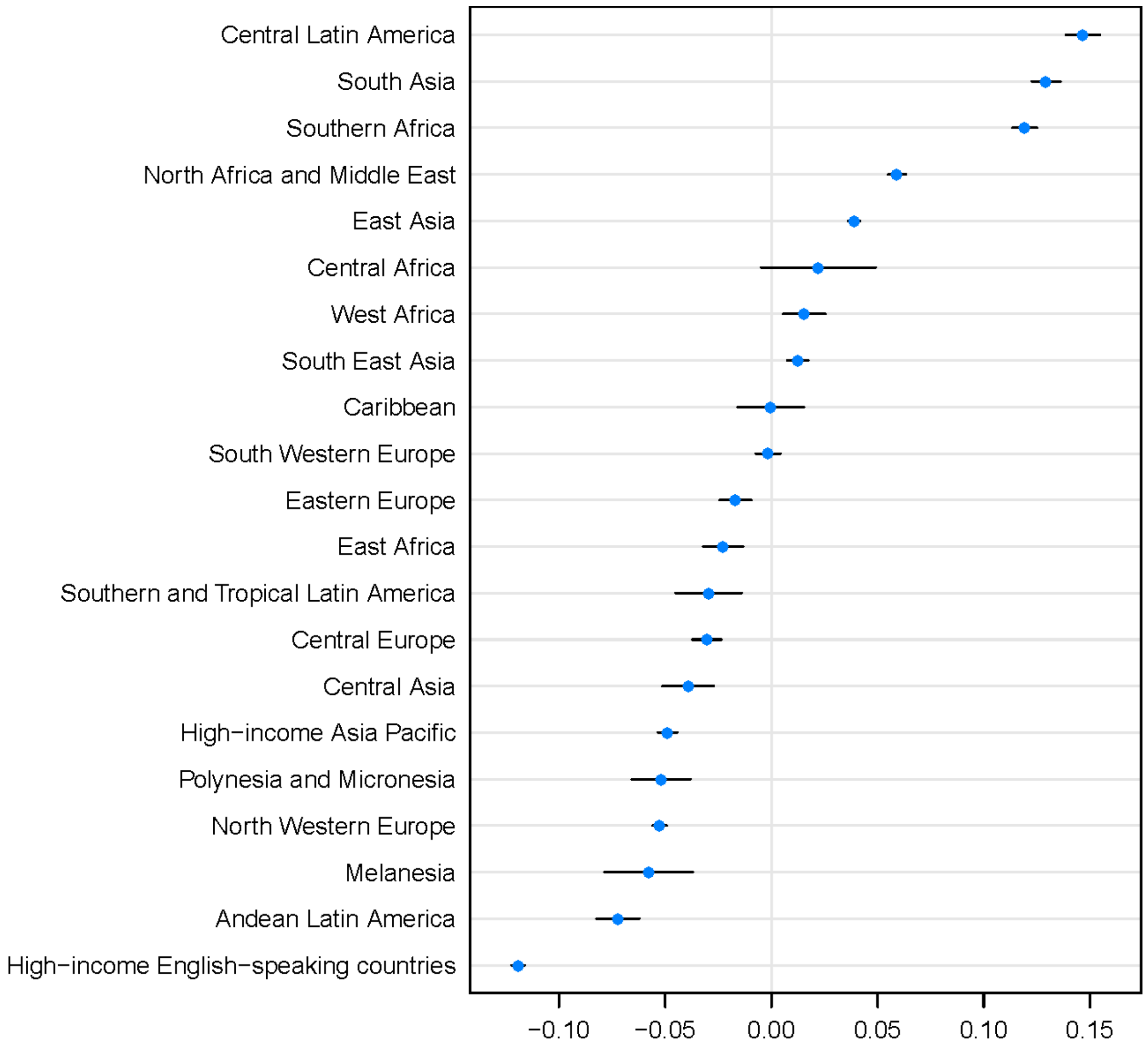
Central Latin America
South Asia
Southern Africa
North Africa and Middle East
East Asia
Central Africa
West Africa
South East Asia
Caribbean
South Western Europe
Eastern Europe
East Africa
Southern and Tropical Latin America
Central Europe
Central Asia
High−income Asia Pacific
Polynesia and Micronesia
North Western Europe
Melanesia
Andean Latin America
High−income English−speaking countries
−0.10
−0.05
0.00
0.05
0.10
0.15

Primary outcome: prevalence of raised blood pressure (SBP ≥140 mmHg or DBP ≥90 mmHg)	
**Independent variables: prevalence (SBP ≥160 mmHg) and prevalence (DBP ≥95 mmHg)**	
**Variables**	**Coefficients (95% CI)**
Intercept	0.82 (0.77, 0.86)
Probit-transformed prevalence (SBP ≥160 mmHg)	0.64 (0.63, 0.64)
Probit-transformed prevalence (DBP ≥85 mmHg)	0.33 (0.33, 0.34)
Mean age of age group (centred at 50 years)	0.0031 (0.0027, 0.0034)
Male sex	-0.016 (-0.025, -0.0073)
Study mid-year (per one more recent year since 1975)	-0.00063 (-0.00086, -0.00040)
Natural logarithm of per-capita gross domestic product	0.0069 (0.0031, 0.011)
Probit-transformed prevalence (SBP ≥160 mmHg) * mean age of age group	0.0076 (0.0074, 0.0078)
Probit-transformed prevalence (SBP ≥160 mmHg) * male sex	-0.092 (-0.098, -0.086)
Probit-transformed prevalence (DBP ≥85 mmHg) * mean age of age group	-0.0070 (-0.0073, -0.0068)
Probit-transformed prevalence (DBP ≥85 mmHg) * male sex	0.027 (0.020, 0.035)
Random effects for regions	Yes
**Number of data points used to fit the model = 6,257**	

Traditional R^2 is not clearly defined for mixed-effect models. The pseudo-R^2 for the model, which describes the proportion of variance explained by both fixed and random factors,[12] was 0.910.

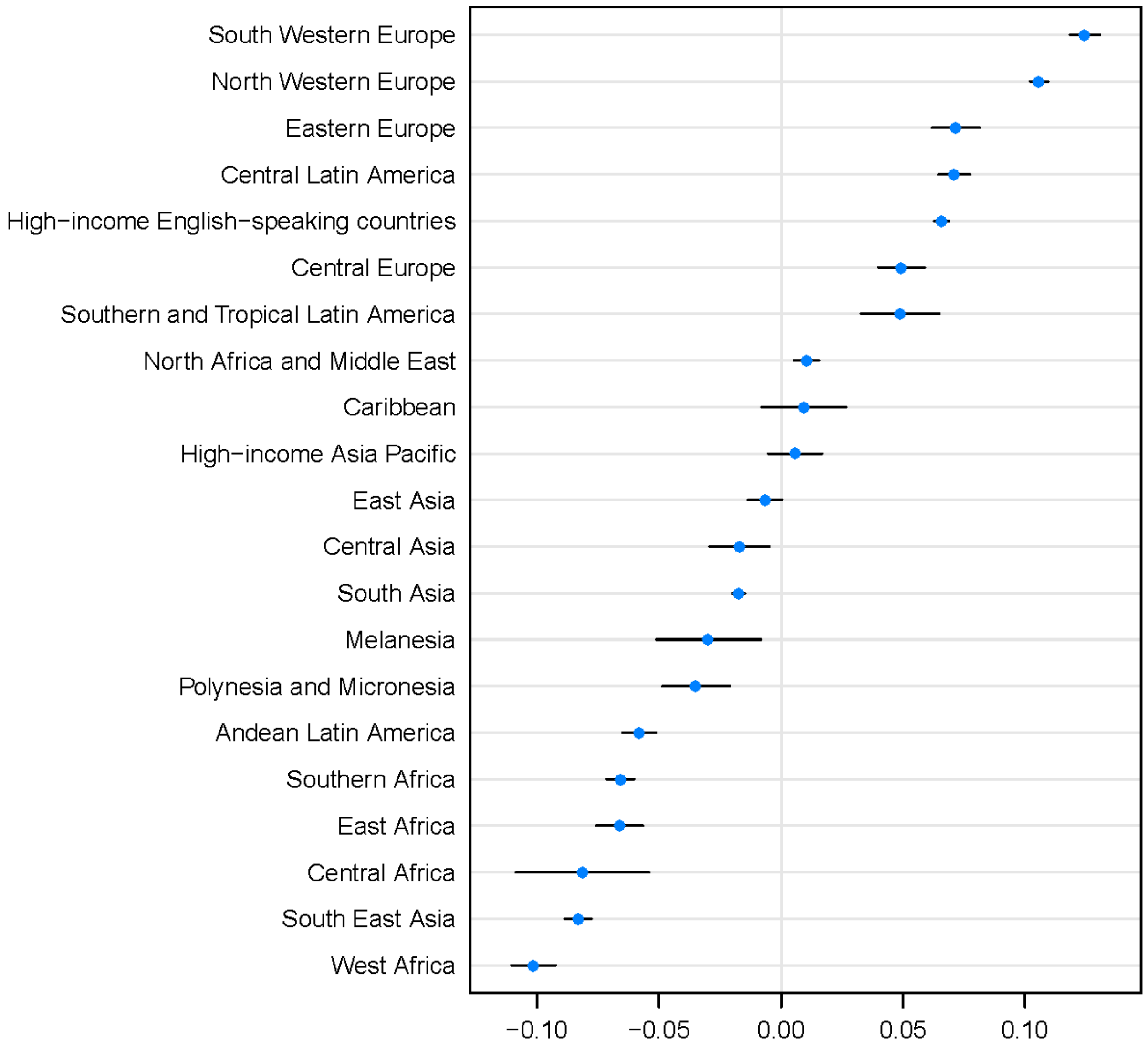
South Western Europe
North Western Europe
Eastern Europe
Central Latin America
High−income English−speaking countries
Central Europe
Southern and Tropical Latin America
North Africa and Middle East
Caribbean
High−income Asia Pacific
East Asia
Central Asia
South Asia
Melanesia
Polynesia and Micronesia
Andean Latin America
Southern Africa
East Africa
Central Africa
South East Asia
West Africa
−0.10
−0.05
0.00
0.05
0.10

**Primary outcome: prevalence of raised blood pressure (SBP ≥140 mmHg or DBP ≥90 mmHg)**	
**Independent variables: mean SBP, mean DBP, prevalence (SBP ≥140 mmHg or DBP ≥90 mmHg or use of antihypertensive medication), and prevalence (SBP ≥160 mmHg or DBP ≥95 mmHg or use of antihypertensive medication)**	
**Variables**	**Coefficients (95% CI)**
Intercept	-0.25 (-0.28, -0.22)
Mean SBP (centred at 130 mmHg)	0.020 (0.020, 0.021)
Mean DBP (centred at 80 mmHg)	0.017 (0.016, 0.017)
Probit-transformed prevalence (SBP ≥140 mmHg or DBP ≥90 mmHg or use of antihypertensive medication)	0.62 (0.60, 0.63)
Probit-transformed prevalence (SBP ≥160 mmHg or DBP ≥95 mmHg or use of antihypertensive medication)	-0.14 (-0.15, -0.13)
Mean age of age group (centred at 50 years)	-0.0045 (-0.0049, -0.0041)
Male sex	-0.061 (-0.069, -0.052)
Study mid-year (per one more recent year since 1975)	-0.0021 (-0.0023, -0.0019)
Natural logarithm of per-capita gross domestic product	-0.0079 (-0.011, -0.0050)
Mean SBP * mean age of age group	0.00057 (0.00055, 0.00059)
Mean SBP * male sex	-0.0032 (-0.0039, -0.0025)
Mean DBP * mean age of age group	-0.00027 (-0.00029, -0.00025)
Mean DBP * male sex	0.00059 (-6.6e-05, 0.0012)
Probit-transformed prevalence (SBP ≥140 mmHg or DBP ≥90 mmHg or use of antihypertensive medication) * mean age of age group	-0.0084 (-0.0091, -0.0078)
Probit-transformed prevalence (SBP ≥140 mmHg or DBP ≥90 mmHg or use of antihypertensive medication) * male sex	0.23 (0.21, 0.25)
Probit-transformed prevalence (SBP ≥160 mmHg or DBP ≥95 mmHg or use of antihypertensive medication) * mean age of age group	-0.00098 (-0.0015, -0.00049)
Probit-transformed prevalence (SBP ≥160 mmHg or DBP ≥95 mmHg or use of antihypertensive medication) * male sex	-0.16 (-0.18, -0.14)
Random effects for regions	Yes
**Number of data points used to fit the model = 8,430**	

Traditional R^2 is not clearly defined for mixed-effect models. The pseudo-R^2 for the model, which describes the proportion of variance explained by both fixed and random factors,[12] was 0.938.

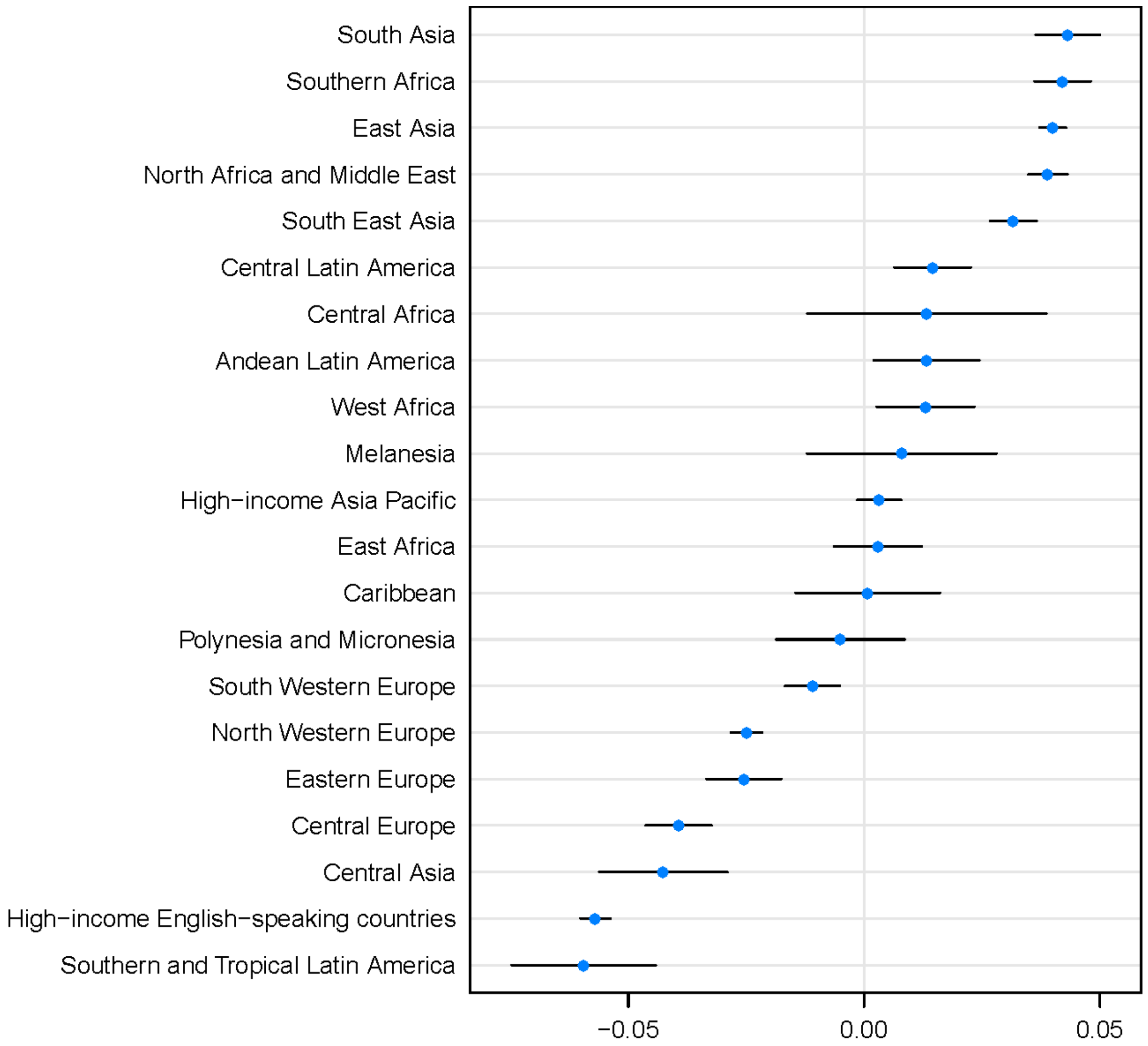

South Asia
Southern Africa
East Asia
North Africa and Middle East
South East Asia
Central Latin America
Central Africa
Andean Latin America
West Africa
Melanesia
High−income Asia Pacific
East Africa
Caribbean
Polynesia and Micronesia
South Western Europe
North Western Europe
Eastern Europe
Central Europe
Central Asia
High−income English−speaking countries
Southern and Tropical Latin America
−0.05
0.00
0.05

Primary outcome: prevalence of raised blood pressure (SBP ≥140 mmHg or DBP ≥90 mmHg)	
**Independent variables: mean SBP, mean DBP, prevalence (SBP ≥160 mmHg or DBP ≥95), and prevalence (SBP ≥160 mmHg or DBP ≥95 mmHg or use of antihypertensive medication)**	
**Variables**	**Coefficients (95% CI)**
Intercept	0.076 (0.031, 0.12)
Mean SBP (centred at 130 mmHg)	0.024 (0.023, 0.024)
Mean DBP (centred at 80 mmHg)	0.0070 (0.0060, 0.0081)
Probit-transformed prevalence (SBP ≥160 mmHg or DBP ≥95 mmHg)	0.46 (0.44, 0.47)
Probit-transformed prevalence (SBP ≥160 mmHg or DBP ≥95 mmHg or use of antihypertensive medication)	0.019 (0.0090, 0.029)
Mean age of age group (centred at 50 years)	-0.0042 (-0.0050, -0.0035)
Male sex	-0.14 (-0.16, -0.12)
Study mid-year (per one more recent year since 1975)	-0.0032 (-0.0035, -0.0030)
Natural logarithm of per-capita gross domestic product	0.0074 (0.0033, 0.011)
Mean SBP * mean age of age group	0.00017 (0.00015, 0.00020)
Mean SBP * male sex	0.0072 (0.0065, 0.0080)
Mean DBP * mean age of age group	-5.4e-05 (-8.8e-05, -2.0e-05)
Mean DBP * male sex	0.0029 (0.0017, 0.0041)
Probit-transformed prevalence (SBP ≥160 mmHg or DBP ≥95 mmHg) * mean age of age group	-0.0068 (-0.0075, -0.0061)
Probit-transformed prevalence (SBP ≥160 mmHg or DBP ≥95 mmHg) * male sex	-0.10 (-0.12, -0.079)
Probit-transformed prevalence (SBP ≥160 mmHg or DBP ≥95 mmHg or use of antihypertensive medication) * mean age of age group	0.00071 (0.00031, 0.0011)
Probit-transformed prevalence (SBP ≥160 mmHg or DBP ≥95 mmHg or use of antihypertensive medication) * male sex	-0.048 (-0.059, -0.038)
Random effects for regions	Yes
**Number of data points used to fit the model = 5,963**	

Traditional R^2 is not clearly defined for mixed-effect models. The pseudo-R^2 for the model, which describes the proportion of variance explained by both fixed and random factors,[12] was 0.905.

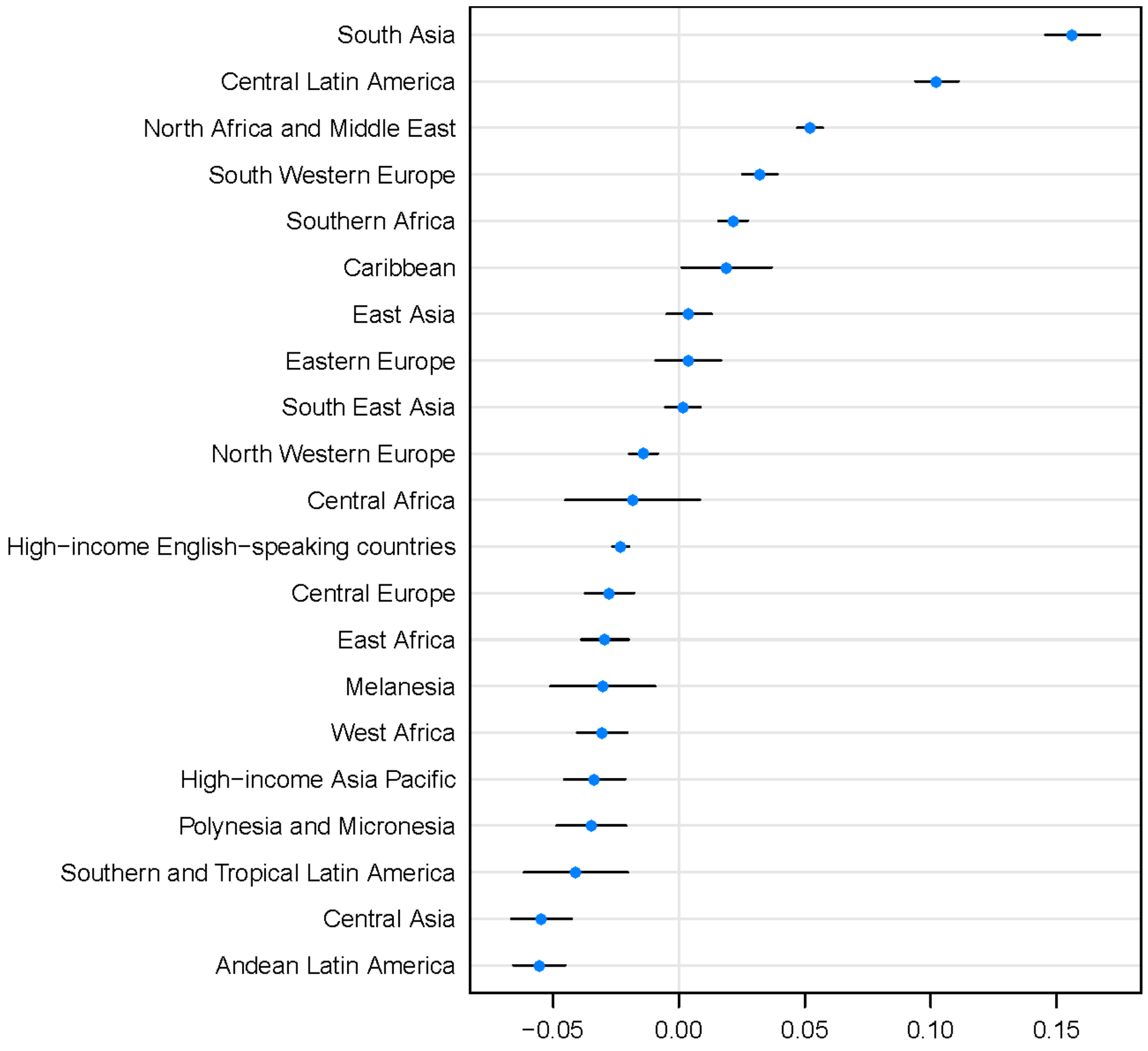
South Asia
Central Latin America
North Africa and Middle East
South Western Europe
Southern Africa
Caribbean
East Asia
Eastern Europe
South East Asia
North Western Europe
Central Africa
High−income English−speaking countries
Central Europe
East Africa
Melanesia
West Africa
High−income Asia Pacific
Polynesia and Micronesia
Southern and Tropical Latin America
Central Asia
Andean Latin America
−0.05
0.00
0.05
0.10
0.15

Appendix Table 4: Results of model validation.

Mean SBP, Test 1, Men											
**Data**		**No. of held out observations**	**Percent covered**	**Error (mmHg) †**				**Absolute error (mmHg)**			
				**Median**	**Q1**	**Q3**	**(p*)**	**Median**	**Q1**	**Q3**	**(p*)**
All		3317	86	0.10	-4.17	4.24	0.3609	4.20	1.95	7.30	0.3609
Super-region	Central and Eastern Europe	219	95	-0.34	-3.59	2.82	0.7176	3.31	1.68	5.86	0.7176
	Central Asia, Middle East and North Africa	501	78	2.84	-1.18	7.42	0.0000	5.04	2.00	8.28	0.0000
	East and Southeast Asia	164	88	-1.23	-4.69	2.33	0.5782	4.03	1.68	7.18	0.5782
	High-income Asia Pacific	*No data from this super-region were among the held-out data*									
	High-income Western countries	1,666	89	-0.93	-4.96	3.11	0.0233	4.09	1.94	6.89	0.0233
	Latin America and Caribbean	342	83	1.11	-3.13	5.88	0.0835	4.83	2.12	7.79	0.0835
	Oceania	95	76	1.54	-2.55	4.98	0.1810	4.23	1.82	8.06	0.1810
	South Asia	42	90	-1.08	-4.47	2.36	0.6070	3.61	1.25	7.31	0.6070
	Sub-Saharan Africa	288	82	1.17	-3.29	5.54	0.1402	4.37	2.00	7.85	0.1402
Urban or rural studies	Rural	385	81	0.07	-4.46	5.07	0.3991	4.61	2.00	8.49	0.3991
	Urban	1,023	83	0.23	-3.69	4.54	0.2371	4.08	1.79	7.48	0.2371
	Both rural and urban	1,909	88	0.09	-4.36	4.07	0.9709	4.17	2.01	6.99	0.9709
Study representative-ness	Community	1,296	81	-0.59	-4.79	4.39	0.8630	4.62	2.05	8.18	0.8630
	Subnational	661	87	0.10	-3.91	4.19	0.2964	4.09	1.86	7.29	0.2964
	National	1,360	90	0.54	-3.75	4.17	0.3989	3.99	1.87	6.61	0.3989
Age band	18-40	1,032	86	0.14	-3.86	3.53	0.4874	3.71	1.73	6.30	0.4874
	40-60	1,060	83	0.17	-3.53	4.27	0.2538	3.91	1.84	6.83	0.2538
	60 and above	1,225	88	0.03	-5.24	4.86	0.9392	5.00	2.35	8.87	0.9392
Years	1975-1989	585	87	0.31	-4.35	4.25	0.6852	4.28	2.02	7.14	0.6852
	1990-1999	743	86	-0.86	-5.63	3.51	0.1383	4.64	2.17	7.70	0.1383
	2000-2009	1,444	83	0.10	-3.83	4.60	0.2735	4.16	1.91	7.54	0.2735
	2010-2015	545	92	0.91	-3.04	4.40	0.0688	3.75	1.66	6.40	0.0688
Data density	Data poor	147	88	0.39	-4.15	2.91	0.6507	3.60	1.65	6.32	0.6507
	Average data density	580	85	-0.21	-4.03	3.50	0.7372	3.78	1.43	7.12	0.7372
	Data rich	2,590	86	0.23	-4.19	4.54	0.2018	4.38	2.06	7.42	0.2018

Mean SBP, Test 1, Women												
**Data**		**No. of held out observations**	**Percent covered**	**Error (mmHg) †**				**Absolute error (mmHg)**			
				**Median**	**Q1**	**Q3**	**(p*)**	**Median**	**Q1**	**Q3**	**(p*)**
All		2,717	89	0.99	-3.21	5.33	0.0038	4.42	2.00	7.73	0.0038
Super-region	Central and Eastern Europe	162	93	4.86	1.61	7.06	0.0034	5.36	3.16	7.52	0.0034
	Central Asia, Middle East and North Africa	255	89	0.31	-2.94	5.14	0.4790	3.72	1.57	7.17	0.4790
	East and Southeast Asia	477	86	-0.02	-5.23	3.31	0.5184	4.18	1.96	8.10	0.5184
	High-income Asia Pacific	120	86	8.18	6.09	9.92	0.0000	8.31	6.21	10.02	0.0000
	High-income Western countries	872	89	0.90	-3.14	4.74	0.1460	4.07	1.92	7.55	0.1460
	Latin America and Caribbean	323	92	0.13	-3.13	4.67	0.3148	4.06	1.77	7.00	0.3148
	Oceania	132	82	0.73	-3.29	4.96	0.4188	4.33	2.05	8.80	0.4188
	South Asia	59	93	0.17	-4.63	4.52	0.9657	4.62	2.17	8.37	0.9657
	Sub-Saharan Africa	317	88	-0.30	-3.72	5.10	0.6070	4.29	2.14	7.38	0.6070
Urban or rural studies	Rural	378	85	1.74	-3.30	7.32	0.0844	5.26	2.70	9.15	0.0844
	Urban	776	87	0.86	-2.87	4.75	0.0549	3.84	1.84	7.46	0.0549
	Both rural and urban	1,563	91	0.97	-3.47	5.29	0.1177	4.46	2.03	7.50	0.1177
Study representative-ness	Community	1,069	86	0.42	-3.96	5.09	0.2525	4.62	2.02	8.27	0.2525
	Subnational	440	83	2.12	-2.44	6.16	0.0425	5.10	2.24	8.92	0.0425
	National	1,208	93	1.17	-2.83	5.16	0.0480	4.02	1.88	7.00	0.0480
Age band	18-40	909	92	0.86	-2.33	4.10	0.0001	3.27	1.60	5.79	0.0001
	40-60	882	85	1.04	-3.12	5.52	0.0014	4.49	2.03	7.70	0.0014
	60 and above	926	89	1.10	-4.88	6.99	0.0130	5.92	2.82	10.19	0.0130
Years	1975-1989	453	81	1.27	-3.83	5.92	0.0509	4.87	2.14	9.18	0.0509
	1990-1999	610	87	1.62	-2.76	5.27	0.0943	4.16	2.10	7.21	0.0943
	2000-2009	1,180	89	1.07	-3.01	5.47	0.0370	4.56	2.02	7.87	0.0370
	2010-2015	474	97	-0.20	-4.19	4.35	0.9349	4.23	1.78	7.36	0.9349
Data density	Data poor	152	86	-2.19	-5.31	1.27	0.2379	4.42	2.04	7.65	0.2379
	Average data density	547	89	-0.04	-4.32	4.42	0.6762	4.40	2.05	7.38	0.6762
	Data rich	2,018	89	1.52	-2.74	5.73	0.0008	4.43	1.99	7.82	0.0008

Mean SBP, Test 2, Men											
**Data**		**No. of held out observations**	**Percent covered**	**Error (mmHg) †**				**Absolute error (mmHg)**			
				**Median**	**Q1**	**Q3**	**(p*)**	**Median**	**Q1**	**Q3**	**(p*)**
All		3,512	74	0.71	-2.63	4.37	0.0004	3.46	1.65	6.43	0.0004
Super-region	Central and Eastern Europe	368	74	0.34	-3.09	3.44	0.4494	3.30	1.64	6.75	0.4494
	Central Asia, Middle East and North Africa	441	67	2.04	-1.95	6.27	0.0023	4.50	1.99	7.56	0.0023
	East and Southeast Asia	353	66	-0.32	-4.74	3.46	0.5541	4.05	1.89	7.03	0.5541
	High-income Asia Pacific	362	75	0.67	-1.78	5.34	0.0088	3.10	1.45	6.17	0.0088
	High-income Western countries	1,127	79	0.52	-2.24	3.50	0.0748	2.95	1.39	5.40	0.0748
	Latin America and Caribbean	282	73	1.49	-2.85	5.65	0.0717	4.44	1.99	7.42	0.0717
	Oceania	108	81	-0.80	-3.35	2.94	0.8421	3.19	1.49	6.76	0.8421
	South Asia	191	74	-1.68	-4.71	1.69	0.1179	3.41	1.69	5.99	0.1179
	Sub-Saharan Africa	280	75	2.49	-1.15	5.22	0.0103	3.90	2.06	6.38	0.0103
Urban or rural studies	Rural	508	68	1.25	-2.67	5.09	0.0523	3.92	1.89	7.28	0.0523
	Urban	2,107	78	0.52	-2.52	4.21	0.0179	3.32	1.58	6.09	0.0179
	Both rural and urban	897	69	0.96	-2.89	4.39	0.0545	3.74	1.75	7.04	0.0545
Study representative-ness	Community	1,346	68	0.78	-3.41	4.32	0.2354	3.90	1.82	7.09	0.2354
	Subnational	551	72	0.55	-2.93	4.38	0.0901	3.70	1.75	6.29	0.0901
	National	1,615	80	0.75	-2.12	4.39	0.0028	3.12	1.48	6.01	0.0028
Age band	18-40	1,131	77	0.24	-2.44	3.22	0.5218	2.82	1.30	5.23	0.5218
	40-60	1,103	73	0.68	-2.44	4.06	0.0185	3.33	1.59	6.04	0.0185
	60 and above	1,278	72	1.28	-2.90	5.80	0.0000	4.43	2.04	7.88	0.0000
Years	1975-1989	558	74	-0.16	-2.65	3.07	0.5002	2.76	1.36	5.55	0.5002
	1990-1999	444	70	0.90	-3.04	3.89	0.2907	3.43	1.90	6.02	0.2907
	2000-2009	1,785	72	0.61	-2.95	4.30	0.0980	3.60	1.72	6.72	0.0980
	2010-2015	725	83	1.85	-1.38	5.51	0.0002	3.74	1.61	6.48	0.0002
Data density	Data poor	*Not applicable for source-based test*									
	Average data density	545	81	-0.08	-3.77	2.61	0.7749	3.31	1.43	6.37	0.7749
	Data rich	2,967	73	0.85	-2.46	4.61	0.0001	3.50	1.69	6.43	0.0001
Hold out pattern	Post-2000 data removed	1,412	75	1.26	-2.30	5.29	0.0003	3.95	1.83	6.69	0.0003
	Random set of data removed	2,100	73	0.36	-2.81	3.59	0.1181	3.23	1.52	6.19	0.1181

Mean SBP, Test 2, Women											
**Data**		**No. of held out observations**	**Percent covered**	**Error (mmHg) †**				**Absolute error (mmHg)**			
				**Median**	**Q1**	**Q3**	**(p*)**	**Median**	**Q1**	**Q3**	**(p*)**
All		3,428	77	0.22	-3.27	3.75	0.3868	3.51	1.56	6.29	0.3868
Super-region	Central and Eastern Europe	283	76	-0.23	-4.09	2.28	0.4455	3.05	1.28	6.32	0.4455
	Central Asia, Middle East and North Africa	420	73	1.94	-2.00	5.92	0.0314	4.47	1.96	7.40	0.0314
	East and Southeast Asia	341	67	0.43	-3.53	4.03	0.8743	3.78	1.61	7.16	0.8743
	High-income Asia Pacific	371	75	0.83	-2.37	5.39	0.1126	3.58	1.93	6.15	0.1126
	High-income Western countries	1,135	80	0.09	-3.09	3.16	0.7701	3.12	1.45	5.74	0.7701
	Latin America and Caribbean	310	84	-1.35	-4.08	1.56	0.2606	3.38	1.47	5.77	0.2606
	Oceania	109	85	0.36	-3.60	3.52	0.9094	3.60	1.37	6.32	0.9094
	South Asia	193	77	-1.04	-4.45	2.24	0.5760	3.75	1.92	6.32	0.5760
	Sub-Saharan Africa	266	80	0.34	-3.73	4.70	0.6762	4.12	1.69	6.57	0.6762
Urban or rural studies	Rural	507	72	-0.17	-4.17	4.15	0.9537	4.17	1.97	6.78	0.9537
	Urban	891	72	-0.24	-4.25	3.34	0.9108	3.76	1.67	6.78	0.9108
	Both rural and urban	2,030	80	0.44	-2.75	3.90	0.2370	3.23	1.46	5.84	0.2370
Study representative-ness	Community	1,353	71	-0.11	-4.29	3.97	0.9556	4.13	1.81	7.04	0.9556
	Subnational	489	80	0.11	-2.91	3.13	0.9065	3.03	1.34	5.60	0.9065
	National	1,586	82	0.45	-2.73	3.90	0.2533	3.22	1.47	5.76	0.2533
Age band	18-40	1,125	80	0.09	-2.68	3.05	0.4368	2.86	1.25	4.91	0.4368
	40-60	1,075	79	-0.03	-3.57	3.03	0.9828	3.29	1.48	5.71	0.9828
	60 and above	1,228	73	0.71	-3.90	5.51	0.0194	4.63	2.19	8.00	0.0194
Years	1975-1989	457	70	-1.64	-4.48	2.34	0.4435	3.75	1.96	6.18	0.4435
	1990-1999	513	68	0.43	-3.37	4.24	0.6315	3.87	1.83	6.47	0.6315
	2000-2009	1,742	76	0.64	-2.76	4.06	0.1798	3.44	1.53	6.41	0.1798
	2010-2015	716	90	0.05	-3.24	3.60	0.9984	3.33	1.30	5.73	0.9984
Data density	Data poor	*Not applicable for source-based test*									
	Average data density	566	81	0.07	-3.34	3.97	0.4948	3.63	1.58	6.57	0.4948
	Data rich	2,862	76	0.24	-3.25	3.71	0.5073	3.50	1.55	6.21	0.5073
Hold out pattern	Post-2000 data removed	1,325	84	0.80	-2.70	4.35	0.1196	3.48	1.58	6.19	0.1196
	Random set of data removed	2,103	73	-0.29	-3.68	3.38	0.9056	3.54	1.53	6.33	0.9056

**Mean DBP, Test 1, Men**											
**Data**		**No. of held out observations**	**Percent covered**	**Error (mmHg) †**				**Absolute error (mmHg)**			
				**Median**	**Q1**	**Q3**	**(p*)**	**Median**	**Q1**	**Q3**	**(p*)**
All		3,317	87	0.50	-2.02	3.12	0.0003	2.61	1.20	4.60	0.0003
Super-region	Central and Eastern Europe	219	93	-0.32	-2.32	1.56	0.1394	1.92	0.92	3.28	0.1394
	Central Asia, Middle East and North Africa	501	91	-0.14	-2.15	2.21	0.8818	2.16	1.06	3.77	0.8818
	East and Southeast Asia	164	90	-0.62	-2.97	1.69	0.1371	2.42	1.00	4.62	0.1371
	High-income Asia Pacific	*No data from this region were among the held-out data*									
	High-income Western countries	1,666	87	1.19	-1.39	4.05	0.0000	2.79	1.26	4.82	0.0000
	Latin America and Caribbean	342	85	0.16	-2.56	3.04	0.2299	2.77	1.20	4.99	0.2299
	Oceania	95	78	0.54	-3.65	3.32	0.6087	3.57	1.34	5.47	0.6087
	South Asia	42	93	-4.27	-5.72	-3.06	0.0001	4.27	3.06	5.72	0.0001
	Sub-Saharan Africa	288	82	0.35	-2.41	2.53	0.8871	2.47	1.22	4.75	0.8871
Urban or rural studies	Rural	385	85	-0.35	-3.26	2.45	0.1929	2.97	1.42	5.01	0.1929
	Urban	1,023	85	0.35	-2.05	3.12	0.0190	2.60	1.13	4.64	0.0190
	Both rural and urban	1,909	89	0.72	-1.72	3.28	0.0003	2.54	1.19	4.48	0.0003
Study representative-ness	Community	1,360	88	0.72	-1.68	3.32	0.0066	2.55	1.19	4.63	0.0066
	Subnational	661	88	1.05	-1.42	3.88	0.0000	2.69	1.24	4.76	0.0000
	National	1,296	86	-0.03	-2.64	2.52	0.8323	2.58	1.19	4.55	0.8323
Age band	18-40	1,032	88	0.68	-1.82	3.24	0.0143	2.63	1.22	4.39	0.0143
	40-60	1,060	85	0.42	-1.93	2.61	0.0493	2.26	1.13	3.98	0.0493
	60 and above	1,225	88	0.43	-2.26	3.77	0.0934	2.93	1.28	5.22	0.0934
Years	1975-1989	585	86	0.93	-1.68	3.70	0.0023	2.60	1.23	4.72	0.0023
	1990-1999	743	85	0.12	-2.38	2.50	0.6996	2.46	1.04	4.62	0.6996
	2000-2009	1,444	87	0.42	-2.03	2.97	0.0660	2.55	1.18	4.35	0.0660
	2010-2015	545	92	1.18	-1.84	3.95	0.0014	2.91	1.48	4.82	0.0014
Data density	Data poor	147	92	0.50	-1.77	3.23	0.4867	2.53	1.17	3.75	0.4867
	Average data density	580	84	-1.15	-3.86	1.35	0.0000	2.57	1.24	4.73	0.0000
	Data rich	2,590	88	0.87	-1.66	3.58	0.0000	2.62	1.19	4.62	0.0000

Mean DBP, Test 1, Women											
**Data**		**No. of held out observations**	**Percent covered**	**Error (mmHg) †**				**Absolute error (mmHg)**			
				**Median**	**Q1**	**Q3**	**(p*)**	**Median**	**Q1**	**Q3**	**(p*)**
All		2,717	88	0.32	-2.48	2.99	0.0833	2.76	1.33	4.81	0.0833
Super-region	Central and Eastern Europe	162	81	3.66	1.62	5.96	0.0000	4.02	2.29	5.96	0.0000
	Central Asia, Middle East and North Africa	255	92	-0.36	-2.40	2.28	0.8480	2.37	1.33	3.92	0.8480
	East and Southeast Asia	477	88	-1.70	-4.35	1.09	0.0000	3.01	1.33	5.38	0.0000
	High-income Asia Pacific	120	98	-0.20	-1.92	0.91	0.1000	1.25	0.52	2.69	0.1000
	High-income Western countries	872	86	2.03	-0.85	4.42	0.0000	3.13	1.55	5.09	0.0000
	Latin America and Caribbean	323	93	-0.10	-2.45	2.36	0.7987	2.40	1.11	3.76	0.7987
	Oceania	132	85	-0.40	-3.37	2.42	0.6792	2.96	1.40	4.93	0.6792
	South Asia	59	92	-1.92	-2.93	0.37	0.0681	2.05	1.13	2.93	0.0681
	Sub-Saharan Africa	317	86	-0.51	-3.19	1.84	0.1067	2.70	1.27	4.55	0.1067
Urban or rural studies	Rural	378	85	0.52	-2.52	3.12	0.5675	2.92	1.30	5.07	0.5675
	Urban	776	86	1.06	-2.12	3.61	0.0009	2.88	1.35	4.91	0.0009
	Both rural and urban	1,563	90	-0.02	-2.65	2.73	0.7970	2.70	1.33	4.73	0.7970
Study representative-ness	Community	1,069	85	0.48	-2.65	3.49	0.0471	2.96	1.46	5.11	0.0471
	Subnational	440	88	1.02	-1.66	3.49	0.0313	2.74	1.30	4.66	0.0313
	National	1,208	91	0.07	-2.55	2.59	0.6670	2.59	1.27	4.57	0.6670
Age band	18-40	909	87	0.83	-2.05	3.12	0.0001	2.70	1.35	4.54	0.0001
	40-60	882	86	0.35	-2.08	2.70	0.0587	2.44	1.20	4.27	0.0587
	60 and above	926	92	0.03	-3.34	3.12	0.3582	3.28	1.59	5.33	0.3582
Years	1980-1989	453	82	1.82	-1.70	4.13	0.0005	3.21	1.80	5.22	0.0005
	1990-1999	610	89	0.80	-1.83	2.86	0.0887	2.50	1.19	4.21	0.0887
	2000-2009	1,180	88	0.13	-2.59	3.01	0.5900	2.75	1.36	5.02	0.5900
	2010-2014	474	93	-0.69	-3.15	1.79	0.0424	2.66	1.16	4.59	0.0424
Data density	Data poor	152	88	0.06	-2.98	2.20	0.6892	2.66	1.40	4.49	0.6892
	Average data density	547	88	-0.85	-3.32	1.92	0.0059	2.59	1.36	4.36	0.0059
	Data rich	2,018	88	0.66	-2.14	3.54	0.0004	2.84	1.31	4.98	0.0004

Mean DBP, Test 2, Men											
Data		No. of held out observations	Percent covered	Error (mmHg) †				Absolute error (mmHg)			
				Median	Q1	Q3	(p*)	Median	Q1	Q3	(p*)
All		3,512	79	0.25	-1.84	2.55	0.0001	2.17	0.98	4.00	0.0001
Super-region	Central and Eastern Europe	368	79	0.21	-1.78	2.53	0.0256	2.10	0.93	3.94	0.0256
	Central Asia, Middle East and North Africa	441	84	0.25	-1.58	2.51	0.0325	2.08	0.85	3.65	0.0325
	East and Southeast Asia	353	77	-0.26	-2.41	1.94	0.6525	2.23	1.14	3.66	0.6525
	High-income Asia Pacific	362	81	-0.17	-1.64	1.46	0.5731	1.59	0.72	3.01	0.5731
	High-income Western countries	1,127	75	0.43	-1.68	2.78	0.0052	2.21	1.08	4.17	0.0052
	Latin America and Caribbean	282	79	0.51	-2.02	3.91	0.0251	2.72	1.23	4.90	0.0251
	Oceania	108	72	0.69	-2.17	3.33	0.5185	2.79	1.46	5.11	0.5185
	South Asia	191	83	0.83	-0.84	2.53	0.0174	1.80	0.84	3.18	0.0174
	Sub-Saharan Africa	280	82	0.02	-2.41	2.64	0.7195	2.56	1.30	4.11	0.7195
Urban or rural studies	Rural	508	76	0.85	-1.67	3.03	0.0517	2.40	1.20	4.21	0.0517
	Urban	897	72	0.21	-2.33	2.66	0.1727	2.47	1.20	4.52	0.1727
	Both rural and urban	2,107	82	0.17	-1.73	2.37	0.0012	2.00	0.89	3.66	0.0012
Study representative-ness	Community	1,346	74	0.17	-2.36	2.55	0.3670	2.43	1.19	4.37	0.3670
	Subnational	551	75	0.54	-1.57	3.23	0.0029	2.25	1.04	4.14	0.0029
	National	1,615	84	0.24	-1.56	2.40	0.0015	1.95	0.87	3.51	0.0015
Age band	18-40	1,131	81	-0.04	-1.83	1.99	0.4810	1.88	0.86	3.42	0.4810
	40-60	1,103	81	0.36	-1.63	2.40	0.0076	2.03	0.95	3.70	0.0076
	60 and above	1,278	75	0.50	-2.02	3.26	0.0004	2.60	1.25	4.74	0.0004
Years	1980-1989	558	72	-0.07	-2.25	2.12	0.9127	2.18	0.92	4.13	0.9127
	1990-1999	444	78	0.17	-2.08	2.24	0.7435	2.17	1.13	3.66	0.7435
	2000-2009	1,785	78	0.28	-1.76	2.54	0.0023	2.16	0.98	4.08	0.0023
	2010-2014	725	84	0.58	-1.47	3.16	0.0000	2.15	0.96	3.98	0.0000
Data density	Data poor	*Not applicable for source-based test*									
	Average data density	545	84	-0.33	-2.34	1.83	0.2300	2.09	0.97	3.81	0.2300
	Data rich	2,967	77	0.36	-1.73	2.70	0.0000	2.18	0.99	4.05	0.0000
Hold out pattern	Post-2000 data removed	1,412	79	0.75	-1.33	3.30	0.0000	2.22	0.99	4.34	0.0000
	Random set of data removed	2,100	78	-0.09	-2.14	2.09	0.7714	2.12	0.98	3.89	0.7714

Mean DBP, Test 2, Women

Data		No. of held out observations	Percent covered	Error (mmHg) †				Absolute error (mmHg)			
				Median	Q1	Q3	(p*)	Median	Q1	Q3	(p*)
All		3,427	78	-0.07	-2.23	2.30	0.1631	2.27	1.05	4.11	0.1631
Super-region	Central and Eastern Europe	283	79	-0.07	-2.70	1.94	0.5592	2.24	1.18	3.73	0.5592
	Central Asia, Middle East and North Africa	420	79	-0.42	-2.69	2.21	0.9722	2.57	1.30	4.20	0.9722
	East and Southeast Asia	341	74	-0.46	-2.60	1.62	0.3684	2.08	0.89	4.11	0.3684
	High-income Asia Pacific	370	83	-0.73	-1.99	0.70	0.1222	1.51	0.70	2.89	0.1222
	High-income Western countries	1,135	73	0.35	-2.06	3.05	0.0125	2.58	1.14	4.54	0.0125
	Latin America and Caribbean	310	90	0.11	-1.79	2.54	0.1529	2.09	0.89	3.46	0.1529
	Oceania	109	74	1.42	-2.57	3.34	0.0965	3.19	1.56	5.78	0.0965
	South Asia	193	88	1.20	-0.24	2.46	0.0007	1.71	0.90	3.06	0.0007
	Sub-Saharan Africa	266	78	-1.17	-4.13	1.71	0.0141	3.04	1.48	4.68	0.0141
Urban or rural studies	Rural	507	73	-0.20	-2.92	2.37	0.5582	2.63	1.21	4.61	0.5582
	Urban	891	75	-0.35	-2.53	2.16	0.8884	2.34	0.96	4.20	0.8884
	Both rural and urban	2,029	81	0.06	-2.01	2.31	0.0388	2.17	1.03	3.94	0.0388
Study representative-ness	Community	1,353	73	-0.37	-2.80	2.40	0.9347	2.63	1.22	4.52	0.9347
	Subnational	489	81	0.40	-1.62	2.56	0.0692	2.08	0.99	3.82	0.0692
	National	1,585	82	0.00	-1.99	2.15	0.2662	2.07	0.93	3.83	0.2662
Age band	18-40	1,125	79	-0.43	-2.36	1.64	0.0825	2.00	0.95	3.66	0.0825
	40-60	1,075	81	-0.21	-2.17	1.85	0.7186	2.02	0.87	3.69	0.7186
	60 and above	1,227	75	0.62	-2.09	3.64	0.0099	2.85	1.34	5.01	0.0099
Years	1980-1989	457	72	-0.92	-2.94	1.37	0.1716	2.31	1.13	4.26	0.1716
	1990-1999	513	75	-0.45	-2.67	2.07	0.4553	2.36	1.27	4.06	0.4553
	2000-2009	1,741	79	0.29	-1.88	2.53	0.0111	2.22	0.98	3.93	0.0111
	2010-2014	716	82	-0.33	-2.44	2.24	0.4852	2.32	1.00	4.47	0.4852
Data density	Data poor	*Not applicable for source-based test*									
	Average data density	566	81	-0.37	-2.99	2.03	0.1547	2.45	1.15	4.35	0.1547
	Data rich	2,861	77	0.00	-2.10	2.36	0.0369	2.22	1.02	4.09	0.0369
Hold out pattern	Post-2000 data removed	1,325	81	0.56	-1.62	2.87	0.0001	2.20	0.96	4.00	0.0001
	Random set of data removed	2,102	76	-0.47	-2.65	2.01	0.0889	2.31	1.10	4.15	0.0889

Prevalence of raised blood pressure, Test 1, Men											
**Data**		**No. of held out observations**	**Percent covered**	**Error (percentage point) †**				**Absolute error (percentage point)**			
				**Median**	**Q1**	**Q3**	**(p*)**	**Median**	**Q1**	**Q3**	**(p*)**
All		3,318	99	-0.60	-7.90	6.57	0.7795	7.19	3.38	13.06	0.7795
Super-region	Central and Eastern Europe	219	99	-2.70	-11.61	2.01	0.0130	6.09	2.55	12.86	0.0130
	Central Asia, Middle East and North Africa	502	98	1.54	-6.02	7.19	0.3717	6.75	3.13	11.80	0.3717
	East and Southeast Asia	164	99	-0.68	-4.09	4.21	0.7821	4.13	2.39	8.60	0.7821
	High-income Asia Pacific	*No data from this region were among the held-out data*									
	High-income Western countries	1,666	99	-1.36	-8.77	6.12	0.2802	7.55	3.52	13.01	0.2802
	Latin America and Caribbean	342	99	1.96	-5.71	9.84	0.0579	7.79	3.95	14.61	0.0579
	Oceania	95	93	0.34	-7.43	9.64	0.5705	8.66	4.01	15.12	0.5705
	South Asia	42	100	-9.98	-16.01	-5.11	0.0268	10.21	7.26	17.16	0.0268
	Sub-Saharan Africa	288	99	0.70	-6.52	7.51	0.5840	7.06	3.78	13.55	0.5840
Urban or rural studies	Rural	385	97	-2.66	-11.89	6.15	0.4166	8.95	4.85	15.86	0.4166
	Urban	1,024	99	0.49	-7.68	8.63	0.3872	8.23	3.85	13.97	0.3872
	Both rural and urban	1,909	99	-0.72	-7.23	5.62	0.4675	6.35	2.98	12.05	0.4675
Study representative-ness	Community	1,297	98	-1.43	-10.35	7.37	0.3142	8.73	3.88	14.91	0.3142
	Subnational	661	98	-0.39	-8.49	6.60	0.8816	7.42	3.69	13.88	0.8816
	National	1,360	99	-0.17	-6.15	6.05	0.8826	6.10	2.84	10.81	0.8826
Age band	18-40	1,032	97	-0.94	-6.31	4.42	0.0422	5.36	2.57	9.12	0.0422
	40-60	1,060	100	-0.21	-7.70	7.33	0.7164	7.52	3.44	13.25	0.7164
	60 and above	1,226	99	-0.81	-9.52	9.18	0.7189	9.41	4.39	16.01	0.7189
Years	1980-1989	585	99	0.47	-8.69	8.59	0.5485	8.67	3.84	14.32	0.5485
	1990-1999	743	100	-2.95	-10.58	5.07	0.0449	8.51	3.87	14.36	0.0449
	2000-2009	1,444	99	-0.57	-7.21	6.17	0.6649	6.63	3.17	12.02	0.6649
	2010-2014	546	97	0.76	-4.89	7.16	0.1457	6.18	3.10	11.95	0.1457
Data density	Data poor	147	99	-2.02	-7.30	4.62	0.4127	5.88	3.22	11.66	0.4127
	Average data density	580	98	-3.33	-10.65	3.96	0.0171	7.28	3.62	13.47	0.0171
	Data rich	2,591	99	0.05	-7.29	7.19	0.3380	7.23	3.32	13.06	0.3380

Prevalence of raised blood pressure, Test 1, Women											
Data		No. of held out observations	Percent covered	Error (percentage point) †				Absolute error (percentage point)			
				Median	Q1	Q3	(p*)	Median	Q1	Q3	(p*)
All		2,720	96	0.91	-4.67	6.86	0.0147	5.86	2.45	12.04	0.0147
Super-region	Central and Eastern Europe	164	100	4.91	0.44	12.95	0.0214	6.70	2.62	13.49	0.0214
	Central Asia, Middle East and North Africa	255	99	-0.07	-6.38	5.06	0.8532	5.76	2.41	12.71	0.8532
	East and Southeast Asia	477	95	0.23	-7.60	5.12	0.9380	5.67	2.39	12.45	0.9380
	High-income Asia Pacific	120	93	3.71	0.99	13.70	0.0070	4.14	1.55	13.79	0.0070
	High-income Western countries	873	95	1.89	-3.84	7.63	0.0089	6.02	2.64	11.63	0.0089
	Latin America and Caribbean	323	97	-0.18	-4.34	4.98	0.6287	4.54	2.18	10.19	0.6287
	Oceania	132	94	-0.37	-4.90	6.97	0.6500	5.77	2.53	11.57	0.6500
	South Asia	59	98	-4.69	-10.77	1.11	0.2939	9.26	4.68	13.73	0.2939
	Sub-Saharan Africa	317	99	-1.01	-7.43	5.54	0.7470	6.35	2.68	11.82	0.7470
Urban or rural studies	Rural	378	94	2.40	-4.22	10.19	0.0587	7.43	3.20	14.38	0.0587
	Urban	778	95	1.09	-4.82	6.81	0.1067	6.01	2.70	12.49	0.1067
	Both rural and urban	1,564	97	0.63	-4.56	6.15	0.2738	5.41	2.27	11.19	0.2738
Study representative-ness	Community	1,071	95	0.20	-6.61	6.83	0.3207	6.77	2.87	13.77	0.3207
	Subnational	440	96	2.40	-4.47	8.46	0.0782	6.37	3.22	12.62	0.0782
	National	1,209	98	0.93	-3.42	6.25	0.1092	4.99	1.93	10.43	0.1092
Age band	18-40	909	90	0.23	-3.48	2.75	0.5680	2.98	1.30	5.86	0.5680
	40-60	882	100	1.43	-5.01	8.87	0.0015	6.93	3.12	12.89	0.0015
	60 and above	929	99	2.63	-6.79	12.16	0.0003	9.89	4.42	16.28	0.0003
Years	1980-1989	454	91	2.94	-4.48	8.44	0.0060	6.60	3.44	13.01	0.0060
	1990-1999	609	99	1.36	-4.41	7.33	0.1573	6.07	2.64	12.55	0.1573
	2000-2009	1,181	97	0.86	-4.21	6.62	0.2002	5.42	2.22	12.06	0.2002
	2010-2014	476	97	-0.40	-6.28	4.35	0.8006	5.54	2.02	10.48	0.8006
Data density	Data poor	152	98	-2.19	-7.63	3.05	0.3532	6.61	2.85	10.23	0.3532
	Average data density	547	98	-1.45	-7.31	4.21	0.4059	6.11	2.54	12.25	0.4059
	Data rich	2,021	96	1.54	-3.52	7.60	0.0006	5.80	2.42	12.06	0.0006

Prevalence of raised blood pressure, Test 2, Men											
**Data**		**No. of held out observations**	**Percent covered**	**Error (percentage point) †**				**Absolute error (percentage point)**			
				**Median**	**Q1**	**Q3**	**(p*)**	**Median**	**Q1**	**Q3**	**(p*)**
All		3,514	98	0.43	-5.57	7.50	0.0061	6.53	2.92	12.17	0.0061
Super-region	Central and Eastern Europe	369	99	-0.26	-6.50	8.16	0.6630	7.12	3.65	14.65	0.6630
	Central Asia, Middle East and North Africa	442	98	1.82	-5.66	7.26	0.2945	6.70	3.57	11.83	0.2945
	East and Southeast Asia	352	97	-0.97	-6.91	5.19	0.6454	6.27	2.83	11.44	0.6454
	High-income Asia Pacific	362	98	0.79	-4.28	10.10	0.0147	5.87	2.70	13.33	0.0147
	High-income Western countries	1,127	99	0.43	-5.81	7.21	0.1872	6.41	2.81	11.38	0.1872
	Latin America and Caribbean	282	99	1.80	-4.90	9.13	0.1540	6.85	2.88	13.17	0.1540
	Oceania	108	93	-1.65	-7.34	6.84	0.8421	7.01	3.60	12.28	0.8421
	South Asia	192	98	-0.41	-5.09	5.76	0.7472	5.33	2.38	9.50	0.7472
	Sub-Saharan Africa	280	99	1.69	-3.91	8.82	0.1318	7.11	2.97	13.98	0.1318
Urban or rural studies	Rural	509	96	1.52	-7.03	9.29	0.1221	8.44	3.88	14.77	0.1221
	Urban	898	98	0.54	-6.35	8.18	0.2495	7.44	3.32	12.80	0.2495
	Both rural and urban	2,107	99	0.24	-5.06	6.70	0.0609	5.87	2.66	11.27	0.0609
Study representative-ness	Community	1,348	97	0.26	-7.41	8.17	0.3631	7.85	3.49	13.85	0.3631
	Subnational	551	97	0.96	-6.41	8.18	0.0985	7.09	3.53	12.69	0.0985
	National	1,615	99	0.37	-4.49	6.71	0.0546	5.43	2.44	10.31	0.0546
Age band	18-40	1,130	96	0.00	-4.48	4.65	0.6376	4.58	2.01	7.90	0.6376
	40-60	1,103	100	0.21	-6.07	7.56	0.1083	6.92	3.10	12.74	0.1083
	60 and above	1,281	99	1.41	-6.65	11.60	0.0000	9.14	4.26	15.51	0.0000
Years	1980-1989	558	98	-1.09	-6.81	7.41	0.6957	7.05	3.10	12.58	0.6957
	1990-1999	443	99	0.78	-7.53	7.54	0.6546	7.57	3.69	12.34	0.6546
	2000-2009	1,786	98	0.29	-5.46	7.12	0.1883	6.33	2.78	12.10	0.1883
	2010-2014	727	98	1.82	-3.88	8.74	0.0021	6.25	2.76	12.12	0.0021
Data density	Data poor	*Not applicable for source-based test*									
	Average data density	545	98	-1.48	-8.52	5.35	0.3163	6.86	3.15	12.14	0.3163
	Data rich	2,969	98	0.86	-5.12	7.83	0.0007	6.48	2.90	12.18	0.0007
Hold out pattern	Post-2000 data removed	1,414	99	1.45	-4.01	8.50	0.0003	6.31	2.76	12.12	0.0003
	Random set of data removed	2,100	98	-0.28	-6.57	6.86	0.5604	6.73	3.10	12.26	0.5604

Prevalence of raised blood pressure, Test 2, Women											
**Data**		**No. of held out observations**	**Percent covered**	**Error (percentage point) †**				**Absolute error (percentage point)**			
				**Median**	**Q1**	**Q3**	**(p*)**	**Median**	**Q1**	**Q3**	**(p*)**
All		3,435	96	0.07	-5.26	4.98	0.3235	5.10	1.98	10.80	0.3235
Super-region	Central and Eastern Europe	283	98	-0.74	-7.10	3.33	0.3206	4.99	2.49	9.75	0.3206
	Central Asia, Middle East and North Africa	423	99	1.70	-3.01	7.09	0.0444	5.62	2.32	10.97	0.0444
	East and Southeast Asia	342	96	-0.10	-6.81	5.43	0.9406	6.27	2.61	11.92	0.9406
	High-income Asia Pacific	370	92	0.50	-5.43	5.79	0.3386	5.48	1.52	11.81	0.3386
	High-income Western countries	1,136	95	-0.23	-5.13	3.89	0.8488	4.55	1.76	10.06	0.8488
	Latin America and Caribbean	310	97	-0.17	-3.90	3.86	0.6683	3.89	1.72	10.06	0.6683
	Oceania	111	91	-1.35	-6.78	4.11	0.8953	5.44	2.45	13.52	0.8953
	South Asia	194	98	0.91	-3.11	7.75	0.3078	4.53	1.59	10.73	0.3078
	Sub-Saharan Africa	266	99	-0.84	-7.46	5.96	0.9073	6.61	2.50	11.77	0.9073
Urban or rural studies	Rural	512	93	0.24	-7.00	5.71	0.5921	6.11	2.39	13.09	0.5921
	Urban	894	97	-0.76	-7.13	3.93	0.5264	5.65	2.41	11.96	0.5264
	Both rural and urban	2,029	97	0.23	-4.16	5.24	0.2031	4.70	1.72	10.01	0.2031
Study representative-ness	Community	1,360	95	-0.66	-7.99	4.67	0.8049	6.35	2.50	13.07	0.8049
	Subnational	490	95	0.19	-5.02	3.59	0.9911	4.24	2.00	9.70	0.9911
	National	1,585	97	0.36	-3.58	5.52	0.1424	4.49	1.59	9.68	0.1424
Age band	18-40	1,126	90	-0.24	-3.06	1.74	0.1434	2.20	0.99	4.40	0.1434
	40-60	1,075	100	-0.22	-6.40	5.91	0.9027	6.14	2.55	10.66	0.9027
	60 and above	1,234	99	1.78	-7.64	11.45	0.0004	9.67	4.23	15.39	0.0004
Years	1980-1989	458	95	-3.37	-10.26	2.30	0.0715	6.57	2.58	12.56	0.0715
	1990-1999	513	97	-0.18	-7.43	5.64	0.7460	6.10	2.43	11.64	0.7460
	2000-2009	1,745	97	0.71	-3.88	5.97	0.0365	4.70	1.86	10.59	0.0365
	2010-2014	719	95	-0.02	-4.37	4.85	0.5867	4.49	1.70	9.97	0.5867
Data density	Data poor	*Not applicable for source-based test*									
	Average data density	568	97	-0.49	-6.69	4.57	0.7481	5.99	2.39	11.12	0.7481
	Data rich	2,867	96	0.14	-4.96	5.06	0.2342	5.00	1.92	10.72	0.2342
Hold out pattern	Post-2000 data removed	1,329	96	1.06	-2.51	6.65	0.0043	4.27	1.67	10.06	0.0043
	Random set of data removed	2,106	96	-0.91	-7.28	3.89	0.2895	5.70	2.29	11.09	0.2895

† Estimated values minus held out values.
* p-values for model error comparisons were calculated using the non-parametric Wilcoxon signed-rank test for paired data. The p-values are calculated assuming independence of the held-out observations. They should therefore be interpreted as an approximation because there is some dependence among the held-out observations, within each of the five repetitions for example.

Appendix Table 5: Mean systolic blood pressure, mean diastolic blood pressure and prevalence of raised blood pressure by sex and age group for the world and nine super-regions in 1975, 1985, 1995, 2005 and 2015. Numbers in brackets show 95% credible intervals.

MENA: Middle East and North Africa

Mean systolic blood pressure (mmHg)					
**Super-region (Men)**	**Year**				
	**1975**	**1985**	**1995**	**2005**	**2015**
**18-19 years**					
World	117.6 (115.4-119.9)	117.4 (116.2-118.5)	117.7 (117.0-118.5)	118.2 (117.6-118.7)	118.9 (117.9-120.0)
Central & Eastern Europe	122.3 (118.9-125.8)	122.3 (120.6-124.0)	121.5 (120.0-123.0)	121.3 (119.6-122.8)	121.9 (119.5-124.4)
Central Asia & MENA	117.9 (112.9-122.6)	117.9 (115.2-120.4)	117.6 (116.0-119.1)	117.8 (116.5-119.1)	118.3 (116.3-120.3)
East & Southeast Asia	113.9 (110.2-117.6)	114.3 (112.8-115.8)	115.1 (113.9-116.2)	116.0 (115.0-116.9)	117.3 (115.2-119.4)
High-income Asia Pacific	121.0 (118.3-123.8)	119.3 (117.8-120.8)	117.9 (116.7-119.1)	116.2 (115.2-117.2)	114.3 (112.3-116.3)
High-income western	124.0 (122.0-126.0)	122.6 (121.5-123.6)	120.5 (119.7-121.4)	119.3 (118.5-120.2)	118.3 (116.6-120.0)
Latin America & Caribbean	120.5 (116.1-124.7)	120.3 (118.0-122.6)	119.6 (118.1-121.0)	119.4 (118.3-120.4)	120.3 (118.4-122.3)
Oceania	119.2 (114.1-124.0)	120.3 (117.6-123.1)	121.4 (118.2-124.6)	122.4 (119.3-125.5)	123.7 (118.6-128.6)
South Asia	116.0 (109.9-121.7)	116.8 (113.6-119.7)	117.7 (115.7-119.5)	119.1 (117.8-120.5)	119.8 (117.5-122.1)
Sub-Saharan Africa	117.9 (114.5-121.7)	118.8 (116.9-120.8)	119.3 (117.8-120.7)	119.6 (118.4-120.9)	119.6 (117.6-121.5)
**20-24 years**					
World	118.7 (116.4-121.0)	118.7 (117.5-119.8)	118.6 (117.9-119.3)	119.3 (118.8-119.9)	119.9 (118.9-121.0)
Central & Eastern Europe	123.9 (120.6-127.4)	124.1 (122.5-125.7)	123.2 (121.8-124.5)	122.9 (121.4-124.4)	123.4 (121.0-125.9)
Central Asia & MENA	118.8 (113.7-123.6)	118.9 (116.2-121.4)	118.6 (117.1-120.1)	118.7 (117.5-119.8)	119.3 (117.4-121.2)
East & Southeast Asia	114.7 (110.9-118.6)	115.2 (113.7-116.7)	115.9 (114.8-117.0)	117.0 (116.1-117.9)	118.3 (116.1-120.6)
High-income Asia Pacific	123.3 (120.9-125.8)	120.8 (119.3-122.3)	119.4 (118.2-120.6)	117.7 (116.7-118.6)	115.6 (113.6-117.6)
High-income western	125.0 (123.0-127.0)	123.4 (122.4-124.4)	121.7 (120.9-122.4)	120.2 (119.4-121.0)	119.1 (117.4-120.8)
Latin America & Caribbean	121.7 (117.3-126.0)	121.6 (119.3-123.9)	120.7 (119.4-122.1)	120.6 (119.6-121.7)	121.4 (119.5-123.4)
Oceania	119.3 (114.2-124.1)	120.5 (117.9-123.0)	121.5 (118.5-124.5)	122.6 (119.6-125.5)	123.9 (118.8-128.7)
South Asia	116.9 (110.7-122.9)	117.7 (114.6-120.7)	118.7 (116.8-120.5)	120.1 (118.8-121.5)	120.9 (118.5-123.2)
Sub-Saharan Africa	119.3 (115.8-123.1)	120.2 (118.2-122.2)	120.6 (119.1-122.0)	121.0 (119.8-122.1)	120.9 (119.0-122.8)
**25-29 years**					
World	120.3 (118.1-122.6)	120.0 (118.9-121.2)	119.8 (119.1-120.5)	120.6 (120.0-121.1)	121.1 (120.0-122.2)
Central & Eastern Europe	125.9 (122.5-129.4)	125.9 (124.4-127.5)	125.0 (123.7-126.3)	124.8 (123.4-126.2)	125.2 (122.6-127.8)
Central Asia & MENA	119.9 (114.7-124.7)	120.1 (117.4-122.6)	119.8 (118.3-121.2)	119.8 (118.7-120.8)	120.4 (118.6-122.2)
East & Southeast Asia	115.8 (112.0-119.8)	116.4 (114.8-117.9)	117.0 (115.9-118.1)	118.2 (117.3-119.1)	119.5 (117.1-121.9)
High-income Asia Pacific	125.7 (123.5-128.1)	122.5 (121.0-124.0)	121.1 (119.9-122.3)	119.3 (118.4-120.3)	117.4 (115.3-119.5)
High-income western	126.0 (124.0-128.1)	124.2 (123.1-125.3)	122.9 (122.1-123.6)	121.2 (120.5-122.0)	120.0 (118.2-121.6)
Latin America & Caribbean	123.1 (118.6-127.5)	122.9 (120.6-125.3)	122.1 (120.7-123.4)	121.9 (120.9-122.9)	122.8 (120.9-124.8)
Oceania	119.1 (113.9-124.1)	120.4 (118.0-122.9)	121.5 (118.7-124.4)	122.5 (119.7-125.4)	123.9 (118.8-128.9)
South Asia	117.8 (111.5-123.8)	118.7 (115.6-121.8)	119.7 (117.8-121.5)	121.1 (119.9-122.5)	121.9 (119.5-124.3)
Sub-Saharan Africa	120.8 (117.2-124.7)	121.7 (119.8-123.8)	122.2 (120.7-123.6)	122.5 (121.4-123.7)	122.4 (120.5-124.3)
**30-34 years**					
World	121.4 (119.1-123.8)	121.1 (119.9-122.3)	121.0 (120.3-121.7)	121.4 (120.9-122.0)	122.2 (121.1-123.2)
Central & Eastern Europe	127.6 (124.2-131.1)	127.6 (126.0-129.2)	126.7 (125.2-128.0)	126.5 (125.0-127.9)	126.9 (124.3-129.5)
Central Asia & MENA	120.9 (115.6-125.9)	121.3 (118.5-123.7)	121.0 (119.6-122.4)	121.0 (119.9-122.0)	121.3 (119.5-123.2)
East & Southeast Asia	117.0 (113.0-121.0)	117.4 (115.8-118.9)	118.1 (117.0-119.2)	119.2 (118.3-120.2)	120.8 (118.4-123.2)
High-income Asia Pacific	127.3 (124.9-129.7)	124.4 (123.0-125.8)	122.4 (121.2-123.7)	120.8 (119.7-121.8)	118.7 (116.6-120.8)
High-income western	127.1 (125.0-129.2)	125.1 (124.0-126.2)	123.5 (122.7-124.3)	122.2 (121.4-122.9)	120.7 (119.0-122.4)
Latin America & Caribbean	124.3 (119.6-128.8)	124.1 (121.7-126.5)	123.3 (121.9-124.7)	123.0 (122.0-124.0)	124.2 (122.2-126.3)
Oceania	119.0 (113.6-124.1)	120.4 (117.9-122.9)	121.6 (118.8-124.4)	122.5 (119.7-125.4)	123.9 (118.8-129.0)
South Asia	118.5 (112.1-124.7)	119.5 (116.3-122.7)	120.5 (118.5-122.3)	122.0 (120.6-123.2)	122.7 (120.2-125.1)
Sub-Saharan Africa	122.2 (118.4-126.2)	123.1 (121.1-125.2)	123.6 (122.2-125.0)	123.9 (122.7-125.0)	123.7 (121.8-125.6)
**35-39 years**					
World	122.9 (120.5-125.3)	122.7 (121.5-123.8)	122.4 (121.7-123.1)	122.6 (122.0-123.2)	123.4 (122.3-124.5)

Mean systolic blood pressure (mmHg)					
Super-region (Men)	Year				
	1975	1985	1995	2005	2015
Central & Eastern Europe	129.3 (125.6-133.2)	129.4 (127.9-131.0)	128.5 (127.1-129.8)	128.3 (126.8-129.7)	128.8 (126.2-131.4)
Central Asia & MENA	122.6 (117.2-127.6)	122.6 (119.9-125.2)	122.4 (120.9-123.8)	122.3 (121.3-123.4)	122.6 (120.7-124.5)
East & Southeast Asia	118.3 (114.2-122.5)	118.8 (117.2-120.4)	119.6 (118.4-120.7)	120.6 (119.6-121.6)	122.3 (119.8-124.7)
High-income Asia Pacific	128.9 (126.4-131.4)	126.3 (125.0-127.7)	123.9 (122.6-125.2)	122.1 (121.1-123.1)	120.2 (118.0-122.4)
High-income western	128.4 (126.3-130.7)	126.4 (125.2-127.4)	124.3 (123.5-125.1)	123.3 (122.6-124.1)	121.8 (120.0-123.5)
Latin America & Caribbean	125.7 (120.9-130.4)	125.5 (123.0-128.0)	124.6 (123.2-126.1)	124.3 (123.3-125.4)	125.4 (123.4-127.6)
Oceania	119.2 (113.7-124.5)	120.6 (118.0-123.2)	121.9 (119.0-124.8)	122.9 (120.2-125.7)	124.2 (118.9-129.5)
South Asia	119.3 (112.7-125.7)	120.3 (116.9-123.5)	121.4 (119.4-123.2)	122.8 (121.5-124.1)	123.6 (121.0-126.1)
Sub-Saharan Africa	123.7 (119.8-127.9)	124.6 (122.5-126.8)	125.2 (123.6-126.6)	125.4 (124.2-126.6)	125.2 (123.3-127.2)
**40-44 years**					
World	124.6 (122.1-127.2)	124.4 (123.2-125.7)	124.1 (123.4-124.9)	124.4 (123.8-125.0)	125.0 (123.8-126.3)
Central & Eastern Europe	131.7 (128.0-135.6)	131.9 (130.2-133.6)	130.8 (129.3-132.2)	130.4 (128.8-132.0)	131.1 (128.4-133.8)
Central Asia & MENA	124.2 (118.5-129.6)	124.5 (121.6-127.1)	124.3 (122.8-125.8)	124.4 (123.3-125.4)	124.5 (122.5-126.4)
East & Southeast Asia	120.1 (115.8-124.5)	120.7 (119.0-122.4)	121.3 (120.2-122.5)	122.6 (121.5-123.6)	124.2 (121.5-126.9)
High-income Asia Pacific	131.4 (128.9-134.0)	128.4 (127.0-129.8)	126.1 (124.9-127.4)	123.8 (122.8-124.9)	122.0 (119.7-124.3)
High-income western	130.1 (127.8-132.4)	128.3 (127.2-129.4)	126.0 (125.1-126.8)	124.7 (123.9-125.5)	123.3 (121.5-125.1)
Latin America & Caribbean	127.5 (122.5-132.4)	127.3 (124.7-129.9)	126.4 (124.9-127.9)	126.2 (125.1-127.3)	127.2 (125.1-129.4)
Oceania	119.9 (114.2-125.5)	121.3 (118.6-124.0)	122.7 (119.7-125.8)	123.9 (121.0-126.8)	125.1 (119.5-130.7)
South Asia	120.5 (113.4-127.2)	121.4 (117.9-124.8)	122.6 (120.5-124.6)	124.1 (122.7-125.5)	124.9 (122.2-127.5)
Sub-Saharan Africa	125.6 (121.6-130.0)	126.7 (124.5-128.9)	127.2 (125.6-128.7)	127.5 (126.2-128.7)	127.2 (125.1-129.2)
**45-49 years**					
World	127.5 (124.9-130.2)	127.3 (126.0-128.6)	126.9 (126.1-127.7)	127.1 (126.4-127.7)	127.5 (126.2-128.9)
Central & Eastern Europe	134.9 (130.8-139.1)	135.0 (133.1-136.8)	134.0 (132.4-135.4)	133.6 (131.9-135.3)	134.4 (131.5-137.2)
Central Asia & MENA	127.2 (121.1-132.9)	127.4 (124.3-130.3)	127.0 (125.4-128.6)	127.1 (126.0-128.3)	127.3 (125.3-129.4)
East & Southeast Asia	122.7 (118.1-127.4)	123.3 (121.5-125.1)	124.0 (122.8-125.3)	125.3 (124.3-126.4)	127.1 (124.1-129.9)
High-income Asia Pacific	134.7 (132.1-137.4)	131.2 (129.6-132.7)	129.0 (127.7-130.3)	126.3 (125.2-127.5)	124.1 (121.7-126.6)
High-income western	132.7 (130.3-135.2)	131.3 (130.1-132.5)	128.6 (127.7-129.5)	126.8 (125.9-127.7)	125.6 (123.7-127.5)
Latin America & Caribbean	130.3 (124.8-135.5)	130.0 (127.2-132.8)	129.0 (127.4-130.6)	128.8 (127.6-130.0)	129.8 (127.6-132.2)
Oceania	121.3 (115.2-127.4)	122.8 (119.8-125.7)	124.3 (121.0-127.6)	125.6 (122.6-128.7)	127.0 (121.2-132.7)
South Asia	122.2 (114.7-129.5)	123.3 (119.4-126.9)	124.5 (122.3-126.5)	126.2 (124.7-127.7)	127.0 (124.0-129.8)
Sub-Saharan Africa	128.3 (123.9-133.0)	129.4 (127.0-131.9)	129.9 (128.2-131.6)	130.3 (128.9-131.6)	130.0 (127.8-132.1)
**50-54 years**					
World	130.7 (127.9-133.5)	130.4 (129.0-131.8)	130.0 (129.2-130.9)	130.2 (129.5-130.9)	130.8 (129.4-132.3)
Central & Eastern Europe	139.0 (134.8-143.4)	139.0 (137.0-140.9)	138.0 (136.3-139.6)	137.5 (135.7-139.3)	138.0 (134.8-141.3)
Central Asia & MENA	130.5 (123.8-136.8)	130.7 (127.3-133.9)	130.4 (128.7-132.2)	130.6 (129.3-131.8)	131.0 (128.7-133.2)
East & Southeast Asia	125.9 (120.8-131.0)	126.5 (124.5-128.4)	127.4 (126.0-128.8)	128.7 (127.4-129.9)	130.6 (127.4-133.8)
High-income Asia Pacific	138.1 (135.1-141.1)	134.9 (133.2-136.5)	132.3 (130.9-133.7)	129.8 (128.5-131.1)	126.8 (124.2-129.4)
High-income western	136.2 (133.5-138.9)	134.5 (133.2-135.8)	132.2 (131.2-133.2)	129.9 (128.9-130.8)	128.4 (126.2-130.4)
Latin America & Caribbean	133.6 (127.7-139.3)	133.2 (130.2-136.2)	132.2 (130.4-133.9)	131.9 (130.7-133.2)	133.2 (130.8-135.9)
Oceania	123.4 (116.6-129.9)	124.9 (121.6-128.2)	126.5 (122.9-130.2)	127.9 (124.6-131.4)	129.6 (123.5-135.6)
South Asia	124.5 (116.3-132.4)	125.6 (121.5-129.6)	126.9 (124.5-129.2)	128.9 (127.3-130.5)	129.7 (126.5-132.8)
Sub-Saharan Africa	131.6 (126.8-136.7)	132.7 (130.2-135.4)	133.4 (131.5-135.2)	133.7 (132.3-135.2)	133.4 (131.1-135.8)
**55-59 years**					
World	133.5 (130.4-136.7)	134.1 (132.6-135.6)	133.7 (132.8-134.6)	133.7 (133.0-134.4)	134.2 (132.7-135.8)
Central & Eastern Europe	143.0 (138.3-147.8)	143.0 (140.8-145.2)	141.7 (139.8-143.6)	141.4 (139.5-143.3)	142.0 (138.6-145.5)
Central Asia & MENA	134.2 (126.9-141.1)	134.6 (130.8-138.0)	134.3 (132.3-136.2)	134.2 (132.9-135.6)	134.6 (132.2-137.0)

Mean systolic blood pressure (mmHg)					
**Super-region (Men)**	**Year**				
	**1975**	**1985**	**1995**	**2005**	**2015**
East & Southeast Asia	129.2 (123.6-134.7)	129.8 (127.7-132.0)	130.8 (129.4-132.3)	132.3 (131.0-133.6)	134.3 (130.9-137.7)
High-income Asia Pacific	141.9 (138.7-145.2)	138.7 (136.9-140.4)	135.6 (134.0-137.1)	133.2 (131.7-134.6)	129.7 (126.9-132.6)
High-income western	139.4 (136.6-142.3)	137.9 (136.5-139.3)	136.0 (135.0-137.1)	133.3 (132.3-134.3)	131.3 (128.9-133.6)
Latin America & Caribbean	137.1 (130.6-143.2)	136.7 (133.4-140.0)	135.5 (133.6-137.5)	135.2 (133.8-136.6)	136.6 (134.0-139.5)
Oceania	125.7 (118.3-132.7)	127.3 (123.6-130.8)	128.8 (124.9-133.0)	130.4 (126.7-134.3)	132.4 (125.9-138.8)
South Asia	126.9 (118.1-135.5)	128.2 (123.7-132.5)	129.5 (127.0-132.0)	131.6 (129.9-133.3)	132.7 (129.2-136.0)
Sub-Saharan Africa	134.9 (129.8-140.5)	136.2 (133.4-139.1)	136.9 (134.9-138.9)	137.3 (135.7-138.8)	136.9 (134.4-139.5)
**60-64 years**					
World	137.5 (134.3-140.7)	136.9 (135.3-138.5)	136.5 (135.6-137.5)	136.6 (135.8-137.3)	137.2 (135.5-139.0)
Central & Eastern Europe	146.4 (141.5-151.6)	146.6 (144.3-148.9)	145.1 (143.2-147.0)	145.0 (143.0-146.9)	145.5 (141.9-149.2)
Central Asia & MENA	137.4 (129.7-144.6)	137.7 (133.7-141.3)	137.3 (135.3-139.4)	137.4 (136.0-138.8)	137.8 (135.1-140.4)
East & Southeast Asia	132.1 (126.3-138.1)	132.8 (130.6-135.1)	133.9 (132.2-135.5)	135.4 (134.0-136.8)	137.6 (133.7-141.4)
High-income Asia Pacific	145.6 (142.4-149.0)	141.5 (139.6-143.3)	138.6 (136.9-140.2)	135.8 (134.3-137.4)	133.1 (129.9-136.3)
High-income western	143.2 (140.1-146.4)	140.9 (139.4-142.4)	138.9 (137.8-140.0)	136.4 (135.4-137.5)	134.1 (131.5-136.5)
Latin America & Caribbean	140.1 (133.2-146.6)	139.6 (136.1-143.0)	138.3 (136.2-140.3)	137.9 (136.4-139.4)	139.4 (136.6-142.5)
Oceania	127.7 (119.9-135.2)	129.3 (125.3-133.2)	131.0 (126.8-135.5)	132.6 (128.6-136.8)	134.7 (127.7-141.5)
South Asia	129.0 (119.7-138.2)	130.4 (125.6-135.0)	131.9 (129.1-134.5)	134.0 (132.2-135.9)	135.3 (131.5-138.9)
Sub-Saharan Africa	137.8 (132.3-143.7)	139.1 (136.1-142.2)	139.8 (137.7-142.0)	140.3 (138.6-142.0)	140.0 (137.2-142.8)
**65-69 years**					
World	140.2 (137.0-143.5)	138.7 (137.0-140.3)	139.0 (138.0-140.0)	139.1 (138.3-139.9)	139.4 (137.7-141.1)
Central & Eastern Europe	149.0 (143.9-154.3)	149.0 (146.5-151.5)	147.6 (145.4-149.7)	147.2 (144.8-149.4)	148.0 (144.3-151.6)
Central Asia & MENA	140.1 (132.1-147.7)	140.3 (136.1-144.2)	140.0 (137.8-142.3)	140.1 (138.5-141.6)	140.3 (137.6-143.1)
East & Southeast Asia	134.5 (128.3-140.7)	135.2 (132.8-137.6)	136.3 (134.6-138.0)	137.9 (136.4-139.3)	140.2 (136.1-144.2)
High-income Asia Pacific	148.4 (145.0-151.9)	143.8 (141.8-145.7)	140.8 (139.1-142.5)	137.8 (136.1-139.3)	135.6 (132.0-139.2)
High-income western	145.8 (142.6-149.2)	142.7 (141.0-144.2)	141.0 (139.9-142.2)	139.0 (137.9-140.1)	136.3 (133.8-138.9)
Latin America & Caribbean	142.3 (135.0-149.0)	141.8 (138.1-145.4)	140.5 (138.3-142.6)	140.0 (138.5-141.6)	141.5 (138.6-144.8)
Oceania	129.2 (121.1-137.1)	131.0 (126.7-135.2)	132.6 (128.1-137.4)	134.2 (129.9-138.8)	136.3 (129.0-143.5)
South Asia	130.7 (120.8-140.3)	132.1 (127.1-136.9)	133.7 (130.7-136.5)	135.9 (134.0-137.9)	137.2 (133.3-140.9)
Sub-Saharan Africa	139.8 (134.2-146.0)	141.3 (138.1-144.5)	142.1 (139.8-144.3)	142.6 (140.8-144.3)	142.2 (139.3-145.2)
**70-74 years**					
World	142.1 (138.9-145.5)	141.3 (139.6-142.9)	140.5 (139.5-141.5)	140.6 (139.8-141.4)	140.8 (139.1-142.6)
Central & Eastern Europe	150.6 (145.3-156.1)	150.6 (148.1-153.3)	149.3 (147.1-151.4)	148.8 (146.6-151.0)	150.0 (146.2-153.7)
Central Asia & MENA	141.9 (133.6-149.8)	142.2 (137.8-146.3)	141.8 (139.5-144.1)	141.8 (140.1-143.4)	141.9 (139.1-144.8)
East & Southeast Asia	136.1 (129.6-142.6)	136.9 (134.3-139.4)	138.0 (136.2-139.7)	139.6 (138.1-141.2)	142.1 (137.9-146.2)
High-income Asia Pacific	150.6 (147.1-154.2)	145.6 (143.6-147.6)	142.1 (140.3-143.9)	139.2 (137.5-140.9)	136.9 (133.1-140.6)
High-income western	147.7 (144.3-151.2)	145.2 (143.5-146.9)	142.5 (141.3-143.8)	140.6 (139.4-141.7)	137.8 (135.1-140.4)
Latin America & Caribbean	143.5 (136.1-150.6)	143.1 (139.3-146.9)	141.7 (139.4-143.9)	141.2 (139.6-142.9)	142.7 (139.6-146.0)
Oceania	130.2 (122.0-138.4)	132.1 (127.5-136.7)	133.7 (128.8-138.8)	135.3 (130.5-140.2)	137.5 (129.9-144.8)
South Asia	131.9 (121.7-141.7)	133.3 (128.1-138.3)	134.9 (131.8-137.9)	137.3 (135.3-139.4)	138.6 (134.6-142.4)
Sub-Saharan Africa	141.1 (135.2-147.5)	142.6 (139.3-146.0)	143.4 (140.9-145.9)	144.0 (142.1-145.9)	143.8 (140.7-146.9)
**75-79 years**					
World	143.4 (140.2-146.8)	142.6 (140.9-144.3)	141.0 (139.9-142.1)	141.6 (140.8-142.4)	141.8 (140.1-143.6)
Central & Eastern Europe	151.4 (145.8-157.2)	151.4 (148.6-154.3)	149.9 (147.5-152.3)	149.5 (147.0-152.1)	150.5 (146.2-154.7)
Central Asia & MENA	143.1 (134.5-151.4)	143.4 (138.8-147.6)	143.0 (140.5-145.5)	143.2 (141.3-145.0)	143.2 (140.3-146.3)
East & Southeast Asia	137.0 (130.4-143.6)	137.8 (135.2-140.4)	138.9 (137.1-140.7)	140.6 (139.0-142.2)	143.1 (138.7-147.4)
High-income Asia Pacific	151.7 (148.1-155.4)	146.7 (144.6-148.8)	143.0 (141.2-144.8)	140.1 (138.3-141.9)	137.5 (133.6-141.4)

Mean systolic blood pressure (mmHg)					
Super-region (Men)	Year				
	1975	1985	1995	2005	2015
High-income western	148.8 (145.4-152.4)	146.3 (144.6-148.1)	142.7 (141.5-144.1)	141.4 (140.2-142.5)	138.7 (135.8-141.5)
Latin America & Caribbean	144.2 (136.5-151.6)	143.7 (139.7-147.5)	142.3 (139.9-144.7)	141.9 (140.1-143.7)	143.3 (140.1-146.7)
Oceania	131.3 (122.9-139.7)	132.9 (127.7-138.1)	134.4 (129.1-139.7)	135.7 (130.6-141.0)	137.9 (129.8-145.6)
South Asia	132.7 (122.1-142.8)	134.1 (128.7-139.2)	135.8 (132.6-138.8)	138.3 (136.1-140.4)	139.5 (135.4-143.4)
Sub-Saharan Africa	141.7 (135.6-148.3)	143.2 (139.7-146.7)	144.1 (141.4-146.7)	144.7 (142.7-146.7)	144.7 (141.4-148.0)
80-84 years					
World	144.3 (141.1-147.7)	143.0 (141.3-144.8)	142.0 (141.0-143.1)	141.7 (140.9-142.6)	141.9 (140.1-143.7)
Central & Eastern Europe	151.6 (145.4-157.9)	151.5 (148.3-154.7)	149.9 (147.2-152.6)	149.8 (147.2-152.4)	150.4 (146.4-154.6)
Central Asia & MENA	144.0 (135.2-152.4)	143.7 (139.0-148.3)	143.4 (140.7-146.1)	143.6 (141.6-145.5)	143.4 (140.2-146.6)
East & Southeast Asia	137.2 (130.3-144.0)	137.9 (135.2-140.6)	139.1 (137.2-141.0)	140.9 (139.2-142.6)	143.4 (138.8-147.9)
High-income Asia Pacific	152.4 (148.7-156.3)	147.4 (145.2-149.5)	143.4 (141.4-145.4)	140.3 (138.4-142.2)	138.1 (134.0-142.2)
High-income western	149.1 (145.6-152.8)	146.7 (144.8-148.6)	144.0 (142.7-145.4)	141.7 (140.4-142.9)	139.1 (136.2-141.9)
Latin America & Caribbean	144.1 (136.0-151.8)	143.3 (139.2-147.4)	142.0 (139.5-144.5)	141.6 (139.6-143.6)	143.1 (139.8-146.6)
Oceania	131.6 (122.7-140.3)	132.7 (126.9-138.6)	134.3 (128.3-140.3)	135.6 (130.0-141.5)	137.6 (129.3-145.8)
South Asia	133.2 (122.3-143.4)	134.5 (128.9-139.8)	136.3 (132.9-139.5)	138.8 (136.4-141.1)	140.0 (135.8-144.0)
Sub-Saharan Africa	141.7 (135.4-148.5)	143.0 (139.3-146.7)	144.0 (141.0-147.0)	144.6 (142.2-147.0)	144.8 (141.2-148.4)
85+ years					
World	144.5 (141.1-148.1)	142.5 (140.7-144.5)	141.4 (140.1-142.7)	140.7 (139.7-141.8)	140.9 (139.1-142.7)
Central & Eastern Europe	150.5 (143.8-157.4)	150.4 (146.5-154.3)	148.8 (145.3-152.3)	148.3 (145.1-151.5)	149.4 (144.6-154.2)
Central Asia & MENA	143.8 (134.2-152.8)	143.4 (137.8-148.7)	142.9 (139.5-146.2)	143.2 (140.7-145.8)	143.5 (139.9-147.2)
East & Southeast Asia	136.2 (129.2-143.4)	136.8 (133.8-139.8)	138.1 (135.8-140.3)	139.9 (137.8-141.9)	142.6 (138.0-147.2)
High-income Asia Pacific	152.9 (148.8-157.1)	147.4 (144.9-150.0)	143.3 (140.9-145.7)	140.1 (137.8-142.4)	138.1 (133.5-142.6)
High-income western	148.8 (145.1-152.7)	145.9 (143.7-147.9)	143.2 (141.6-144.8)	140.7 (139.2-142.1)	138.3 (135.3-141.3)
Latin America & Caribbean	142.5 (134.3-150.6)	141.9 (137.4-146.4)	140.5 (137.6-143.4)	140.4 (137.9-142.9)	142.2 (138.2-146.3)
Oceania	128.4 (117.6-139.1)	131.2 (124.1-138.6)	132.9 (125.5-140.3)	134.4 (127.3-141.7)	136.2 (126.8-145.5)
South Asia	133.4 (122.0-144.3)	134.8 (128.6-140.6)	136.5 (132.7-140.2)	139.0 (136.2-141.9)	140.2 (135.5-144.8)
Sub-Saharan Africa	140.7 (134.2-147.8)	141.9 (137.9-146.0)	142.9 (139.4-146.3)	143.7 (140.5-147.1)	144.0 (139.9-148.3)

Mean systolic blood pressure (mmHg)					
**Super-region (Women)**	**Year**				
	**1975**	**1985**	**1995**	**2005**	**2015**
**18-19 years**					
World	111.6 (109.6-113.6)	111.4 (110.4-112.4)	111.8 (111.1-112.4)	111.7 (111.2-112.2)	112.2 (111.3-113.1)
Central & Eastern Europe	116.5 (113.1-119.9)	116.0 (114.5-117.7)	114.7 (113.4-116.1)	113.2 (111.7-114.7)	111.8 (109.6-113.8)
Central Asia & MENA	114.1 (109.9-118.4)	114.2 (112.0-116.5)	113.6 (112.2-115.1)	112.6 (111.3-113.8)	111.7 (109.8-113.6)
East & Southeast Asia	107.7 (104.6-111.0)	108.2 (106.9-109.6)	108.6 (107.5-109.5)	108.7 (107.8-109.6)	109.9 (108.1-111.7)
High-income Asia Pacific	111.7 (109.2-114.0)	110.1 (108.8-111.4)	108.5 (107.5-109.6)	106.3 (105.4-107.2)	104.3 (102.6-106.0)
High-income western	115.2 (113.4-117.1)	113.3 (112.3-114.2)	111.2 (110.5-112.0)	109.7 (108.9-110.5)	108.5 (107.0-110.0)
Latin America & Caribbean	114.1 (110.4-117.9)	113.4 (111.3-115.3)	112.1 (110.8-113.3)	110.8 (109.8-111.8)	110.7 (109.0-112.4)
Oceania	111.7 (106.9-116.4)	113.1 (110.4-115.9)	115.0 (112.0-117.9)	116.7 (113.7-119.7)	118.9 (114.1-123.7)
South Asia	111.2 (105.8-116.4)	112.4 (109.8-115.0)	113.6 (111.9-115.2)	114.5 (113.3-115.8)	114.7 (112.7-116.8)
Sub-Saharan Africa	111.9 (108.7-115.1)	113.2 (111.4-115.0)	114.2 (112.9-115.6)	114.7 (113.6-115.9)	114.8 (113.0-116.5)
**20-24 years**					
World	111.5 (109.5-113.5)	111.5 (110.5-112.4)	111.5 (110.9-112.0)	111.7 (111.2-112.1)	111.9 (111.0-112.8)
Central & Eastern Europe	116.3 (112.9-119.7)	116.1 (114.6-117.6)	114.5 (113.4-115.8)	112.9 (111.6-114.3)	111.4 (109.3-113.5)
Central Asia & MENA	114.2 (110.0-118.4)	114.3 (112.1-116.4)	113.8 (112.4-115.1)	112.5 (111.5-113.6)	111.5 (109.9-113.2)
East & Southeast Asia	107.6 (104.4-111.0)	108.2 (106.9-109.5)	108.5 (107.6-109.5)	108.8 (108.0-109.6)	109.9 (108.0-111.9)
High-income Asia Pacific	112.8 (110.8-114.8)	110.4 (109.1-111.7)	108.9 (107.9-109.9)	106.6 (105.8-107.5)	104.5 (102.9-106.2)
High-income western	114.9 (113.1-116.8)	112.8 (111.9-113.7)	111.0 (110.4-111.7)	109.3 (108.7-110.0)	108.1 (106.6-109.5)
Latin America & Caribbean	114.3 (110.4-118.1)	113.5 (111.5-115.5)	112.2 (111.0-113.4)	111.0 (110.1-111.8)	110.8 (109.1-112.4)
Oceania	110.7 (106.0-115.4)	112.1 (109.7-114.5)	114.0 (111.3-116.7)	115.7 (113.1-118.4)	117.8 (113.3-122.5)
South Asia	111.1 (105.6-116.4)	112.4 (109.7-115.0)	113.5 (111.9-115.0)	114.5 (113.4-115.7)	114.7 (112.7-116.7)
Sub-Saharan Africa	111.9 (108.7-115.0)	113.2 (111.4-114.9)	114.2 (112.9-115.5)	114.6 (113.6-115.7)	114.7 (113.0-116.3)
**25-29 years**					
World	112.7 (110.7-114.7)	112.5 (111.5-113.5)	112.3 (111.7-112.9)	112.5 (112.1-113.0)	112.6 (111.7-113.6)
Central & Eastern Europe	117.5 (114.0-121.0)	117.2 (115.7-118.7)	115.6 (114.5-116.8)	113.9 (112.6-115.2)	112.2 (109.9-114.5)
Central Asia & MENA	115.5 (111.1-120.0)	115.5 (113.3-117.7)	115.0 (113.7-116.3)	113.7 (112.8-114.7)	112.4 (110.8-114.0)
East & Southeast Asia	108.7 (105.3-112.2)	109.2 (107.9-110.6)	109.5 (108.6-110.4)	110.0 (109.1-110.8)	111.0 (108.9-113.2)
High-income Asia Pacific	115.2 (113.2-117.1)	111.9 (110.5-113.3)	110.3 (109.3-111.4)	108.0 (107.2-108.8)	105.9 (104.2-107.7)
High-income western	115.6 (113.7-117.5)	113.1 (112.2-114.0)	111.7 (111.0-112.3)	109.7 (109.1-110.4)	108.3 (106.9-109.8)
Latin America & Caribbean	115.5 (111.4-119.4)	114.8 (112.6-116.7)	113.3 (112.1-114.5)	112.0 (111.2-112.9)	111.8 (110.1-113.6)
Oceania	110.1 (105.2-114.9)	111.7 (109.4-114.0)	113.5 (111.0-116.0)	115.4 (112.7-118.0)	117.5 (112.9-122.3)
South Asia	111.8 (106.0-117.2)	113.0 (110.3-115.8)	114.2 (112.6-115.8)	115.3 (114.2-116.4)	115.5 (113.5-117.5)
Sub-Saharan Africa	112.8 (109.4-116.1)	114.2 (112.4-115.9)	115.3 (114.0-116.6)	115.7 (114.7-116.7)	115.6 (113.9-117.2)
**30-34 years**					
World	114.7 (112.5-117.0)	114.4 (113.3-115.5)	114.2 (113.6-114.9)	114.1 (113.6-114.6)	114.4 (113.5-115.4)
Central & Eastern Europe	120.2 (116.4-123.9)	119.8 (118.3-121.4)	118.2 (116.9-119.5)	116.2 (114.9-117.6)	114.4 (112.0-116.8)
Central Asia & MENA	117.8 (113.1-122.7)	117.9 (115.6-120.3)	117.3 (115.9-118.6)	116.0 (115.1-117.0)	114.3 (112.6-116.0)
East & Southeast Asia	110.7 (107.1-114.5)	111.1 (109.7-112.5)	111.5 (110.6-112.5)	111.9 (111.0-112.8)	113.3 (111.1-115.5)
High-income Asia Pacific	118.0 (115.8-120.1)	115.0 (113.6-116.3)	112.7 (111.6-113.8)	110.2 (109.3-111.1)	107.9 (106.0-109.7)
High-income western	117.6 (115.6-119.8)	114.9 (113.9-115.9)	113.1 (112.4-113.8)	111.2 (110.6-111.9)	109.6 (108.0-111.1)
Latin America & Caribbean	117.8 (113.5-122.1)	117.0 (114.7-119.1)	115.4 (114.1-116.7)	114.0 (113.1-115.0)	113.9 (112.0-115.8)
Oceania	110.6 (105.4-115.8)	112.3 (109.8-114.6)	114.2 (111.6-116.8)	116.1 (113.4-119.0)	118.4 (113.5-123.5)
South Asia	113.2 (106.9-119.1)	114.5 (111.5-117.5)	115.8 (114.1-117.5)	116.9 (115.8-118.1)	117.1 (114.9-119.3)
Sub-Saharan Africa	114.8 (111.1-118.4)	116.2 (114.3-118.1)	117.4 (116.1-118.8)	117.8 (116.8-118.9)	117.7 (115.9-119.4)
**35-39 years**					
World	117.9 (115.5-120.4)	117.5 (116.3-118.6)	117.2 (116.5-117.8)	116.7 (116.2-117.3)	117.1 (116.1-118.2)

Mean systolic blood pressure (mmHg)					
Super-region (Women)	Year				
	1975	1985	1995	2005	2015
Central & Eastern Europe	124.0 (119.6-128.4)	123.7 (122.0-125.4)	121.9 (120.5-123.2)	119.8 (118.3-121.3)	117.8 (115.1-120.4)
Central Asia & MENA	121.2 (116.2-126.5)	121.3 (118.8-123.9)	120.6 (119.2-122.0)	119.2 (118.2-120.2)	117.3 (115.4-119.2)
East & Southeast Asia	113.5 (109.5-117.7)	114.1 (112.6-115.7)	114.5 (113.4-115.6)	114.8 (113.8-115.7)	116.4 (114.0-118.9)
High-income Asia Pacific	121.6 (119.2-123.9)	118.8 (117.4-120.1)	115.9 (114.6-117.1)	113.1 (112.1-114.0)	110.6 (108.6-112.7)
High-income western	120.9 (118.6-123.3)	117.7 (116.6-118.8)	115.4 (114.6-116.1)	113.7 (113.0-114.4)	111.8 (110.0-113.4)
Latin America & Caribbean	121.1 (116.2-125.8)	120.1 (117.5-122.4)	118.4 (116.9-119.8)	116.8 (115.8-117.8)	116.7 (114.6-118.8)
Oceania	111.9 (106.0-117.6)	113.7 (111.0-116.3)	115.9 (113.0-118.7)	117.9 (115.0-120.8)	120.3 (115.0-126.0)
South Asia	115.2 (108.2-121.8)	116.7 (113.3-120.0)	118.2 (116.2-120.0)	119.4 (118.1-120.6)	119.5 (117.1-122.0)
Sub-Saharan Africa	117.7 (113.5-121.6)	119.2 (117.0-121.3)	120.5 (119.0-122.0)	121.0 (119.9-122.1)	120.8 (118.8-122.7)
40-44 years					
World	121.5 (118.9-124.3)	121.2 (119.9-122.5)	120.6 (119.9-121.4)	120.2 (119.6-120.8)	120.4 (119.1-121.7)
Central & Eastern Europe	129.0 (124.3-133.6)	128.6 (126.7-130.6)	126.6 (125.1-128.1)	124.2 (122.5-126.0)	122.0 (119.1-124.9)
Central Asia & MENA	125.3 (119.6-131.3)	125.6 (122.7-128.5)	124.8 (123.2-126.4)	123.2 (122.1-124.3)	121.0 (119.0-123.1)
East & Southeast Asia	117.0 (112.5-121.7)	117.8 (116.0-119.6)	118.0 (116.8-119.2)	118.4 (117.3-119.5)	120.1 (117.3-123.1)
High-income Asia Pacific	126.3 (123.7-128.9)	122.9 (121.4-124.4)	120.0 (118.7-121.3)	116.5 (115.4-117.5)	113.9 (111.6-116.3)
High-income western	124.8 (122.3-127.5)	121.6 (120.5-122.8)	118.8 (118.0-119.6)	116.7 (115.9-117.5)	114.7 (112.8-116.6)
Latin America & Caribbean	125.0 (119.6-130.3)	124.0 (121.1-126.6)	122.0 (120.4-123.6)	120.3 (119.2-121.5)	120.2 (117.8-122.5)
Oceania	113.9 (107.4-120.2)	115.9 (112.9-118.8)	118.2 (115.0-121.4)	120.6 (117.5-123.7)	123.2 (117.2-129.4)
South Asia	117.8 (109.9-125.2)	119.5 (115.7-123.1)	121.1 (119.0-123.2)	122.5 (121.1-123.9)	122.6 (119.9-125.3)
Sub-Saharan Africa	121.2 (116.6-125.6)	123.0 (120.6-125.4)	124.4 (122.8-126.1)	124.9 (123.7-126.1)	124.6 (122.4-126.8)
45-49 years					
World	126.4 (123.4-129.3)	125.9 (124.4-127.3)	124.9 (124.1-125.7)	124.4 (123.7-125.0)	124.3 (122.8-125.9)
Central & Eastern Europe	134.9 (129.4-140.2)	134.4 (132.2-136.7)	132.0 (130.4-133.7)	129.4 (127.5-131.3)	127.1 (123.8-130.3)
Central Asia & MENA	130.2 (123.9-136.7)	130.3 (127.1-133.6)	129.5 (127.8-131.3)	127.8 (126.6-129.0)	125.4 (123.1-127.7)
East & Southeast Asia	121.0 (115.9-126.3)	121.9 (119.9-123.9)	122.3 (120.9-123.7)	122.7 (121.5-123.8)	124.5 (121.3-127.9)
High-income Asia Pacific	131.6 (128.7-134.4)	127.5 (125.8-129.2)	124.5 (123.1-125.9)	120.4 (119.2-121.6)	117.3 (114.8-120.0)
High-income western	129.5 (126.7-132.5)	126.5 (125.2-127.9)	123.0 (122.1-123.9)	120.3 (119.4-121.3)	118.3 (116.2-120.4)
Latin America & Caribbean	129.7 (123.6-135.7)	128.5 (125.3-131.4)	126.3 (124.4-128.1)	124.4 (123.1-125.7)	124.1 (121.5-126.7)
Oceania	116.2 (108.9-123.5)	118.6 (115.2-121.8)	121.3 (117.7-124.8)	123.9 (120.5-127.4)	126.8 (120.3-133.5)
South Asia	120.8 (112.0-129.1)	122.7 (118.5-126.8)	124.5 (122.1-126.9)	126.1 (124.6-127.7)	126.3 (123.1-129.3)
Sub-Saharan Africa	125.4 (120.3-130.3)	127.5 (124.7-130.0)	128.9 (127.1-130.8)	129.5 (128.1-130.9)	129.1 (126.7-131.6)
50-54 years					
World	131.2 (128.1-134.4)	130.3 (128.8-131.9)	129.5 (128.6-130.4)	128.7 (128.0-129.4)	128.6 (127.0-130.2)
Central & Eastern Europe	141.1 (135.1-146.8)	140.5 (138.1-142.9)	137.9 (136.1-139.8)	135.0 (132.9-137.1)	132.3 (128.5-136.0)
Central Asia & MENA	135.2 (128.1-142.6)	135.4 (131.8-139.0)	134.7 (132.7-136.7)	132.6 (131.3-134.0)	130.1 (127.6-132.6)
East & Southeast Asia	125.3 (119.6-131.1)	126.2 (124.0-128.4)	126.8 (125.3-128.3)	127.0 (125.6-128.3)	129.1 (125.5-132.9)
High-income Asia Pacific	137.0 (133.7-140.1)	132.5 (130.7-134.4)	129.0 (127.5-130.6)	124.9 (123.5-126.2)	121.0 (118.2-123.9)
High-income western	134.9 (131.7-138.2)	131.3 (129.9-132.8)	128.0 (127.0-129.0)	124.6 (123.6-125.6)	122.2 (119.9-124.6)
Latin America & Caribbean	134.6 (127.9-141.2)	133.2 (129.7-136.5)	130.8 (128.8-132.8)	128.7 (127.3-130.1)	128.5 (125.6-131.4)
Oceania	119.2 (111.0-127.2)	121.8 (118.0-125.5)	124.7 (120.8-128.7)	127.5 (123.6-131.4)	130.9 (123.9-138.2)
South Asia	124.0 (114.2-133.3)	126.1 (121.4-130.7)	128.1 (125.5-130.7)	130.0 (128.3-131.7)	130.2 (126.7-133.6)
Sub-Saharan Africa	129.9 (124.1-135.4)	132.1 (129.0-135.0)	133.9 (131.8-136.0)	134.4 (132.9-135.9)	134.0 (131.3-136.6)
55-59 years					
World	135.2 (131.7-138.8)	135.4 (133.7-137.0)	134.4 (133.4-135.3)	132.9 (132.2-133.7)	132.8 (131.1-134.5)
Central & Eastern Europe	147.1 (140.3-153.6)	146.6 (143.8-149.3)	143.8 (141.7-146.0)	140.4 (138.1-142.7)	137.4 (133.3-141.4)
Central Asia & MENA	140.2 (132.4-148.3)	140.3 (136.3-144.3)	139.4 (137.2-141.6)	137.3 (135.8-138.8)	134.5 (131.7-137.3)

Mean systolic blood pressure (mmHg)					
Super-region (Women)	Year				
	1975	1985	1995	2005	2015
East & Southeast Asia	129.4 (123.2-135.8)	130.4 (128.0-132.9)	131.0 (129.4-132.7)	131.4 (129.9-132.8)	133.5 (129.7-137.6)
High-income Asia Pacific	142.1 (138.6-145.5)	137.3 (135.3-139.3)	133.3 (131.6-135.0)	129.0 (127.4-130.6)	124.7 (121.7-127.9)
High-income western	139.5 (136.2-143.0)	136.1 (134.6-137.8)	133.2 (132.1-134.3)	129.1 (128.1-130.2)	126.3 (123.7-129.0)
Latin America & Caribbean	139.4 (132.1-146.6)	137.9 (134.1-141.6)	135.3 (133.1-137.4)	132.9 (131.4-134.5)	132.7 (129.6-136.0)
Oceania	122.1 (113.1-131.0)	124.7 (120.4-128.9)	128.0 (123.5-132.4)	131.2 (127.0-135.5)	134.9 (127.3-142.8)
South Asia	127.1 (116.4-137.3)	129.4 (124.3-134.4)	131.6 (128.8-134.5)	133.6 (131.8-135.5)	133.9 (130.1-137.8)
Sub-Saharan Africa	134.3 (128.0-140.3)	136.6 (133.3-139.8)	138.6 (136.4-140.9)	139.1 (137.4-140.8)	138.6 (135.7-141.5)
60-64 years					
World	140.6 (137.1-144.2)	139.4 (137.7-141.2)	138.1 (137.1-139.1)	136.7 (135.9-137.5)	136.5 (134.6-138.4)
Central & Eastern Europe	152.3 (145.2-159.2)	151.6 (148.6-154.4)	148.5 (146.4-150.8)	145.2 (142.8-147.5)	142.0 (137.7-146.3)
Central Asia & MENA	144.4 (136.2-152.8)	144.4 (140.2-148.7)	143.5 (141.3-145.9)	141.3 (139.7-143.0)	138.2 (135.1-141.2)
East & Southeast Asia	133.0 (126.4-139.8)	134.0 (131.5-136.6)	134.6 (132.9-136.4)	135.1 (133.6-136.6)	137.3 (133.0-141.8)
High-income Asia Pacific	146.5 (142.9-150.1)	141.3 (139.2-143.4)	137.1 (135.3-138.9)	132.4 (130.7-134.1)	128.6 (125.1-132.2)
High-income western	145.0 (141.3-148.9)	140.8 (139.1-142.6)	137.4 (136.3-138.6)	133.5 (132.4-134.6)	130.3 (127.5-133.1)
Latin America & Caribbean	143.6 (135.8-151.2)	141.9 (137.9-145.8)	139.1 (136.7-141.4)	136.6 (135.0-138.3)	136.4 (133.1-139.8)
Oceania	124.6 (115.1-134.0)	127.5 (122.9-132.1)	131.1 (126.3-135.7)	134.4 (129.9-139.2)	138.2 (130.0-146.7)
South Asia	129.8 (118.5-140.6)	132.3 (126.8-137.6)	134.6 (131.6-137.6)	136.7 (134.7-138.7)	137.1 (133.0-141.3)
Sub-Saharan Africa	138.0 (131.4-144.4)	140.5 (137.0-144.0)	142.5 (140.1-145.0)	143.3 (141.5-145.1)	142.7 (139.6-145.7)
65-69 years					
World	144.3 (140.8-148.0)	142.1 (140.3-143.9)	141.7 (140.7-142.7)	140.2 (139.3-141.0)	139.2 (137.3-141.1)
Central & Eastern Europe	156.3 (149.0-163.4)	155.7 (152.6-158.8)	152.6 (150.1-155.1)	149.1 (146.4-151.9)	145.4 (141.1-149.7)
Central Asia & MENA	147.6 (139.2-156.3)	147.7 (143.4-152.2)	146.7 (144.2-149.3)	144.5 (142.7-146.3)	141.1 (137.9-144.2)
East & Southeast Asia	135.8 (129.0-143.0)	136.9 (134.3-139.5)	137.5 (135.7-139.4)	138.0 (136.4-139.6)	140.4 (136.0-145.0)
High-income Asia Pacific	149.9 (146.1-153.6)	144.4 (142.3-146.6)	140.0 (138.2-141.9)	135.2 (133.4-136.8)	131.6 (127.8-135.5)
High-income western	149.1 (145.2-153.1)	143.9 (142.1-145.7)	141.1 (139.9-142.3)	137.5 (136.3-138.7)	134.0 (131.2-136.8)
Latin America & Caribbean	147.0 (139.0-154.9)	145.2 (141.1-149.2)	142.4 (139.9-144.8)	139.8 (138.1-141.6)	139.5 (136.0-143.1)
Oceania	126.7 (116.6-136.7)	129.9 (124.8-135.0)	133.3 (128.1-138.6)	136.9 (131.9-142.0)	141.0 (132.5-149.7)
South Asia	132.0 (120.2-143.2)	134.5 (128.9-140.0)	136.9 (133.7-140.0)	139.1 (137.0-141.2)	139.4 (135.2-143.7)
Sub-Saharan Africa	141.1 (134.3-147.7)	143.8 (140.1-147.3)	145.8 (143.3-148.3)	146.5 (144.5-148.4)	145.9 (142.6-149.1)
70-74 years					
World	147.4 (143.8-151.1)	146.0 (144.3-147.8)	144.2 (143.2-145.2)	142.3 (141.5-143.2)	141.4 (139.6-143.3)
Central & Eastern Europe	159.1 (151.7-166.4)	158.4 (155.2-161.7)	155.2 (152.6-157.7)	151.5 (148.8-154.1)	148.3 (143.9-152.8)
Central Asia & MENA	149.9 (141.4-158.9)	150.0 (145.4-154.6)	148.9 (146.3-151.5)	146.5 (144.7-148.4)	143.0 (139.7-146.3)
East & Southeast Asia	138.0 (131.0-145.4)	139.1 (136.5-141.8)	139.7 (137.8-141.6)	140.2 (138.5-141.8)	142.7 (138.2-147.4)
High-income Asia Pacific	152.7 (148.8-156.4)	146.9 (144.7-149.1)	142.3 (140.4-144.2)	137.4 (135.6-139.2)	133.7 (129.7-137.7)
High-income western	152.5 (148.5-156.7)	148.1 (146.3-150.0)	144.4 (143.2-145.7)	140.7 (139.5-141.9)	137.0 (134.2-139.8)
Latin America & Caribbean	149.6 (141.4-157.6)	147.8 (143.5-151.7)	144.8 (142.2-147.3)	142.2 (140.4-144.0)	141.9 (138.3-145.4)
Oceania	128.6 (118.1-139.0)	131.6 (125.9-137.3)	135.2 (129.4-141.0)	138.8 (133.3-144.4)	143.1 (134.5-151.9)
South Asia	133.5 (121.6-144.8)	136.1 (130.4-141.8)	138.6 (135.3-141.7)	140.8 (138.6-143.0)	141.1 (136.8-145.5)
Sub-Saharan Africa	143.3 (136.4-150.0)	146.0 (142.2-149.6)	148.0 (145.4-150.7)	148.6 (146.6-150.7)	148.3 (144.9-151.6)
75-79 years					
World	149.8 (146.3-153.3)	148.3 (146.6-150.0)	145.5 (144.4-146.5)	144.3 (143.4-145.1)	143.1 (141.3-145.0)
Central & Eastern Europe	160.6 (153.2-168.0)	160.0 (156.6-163.4)	156.9 (154.0-159.7)	153.1 (150.2-156.1)	149.8 (144.9-154.7)
Central Asia & MENA	151.1 (142.4-160.4)	151.4 (146.7-156.1)	150.4 (147.6-153.1)	148.0 (145.9-150.2)	144.8 (141.5-148.1)
East & Southeast Asia	139.6 (132.5-146.9)	140.6 (138.0-143.4)	141.2 (139.3-143.2)	141.7 (139.9-143.4)	144.3 (139.7-149.0)
High-income Asia Pacific	154.5 (150.6-158.3)	148.8 (146.6-150.9)	144.1 (142.2-146.0)	139.2 (137.3-141.0)	135.3 (131.4-139.4)

Mean systolic blood pressure (mmHg)					
Super-region (Women)	Year				
	1975	1985	1995	2005	2015
High-income western	154.9 (150.9-159.1)	150.7 (148.9-152.6)	146.2 (145.0-147.5)	143.1 (141.9-144.3)	139.4 (136.4-142.3)
Latin America & Caribbean	151.7 (143.3-160.0)	149.6 (145.4-153.7)	146.8 (144.2-149.4)	144.1 (142.2-146.0)	143.9 (140.2-147.5)
Oceania	130.5 (119.7-141.1)	133.0 (126.3-139.7)	136.7 (130.0-143.3)	139.9 (133.4-146.3)	144.3 (135.0-153.6)
South Asia	134.6 (122.4-146.1)	137.2 (131.4-142.9)	139.7 (136.4-142.9)	141.9 (139.6-144.1)	142.3 (137.9-146.6)
Sub-Saharan Africa	144.9 (137.9-151.5)	147.5 (143.7-151.1)	149.6 (146.9-152.3)	150.1 (148.0-152.2)	149.8 (146.3-153.3)
**80-84 years**					
World	151.8 (148.4-155.3)	149.7 (148.0-151.4)	147.7 (146.6-148.8)	145.2 (144.3-146.1)	143.8 (141.9-145.6)
Central & Eastern Europe	161.2 (153.3-168.8)	160.4 (156.6-164.0)	157.1 (154.1-160.3)	153.4 (150.2-156.6)	149.8 (145.0-154.5)
Central Asia & MENA	152.0 (143.3-161.3)	152.0 (146.9-157.1)	150.9 (147.8-154.3)	148.4 (146.0-150.8)	144.5 (141.0-148.1)
East & Southeast Asia	140.6 (133.5-147.9)	141.6 (138.8-144.3)	142.2 (140.2-144.2)	142.8 (141.0-144.6)	145.2 (140.6-150.1)
High-income Asia Pacific	156.0 (152.1-159.8)	150.3 (148.0-152.5)	145.5 (143.6-147.4)	140.5 (138.6-142.4)	136.7 (132.6-141.0)
High-income western	156.6 (152.6-160.9)	152.5 (150.6-154.5)	148.9 (147.5-150.2)	144.9 (143.6-146.2)	141.3 (138.4-144.1)
Latin America & Caribbean	153.2 (144.6-161.4)	150.7 (146.3-154.9)	147.8 (145.0-150.5)	145.3 (143.2-147.4)	145.1 (141.3-148.8)
Oceania	131.7 (120.5-143.0)	133.7 (125.6-141.8)	137.0 (128.9-145.2)	140.4 (132.7-148.3)	144.8 (134.8-154.9)
South Asia	135.3 (123.1-146.7)	137.8 (131.9-143.4)	140.3 (136.9-143.7)	142.5 (140.2-144.8)	142.8 (138.4-147.3)
Sub-Saharan Africa	145.7 (138.8-152.3)	148.0 (144.0-151.8)	150.1 (147.2-153.1)	150.7 (148.2-153.2)	150.5 (146.6-154.1)
**85+ years**					
World	153.4 (149.9-157.0)	150.5 (148.6-152.4)	148.2 (146.9-149.5)	145.3 (144.2-146.4)	143.8 (141.9-145.7)
Central & Eastern Europe	160.1 (151.2-168.9)	159.2 (154.4-163.8)	155.6 (151.5-159.9)	151.8 (147.7-155.8)	148.3 (142.8-153.9)
Central Asia & MENA	152.3 (142.2-162.3)	151.7 (145.6-157.7)	150.4 (146.0-154.9)	147.9 (144.4-151.2)	144.1 (140.0-148.3)
East & Southeast Asia	141.1 (133.6-148.7)	142.0 (138.9-145.2)	142.7 (140.2-145.1)	143.1 (140.9-145.4)	145.9 (141.2-151.0)
High-income Asia Pacific	157.7 (153.6-161.9)	151.7 (149.3-154.2)	146.9 (144.7-149.1)	141.9 (139.6-144.1)	138.3 (133.7-142.9)
High-income western	157.8 (153.6-162.2)	153.1 (151.1-155.3)	149.6 (148.0-151.2)	145.7 (144.2-147.2)	142.3 (139.2-145.4)
Latin America & Caribbean	153.8 (145.1-162.5)	151.7 (146.9-156.4)	148.5 (145.2-151.7)	146.4 (143.7-149.2)	146.1 (141.9-150.6)
Oceania	129.2 (114.5-144.0)	132.7 (121.6-143.9)	136.2 (124.6-147.5)	140.0 (129.2-151.1)	144.1 (131.3-156.7)
South Asia	135.3 (122.5-147.4)	137.9 (131.4-144.1)	140.5 (136.4-144.4)	142.7 (139.7-145.8)	143.0 (138.0-148.0)
Sub-Saharan Africa	145.8 (138.6-152.9)	148.0 (143.5-152.4)	149.8 (146.1-153.6)	150.5 (147.1-154.1)	150.0 (145.5-154.4)

Mean diastolic blood pressure (mmHg)					
Super-region (Men)	Year				
	1975	1985	1995	2005	2015
18-19 years					
World	71.9 (70.2-73.6)	71.9 (71.1-72.8)	72.2 (71.6-72.7)	72.4 (72.0-72.8)	72.5 (71.7-73.2)
Central & Eastern Europe	76.3 (73.6-78.9)	76.2 (74.9-77.5)	75.7 (74.6-76.8)	75.2 (74.0-76.4)	74.9 (73.1-76.7)
Central Asia & MENA	71.9 (68.4-75.3)	72.1 (70.1-73.9)	72.2 (71.1-73.4)	72.5 (71.5-73.4)	72.6 (71.2-74.0)
East & Southeast Asia	70.6 (67.8-73.4)	71.2 (70.0-72.3)	71.8 (70.9-72.6)	72.6 (71.9-73.3)	72.4 (70.9-74.0)
High-income Asia Pacific	70.9 (68.8-72.9)	70.6 (69.5-71.7)	70.4 (69.5-71.3)	69.9 (69.2-70.7)	69.4 (68.0-70.8)
High-income western	72.7 (71.2-74.3)	71.0 (70.2-71.8)	69.5 (68.8-70.1)	68.2 (67.6-68.8)	67.3 (66.0-68.5)
Latin America & Caribbean	72.0 (68.7-75.0)	72.6 (70.9-74.3)	72.6 (71.5-73.8)	72.1 (71.3-72.9)	71.2 (69.8-72.7)
Oceania	67.6 (63.7-71.4)	68.0 (65.7-70.3)	68.5 (65.9-71.0)	68.9 (66.4-71.4)	69.6 (65.7-73.6)
South Asia	73.2 (68.7-77.6)	73.7 (71.3-75.9)	74.0 (72.5-75.4)	74.5 (73.4-75.5)	75.6 (73.9-77.3)
Sub-Saharan Africa	68.4 (65.8-71.0)	69.4 (67.9-70.8)	70.0 (68.9-71.2)	70.3 (69.3-71.3)	70.5 (69.1-71.9)
20-24 years					
World	73.3 (71.7-75.1)	73.4 (72.6-74.3)	73.6 (73.1-74.1)	73.8 (73.4-74.2)	73.9 (73.2-74.6)
Central & Eastern Europe	77.9 (75.4-80.5)	78.2 (77.0-79.4)	77.4 (76.4-78.4)	76.9 (75.7-78.0)	76.6 (74.8-78.4)
Central Asia & MENA	73.2 (69.6-76.5)	73.4 (71.5-75.2)	73.5 (72.5-74.6)	73.7 (72.9-74.5)	74.0 (72.7-75.3)
East & Southeast Asia	71.7 (68.9-74.6)	72.3 (71.1-73.4)	73.0 (72.2-73.7)	73.6 (73.0-74.3)	73.5 (71.9-75.2)
High-income Asia Pacific	72.8 (71.0-74.6)	72.7 (71.5-73.8)	72.4 (71.5-73.2)	72.0 (71.3-72.7)	71.4 (70.0-72.8)
High-income western	75.0 (73.5-76.5)	73.2 (72.4-73.9)	71.8 (71.2-72.4)	70.5 (69.9-71.0)	69.4 (68.2-70.6)
Latin America & Caribbean	73.6 (70.3-76.6)	74.2 (72.5-75.8)	74.3 (73.2-75.4)	73.7 (72.9-74.5)	72.8 (71.4-74.2)
Oceania	68.8 (64.9-72.7)	69.3 (67.2-71.3)	69.7 (67.4-72.1)	70.1 (67.8-72.4)	70.8 (67.0-74.6)
South Asia	74.3 (69.9-78.9)	74.9 (72.5-77.1)	75.1 (73.7-76.5)	75.6 (74.6-76.6)	76.8 (75.1-78.5)
Sub-Saharan Africa	70.3 (67.7-72.9)	71.2 (69.8-72.6)	71.9 (70.8-73.0)	72.2 (71.3-73.0)	72.3 (71.0-73.7)
25-29 years					
World	75.4 (73.8-77.1)	75.5 (74.6-76.3)	75.4 (74.9-76.0)	75.7 (75.3-76.1)	75.8 (75.0-76.6)
Central & Eastern Europe	80.2 (77.6-82.8)	80.4 (79.2-81.5)	79.6 (78.7-80.6)	79.1 (78.0-80.1)	78.8 (76.9-80.6)
Central Asia & MENA	75.0 (71.4-78.4)	75.3 (73.4-77.0)	75.4 (74.4-76.3)	75.5 (74.8-76.2)	75.8 (74.6-77.1)
East & Southeast Asia	73.3 (70.5-76.2)	73.8 (72.7-75.0)	74.5 (73.8-75.3)	75.3 (74.6-75.9)	75.2 (73.4-76.9)
High-income Asia Pacific	75.5 (73.8-77.2)	75.2 (74.1-76.4)	75.1 (74.2-75.9)	74.5 (73.8-75.2)	74.0 (72.5-75.4)
High-income western	77.7 (76.2-79.3)	75.9 (75.1-76.6)	74.6 (74.1-75.2)	73.3 (72.8-73.9)	72.1 (70.9-73.3)
Latin America & Caribbean	75.7 (72.4-78.8)	76.3 (74.7-78.0)	76.4 (75.3-77.5)	75.9 (75.1-76.6)	74.9 (73.5-76.3)
Oceania	70.4 (66.5-74.3)	70.9 (68.9-72.9)	71.4 (69.2-73.5)	71.7 (69.6-73.8)	72.5 (68.7-76.2)
South Asia	75.9 (71.3-80.4)	76.4 (74.0-78.7)	76.6 (75.2-78.0)	77.1 (76.2-78.0)	78.3 (76.6-80.1)
Sub-Saharan Africa	72.7 (70.1-75.3)	73.7 (72.3-75.1)	74.3 (73.3-75.4)	74.6 (73.8-75.4)	74.8 (73.5-76.2)
30-34 years					
World	77.2 (75.5-78.9)	77.2 (76.4-78.1)	77.2 (76.7-77.7)	77.4 (77.0-77.8)	77.5 (76.8-78.2)
Central & Eastern Europe	82.2 (79.7-84.8)	82.4 (81.3-83.6)	81.8 (80.9-82.8)	81.1 (80.1-82.2)	80.8 (78.9-82.7)
Central Asia & MENA	76.6 (72.9-80.1)	77.0 (75.1-78.7)	77.1 (76.2-78.1)	77.2 (76.5-77.9)	77.4 (76.2-78.7)
East & Southeast Asia	74.8 (72.0-77.7)	75.3 (74.2-76.5)	76.1 (75.3-76.8)	76.9 (76.2-77.6)	76.7 (75.1-78.4)
High-income Asia Pacific	78.0 (76.2-79.7)	77.4 (76.4-78.5)	77.4 (76.5-78.3)	76.7 (76.0-77.5)	76.2 (74.8-77.7)
High-income western	80.2 (78.7-81.8)	78.3 (77.5-79.1)	76.9 (76.3-77.5)	75.7 (75.2-76.3)	74.5 (73.3-75.7)
Latin America & Caribbean	77.8 (74.3-80.9)	78.3 (76.6-80.0)	78.4 (77.3-79.5)	77.8 (77.1-78.6)	76.8 (75.3-78.3)
Oceania	71.8 (67.9-75.8)	72.4 (70.4-74.4)	72.8 (70.8-75.0)	73.1 (71.0-75.2)	73.9 (70.2-77.6)
South Asia	77.2 (72.6-81.9)	77.7 (75.3-80.1)	78.0 (76.6-79.3)	78.5 (77.5-79.4)	79.6 (77.9-81.4)
Sub-Saharan Africa	74.9 (72.3-77.7)	75.9 (74.5-77.3)	76.6 (75.5-77.7)	76.8 (76.0-77.7)	77.0 (75.7-78.4)
35-39 years					
World	79.0 (77.3-80.8)	78.9 (78.1-79.8)	78.9 (78.4-79.4)	78.9 (78.5-79.3)	79.1 (78.3-79.8)

Mean diastolic blood pressure (mmHg)					
**Super-region (Men)**	**Year**				
	**1975**	**1985**	**1995**	**2005**	**2015**
Central & Eastern Europe	84.2 (81.4-87.1)	84.3 (83.1-85.5)	83.6 (82.6-84.6)	83.0 (81.9-84.0)	82.6 (80.8-84.5)
Central Asia & MENA	78.4 (74.6-81.9)	78.6 (76.7-80.3)	78.7 (77.7-79.7)	78.8 (78.1-79.5)	78.9 (77.7-80.2)
East & Southeast Asia	76.2 (73.3-79.2)	76.8 (75.7-78.0)	77.6 (76.8-78.3)	78.4 (77.7-79.1)	78.2 (76.6-80.0)
High-income Asia Pacific	80.0 (78.2-81.8)	79.5 (78.5-80.5)	79.3 (78.4-80.2)	78.7 (78.0-79.5)	78.2 (76.6-79.7)
High-income western	82.4 (80.7-84.0)	80.3 (79.6-81.1)	78.7 (78.1-79.3)	77.7 (77.2-78.3)	76.6 (75.4-77.8)
Latin America & Caribbean	79.6 (76.0-82.8)	80.1 (78.3-81.8)	80.1 (79.0-81.2)	79.5 (78.8-80.3)	78.6 (77.0-80.1)
Oceania	73.1 (69.1-77.1)	73.6 (71.6-75.6)	74.1 (72.0-76.3)	74.4 (72.5-76.5)	75.1 (71.3-78.9)
South Asia	78.3 (73.6-83.1)	78.8 (76.4-81.2)	79.1 (77.7-80.5)	79.6 (78.6-80.5)	80.8 (79.0-82.6)
Sub-Saharan Africa	76.8 (74.1-79.6)	77.9 (76.4-79.3)	78.6 (77.5-79.6)	78.8 (78.0-79.6)	78.9 (77.6-80.3)
**40-44 years**					
World	80.4 (78.6-82.2)	80.3 (79.4-81.2)	80.3 (79.7-80.8)	80.3 (79.9-80.7)	80.3 (79.5-81.2)
Central & Eastern Europe	85.7 (83.0-88.5)	85.9 (84.8-87.1)	85.2 (84.3-86.2)	84.6 (83.5-85.8)	84.2 (82.3-86.2)
Central Asia & MENA	79.6 (75.7-83.2)	79.9 (78.0-81.7)	80.1 (79.1-81.1)	80.3 (79.6-81.0)	80.4 (79.1-81.6)
East & Southeast Asia	77.6 (74.6-80.7)	78.2 (77.0-79.4)	78.9 (78.1-79.7)	79.8 (79.0-80.5)	79.6 (77.8-81.5)
High-income Asia Pacific	81.9 (80.1-83.6)	81.2 (80.2-82.2)	80.9 (80.0-81.7)	80.3 (79.6-81.1)	79.8 (78.2-81.3)
High-income western	83.9 (82.3-85.6)	82.1 (81.3-82.8)	80.3 (79.7-80.9)	79.2 (78.6-79.7)	78.1 (76.9-79.3)
Latin America & Caribbean	81.1 (77.5-84.4)	81.6 (79.8-83.4)	81.7 (80.5-82.8)	81.1 (80.3-81.9)	80.0 (78.5-81.5)
Oceania	74.1 (70.1-78.3)	74.7 (72.7-76.8)	75.2 (73.0-77.4)	75.6 (73.6-77.6)	76.2 (72.2-80.1)
South Asia	79.2 (74.4-84.2)	79.8 (77.3-82.2)	80.1 (78.6-81.5)	80.6 (79.6-81.5)	81.8 (79.9-83.6)
Sub-Saharan Africa	78.5 (75.7-81.3)	79.6 (78.1-81.1)	80.2 (79.2-81.4)	80.5 (79.7-81.4)	80.6 (79.2-82.1)
**45-49 years**					
World	81.8 (80.1-83.6)	81.7 (80.9-82.6)	81.5 (81.0-82.0)	81.5 (81.1-81.9)	81.4 (80.5-82.3)
Central & Eastern Europe	87.2 (84.4-90.1)	87.4 (86.1-88.7)	86.6 (85.6-87.6)	85.9 (84.8-87.1)	85.6 (83.7-87.6)
Central Asia & MENA	81.0 (77.1-84.7)	81.4 (79.4-83.2)	81.4 (80.3-82.4)	81.6 (80.9-82.2)	81.6 (80.3-82.9)
East & Southeast Asia	78.8 (75.7-81.9)	79.4 (78.2-80.6)	80.2 (79.3-81.0)	81.0 (80.3-81.8)	80.9 (78.9-82.8)
High-income Asia Pacific	83.4 (81.6-85.1)	82.6 (81.5-83.6)	82.2 (81.3-83.1)	81.6 (80.8-82.3)	80.9 (79.4-82.5)
High-income western	85.2 (83.5-86.9)	83.5 (82.7-84.3)	81.6 (81.0-82.2)	80.1 (79.6-80.7)	79.2 (78.0-80.4)
Latin America & Caribbean	82.4 (78.7-85.8)	82.9 (81.1-84.7)	82.9 (81.8-84.1)	82.3 (81.5-83.2)	81.2 (79.7-82.8)
Oceania	75.0 (70.8-79.2)	75.5 (73.4-77.7)	76.0 (73.8-78.3)	76.5 (74.5-78.5)	77.2 (73.3-81.1)
South Asia	80.0 (75.1-85.1)	80.6 (78.0-83.0)	80.8 (79.3-82.3)	81.4 (80.4-82.3)	82.6 (80.7-84.5)
Sub-Saharan Africa	79.8 (77.0-82.7)	80.9 (79.4-82.5)	81.6 (80.5-82.8)	81.9 (81.1-82.8)	82.0 (80.6-83.5)
**50-54 years**					
World	82.6 (80.8-84.4)	82.5 (81.6-83.4)	82.3 (81.8-82.9)	82.4 (82.0-82.8)	82.2 (81.3-83.1)
Central & Eastern Europe	88.1 (85.3-90.9)	88.4 (87.2-89.7)	87.7 (86.7-88.8)	87.0 (85.9-88.1)	86.6 (84.6-88.7)
Central Asia & MENA	81.8 (77.8-85.7)	82.2 (80.2-84.1)	82.4 (81.3-83.4)	82.6 (81.8-83.3)	82.7 (81.4-84.1)
East & Southeast Asia	79.7 (76.6-83.0)	80.4 (79.2-81.6)	81.2 (80.3-82.0)	82.1 (81.3-82.9)	81.9 (79.9-83.9)
High-income Asia Pacific	84.2 (82.3-86.0)	83.6 (82.5-84.6)	83.1 (82.2-84.0)	82.5 (81.7-83.3)	81.6 (80.0-83.2)
High-income western	85.9 (84.2-87.7)	84.2 (83.3-85.0)	82.5 (81.9-83.1)	80.8 (80.2-81.4)	79.7 (78.5-81.0)
Latin America & Caribbean	83.3 (79.5-86.7)	83.8 (81.9-85.7)	83.8 (82.6-85.0)	83.2 (82.4-84.1)	82.2 (80.5-83.8)
Oceania	75.6 (71.3-79.9)	76.1 (73.9-78.4)	76.6 (74.3-78.9)	77.0 (75.0-79.1)	77.9 (74.1-81.8)
South Asia	80.5 (75.5-85.7)	81.1 (78.5-83.6)	81.4 (79.9-82.9)	81.9 (80.9-82.9)	83.2 (81.2-85.1)
Sub-Saharan Africa	80.8 (77.8-83.8)	81.9 (80.3-83.5)	82.7 (81.5-83.9)	82.9 (82.1-83.8)	83.1 (81.6-84.6)
**55-59 years**					
World	82.7 (80.9-84.7)	83.1 (82.2-84.0)	83.0 (82.5-83.5)	82.8 (82.4-83.2)	82.6 (81.7-83.5)
Central & Eastern Europe	88.8 (85.9-91.7)	89.1 (87.8-90.4)	88.3 (87.2-89.5)	87.5 (86.4-88.7)	87.2 (85.1-89.2)
Central Asia & MENA	82.4 (78.3-86.4)	83.0 (80.9-84.9)	83.2 (82.1-84.3)	83.2 (82.4-83.9)	83.3 (81.9-84.7)

Mean diastolic blood pressure (mmHg)					
Super-region (Men)	Year				
	1975	1985	1995	2005	2015
East & Southeast Asia	80.3 (77.0-83.7)	81.0 (79.7-82.2)	81.8 (80.9-82.7)	82.7 (81.9-83.5)	82.5 (80.5-84.5)
High-income Asia Pacific	84.6 (82.6-86.5)	84.1 (83.0-85.1)	83.5 (82.6-84.4)	82.9 (82.1-83.7)	81.9 (80.3-83.6)
High-income western	85.9 (84.2-87.7)	84.2 (83.4-85.1)	82.9 (82.3-83.5)	81.0 (80.4-81.6)	79.6 (78.3-81.0)
Latin America & Caribbean	83.7 (79.8-87.2)	84.3 (82.3-86.2)	84.2 (83.0-85.5)	83.6 (82.8-84.5)	82.5 (80.8-84.2)
Oceania	75.8 (71.4-80.2)	76.2 (73.9-78.6)	76.7 (74.3-79.2)	77.1 (75.0-79.4)	78.1 (74.2-82.1)
South Asia	80.7 (75.5-86.0)	81.3 (78.6-83.9)	81.6 (80.0-83.1)	82.1 (81.1-83.1)	83.5 (81.4-85.5)
Sub-Saharan Africa	81.3 (78.2-84.4)	82.4 (80.7-84.0)	83.2 (82.0-84.4)	83.5 (82.6-84.4)	83.6 (82.1-85.2)
**60-64 years**					
World	83.1 (81.3-85.0)	82.8 (81.9-83.7)	82.7 (82.2-83.3)	82.6 (82.2-83.0)	82.5 (81.5-83.5)
Central & Eastern Europe	88.7 (85.7-91.7)	88.9 (87.6-90.3)	88.2 (87.1-89.4)	87.5 (86.4-88.6)	87.1 (85.0-89.2)
Central Asia & MENA	82.6 (78.3-86.7)	83.0 (80.8-85.0)	83.2 (82.1-84.3)	83.3 (82.5-84.0)	83.3 (81.9-84.8)
East & Southeast Asia	80.4 (77.0-83.9)	81.0 (79.8-82.3)	81.9 (80.9-82.8)	82.8 (82.0-83.7)	82.6 (80.4-84.8)
High-income Asia Pacific	84.4 (82.5-86.3)	83.7 (82.5-84.7)	83.2 (82.2-84.1)	82.5 (81.7-83.4)	82.0 (80.2-83.8)
High-income western	85.7 (83.8-87.6)	83.6 (82.8-84.5)	82.2 (81.6-82.8)	80.5 (79.9-81.1)	79.0 (77.6-80.3)
Latin America & Caribbean	83.5 (79.5-87.0)	84.0 (82.0-86.0)	84.0 (82.7-85.3)	83.3 (82.5-84.2)	82.2 (80.5-84.0)
Oceania	75.3 (70.8-79.9)	75.8 (73.3-78.3)	76.3 (73.7-78.8)	76.6 (74.3-79.1)	77.7 (73.6-81.8)
South Asia	80.4 (75.1-85.8)	81.0 (78.3-83.7)	81.3 (79.7-82.9)	81.9 (80.8-83.0)	83.3 (81.1-85.4)
Sub-Saharan Africa	81.0 (77.9-84.2)	82.2 (80.5-83.9)	83.0 (81.8-84.3)	83.4 (82.4-84.3)	83.5 (81.9-85.1)
**65-69 years**					
World	82.5 (80.7-84.4)	81.8 (80.9-82.7)	82.1 (81.6-82.7)	82.0 (81.6-82.5)	81.6 (80.7-82.6)
Central & Eastern Europe	88.0 (85.0-91.0)	88.4 (87.0-89.9)	87.7 (86.5-89.0)	87.0 (85.7-88.3)	86.4 (84.4-88.5)
Central Asia & MENA	82.3 (77.9-86.4)	82.6 (80.3-84.7)	82.9 (81.6-84.1)	83.0 (82.2-83.8)	82.9 (81.4-84.5)
East & Southeast Asia	79.9 (76.3-83.5)	80.5 (79.2-81.9)	81.4 (80.4-82.3)	82.4 (81.5-83.3)	82.2 (79.9-84.5)
High-income Asia Pacific	83.6 (81.7-85.6)	82.7 (81.6-83.8)	82.3 (81.3-83.2)	81.5 (80.6-82.4)	81.3 (79.3-83.3)
High-income western	84.6 (82.7-86.7)	82.0 (81.1-82.9)	80.8 (80.2-81.5)	79.5 (78.8-80.1)	77.8 (76.4-79.1)
Latin America & Caribbean	82.7 (78.6-86.4)	83.2 (81.2-85.2)	83.2 (81.9-84.5)	82.5 (81.6-83.4)	81.4 (79.6-83.1)
Oceania	74.3 (69.6-79.1)	74.9 (72.1-77.6)	75.3 (72.4-78.1)	75.7 (73.1-78.4)	76.7 (72.5-81.1)
South Asia	79.7 (74.3-85.3)	80.4 (77.5-83.2)	80.7 (79.0-82.4)	81.3 (80.2-82.4)	82.7 (80.5-84.9)
Sub-Saharan Africa	80.2 (77.0-83.5)	81.5 (79.7-83.2)	82.3 (81.0-83.6)	82.7 (81.6-83.7)	82.8 (81.1-84.5)
**70-74 years**					
World	81.5 (79.7-83.5)	81.1 (80.2-82.1)	80.8 (80.2-81.4)	80.9 (80.4-81.3)	80.4 (79.5-81.4)
Central & Eastern Europe	87.0 (83.8-90.1)	87.4 (85.9-89.0)	86.6 (85.3-87.9)	85.9 (84.6-87.2)	85.5 (83.4-87.8)
Central Asia & MENA	81.5 (76.9-85.9)	82.0 (79.6-84.2)	82.1 (80.9-83.4)	82.3 (81.4-83.2)	82.2 (80.5-83.8)
East & Southeast Asia	78.9 (75.2-82.8)	79.7 (78.2-81.1)	80.6 (79.6-81.5)	81.6 (80.7-82.6)	81.4 (79.0-83.8)
High-income Asia Pacific	82.5 (80.5-84.5)	81.5 (80.3-82.6)	80.8 (79.8-81.9)	80.1 (79.2-81.1)	79.9 (77.8-82.1)
High-income western	83.2 (81.2-85.4)	80.9 (80.0-81.9)	79.1 (78.4-79.8)	77.8 (77.1-78.4)	76.0 (74.6-77.5)
Latin America & Caribbean	81.4 (77.2-85.3)	82.0 (79.9-84.1)	82.0 (80.6-83.4)	81.3 (80.3-82.2)	80.1 (78.2-81.9)
Oceania	73.1 (68.2-78.1)	73.6 (70.4-76.8)	74.0 (70.8-77.2)	74.4 (71.4-77.4)	75.5 (70.9-80.1)
South Asia	78.9 (73.2-84.7)	79.6 (76.6-82.5)	79.9 (78.2-81.7)	80.5 (79.3-81.7)	82.0 (79.8-84.2)
Sub-Saharan Africa	78.9 (75.5-82.4)	80.2 (78.4-82.1)	81.2 (79.7-82.6)	81.5 (80.4-82.6)	81.7 (80.0-83.5)
**75-79 years**					
World	80.4 (78.5-82.4)	80.0 (79.0-81.0)	79.3 (78.7-79.9)	79.5 (79.1-80.0)	79.2 (78.2-80.2)
Central & Eastern Europe	85.9 (82.5-89.3)	86.4 (84.6-88.2)	85.8 (84.2-87.2)	85.0 (83.4-86.6)	84.6 (82.1-87.2)
Central Asia & MENA	80.8 (75.8-85.4)	81.2 (78.6-83.6)	81.4 (80.0-82.9)	81.7 (80.6-82.7)	81.6 (79.8-83.3)
East & Southeast Asia	77.8 (73.8-81.8)	78.6 (77.1-80.1)	79.6 (78.5-80.6)	80.6 (79.6-81.6)	80.4 (77.8-83.0)
High-income Asia Pacific	81.1 (78.9-83.2)	80.1 (78.8-81.3)	79.3 (78.3-80.4)	78.6 (77.5-79.7)	78.3 (76.1-80.7)

Mean diastolic blood pressure (mmHg)					
**Super-region (Men)**	**Year**				
	**1975**	**1985**	**1995**	**2005**	**2015**
High-income western	81.6 (79.5-83.8)	79.3 (78.3-80.3)	76.7 (76.0-77.5)	75.8 (75.1-76.5)	74.2 (72.7-75.8)
Latin America & Caribbean	80.1 (75.5-84.3)	80.7 (78.5-83.0)	80.6 (79.1-82.1)	79.9 (78.9-81.0)	78.6 (76.7-80.6)
Oceania	71.9 (66.6-77.2)	72.2 (68.6-75.8)	72.7 (69.0-76.2)	73.0 (69.4-76.5)	74.1 (69.0-79.2)
South Asia	78.2 (72.1-84.4)	78.9 (75.8-82.0)	79.3 (77.4-81.1)	79.9 (78.6-81.2)	81.5 (79.1-83.8)
Sub-Saharan Africa	77.6 (74.0-81.2)	78.9 (76.8-80.9)	79.9 (78.2-81.5)	80.4 (79.1-81.7)	80.6 (78.7-82.6)
**80-84 years**					
World	79.5 (77.6-81.5)	78.9 (77.8-79.9)	78.4 (77.8-79.1)	78.0 (77.5-78.5)	77.7 (76.6-78.8)
Central & Eastern Europe	85.5 (81.7-89.5)	85.5 (83.5-87.6)	84.9 (83.1-86.7)	83.8 (82.1-85.5)	83.4 (80.9-86.0)
Central Asia & MENA	80.3 (75.0-85.3)	80.6 (77.7-83.2)	80.8 (79.2-82.5)	81.1 (79.9-82.3)	80.9 (79.0-82.9)
East & Southeast Asia	76.6 (72.3-80.9)	77.4 (75.8-79.1)	78.5 (77.3-79.6)	79.6 (78.5-80.6)	79.4 (76.6-82.1)
High-income Asia Pacific	79.8 (77.5-82.1)	78.8 (77.4-80.0)	77.9 (76.8-79.1)	77.1 (76.0-78.3)	77.1 (74.6-79.6)
High-income western	80.1 (77.8-82.4)	77.8 (76.7-78.9)	75.8 (75.0-76.6)	74.0 (73.2-74.8)	72.5 (70.9-74.1)
Latin America & Caribbean	78.8 (73.8-83.4)	79.5 (77.1-82.0)	79.5 (77.8-81.1)	78.6 (77.4-79.9)	77.3 (75.1-79.4)
Oceania	70.7 (64.9-76.6)	70.9 (66.7-75.0)	71.3 (67.1-75.5)	71.8 (67.6-75.8)	72.8 (67.2-78.5)
South Asia	77.9 (71.5-84.4)	78.6 (75.2-81.9)	79.0 (77.0-81.0)	79.7 (78.3-81.1)	81.3 (78.8-83.9)
Sub-Saharan Africa	76.3 (72.5-80.2)	77.6 (75.3-79.9)	78.7 (76.7-80.6)	79.2 (77.6-80.8)	79.6 (77.3-81.8)
**85+ years**					
World	79.0 (76.8-81.3)	77.8 (76.6-79.0)	77.2 (76.4-78.0)	76.6 (75.9-77.2)	76.0 (74.8-77.2)
Central & Eastern Europe	85.3 (80.9-89.9)	85.4 (82.8-88.1)	84.4 (82.1-86.8)	83.3 (81.1-85.5)	82.8 (79.6-86.0)
Central Asia & MENA	80.2 (73.9-86.0)	80.3 (76.6-83.7)	80.4 (78.1-82.6)	81.0 (79.3-82.7)	81.1 (78.8-83.5)
East & Southeast Asia	75.2 (70.6-79.9)	76.2 (74.3-78.1)	77.4 (75.9-78.7)	78.6 (77.3-79.9)	78.2 (75.2-81.1)
High-income Asia Pacific	78.9 (76.2-81.5)	77.6 (76.0-79.1)	76.7 (75.2-78.1)	75.8 (74.3-77.2)	75.9 (73.0-78.9)
High-income western	79.0 (76.4-81.6)	76.0 (74.7-77.3)	74.0 (73.0-75.0)	71.8 (70.9-72.7)	70.4 (68.5-72.2)
Latin America & Caribbean	77.7 (72.2-82.8)	78.6 (75.8-81.4)	78.6 (76.6-80.5)	77.6 (76.0-79.2)	76.0 (73.4-78.5)
Oceania	68.5 (61.0-76.0)	69.5 (64.3-74.7)	70.0 (64.5-75.3)	70.6 (65.3-75.6)	71.7 (65.0-78.5)
South Asia	78.4 (71.1-85.8)	79.2 (75.4-83.0)	79.7 (77.2-82.1)	80.4 (78.6-82.2)	82.2 (79.1-85.2)
Sub-Saharan Africa	75.5 (71.2-79.8)	76.6 (73.9-79.3)	77.8 (75.5-80.2)	78.4 (75.9-80.7)	79.3 (76.4-82.1)

Mean diastolic blood pressure (mmHg)					
**Super-region (Women)**	**Year**				
	**1975**	**1985**	**1995**	**2005**	**2015**
**18-19 years**					
World	70.7 (69.1-72.2)	70.5 (69.7-71.3)	70.8 (70.3-71.3)	71.0 (70.6-71.4)	71.4 (70.7-72.0)
Central & Eastern Europe	74.8 (72.2-77.6)	74.1 (72.9-75.4)	73.0 (72.0-74.0)	72.0 (70.9-73.1)	71.5 (69.9-73.1)
Central Asia & MENA	70.9 (67.3-74.4)	71.2 (69.4-72.9)	71.4 (70.3-72.4)	71.2 (70.4-72.1)	70.9 (69.6-72.3)
East & Southeast Asia	69.4 (66.9-72.1)	69.4 (68.3-70.5)	69.7 (68.9-70.5)	70.1 (69.4-70.8)	70.4 (69.1-71.9)
High-income Asia Pacific	68.1 (66.3-70.0)	67.2 (66.2-68.2)	66.3 (65.5-67.2)	65.4 (64.7-66.1)	64.7 (63.5-66.0)
High-income western	71.6 (70.2-73.1)	69.9 (69.1-70.6)	68.1 (67.5-68.6)	66.8 (66.2-67.4)	66.2 (65.1-67.4)
Latin America & Caribbean	71.1 (67.9-74.2)	71.3 (69.7-72.9)	70.9 (69.9-71.9)	70.0 (69.3-70.7)	69.0 (67.8-70.3)
Oceania	67.7 (64.0-71.4)	68.5 (66.4-70.6)	69.7 (67.4-72.0)	70.9 (68.7-73.3)	72.4 (68.8-76.0)
South Asia	71.4 (67.1-75.5)	72.3 (70.2-74.3)	72.9 (71.7-74.2)	73.4 (72.5-74.3)	74.3 (72.8-75.8)
Sub-Saharan Africa	68.6 (65.9-71.2)	69.9 (68.5-71.3)	71.0 (70.0-72.1)	71.8 (71.0-72.7)	72.4 (71.1-73.8)
**20-24 years**					
World	71.2 (69.6-72.8)	71.1 (70.3-71.9)	71.2 (70.7-71.6)	71.4 (71.1-71.8)	71.7 (71.0-72.4)
Central & Eastern Europe	75.5 (72.8-78.2)	75.0 (73.8-76.2)	73.6 (72.7-74.5)	72.6 (71.6-73.6)	72.0 (70.4-73.7)
Central Asia & MENA	71.4 (67.7-74.9)	71.7 (69.9-73.5)	71.9 (70.9-72.9)	71.7 (71.0-72.5)	71.4 (70.2-72.7)
East & Southeast Asia	69.8 (67.2-72.5)	69.7 (68.7-70.8)	70.0 (69.3-70.8)	70.5 (69.9-71.1)	70.7 (69.2-72.2)
High-income Asia Pacific	68.8 (67.2-70.5)	68.0 (66.9-69.0)	67.1 (66.3-67.9)	66.2 (65.5-66.8)	65.4 (64.2-66.7)
High-income western	72.6 (71.1-74.1)	70.7 (69.9-71.4)	69.0 (68.4-69.5)	67.6 (67.1-68.1)	67.0 (65.8-68.1)
Latin America & Caribbean	71.8 (68.5-74.9)	71.9 (70.3-73.5)	71.7 (70.7-72.6)	70.7 (70.0-71.3)	69.6 (68.4-70.9)
Oceania	67.6 (63.9-71.3)	68.4 (66.5-70.3)	69.7 (67.5-71.8)	70.8 (68.8-73.0)	72.3 (68.8-75.9)
South Asia	71.8 (67.4-76.0)	72.7 (70.6-74.7)	73.3 (72.1-74.5)	73.8 (73.0-74.7)	74.8 (73.3-76.2)
Sub-Saharan Africa	69.3 (66.5-71.9)	70.6 (69.2-71.9)	71.7 (70.7-72.8)	72.5 (71.7-73.3)	73.1 (71.8-74.5)
**25-29 years**					
World	72.6 (70.9-74.2)	72.4 (71.6-73.3)	72.3 (71.9-72.8)	72.6 (72.3-73.0)	72.8 (72.1-73.5)
Central & Eastern Europe	77.1 (74.4-79.9)	76.5 (75.3-77.7)	75.1 (74.2-76.0)	74.0 (73.1-75.0)	73.4 (71.7-75.1)
Central Asia & MENA	72.8 (68.9-76.5)	73.1 (71.2-74.8)	73.3 (72.3-74.2)	73.1 (72.3-73.8)	72.7 (71.4-74.0)
East & Southeast Asia	70.9 (68.2-73.8)	70.8 (69.7-72.0)	71.1 (70.4-71.8)	71.7 (71.1-72.3)	71.7 (70.2-73.4)
High-income Asia Pacific	70.6 (69.0-72.2)	69.6 (68.5-70.7)	68.7 (67.9-69.6)	67.7 (67.0-68.3)	66.9 (65.6-68.3)
High-income western	74.3 (72.7-75.8)	72.2 (71.5-72.9)	70.6 (70.1-71.1)	69.1 (68.6-69.6)	68.4 (67.3-69.6)
Latin America & Caribbean	73.3 (69.9-76.5)	73.4 (71.7-75.1)	73.1 (72.1-74.0)	72.1 (71.4-72.7)	71.0 (69.7-72.3)
Oceania	68.0 (64.2-71.8)	68.9 (67.1-70.6)	70.1 (68.0-72.1)	71.3 (69.3-73.3)	72.8 (69.3-76.4)
South Asia	72.7 (68.2-77.1)	73.7 (71.5-75.8)	74.3 (73.1-75.6)	74.8 (74.0-75.7)	75.8 (74.3-77.4)
Sub-Saharan Africa	70.6 (67.7-73.3)	72.0 (70.6-73.4)	73.2 (72.2-74.2)	74.0 (73.2-74.8)	74.6 (73.2-75.9)
**30-34 years**					
World	74.3 (72.6-76.0)	74.1 (73.3-74.9)	74.0 (73.5-74.5)	74.1 (73.7-74.4)	74.4 (73.7-75.2)
Central & Eastern Europe	79.3 (76.4-82.2)	78.7 (77.5-79.9)	77.3 (76.3-78.2)	76.1 (75.1-77.1)	75.4 (73.6-77.2)
Central Asia & MENA	74.5 (70.4-78.4)	74.9 (72.9-76.7)	75.1 (74.1-76.1)	74.9 (74.1-75.6)	74.3 (73.0-75.6)
East & Southeast Asia	72.5 (69.7-75.5)	72.3 (71.2-73.5)	72.7 (71.9-73.4)	73.2 (72.5-73.9)	73.5 (71.9-75.2)
High-income Asia Pacific	72.9 (71.2-74.6)	71.6 (70.6-72.6)	70.8 (69.9-71.7)	69.7 (69.0-70.3)	68.8 (67.5-70.3)
High-income western	76.4 (74.8-78.1)	74.2 (73.5-75.0)	72.4 (71.8-72.9)	70.9 (70.4-71.5)	70.2 (69.0-71.4)
Latin America & Caribbean	75.2 (71.6-78.6)	75.3 (73.5-77.1)	74.9 (73.9-75.9)	73.9 (73.2-74.6)	72.7 (71.3-74.2)
Oceania	68.9 (64.9-73.0)	69.8 (67.9-71.6)	71.0 (69.0-73.0)	72.2 (70.2-74.3)	73.8 (70.1-77.6)
South Asia	74.0 (69.2-78.6)	75.0 (72.6-77.3)	75.7 (74.4-77.0)	76.2 (75.3-77.1)	77.2 (75.6-78.9)
Sub-Saharan Africa	72.4 (69.3-75.3)	73.8 (72.3-75.3)	75.1 (74.1-76.2)	75.9 (75.1-76.7)	76.5 (75.1-77.9)
**35-39 years**					
World	76.5 (74.7-78.3)	76.1 (75.2-76.9)	75.9 (75.4-76.4)	75.8 (75.4-76.2)	76.2 (75.4-77.0)

Mean diastolic blood pressure (mmHg)					
**Super-region (Women)**	**Year**				
	**1975**	**1985**	**1995**	**2005**	**2015**
Central & Eastern Europe	82.0 (78.9-85.2)	81.1 (79.9-82.4)	79.6 (78.7-80.6)	78.5 (77.4-79.5)	77.7 (75.8-79.6)
Central Asia & MENA	76.7 (72.4-80.8)	77.0 (74.9-78.9)	77.2 (76.1-78.2)	76.9 (76.2-77.7)	76.3 (74.9-77.7)
East & Southeast Asia	74.4 (71.3-77.6)	74.2 (73.0-75.4)	74.6 (73.7-75.4)	75.1 (74.4-75.8)	75.4 (73.7-77.2)
High-income Asia Pacific	75.3 (73.6-77.2)	74.0 (73.0-74.9)	73.0 (72.1-74.0)	71.9 (71.2-72.6)	71.0 (69.5-72.5)
High-income western	78.8 (77.0-80.6)	76.3 (75.5-77.1)	74.2 (73.6-74.8)	72.9 (72.3-73.4)	72.0 (70.8-73.3)
Latin America & Caribbean	77.3 (73.5-80.9)	77.4 (75.5-79.2)	77.0 (75.9-78.0)	75.8 (75.1-76.6)	74.6 (73.1-76.1)
Oceania	70.0 (65.7-74.3)	70.9 (68.9-72.8)	72.2 (70.0-74.2)	73.5 (71.5-75.5)	75.1 (71.2-78.9)
South Asia	75.4 (70.3-80.3)	76.4 (74.0-78.9)	77.2 (75.8-78.5)	77.7 (76.8-78.6)	78.8 (77.0-80.5)
Sub-Saharan Africa	74.2 (71.0-77.3)	75.8 (74.2-77.3)	77.2 (76.1-78.3)	78.0 (77.2-78.8)	78.6 (77.1-80.0)
**40-44 years**					
World	78.4 (76.6-80.3)	78.0 (77.1-78.9)	77.8 (77.3-78.3)	77.7 (77.3-78.1)	77.8 (76.9-78.7)
Central & Eastern Europe	84.5 (81.3-87.8)	83.7 (82.4-85.1)	82.2 (81.1-83.2)	80.9 (79.8-82.1)	80.1 (78.1-82.1)
Central Asia & MENA	78.6 (74.0-83.0)	79.0 (76.8-81.1)	79.3 (78.2-80.4)	79.1 (78.3-79.8)	78.4 (76.9-79.8)
East & Southeast Asia	76.2 (73.0-79.6)	76.2 (74.9-77.5)	76.4 (75.5-77.2)	77.0 (76.3-77.7)	77.2 (75.3-79.2)
High-income Asia Pacific	77.9 (76.0-79.7)	76.4 (75.4-77.4)	75.3 (74.4-76.2)	74.1 (73.3-74.8)	73.1 (71.6-74.8)
High-income western	80.9 (79.0-82.8)	78.5 (77.7-79.3)	76.1 (75.5-76.7)	74.6 (74.0-75.2)	73.8 (72.5-75.2)
Latin America & Caribbean	79.3 (75.3-83.2)	79.4 (77.4-81.4)	79.0 (77.8-80.1)	77.8 (77.0-78.6)	76.5 (74.9-78.1)
Oceania	71.1 (66.6-75.6)	72.0 (69.9-74.1)	73.3 (71.1-75.5)	74.8 (72.7-76.9)	76.4 (72.3-80.5)
South Asia	76.8 (71.4-82.1)	77.9 (75.3-80.4)	78.7 (77.2-80.1)	79.2 (78.3-80.2)	80.3 (78.5-82.2)
Sub-Saharan Africa	76.1 (72.7-79.3)	77.8 (76.0-79.4)	79.2 (78.0-80.4)	80.1 (79.2-81.0)	80.6 (79.1-82.2)
**45-49 years**					
World	80.6 (78.7-82.5)	80.1 (79.2-81.0)	79.5 (79.0-80.0)	79.4 (79.0-79.8)	79.4 (78.4-80.4)
Central & Eastern Europe	87.1 (83.7-90.7)	86.4 (84.9-87.9)	84.5 (83.4-85.5)	83.2 (82.0-84.4)	82.4 (80.3-84.5)
Central Asia & MENA	80.6 (75.8-85.2)	81.0 (78.6-83.2)	81.2 (80.1-82.4)	81.0 (80.2-81.8)	80.3 (78.7-81.8)
East & Southeast Asia	78.0 (74.6-81.6)	77.9 (76.5-79.3)	78.2 (77.3-79.1)	78.8 (78.0-79.5)	79.0 (76.9-81.2)
High-income Asia Pacific	80.1 (78.2-82.1)	78.5 (77.4-79.6)	77.3 (76.4-78.2)	76.0 (75.2-76.8)	75.0 (73.4-76.7)
High-income western	82.8 (80.8-84.8)	80.5 (79.7-81.4)	77.8 (77.2-78.4)	76.0 (75.4-76.6)	75.3 (74.0-76.8)
Latin America & Caribbean	81.2 (76.9-85.2)	81.3 (79.1-83.3)	80.7 (79.6-81.9)	79.5 (78.7-80.3)	78.1 (76.4-79.8)
Oceania	72.0 (67.1-76.8)	72.9 (70.6-75.2)	74.3 (72.0-76.7)	75.9 (73.7-78.1)	77.6 (73.4-81.9)
South Asia	78.0 (72.2-83.5)	79.1 (76.4-81.8)	79.9 (78.4-81.5)	80.6 (79.6-81.6)	81.7 (79.8-83.7)
Sub-Saharan Africa	77.7 (74.1-81.1)	79.5 (77.7-81.2)	81.0 (79.7-82.2)	82.0 (81.1-82.9)	82.5 (80.9-84.2)
**50-54 years**					
World	82.1 (80.2-84.1)	81.4 (80.4-82.3)	80.9 (80.3-81.4)	80.7 (80.2-81.1)	80.6 (79.7-81.7)
Central & Eastern Europe	89.1 (85.6-92.8)	88.3 (86.8-89.8)	86.4 (85.3-87.6)	85.1 (83.8-86.4)	84.2 (82.0-86.6)
Central Asia & MENA	82.1 (77.1-86.9)	82.5 (80.1-84.8)	82.8 (81.7-84.0)	82.6 (81.8-83.4)	81.9 (80.3-83.5)
East & Southeast Asia	79.4 (75.8-83.2)	79.3 (77.9-80.7)	79.7 (78.8-80.6)	80.2 (79.4-81.0)	80.4 (78.3-82.7)
High-income Asia Pacific	82.0 (80.0-84.0)	80.3 (79.2-81.4)	79.0 (78.0-79.9)	77.6 (76.7-78.4)	76.4 (74.7-78.2)
High-income western	84.3 (82.2-86.4)	81.8 (80.9-82.7)	79.3 (78.6-79.9)	77.2 (76.5-77.8)	76.4 (74.9-77.9)
Latin America & Caribbean	82.6 (78.1-86.8)	82.6 (80.4-84.7)	82.1 (80.9-83.3)	80.8 (80.0-81.7)	79.4 (77.6-81.2)
Oceania	72.6 (67.5-77.6)	73.6 (71.2-76.0)	75.1 (72.6-77.6)	76.6 (74.2-78.9)	78.6 (74.3-83.0)
South Asia	78.8 (72.8-84.7)	80.0 (77.1-82.9)	80.9 (79.3-82.5)	81.6 (80.5-82.6)	82.8 (80.7-84.9)
Sub-Saharan Africa	78.9 (75.1-82.6)	80.7 (78.9-82.6)	82.4 (81.1-83.7)	83.4 (82.5-84.4)	84.0 (82.3-85.7)
**55-59 years**					
World	82.7 (80.6-84.8)	82.6 (81.6-83.6)	82.1 (81.5-82.6)	81.4 (81.0-81.9)	81.4 (80.5-82.5)
Central & Eastern Europe	90.8 (87.0-94.8)	90.0 (88.4-91.6)	88.1 (86.8-89.3)	86.4 (85.1-87.7)	85.5 (83.2-87.9)
Central Asia & MENA	83.1 (77.8-88.2)	83.6 (81.0-86.0)	83.9 (82.6-85.2)	83.7 (82.8-84.5)	82.9 (81.3-84.6)

Mean diastolic blood pressure (mmHg)					
Super-region (Women)	Year				
	1975	1985	1995	2005	2015
East & Southeast Asia	80.4 (76.6-84.4)	80.2 (78.8-81.7)	80.6 (79.7-81.6)	81.2 (80.4-82.1)	81.4 (79.2-83.7)
High-income Asia Pacific	83.2 (81.1-85.3)	81.5 (80.3-82.6)	80.0 (79.0-81.1)	78.6 (77.7-79.5)	77.4 (75.6-79.3)
High-income western	84.8 (82.7-87.0)	82.5 (81.6-83.5)	80.3 (79.7-81.0)	77.8 (77.2-78.5)	76.8 (75.3-78.5)
Latin America & Caribbean	83.4 (78.8-87.8)	83.5 (81.2-85.7)	82.9 (81.6-84.3)	81.6 (80.7-82.5)	80.1 (78.3-82.0)
Oceania	72.7 (67.3-78.0)	73.7 (71.1-76.3)	75.2 (72.5-77.9)	76.9 (74.3-79.4)	79.0 (74.5-83.6)
South Asia	79.3 (72.9-85.4)	80.6 (77.5-83.5)	81.5 (79.8-83.2)	82.2 (81.0-83.3)	83.4 (81.2-85.7)
Sub-Saharan Africa	79.7 (75.7-83.5)	81.5 (79.6-83.5)	83.3 (81.9-84.6)	84.3 (83.3-85.3)	85.0 (83.2-86.8)
**60-64 years**					
World	83.8 (81.7-85.9)	82.9 (81.9-83.9)	82.2 (81.6-82.8)	81.6 (81.2-82.1)	81.6 (80.6-82.7)
Central & Eastern Europe	91.7 (87.7-95.8)	90.6 (88.9-92.3)	88.7 (87.4-89.9)	87.1 (85.7-88.4)	86.2 (83.8-88.7)
Central Asia & MENA	83.7 (78.3-88.9)	84.0 (81.3-86.5)	84.4 (83.1-85.7)	84.1 (83.2-85.1)	83.3 (81.5-85.0)
East & Southeast Asia	80.7 (76.8-84.9)	80.6 (79.0-82.1)	80.9 (79.9-82.0)	81.6 (80.7-82.5)	81.7 (79.3-84.2)
High-income Asia Pacific	83.7 (81.6-85.9)	81.9 (80.7-83.1)	80.4 (79.4-81.5)	78.9 (77.9-79.9)	77.9 (76.0-80.0)
High-income western	85.5 (83.2-87.8)	82.8 (81.8-83.7)	80.3 (79.6-81.0)	78.0 (77.3-78.7)	76.7 (75.1-78.4)
Latin America & Caribbean	83.7 (78.9-88.2)	83.7 (81.3-86.0)	83.1 (81.7-84.5)	81.7 (80.8-82.6)	80.2 (78.3-82.1)
Oceania	72.2 (66.5-77.8)	73.3 (70.5-76.1)	74.9 (72.0-77.8)	76.6 (73.9-79.3)	78.7 (74.0-83.6)
South Asia	79.3 (72.7-85.6)	80.6 (77.4-83.6)	81.5 (79.8-83.3)	82.3 (81.1-83.4)	83.6 (81.3-85.9)
Sub-Saharan Africa	79.7 (75.6-83.7)	81.7 (79.6-83.7)	83.4 (82.0-84.8)	84.6 (83.5-85.6)	85.3 (83.4-87.1)
**65-69 years**					
World	83.8 (81.7-85.9)	82.3 (81.3-83.3)	82.1 (81.5-82.7)	81.6 (81.1-82.1)	81.1 (80.0-82.1)
Central & Eastern Europe	91.8 (87.7-96.0)	91.0 (89.2-92.8)	88.9 (87.5-90.3)	87.3 (85.8-88.9)	86.1 (83.7-88.6)
Central Asia & MENA	83.5 (77.9-88.9)	83.9 (81.1-86.5)	84.2 (82.8-85.7)	84.0 (83.0-85.1)	83.2 (81.3-85.0)
East & Southeast Asia	80.5 (76.4-84.8)	80.4 (78.8-81.9)	80.7 (79.7-81.8)	81.4 (80.4-82.3)	81.6 (79.1-84.1)
High-income Asia Pacific	83.5 (81.3-85.8)	81.6 (80.4-82.8)	80.1 (79.0-81.2)	78.5 (77.5-79.5)	77.7 (75.6-80.0)
High-income western	85.2 (82.8-87.6)	81.7 (80.7-82.7)	79.7 (79.0-80.4)	77.7 (77.0-78.4)	76.2 (74.5-77.9)
Latin America & Caribbean	83.3 (78.4-88.0)	83.3 (80.8-85.7)	82.7 (81.3-84.2)	81.3 (80.3-82.2)	79.7 (77.7-81.6)
Oceania	71.1 (65.1-77.0)	72.3 (69.2-75.4)	73.9 (70.8-77.1)	75.7 (72.7-78.7)	77.9 (73.0-83.0)
South Asia	78.9 (72.1-85.5)	80.2 (76.9-83.4)	81.2 (79.4-83.0)	81.9 (80.7-83.1)	83.3 (81.0-85.7)
Sub-Saharan Africa	79.2 (74.9-83.3)	81.3 (79.1-83.4)	83.0 (81.6-84.5)	84.2 (83.1-85.3)	84.9 (83.0-86.9)
**70-74 years**					
World	83.4 (81.3-85.6)	82.4 (81.3-83.4)	81.3 (80.7-81.9)	80.8 (80.2-81.3)	80.3 (79.2-81.4)
Central & Eastern Europe	91.6 (87.3-96.0)	90.7 (88.8-92.7)	88.4 (86.9-89.9)	86.8 (85.2-88.3)	85.8 (83.2-88.4)
Central Asia & MENA	83.0 (77.2-88.5)	83.5 (80.5-86.2)	83.8 (82.3-85.3)	83.6 (82.6-84.7)	82.6 (80.7-84.5)
East & Southeast Asia	80.0 (75.7-84.5)	79.8 (78.2-81.5)	80.2 (79.1-81.3)	80.9 (79.9-81.8)	81.0 (78.6-83.7)
High-income Asia Pacific	82.9 (80.7-85.3)	80.9 (79.7-82.2)	79.3 (78.2-80.4)	77.7 (76.6-78.7)	76.9 (74.7-79.3)
High-income western	84.6 (82.1-87.2)	81.5 (80.5-82.6)	78.9 (78.1-79.6)	76.8 (76.1-77.5)	75.2 (73.5-77.0)
Latin America & Caribbean	82.6 (77.4-87.5)	82.6 (80.0-85.1)	82.0 (80.5-83.5)	80.4 (79.4-81.5)	78.8 (76.7-80.8)
Oceania	69.9 (63.6-76.1)	71.0 (67.5-74.5)	72.6 (69.2-76.2)	74.5 (71.2-77.8)	76.9 (71.8-82.2)
South Asia	78.3 (71.4-85.2)	79.7 (76.3-83.0)	80.7 (78.9-82.6)	81.5 (80.2-82.8)	82.9 (80.5-85.4)
Sub-Saharan Africa	78.3 (73.9-82.5)	80.4 (78.2-82.6)	82.3 (80.7-83.8)	83.4 (82.2-84.6)	84.3 (82.2-86.3)
**75-79 years**					
World	82.8 (80.7-85.0)	81.8 (80.7-82.8)	80.1 (79.5-80.8)	79.9 (79.4-80.4)	79.6 (78.5-80.7)
Central & Eastern Europe	91.1 (86.7-95.7)	90.3 (88.2-92.4)	88.1 (86.4-89.9)	86.3 (84.5-88.1)	85.3 (82.5-88.2)
Central Asia & MENA	82.4 (76.3-88.2)	83.0 (79.8-85.9)	83.3 (81.6-84.9)	83.1 (81.9-84.4)	82.3 (80.2-84.3)
East & Southeast Asia	79.4 (75.0-84.0)	79.2 (77.5-80.9)	79.6 (78.4-80.8)	80.3 (79.2-81.3)	80.4 (77.8-83.2)
High-income Asia Pacific	82.1 (79.7-84.5)	80.0 (78.7-81.3)	78.3 (77.1-79.5)	76.7 (75.5-77.8)	75.8 (73.5-78.3)

Mean diastolic blood pressure (mmHg)					
**Super-region (Women)**	**Year**				
	**1975**	**1985**	**1995**	**2005**	**2015**
High-income western	83.7 (81.1-86.3)	80.6 (79.5-81.7)	77.1 (76.3-77.9)	75.6 (74.8-76.4)	74.2 (72.4-76.1)
Latin America & Caribbean	81.9 (76.4-87.1)	81.8 (79.1-84.4)	81.1 (79.5-82.7)	79.6 (78.5-80.7)	77.9 (75.7-80.0)
Oceania	68.7 (62.1-75.2)	69.6 (65.6-73.6)	71.4 (67.5-75.3)	73.2 (69.4-77.0)	75.7 (70.1-81.4)
South Asia	77.9 (70.7-85.1)	79.4 (75.8-82.8)	80.4 (78.4-82.4)	81.2 (79.9-82.6)	82.7 (80.2-85.3)
Sub-Saharan Africa	77.4 (72.8-81.7)	79.5 (77.2-81.7)	81.4 (79.8-83.1)	82.6 (81.3-83.9)	83.5 (81.4-85.6)
**80-84 years**					
World	82.5 (80.3-84.8)	81.2 (80.1-82.3)	79.9 (79.2-80.6)	78.7 (78.2-79.3)	78.5 (77.3-79.6)
Central & Eastern Europe	90.9 (86.2-95.9)	90.0 (87.7-92.3)	87.7 (85.8-89.6)	85.6 (83.7-87.5)	84.5 (81.6-87.4)
Central Asia & MENA	82.1 (75.7-88.2)	82.5 (79.0-85.7)	82.9 (81.0-84.9)	82.7 (81.3-84.1)	81.7 (79.5-83.8)
East & Southeast Asia	78.9 (74.3-83.8)	78.8 (77.0-80.6)	79.1 (77.9-80.4)	79.9 (78.8-80.9)	80.0 (77.2-82.9)
High-income Asia Pacific	81.4 (78.9-83.8)	79.1 (77.8-80.5)	77.3 (76.1-78.6)	75.6 (74.4-76.8)	74.9 (72.4-77.5)
High-income western	82.8 (80.1-85.5)	79.8 (78.6-81.0)	76.9 (76.1-77.8)	74.6 (73.8-75.4)	73.3 (71.4-75.2)
Latin America & Caribbean	81.3 (75.5-86.8)	81.2 (78.4-84.0)	80.5 (78.8-82.2)	78.9 (77.6-80.1)	77.1 (74.8-79.3)
Oceania	67.7 (60.7-74.6)	68.3 (63.6-73.0)	70.0 (65.3-74.7)	71.9 (67.4-76.5)	74.6 (68.5-80.8)
South Asia	78.0 (70.4-85.4)	79.5 (75.7-83.1)	80.6 (78.5-82.7)	81.4 (79.9-82.9)	82.9 (80.2-85.7)
Sub-Saharan Africa	76.5 (71.9-81.0)	78.5 (76.1-80.9)	80.6 (78.8-82.4)	81.8 (80.3-83.3)	82.9 (80.6-85.3)
**85+ years**					
World	82.8 (80.5-85.3)	80.7 (79.5-81.9)	79.0 (78.2-79.8)	77.5 (76.8-78.2)	77.1 (75.8-78.3)
Central & Eastern Europe	91.4 (86.1-97.1)	90.2 (87.3-93.1)	87.7 (85.2-90.1)	85.5 (83.1-87.9)	84.1 (80.7-87.6)
Central Asia & MENA	82.4 (75.1-89.3)	82.6 (78.5-86.5)	83.0 (80.4-85.4)	82.9 (81.0-84.7)	81.9 (79.4-84.5)
East & Southeast Asia	78.7 (73.8-84.0)	78.8 (76.7-80.9)	79.2 (77.7-80.7)	80.0 (78.6-81.3)	80.0 (76.9-83.2)
High-income Asia Pacific	81.0 (78.2-83.7)	78.5 (76.9-80.1)	76.5 (75.0-78.0)	74.6 (73.1-76.1)	74.0 (71.1-77.1)
High-income western	82.4 (79.5-85.4)	78.8 (77.4-80.2)	75.8 (74.8-76.8)	73.1 (72.2-74.1)	72.2 (70.1-74.3)
Latin America & Caribbean	81.2 (75.0-87.3)	81.2 (78.0-84.3)	80.4 (78.3-82.4)	78.6 (77.0-80.3)	76.7 (74.0-79.3)
Oceania	65.5 (56.7-74.1)	66.8 (60.7-72.8)	68.6 (62.4-75.0)	71.1 (65.1-77.0)	73.9 (66.4-81.4)
South Asia	79.1 (70.6-87.3)	80.7 (76.4-84.8)	81.9 (79.3-84.3)	82.7 (80.8-84.7)	84.3 (81.2-87.5)
Sub-Saharan Africa	76.3 (71.2-81.0)	78.2 (75.5-80.9)	80.3 (78.2-82.5)	81.6 (79.6-83.6)	83.1 (80.4-85.9)

Prevalence of raised blood pressure					
**Super-region (Men)**	**Year**				
	**1975**	**1985**	**1995**	**2005**	**2015**
**18-19 years**					
World	8.4% (5.8-12.0)	7.5% (5.8-9.7)	7.3% (5.9-9.0)	6.8% (5.5-8.3)	6.5% (5.0-8.3)
Central & Eastern Europe	18.2% (10.6-28.9)	17.1% (11.7-24.3)	14.8% (10.5-20.2)	12.0% (8.8-16.4)	10.8% (7.1-16.1)
Central Asia & MENA	9.2% (4.4-15.7)	8.5% (5.5-12.3)	7.7% (5.7-10.2)	6.9% (5.3-8.8)	6.3% (4.2-8.9)
East & Southeast Asia	4.7% (2.0-8.4)	4.7% (2.8-6.8)	5.3% (3.7-7.1)	5.4% (3.9-7.3)	5.3% (3.2-7.9)
High-income Asia Pacific	11.3% (7.4-17.0)	9.1% (6.7-12.0)	7.2% (5.3-9.4)	5.6% (3.9-7.4)	4.1% (2.2-6.5)
High-income western	13.1% (8.8-19.1)	11.0% (8.3-14.5)	8.2% (6.5-10.3)	6.0% (4.6-7.5)	4.3% (2.7-6.1)
Latin America & Caribbean	11.1% (6.1-18.2)	10.0% (6.7-14.2)	8.6% (6.2-11.5)	7.1% (5.3-9.3)	6.0% (3.8-8.8)
Oceania	5.2% (1.9-10.5)	5.1% (2.4-8.8)	5.4% (2.7-9.0)	5.7% (3.0-9.2)	6.4% (2.6-11.8)
South Asia	6.4% (2.4-12.3)	6.6% (3.7-10.2)	6.8% (4.4-9.8)	7.2% (5.1-9.8)	7.6% (4.9-11.4)
Sub-Saharan Africa	7.8% (4.0-13.1)	8.2% (5.5-11.8)	8.3% (6.2-11.1)	7.9% (6.0-10.3)	7.6% (5.1-10.7)
**20-24 years**					
World	10.3% (7.7-13.5)	9.6% (7.9-11.7)	9.2% (7.9-10.5)	8.8% (7.8-10.0)	8.3% (6.9-10.0)
Central & Eastern Europe	19.5% (13.1-28.0)	18.8% (14.4-24.3)	16.5% (13.2-20.7)	14.0% (11.2-17.5)	12.7% (9.2-17.4)
Central Asia & MENA	11.2% (6.2-17.3)	10.6% (7.6-14.1)	9.8% (7.8-12.0)	8.9% (7.4-10.5)	8.2% (6.1-10.7)
East & Southeast Asia	6.4% (3.4-10.4)	6.6% (4.6-8.7)	7.2% (5.5-8.9)	7.5% (5.9-9.1)	7.1% (4.8-9.9)
High-income Asia Pacific	13.7% (10.0-18.8)	11.0% (8.8-13.6)	9.3% (7.4-11.3)	7.5% (5.9-9.2)	5.8% (3.8-8.2)
High-income western	14.9% (11.0-19.8)	12.7% (10.5-15.4)	10.4% (8.9-12.1)	7.9% (6.7-9.3)	6.0% (4.3-7.9)
Latin America & Caribbean	13.0% (8.1-19.1)	12.1% (9.0-15.9)	10.6% (8.3-13.1)	9.2% (7.5-11.1)	7.9% (5.7-10.6)
Oceania	6.9% (3.2-12.5)	7.0% (4.1-10.7)	7.3% (4.4-10.9)	7.6% (4.8-11.2)	8.3% (4.3-13.7)
South Asia	8.3% (3.9-14.3)	8.6% (5.5-12.2)	8.9% (6.5-11.7)	9.3% (7.2-11.5)	9.6% (6.9-13.2)
Sub-Saharan Africa	9.7% (5.8-14.9)	10.2% (7.5-13.6)	10.4% (8.3-12.9)	9.9% (8.2-11.9)	9.5% (7.2-12.3)
**25-29 years**					
World	13.9% (10.8-17.8)	13.1% (11.1-15.5)	12.4% (10.8-14.0)	12.1% (10.7-13.5)	11.3% (9.6-13.3)
Central & Eastern Europe	23.6% (16.9-32.5)	22.9% (18.2-28.7)	20.5% (16.9-24.9)	17.9% (14.9-21.5)	16.4% (12.4-21.5)
Central Asia & MENA	14.7% (8.9-21.4)	14.2% (10.6-18.2)	13.2% (10.9-15.9)	12.2% (10.4-14.1)	11.2% (8.6-14.1)
East & Southeast Asia	9.2% (5.3-13.9)	9.5% (7.0-12.0)	10.1% (8.1-12.2)	10.5% (8.6-12.4)	9.9% (6.9-13.3)
High-income Asia Pacific	18.0% (13.7-23.6)	14.6% (12.1-17.5)	12.5% (10.3-14.8)	10.7% (8.7-12.6)	8.6% (6.0-11.5)
High-income western	18.9% (14.6-24.5)	16.0% (13.6-19.0)	13.9% (12.1-15.9)	11.1% (9.5-12.7)	8.8% (6.6-11.0)
Latin America & Caribbean	16.8% (11.4-23.6)	15.8% (12.3-19.8)	14.0% (11.5-16.9)	12.4% (10.4-14.5)	11.0% (8.2-14.1)
Oceania	9.7% (4.9-16.2)	9.9% (6.3-14.1)	10.2% (6.7-14.3)	10.6% (7.2-14.7)	11.4% (6.6-17.4)
South Asia	11.4% (6.1-18.2)	11.7% (8.2-15.8)	12.0% (9.4-15.1)	12.5% (10.2-15.0)	12.9% (9.7-16.8)
Sub-Saharan Africa	13.0% (8.4-18.9)	13.7% (10.5-17.3)	13.9% (11.5-16.6)	13.3% (11.3-15.5)	12.8% (10.1-15.9)
**30-34 years**					
World	17.9% (14.0-22.6)	16.9% (14.4-19.7)	16.1% (14.3-18.0)	15.4% (13.8-17.0)	14.7% (12.6-17.0)
Central & Eastern Europe	29.7% (21.7-39.4)	29.1% (23.9-35.3)	26.2% (22.0-31.0)	22.9% (19.4-27.1)	21.1% (16.3-27.1)
Central Asia & MENA	18.9% (11.6-27.1)	18.4% (13.8-23.4)	17.1% (14.3-20.3)	15.8% (13.6-18.2)	14.3% (11.1-17.9)
East & Southeast Asia	12.0% (7.1-18.0)	12.2% (9.2-15.3)	13.2% (10.8-15.7)	13.6% (11.2-15.9)	13.0% (9.4-17.1)
High-income Asia Pacific	23.1% (17.9-29.7)	19.4% (16.4-22.9)	16.2% (13.5-18.9)	13.9% (11.6-16.3)	11.2% (8.0-14.8)
High-income western	24.4% (19.1-31.0)	20.7% (17.7-24.3)	17.6% (15.5-19.8)	14.6% (12.8-16.4)	11.5% (8.8-14.3)
Latin America & Caribbean	21.6% (14.8-29.7)	20.3% (15.9-25.1)	18.2% (15.1-21.6)	16.0% (13.6-18.5)	14.3% (10.8-18.1)
Oceania	12.5% (6.5-20.7)	12.8% (8.4-18.1)	13.3% (9.0-18.2)	13.8% (9.4-18.7)	14.7% (8.6-22.2)
South Asia	14.7% (8.0-23.2)	15.1% (10.8-20.1)	15.5% (12.2-19.2)	16.1% (13.4-19.1)	16.5% (12.5-21.3)
Sub-Saharan Africa	16.8% (11.0-24.1)	17.6% (13.8-22.1)	17.9% (15.0-21.1)	17.3% (14.8-19.9)	16.5% (13.2-20.2)
**35-39 years**					
World	23.1% (18.0-29.0)	21.7% (18.6-25.0)	20.4% (18.4-22.6)	19.1% (17.3-21.0)	18.3% (15.7-21.1)

Prevalence of raised blood pressure					
Super-region (Men)	Year				
	1975	1985	1995	2005	2015
Central & Eastern Europe	37.8% (27.6-49.1)	36.9% (30.8-43.6)	33.3% (28.5-38.6)	29.0% (24.9-33.9)	26.7% (20.7-33.8)
Central Asia & MENA	23.9% (14.4-34.4)	23.1% (17.3-29.3)	21.5% (17.9-25.5)	19.8% (17.1-22.7)	17.8% (13.8-22.4)
East & Southeast Asia	14.9% (8.6-22.7)	15.2% (11.6-19.1)	16.5% (13.6-19.4)	16.9% (14.0-19.8)	16.2% (11.7-21.5)
High-income Asia Pacific	29.2% (22.7-36.9)	25.1% (21.3-29.3)	20.4% (17.2-23.7)	17.2% (14.5-20.0)	13.9% (9.9-18.5)
High-income western	31.4% (24.6-39.4)	26.5% (22.8-30.7)	21.7% (19.2-24.4)	18.3% (16.1-20.4)	14.3% (11.1-17.7)
Latin America & Caribbean	27.3% (18.5-37.3)	25.8% (20.2-31.7)	23.0% (19.0-27.1)	20.1% (17.1-23.1)	17.7% (13.4-22.6)
Oceania	15.5% (7.8-26.0)	15.8% (10.1-22.7)	16.5% (11.0-22.8)	17.2% (11.7-23.3)	18.3% (10.4-27.9)
South Asia	18.3% (9.7-29.1)	18.8% (13.3-25.0)	19.3% (15.2-23.9)	20.1% (16.7-23.8)	20.6% (15.5-26.7)
Sub-Saharan Africa	21.1% (13.6-30.6)	22.2% (17.3-27.8)	22.6% (18.9-26.7)	21.8% (18.7-24.9)	20.7% (16.4-25.4)
**40-44 years**					
World	28.2% (21.9-35.2)	26.7% (22.8-30.8)	25.1% (22.5-27.9)	23.7% (21.3-26.0)	22.1% (18.6-25.8)
Central & Eastern Europe	46.0% (34.2-58.3)	45.1% (38.3-52.5)	41.0% (35.4-47.1)	35.9% (30.7-41.8)	33.0% (25.6-41.6)
Central Asia & MENA	29.2% (17.1-42.0)	28.5% (21.1-36.0)	26.6% (22.1-31.3)	24.4% (21.1-28.1)	21.9% (16.9-27.6)
East & Southeast Asia	18.2% (10.2-28.0)	18.7% (14.1-23.7)	20.1% (16.4-23.9)	20.8% (17.2-24.5)	19.7% (13.8-26.8)
High-income Asia Pacific	36.5% (28.4-45.7)	31.1% (26.4-36.2)	25.9% (21.9-30.0)	21.0% (17.6-24.5)	17.1% (12.0-23.0)
High-income western	38.4% (30.2-47.5)	33.3% (28.6-38.1)	27.0% (23.7-30.2)	22.2% (19.5-24.8)	17.6% (13.6-22.0)
Latin America & Caribbean	33.8% (22.9-45.8)	31.8% (25.0-39.0)	28.4% (23.6-33.6)	24.9% (21.1-28.8)	21.7% (16.3-27.8)
Oceania	18.9% (9.1-32.0)	19.4% (12.1-27.9)	20.3% (13.2-28.3)	21.1% (14.2-28.7)	22.6% (12.6-34.5)
South Asia	22.4% (11.5-35.7)	23.1% (16.1-30.9)	23.7% (18.4-29.5)	24.7% (20.4-29.3)	25.3% (18.8-32.9)
Sub-Saharan Africa	26.0% (16.5-37.6)	27.5% (21.2-34.6)	27.9% (23.5-33.0)	26.9% (23.1-30.9)	25.5% (20.1-31.4)
**45-49 years**					
World	34.7% (27.5-42.4)	33.2% (28.7-37.9)	31.1% (28.1-34.1)	29.1% (26.5-31.8)	26.8% (22.7-31.3)
Central & Eastern Europe	53.9% (41.3-66.2)	52.9% (45.7-60.6)	48.5% (42.8-54.7)	43.0% (37.4-49.2)	39.7% (31.5-48.8)
Central Asia & MENA	35.4% (21.4-49.5)	34.5% (26.0-42.9)	32.2% (27.0-37.7)	29.9% (26.1-33.9)	27.0% (21.0-33.5)
East & Southeast Asia	22.5% (13.1-34.1)	23.3% (17.7-29.0)	25.0% (20.7-29.4)	25.8% (21.7-30.1)	24.4% (17.1-32.9)
High-income Asia Pacific	44.0% (35.1-53.9)	37.5% (32.2-43.2)	32.2% (27.7-36.9)	26.0% (22.0-30.0)	21.0% (15.1-27.9)
High-income western	45.3% (36.4-54.5)	40.7% (35.6-46.1)	33.2% (29.5-36.7)	26.8% (23.8-29.9)	21.9% (17.1-27.0)
Latin America & Caribbean	40.6% (28.2-53.4)	38.5% (30.6-46.2)	34.6% (29.1-40.2)	30.5% (26.2-35.0)	26.8% (20.4-33.8)
Oceania	23.4% (11.6-38.7)	24.0% (15.1-34.0)	25.0% (16.6-34.3)	26.0% (17.9-34.8)	27.6% (16.1-40.9)
South Asia	27.4% (14.4-42.6)	28.3% (20.0-37.4)	29.1% (23.0-35.7)	30.2% (25.2-35.5)	30.9% (23.2-39.5)
Sub-Saharan Africa	31.6% (20.4-44.7)	33.4% (26.2-41.4)	33.9% (28.8-39.7)	32.8% (28.4-37.3)	31.1% (24.9-37.8)
**50-54 years**					
World	40.5% (33.1-48.5)	39.2% (34.5-44.0)	37.3% (34.2-40.5)	35.2% (32.5-38.0)	32.8% (28.6-37.4)
Central & Eastern Europe	59.8% (47.8-71.0)	59.3% (52.5-66.1)	55.1% (49.7-60.7)	49.7% (44.3-55.5)	46.2% (37.3-55.4)
Central Asia & MENA	41.6% (26.9-55.7)	40.6% (31.8-49.1)	38.6% (33.1-44.2)	36.2% (32.3-40.3)	33.1% (26.8-40.0)
East & Southeast Asia	28.1% (17.4-40.7)	28.9% (23.0-35.1)	31.0% (26.4-35.7)	31.8% (27.3-36.4)	30.3% (22.2-39.4)
High-income Asia Pacific	50.1% (41.2-59.7)	44.6% (39.1-50.3)	38.7% (33.9-43.7)	32.9% (28.5-37.3)	26.3% (19.7-33.5)
High-income western	51.6% (42.7-60.5)	46.9% (41.8-52.3)	40.1% (36.4-43.8)	32.8% (29.6-36.0)	27.1% (21.9-32.7)
Latin America & Caribbean	47.1% (34.5-59.4)	45.1% (37.0-52.8)	41.0% (35.3-46.7)	36.8% (32.3-41.4)	32.9% (25.8-40.3)
Oceania	29.1% (15.6-45.2)	29.7% (19.7-40.5)	30.9% (21.5-40.9)	32.0% (22.9-41.5)	33.6% (21.1-47.2)
South Asia	33.4% (19.1-49.2)	34.4% (25.5-44.0)	35.4% (28.9-42.2)	36.4% (31.1-42.0)	37.1% (28.9-46.1)
Sub-Saharan Africa	37.7% (25.6-51.2)	39.8% (32.0-47.9)	40.5% (35.1-46.3)	39.2% (34.7-43.9)	37.4% (30.8-44.3)
**55-59 years**					
World	45.3% (37.6-53.5)	45.8% (41.1-50.7)	44.1% (40.9-47.4)	41.6% (38.8-44.4)	39.0% (34.6-43.7)
Central & Eastern Europe	65.1% (53.9-75.8)	64.7% (58.2-71.3)	60.6% (54.8-66.3)	55.7% (50.3-61.2)	52.3% (43.8-61.0)
Central Asia & MENA	47.7% (32.9-61.6)	46.9% (38.3-55.4)	44.8% (39.3-50.6)	42.4% (38.3-46.7)	39.4% (32.7-46.3)

Prevalence of raised blood pressure					
Super-region (Men)	Year				
	1975	1985	1995	2005	2015
East & Southeast Asia	34.1% (22.4-47.1)	35.2% (28.7-41.5)	37.3% (32.4-42.2)	38.1% (33.3-42.9)	36.5% (28.1-45.6)
High-income Asia Pacific	56.0% (47.4-65.3)	51.2% (45.8-56.8)	45.0% (40.1-50.1)	39.8% (35.1-44.5)	32.4% (25.1-40.0)
High-income western	56.2% (47.6-64.6)	52.5% (47.4-57.8)	47.1% (43.4-50.9)	39.3% (35.9-42.6)	32.7% (27.0-38.8)
Latin America & Caribbean	53.1% (40.6-64.8)	51.3% (43.4-59.0)	47.3% (41.6-53.0)	43.1% (38.5-47.7)	39.1% (31.8-46.5)
Oceania	35.2% (20.4-51.3)	35.9% (25.1-46.9)	37.1% (26.9-47.3)	38.3% (28.6-47.7)	39.8% (26.7-52.9)
South Asia	39.4% (24.3-55.3)	40.6% (31.3-50.1)	41.6% (34.9-48.4)	42.7% (37.2-48.1)	43.3% (34.7-52.2)
Sub-Saharan Africa	43.8% (31.5-57.0)	45.9% (38.0-54.0)	46.7% (41.3-52.3)	45.4% (40.8-50.2)	43.6% (36.9-50.4)
**60-64 years**					
World	52.2% (44.8-59.9)	50.7% (46.0-55.4)	49.2% (45.9-52.4)	46.9% (44.0-49.8)	44.1% (39.3-48.9)
Central & Eastern Europe	69.1% (58.8-78.9)	68.6% (62.5-75.0)	65.1% (59.7-70.4)	60.6% (55.4-65.6)	57.3% (49.2-65.4)
Central Asia & MENA	52.7% (38.4-65.7)	52.1% (43.4-60.3)	50.0% (44.5-55.6)	47.7% (43.5-51.9)	44.6% (37.6-51.6)
East & Southeast Asia	39.5% (27.3-52.5)	40.6% (33.9-47.1)	42.7% (37.7-47.7)	43.6% (38.7-48.3)	41.8% (32.7-51.2)
High-income Asia Pacific	61.3% (53.2-70.0)	56.1% (51.0-61.4)	50.7% (45.9-55.6)	45.3% (40.6-50.0)	39.0% (31.0-47.0)
High-income western	62.1% (54.0-69.7)	57.1% (52.2-62.1)	51.9% (48.2-55.6)	45.1% (41.7-48.5)	38.1% (32.0-44.4)
Latin America & Caribbean	58.0% (46.0-69.1)	56.3% (48.7-63.5)	52.5% (46.8-58.0)	48.3% (43.8-52.8)	44.4% (37.1-51.7)
Oceania	40.5% (25.2-56.5)	41.4% (30.0-52.2)	42.5% (32.0-52.4)	43.6% (33.5-52.9)	45.1% (32.0-58.0)
South Asia	44.6% (29.2-60.0)	45.9% (36.5-55.3)	46.8% (40.1-53.5)	48.1% (42.6-53.4)	48.5% (39.8-57.1)
Sub-Saharan Africa	49.0% (36.7-61.6)	51.1% (43.3-58.8)	51.8% (46.4-57.2)	50.7% (46.1-55.3)	48.9% (42.2-55.6)
**65-69 years**					
World	56.7% (49.5-64.1)	53.5% (48.8-58.2)	53.4% (50.2-56.5)	51.2% (48.4-54.0)	47.8% (43.2-52.2)
Central & Eastern Europe	72.0% (62.2-81.1)	71.9% (65.9-77.9)	68.5% (63.2-73.7)	64.0% (58.7-69.4)	61.2% (53.3-68.8)
Central Asia & MENA	56.7% (42.4-69.2)	56.0% (47.5-64.2)	54.0% (48.6-59.4)	51.7% (47.6-55.8)	48.5% (41.6-55.3)
East & Southeast Asia	43.5% (30.8-56.4)	44.6% (37.6-51.1)	46.8% (41.7-51.8)	47.7% (42.8-52.4)	45.8% (36.7-55.0)
High-income Asia Pacific	65.3% (57.4-73.6)	60.1% (55.1-65.3)	55.2% (50.4-59.9)	49.5% (44.8-54.0)	43.9% (35.6-52.2)
High-income western	65.8% (58.0-73.5)	59.4% (54.5-64.4)	55.4% (51.7-58.9)	49.8% (46.4-53.0)	42.3% (36.2-48.4)
Latin America & Caribbean	61.7% (50.1-72.4)	60.1% (52.8-67.2)	56.5% (51.0-61.8)	52.3% (47.9-56.8)	48.3% (41.1-55.4)
Oceania	44.5% (28.7-60.2)	45.5% (33.9-56.3)	46.6% (35.9-56.5)	47.7% (37.6-56.8)	49.0% (35.5-61.6)
South Asia	48.4% (32.5-63.4)	49.8% (40.4-58.9)	50.7% (43.8-57.5)	51.9% (46.5-57.2)	52.4% (43.8-60.8)
Sub-Saharan Africa	52.7% (40.3-64.9)	54.9% (47.1-62.5)	55.6% (50.2-61.1)	54.7% (50.1-59.3)	52.8% (46.1-59.2)
70-74 years					
World	59.7% (52.5-66.9)	57.8% (53.1-62.5)	55.7% (52.3-59.2)	53.6% (50.6-56.6)	50.3% (45.5-54.7)
Central & Eastern Europe	74.1% (64.2-83.1)	74.0% (67.5-80.5)	70.9% (65.3-76.5)	66.6% (61.2-72.1)	63.7% (56.0-71.4)
Central Asia & MENA	59.1% (44.6-71.6)	58.5% (50.0-66.6)	56.6% (51.1-62.3)	54.2% (49.9-58.3)	50.8% (43.6-57.8)
East & Southeast Asia	46.1% (32.5-59.2)	47.3% (40.0-54.1)	49.5% (44.1-54.8)	50.4% (45.3-55.3)	48.6% (39.1-57.8)
High-income Asia Pacific	68.4% (60.3-76.7)	63.3% (58.0-68.8)	57.9% (53.0-62.9)	52.6% (47.8-57.3)	46.8% (38.2-55.4)
High-income western	68.6% (60.7-76.6)	63.5% (58.4-68.7)	57.7% (53.8-61.5)	52.3% (48.8-55.6)	45.2% (38.9-51.3)
Latin America & Caribbean	64.0% (52.5-74.9)	62.6% (55.2-69.7)	58.9% (53.2-64.6)	54.8% (50.2-59.4)	50.8% (43.5-57.9)
Oceania	47.1% (31.3-62.5)	48.2% (36.4-59.2)	49.3% (38.4-59.2)	50.3% (40.2-59.4)	51.5% (37.6-64.1)
South Asia	50.8% (34.8-65.6)	52.2% (42.5-61.5)	53.2% (46.0-60.2)	54.3% (48.5-60.1)	54.9% (46.2-63.4)
Sub-Saharan Africa	55.1% (42.5-67.4)	57.4% (49.4-65.0)	58.1% (52.5-63.9)	57.2% (52.2-62.0)	55.4% (48.6-62.0)
**75-79 years**					
World	61.5% (54.2-68.9)	59.7% (54.9-64.7)	56.3% (52.5-60.0)	55.1% (51.8-58.3)	51.7% (46.8-56.5)
Central & Eastern Europe	75.1% (65.1-84.6)	75.1% (68.0-82.2)	72.1% (65.9-78.3)	67.8% (61.9-73.9)	64.8% (56.7-73.0)
Central Asia & MENA	60.7% (46.2-73.1)	60.1% (51.3-68.3)	58.1% (52.3-64.1)	55.7% (51.1-60.1)	52.2% (44.9-59.2)
East & Southeast Asia	47.6% (33.4-60.9)	48.8% (40.8-56.0)	51.0% (44.9-56.7)	51.9% (46.3-57.3)	50.0% (40.0-59.6)
High-income Asia Pacific	70.1% (61.8-78.8)	65.2% (59.6-71.1)	59.7% (54.4-65.2)	54.7% (49.4-59.9)	48.5% (39.4-57.5)

Prevalence of raised blood pressure					
**Super-region (Men)**	**Year**				
	**1975**	**1985**	**1995**	**2005**	**2015**
High-income western	69.4% (61.3-77.7)	65.0% (59.7-70.5)	57.4% (53.4-61.5)	53.4% (49.6-57.0)	47.1% (40.5-53.3)
Latin America & Caribbean	65.3% (53.5-76.2)	63.9% (56.3-71.3)	60.3% (54.4-66.1)	56.2% (51.3-61.1)	52.0% (44.3-59.4)
Oceania	48.6% (32.4-63.9)	49.7% (37.6-60.7)	50.7% (39.8-60.8)	51.7% (41.4-61.2)	52.9% (38.9-66.1)
South Asia	51.8% (35.4-66.9)	53.2% (42.9-63.0)	54.3% (46.5-61.8)	55.4% (48.9-61.8)	55.9% (46.8-64.9)
Sub-Saharan Africa	56.4% (43.6-68.7)	58.6% (50.6-66.4)	59.3% (53.2-65.4)	58.6% (53.4-63.9)	56.9% (49.6-63.7)
80-84 years					
World	62.5% (55.4-69.9)	60.4% (55.4-65.2)	58.0% (54.2-61.6)	55.1% (51.7-58.4)	51.6% (46.5-56.4)
Central & Eastern Europe	75.6% (65.1-85.4)	75.3% (67.5-82.7)	72.4% (65.4-79.1)	68.3% (62.2-74.5)	65.3% (57.3-73.8)
Central Asia & MENA	61.5% (47.2-74.0)	60.3% (51.6-68.8)	58.4% (52.5-64.4)	55.9% (51.0-60.8)	52.2% (44.5-59.6)
East & Southeast Asia	48.0% (33.3-61.5)	49.2% (40.3-57.3)	51.4% (44.7-58.2)	52.4% (46.1-58.6)	50.4% (39.7-60.8)
High-income Asia Pacific	70.9% (61.9-79.8)	66.3% (59.7-72.9)	60.8% (54.7-66.9)	55.5% (49.3-61.6)	49.8% (39.9-59.5)
High-income western	68.9% (60.3-77.9)	65.4% (59.6-71.3)	59.5% (55.4-63.7)	53.5% (49.8-57.4)	47.3% (40.4-53.7)
Latin America & Caribbean	65.5% (53.6-76.5)	63.9% (56.0-71.6)	60.3% (54.0-66.4)	56.3% (50.7-61.5)	52.1% (44.1-59.8)
Oceania	49.5% (33.2-64.9)	50.0% (37.7-61.4)	51.0% (39.9-61.2)	52.0% (41.5-61.5)	53.1% (38.8-66.0)
South Asia	51.8% (34.7-67.4)	53.1% (41.8-63.5)	54.1% (45.3-62.3)	55.3% (47.4-62.5)	55.7% (45.9-65.4)
Sub-Saharan Africa	56.7% (43.7-69.0)	58.6% (50.2-66.7)	59.4% (53.3-65.7)	58.7% (53.2-64.1)	57.3% (49.7-64.3)
**85+ years**					
World	62.3% (52.3-73.2)	59.2% (51.3-67.6)	56.8% (50.1-63.6)	53.3% (47.4-59.4)	50.2% (43.4-56.9)
Central & Eastern Europe	73.8% (58.2-88.3)	73.6% (59.0-86.7)	70.7% (57.3-83.1)	66.8% (55.1-78.1)	63.9% (52.5-76.4)
Central Asia & MENA	60.7% (45.5-76.0)	59.4% (48.5-71.0)	57.5% (49.0-66.7)	55.1% (47.4-63.0)	51.5% (42.0-61.0)
East & Southeast Asia	47.7% (31.9-62.5)	48.5% (37.2-59.9)	50.7% (40.7-61.1)	51.6% (41.7-61.8)	49.9% (37.3-62.2)
High-income Asia Pacific	70.7% (56.7-84.2)	66.2% (54.5-77.9)	60.9% (50.4-71.8)	55.7% (45.6-66.1)	50.4% (37.3-63.4)
High-income western	67.2% (54.0-80.6)	62.5% (52.6-72.4)	57.7% (50.5-64.9)	51.2% (45.5-57.0)	46.0% (37.6-53.6)
Latin America & Caribbean	63.3% (49.4-77.5)	62.0% (50.6-73.6)	58.8% (49.2-68.2)	55.1% (46.4-63.5)	51.2% (40.8-61.4)
Oceania	48.3% (30.3-65.7)	48.9% (34.9-62.3)	50.1% (36.9-62.7)	51.1% (38.4-63.3)	52.1% (36.4-67.4)
South Asia	50.2% (30.9-68.8)	51.2% (36.5-65.3)	52.1% (39.2-64.3)	53.2% (40.9-64.6)	53.6% (40.0-67.1)
Sub-Saharan Africa	56.1% (42.4-70.4)	57.4% (47.3-68.0)	58.2% (49.5-67.4)	57.4% (49.2-66.4)	56.3% (47.0-66.2)

Prevalence of raised blood pressure					
**Super-region (Women)**	**Year**				
	**1975**	**1985**	**1995**	**2005**	**2015**
**18-19 years**					
World	3.7% (2.0-6.5)	3.1% (2.0-4.6)	3.0% (2.1-4.2)	2.6% (1.9-3.6)	2.7% (1.8-4.0)
Central & Eastern Europe	10.1% (4.0-20.4)	8.0% (4.2-13.8)	5.6% (3.3-8.8)	3.4% (2.0-5.4)	2.4% (1.1-4.4)
Central Asia & MENA	6.0% (2.1-13.2)	5.5% (2.8-9.7)	4.7% (2.8-7.5)	3.5% (2.3-5.2)	2.7% (1.5-4.5)
East & Southeast Asia	1.4% (0.4-3.3)	1.4% (0.7-2.3)	1.7% (1.0-2.5)	1.5% (1.0-2.3)	1.5% (0.7-2.7)
High-income Asia Pacific	3.1% (1.5-6.0)	1.7% (1.0-2.8)	1.0% (0.5-1.6)	0.5% (0.2-0.9)	0.3% (0.1-0.7)
High-income western	4.3% (2.2-7.9)	2.9% (1.9-4.5)	1.8% (1.2-2.5)	0.9% (0.6-1.4)	0.5% (0.2-0.9)
Latin America & Caribbean	6.3% (2.4-13.8)	4.9% (2.6-8.8)	3.5% (2.1-5.7)	2.2% (1.3-3.3)	1.4% (0.7-2.5)
Oceania	1.7% (0.4-4.4)	1.7% (0.6-3.6)	2.0% (0.8-4.1)	2.4% (1.0-4.8)	3.2% (0.9-8.2)
South Asia	2.8% (0.7-7.5)	3.0% (1.3-6.0)	3.3% (1.8-5.7)	3.5% (2.1-5.5)	3.7% (1.9-6.7)
Sub-Saharan Africa	3.5% (1.3-7.5)	3.9% (2.2-6.7)	4.4% (2.7-6.9)	4.4% (2.8-6.7)	4.5% (2.5-7.6)
**20-24 years**					
World	4.2% (2.7-6.5)	3.8% (2.8-5.0)	3.6% (2.8-4.4)	3.4% (2.7-4.1)	3.2% (2.4-4.2)
Central & Eastern Europe	10.0% (5.1-17.6)	8.6% (5.6-12.6)	6.3% (4.4-8.7)	4.2% (2.9-5.8)	3.1% (1.7-5.0)
Central Asia & MENA	6.5% (2.9-12.4)	6.1% (3.7-9.4)	5.4% (3.8-7.5)	4.3% (3.3-5.6)	3.3% (2.2-4.9)
East & Southeast Asia	2.0% (0.8-3.9)	2.0% (1.2-3.1)	2.3% (1.6-3.1)	2.3% (1.6-3.0)	2.0% (1.2-3.2)
High-income Asia Pacific	4.0% (2.3-6.6)	2.5% (1.6-3.5)	1.6% (1.0-2.3)	1.0% (0.6-1.4)	0.5% (0.2-1.0)
High-income western	5.0% (3.0-7.9)	3.6% (2.6-4.8)	2.5% (1.9-3.2)	1.5% (1.1-1.9)	0.9% (0.5-1.4)
Latin America & Caribbean	6.8% (3.3-12.6)	5.6% (3.5-8.8)	4.3% (3.0-6.1)	2.9% (2.1-4.0)	2.0% (1.2-3.2)
Oceania	2.3% (0.7-5.1)	2.4% (1.1-4.4)	2.7% (1.3-4.9)	3.2% (1.6-5.6)	3.9% (1.5-8.1)
South Asia	3.5% (1.2-7.9)	3.8% (2.0-6.4)	4.1% (2.6-6.2)	4.3% (2.9-6.0)	4.5% (2.7-7.0)
Sub-Saharan Africa	4.2% (2.0-7.7)	4.6% (3.0-7.0)	5.1% (3.6-7.1)	5.1% (3.7-6.9)	5.1% (3.4-7.6)
**25-29 years**					
World	6.2% (4.4-8.8)	5.8% (4.5-7.3)	5.3% (4.4-6.3)	5.1% (4.3-6.0)	4.7% (3.7-5.8)
Central & Eastern Europe	12.5% (7.3-20.2)	11.1% (7.9-15.3)	8.7% (6.6-11.3)	6.3% (4.8-8.3)	4.8% (3.0-7.2)
Central Asia & MENA	8.8% (4.6-15.1)	8.4% (5.7-12.0)	7.7% (5.8-10.0)	6.4% (5.1-7.9)	5.1% (3.6-6.9)
East & Southeast Asia	3.4% (1.6-6.0)	3.6% (2.4-4.9)	3.8% (2.8-4.9)	3.9% (2.9-4.9)	3.4% (2.1-5.1)
High-income Asia Pacific	6.2% (4.0-9.3)	4.2% (3.1-5.6)	2.9% (2.1-3.9)	2.0% (1.4-2.8)	1.3% (0.6-2.2)
High-income western	7.2% (4.8-10.5)	5.3% (4.1-6.8)	4.1% (3.3-5.0)	2.8% (2.2-3.4)	1.9% (1.2-2.7)
Latin America & Caribbean	9.1% (5.1-15.2)	7.9% (5.4-11.4)	6.3% (4.8-8.3)	4.7% (3.6-6.0)	3.4% (2.3-5.0)
Oceania	3.8% (1.5-7.4)	4.0% (2.2-6.5)	4.5% (2.6-7.1)	5.0% (3.0-7.9)	5.9% (2.8-10.7)
South Asia	5.3% (2.2-10.2)	5.7% (3.5-8.7)	6.1% (4.2-8.4)	6.3% (4.7-8.3)	6.5% (4.4-9.5)
Sub-Saharan Africa	6.1% (3.3-10.1)	6.7% (4.7-9.5)	7.3% (5.5-9.6)	7.3% (5.7-9.3)	7.3% (5.3-9.9)
**30-34 years**					
World	9.7% (7.0-13.2)	9.0% (7.3-11.0)	8.5% (7.2-9.8)	7.8% (6.8-8.9)	7.4% (6.1-9.0)
Central & Eastern Europe	18.1% (11.6-27.2)	16.4% (12.4-21.6)	13.4% (10.7-16.9)	10.0% (7.9-12.6)	7.8% (5.1-11.1)
Central Asia & MENA	13.3% (7.6-21.3)	12.8% (9.2-17.5)	11.7% (9.2-14.7)	10.0% (8.3-12.0)	8.0% (5.9-10.6)
East & Southeast Asia	5.8% (3.0-9.6)	5.9% (4.1-7.8)	6.4% (5.0-7.9)	6.2% (4.9-7.7)	5.8% (3.9-8.1)
High-income Asia Pacific	10.1% (6.9-14.3)	7.2% (5.5-9.1)	5.1% (3.9-6.5)	3.7% (2.7-4.8)	2.4% (1.3-3.8)
High-income western	11.3% (7.9-15.7)	8.6% (6.8-10.6)	6.7% (5.5-7.9)	4.8% (3.9-5.8)	3.3% (2.3-4.6)
Latin America & Caribbean	13.6% (8.3-21.2)	12.0% (8.7-16.5)	9.9% (7.8-12.5)	7.5% (6.1-9.2)	5.7% (3.9-7.9)
Oceania	6.2% (2.8-11.2)	6.6% (3.8-10.1)	7.3% (4.5-10.8)	8.1% (5.2-11.9)	9.2% (4.9-15.6)
South Asia	8.3% (3.9-15.0)	8.9% (5.7-12.9)	9.4% (6.9-12.5)	9.7% (7.6-12.3)	10.0% (7.0-13.8)
Sub-Saharan Africa	9.5% (5.5-15.0)	10.5% (7.6-14.2)	11.3% (8.9-14.2)	11.3% (9.1-13.9)	11.1% (8.5-14.5)
**35-39 years**					
World	15.6% (11.6-20.7)	14.1% (11.7-17.0)	13.3% (11.7-15.2)	12.0% (10.6-13.5)	11.4% (9.5-13.6)

Prevalence of raised blood pressure					
Super-region (Women)	Year				
	1975	1985	1995	2005	2015
Central & Eastern Europe	27.7% (18.5-39.1)	25.0% (19.8-31.2)	20.6% (17.0-24.9)	15.7% (12.7-19.2)	12.4% (8.4-17.1)
Central Asia & MENA	20.2% (12.1-30.9)	19.6% (14.5-26.1)	18.0% (14.6-22.1)	15.5% (13.1-18.2)	12.6% (9.4-16.2)
East & Southeast Asia	9.2% (4.9-15.0)	9.4% (6.8-12.3)	10.2% (8.2-12.4)	9.9% (8.0-11.9)	9.2% (6.4-12.8)
High-income Asia Pacific	16.1% (11.3-22.1)	11.9% (9.4-14.6)	8.4% (6.6-10.4)	6.0% (4.6-7.5)	4.0% (2.4-6.1)
High-income western	18.0% (13.0-24.1)	13.6% (11.1-16.5)	10.4% (8.8-12.1)	7.8% (6.5-9.1)	5.4% (3.8-7.2)
Latin America & Caribbean	20.7% (13.0-31.0)	18.5% (13.8-24.5)	15.4% (12.3-18.9)	11.8% (9.7-14.2)	9.0% (6.4-12.2)
Oceania	9.7% (4.5-17.4)	10.4% (6.2-15.6)	11.5% (7.4-16.5)	12.6% (8.4-17.9)	14.4% (7.9-23.5)
South Asia	12.7% (6.2-22.2)	13.6% (9.1-19.3)	14.4% (10.8-18.7)	14.9% (12.0-18.3)	15.2% (11.0-20.5)
Sub-Saharan Africa	14.7% (8.8-22.6)	16.2% (12.1-21.3)	17.4% (14.1-21.3)	17.5% (14.6-20.8)	17.2% (13.3-21.6)
**40-44 years**					
World	22.7% (17.1-29.3)	21.0% (17.5-25.1)	19.7% (17.3-22.2)	17.8% (16.0-19.9)	16.3% (13.5-19.4)
Central & Eastern Europe	39.0% (27.1-52.7)	35.8% (29.0-43.5)	30.3% (25.5-35.8)	23.6% (19.1-28.6)	18.8% (12.9-25.5)
Central Asia & MENA	29.3% (18.0-43.2)	28.8% (21.7-37.4)	26.6% (21.9-32.0)	23.0% (19.6-26.7)	18.8% (14.1-23.9)
East & Southeast Asia	14.0% (7.6-22.5)	14.5% (10.7-18.8)	15.4% (12.5-18.5)	15.1% (12.4-18.1)	13.6% (9.3-19.2)
High-income Asia Pacific	24.5% (17.6-32.8)	18.3% (14.7-22.3)	13.3% (10.6-16.1)	9.3% (7.3-11.6)	6.3% (3.7-9.5)
High-income western	26.4% (19.5-34.6)	20.9% (17.3-25.0)	15.9% (13.6-18.4)	11.7% (9.9-13.7)	8.3% (5.9-11.0)
Latin America & Caribbean	30.0% (19.6-43.4)	27.2% (20.6-34.9)	22.8% (18.4-27.7)	17.8% (14.7-21.2)	13.5% (9.6-18.1)
Oceania	14.6% (6.9-25.6)	15.6% (9.5-23.2)	17.3% (11.2-24.4)	18.9% (12.7-26.1)	21.4% (12.0-33.7)
South Asia	18.8% (9.3-32.4)	20.1% (13.6-28.2)	21.2% (16.1-27.0)	21.9% (17.9-26.5)	22.3% (16.3-29.5)
Sub-Saharan Africa	21.7% (13.3-32.3)	24.1% (18.2-31.1)	25.8% (21.1-30.8)	25.8% (21.9-30.2)	25.3% (19.9-31.4)
**45-49 years**					
World	32.0% (24.9-40.0)	30.2% (25.7-35.1)	27.6% (24.7-30.6)	25.2% (22.9-27.6)	22.5% (18.9-26.5)
Central & Eastern Europe	51.1% (37.4-65.4)	48.0% (40.2-56.2)	40.9% (35.4-47.1)	32.8% (27.3-38.6)	26.7% (19.1-35.0)
Central Asia & MENA	39.4% (25.6-55.1)	38.6% (30.0-48.3)	36.3% (30.6-42.6)	32.0% (27.8-36.3)	26.5% (20.5-33.0)
East & Southeast Asia	20.1% (11.4-31.4)	21.0% (15.8-26.7)	22.2% (18.4-26.4)	21.9% (18.3-25.7)	19.5% (13.5-27.1)
High-income Asia Pacific	34.3% (25.6-44.1)	26.3% (21.6-31.4)	19.7% (16.1-23.6)	14.0% (11.2-17.2)	9.4% (5.7-13.9)
High-income western	35.9% (27.4-45.3)	30.1% (25.4-35.2)	22.9% (19.9-26.0)	16.9% (14.5-19.4)	12.4% (9.0-16.1)
Latin America & Caribbean	40.3% (27.6-55.4)	37.0% (29.0-46.0)	31.7% (26.2-37.5)	25.2% (21.2-29.5)	19.4% (14.2-25.5)
Oceania	20.9% (10.1-35.1)	22.4% (14.1-32.2)	24.6% (16.3-33.8)	26.7% (18.5-35.8)	29.5% (17.5-44.0)
South Asia	26.1% (13.6-42.9)	28.0% (19.5-38.0)	29.5% (22.9-36.5)	30.3% (25.1-35.8)	30.7% (22.9-39.6)
Sub-Saharan Africa	30.0% (19.0-43.1)	33.2% (25.7-41.6)	35.3% (29.7-41.2)	35.4% (30.9-40.5)	34.6% (28.0-41.7)
**50-54 years**					
World	40.8% (33.0-49.3)	38.5% (33.6-43.7)	36.2% (33.0-39.5)	33.0% (30.4-35.6)	29.7% (25.4-34.2)
Central & Eastern Europe	60.6% (47.0-73.8)	57.8% (50.3-65.3)	50.7% (45.1-56.6)	42.2% (36.3-48.2)	35.0% (25.8-44.8)
Central Asia & MENA	48.9% (33.9-64.3)	48.3% (39.0-57.9)	46.1% (39.9-52.4)	41.2% (36.7-45.7)	35.0% (28.1-42.2)
East & Southeast Asia	27.4% (16.3-40.5)	28.5% (22.3-35.1)	30.4% (25.8-35.1)	29.4% (25.1-34.0)	26.8% (19.5-35.6)
High-income Asia Pacific	44.2% (34.4-54.6)	35.4% (29.8-41.2)	27.5% (23.0-32.1)	20.7% (17.1-24.6)	14.0% (9.2-19.8)
High-income western	45.5% (36.2-54.9)	38.8% (33.6-44.2)	31.4% (27.9-34.9)	23.6% (20.7-26.6)	17.7% (13.3-22.5)
Latin America & Caribbean	49.9% (36.2-64.7)	46.5% (37.7-55.6)	40.7% (34.7-46.9)	33.4% (28.8-38.2)	26.6% (20.1-33.9)
Oceania	28.2% (14.8-44.7)	30.1% (20.2-41.4)	32.7% (22.9-43.2)	35.3% (25.4-45.4)	38.0% (24.3-53.3)
South Asia	34.1% (19.3-52.5)	36.4% (26.7-47.5)	38.2% (30.9-45.7)	39.0% (33.1-45.0)	39.5% (30.3-49.2)
Sub-Saharan Africa	38.7% (25.9-52.9)	42.3% (34.1-51.0)	44.9% (38.9-51.1)	44.9% (40.2-50.2)	43.9% (36.7-51.1)
**55-59 years**					
World	47.6% (39.5-56.4)	47.3% (42.3-52.6)	45.0% (41.6-48.5)	40.8% (37.9-43.6)	37.3% (32.5-41.9)
Central & Eastern Europe	68.4% (55.4-80.1)	65.8% (58.6-73.1)	59.5% (53.5-65.5)	50.7% (44.7-56.7)	43.4% (33.7-53.4)
Central Asia & MENA	57.0% (41.9-72.0)	56.3% (47.0-65.8)	53.9% (47.6-60.3)	49.6% (44.8-54.3)	43.2% (35.8-50.5)

Prevalence of raised blood pressure					
Super-region (Women)	Year				
	1975	1985	1995	2005	2015
East & Southeast Asia	35.0% (22.2-49.2)	36.4% (29.6-43.6)	38.5% (33.5-43.6)	37.6% (32.8-42.5)	34.7% (26.5-44.0)
High-income Asia Pacific	53.0% (43.0-63.3)	44.3% (38.4-50.4)	35.7% (30.7-40.9)	28.5% (24.0-33.1)	20.2% (14.0-27.2)
High-income western	52.5% (43.1-61.6)	46.8% (41.4-52.4)	40.4% (36.6-44.3)	31.3% (27.8-34.8)	24.1% (18.6-29.9)
Latin America & Caribbean	57.8% (44.5-71.7)	54.8% (46.2-63.6)	49.1% (42.9-55.4)	41.6% (36.6-46.6)	34.2% (26.9-42.1)
Oceania	36.0% (20.7-53.2)	38.1% (27.0-49.7)	40.9% (30.3-52.0)	43.6% (33.1-53.7)	46.2% (31.7-61.1)
South Asia	41.9% (25.6-60.1)	44.4% (34.0-55.4)	46.2% (38.4-53.9)	47.2% (40.9-53.3)	47.5% (37.7-57.3)
Sub-Saharan Africa	46.9% (33.4-60.9)	50.6% (42.2-59.2)	53.2% (47.2-59.4)	53.3% (48.3-58.4)	52.1% (44.9-59.0)
**60-64 years**					
World	55.9% (48.4-63.9)	53.9% (49.2-58.8)	51.5% (48.2-54.8)	47.8% (44.9-50.7)	43.7% (38.8-48.4)
Central & Eastern Europe	73.1% (61.6-83.4)	70.7% (64.0-77.4)	65.2% (59.6-70.8)	57.4% (51.8-62.9)	50.6% (41.1-59.8)
Central Asia & MENA	62.8% (49.1-75.9)	62.4% (53.8-70.9)	60.3% (54.6-66.1)	56.4% (51.9-60.8)	50.0% (42.7-57.0)
East & Southeast Asia	42.3% (29.0-56.3)	43.7% (36.7-50.7)	45.7% (40.5-50.8)	45.1% (40.2-49.9)	41.6% (32.6-51.2)
High-income Asia Pacific	60.0% (50.7-69.5)	51.9% (46.0-57.9)	43.7% (38.4-49.0)	36.2% (31.1-41.3)	28.1% (20.5-36.4)
High-income western	60.3% (51.6-68.7)	53.8% (48.6-59.1)	47.5% (43.7-51.2)	39.1% (35.3-42.8)	31.2% (25.0-37.6)
Latin America & Caribbean	63.6% (51.5-76.0)	60.9% (53.2-69.0)	55.7% (49.8-61.3)	48.6% (43.6-53.3)	41.3% (33.7-49.1)
Oceania	43.0% (27.0-59.6)	45.3% (33.8-56.6)	48.0% (37.6-58.4)	50.6% (40.3-60.2)	53.1% (38.8-66.9)
South Asia	48.4% (32.2-65.6)	51.0% (40.8-61.3)	52.7% (45.1-60.1)	53.7% (47.4-59.7)	53.8% (44.2-63.2)
Sub-Saharan Africa	53.5% (40.4-66.5)	57.2% (49.3-65.1)	59.4% (53.7-65.3)	59.7% (54.9-64.7)	58.5% (51.6-65.0)
**65-69 years**					
World	60.9% (54.2-68.0)	57.9% (53.7-62.4)	57.0% (53.8-60.1)	53.6% (50.8-56.3)	48.9% (44.4-53.1)
Central & Eastern Europe	75.8% (65.8-84.9)	74.0% (67.8-80.3)	69.2% (63.9-74.5)	62.3% (56.8-67.9)	56.2% (47.6-64.3)
Central Asia & MENA	66.6% (54.2-78.1)	66.5% (58.9-73.8)	64.4% (59.1-69.8)	60.8% (56.5-65.1)	55.3% (48.5-61.9)
East & Southeast Asia	48.3% (35.7-61.1)	49.6% (42.9-56.2)	51.6% (46.4-56.4)	50.9% (46.2-55.6)	47.8% (39.2-56.5)
High-income Asia Pacific	64.9% (56.5-73.5)	57.8% (52.4-63.6)	50.4% (45.4-55.5)	43.3% (38.3-48.2)	36.0% (27.7-44.4)
High-income western	64.8% (56.9-72.5)	57.5% (52.7-62.5)	53.0% (49.3-56.5)	46.3% (42.7-49.7)	38.1% (32.0-44.2)
Latin America & Caribbean	67.4% (56.8-78.3)	65.1% (58.0-72.4)	60.5% (55.1-65.8)	54.2% (49.5-58.6)	47.3% (40.1-54.6)
Oceania	48.8% (33.4-64.2)	51.2% (40.0-61.8)	53.6% (43.5-63.1)	55.9% (46.2-64.8)	58.0% (45.0-70.8)
South Asia	53.3% (38.2-68.6)	55.7% (46.3-65.2)	57.3% (50.0-64.3)	58.1% (51.9-63.8)	58.3% (49.4-66.8)
Sub-Saharan Africa	58.4% (46.5-69.9)	61.8% (54.7-69.0)	63.8% (58.4-69.1)	64.0% (59.6-68.6)	63.0% (56.6-69.0)
**70-74 years**					
World	64.4% (58.2-70.9)	62.7% (58.4-67.1)	60.4% (57.1-63.7)	57.2% (54.2-60.2)	53.2% (49.1-57.2)
Central & Eastern Europe	77.1% (67.8-85.9)	75.6% (68.8-82.2)	71.2% (65.6-76.9)	65.5% (60.2-71.1)	60.2% (52.6-67.7)
Central Asia & MENA	68.9% (58.0-79.5)	68.7% (61.7-75.7)	67.0% (61.8-72.4)	63.8% (59.4-68.2)	58.9% (52.4-65.1)
East & Southeast Asia	52.8% (41.2-64.5)	54.2% (47.4-60.6)	55.8% (50.6-60.9)	55.3% (50.5-60.0)	52.6% (44.7-60.7)
High-income Asia Pacific	68.5% (60.8-76.3)	62.3% (57.0-68.0)	55.8% (50.9-60.8)	49.5% (44.6-54.6)	42.5% (34.2-50.8)
High-income western	67.9% (60.7-75.4)	62.7% (58.0-67.6)	57.5% (54.0-61.1)	51.5% (48.0-54.8)	44.3% (38.2-50.1)
Latin America & Caribbean	69.6% (59.9-79.9)	67.6% (61.0-74.8)	63.5% (58.3-68.8)	58.0% (53.5-62.5)	51.9% (45.2-58.5)
Oceania	53.5% (38.8-67.5)	55.5% (44.9-65.5)	57.7% (48.1-66.7)	59.7% (50.8-68.0)	61.4% (49.7-72.9)
South Asia	56.6% (42.8-70.4)	58.7% (49.8-67.8)	60.2% (53.1-67.2)	60.8% (54.3-67.0)	61.1% (52.5-69.3)
Sub-Saharan Africa	61.8% (51.1-72.3)	64.8% (58.0-71.6)	66.5% (61.1-71.9)	66.6% (61.9-71.6)	65.9% (59.7-72.1)
**75-79 years**					
World	66.6% (60.8-72.9)	65.0% (60.8-69.5)	62.1% (58.7-65.5)	59.7% (56.6-62.8)	56.2% (52.1-60.3)
Central & Eastern Europe	77.5% (68.3-86.3)	76.3% (68.9-83.2)	72.7% (66.3-78.9)	67.5% (61.5-73.1)	62.8% (55.1-70.3)
Central Asia & MENA	70.1% (60.1-80.2)	69.9% (63.1-76.9)	68.5% (63.1-74.2)	65.6% (61.0-70.3)	61.3% (55.2-67.1)
East & Southeast Asia	56.1% (44.8-66.7)	57.2% (50.2-63.6)	58.6% (53.0-64.1)	58.1% (52.8-63.2)	55.7% (48.1-63.5)
High-income Asia Pacific	70.8% (63.5-78.4)	65.6% (60.0-71.3)	59.9% (54.7-65.2)	54.4% (49.0-59.7)	47.8% (39.0-55.9)

Prevalence of raised blood pressure					
Super-region (Women)	Year				
	1975	1985	1995	2005	2015
High-income western	69.3% (62.6-76.7)	65.2% (60.3-70.2)	59.4% (55.7-63.0)	55.1% (51.4-58.5)	49.3% (42.9-55.0)
Latin America & Caribbean	70.9% (61.9-80.6)	69.0% (62.4-76.0)	65.5% (60.1-70.9)	60.6% (55.8-65.1)	55.2% (48.4-61.7)
Oceania	56.8% (42.9-69.5)	58.6% (47.6-68.4)	60.4% (50.7-69.3)	62.1% (53.0-70.6)	63.6% (52.3-74.6)
South Asia	58.4% (45.1-71.3)	60.2% (51.2-69.5)	61.5% (54.0-69.0)	62.1% (54.9-68.9)	62.3% (53.5-70.8)
Sub-Saharan Africa	64.0% (54.4-73.6)	66.6% (60.1-73.2)	68.1% (62.6-73.6)	68.1% (63.1-73.2)	67.5% (61.5-73.7)
**80-84 years**					
World	68.0% (62.5-74.1)	66.2% (62.0-70.8)	64.2% (60.8-67.7)	60.9% (57.7-63.9)	57.3% (53.0-61.3)
Central & Eastern Europe	77.9% (68.4-86.8)	76.7% (68.6-84.0)	73.4% (66.0-80.0)	68.6% (62.4-74.5)	64.5% (57.2-71.6)
Central Asia & MENA	70.6% (61.5-80.5)	70.3% (63.4-77.5)	69.1% (63.4-75.1)	66.3% (61.2-71.3)	62.2% (56.0-68.1)
East & Southeast Asia	57.8% (46.3-68.3)	58.7% (50.8-65.8)	60.1% (53.5-66.3)	59.7% (53.4-65.5)	57.3% (48.9-65.2)
High-income Asia Pacific	72.5% (65.0-80.2)	67.9% (61.7-74.4)	62.9% (56.9-69.0)	57.9% (51.5-64.1)	51.9% (42.3-60.8)
High-income western	70.0% (63.2-77.5)	66.6% (61.6-71.9)	62.5% (58.7-66.5)	57.6% (53.7-61.3)	52.2% (45.9-57.9)
Latin America & Caribbean	71.4% (62.5-80.9)	69.6% (62.7-76.6)	66.3% (60.5-71.9)	61.9% (56.5-66.7)	56.9% (49.8-63.4)
Oceania	58.9% (45.2-71.3)	60.3% (48.6-70.5)	61.9% (51.1-71.7)	63.4% (53.2-72.8)	64.8% (53.1-76.0)
South Asia	58.9% (44.9-71.7)	60.5% (50.3-70.3)	61.6% (52.7-69.9)	62.2% (53.6-70.0)	62.2% (52.3-71.4)
Sub-Saharan Africa	65.2% (56.2-74.2)	67.4% (61.0-73.9)	68.8% (63.0-74.4)	68.8% (63.7-74.1)	68.2% (62.4-74.4)
**85+ years**					
World	68.8% (58.9-78.7)	66.2% (58.0-74.4)	64.1% (57.2-70.9)	60.4% (54.6-66.0)	56.9% (50.4-63.0)
Central & Eastern Europe	78.1% (62.3-91.5)	76.9% (62.2-89.3)	73.8% (60.6-85.5)	69.3% (58.0-79.9)	65.1% (54.4-75.8)
Central Asia & MENA	70.6% (57.3-84.6)	70.1% (58.0-81.8)	68.8% (57.6-79.5)	66.3% (56.3-75.9)	62.1% (52.8-71.4)
East & Southeast Asia	58.0% (44.1-70.7)	58.7% (47.2-69.3)	60.0% (49.1-69.9)	59.6% (48.7-69.2)	57.5% (46.0-68.1)
High-income Asia Pacific	74.3% (61.9-85.7)	69.9% (59.3-79.8)	65.2% (55.4-74.7)	60.5% (50.0-70.7)	54.8% (40.7-67.4)
High-income western	70.2% (58.8-81.8)	66.2% (57.6-75.0)	62.6% (56.1-69.4)	57.7% (52.0-63.1)	53.0% (44.1-60.7)
Latin America & Caribbean	71.2% (57.9-84.7)	69.5% (57.6-81.2)	66.3% (56.1-76.1)	62.3% (53.2-70.9)	57.4% (47.1-66.9)
Oceania	58.9% (40.5-75.8)	60.5% (45.0-74.5)	62.2% (47.1-76.1)	63.6% (49.2-76.9)	64.9% (49.7-79.6)
South Asia	57.4% (39.2-74.8)	58.7% (42.9-73.5)	59.9% (44.4-73.4)	60.5% (45.1-73.6)	60.5% (44.3-74.7)
Sub-Saharan Africa	65.8% (54.2-78.0)	67.5% (57.0-78.0)	68.8% (58.2-79.1)	68.9% (58.4-79.0)	67.9% (57.2-78.2)

Appendix Figure 1: Flowchart of secondary search for data sources.

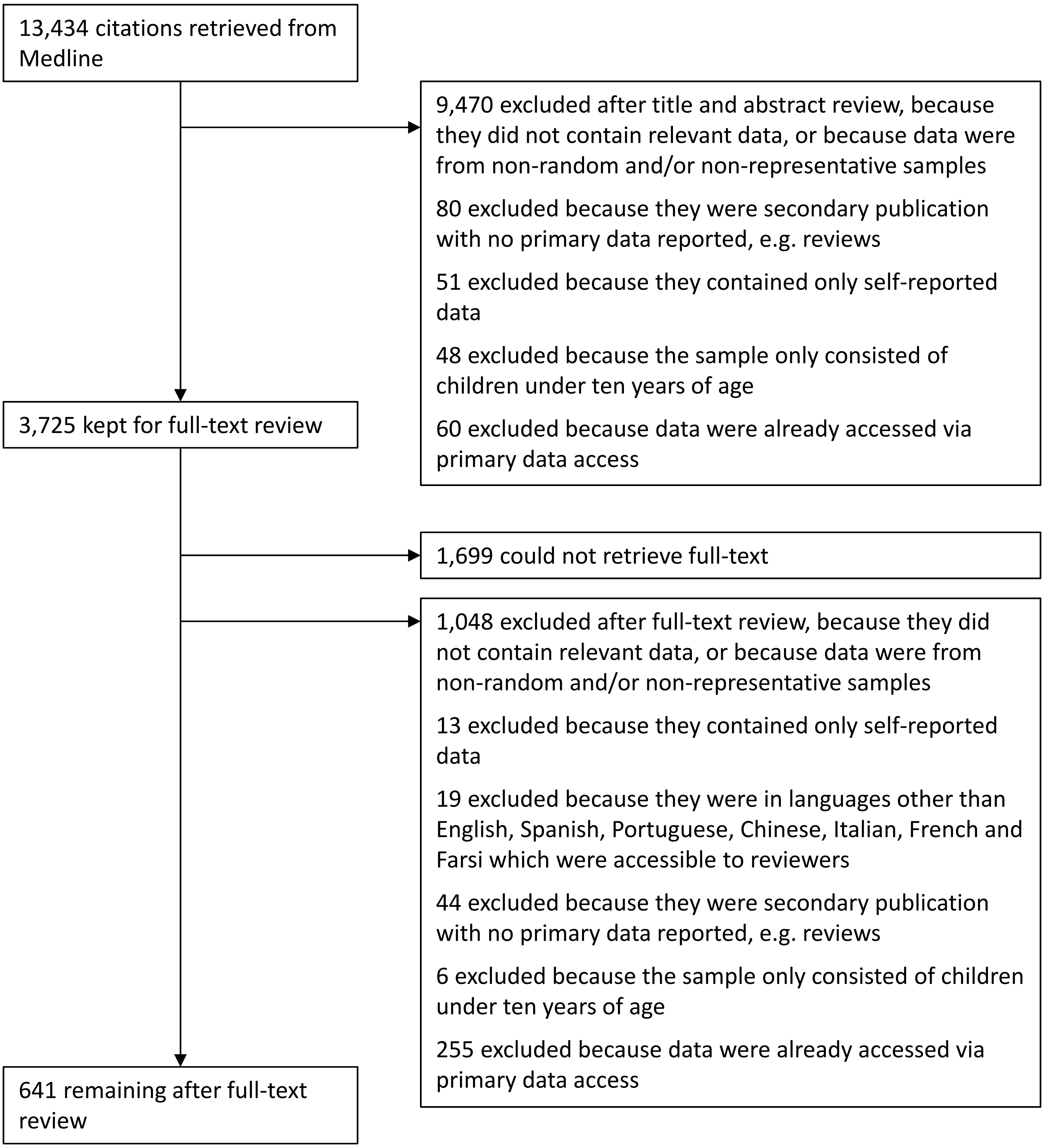
13,434 citations retrieved from Medline
9,470 excluded after title and abstract review, because they did not contain relevant data, or because data were from non-random and/or non-representative samples
80 excluded because they were secondary publication with no primary data reported, e.g. reviews
51 excluded because they contained only self-reported data
48 excluded because the sample only consisted of children under ten years of age
60 excluded because data were already accessed via primary data access
3,725 kept for full-text review
1,699 could not retrieve full-text
1,048 excluded after full-text review, because they did not contain relevant data, or because data were from non-random and/or non-representative samples
13 excluded because they contained only self-reported data
19 excluded because they were in languages other than English, Spanish, Portuguese, Chinese, Italian, French and Farsi which were accessible to reviewers
44 excluded because they were secondary publication with no primary data reported, e.g. reviews
6 excluded because the sample only consisted of children under ten years of age
255 excluded because data were already accessed via primary data access
641 remaining after full-text review

Appendix Figure 2: Number of data sources by country.

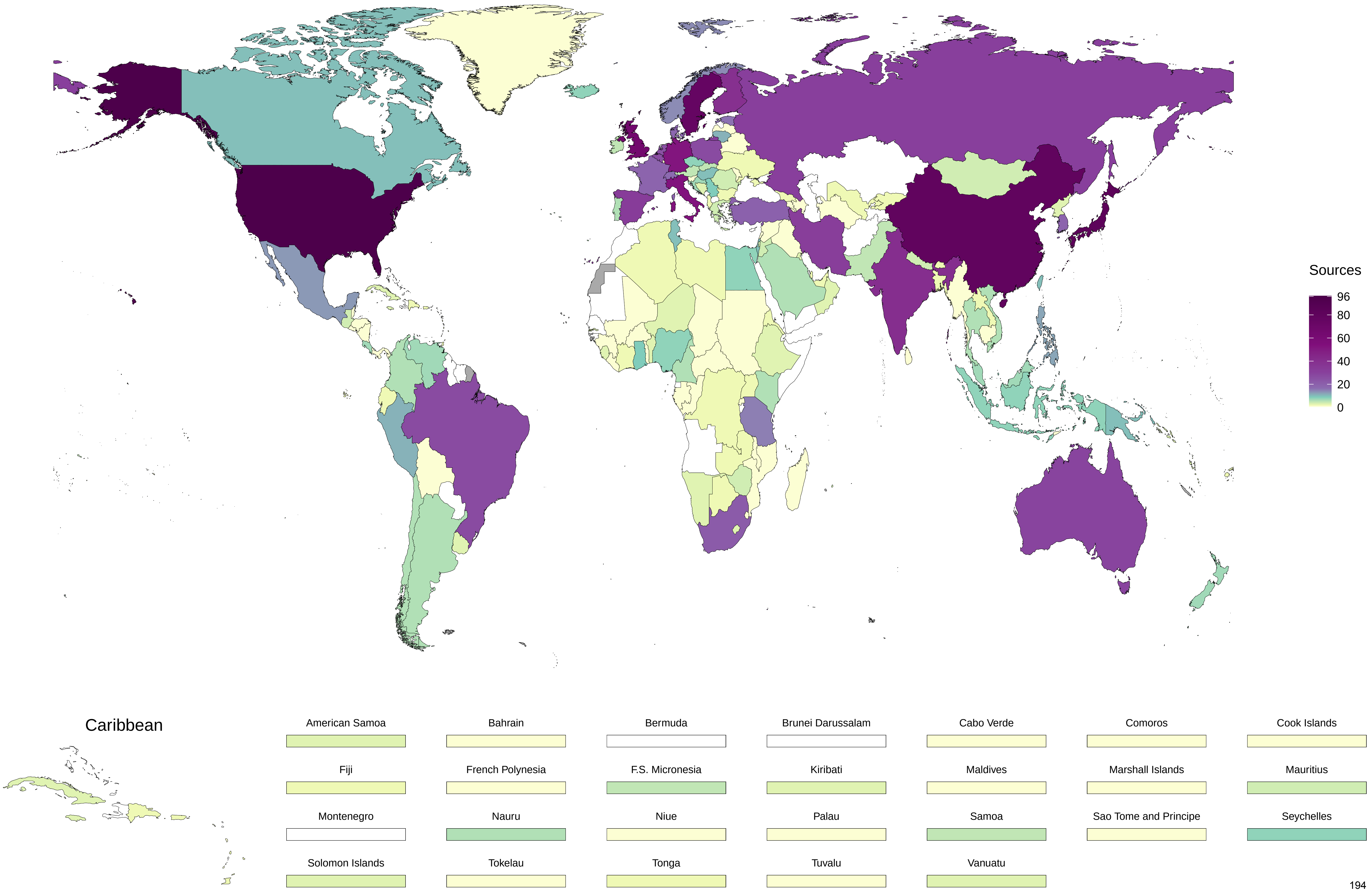
Sources
96
80
60
40
20
0
Caribbean
American Samoa
Bahrain
Bermuda
Brunei Darussalam
Cabo Verde
Comoros
Cook Islands
Fiji
French Polynesia
F.S. Micronesia
Kiribati
Maldives
Marshall Islands
Mauritius
Montenegro
Nauru
Niue
Palau
Samoa
Sao Tome and Principe
Seychelles
Solomon Islands
Tokelau
Tonga
Tuvalu
Vanuatu

Appendix Figure 3: Number of data sources by region and year.

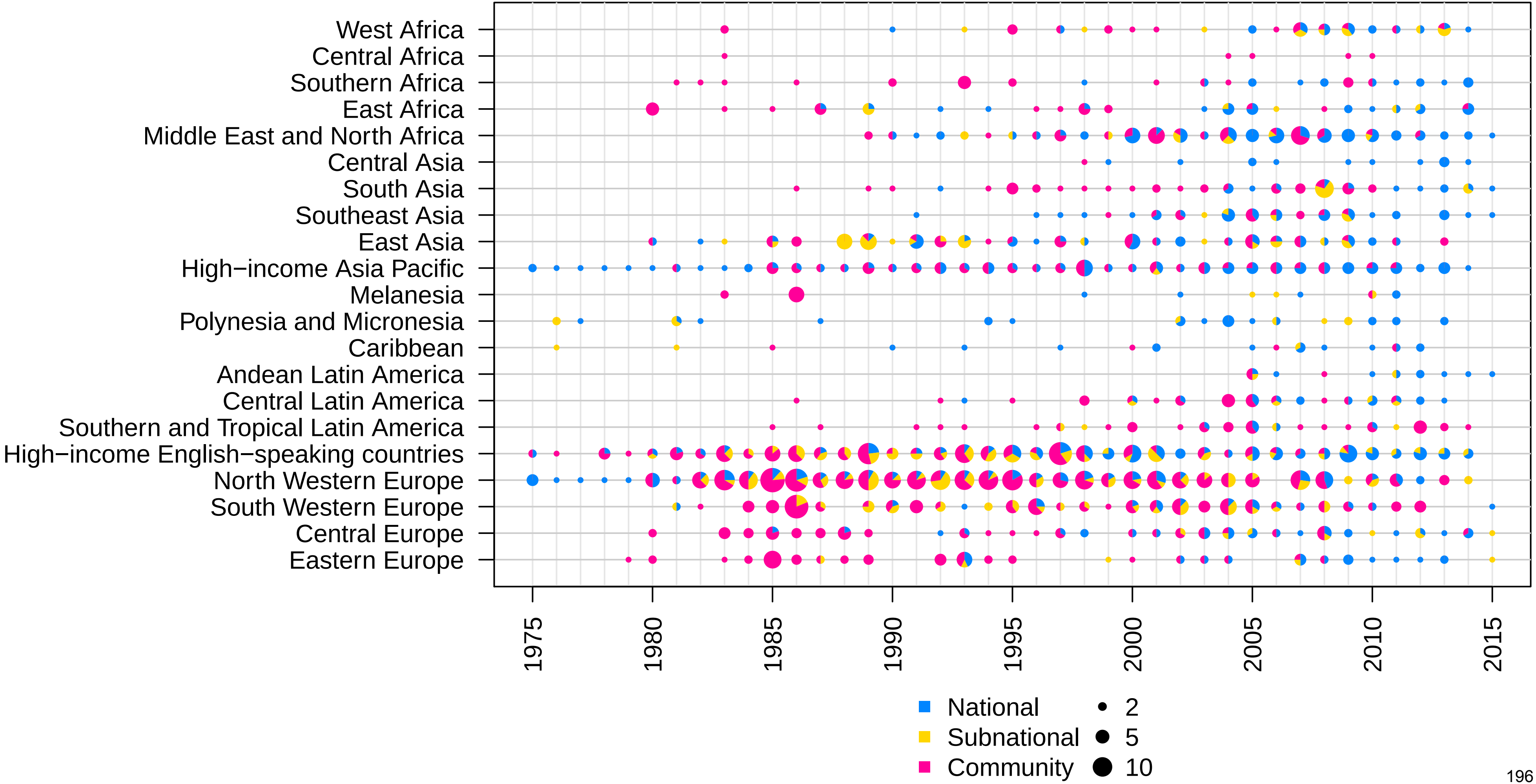
West Africa
Central Africa
Southern Africa
East Africa
Middle East and North Africa
Central Asia
South Asia
Southeast Asia
East Asia
High−income Asia Pacific
Melanesia
Polynesia and Micronesia
Caribbean
Andean Latin America
Central Latin America
Southern and Tropical Latin America
High−income English−speaking countries
North Western Europe
South Western Europe
Central Europe
Eastern Europe
1975
1980
1985
1990
1995
2000
2005
2010
2015
National
Subnational
Community
2
5
10

Appendix Figure 4: Comparison of age-standardised mean systolic blood pressure, mean diastolic blood pressure and prevalence of raised blood pressure in the main analysis, which used data from 1975 to 2015, with a sensitivity analysis using data from 1990 to 2015. Each point shows the age-standardised estimate for one country and sex.

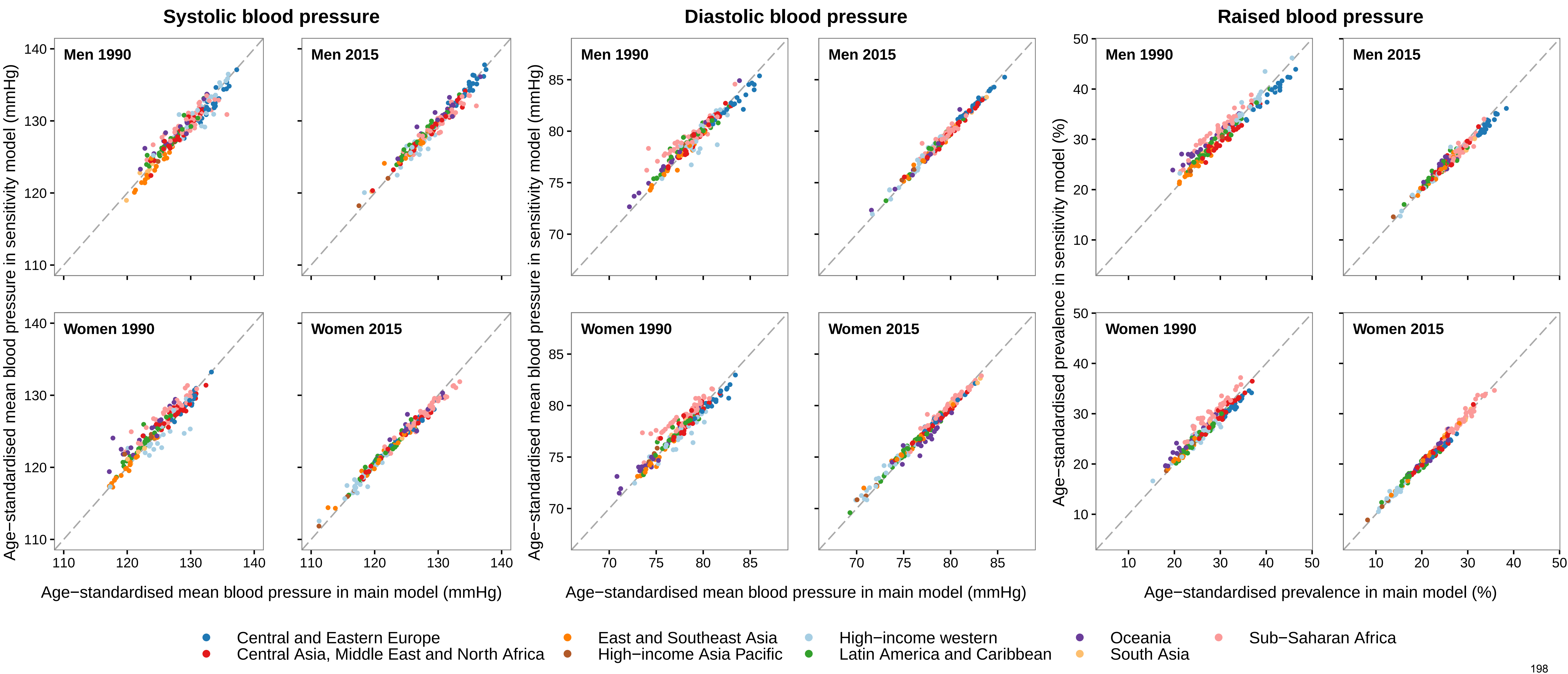

Systolic blood pressure
Diastolic blood pressure
Raised blood pressure
Men 1990
Men 2015
Women 1990
Women 2015
Age−standardised mean blood pressure in sensitivity model (mmHg)
Age−standardised prevalence in sensitivity model (%)
Age−standardised mean blood pressure in main model (mmHg)
Age−standardised prevalence in main model (%)
110
120
130
140
70
75
80
85
10
20
30
40
50
Central and Eastern Europe
Central Asia, Middle East and North Africa
East and Southeast Asia
High−income Asia Pacific
High−income western
Latin America and Caribbean
Oceania
South Asia
Sub−Saharan Africa

Appendix Figure 5: Trends in age-standardised mean systolic and diastolic blood pressure by sex and country in people aged 18 years and older. The lines show the posterior mean and the shaded area shows the 95% credible interval.

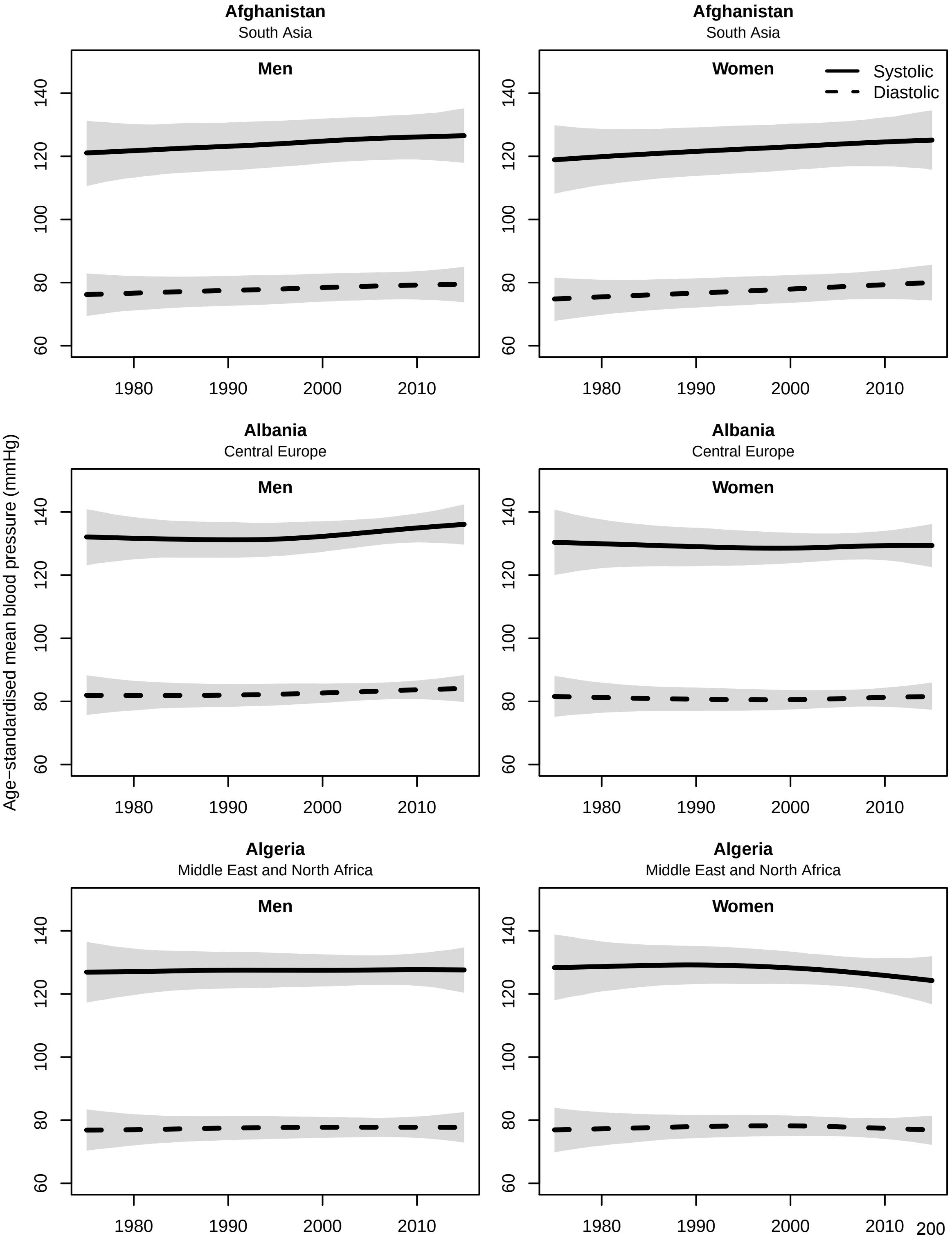
Afghanistan
South Asia
Men
Afghanistan
South Asia
Women
Systolic
Diastolic
Albania
Central Europe
Men
Albania
Central Europe
Women
Algeria
Middle East and North Africa
Men
Algeria
Middle East and North Africa
Women
Age−standardised mean blood pressure (mmHg)
60
80
100
120
140
1980
1990
2000
2010

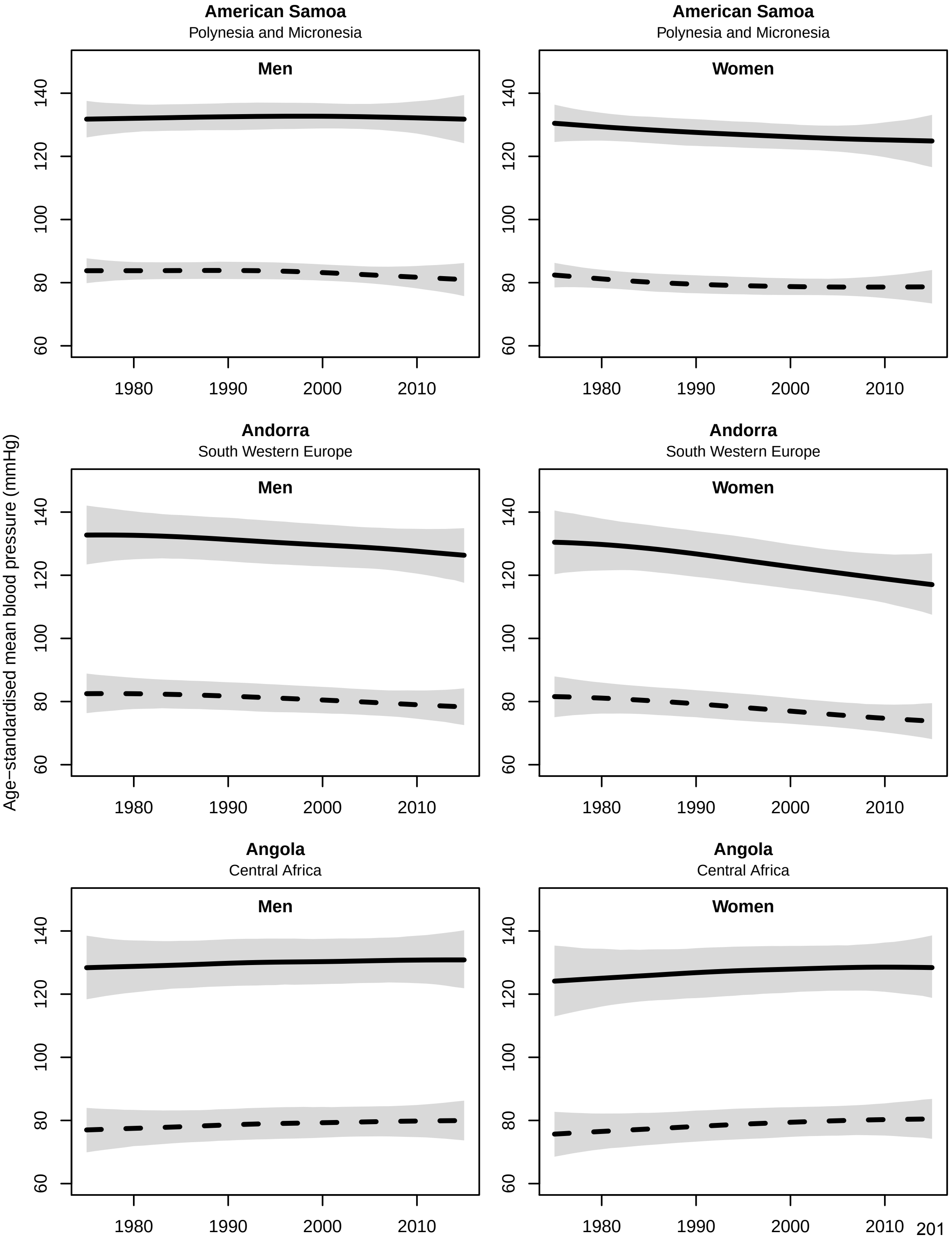
American Samoa
Polynesia and Micronesia
Men
Women
Andorra
South Western Europe
Angola
Central Africa
Age−standardised mean blood pressure (mmHg)
60
80
100
120
140
1980
1990
2000
2010

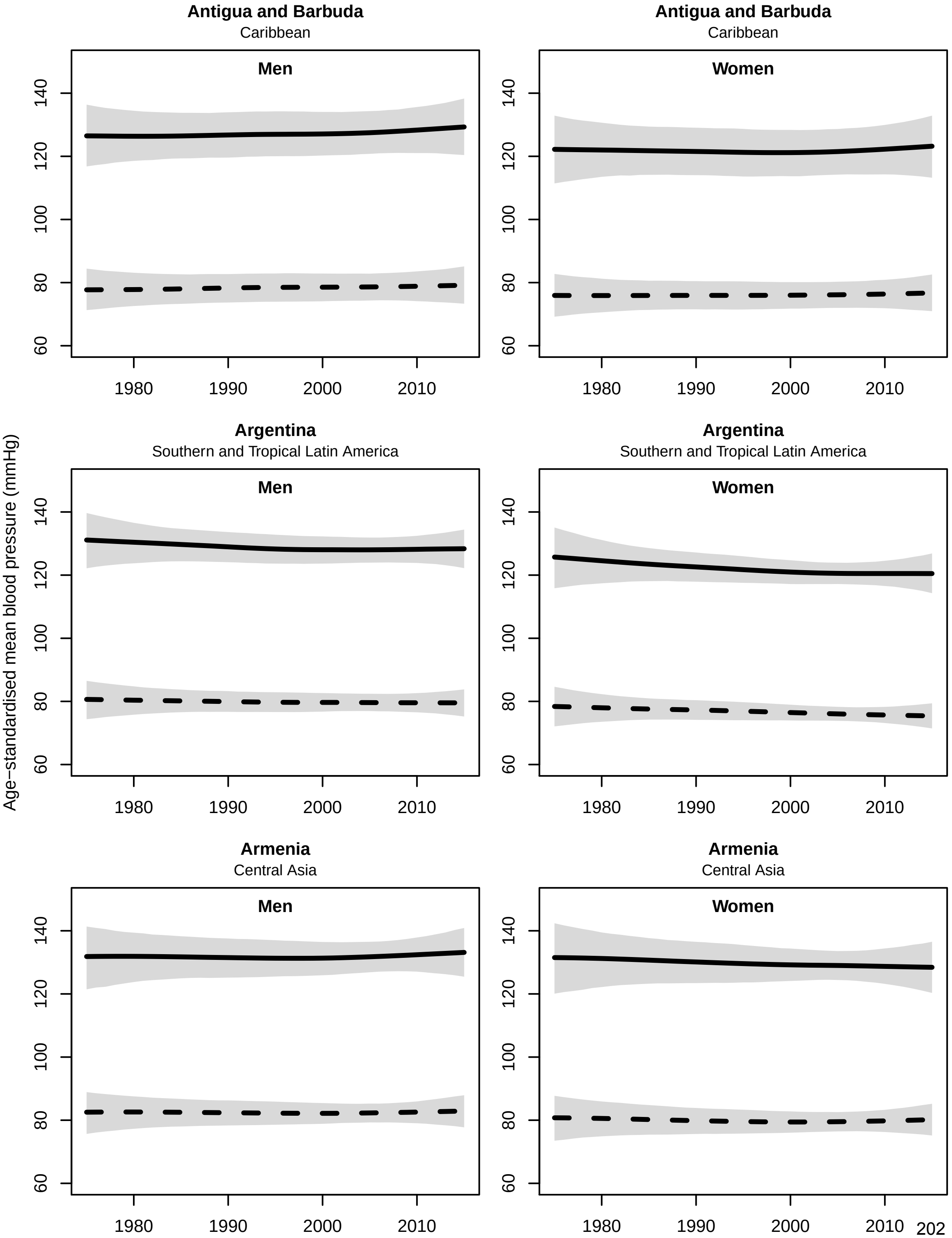
Antigua and Barbuda
Caribbean
Men
Antigua and Barbuda
Caribbean
Women
Argentina
Southern and Tropical Latin America
Men
Argentina
Southern and Tropical Latin America
Women
Armenia
Central Asia
Men
Armenia
Central Asia
Women
Age−standardised mean blood pressure (mmHg)
60
80
100
120
140
1980
1990
2000
2010

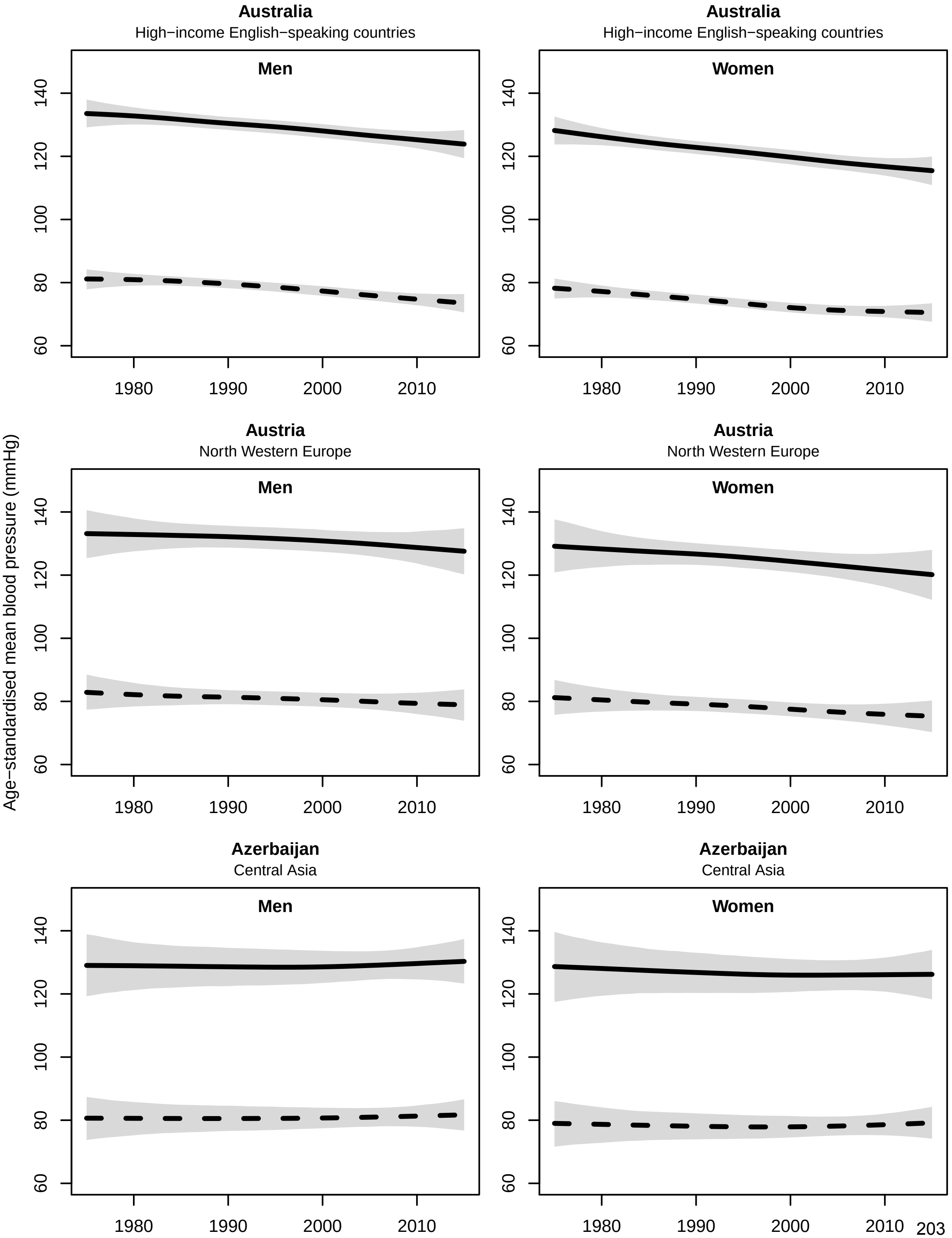
Australia
High−income English−speaking countries
Men
Women
Austria
North Western Europe
Men
Women
Azerbaijan
Central Asia
Men
Women
Age−standardised mean blood pressure (mmHg)
60
80
100
120
140
1980
1990
2000
2010

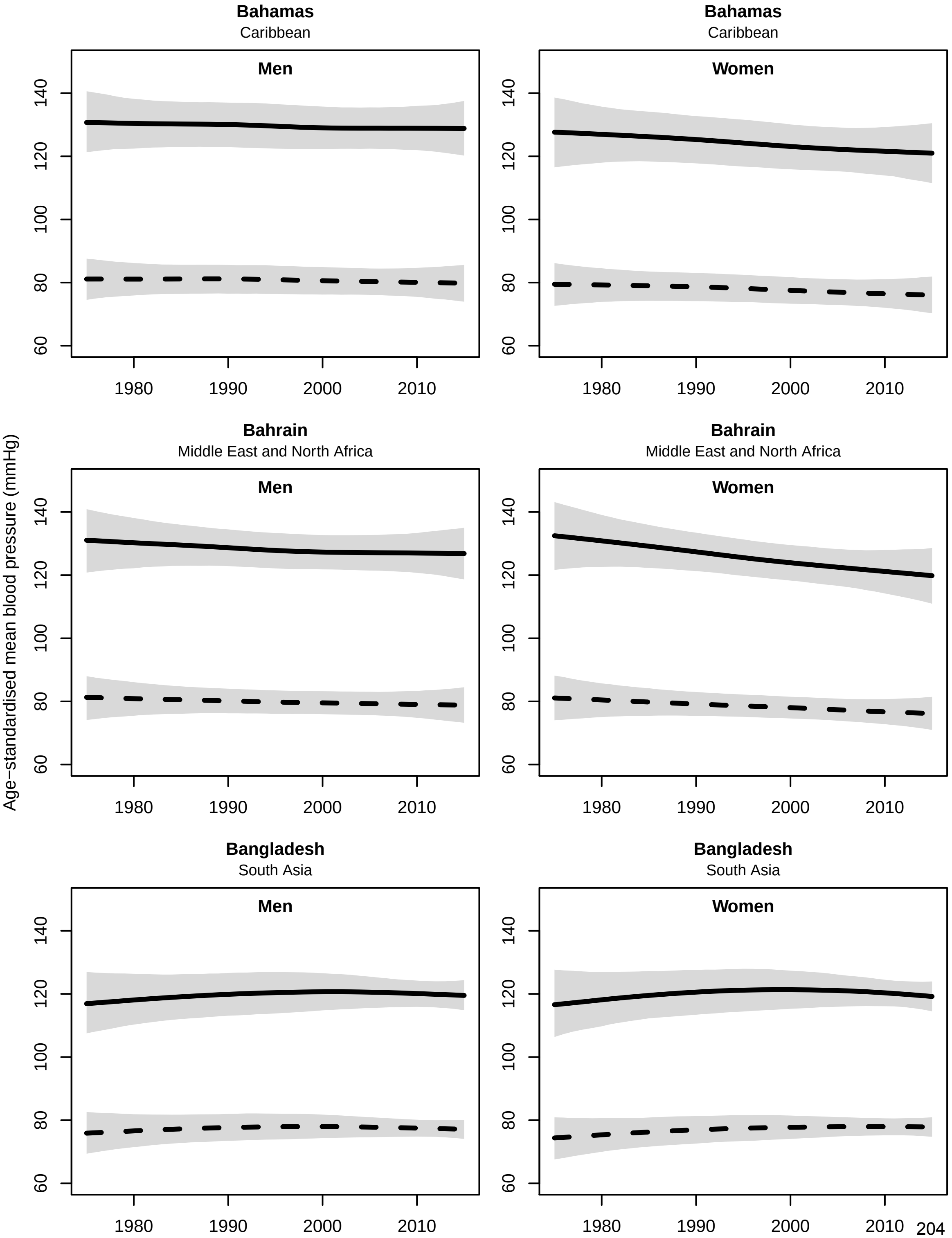
Bahamas
Caribbean
Men
Bahamas
Caribbean
Women
Bahrain
Middle East and North Africa
Men
Bahrain
Middle East and North Africa
Women
Bangladesh
South Asia
Men
Bangladesh
South Asia
Women
Age−standardised mean blood pressure (mmHg)
60
80
100
120
140
1980
1990
2000
2010

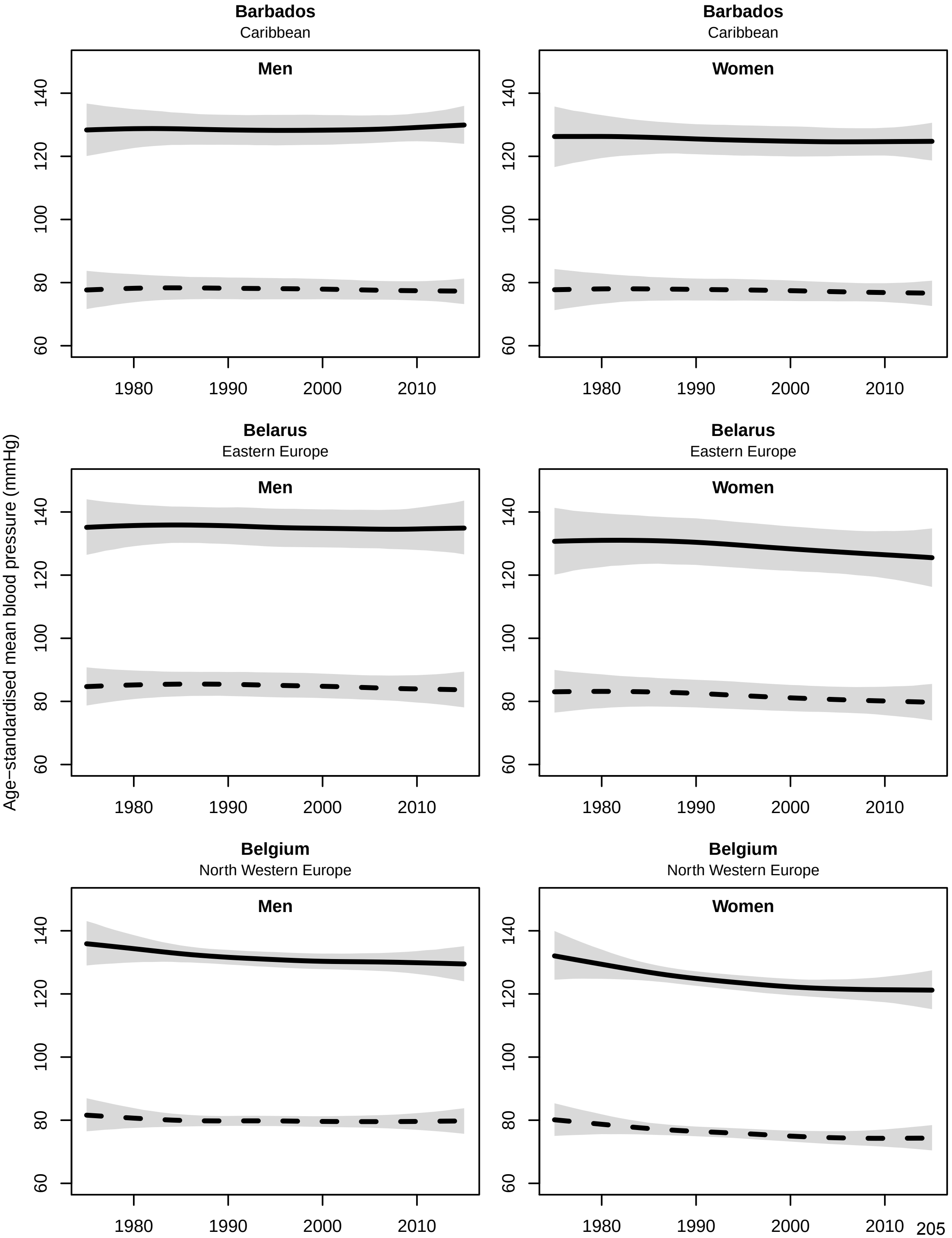
Barbados
Caribbean
Men
Women
Belarus
Eastern Europe
Men
Women
Belgium
North Western Europe
Men
Women
Age−standardised mean blood pressure (mmHg)
60
80
100
120
140
1980
1990
2000
2010

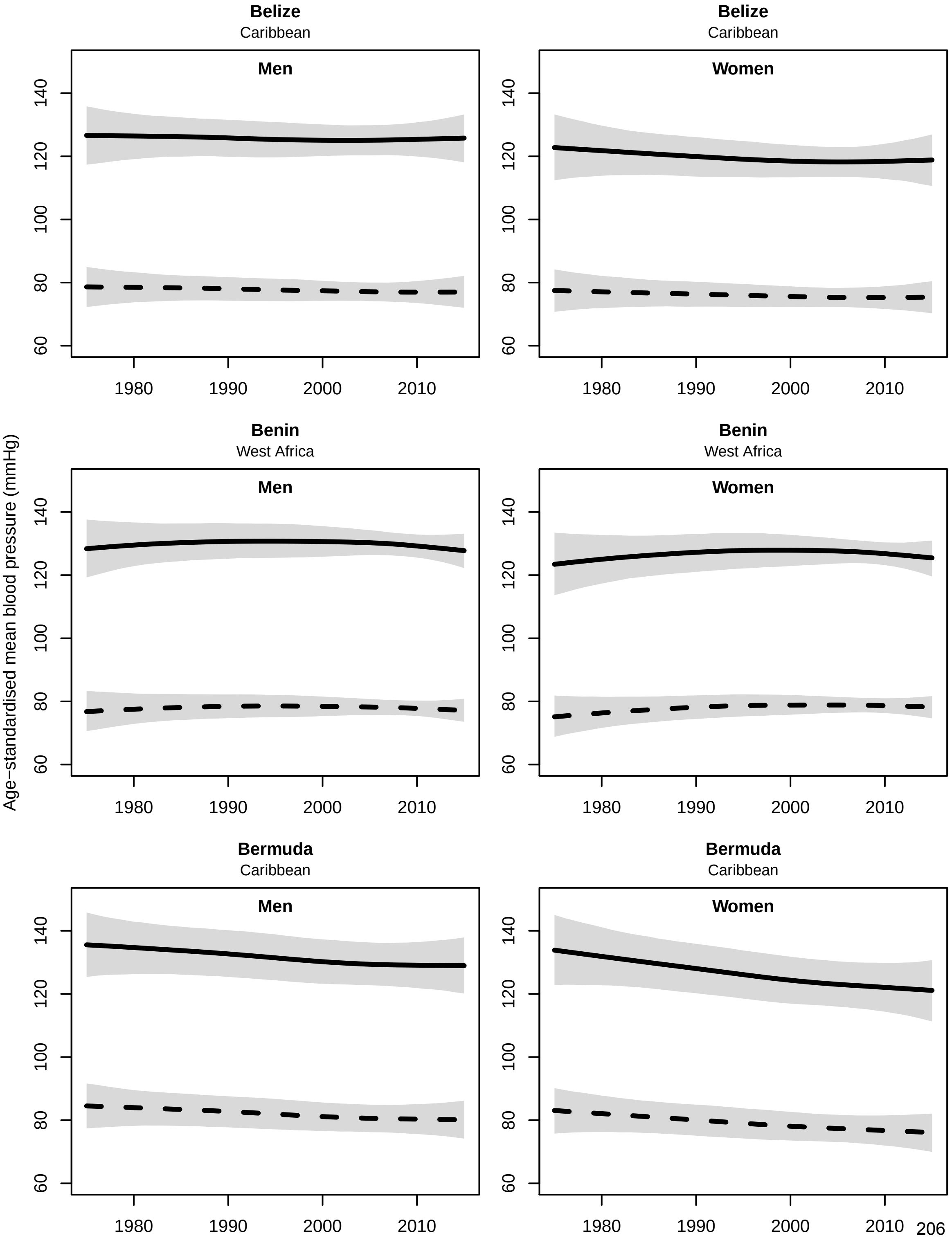
Belize
Caribbean
Men
Women
Benin
West Africa
Men
Women
Bermuda
Caribbean
Men
Women
Age−standardised mean blood pressure (mmHg)
60
80
100
120
140
1980
1990
2000
2010

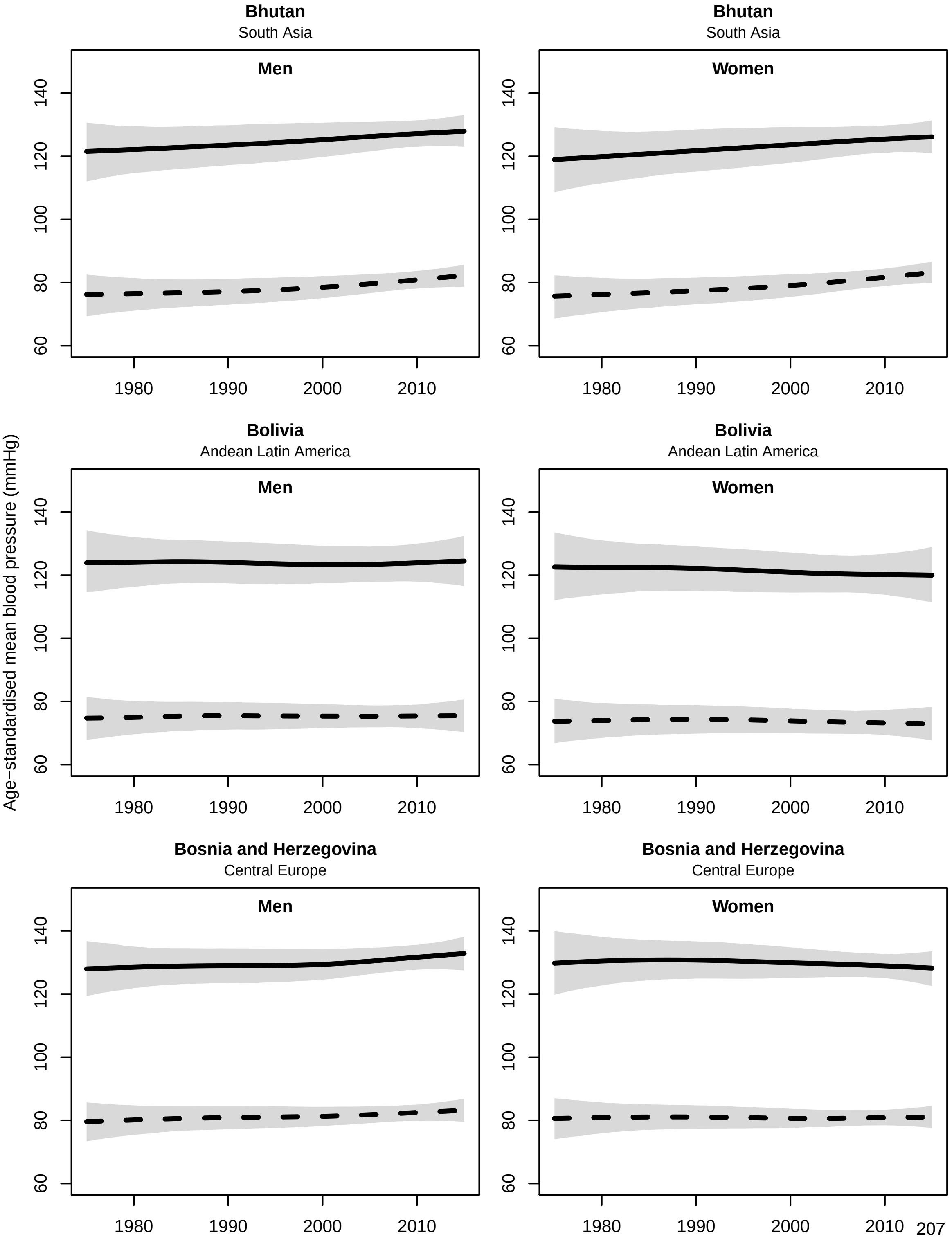
Bhutan
South Asia
Men
Bhutan
South Asia
Women
Bolivia
Andean Latin America
Men
Bolivia
Andean Latin America
Women
Bosnia and Herzegovina
Central Europe
Men
Bosnia and Herzegovina
Central Europe
Women
Age−standardised mean blood pressure (mmHg)
60
80
100
120
140
1980
1990
2000
2010

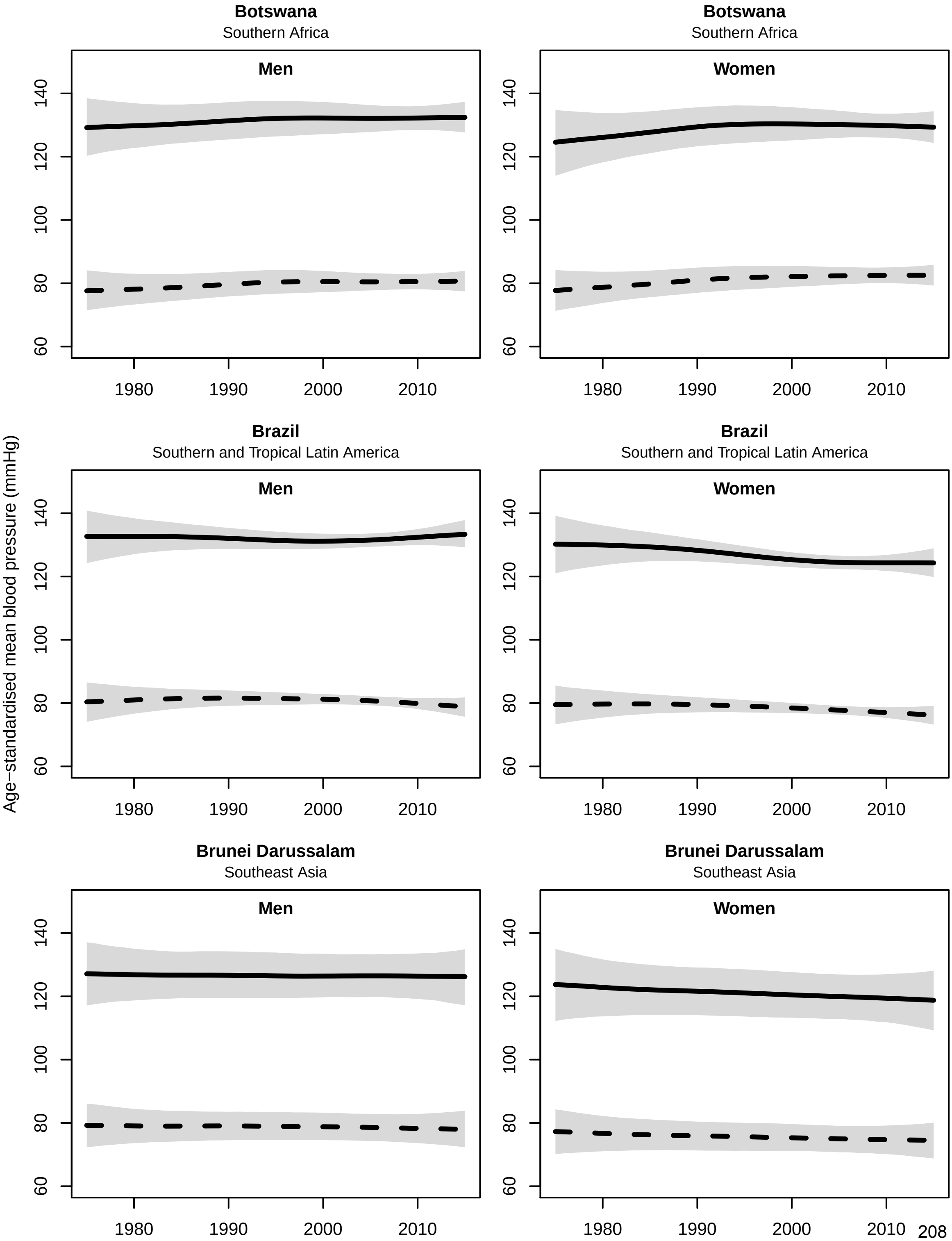
Botswana
Southern Africa
Men
Women
Brazil
Southern and Tropical Latin America
Brunei Darussalam
Southeast Asia
Age−standardised mean blood pressure (mmHg)
60
80
100
120
140
1980
1990
2000
2010

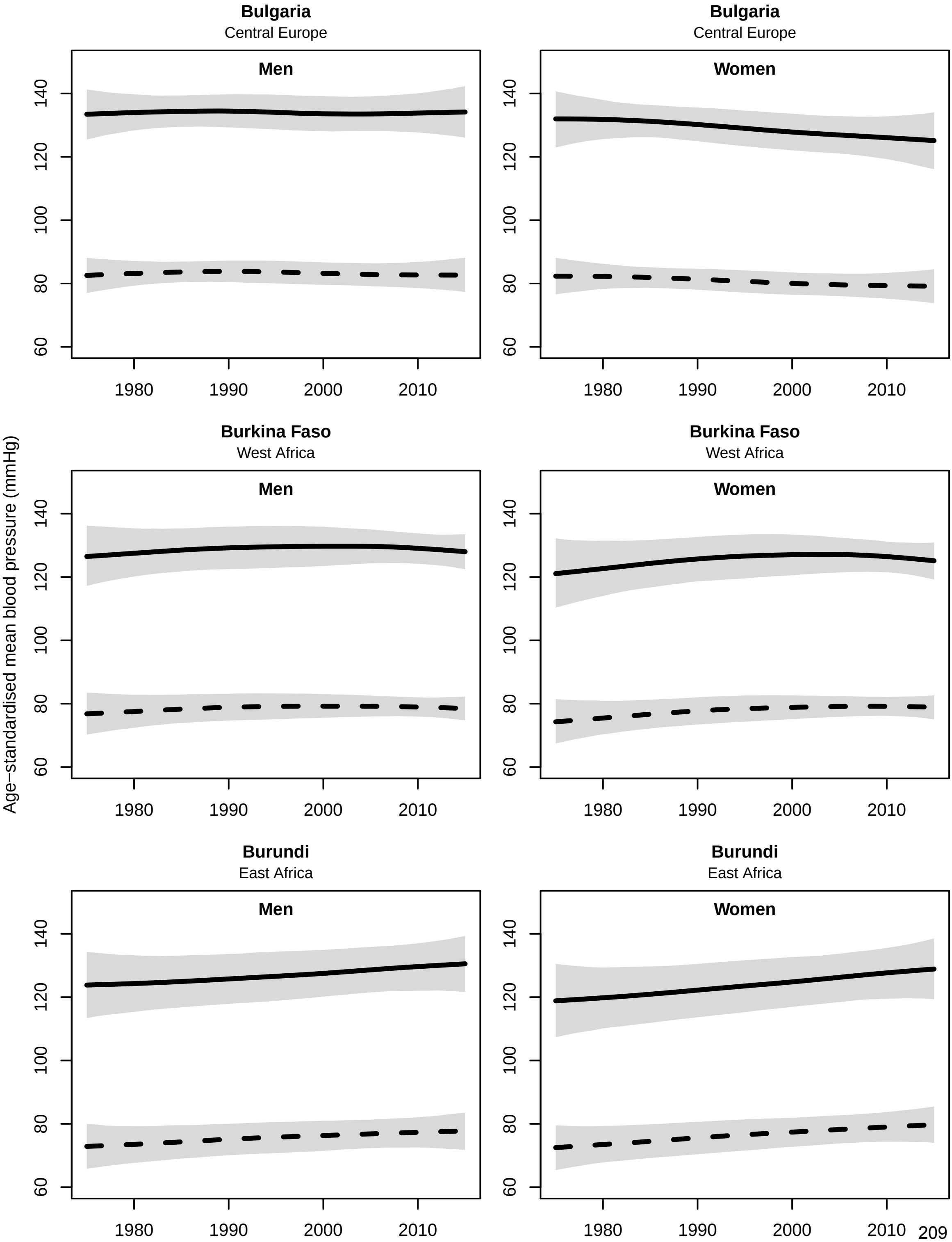
Bulgaria
Central Europe
Men
Bulgaria
Central Europe
Women
Burkina Faso
West Africa
Men
Burkina Faso
West Africa
Women
Burundi
East Africa
Men
Burundi
East Africa
Women
Age−standardised mean blood pressure (mmHg)
60
80
100
120
140
1980
1990
2000
2010

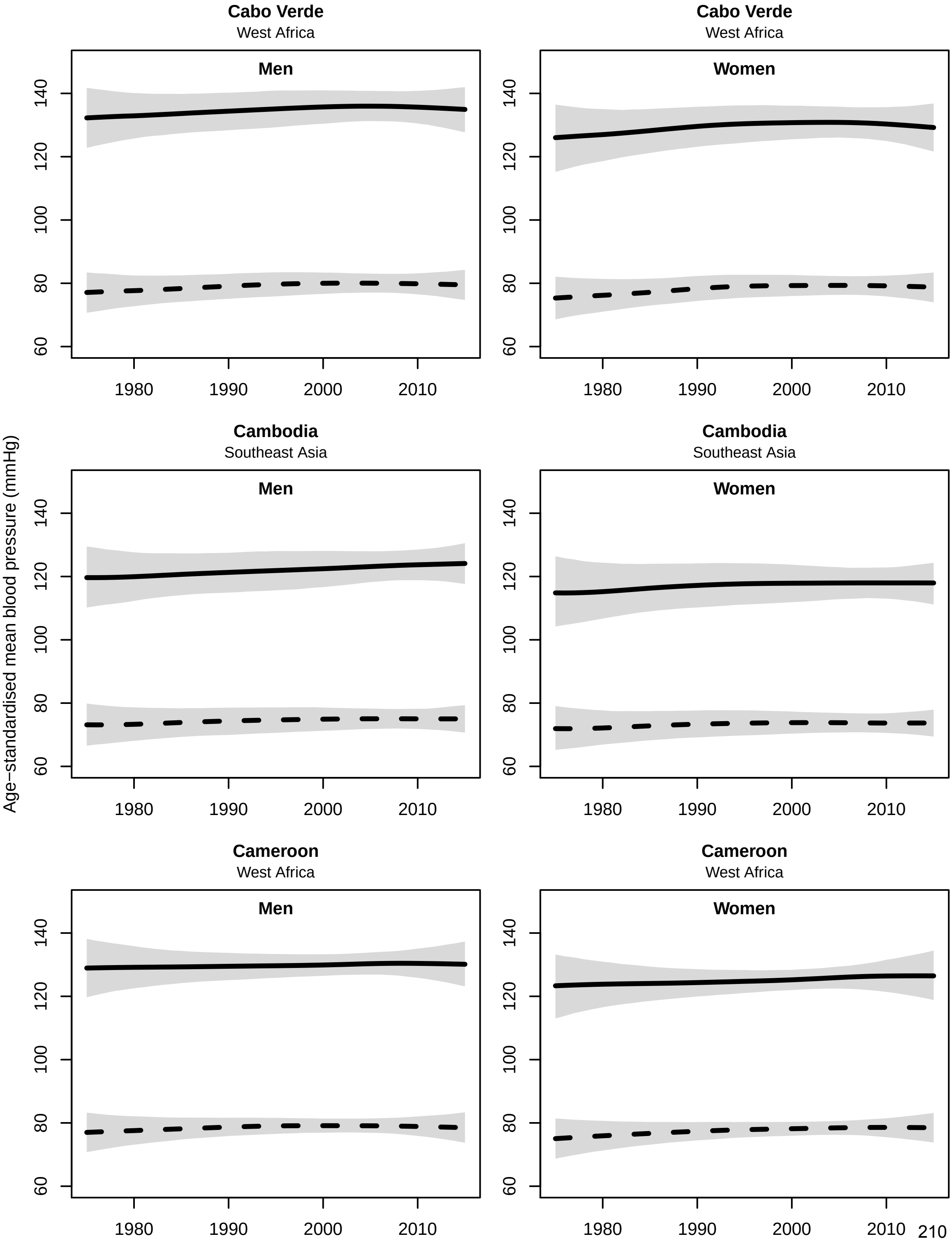
Cabo Verde
West Africa
Men
Cabo Verde
West Africa
Women
Cambodia
Southeast Asia
Men
Cambodia
Southeast Asia
Women
Cameroon
West Africa
Men
Cameroon
West Africa
Women
Age−standardised mean blood pressure (mmHg)
60
80
100
120
140
1980
1990
2000
2010

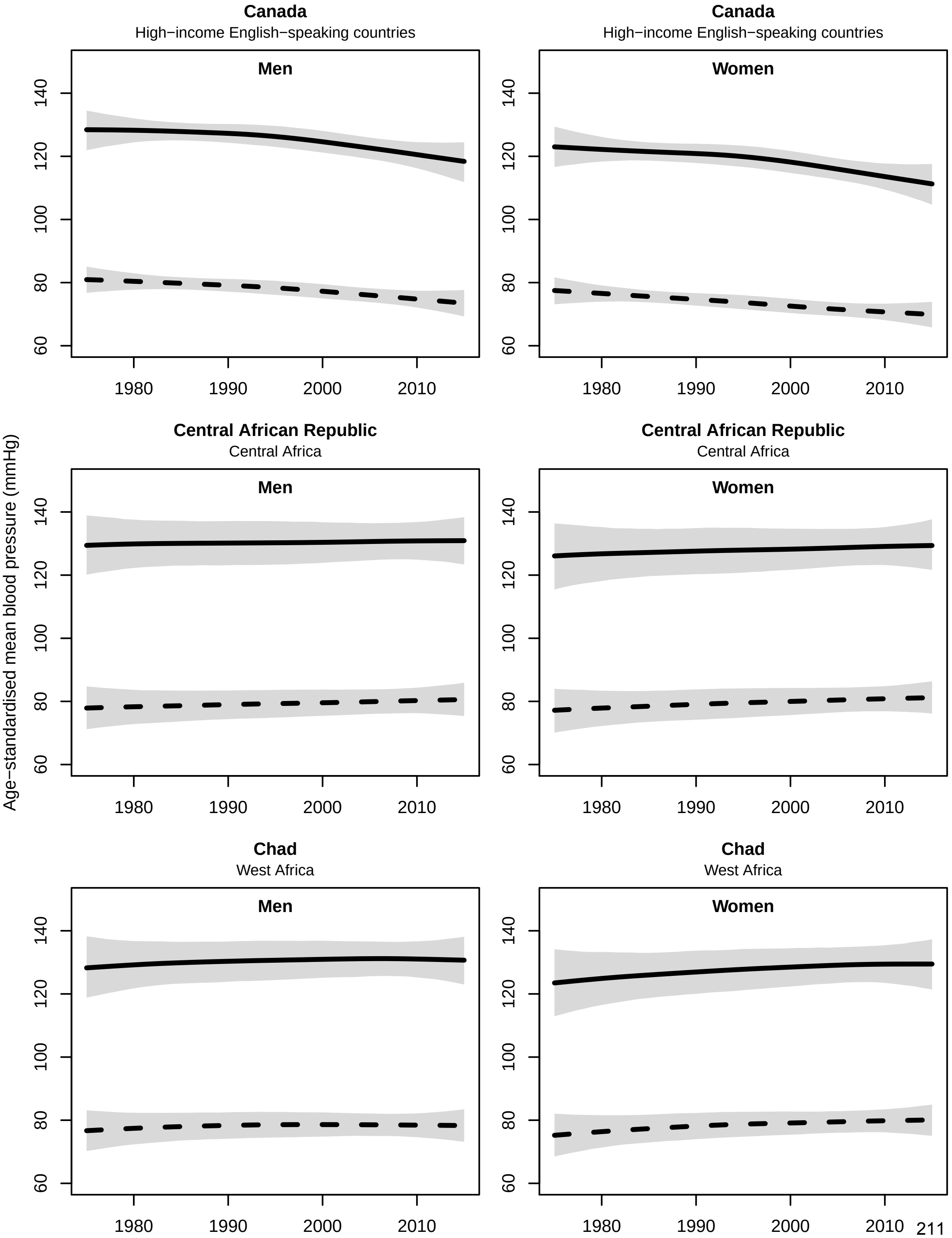
Canada
High−income English−speaking countries
Men
Canada
High−income English−speaking countries
Women
Central African Republic
Central Africa
Men
Central African Republic
Central Africa
Women
Chad
West Africa
Men
Chad
West Africa
Women
Age−standardised mean blood pressure (mmHg)
60
80
100
120
140
1980
1990
2000
2010

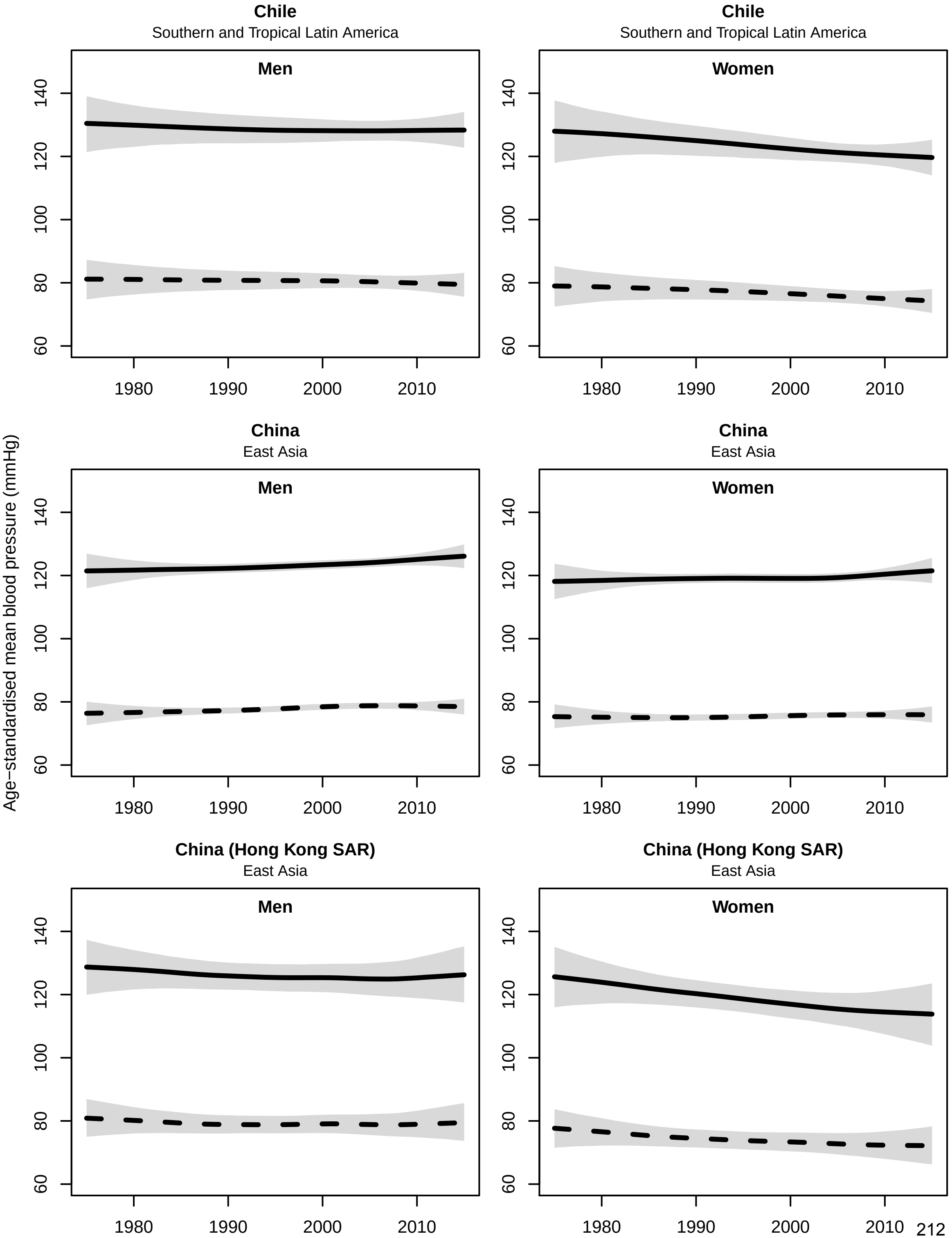
Chile
Southern and Tropical Latin America
Men
Chile
Southern and Tropical Latin America
Women
China
East Asia
Men
China
East Asia
Women
China (Hong Kong SAR)
East Asia
Men
China (Hong Kong SAR)
East Asia
Women
Age−standardised mean blood pressure (mmHg)
60
80
100
120
140
1980
1990
2000
2010

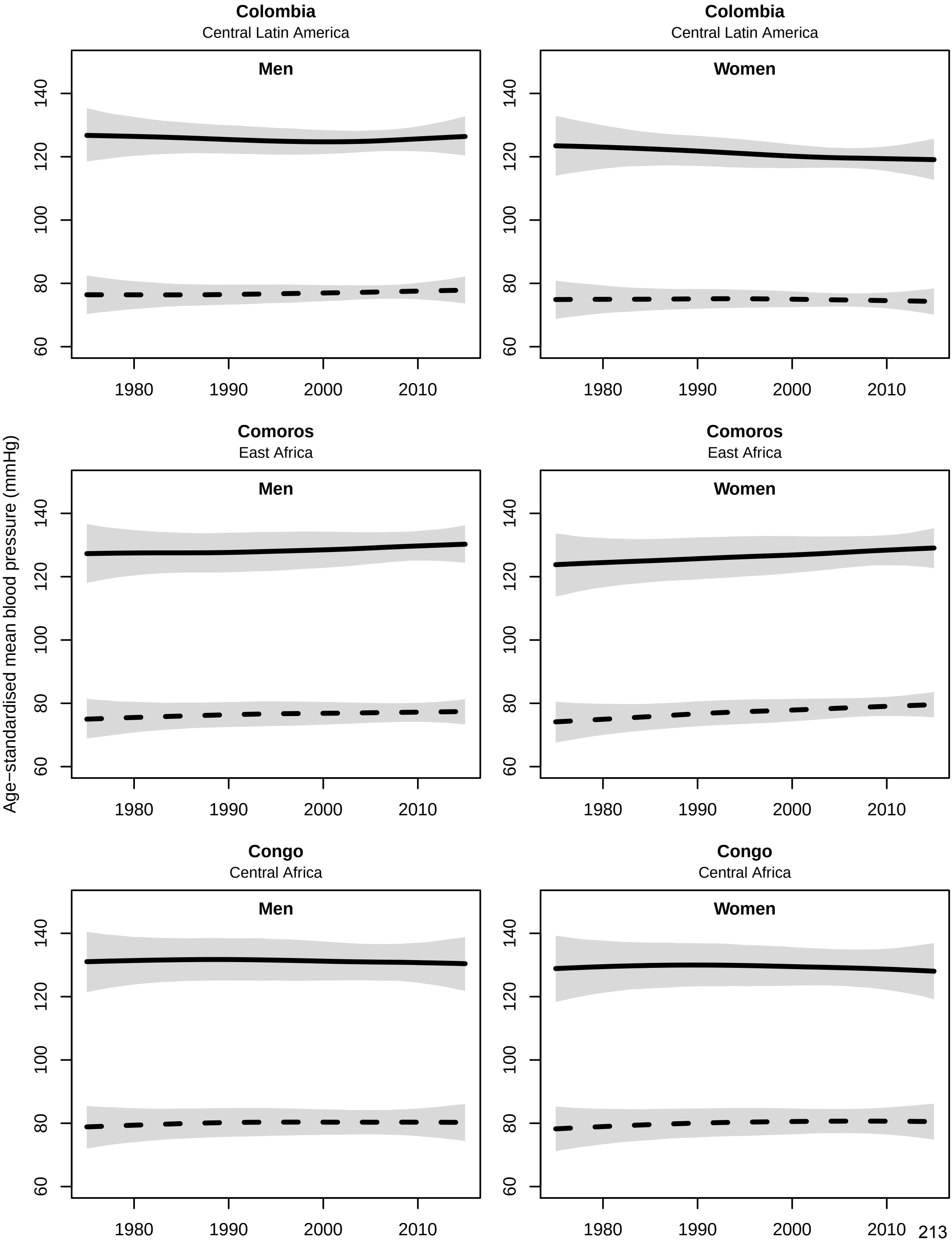
Colombia
Central Latin America
Men
Colombia
Central Latin America
Women
Comoros
East Africa
Men
Comoros
East Africa
Women
Congo
Central Africa
Men
Congo
Central Africa
Women
Age−standardised mean blood pressure (mmHg)
60
80
100
120
140
1980
1990
2000
2010

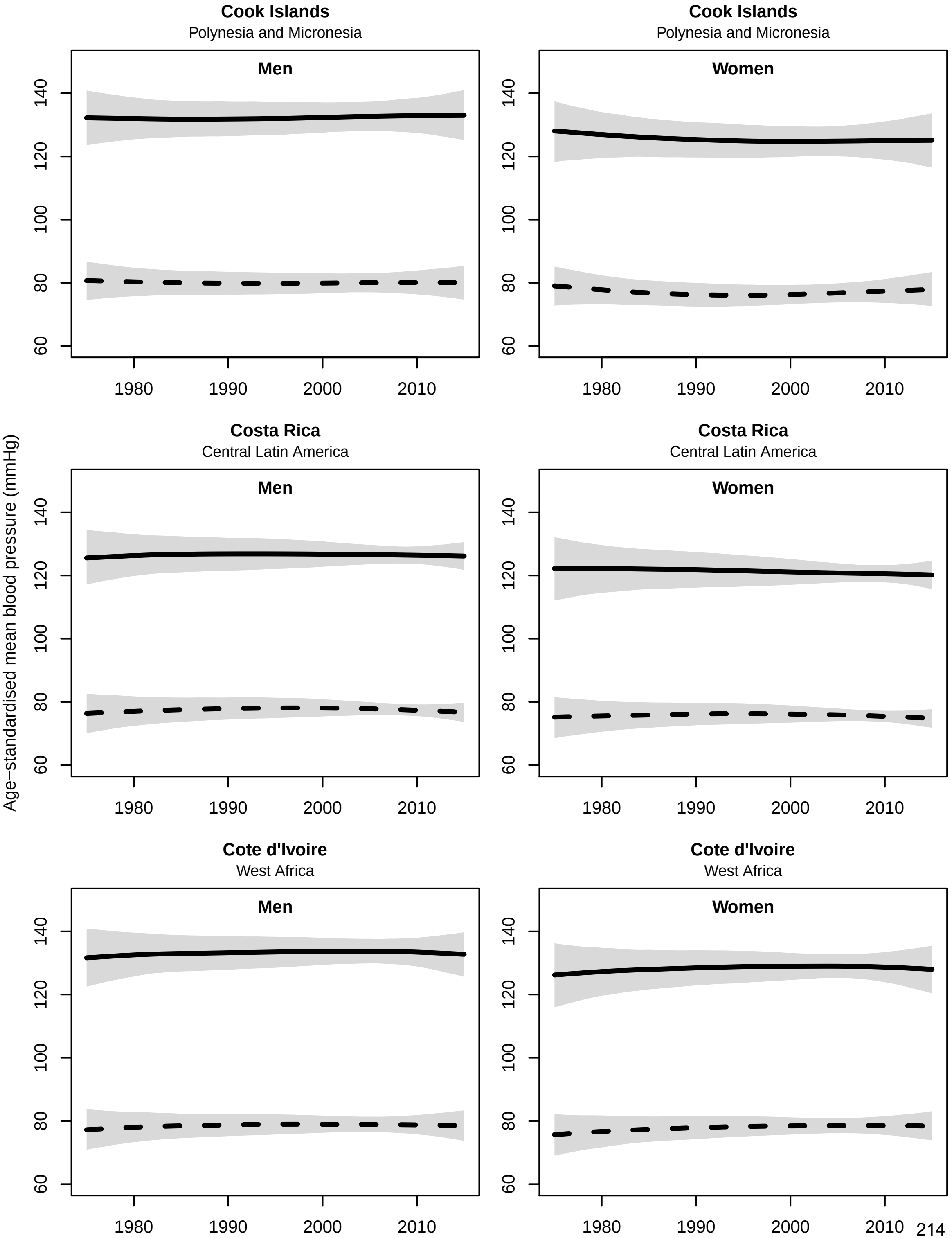
Cook Islands
Polynesia and Micronesia
Men
Cook Islands
Polynesia and Micronesia
Women
Costa Rica
Central Latin America
Men
Costa Rica
Central Latin America
Women
Cote d'Ivoire
West Africa
Men
Cote d'Ivoire
West Africa
Women
Age−standardised mean blood pressure (mmHg)
60
80
100
120
140
1980
1990
2000
2010

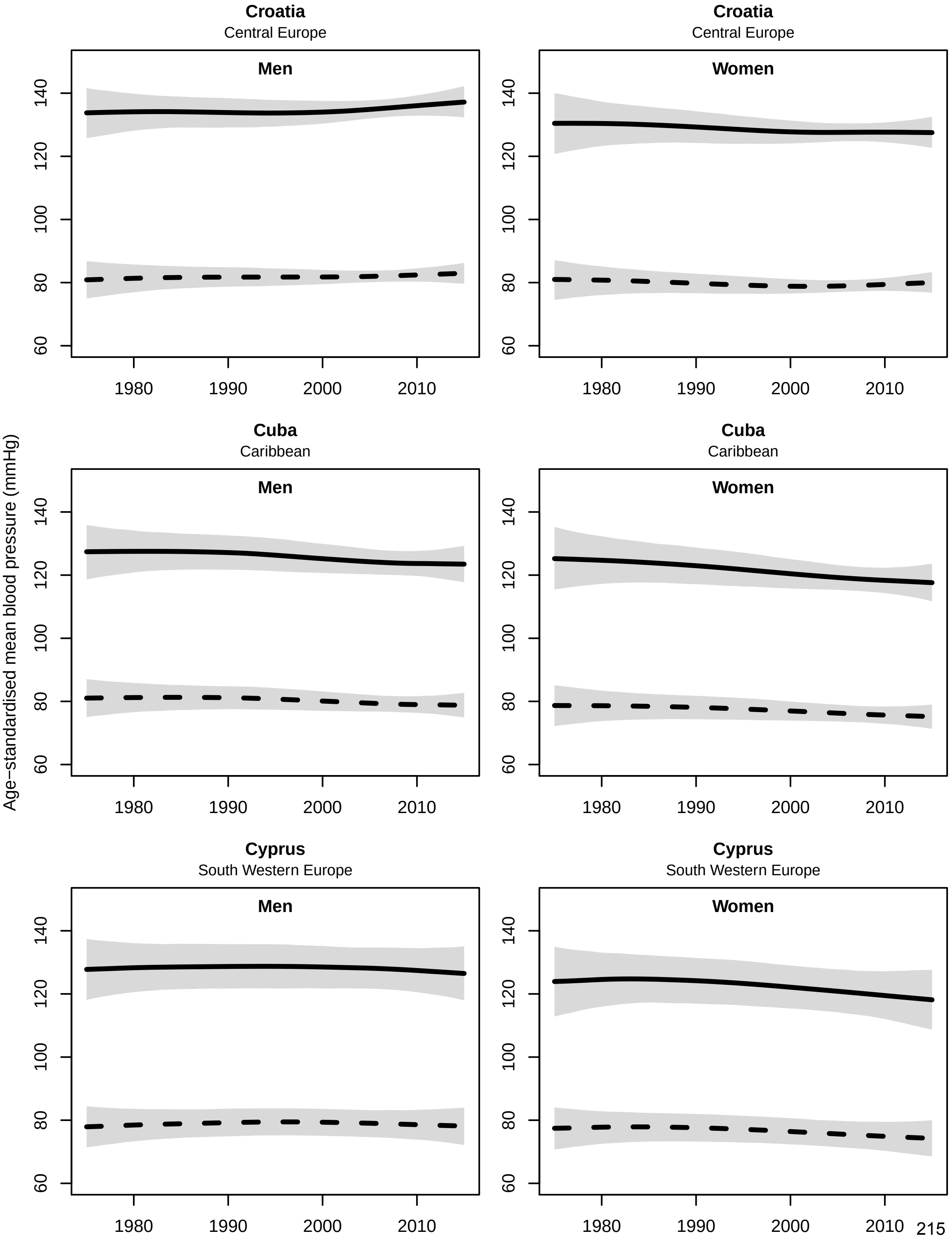
Croatia
Central Europe
Men
Croatia
Central Europe
Women
Cuba
Caribbean
Men
Cuba
Caribbean
Women
Cyprus
South Western Europe
Men
Cyprus
South Western Europe
Women
Age−standardised mean blood pressure (mmHg)
60
80
100
120
140
1980
1990
2000
2010

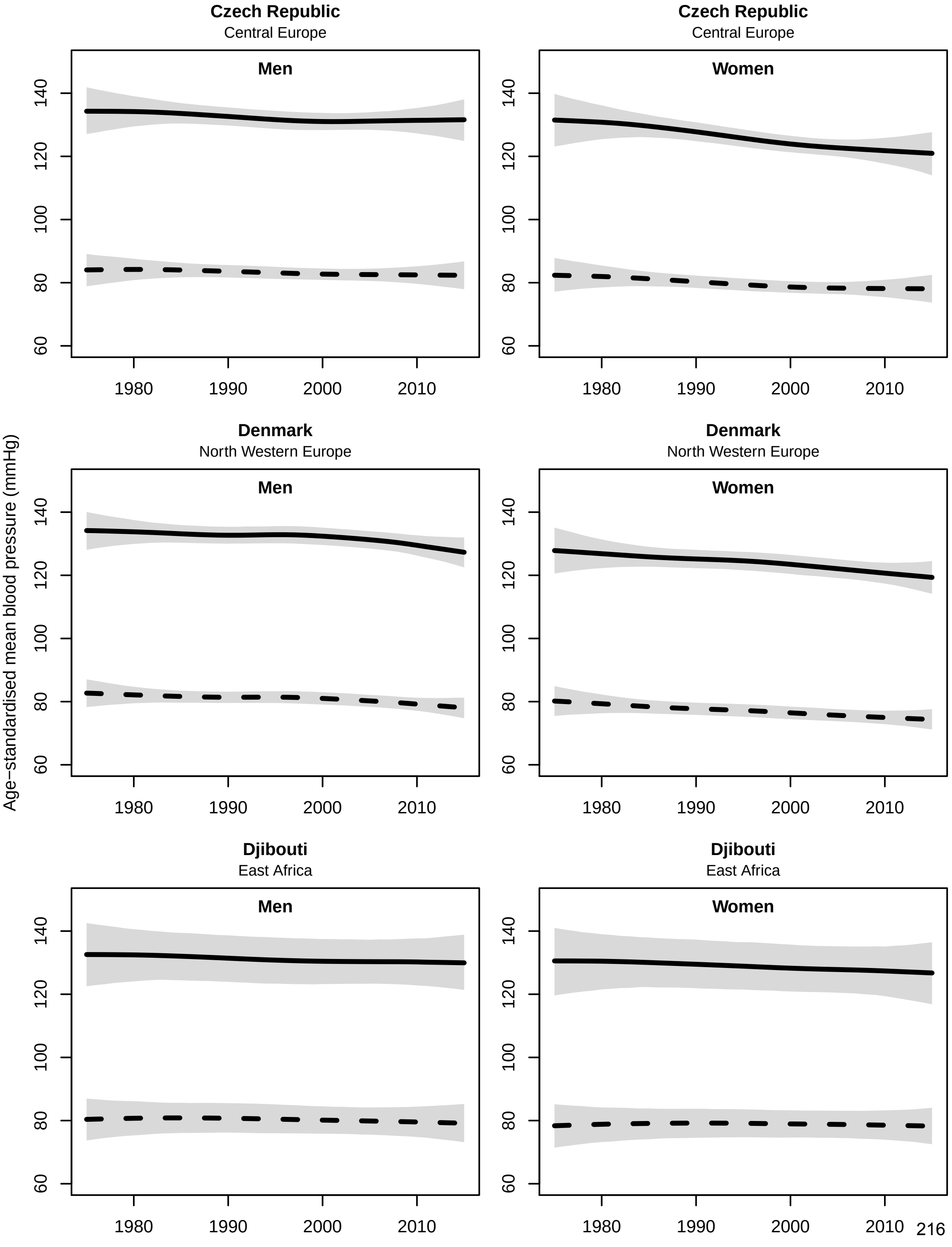

Czech Republic
Central Europe
Men
Czech Republic
Central Europe
Women
Denmark
North Western Europe
Men
Denmark
North Western Europe
Women
Djibouti
East Africa
Men
Djibouti
East Africa
Women
Age−standardised mean blood pressure (mmHg)
60
80
100
120
140
1980
1990
2000
2010

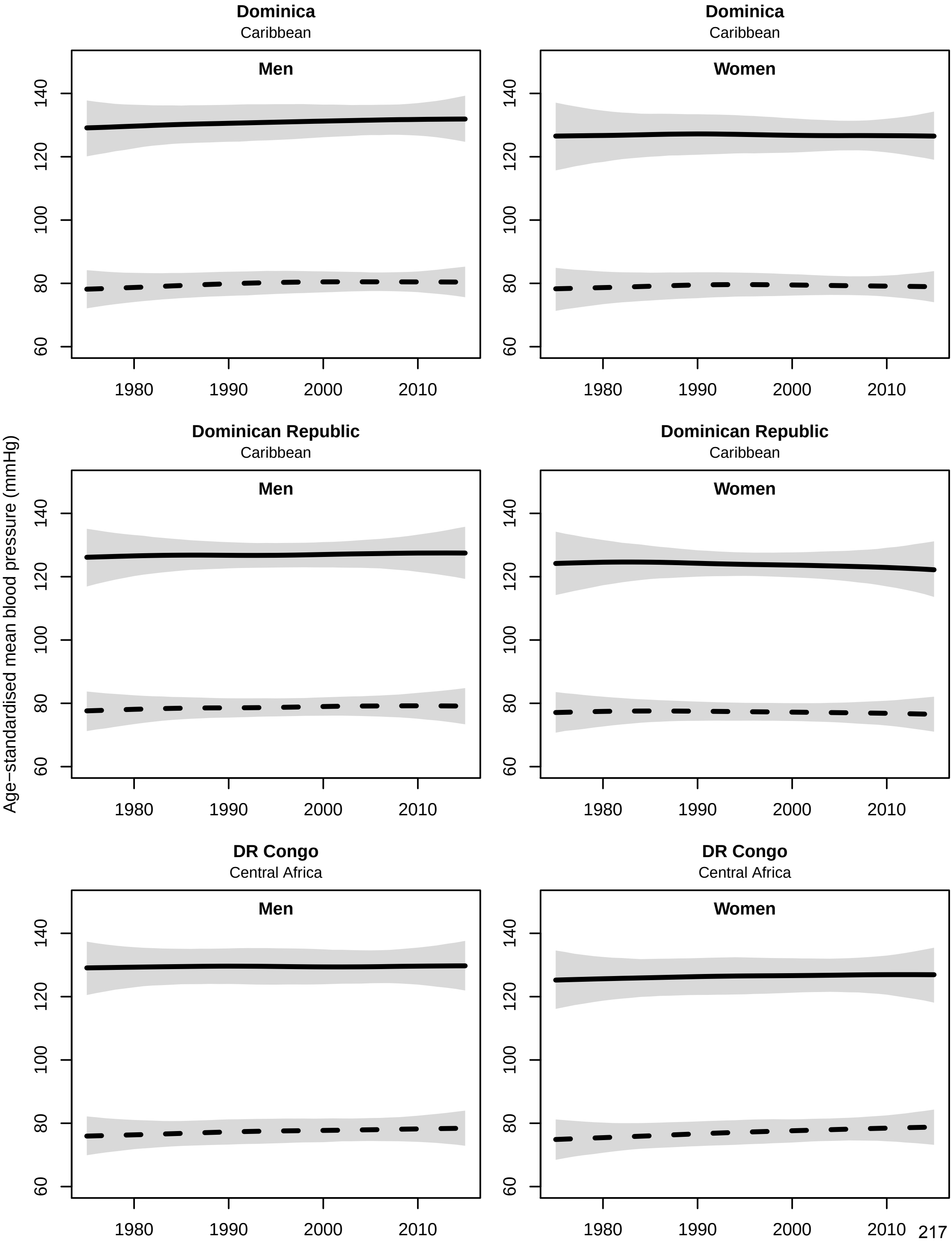
Dominica
Caribbean
Men
Women
Dominican Republic
Caribbean
Men
Women
DR Congo
Central Africa
Men
Women
Age−standardised mean blood pressure (mmHg)
60
80
100
120
140
1980
1990
2000
2010

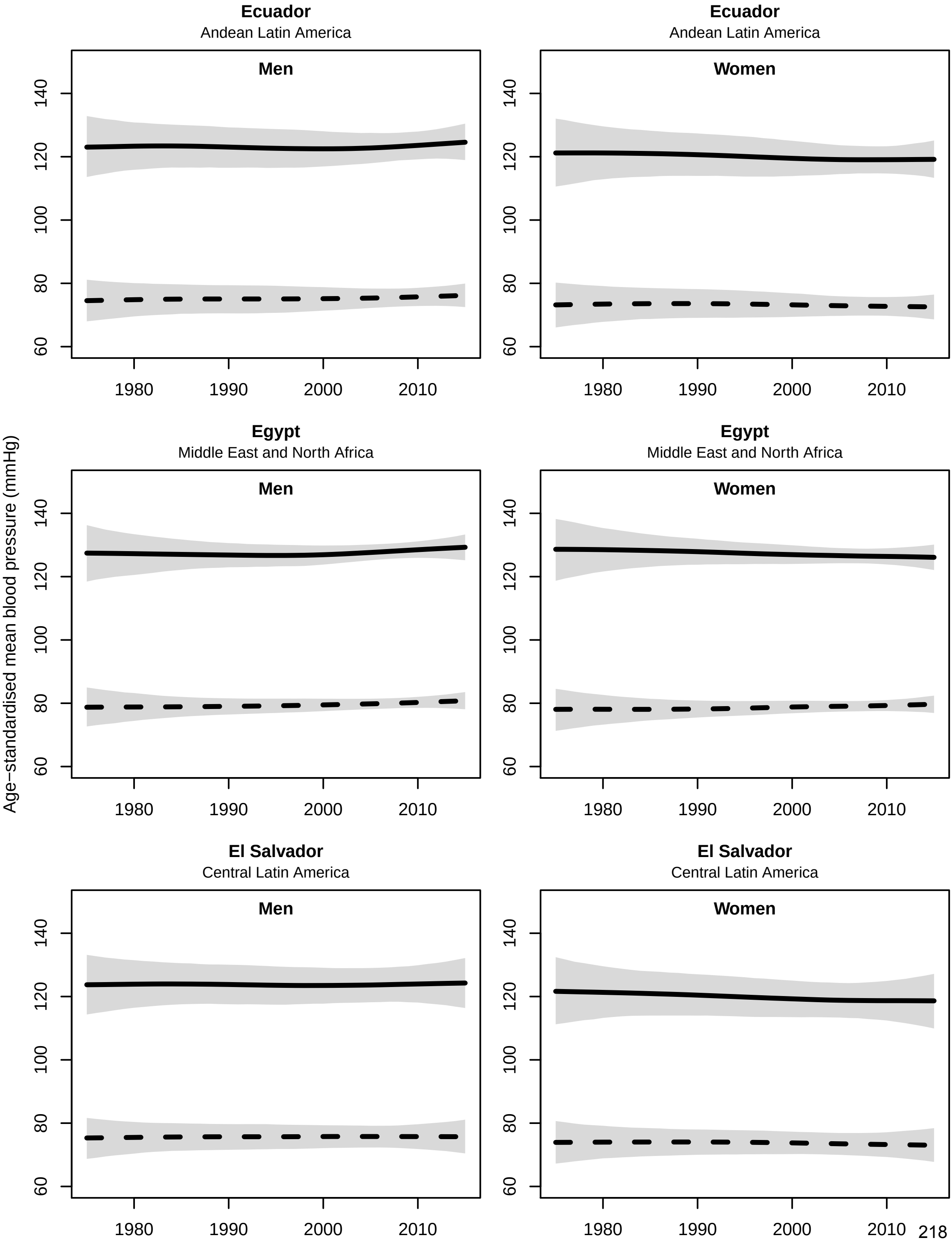

Ecuador
Andean Latin America
Men
Ecuador
Andean Latin America
Women
Egypt
Middle East and North Africa
Men
Egypt
Middle East and North Africa
Women
El Salvador
Central Latin America
Men
El Salvador
Central Latin America
Women
Age−standardised mean blood pressure (mmHg)
60
80
100
120
140
1980
1990
2000
2010

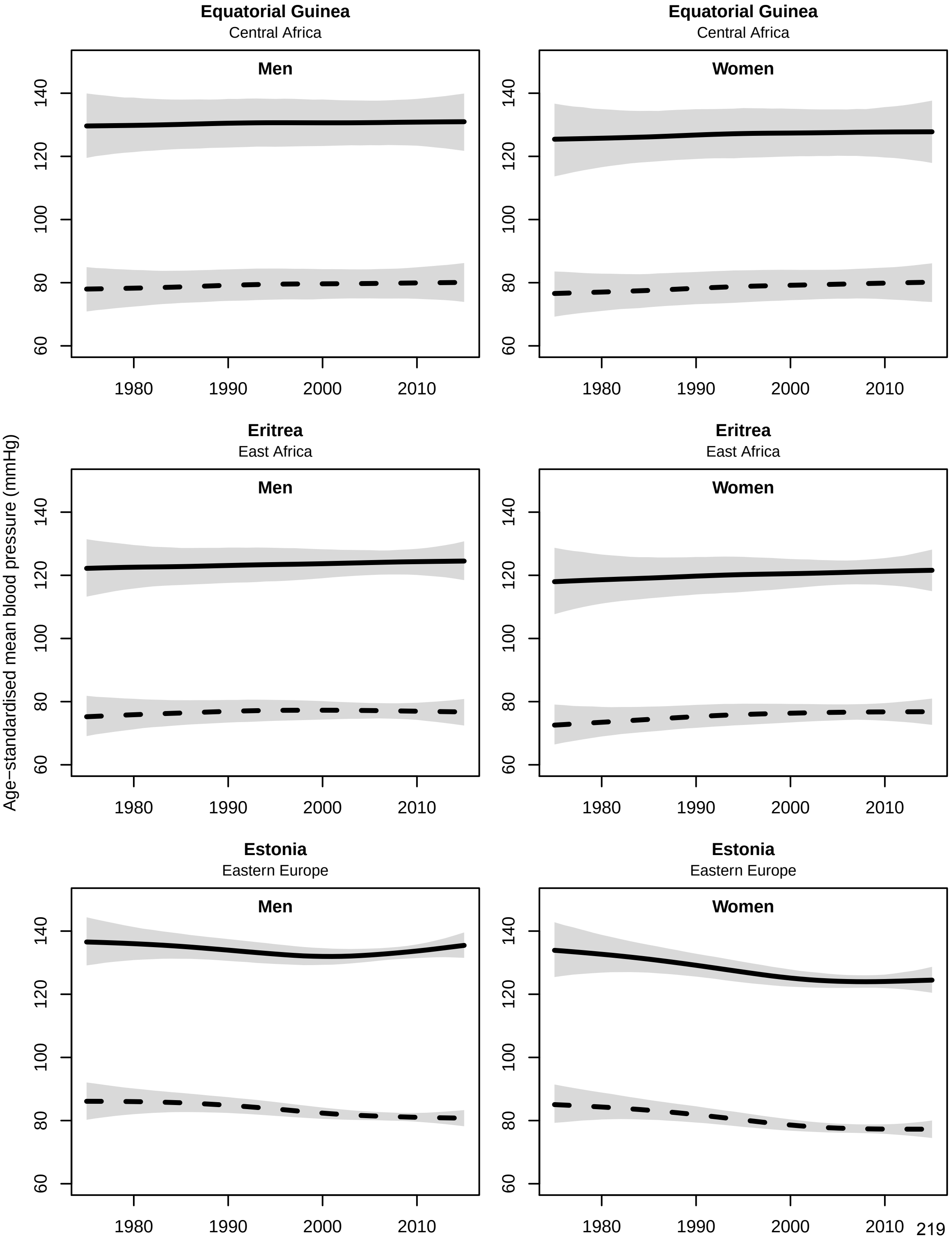
Equatorial Guinea
Central Africa
Men
Equatorial Guinea
Central Africa
Women
Eritrea
East Africa
Men
Eritrea
East Africa
Women
Estonia
Eastern Europe
Men
Estonia
Eastern Europe
Women
Age−standardised mean blood pressure (mmHg)
60
80
100
120
140
1980
1990
2000
2010

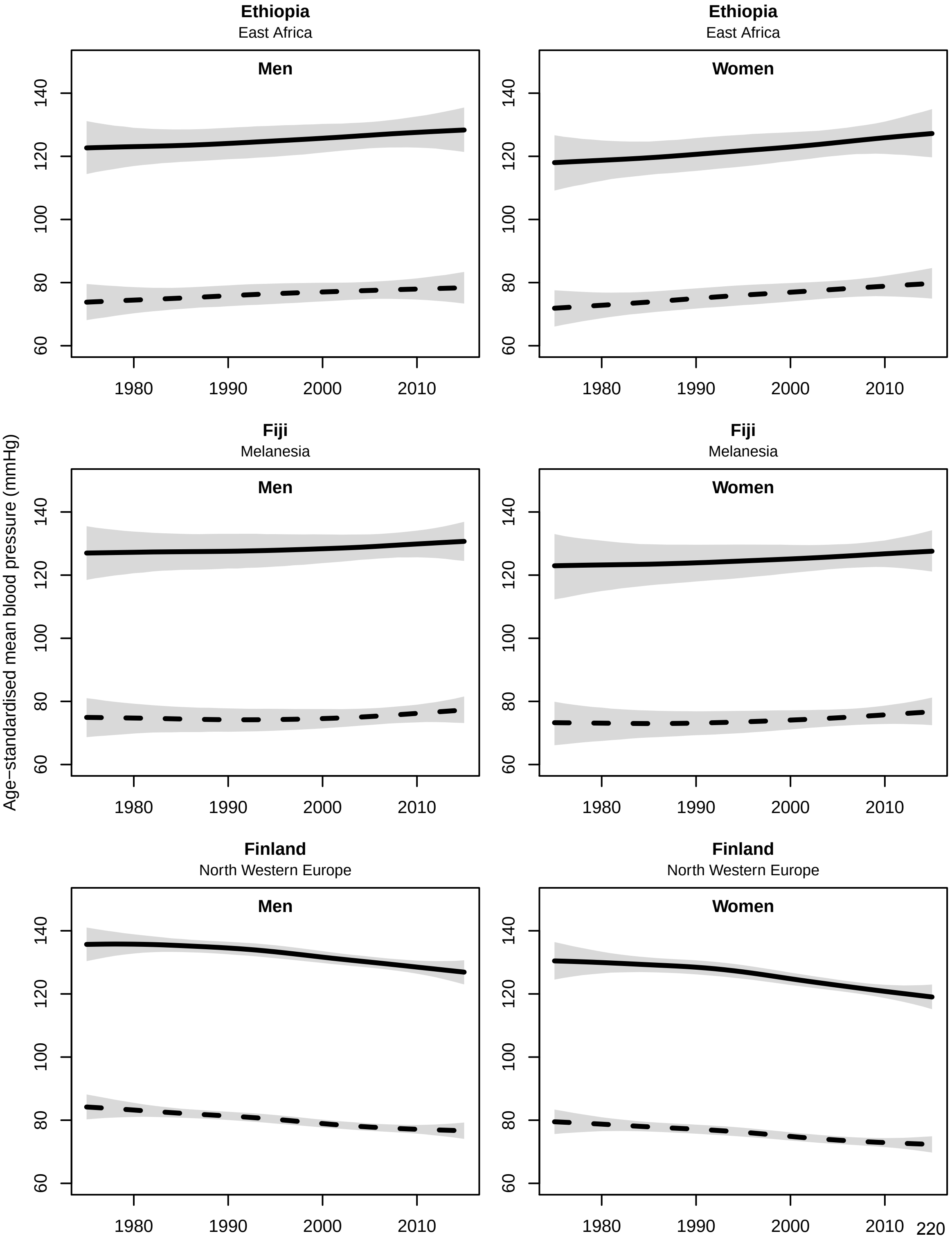
Ethiopia
East Africa
Men
Ethiopia
East Africa
Women
Fiji
Melanesia
Men
Fiji
Melanesia
Women
Finland
North Western Europe
Men
Finland
North Western Europe
Women
Age−standardised mean blood pressure (mmHg)
60
80
100
120
140
1980
1990
2000
2010

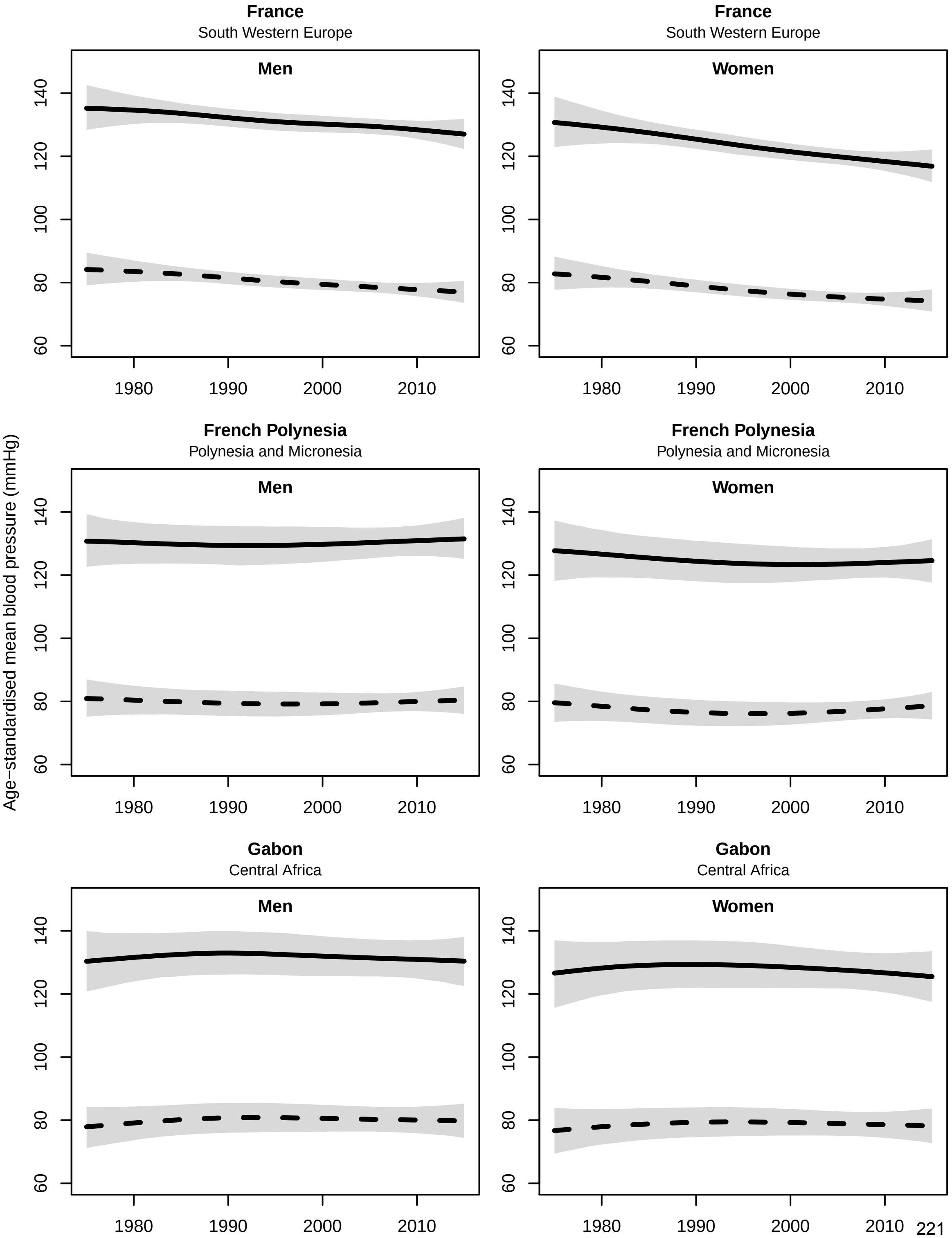

France
South Western Europe
Men
France
South Western Europe
Women
French Polynesia
Polynesia and Micronesia
Men
French Polynesia
Polynesia and Micronesia
Women
Gabon
Central Africa
Men
Gabon
Central Africa
Women
Age−standardised mean blood pressure (mmHg)
60
80
100
120
140
1980
1990
2000
2010

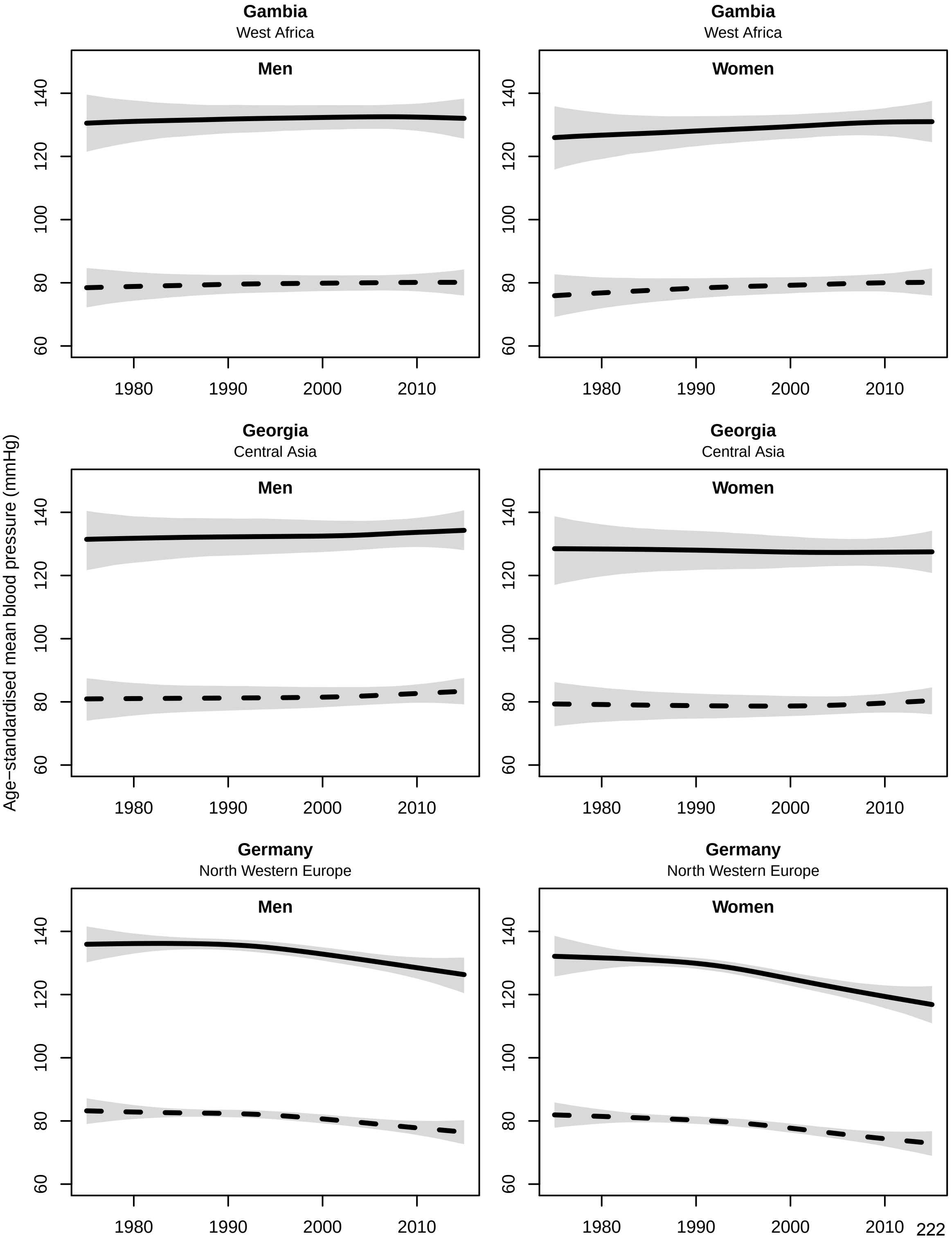

Gambia
West Africa
Men
Gambia
West Africa
Women
Georgia
Central Asia
Men
Georgia
Central Asia
Women
Germany
North Western Europe
Men
Germany
North Western Europe
Women
Age−standardised mean blood pressure (mmHg)
60
80
100
120
140
1980
1990
2000
2010

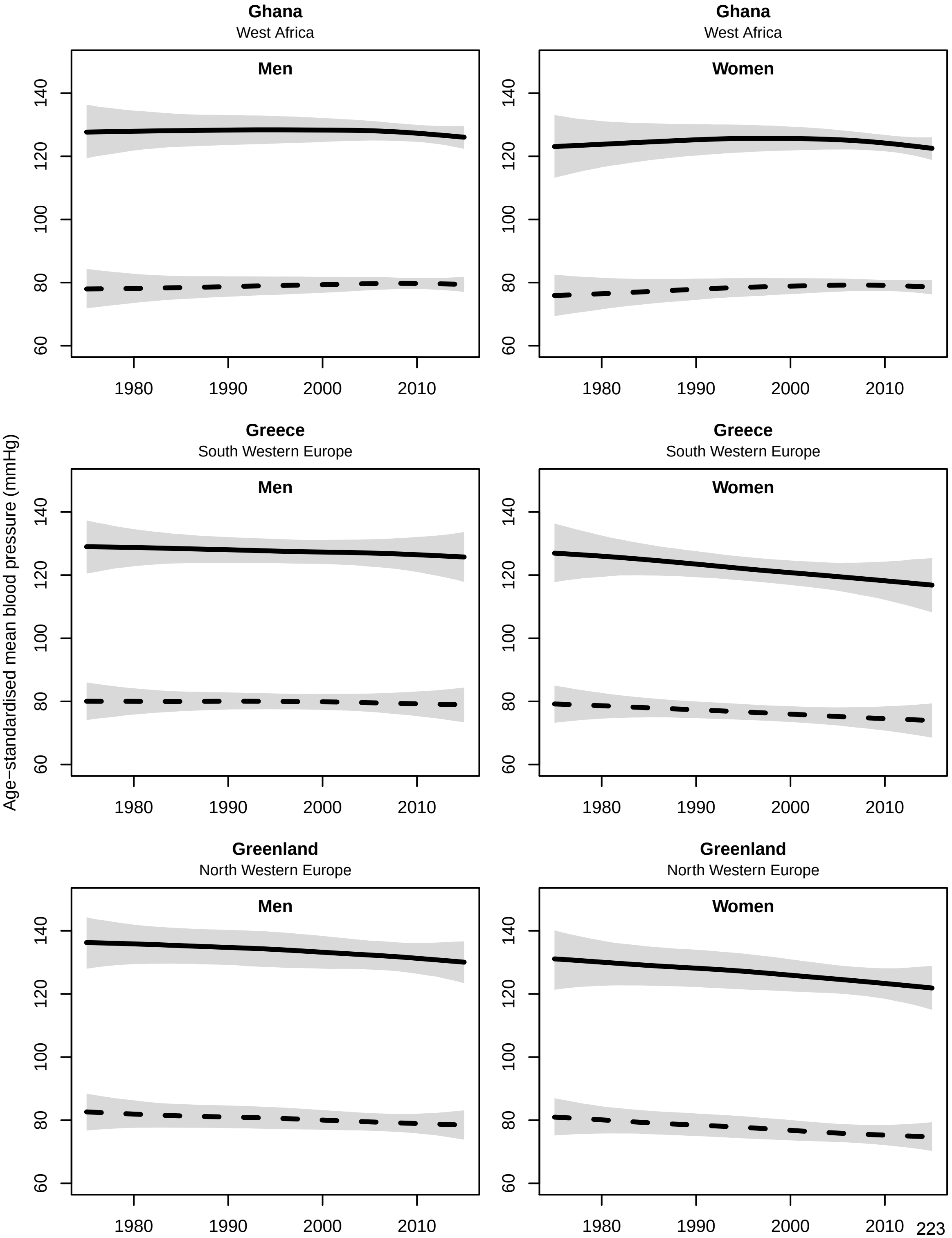
Ghana
West Africa
Men
Ghana
West Africa
Women
Greece
South Western Europe
Men
Greece
South Western Europe
Women
Greenland
North Western Europe
Men
Greenland
North Western Europe
Women
Age−standardised mean blood pressure (mmHg)
60
80
100
120
140
1980
1990
2000
2010

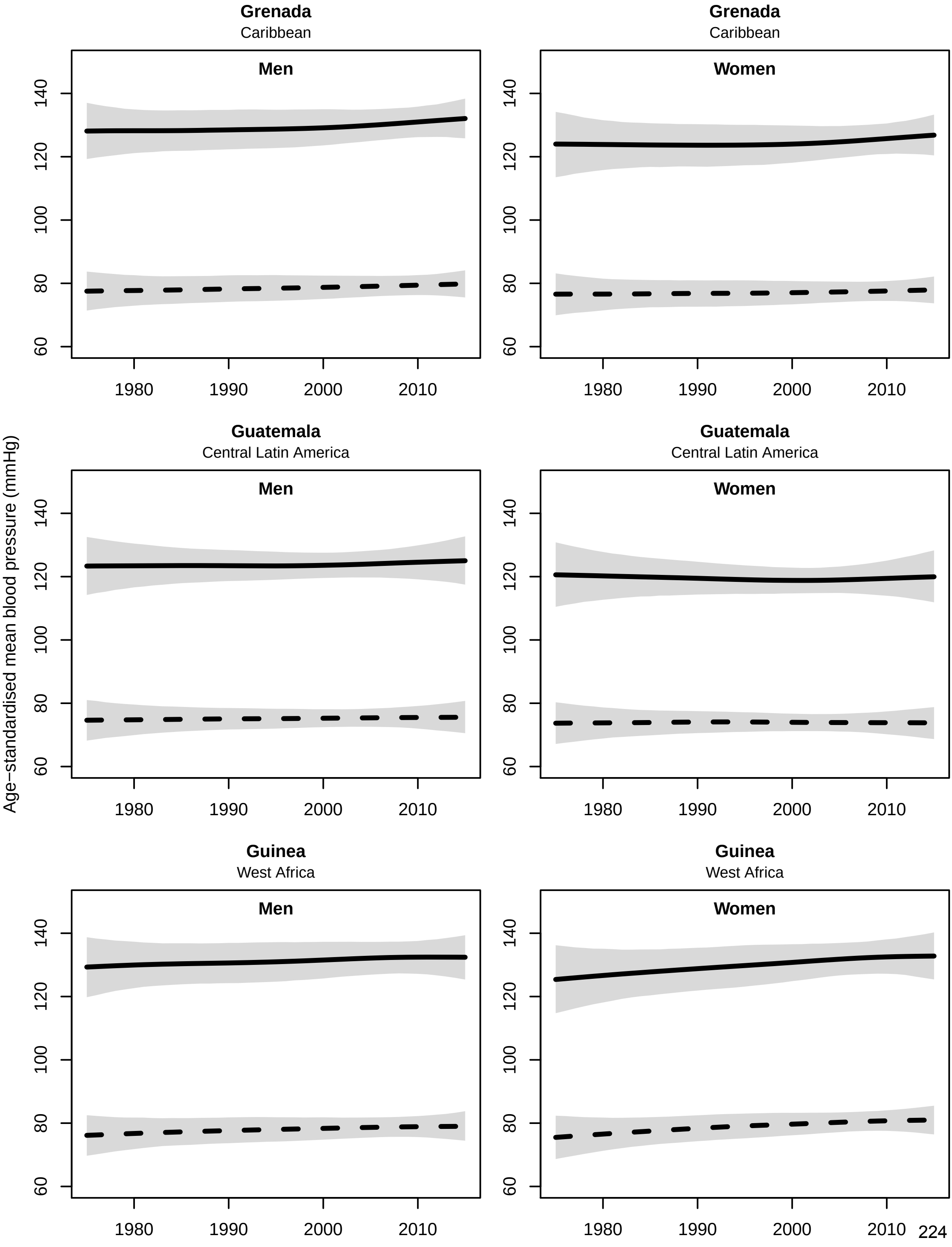
Grenada
Caribbean
Men
Grenada
Caribbean
Women
Guatemala
Central Latin America
Men
Guatemala
Central Latin America
Women
Guinea
West Africa
Men
Guinea
West Africa
Women
Age−standardised mean blood pressure (mmHg)
60
80
100
120
140
1980
1990
2000
2010

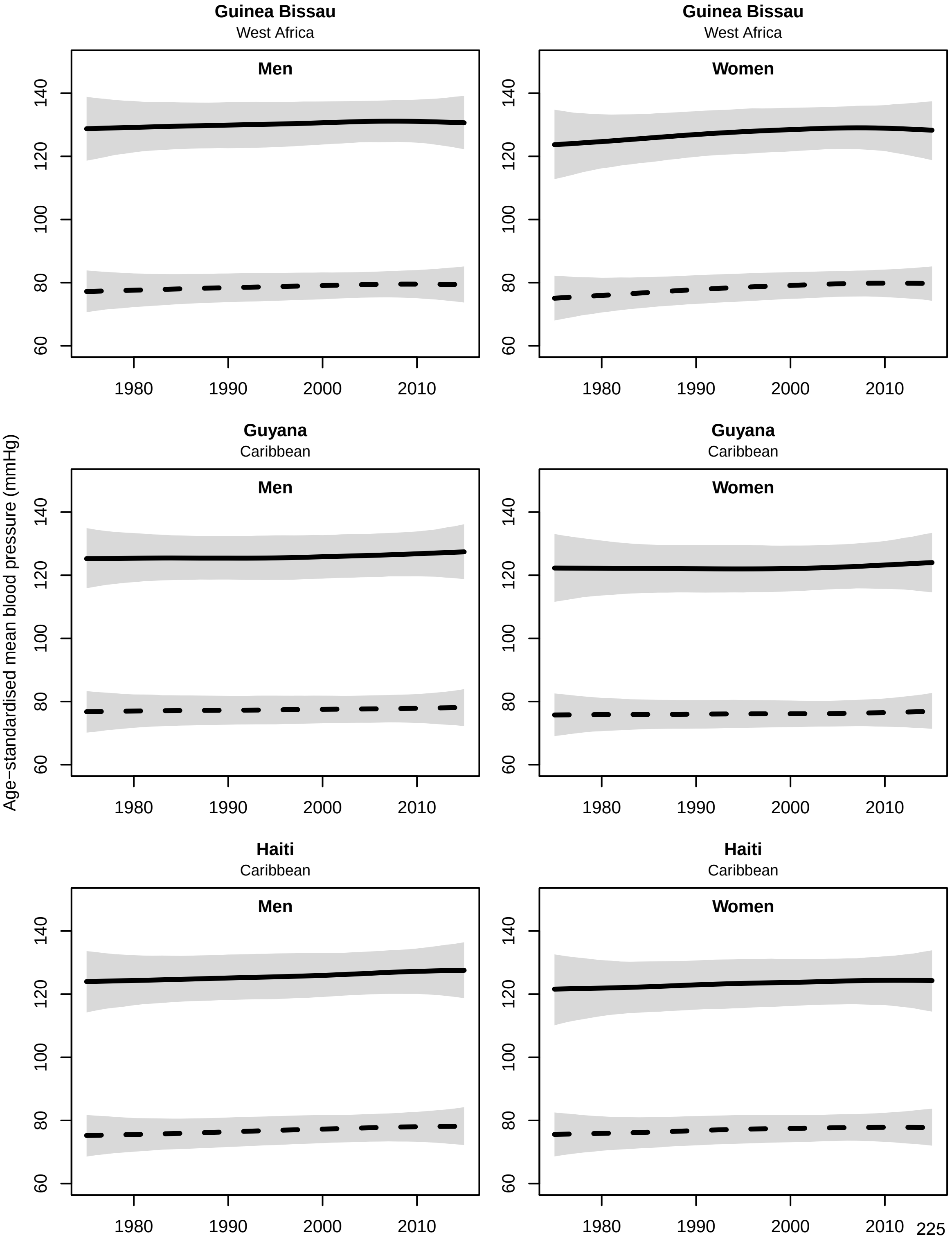
Guinea Bissau
West Africa
Men
Women
Guyana
Caribbean
Men
Women
Haiti
Caribbean
Men
Women
Age−standardised mean blood pressure (mmHg)
60
80
100
120
140
1980
1990
2000
2010

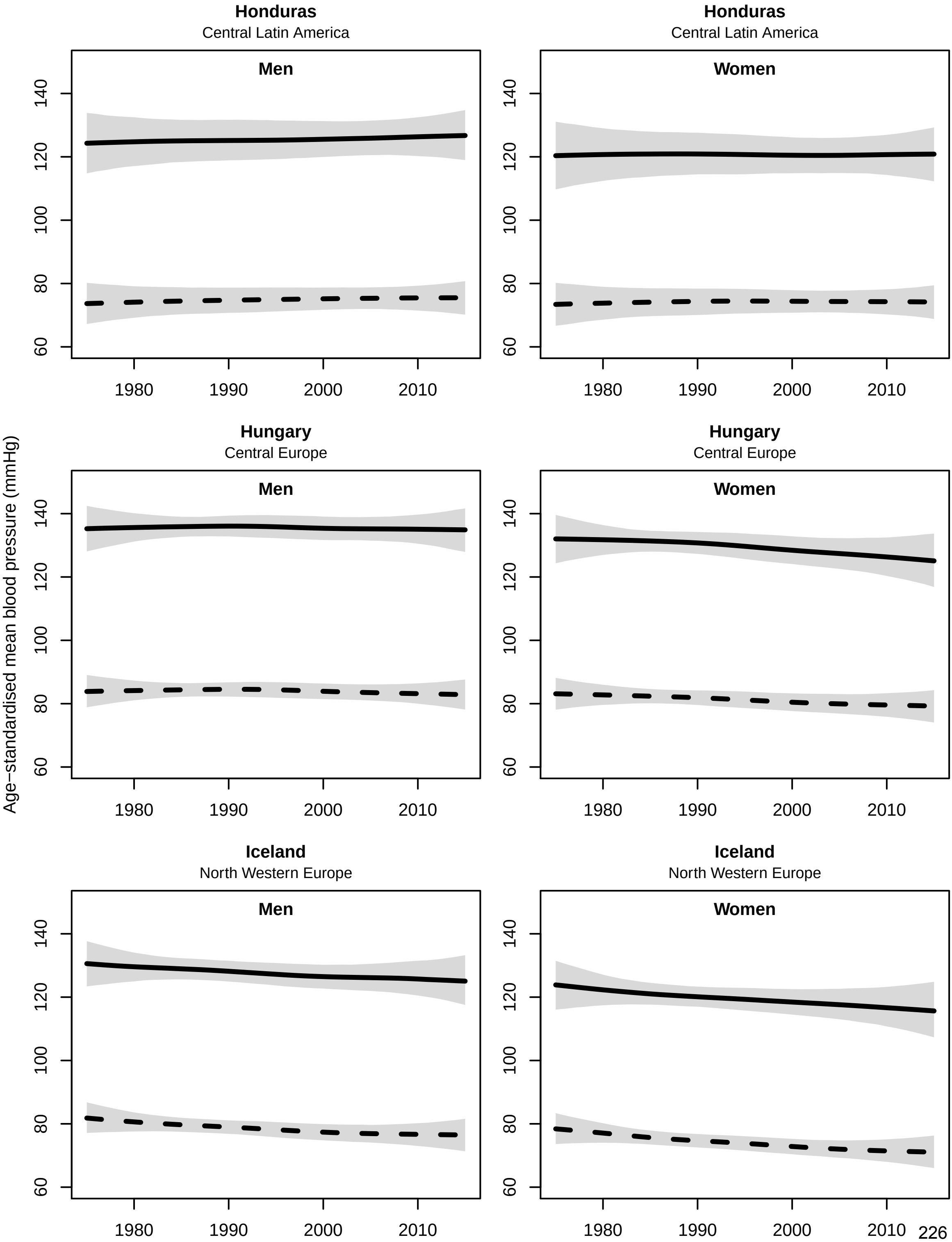
Honduras
Central Latin America
Men
Women
Hungary
Central Europe
Iceland
North Western Europe
Age−standardised mean blood pressure (mmHg)
60
80
100
120
140
1980
1990
2000
2010

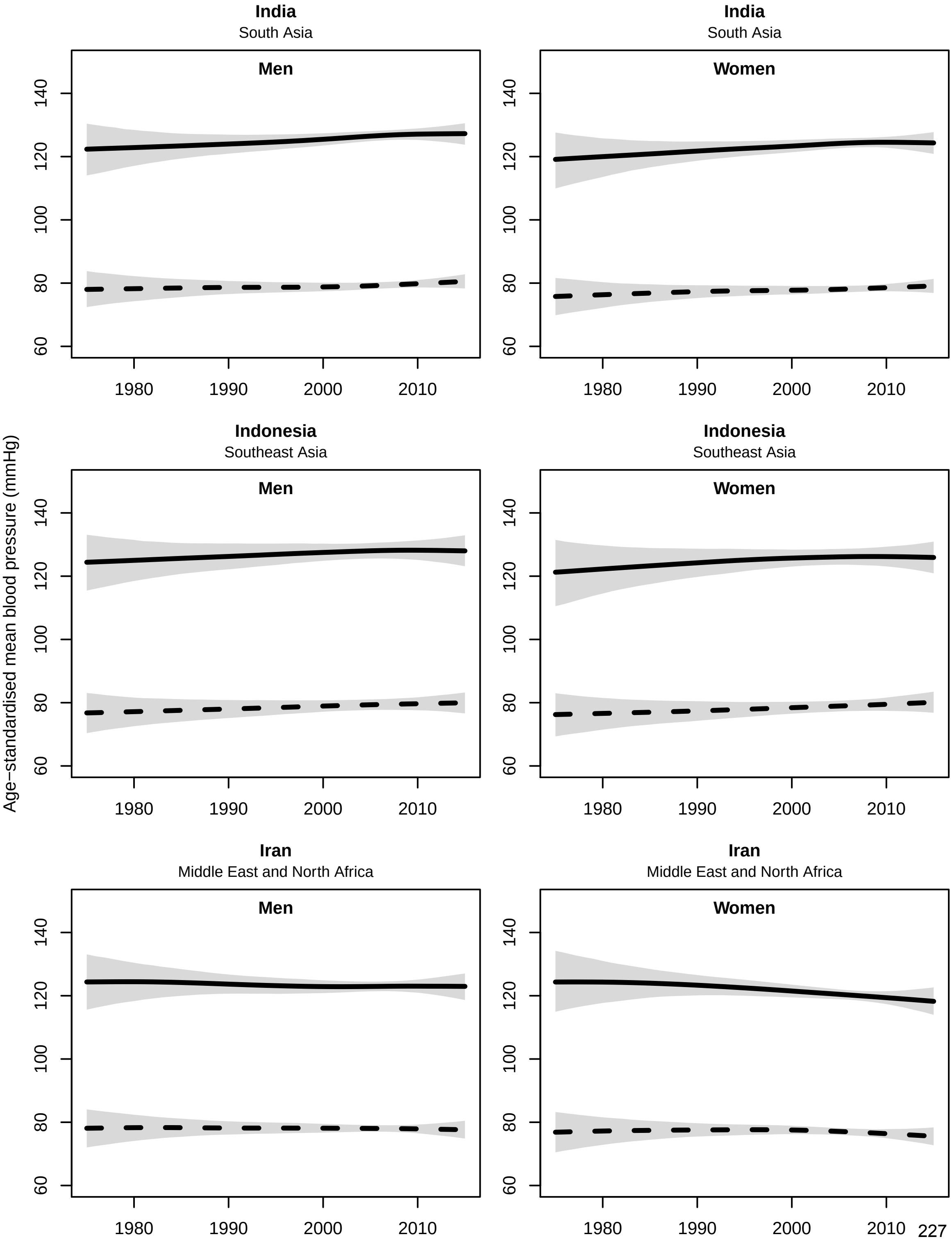
India
South Asia
Men
India
South Asia
Women
Indonesia
Southeast Asia
Men
Indonesia
Southeast Asia
Women
Iran
Middle East and North Africa
Men
Iran
Middle East and North Africa
Women
Age−standardised mean blood pressure (mmHg)
60
80
100
120
140
1980
1990
2000
2010

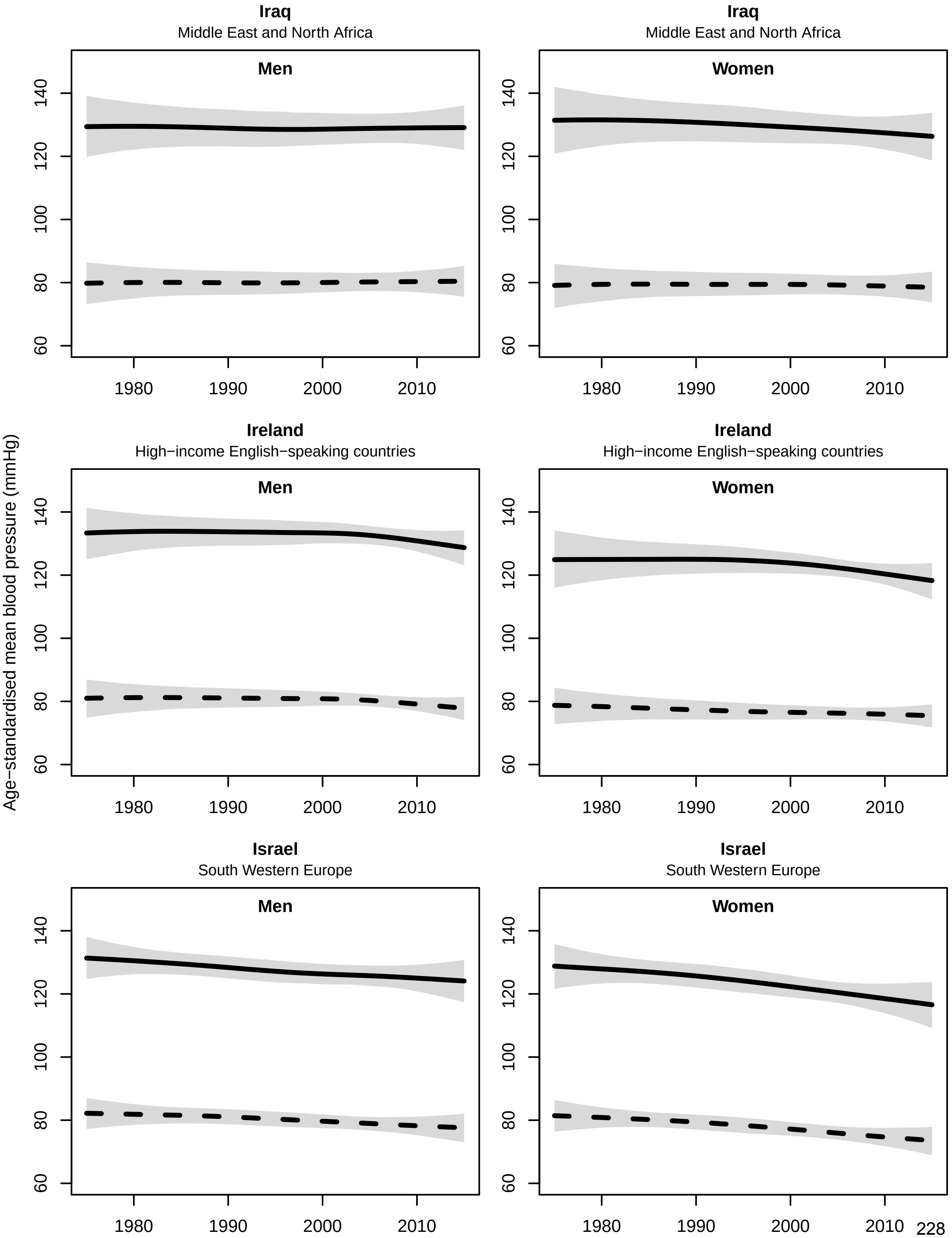
Iraq
Middle East and North Africa
Men
Women
Ireland
High−income English−speaking countries
Israel
South Western Europe
Age−standardised mean blood pressure (mmHg)
60
80
100
120
140
1980
1990
2000
2010

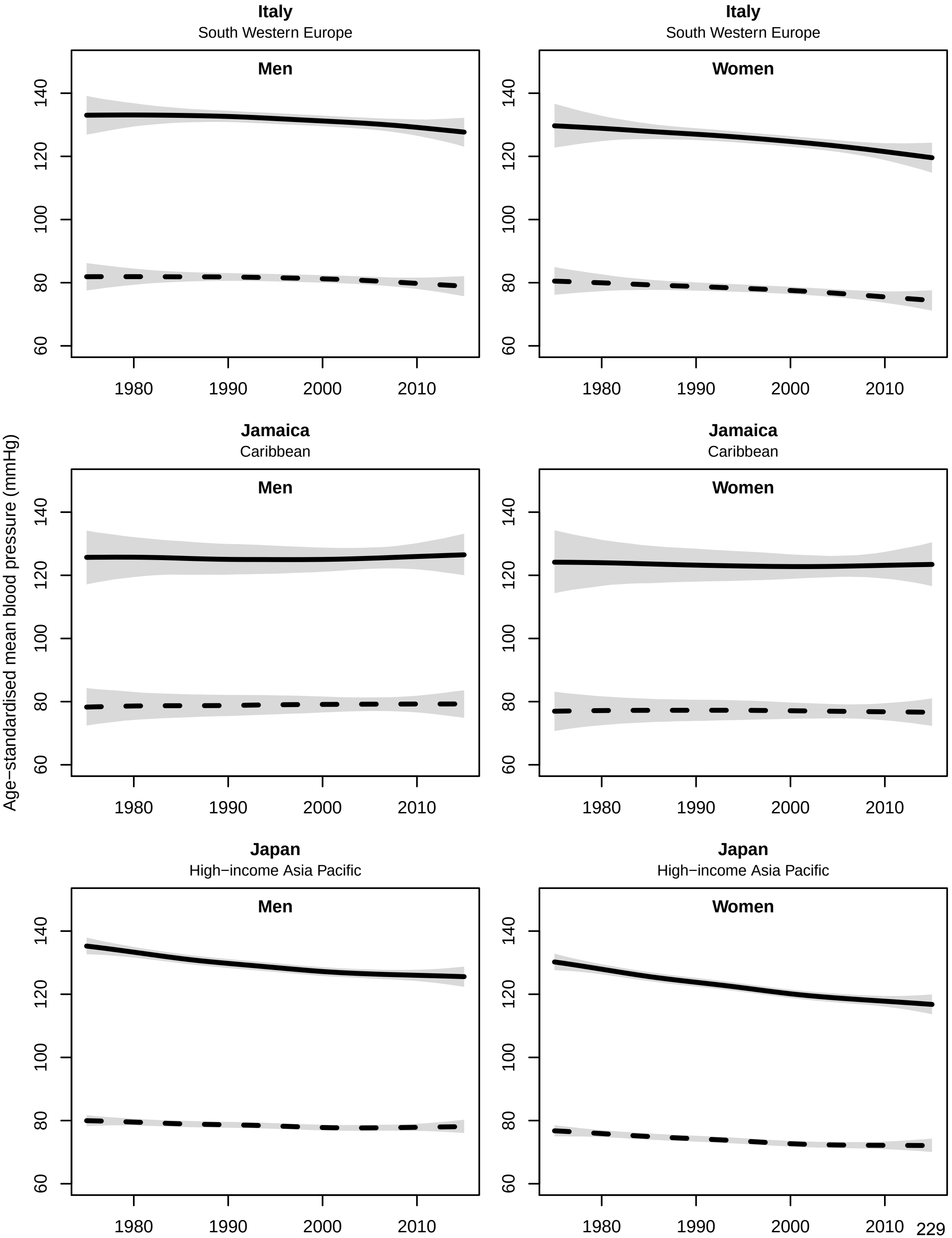

Italy
South Western Europe
Men
Italy
South Western Europe
Women
Jamaica
Caribbean
Men
Jamaica
Caribbean
Women
Japan
High−income Asia Pacific
Men
Japan
High−income Asia Pacific
Women
Age−standardised mean blood pressure (mmHg)
60
80
100
120
140
1980
1990
2000
2010

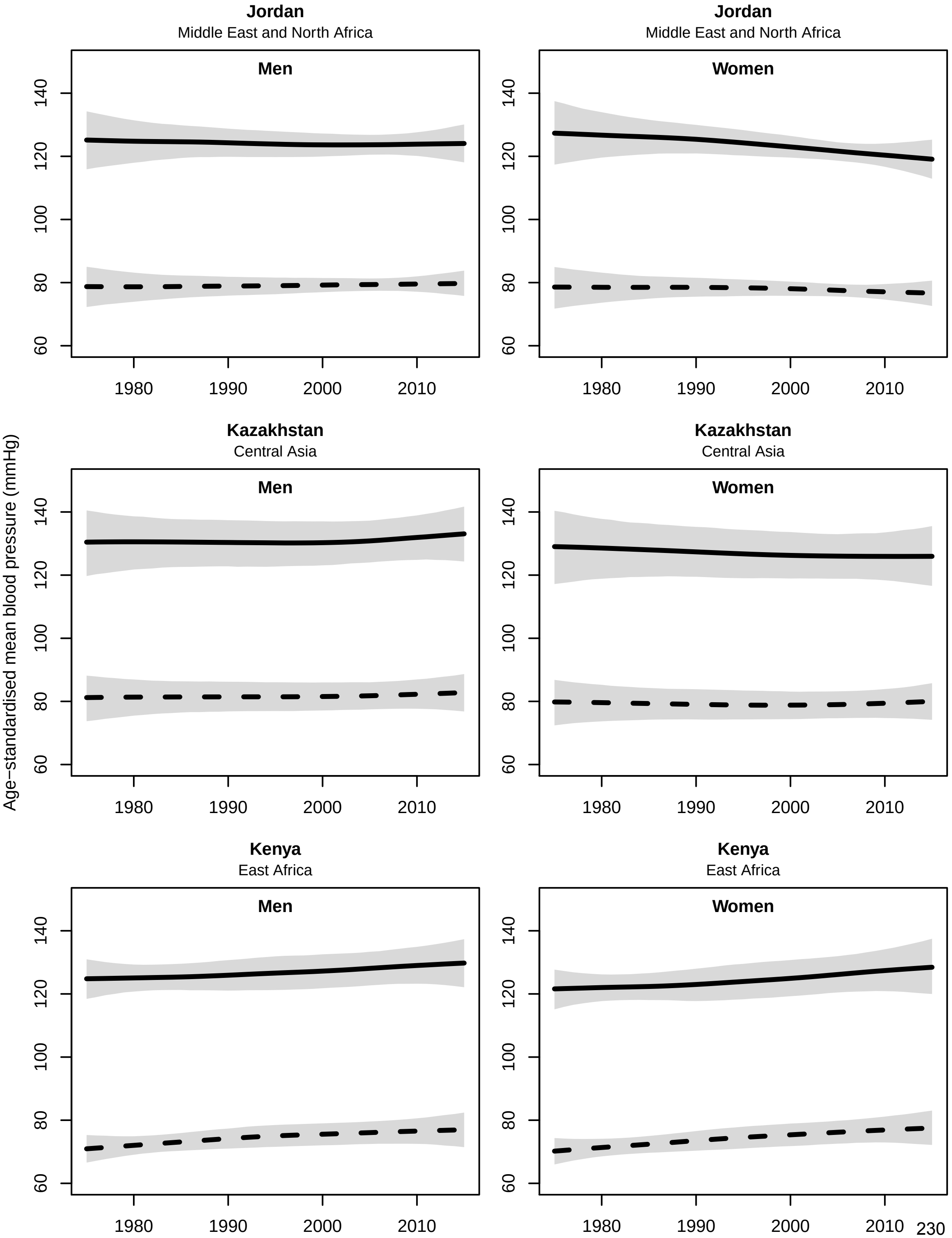

Jordan
Middle East and North Africa
Men
Women
Kazakhstan
Central Asia
Kenya
East Africa
Age−standardised mean blood pressure (mmHg)
60
80
100
120
140
1980
1990
2000
2010

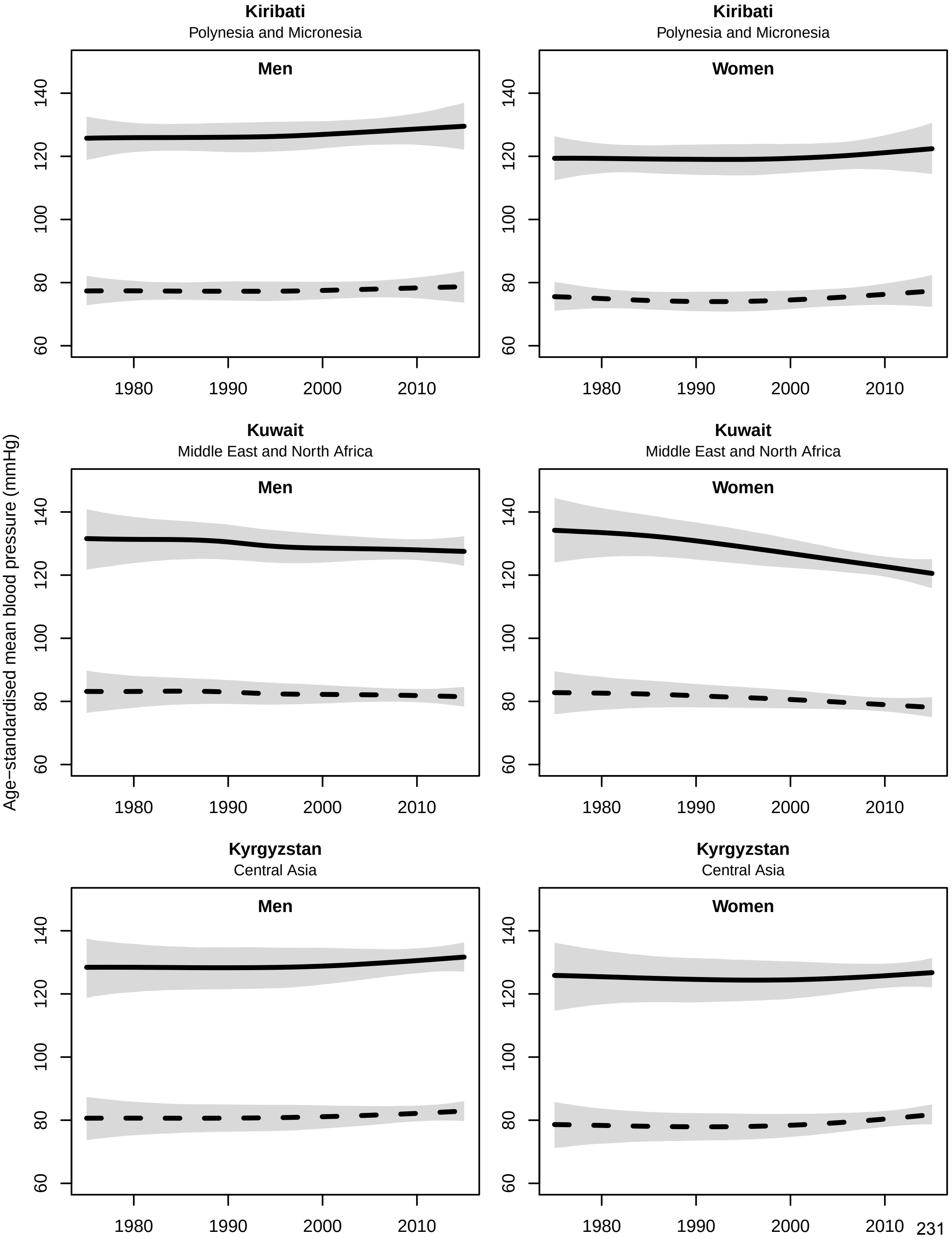
Kiribati
Polynesia and Micronesia
Men
Kiribati
Polynesia and Micronesia
Women
Kuwait
Middle East and North Africa
Men
Kuwait
Middle East and North Africa
Women
Kyrgyzstan
Central Asia
Men
Kyrgyzstan
Central Asia
Women
Age−standardised mean blood pressure (mmHg)
60
80
100
120
140
1980
1990
2000
2010

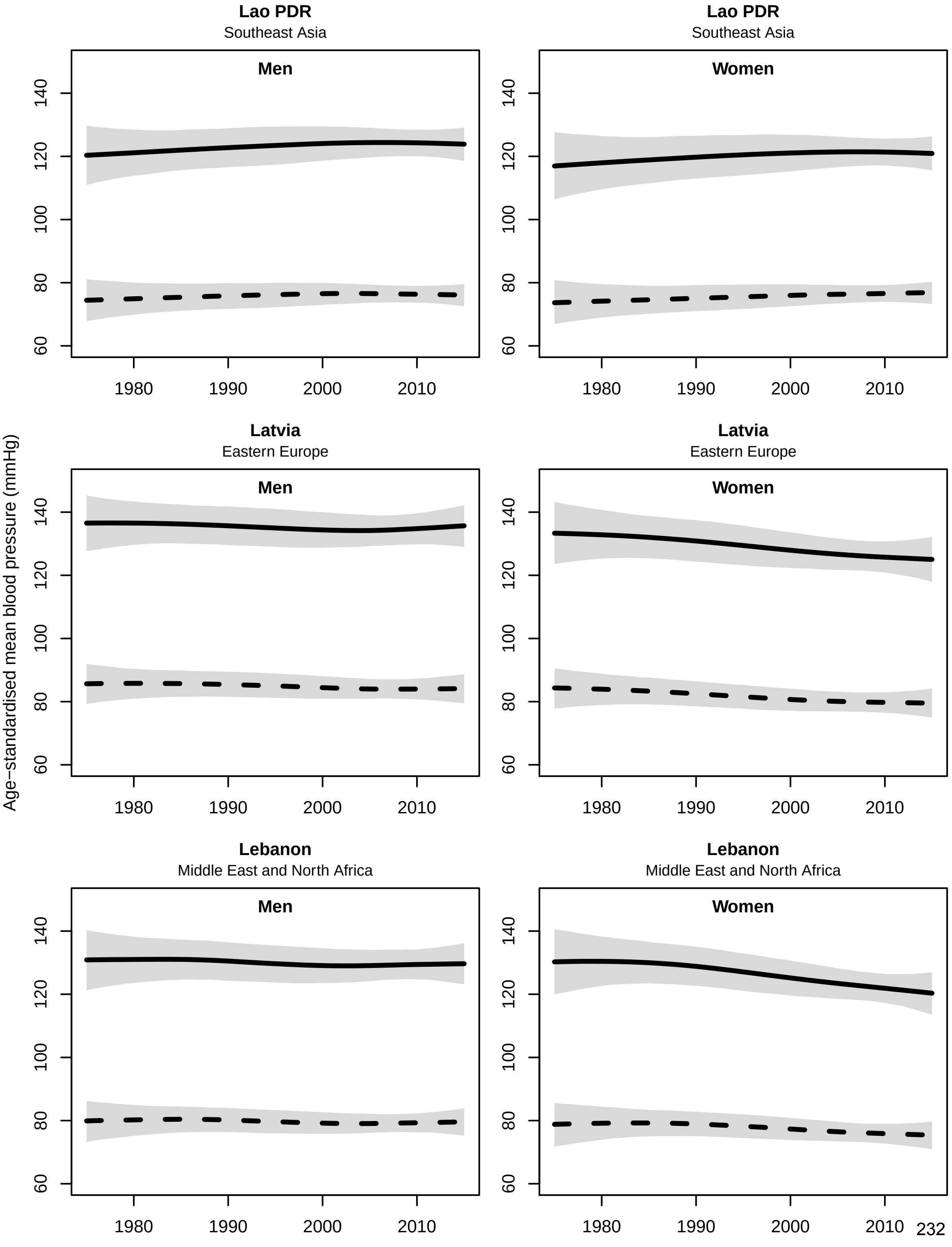
Lao PDR
Southeast Asia
Men
Women
Latvia
Eastern Europe
Lebanon
Middle East and North Africa
Age−standardised mean blood pressure (mmHg)
60
80
100
120
140
1980
1990
2000
2010

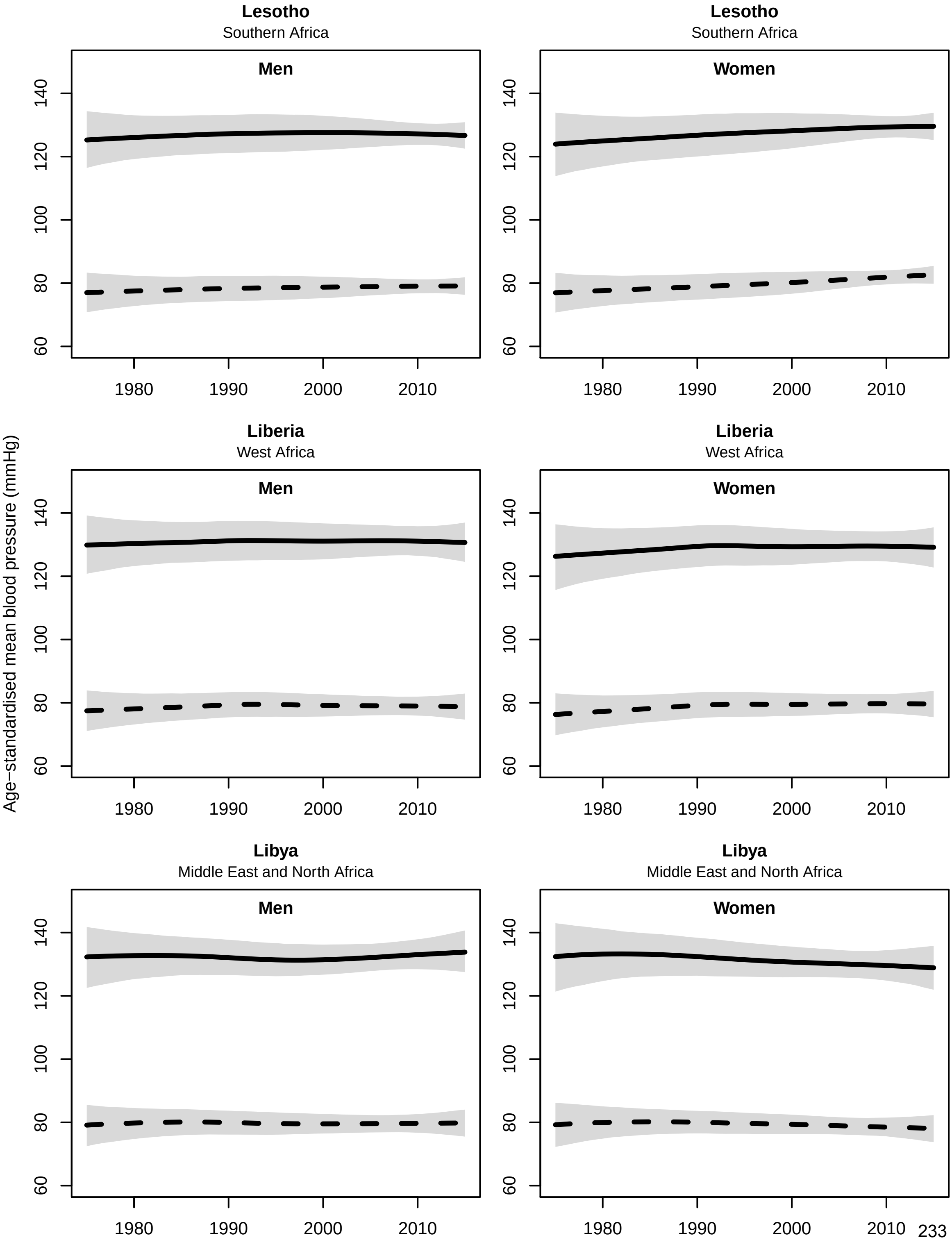
Lesotho
Southern Africa
Men
Lesotho
Southern Africa
Women
Liberia
West Africa
Men
Liberia
West Africa
Women
Libya
Middle East and North Africa
Men
Libya
Middle East and North Africa
Women
Age−standardised mean blood pressure (mmHg)
60
80
100
120
140
1980
1990
2000
2010

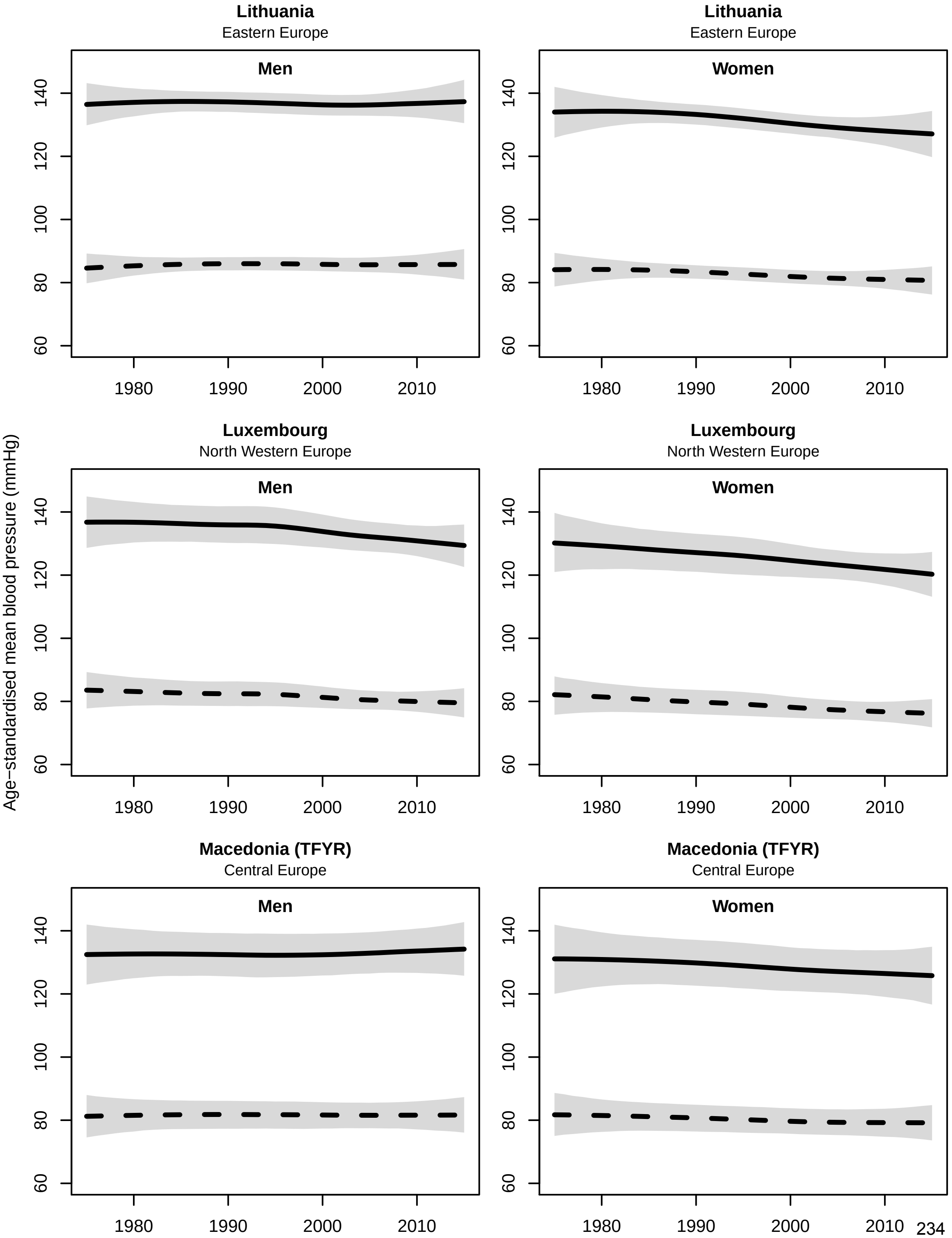
Lithuania
Eastern Europe
Men
Lithuania
Eastern Europe
Women
Luxembourg
North Western Europe
Men
Luxembourg
North Western Europe
Women
Macedonia (TFYR)
Central Europe
Men
Macedonia (TFYR)
Central Europe
Women
Age−standardised mean blood pressure (mmHg)
60
80
100
120
140
1980
1990
2000
2010

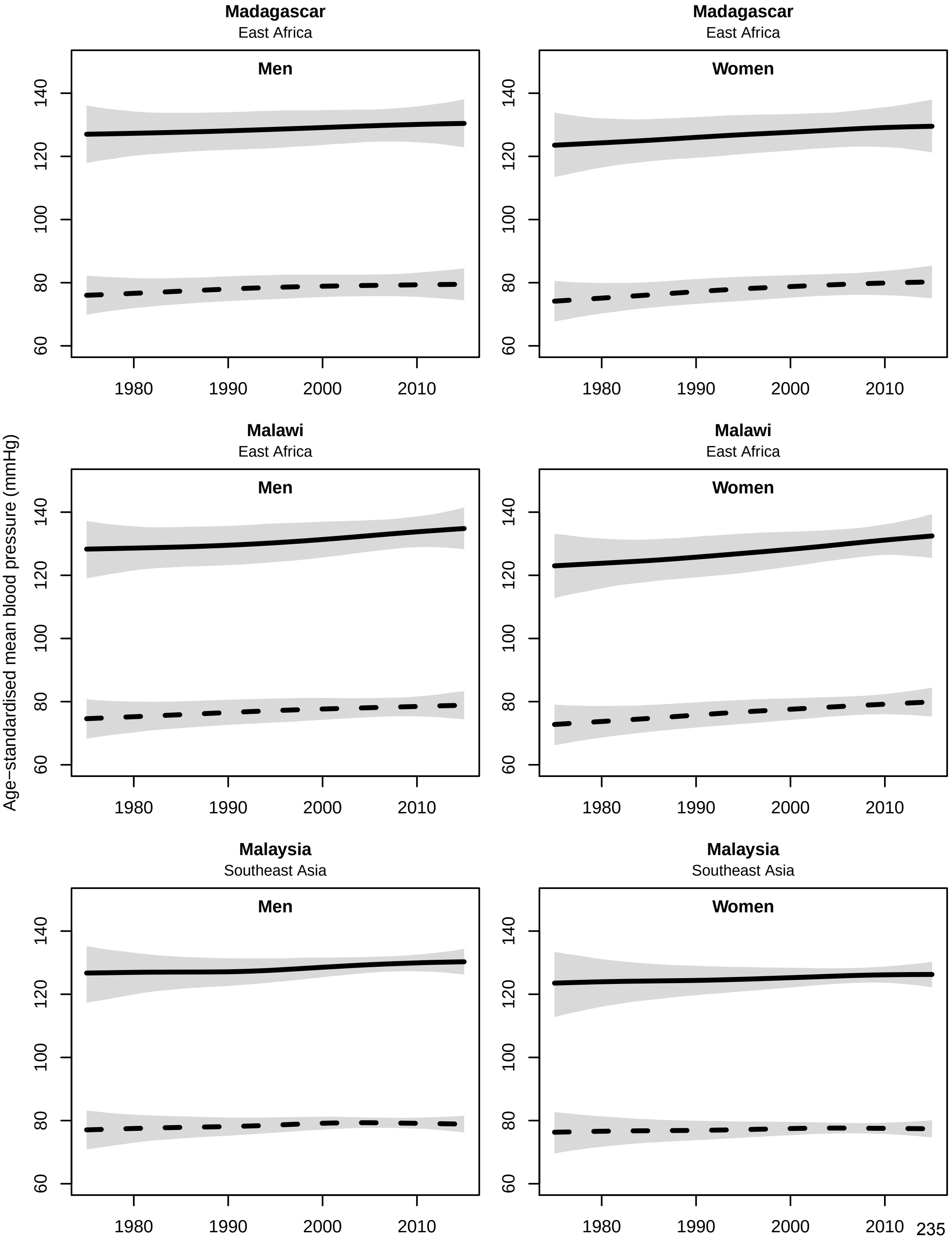

Madagascar
East Africa
Men
Madagascar
East Africa
Women
Malawi
East Africa
Men
Malawi
East Africa
Women
Malaysia
Southeast Asia
Men
Malaysia
Southeast Asia
Women
Age−standardised mean blood pressure (mmHg)
60
80
100
120
140
1980
1990
2000
2010

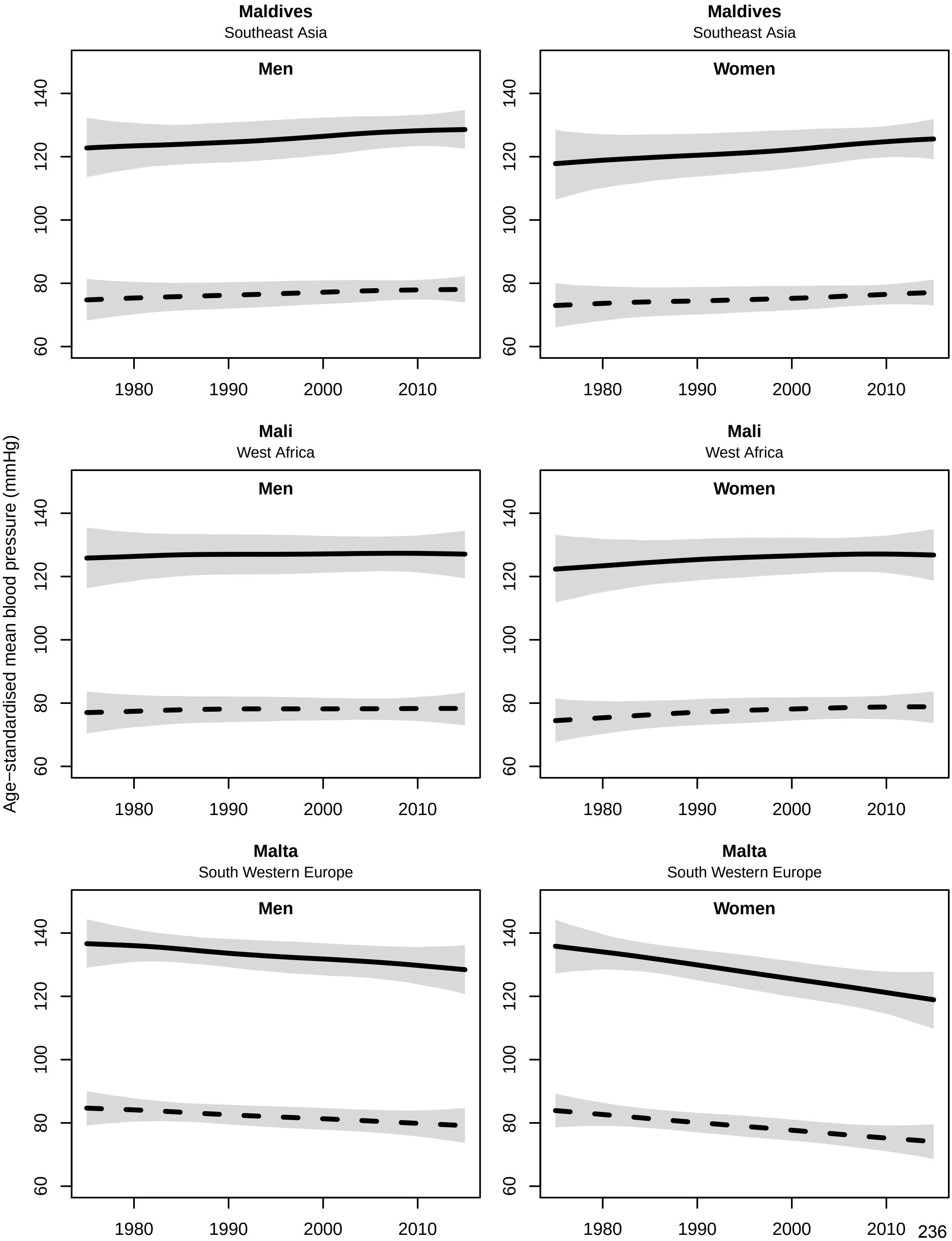
Maldives
Southeast Asia
Men
Maldives
Southeast Asia
Women
Mali
West Africa
Men
Mali
West Africa
Women
Malta
South Western Europe
Men
Malta
South Western Europe
Women
Age−standardised mean blood pressure (mmHg)
60
80
100
120
140
1980
1990
2000
2010

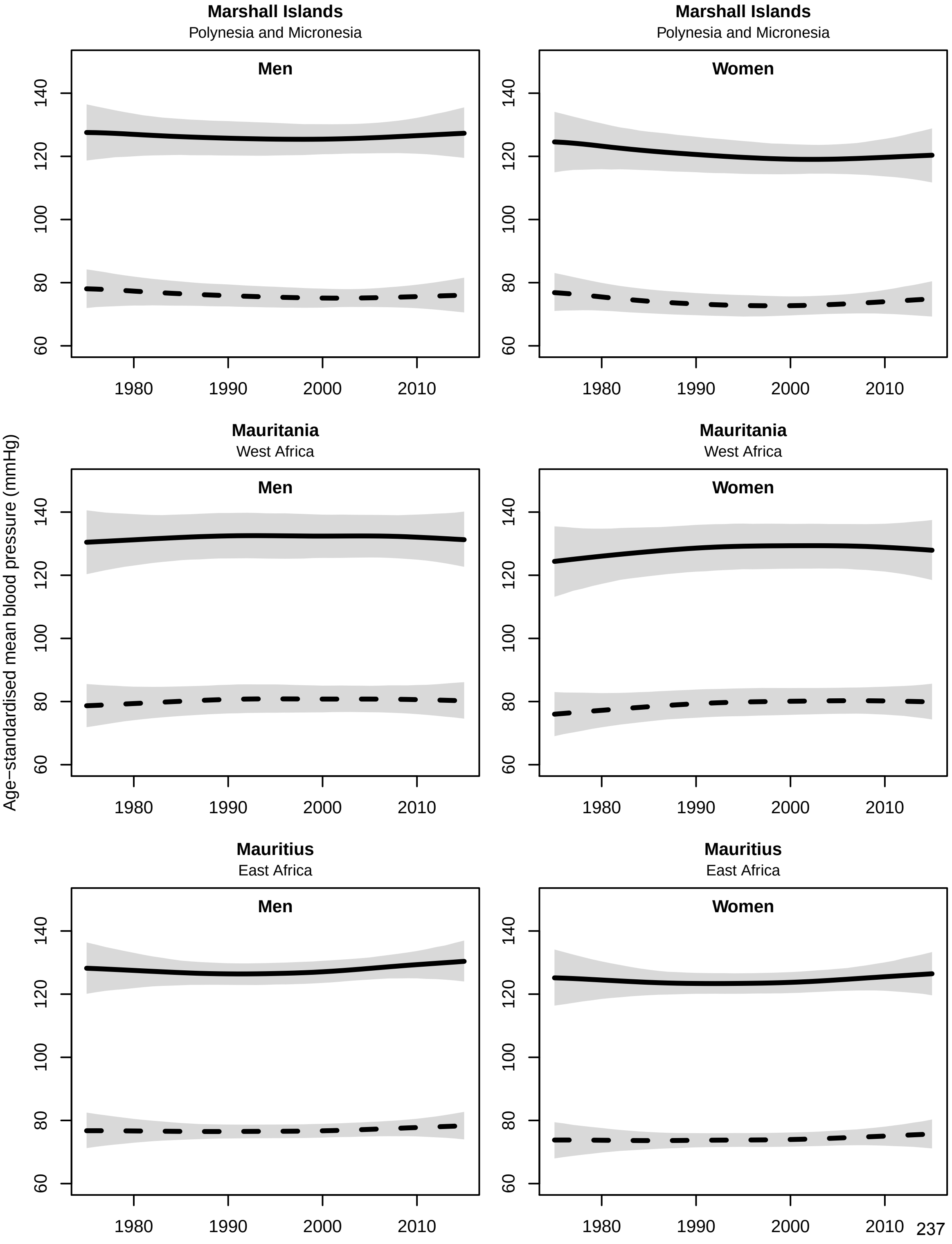
Marshall Islands
Polynesia and Micronesia
Men
Women
Mauritania
West Africa
Mauritius
East Africa
Age−standardised mean blood pressure (mmHg)
60
80
100
120
140
1980
1990
2000
2010

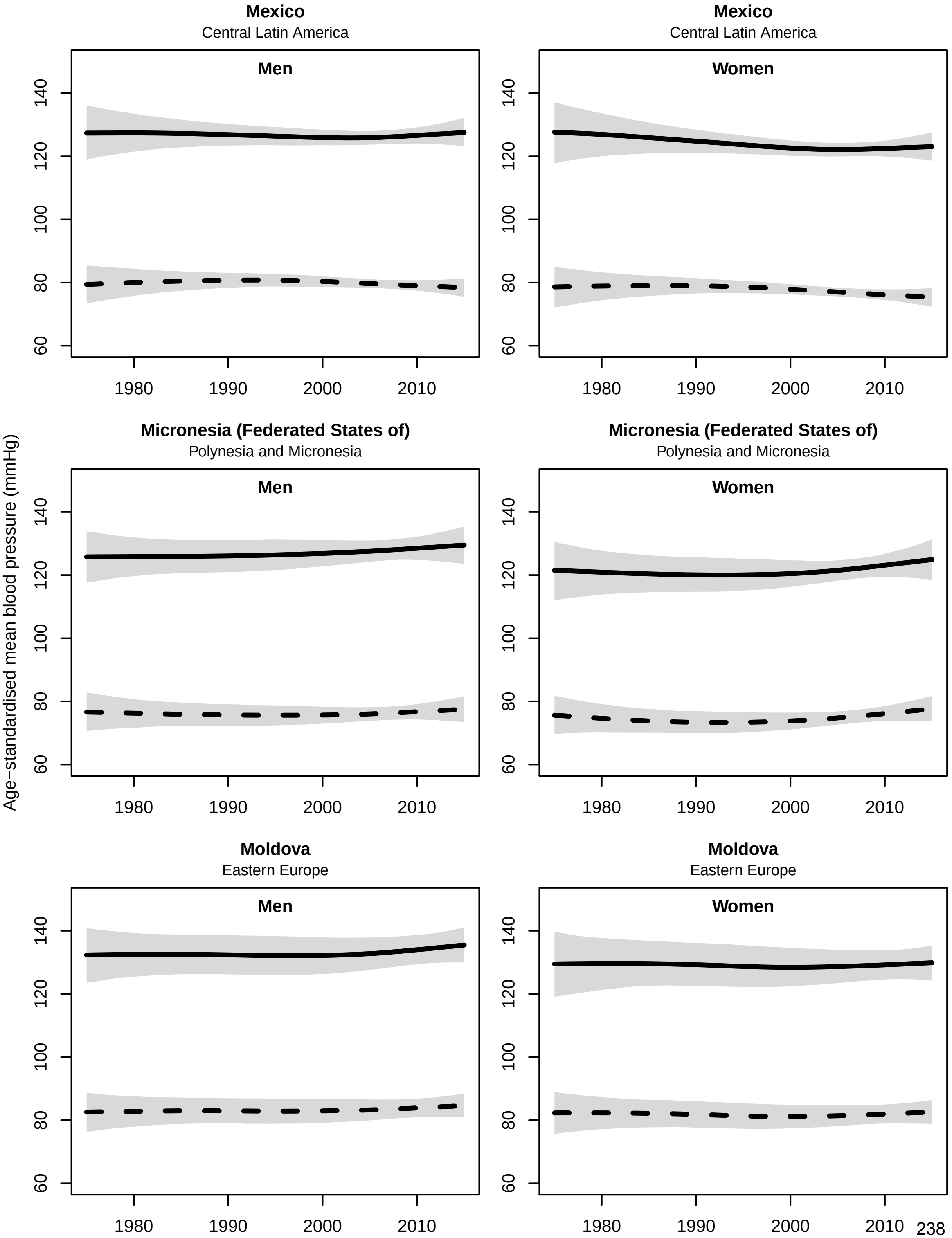
Mexico
Central Latin America
Men
Mexico
Central Latin America
Women
Micronesia (Federated States of)
Polynesia and Micronesia
Men
Micronesia (Federated States of)
Polynesia and Micronesia
Women
Moldova
Eastern Europe
Men
Moldova
Eastern Europe
Women
Age−standardised mean blood pressure (mmHg)
60
80
100
120
140
1980
1990
2000
2010

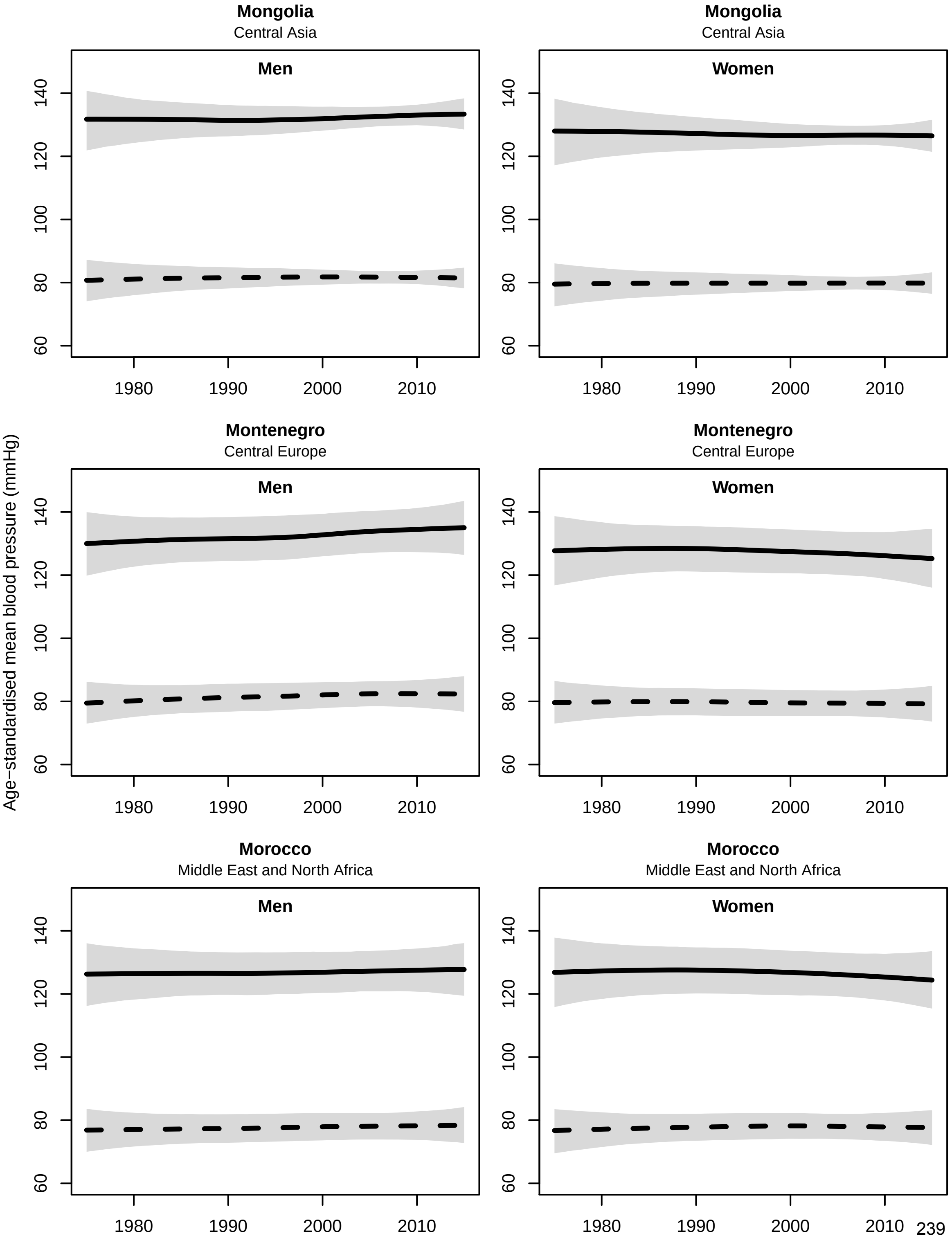

Mongolia
Central Asia
Men
Mongolia
Central Asia
Women
Montenegro
Central Europe
Men
Montenegro
Central Europe
Women
Morocco
Middle East and North Africa
Men
Morocco
Middle East and North Africa
Women
Age−standardised mean blood pressure (mmHg)
60
80
100
120
140
1980
1990
2000
2010

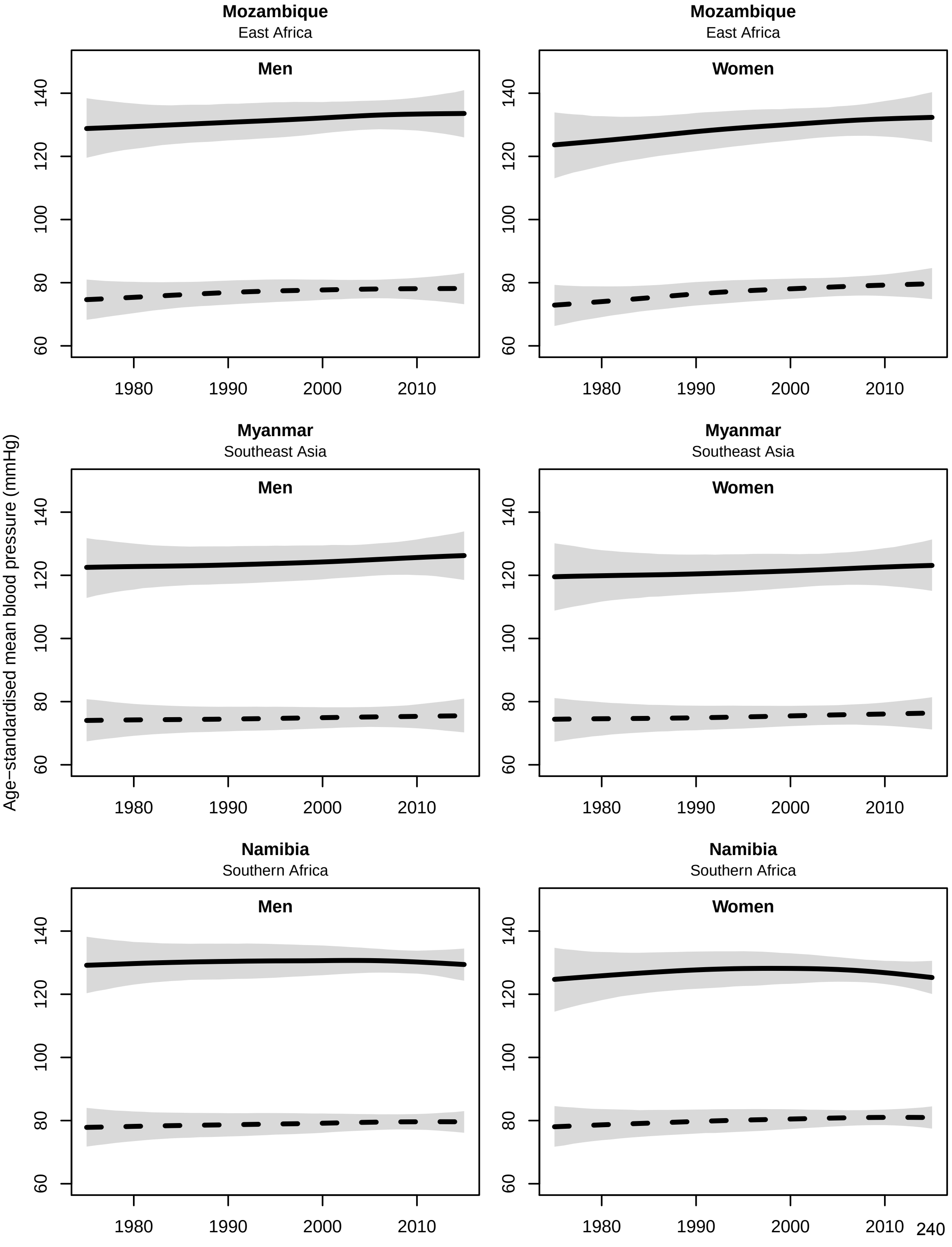
Mozambique
East Africa
Men
Mozambique
East Africa
Women
Myanmar
Southeast Asia
Men
Myanmar
Southeast Asia
Women
Namibia
Southern Africa
Men
Namibia
Southern Africa
Women
Age−standardised mean blood pressure (mmHg)
60
80
100
120
140
1980
1990
2000
2010

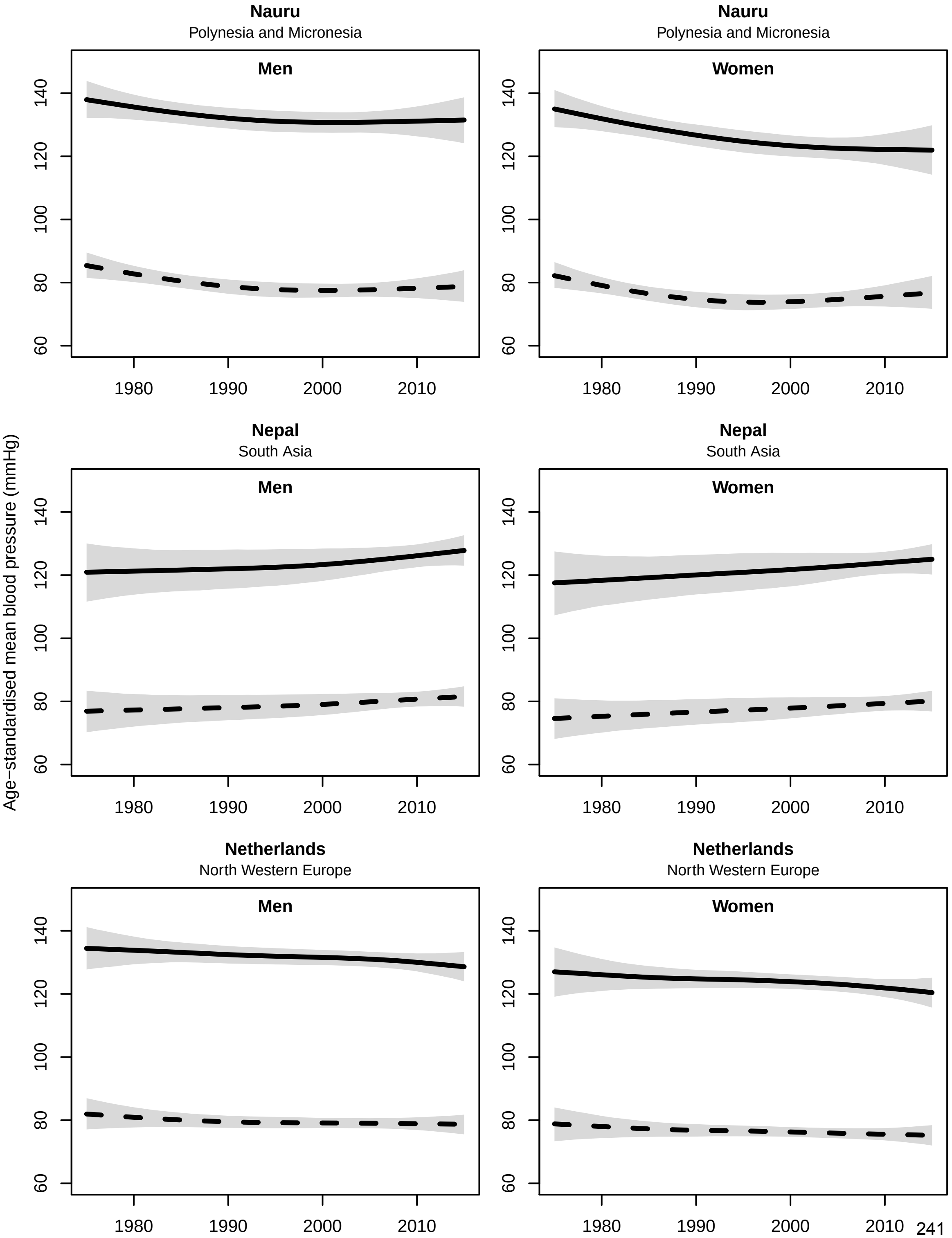
Nauru
Polynesia and Micronesia
Men
Women
Nepal
South Asia
Netherlands
North Western Europe
Age−standardised mean blood pressure (mmHg)
140
120
100
80
60
1980
1990
2000
2010

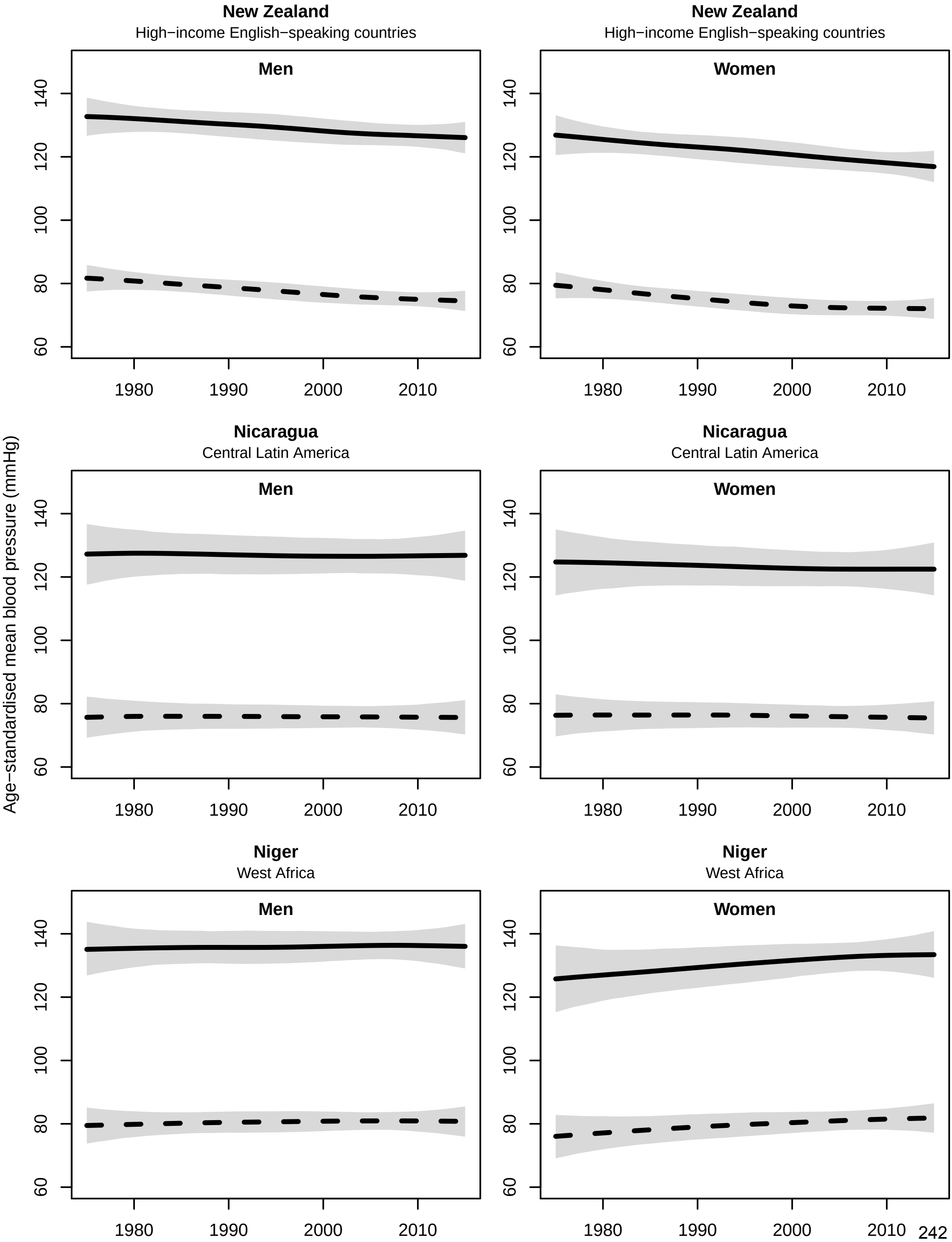
New Zealand
High−income English−speaking countries
Men
Women
Nicaragua
Central Latin America
Niger
West Africa
Age−standardised mean blood pressure (mmHg)
60
80
100
120
140
1980
1990
2000
2010

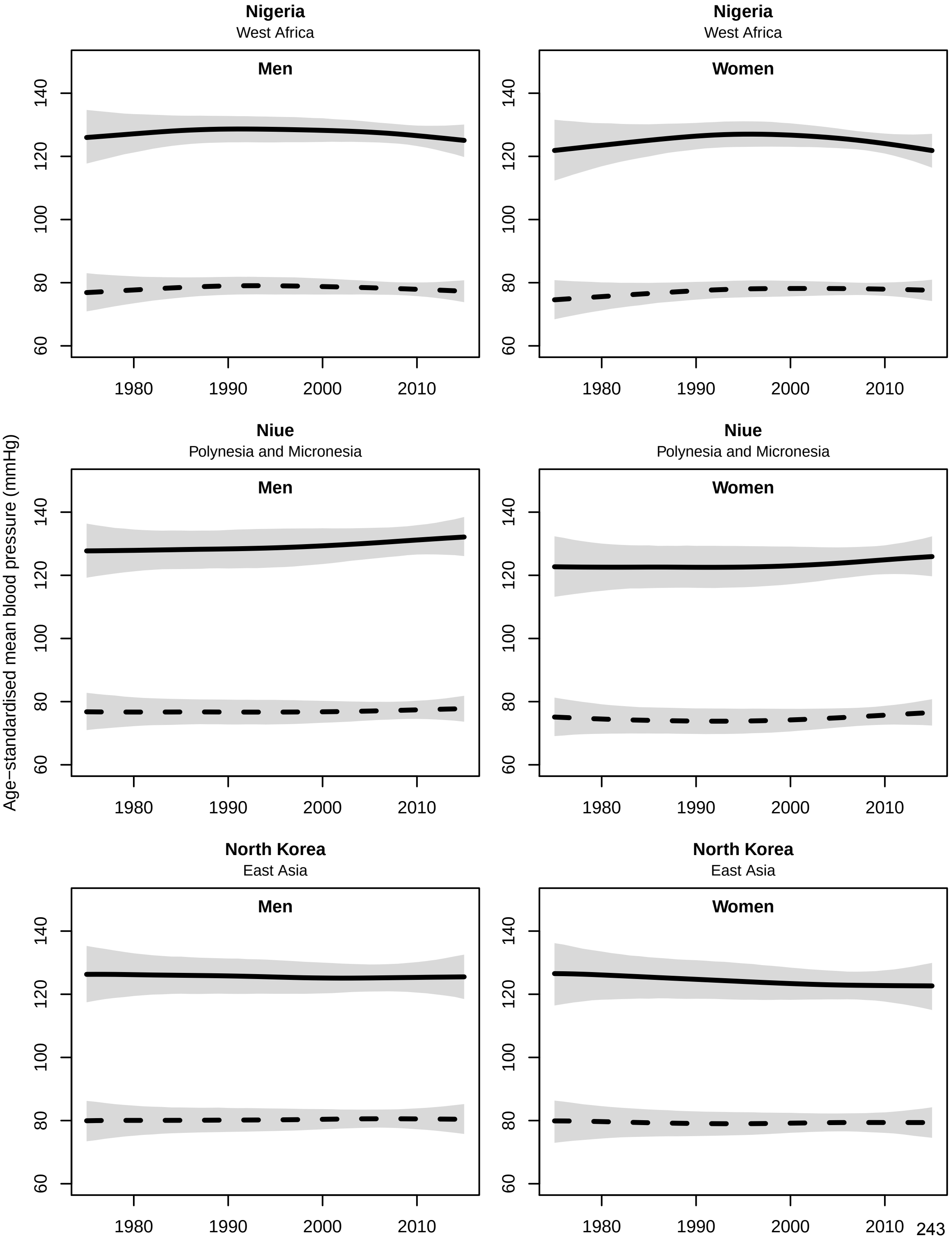
Nigeria
West Africa
Men
Nigeria
West Africa
Women
Niue
Polynesia and Micronesia
Men
Niue
Polynesia and Micronesia
Women
North Korea
East Asia
Men
North Korea
East Asia
Women
Age−standardised mean blood pressure (mmHg)
60
80
100
120
140
1980
1990
2000
2010

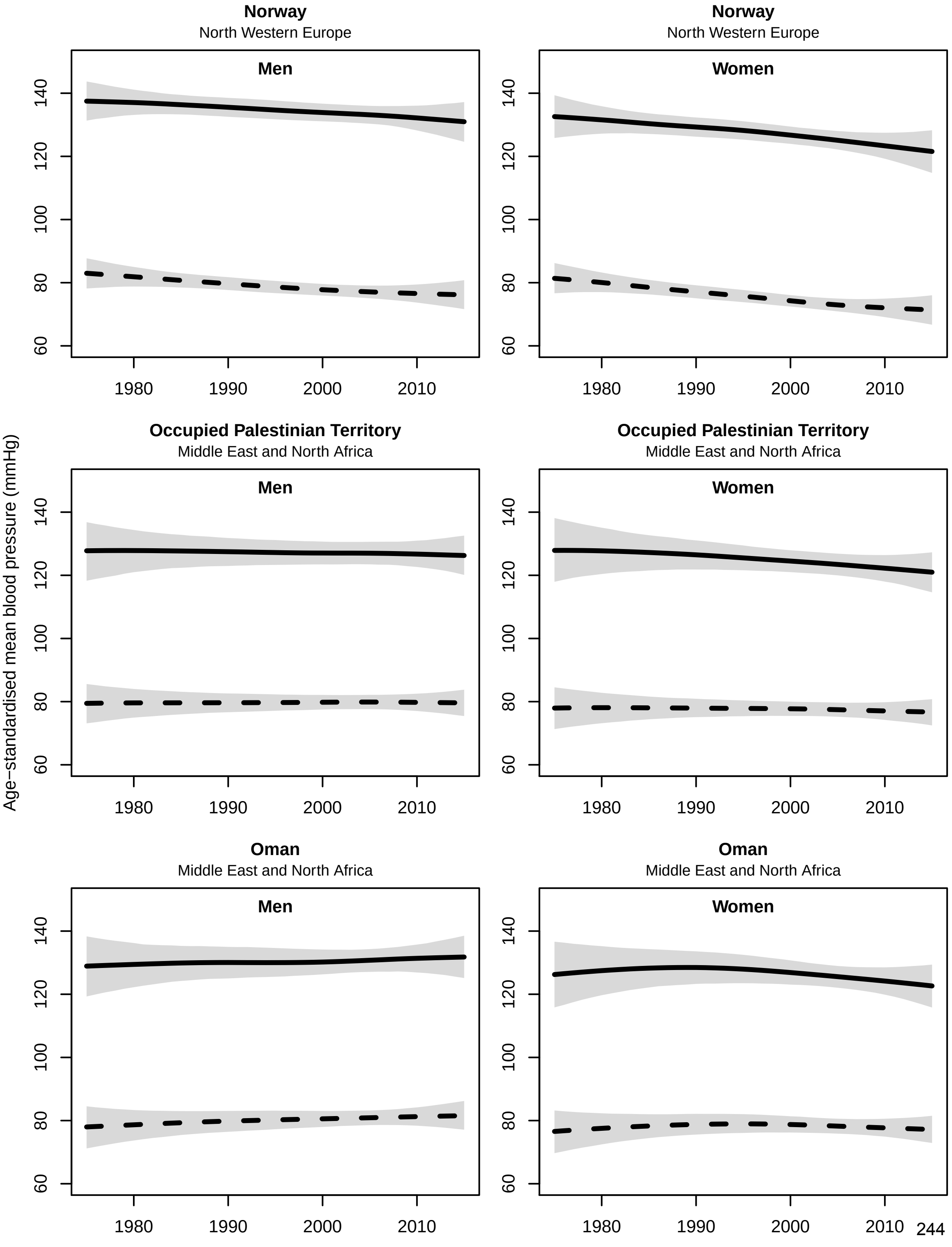
Norway
North Western Europe
Men
Norway
North Western Europe
Women
Occupied Palestinian Territory
Middle East and North Africa
Men
Occupied Palestinian Territory
Middle East and North Africa
Women
Oman
Middle East and North Africa
Men
Oman
Middle East and North Africa
Women
Age−standardised mean blood pressure (mmHg)
60
80
100
120
140
1980
1990
2000
2010

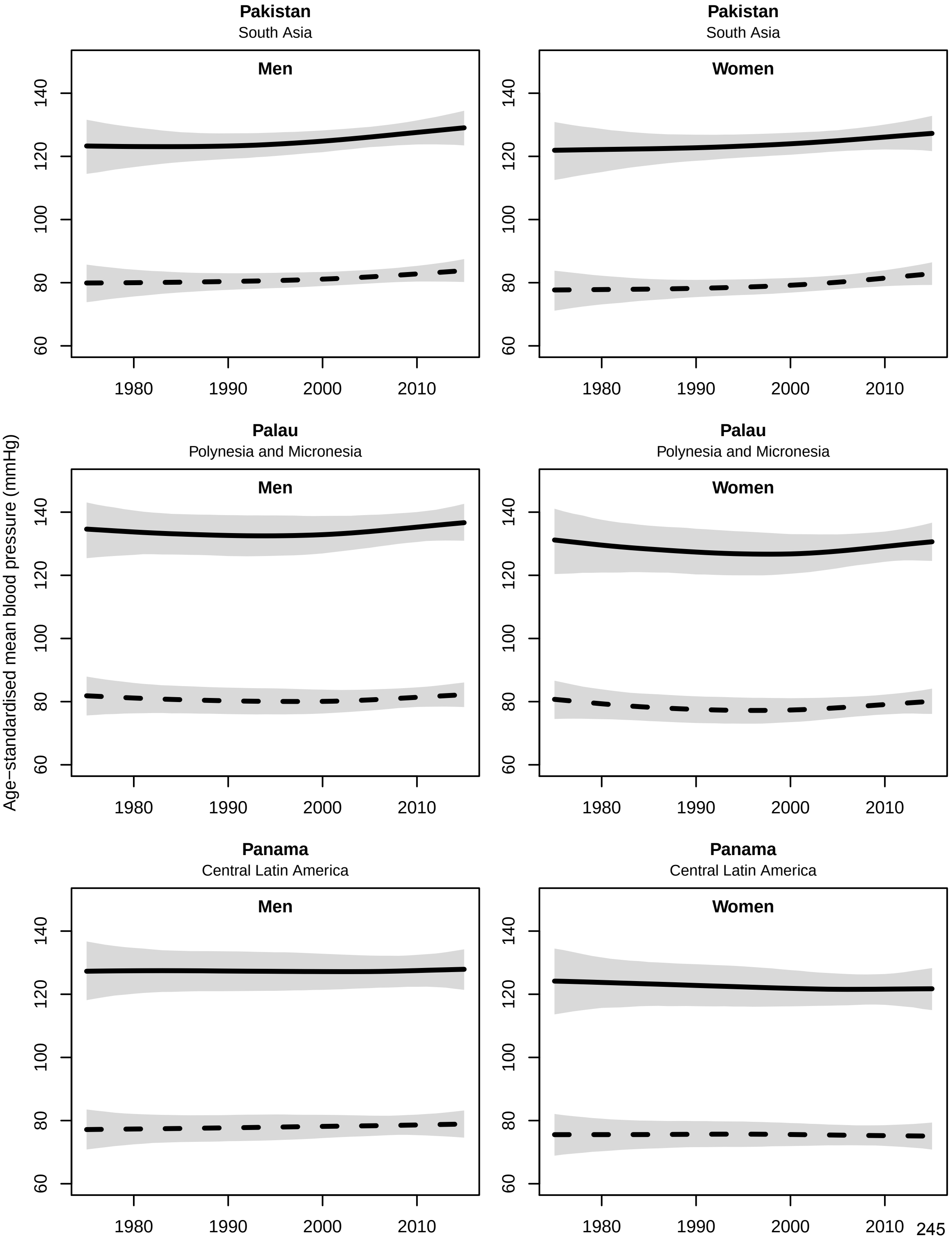
Pakistan
South Asia
Men
Pakistan
South Asia
Women
Palau
Polynesia and Micronesia
Men
Palau
Polynesia and Micronesia
Women
Panama
Central Latin America
Men
Panama
Central Latin America
Women
Age−standardised mean blood pressure (mmHg)
60
80
100
120
140
1980
1990
2000
2010

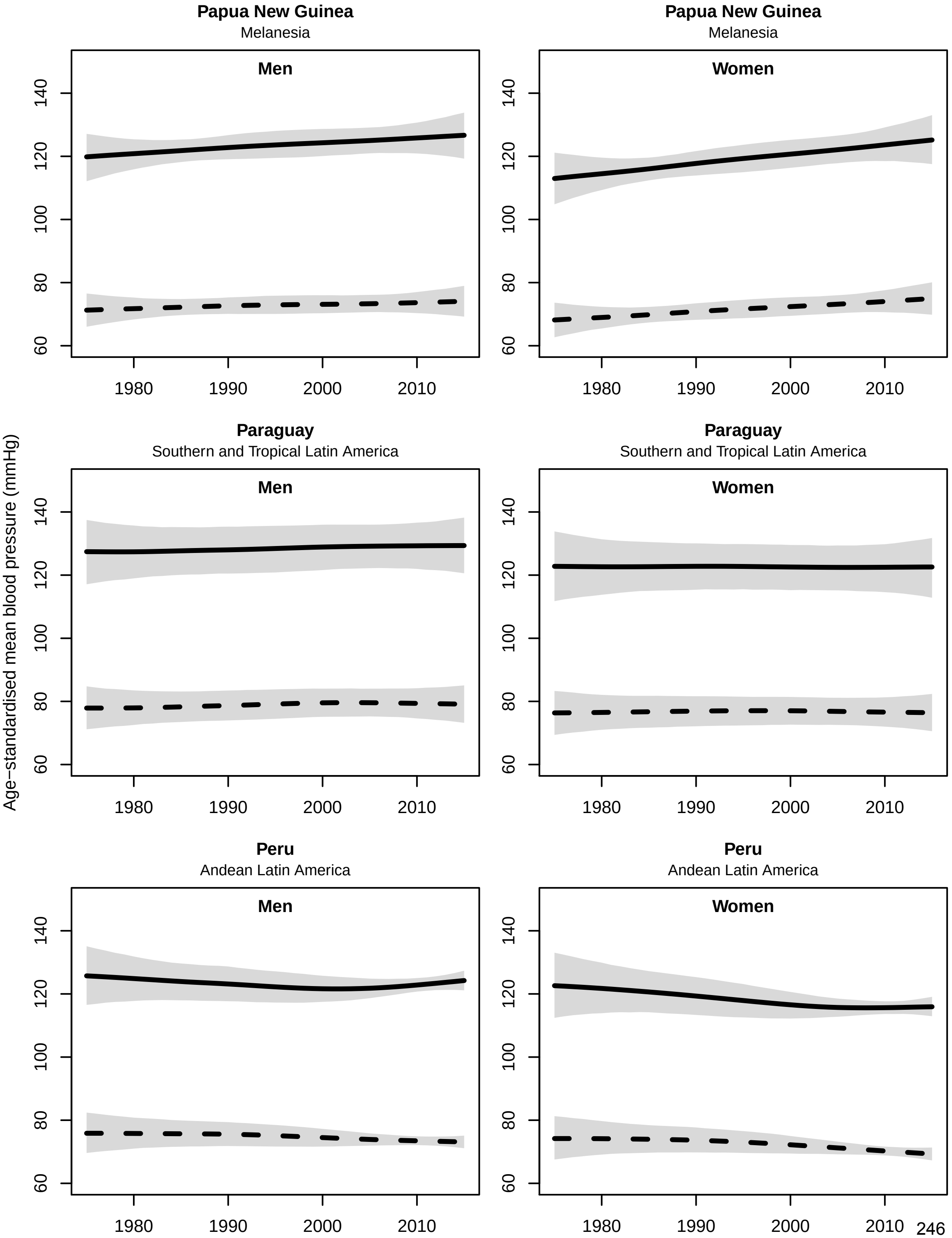
Papua New Guinea
Melanesia
Men
Women
Paraguay
Southern and Tropical Latin America
Peru
Andean Latin America
Age−standardised mean blood pressure (mmHg)
60
80
100
120
140
1980
1990
2000
2010

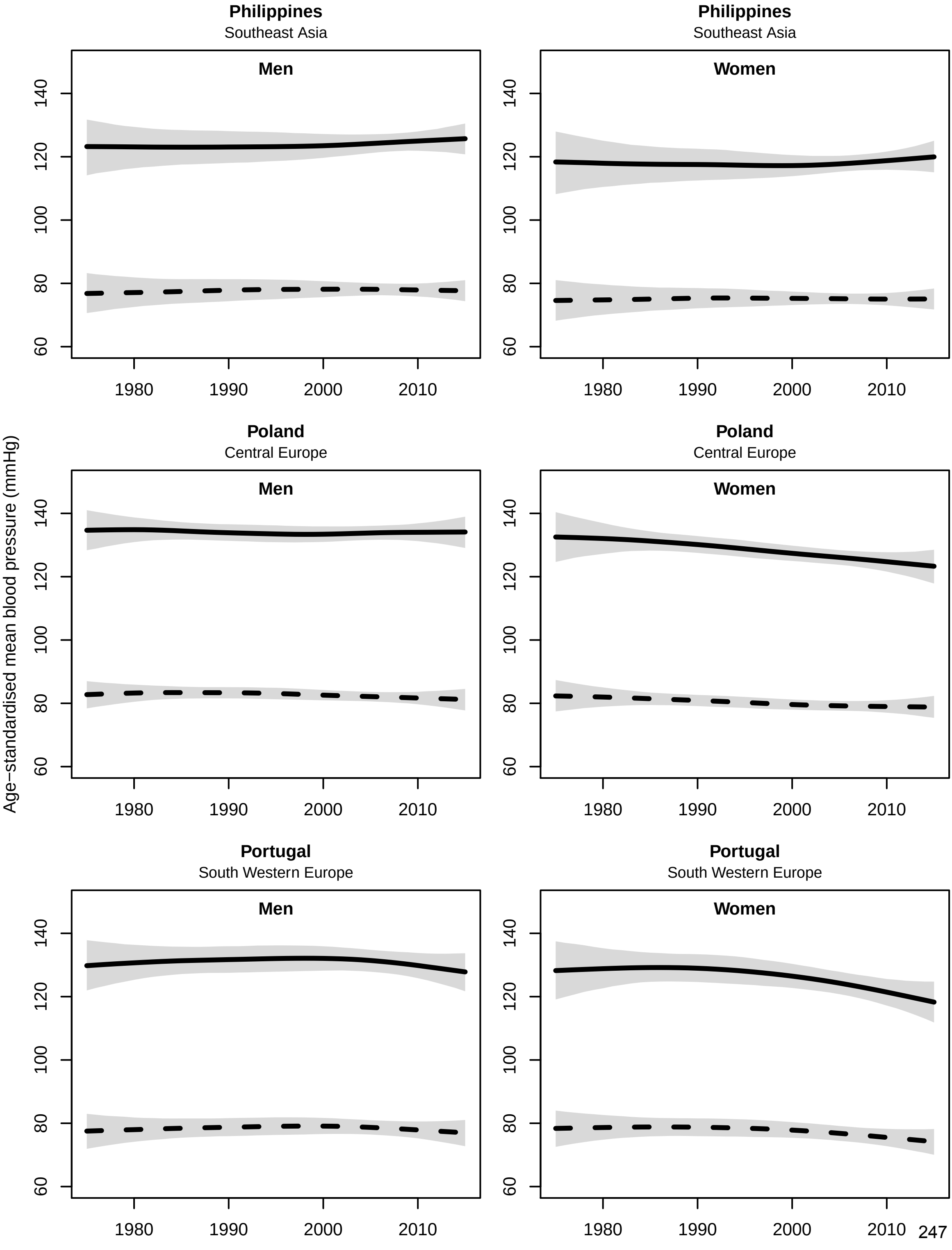
Philippines
Southeast Asia
Men
Philippines
Southeast Asia
Women
Poland
Central Europe
Men
Poland
Central Europe
Women
Portugal
South Western Europe
Men
Portugal
South Western Europe
Women
Age−standardised mean blood pressure (mmHg)
60
80
100
120
140
1980
1990
2000
2010

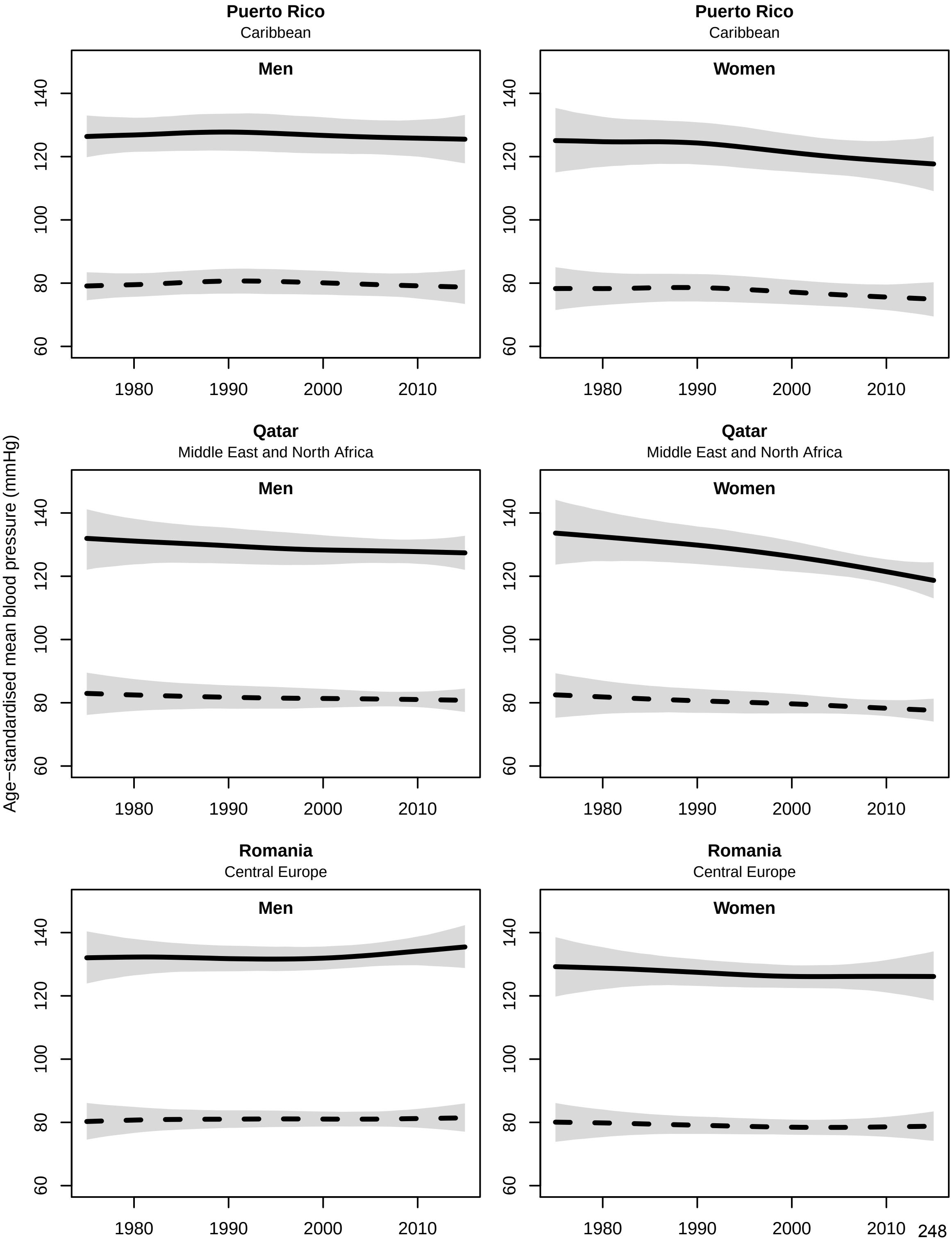
Puerto Rico
Caribbean
Men
Puerto Rico
Caribbean
Women
Qatar
Middle East and North Africa
Men
Qatar
Middle East and North Africa
Women
Romania
Central Europe
Men
Romania
Central Europe
Women
Age−standardised mean blood pressure (mmHg)
60
80
100
120
140
1980
1990
2000
2010

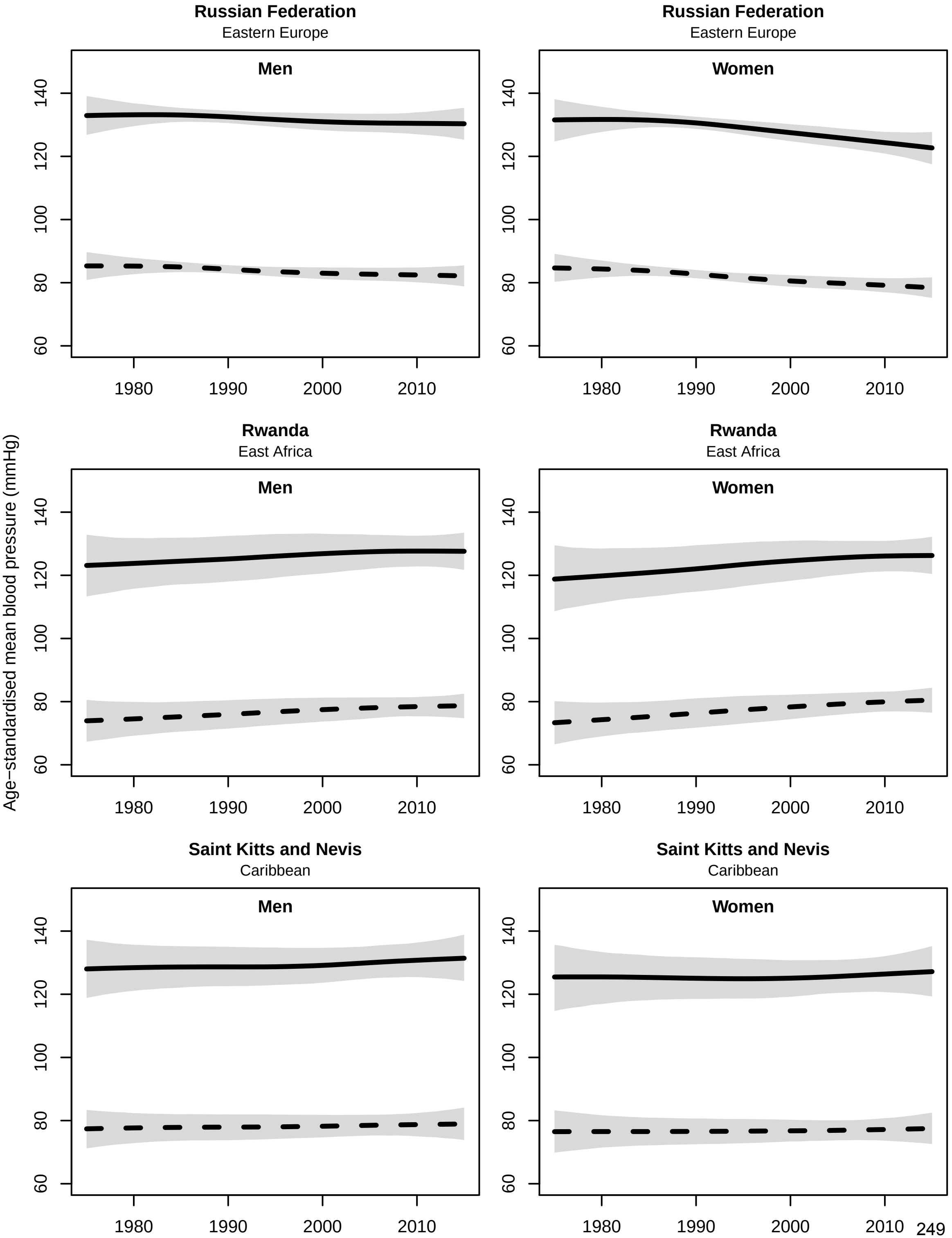
Russian Federation
Eastern Europe
Men
Russian Federation
Eastern Europe
Women
Rwanda
East Africa
Men
Rwanda
East Africa
Women
Saint Kitts and Nevis
Caribbean
Men
Saint Kitts and Nevis
Caribbean
Women
Age−standardised mean blood pressure (mmHg)
60
80
100
120
140
1980
1990
2000
2010

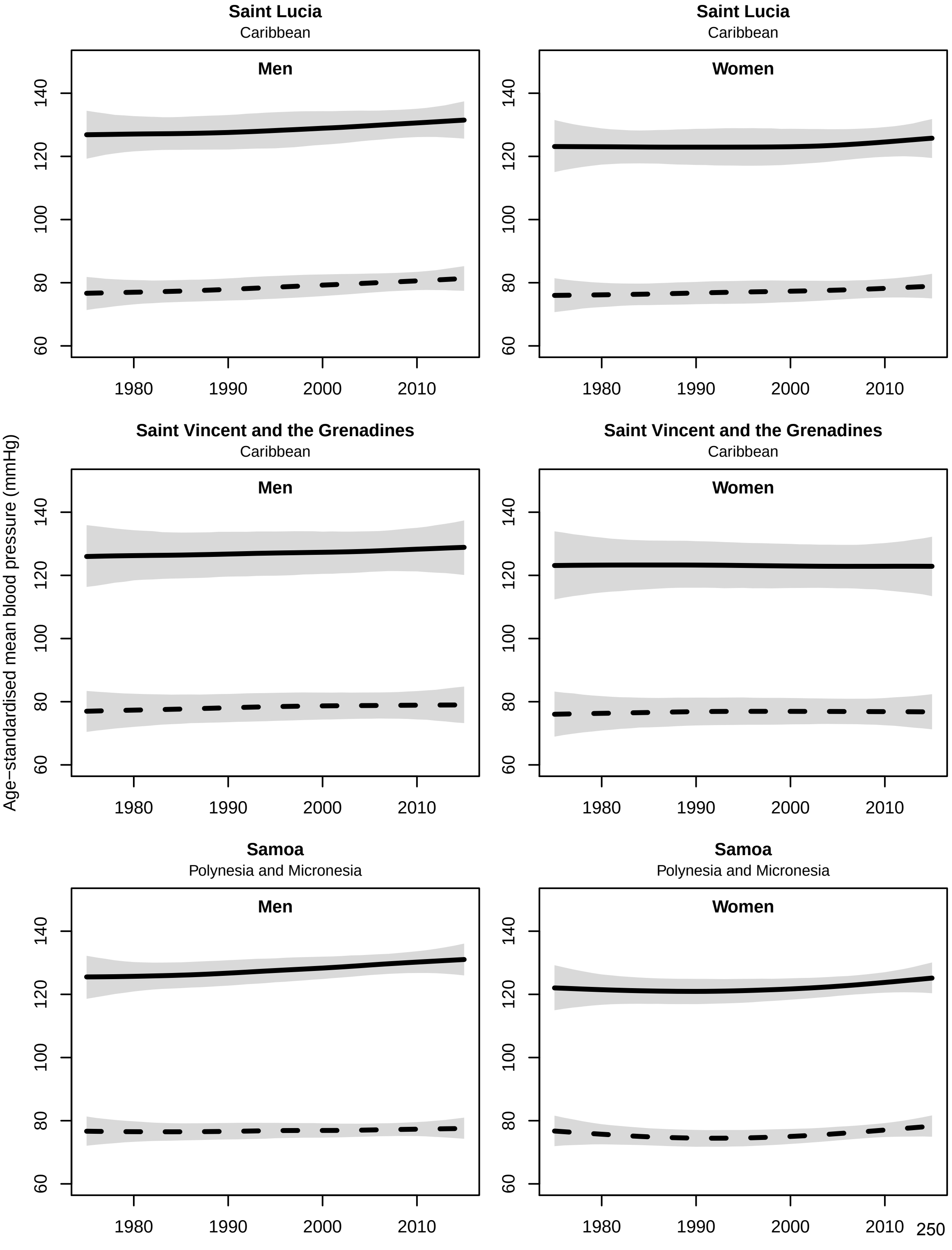

Saint Lucia
Caribbean
Men
Saint Lucia
Caribbean
Women
Saint Vincent and the Grenadines
Caribbean
Men
Saint Vincent and the Grenadines
Caribbean
Women
Samoa
Polynesia and Micronesia
Men
Samoa
Polynesia and Micronesia
Women
Age−standardised mean blood pressure (mmHg)
60
80
100
120
140
1980
1990
2000
2010

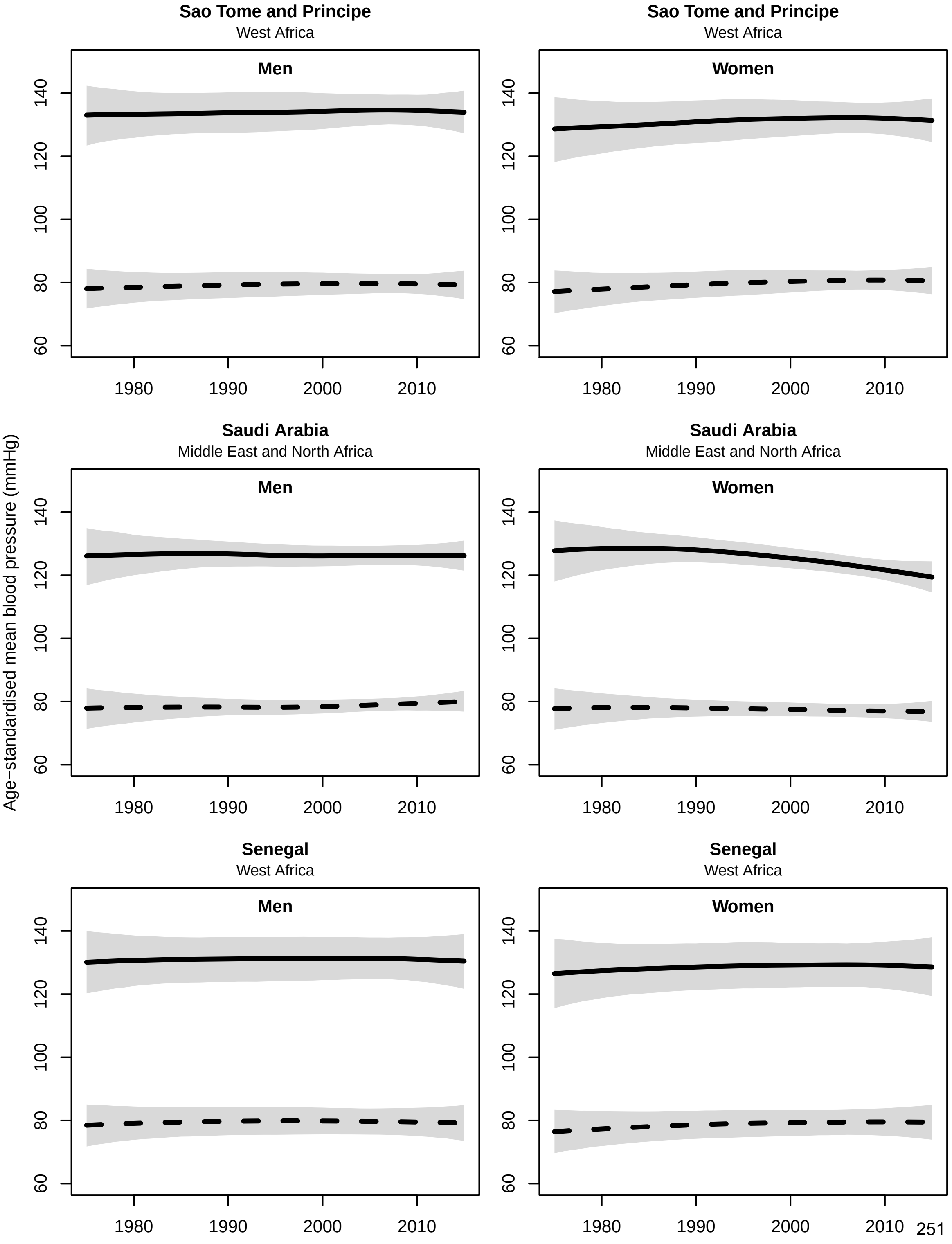
Sao Tome and Principe
West Africa
Men
Sao Tome and Principe
West Africa
Women
Saudi Arabia
Middle East and North Africa
Men
Saudi Arabia
Middle East and North Africa
Women
Senegal
West Africa
Men
Senegal
West Africa
Women
Age−standardised mean blood pressure (mmHg)
60
80
100
120
140
1980
1990
2000
2010

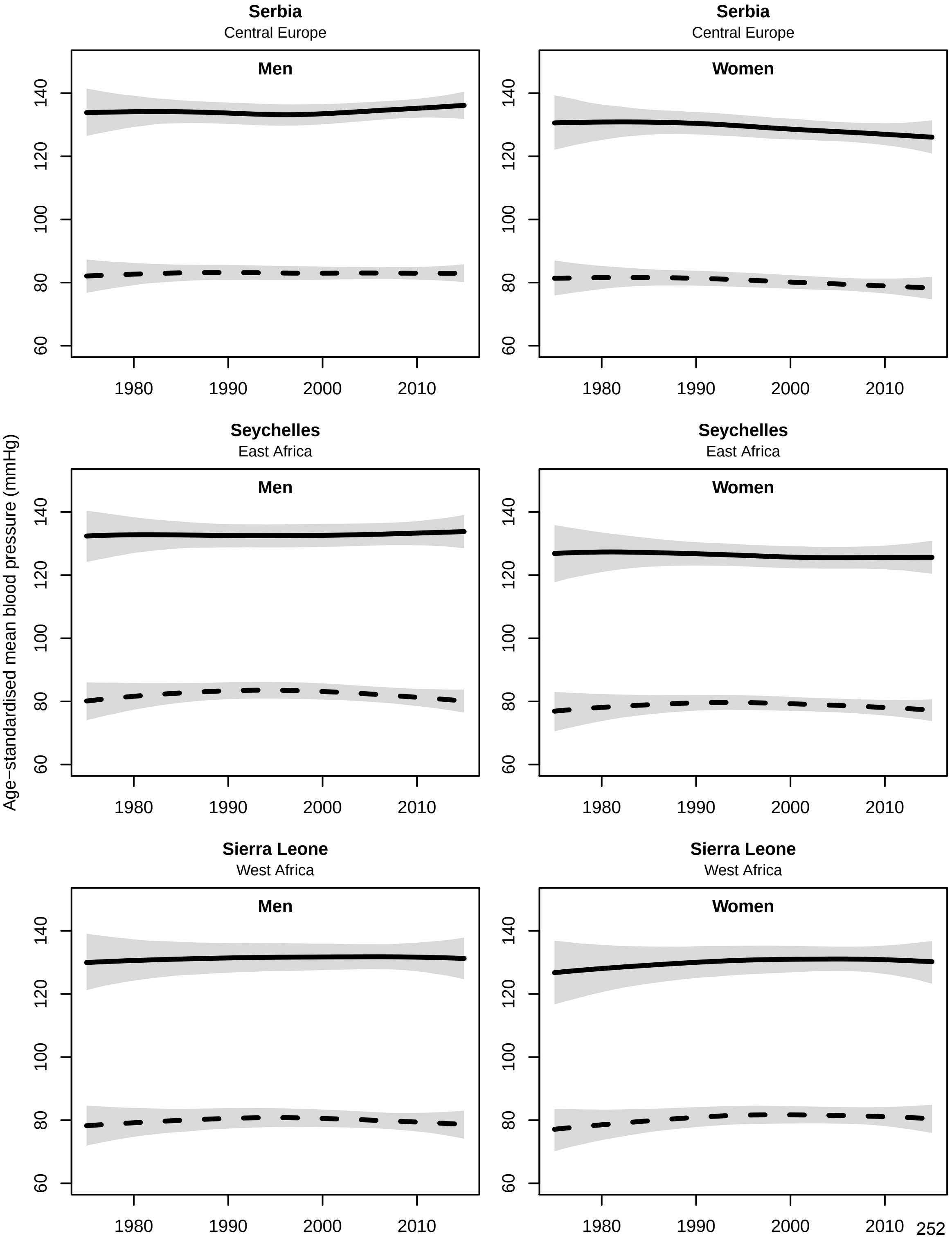
Serbia
Central Europe
Men
Serbia
Central Europe
Women
Seychelles
East Africa
Men
Seychelles
East Africa
Women
Sierra Leone
West Africa
Men
Sierra Leone
West Africa
Women
Age−standardised mean blood pressure (mmHg)
60
80
100
120
140
1980
1990
2000
2010

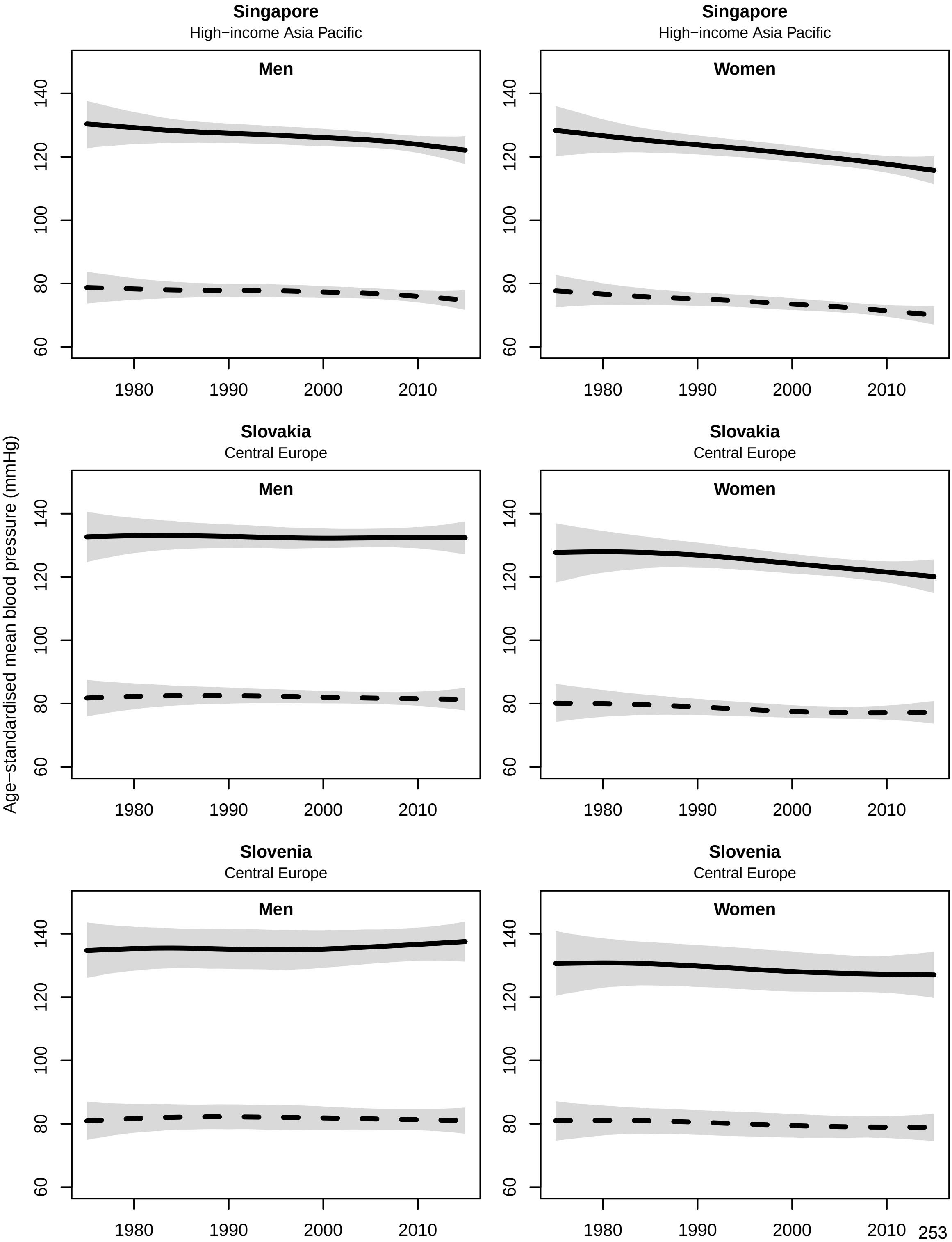
Singapore
High−income Asia Pacific
Men
Singapore
High−income Asia Pacific
Women
Slovakia
Central Europe
Men
Slovakia
Central Europe
Women
Slovenia
Central Europe
Men
Slovenia
Central Europe
Women
Age−standardised mean blood pressure (mmHg)
60
80
100
120
140
1980
1990
2000
2010

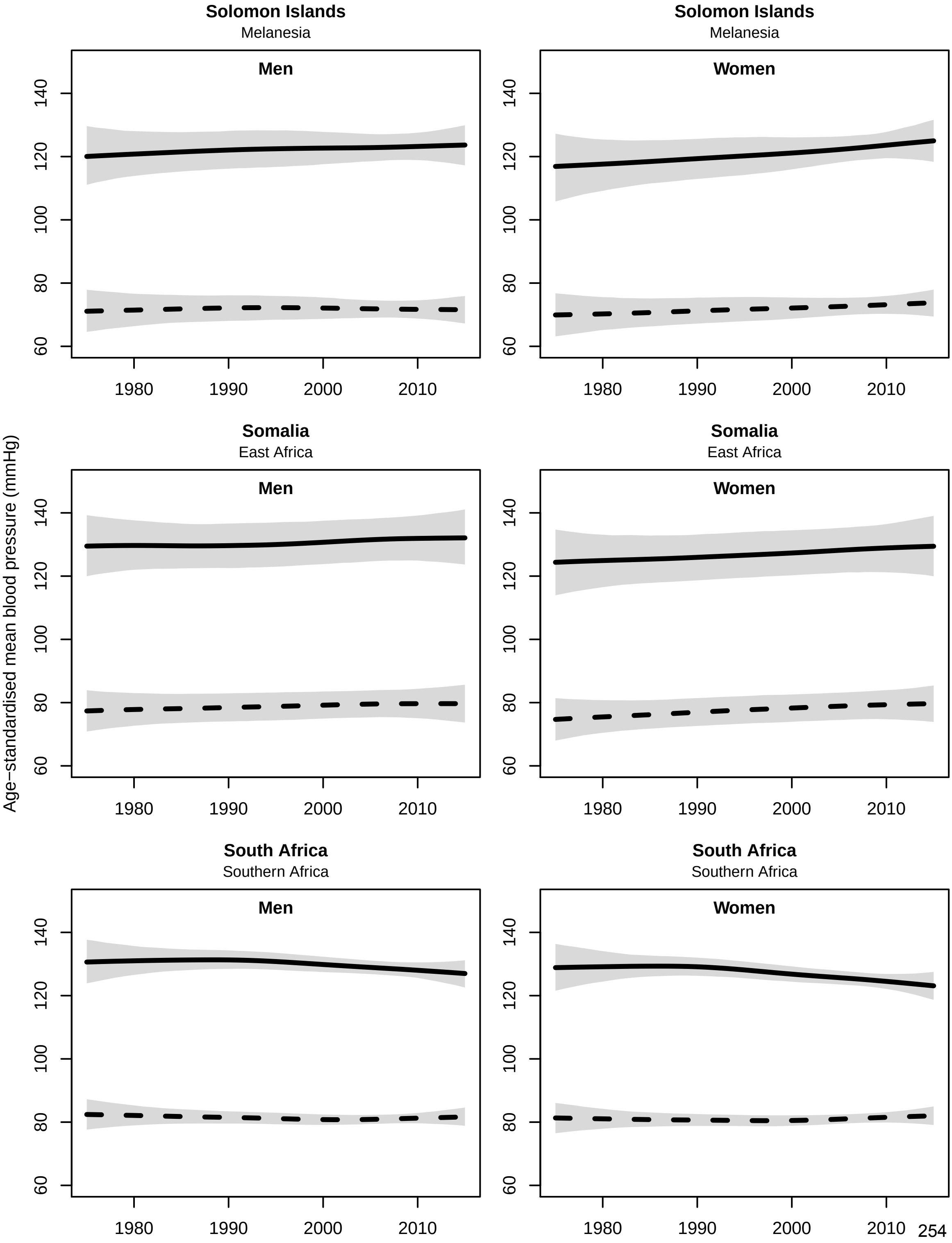

Solomon Islands
Melanesia
Men
Women
Somalia
East Africa
South Africa
Southern Africa
Age−standardised mean blood pressure (mmHg)
60
80
100
120
140
1980
1990
2000
2010

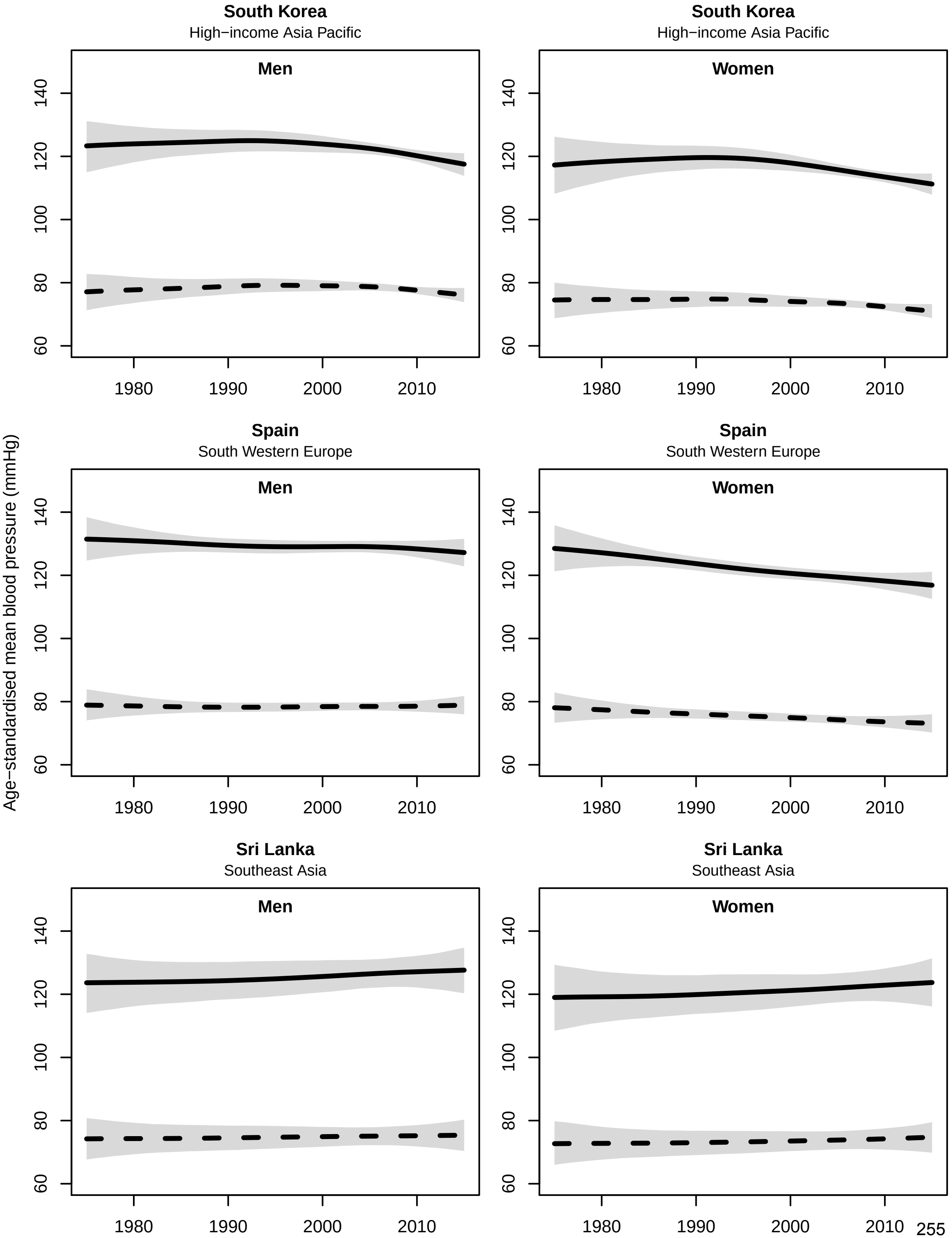
South Korea
High−income Asia Pacific
Men
South Korea
High−income Asia Pacific
Women
Spain
South Western Europe
Men
Spain
South Western Europe
Women
Sri Lanka
Southeast Asia
Men
Sri Lanka
Southeast Asia
Women
Age−standardised mean blood pressure (mmHg)
60
80
100
120
140
1980
1990
2000
2010

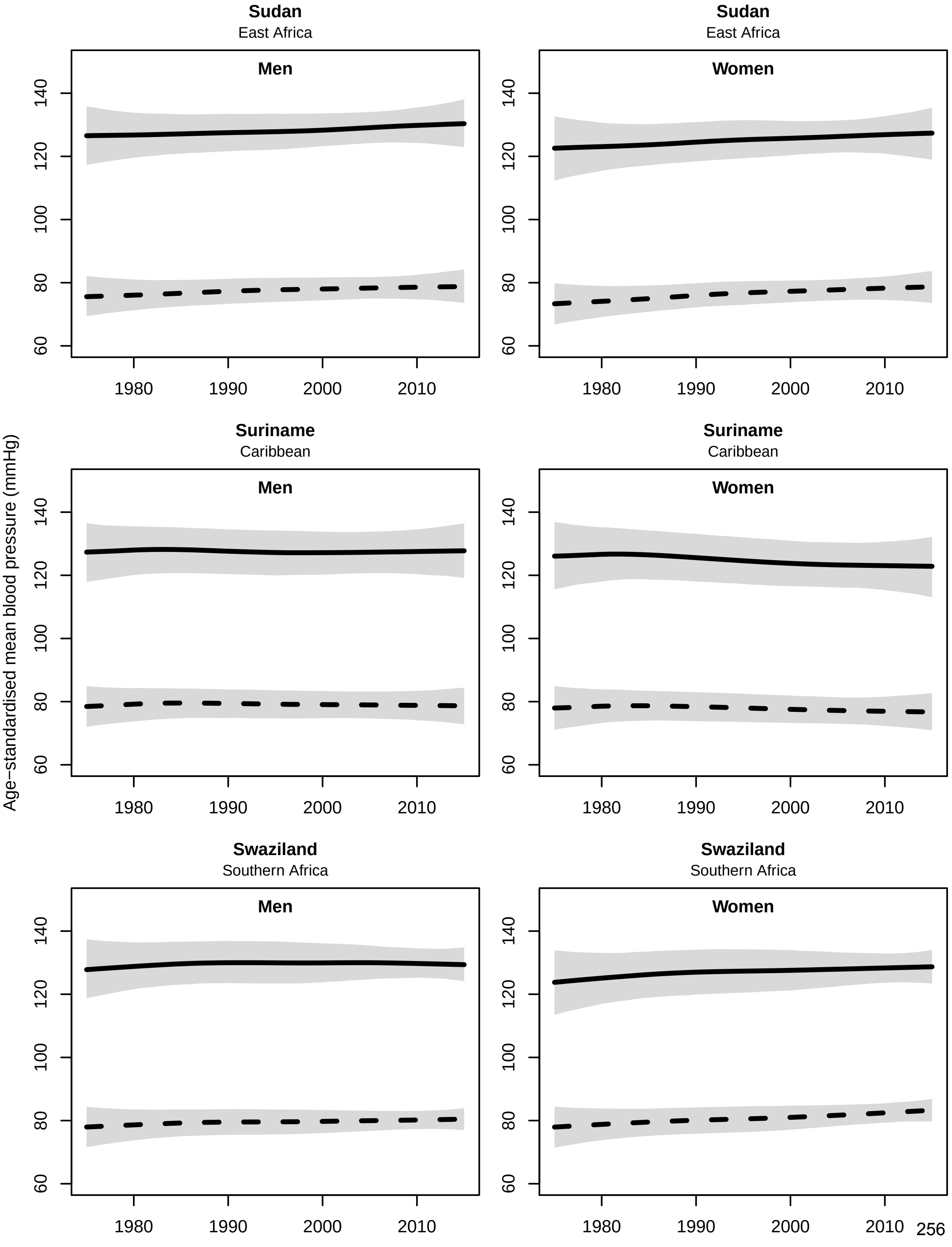
Sudan
East Africa
Men
Sudan
East Africa
Women
Suriname
Caribbean
Men
Suriname
Caribbean
Women
Swaziland
Southern Africa
Men
Swaziland
Southern Africa
Women
Age−standardised mean blood pressure (mmHg)
60
80
100
120
140
1980
1990
2000
2010

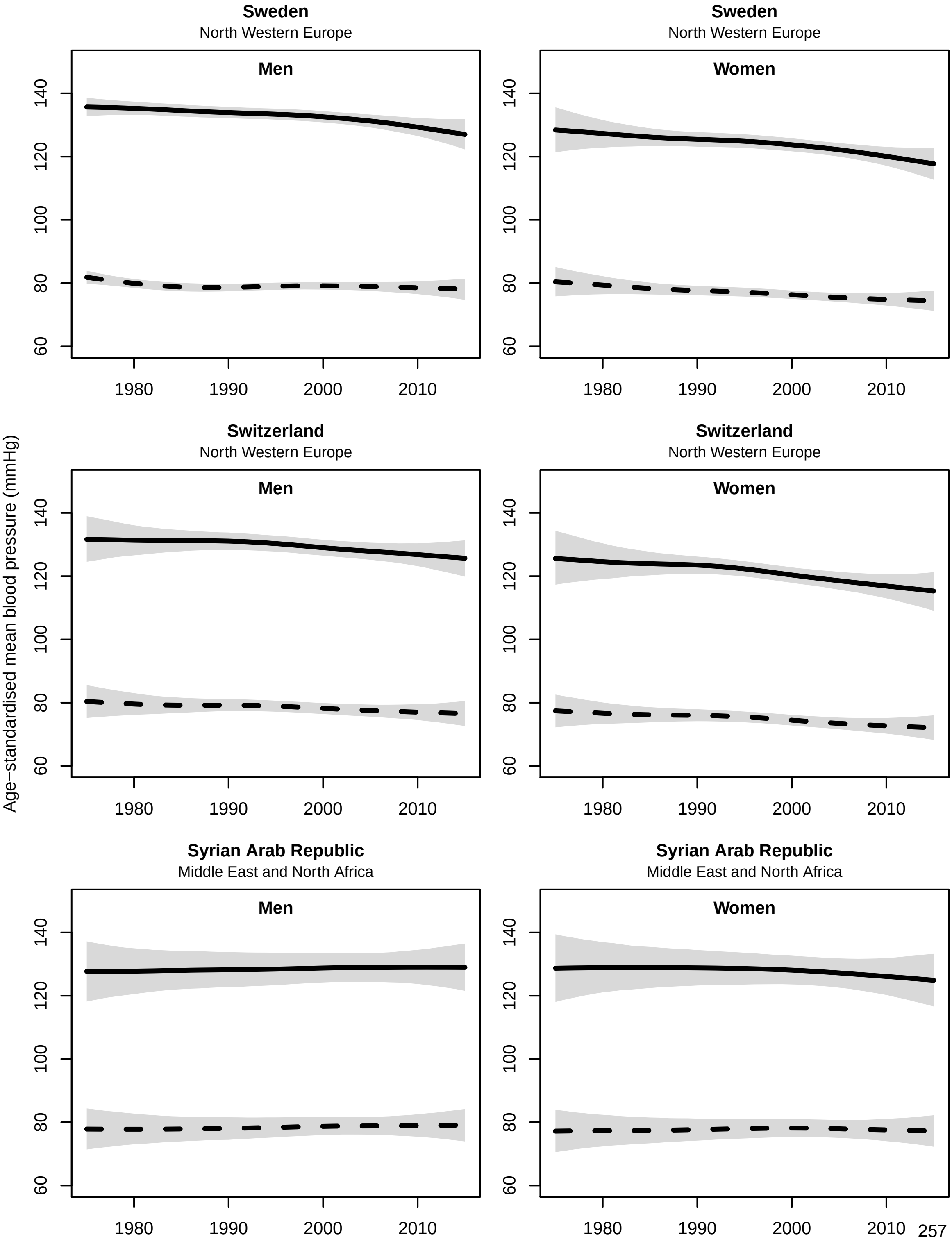
Sweden
North Western Europe
Men
Sweden
North Western Europe
Women
Switzerland
North Western Europe
Men
Switzerland
North Western Europe
Women
Syrian Arab Republic
Middle East and North Africa
Men
Syrian Arab Republic
Middle East and North Africa
Women
Age−standardised mean blood pressure (mmHg)
60
80
100
120
140
1980
1990
2000
2010

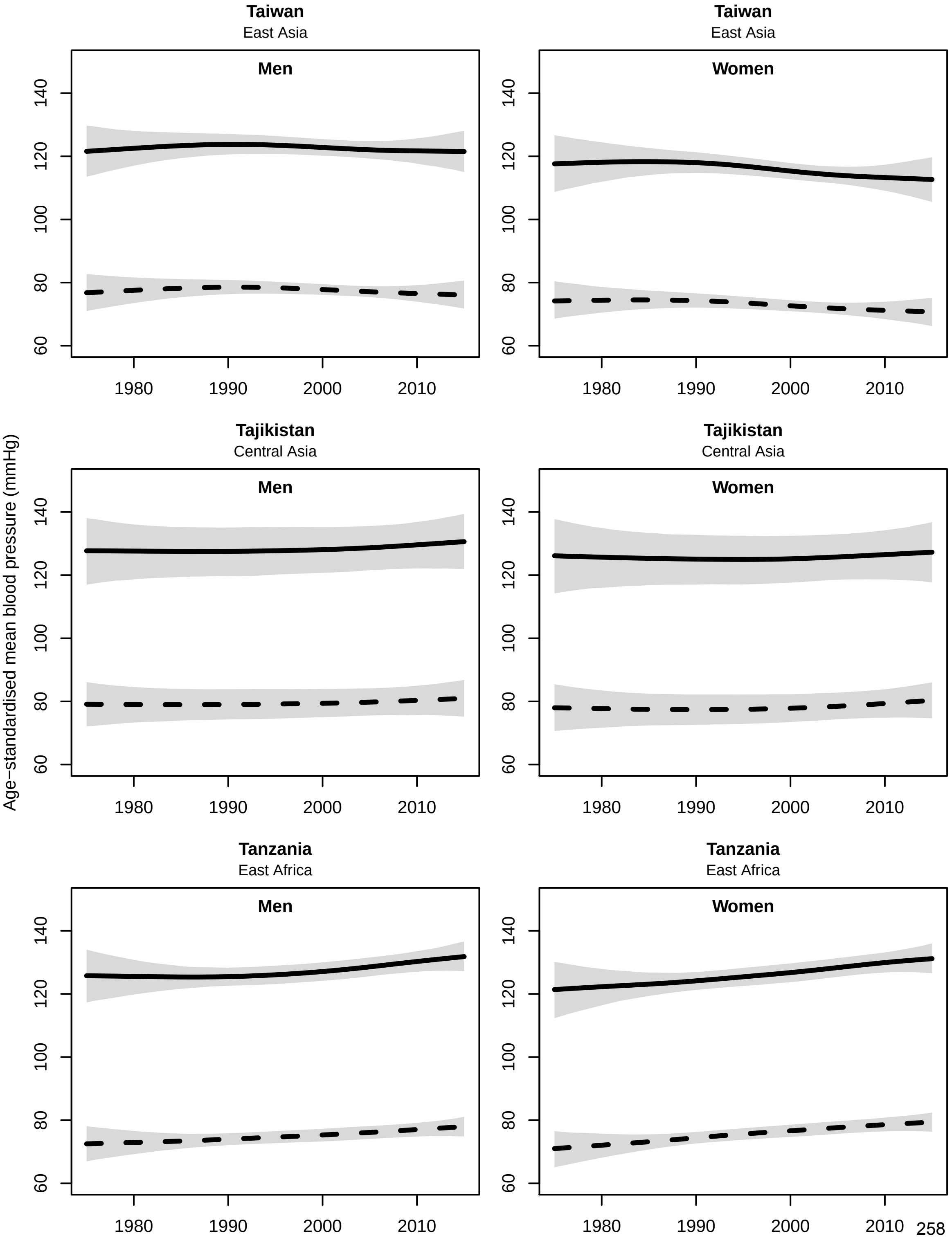
Taiwan
East Asia
Men
Taiwan
East Asia
Women
Tajikistan
Central Asia
Men
Tajikistan
Central Asia
Women
Tanzania
East Africa
Men
Tanzania
East Africa
Women
Age−standardised mean blood pressure (mmHg)
60
80
100
120
140
1980
1990
2000
2010

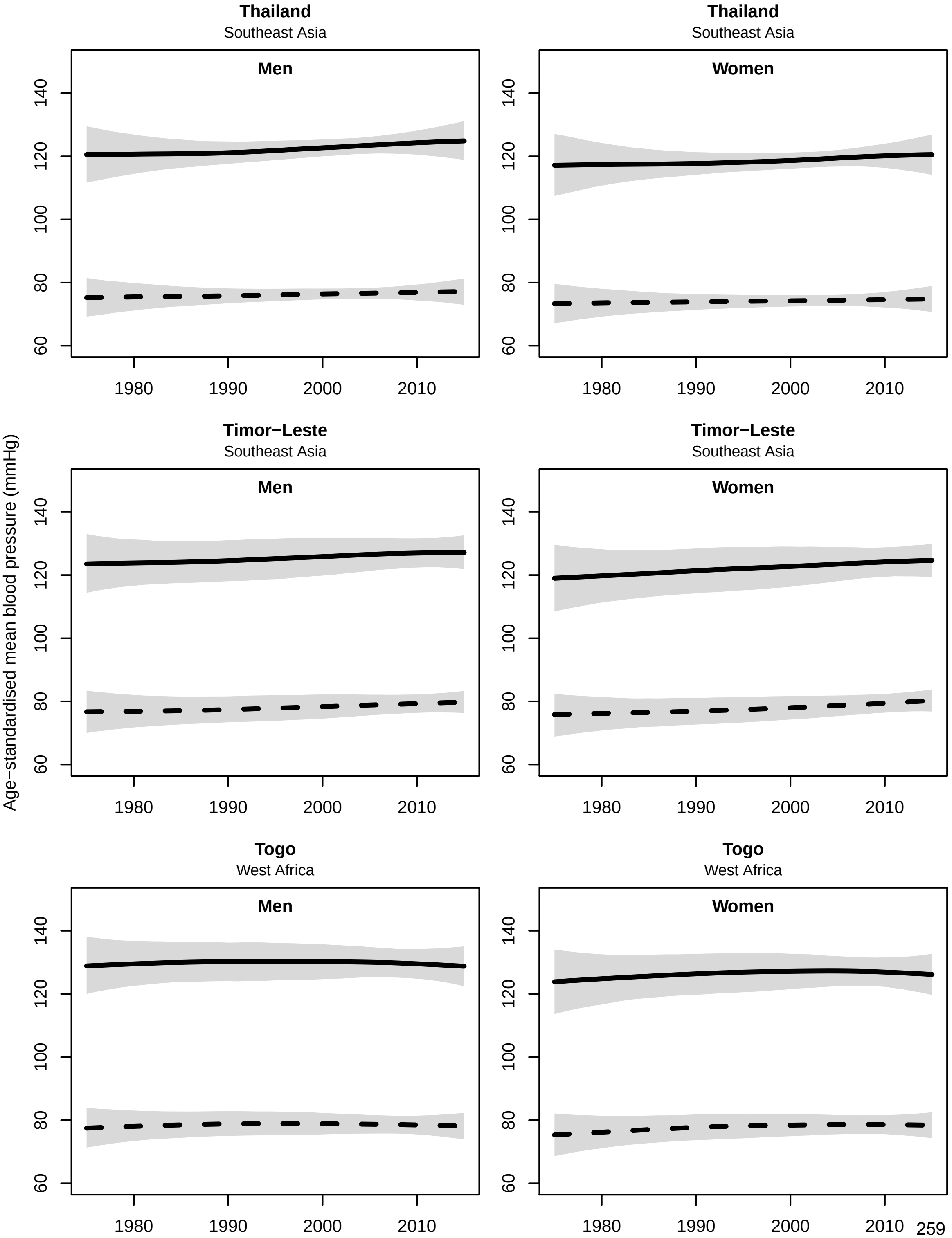
Thailand
Southeast Asia
Men
Thailand
Southeast Asia
Women
Timor−Leste
Southeast Asia
Men
Timor−Leste
Southeast Asia
Women
Togo
West Africa
Men
Togo
West Africa
Women
Age−standardised mean blood pressure (mmHg)
60
80
100
120
140
1980
1990
2000
2010

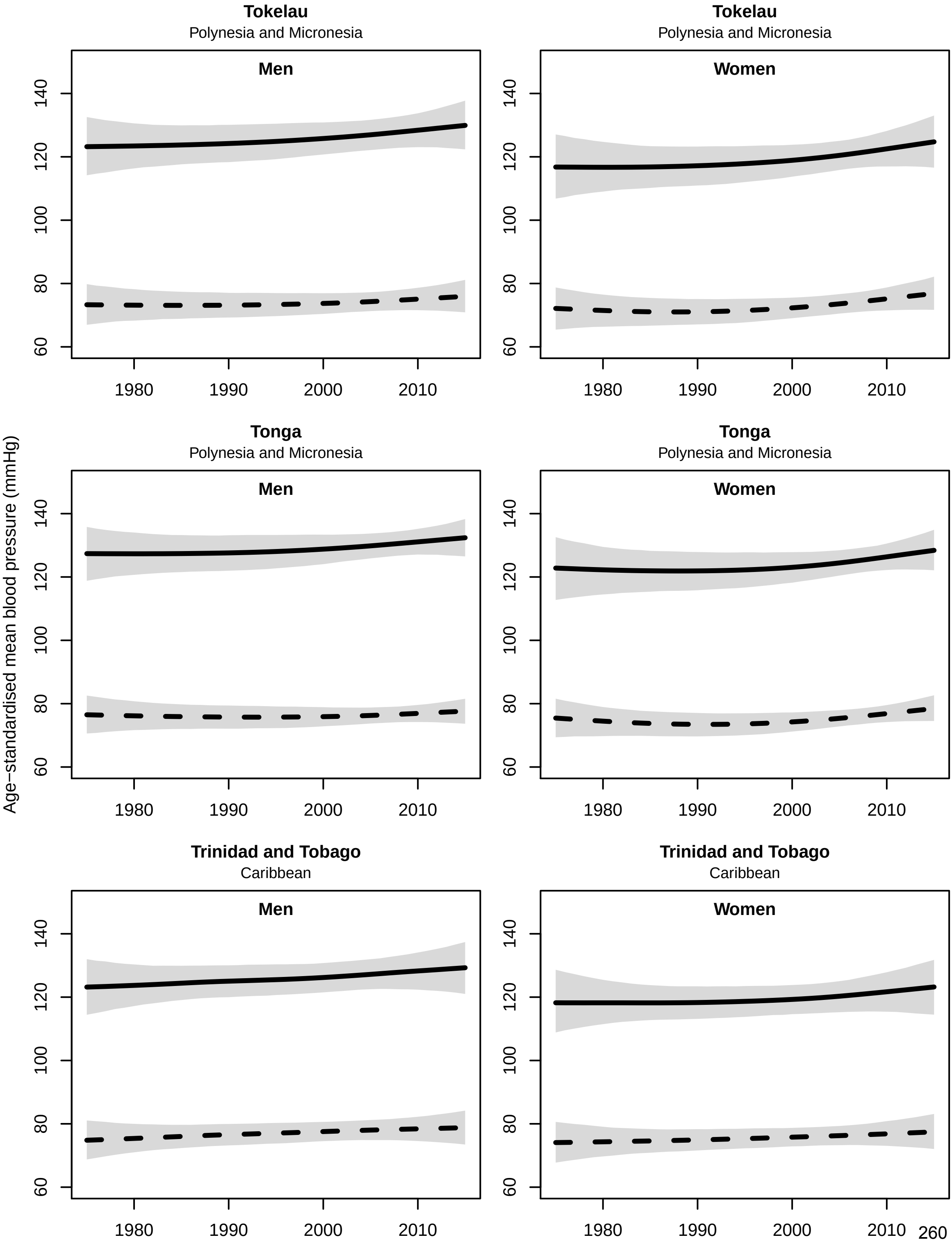

Tokelau
Polynesia and Micronesia
Men
Tokelau
Polynesia and Micronesia
Women
Tonga
Polynesia and Micronesia
Men
Tonga
Polynesia and Micronesia
Women
Trinidad and Tobago
Caribbean
Men
Trinidad and Tobago
Caribbean
Women
Age−standardised mean blood pressure (mmHg)
60
80
100
120
140
1980
1990
2000
2010

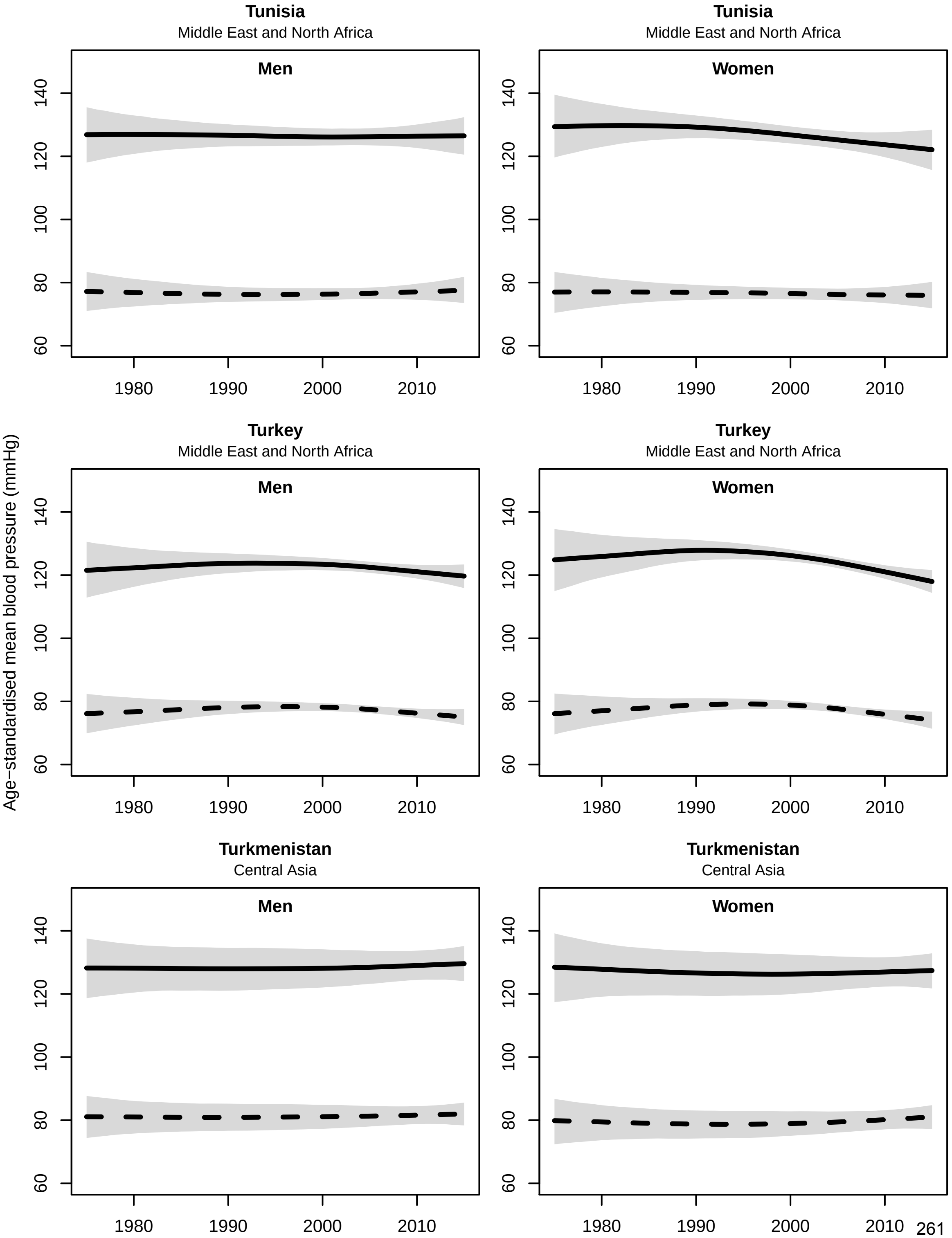
Tunisia
Middle East and North Africa
Men
Tunisia
Middle East and North Africa
Women
Turkey
Middle East and North Africa
Men
Turkey
Middle East and North Africa
Women
Turkmenistan
Central Asia
Men
Turkmenistan
Central Asia
Women
Age−standardised mean blood pressure (mmHg)
60
80
100
120
140
1980
1990
2000
2010

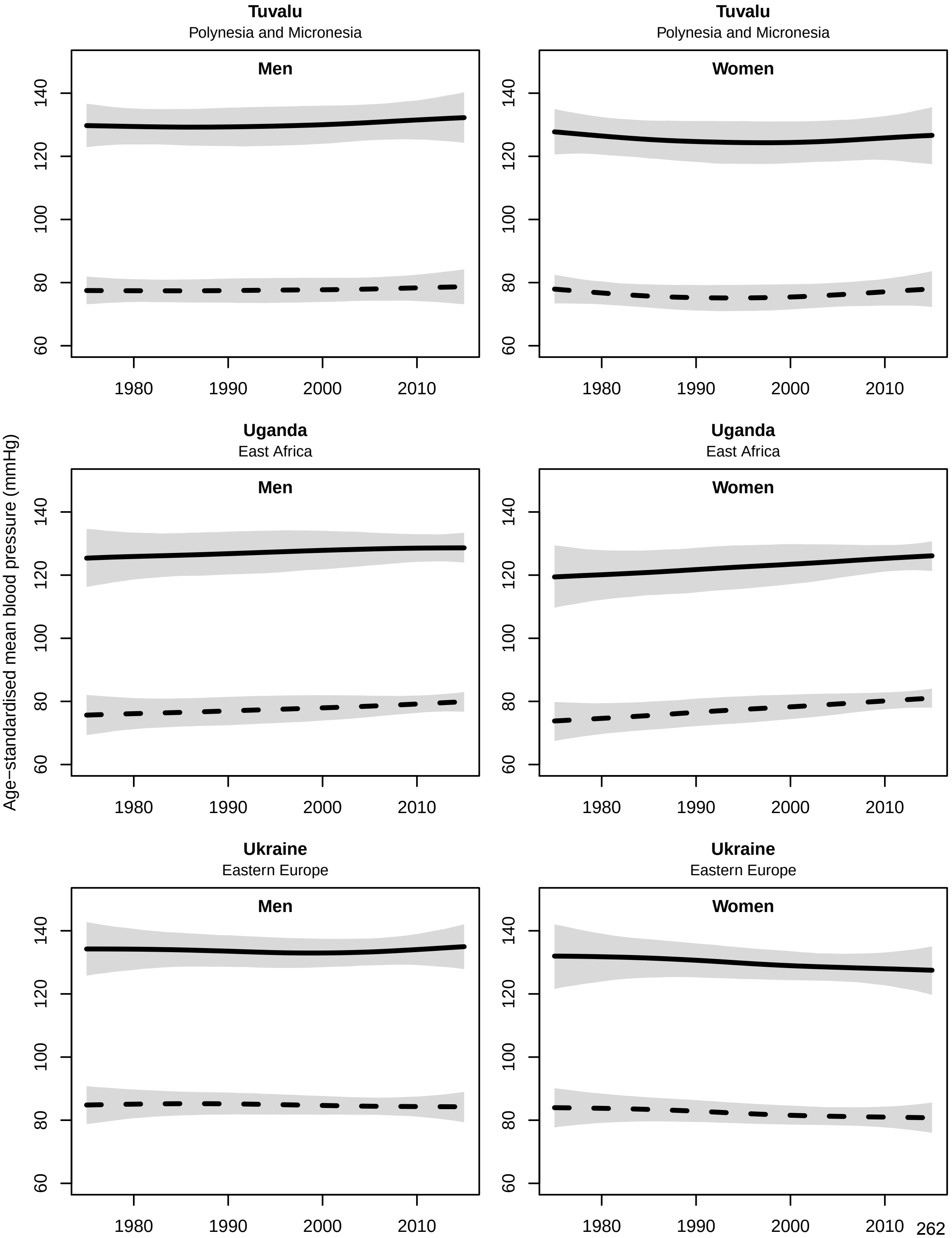
Tuvalu
Polynesia and Micronesia
Men
Tuvalu
Polynesia and Micronesia
Women
Uganda
East Africa
Men
Uganda
East Africa
Women
Ukraine
Eastern Europe
Men
Ukraine
Eastern Europe
Women
Age−standardised mean blood pressure (mmHg)
60
80
100
120
140
1980
1990
2000
2010

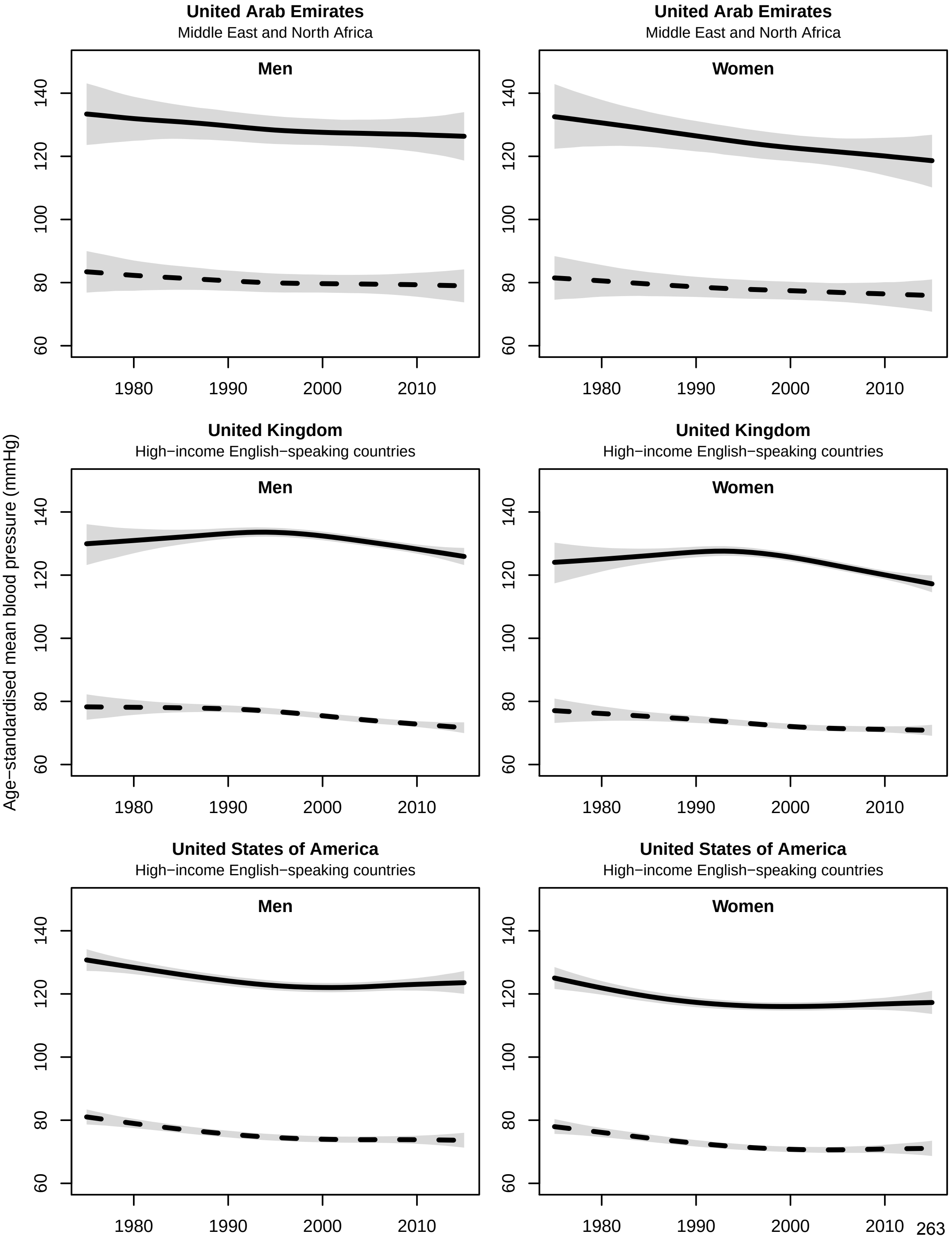

United Arab Emirates
Middle East and North Africa
Men
United Arab Emirates
Middle East and North Africa
Women
United Kingdom
High−income English−speaking countries
Men
United Kingdom
High−income English−speaking countries
Women
United States of America
High−income English−speaking countries
Men
United States of America
High−income English−speaking countries
Women
Age−standardised mean blood pressure (mmHg)
60
80
100
120
140
1980
1990
2000
2010

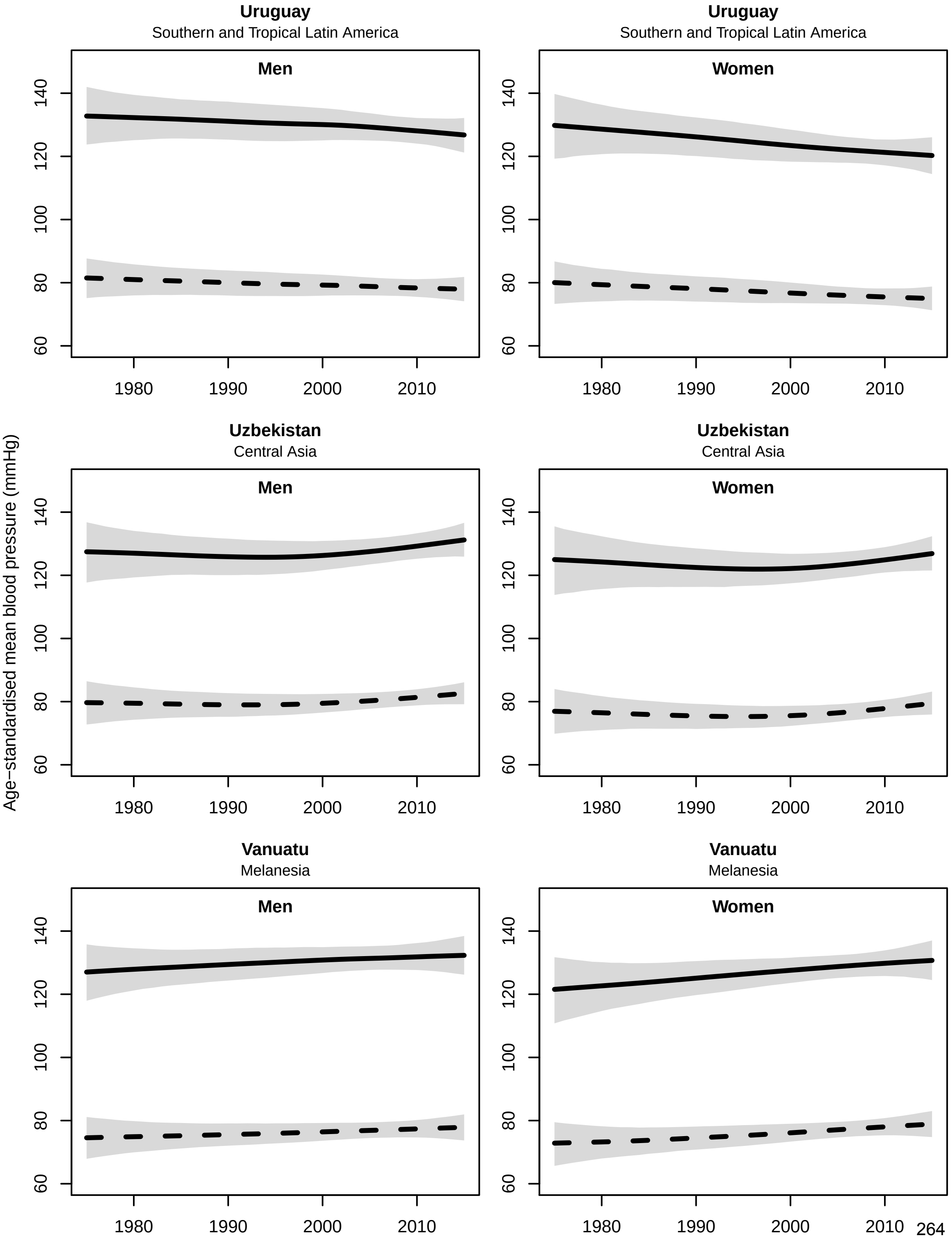
Uruguay
Southern and Tropical Latin America
Men
Women
Uzbekistan
Central Asia
Vanuatu
Melanesia
Age−standardised mean blood pressure (mmHg)
60
80
100
120
140
1980
1990
2000
2010

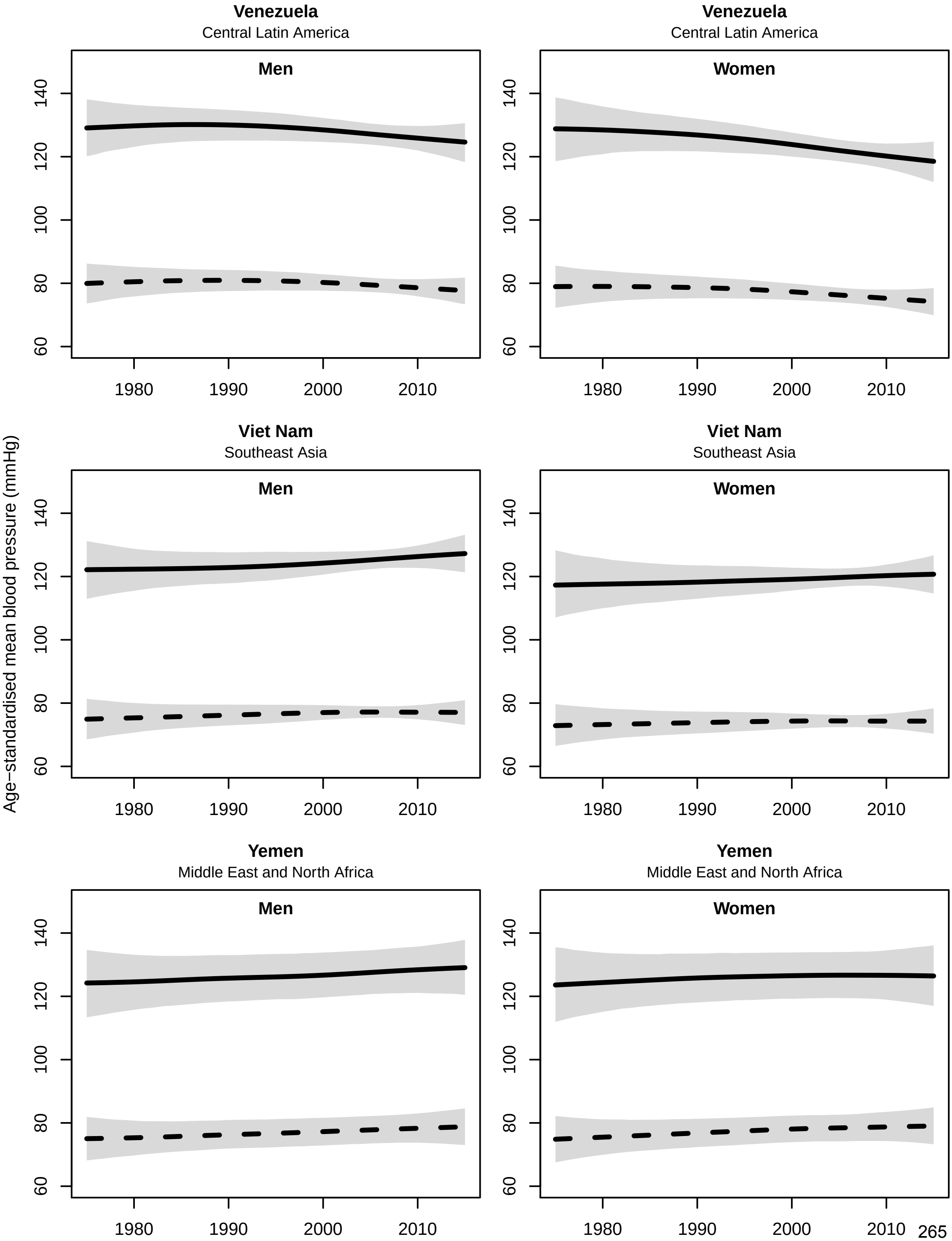
Venezuela
Central Latin America
Men
Venezuela
Central Latin America
Women
Viet Nam
Southeast Asia
Men
Viet Nam
Southeast Asia
Women
Yemen
Middle East and North Africa
Men
Yemen
Middle East and North Africa
Women
Age−standardised mean blood pressure (mmHg)
60
80
100
120
140
1980
1990
2000
2010

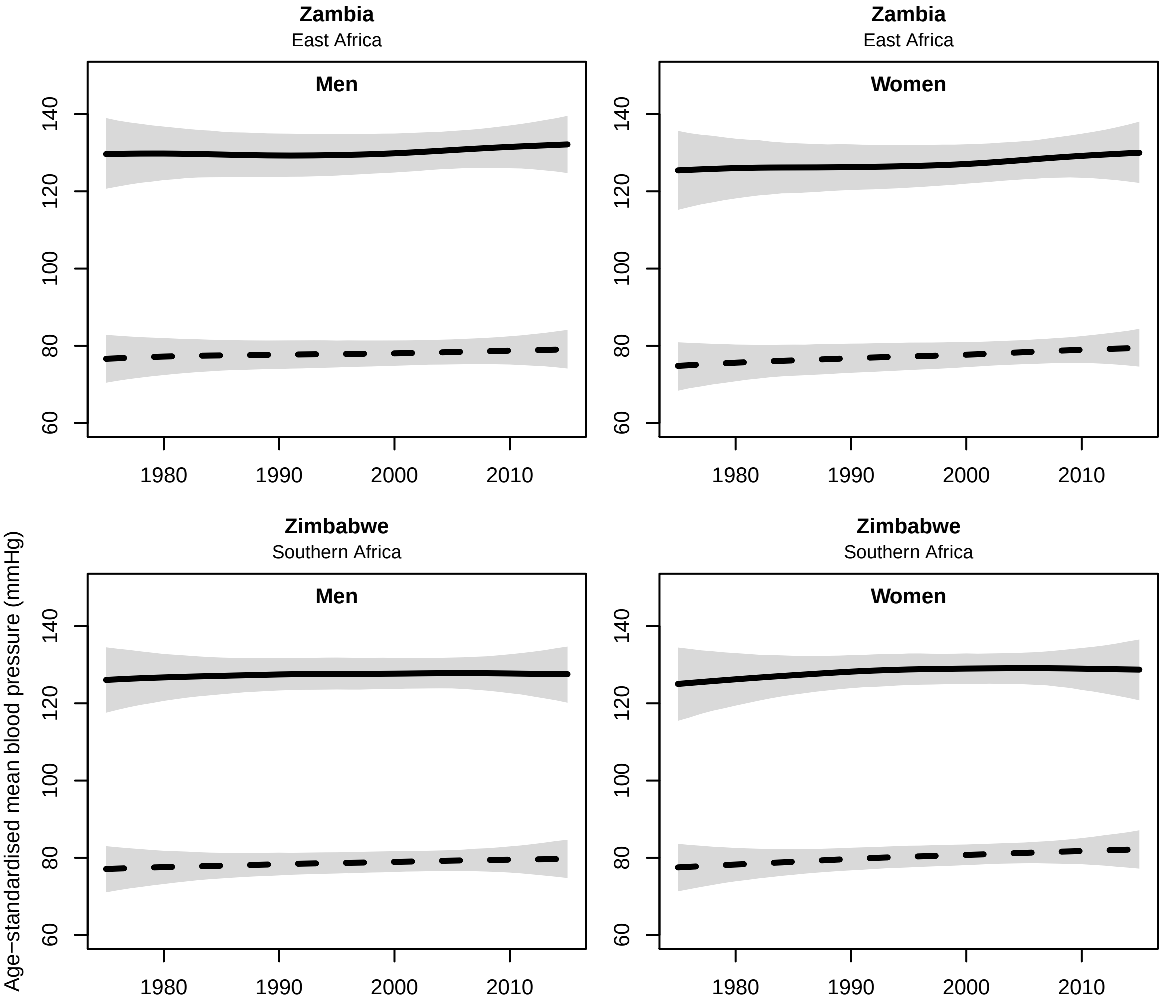
Zambia
East Africa
Men
Zambia
East Africa
Women
Zimbabwe
Southern Africa
Men
Zimbabwe
Southern Africa
Women
Age−standardised mean blood pressure (mmHg)
60
80
100
120
140
1980
1990
2000
2010

Appendix Figure 6: Trends in age-standardised and crude prevalence of raised blood pressure by sex and country in people aged 18 years and older. The lines show the posterior mean and the shaded area shows the 95% credible interval for age-standardised prevalence.

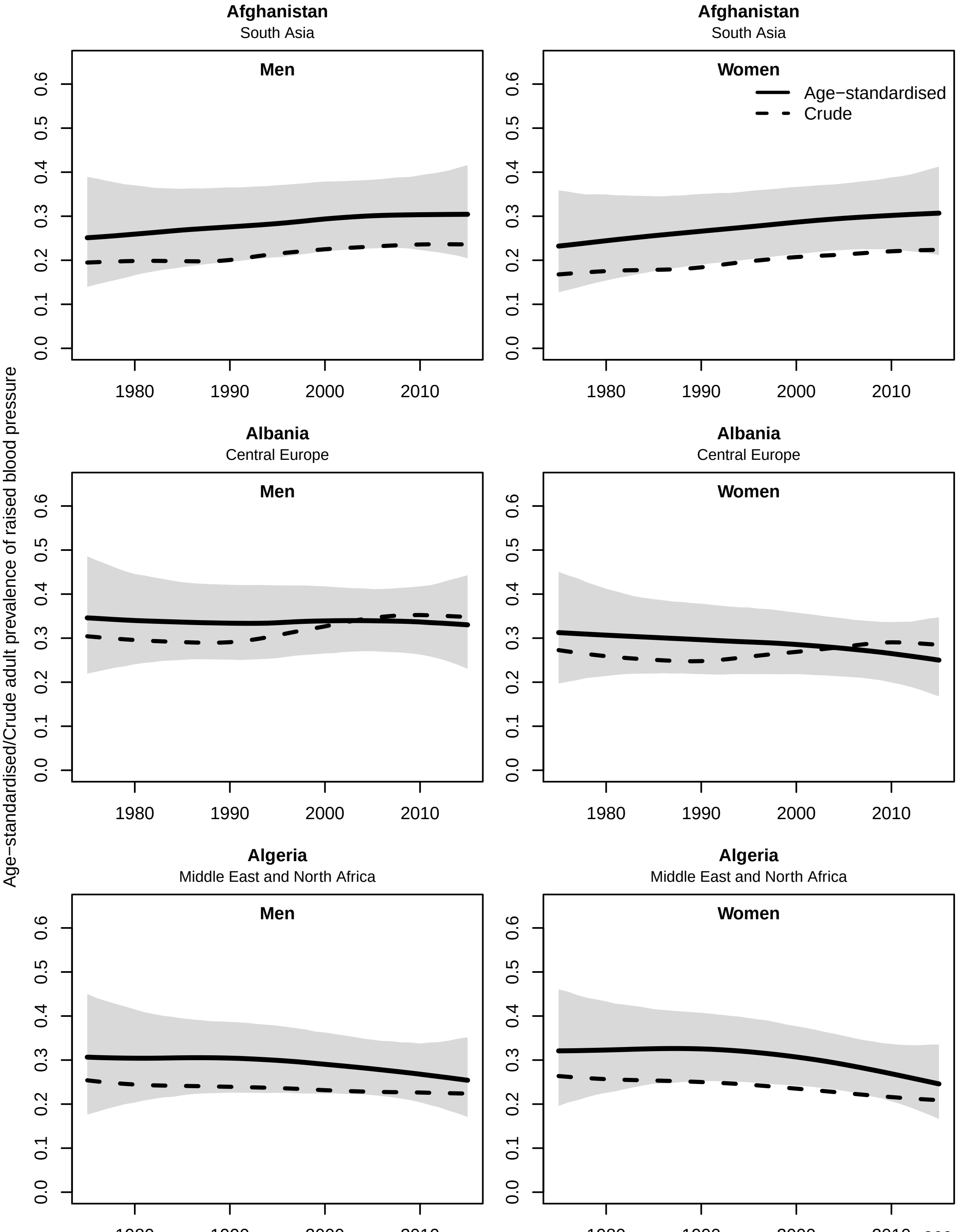
Afghanistan
South Asia
Men
Afghanistan
South Asia
Women
Age−standardised
Crude
Albania
Central Europe
Men
Albania
Central Europe
Women
Algeria
Middle East and North Africa
Men
Algeria
Middle East and North Africa
Women
Age−standardised/Crude adult prevalence of raised blood pressure
0.0
0.1
0.2
0.3
0.4
0.5
0.6
1980
1990
2000
2010

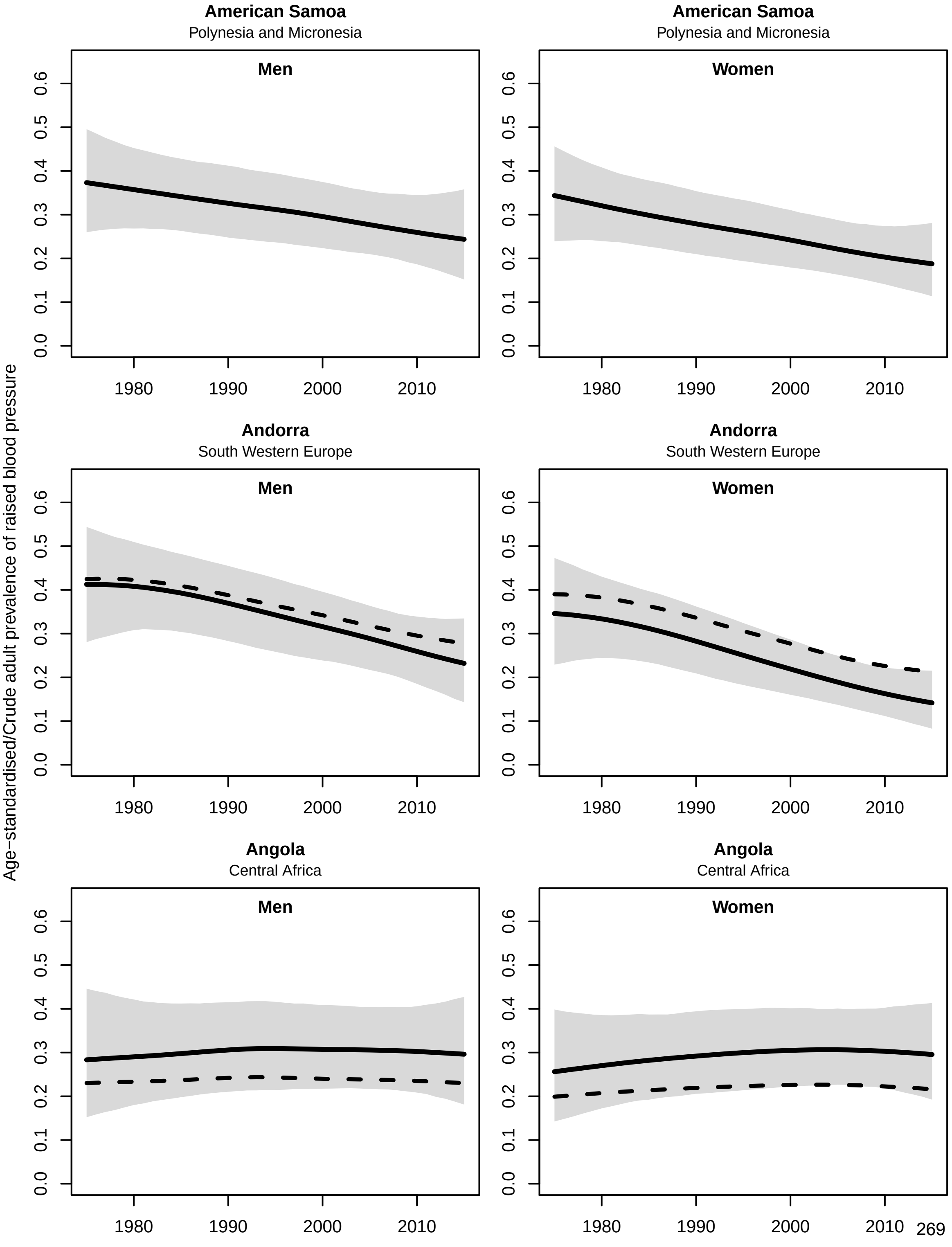
Age−standardised/Crude adult prevalence of raised blood pressure
American Samoa
Polynesia and Micronesia
Men
Women
Andorra
South Western Europe
Angola
Central Africa
0.0
0.1
0.2
0.3
0.4
0.5
0.6
1980
1990
2000
2010

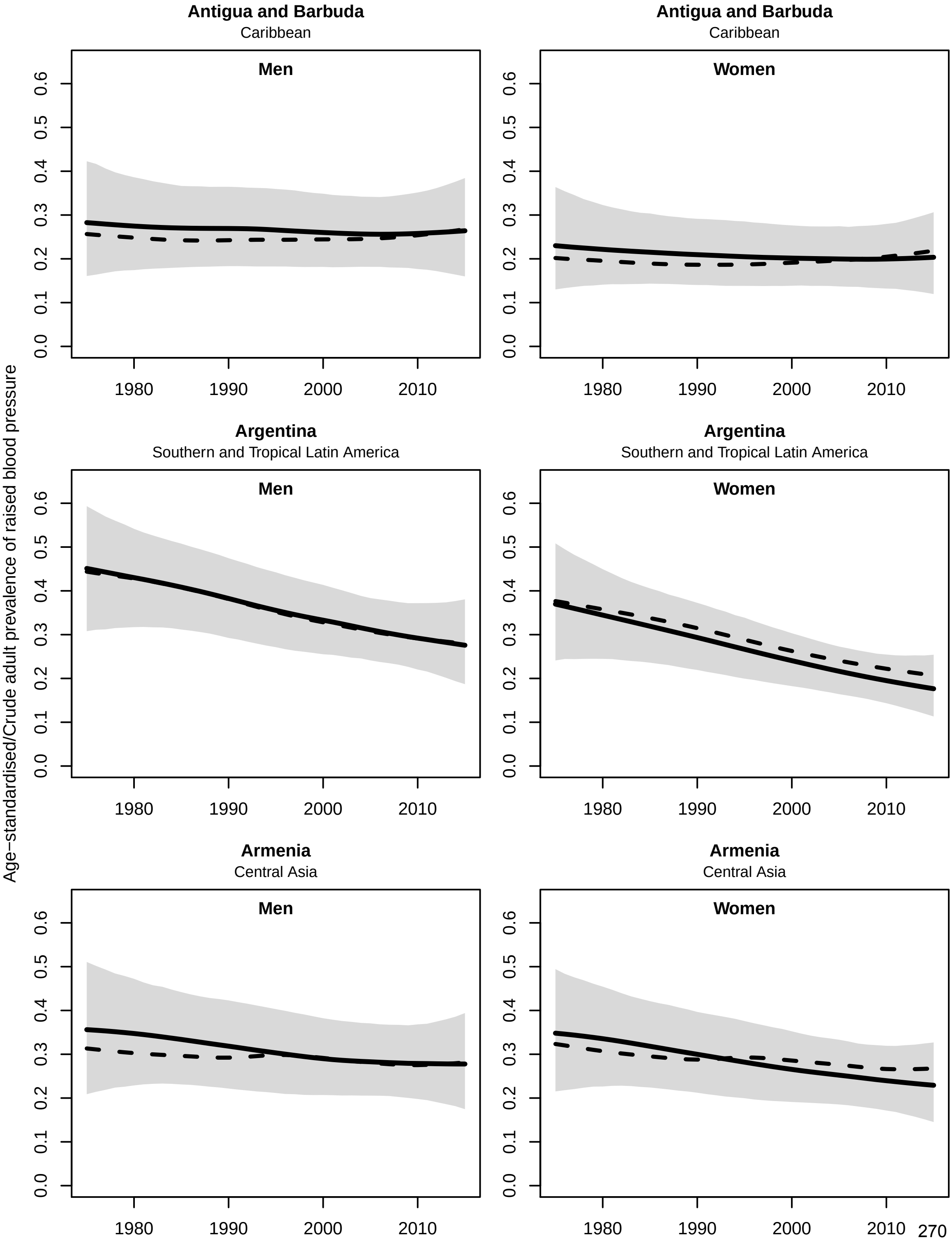
Antigua and Barbuda
Caribbean
Men
Women
Argentina
Southern and Tropical Latin America
Men
Women
Armenia
Central Asia
Men
Women
Age−standardised/Crude adult prevalence of raised blood pressure
0.0
0.1
0.2
0.3
0.4
0.5
0.6
1980
1990
2000
2010

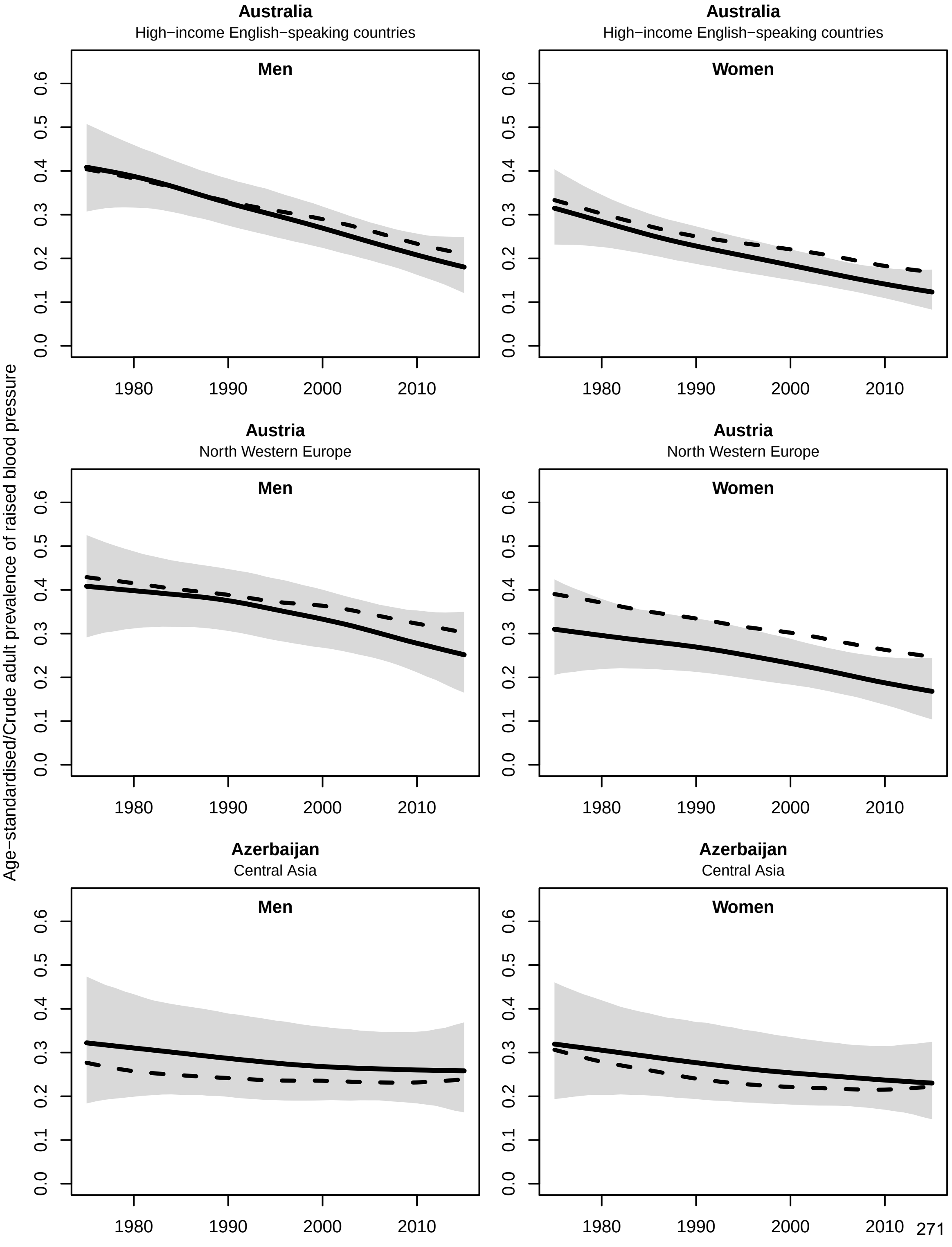
Australia
High−income English−speaking countries
Men
Australia
High−income English−speaking countries
Women
Austria
North Western Europe
Men
Austria
North Western Europe
Women
Azerbaijan
Central Asia
Men
Azerbaijan
Central Asia
Women
Age−standardised/Crude adult prevalence of raised blood pressure
0.0
0.1
0.2
0.3
0.4
0.5
0.6
1980
1990
2000
2010

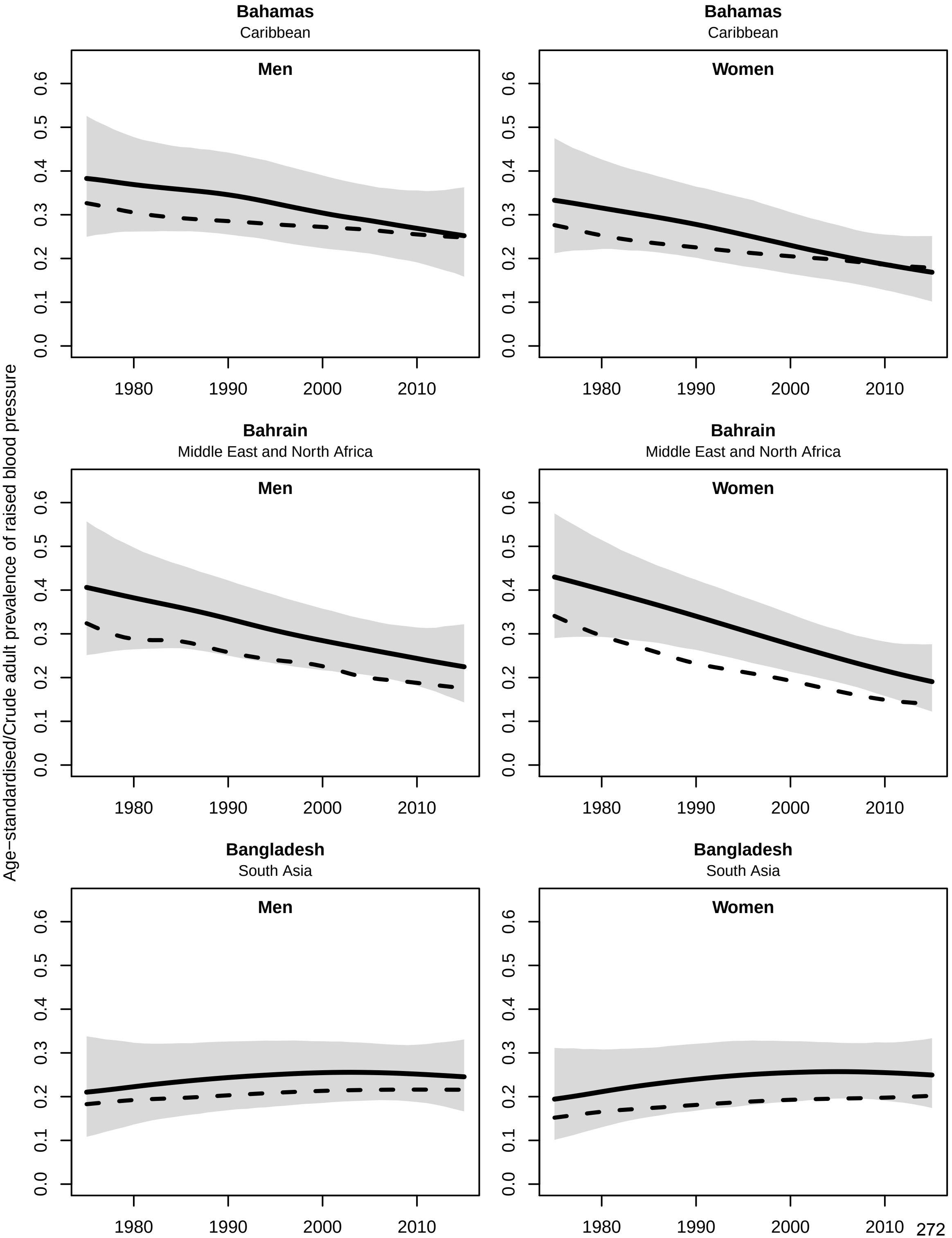
Bahamas
Caribbean
Men
Women
Bahrain
Middle East and North Africa
Bangladesh
South Asia
Age−standardised/Crude adult prevalence of raised blood pressure
0.0
0.1
0.2
0.3
0.4
0.5
0.6
1980
1990
2000
2010

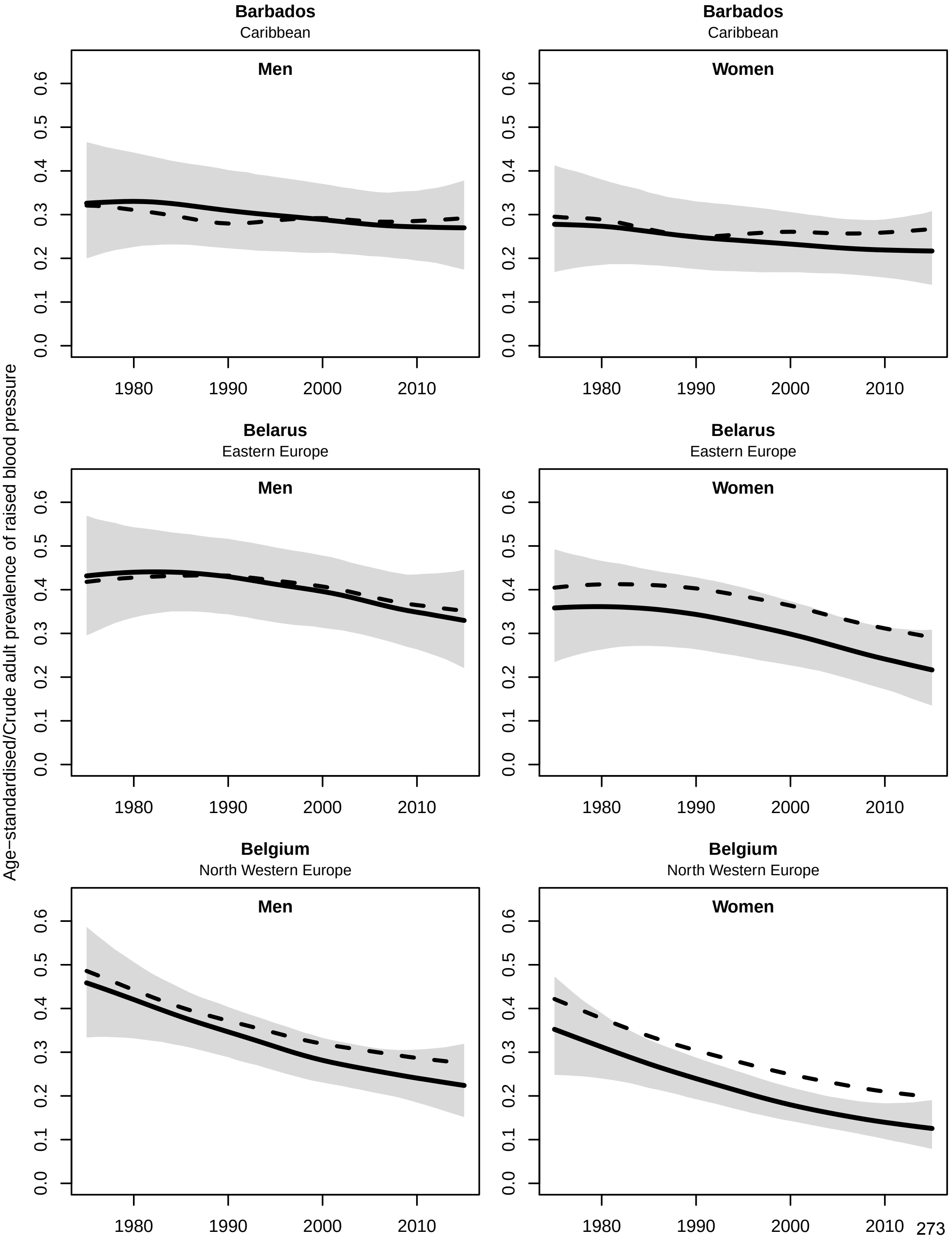
Barbados
Caribbean
Men
Barbados
Caribbean
Women
Belarus
Eastern Europe
Men
Belarus
Eastern Europe
Women
Belgium
North Western Europe
Men
Belgium
North Western Europe
Women
Age−standardised/Crude adult prevalence of raised blood pressure
0.0
0.1
0.2
0.3
0.4
0.5
0.6
1980
1990
2000
2010

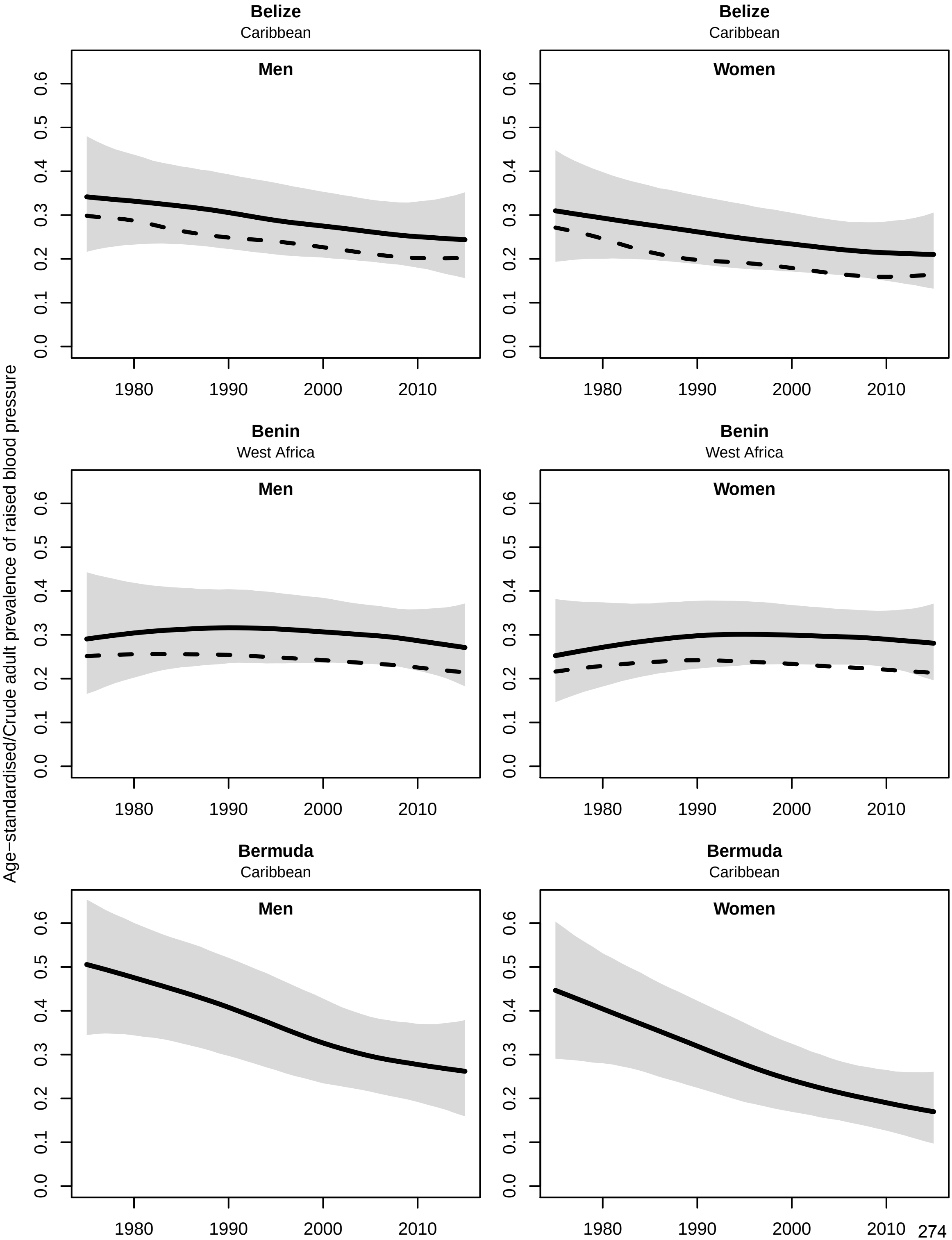
Belize
Caribbean
Men
Women
Benin
West Africa
Men
Women
Bermuda
Caribbean
Men
Women
Age−standardised/Crude adult prevalence of raised blood pressure
0.0
0.1
0.2
0.3
0.4
0.5
0.6
1980
1990
2000
2010

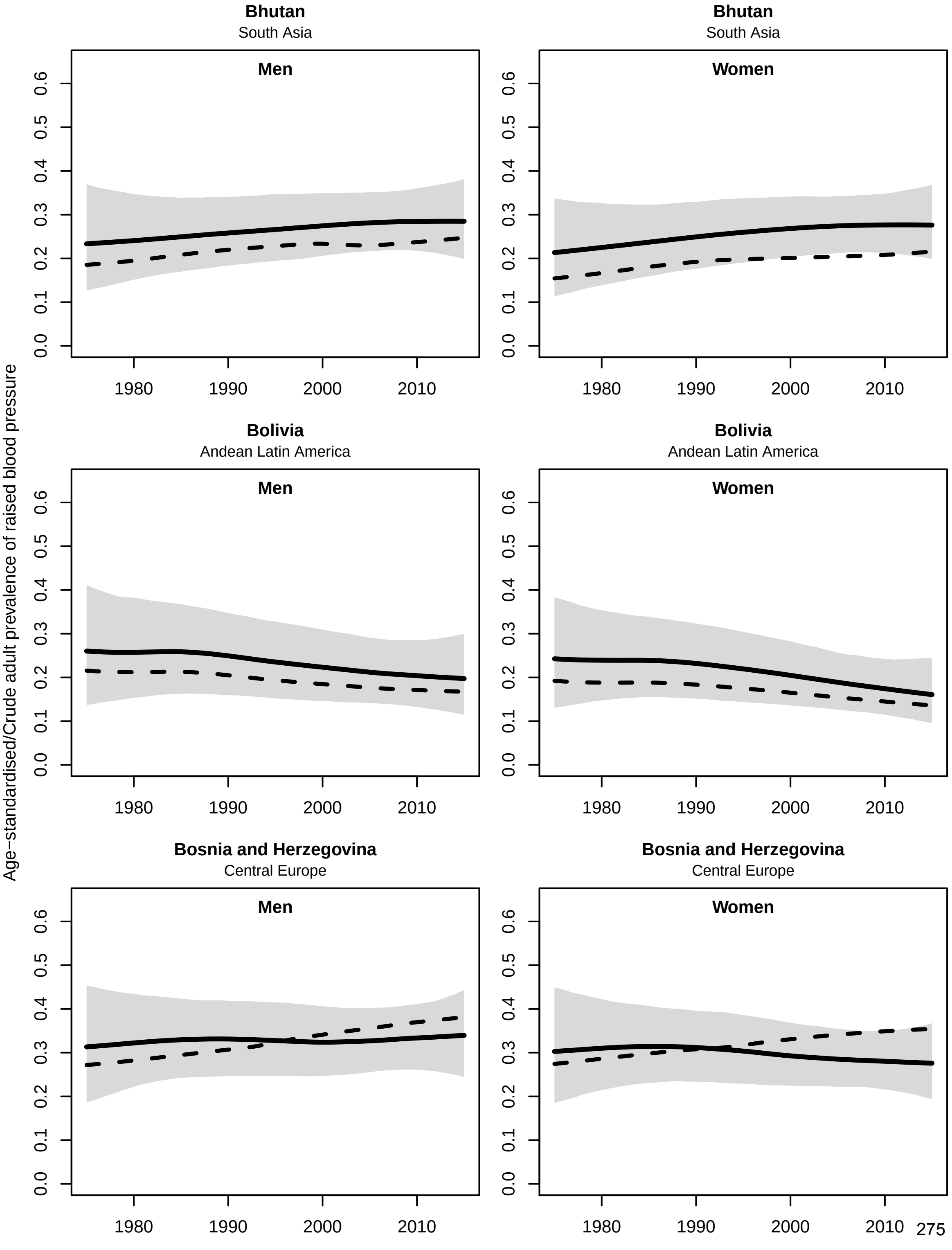

Age−standardised/Crude adult prevalence of raised blood pressure
Bhutan
South Asia
Men
Women
Bolivia
Andean Latin America
Men
Women
Bosnia and Herzegovina
Central Europe
Men
Women
0.0
0.1
0.2
0.3
0.4
0.5
0.6
1980
1990
2000
2010

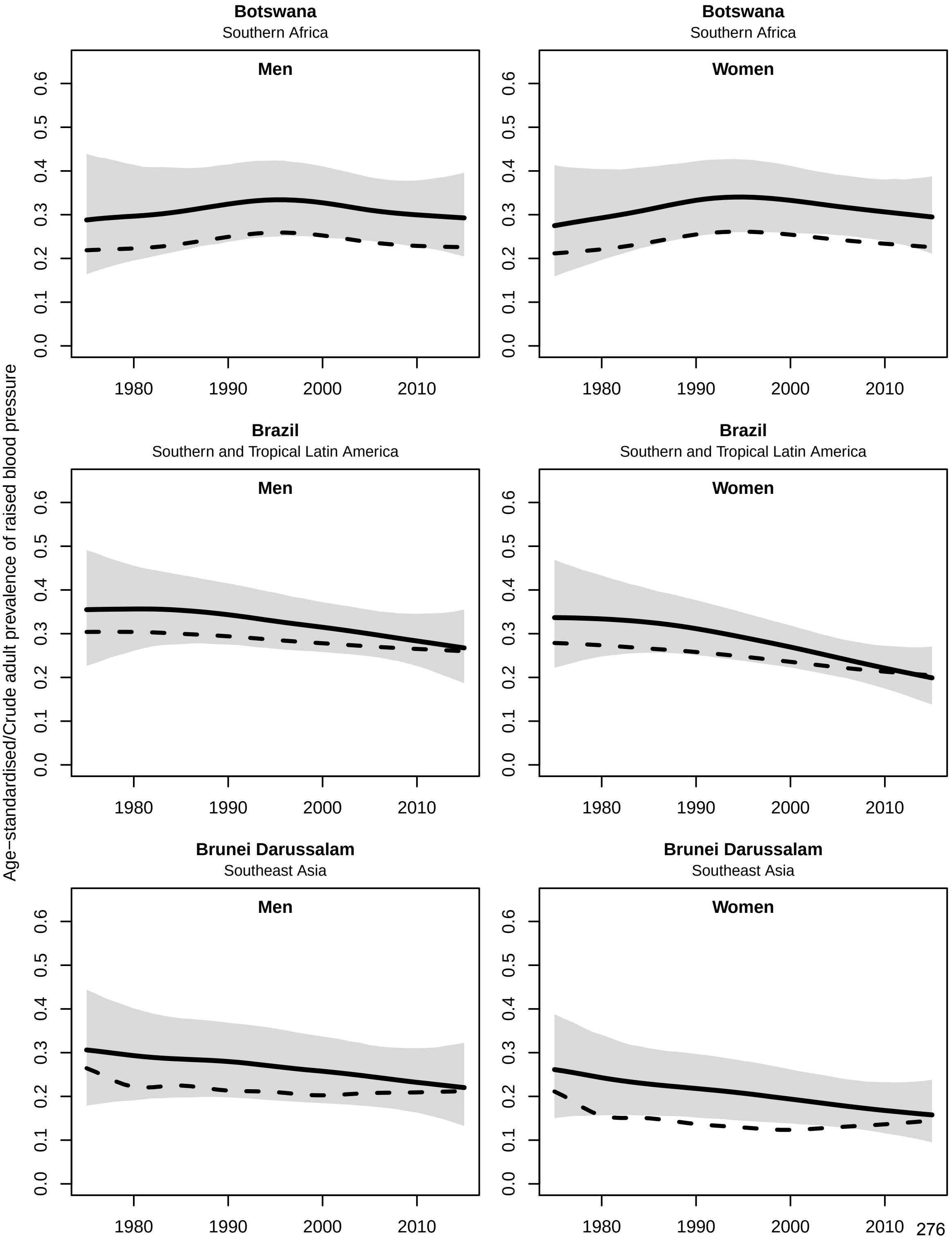
Botswana
Southern Africa
Men
Women
Brazil
Southern and Tropical Latin America
Brunei Darussalam
Southeast Asia
Age−standardised/Crude adult prevalence of raised blood pressure
0.0
0.1
0.2
0.3
0.4
0.5
0.6
1980
1990
2000
2010

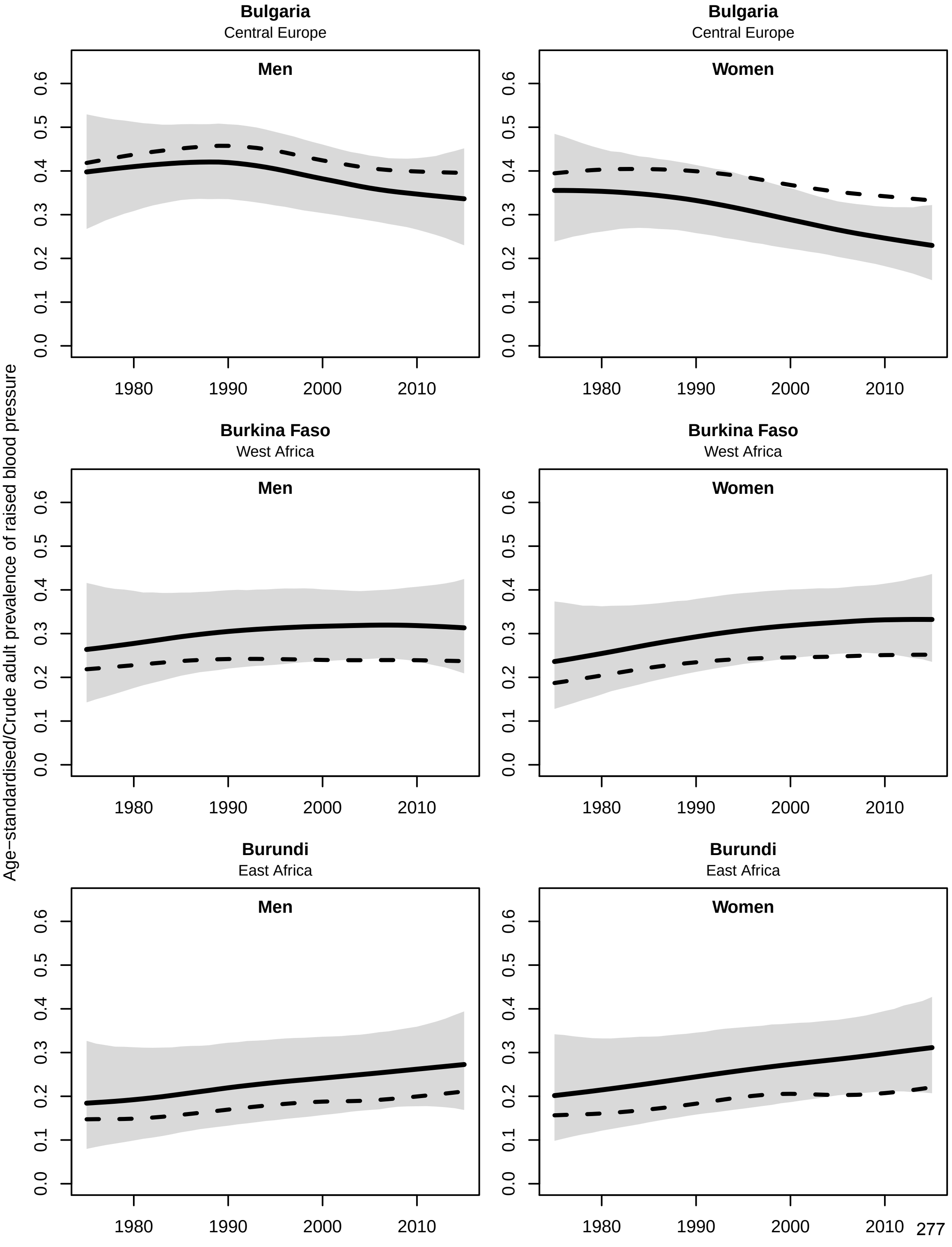

Bulgaria
Central Europe
Men
Bulgaria
Central Europe
Women
Burkina Faso
West Africa
Men
Burkina Faso
West Africa
Women
Burundi
East Africa
Men
Burundi
East Africa
Women
Age−standardised/Crude adult prevalence of raised blood pressure
0.0
0.1
0.2
0.3
0.4
0.5
0.6
1980
1990
2000
2010

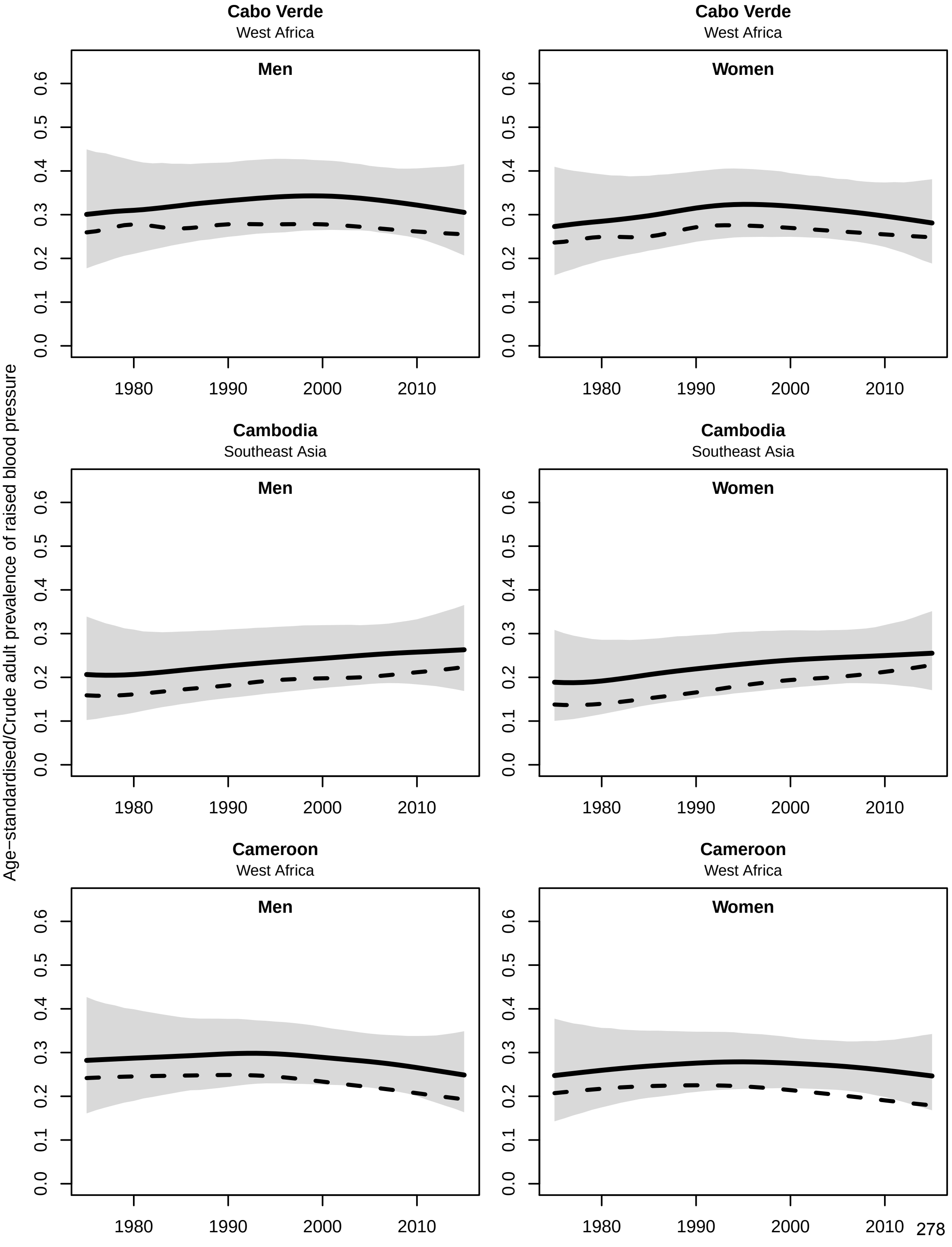

Cabo Verde
West Africa
Men
Cabo Verde
West Africa
Women
Cambodia
Southeast Asia
Men
Cambodia
Southeast Asia
Women
Cameroon
West Africa
Men
Cameroon
West Africa
Women
Age−standardised/Crude adult prevalence of raised blood pressure
0.0
0.1
0.2
0.3
0.4
0.5
0.6
1980
1990
2000
2010

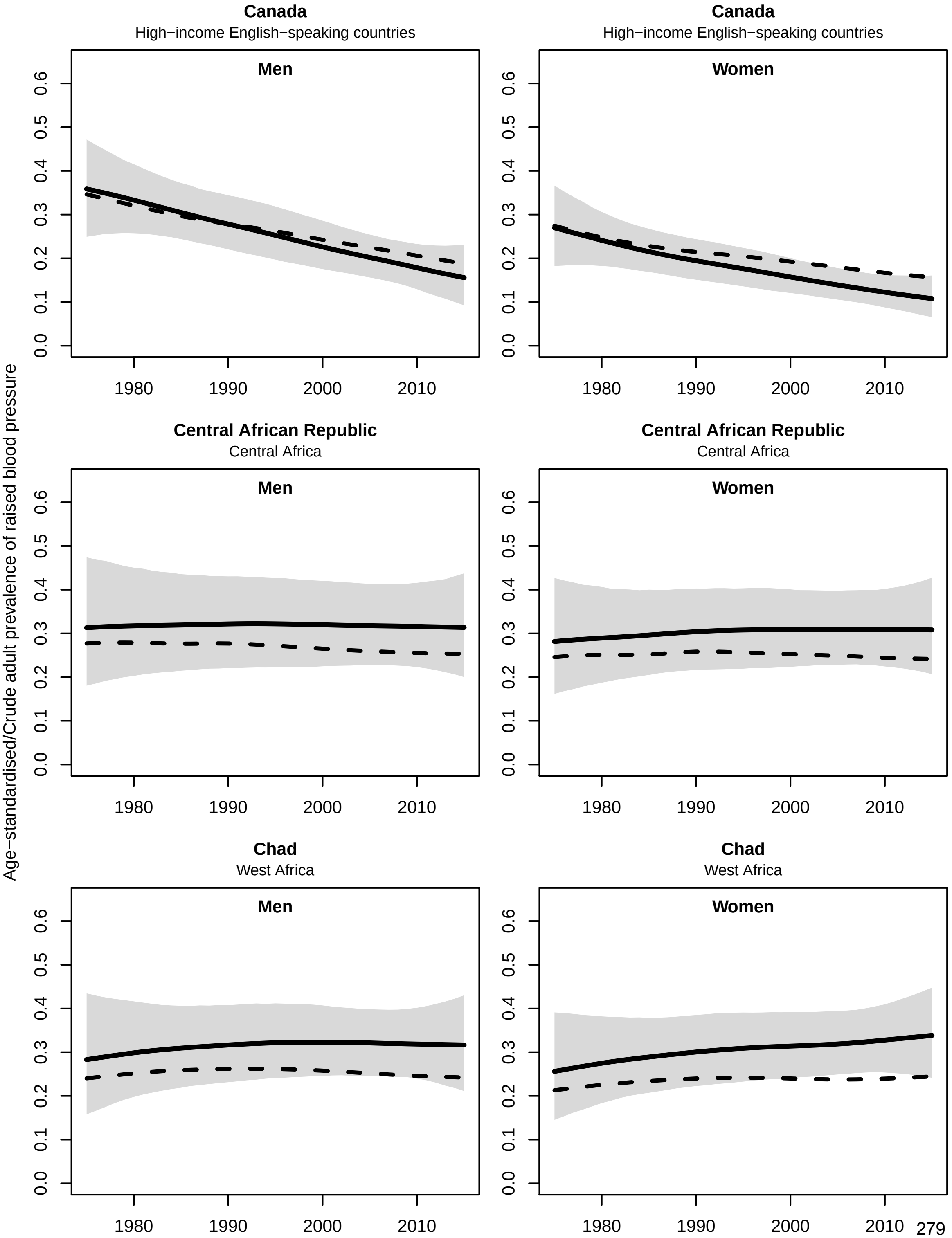
Canada
High−income English−speaking countries
Men
Canada
High−income English−speaking countries
Women
Central African Republic
Central Africa
Men
Central African Republic
Central Africa
Women
Chad
West Africa
Men
Chad
West Africa
Women
Age−standardised/Crude adult prevalence of raised blood pressure
0.0
0.1
0.2
0.3
0.4
0.5
0.6
1980
1990
2000
2010

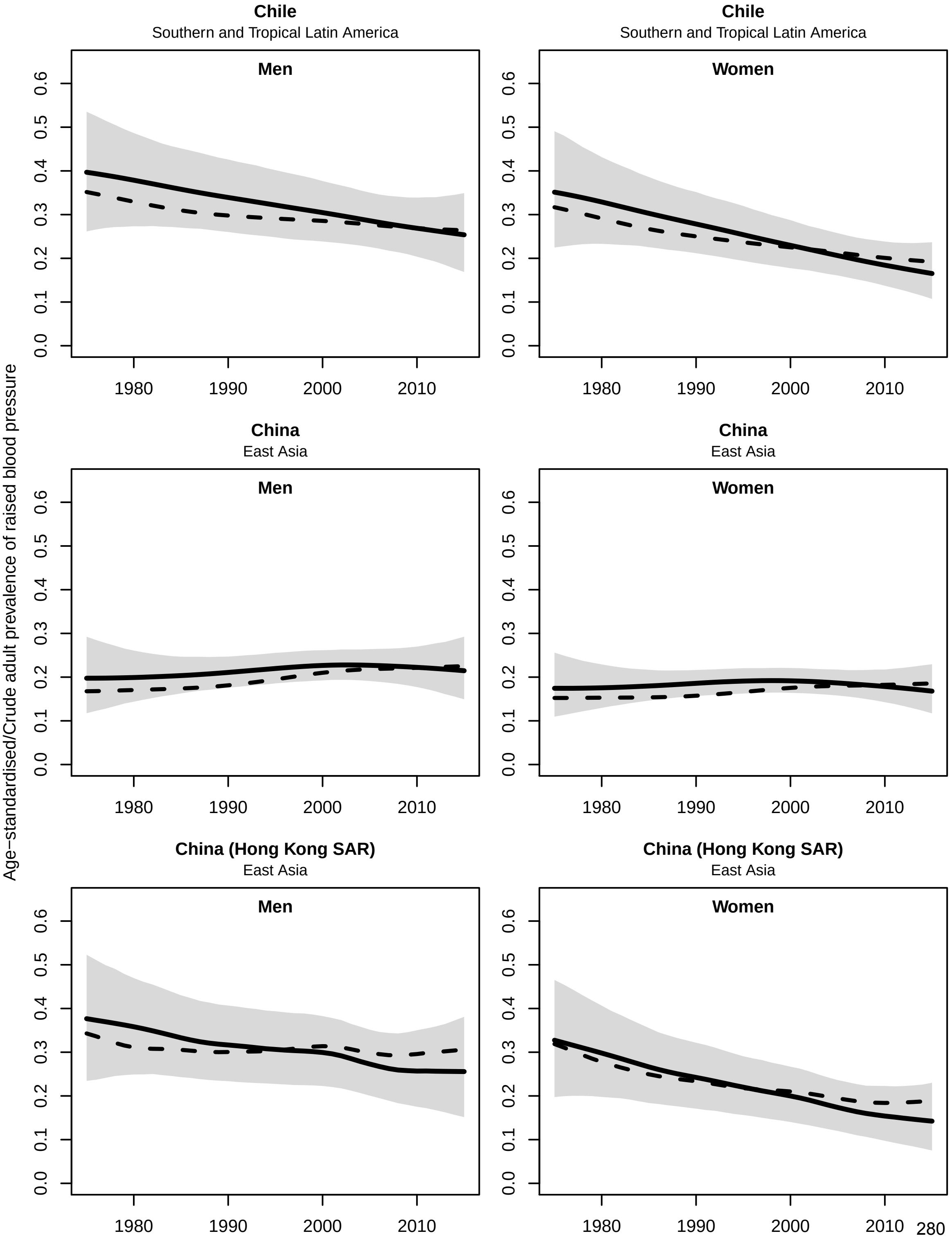

Age−standardised/Crude adult prevalence of raised blood pressure
Chile
Southern and Tropical Latin America
Men
Women
China
East Asia
China (Hong Kong SAR)
0.0
0.1
0.2
0.3
0.4
0.5
0.6
1980
1990
2000
2010

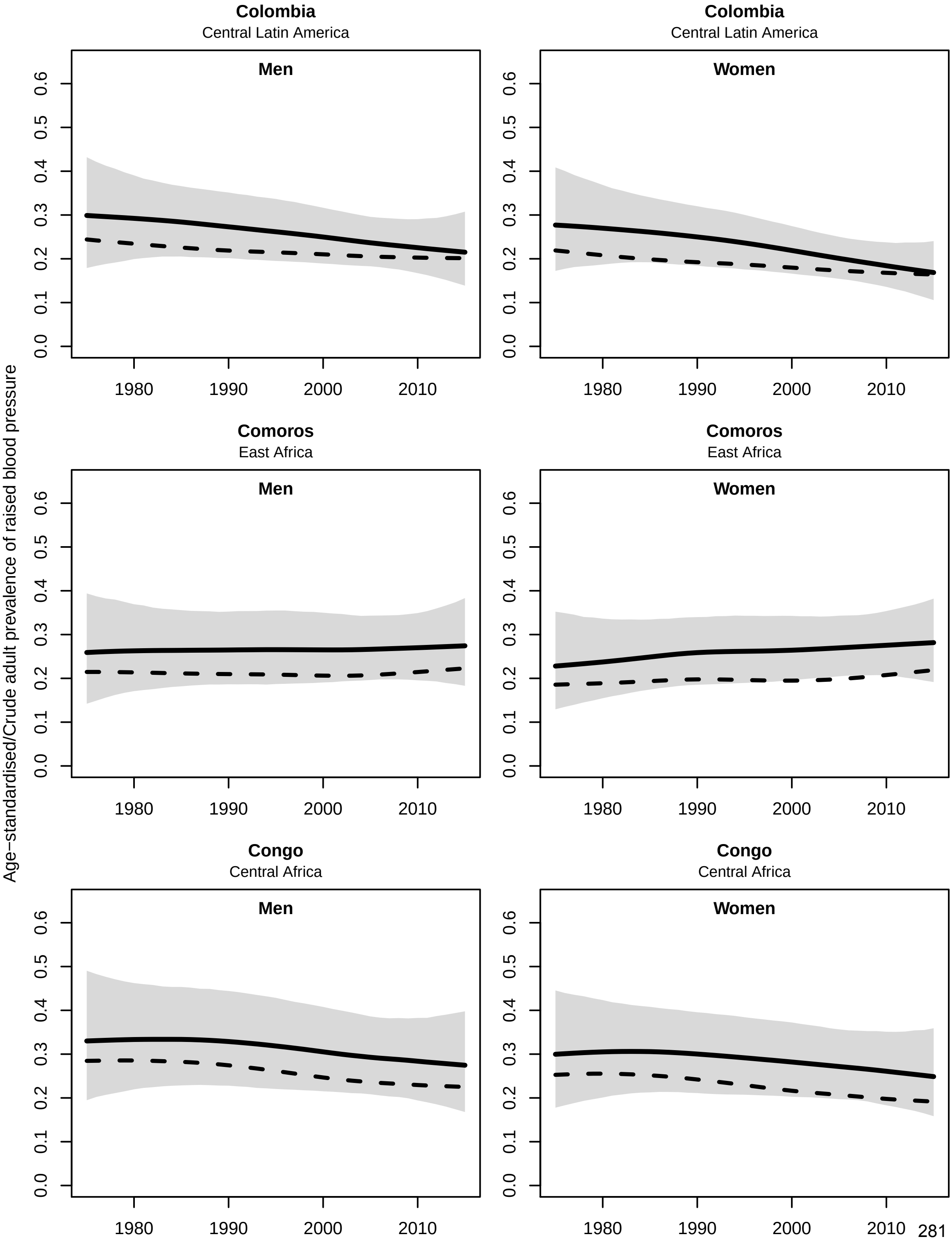
Colombia
Central Latin America
Men
Colombia
Central Latin America
Women
Comoros
East Africa
Men
Comoros
East Africa
Women
Congo
Central Africa
Men
Congo
Central Africa
Women
Age−standardised/Crude adult prevalence of raised blood pressure
0.0
0.1
0.2
0.3
0.4
0.5
0.6
1980
1990
2000
2010

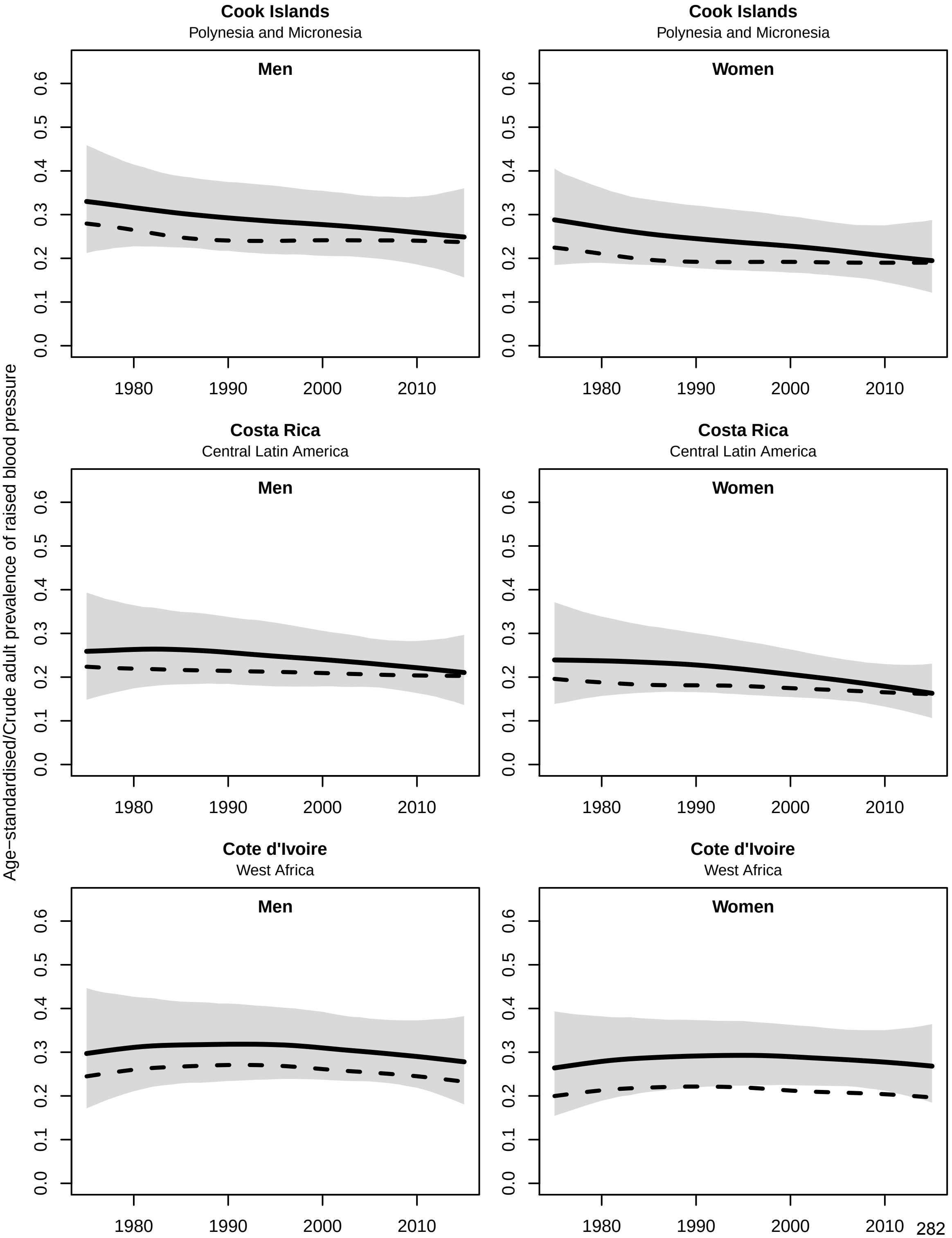

Cook Islands
Polynesia and Micronesia
Men
Cook Islands
Polynesia and Micronesia
Women
Costa Rica
Central Latin America
Men
Costa Rica
Central Latin America
Women
Cote d'Ivoire
West Africa
Men
Cote d'Ivoire
West Africa
Women
Age−standardised/Crude adult prevalence of raised blood pressure
0.0
0.1
0.2
0.3
0.4
0.5
0.6
1980
1990
2000
2010

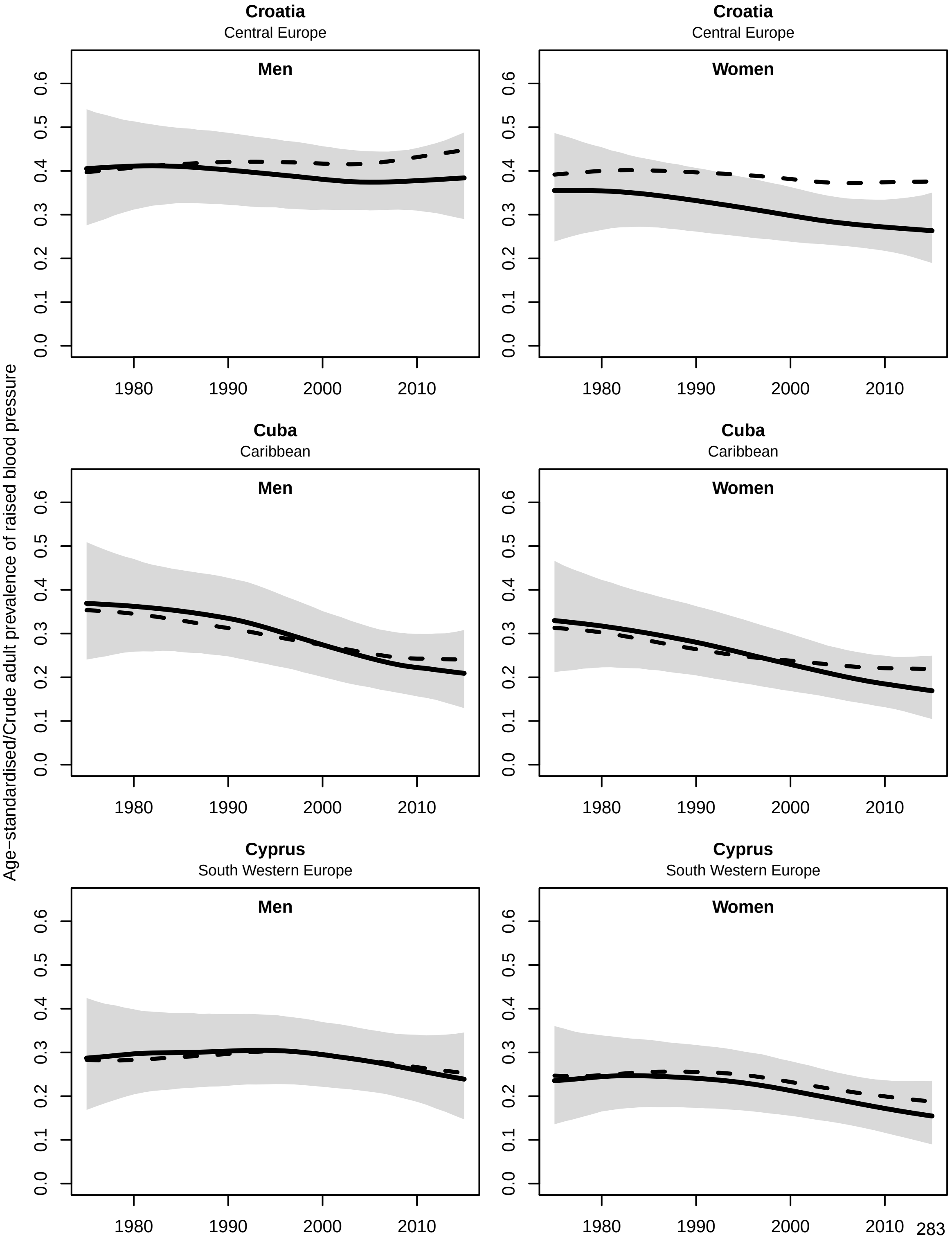
Croatia
Central Europe
Men
Croatia
Central Europe
Women
Cuba
Caribbean
Men
Cuba
Caribbean
Women
Cyprus
South Western Europe
Men
Cyprus
South Western Europe
Women
Age−standardised/Crude adult prevalence of raised blood pressure
0.0
0.1
0.2
0.3
0.4
0.5
0.6
1980
1990
2000
2010

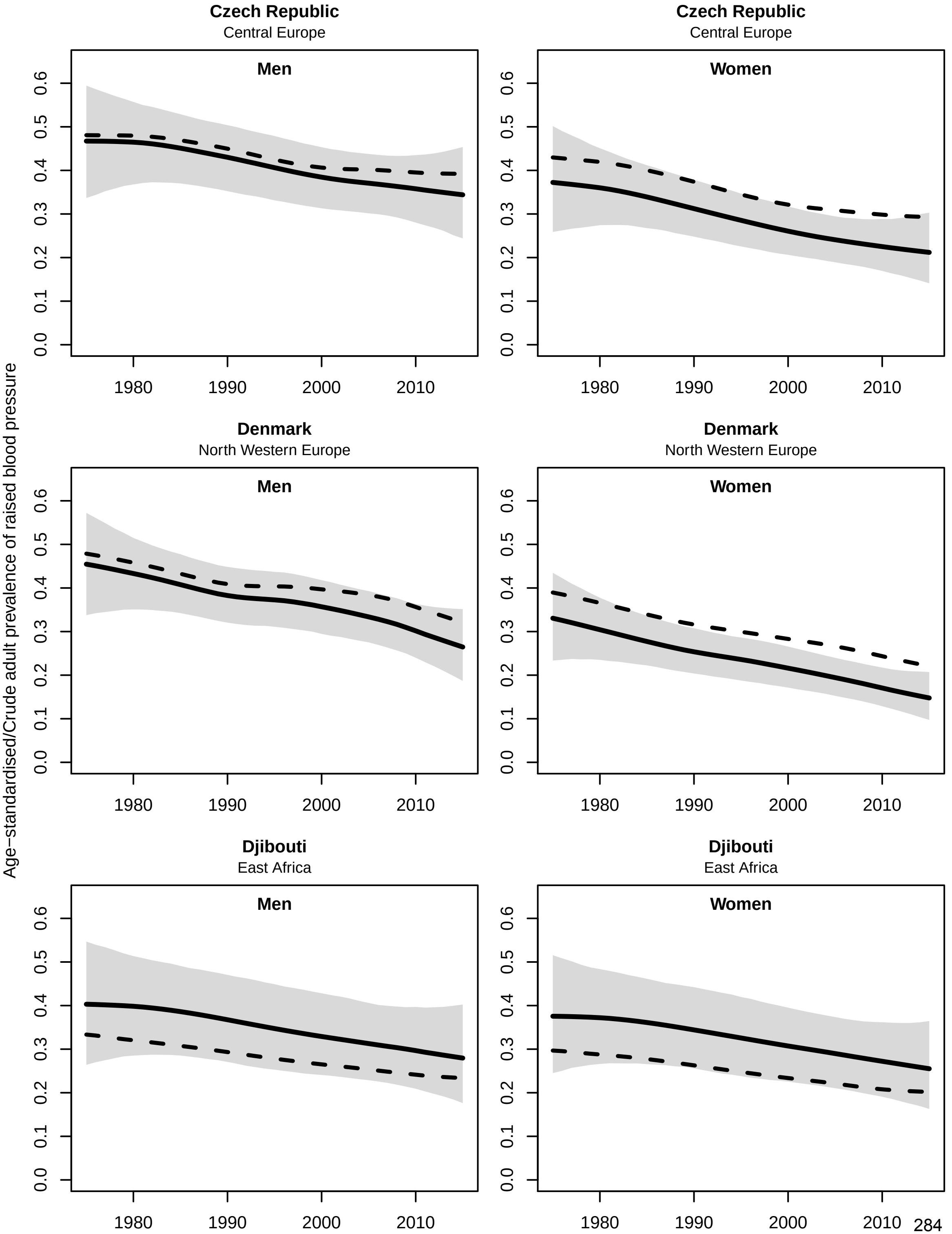

Czech Republic
Central Europe
Men
Women
Denmark
North Western Europe
Djibouti
East Africa
Age−standardised/Crude adult prevalence of raised blood pressure
0.0
0.1
0.2
0.3
0.4
0.5
0.6
1980
1990
2000
2010

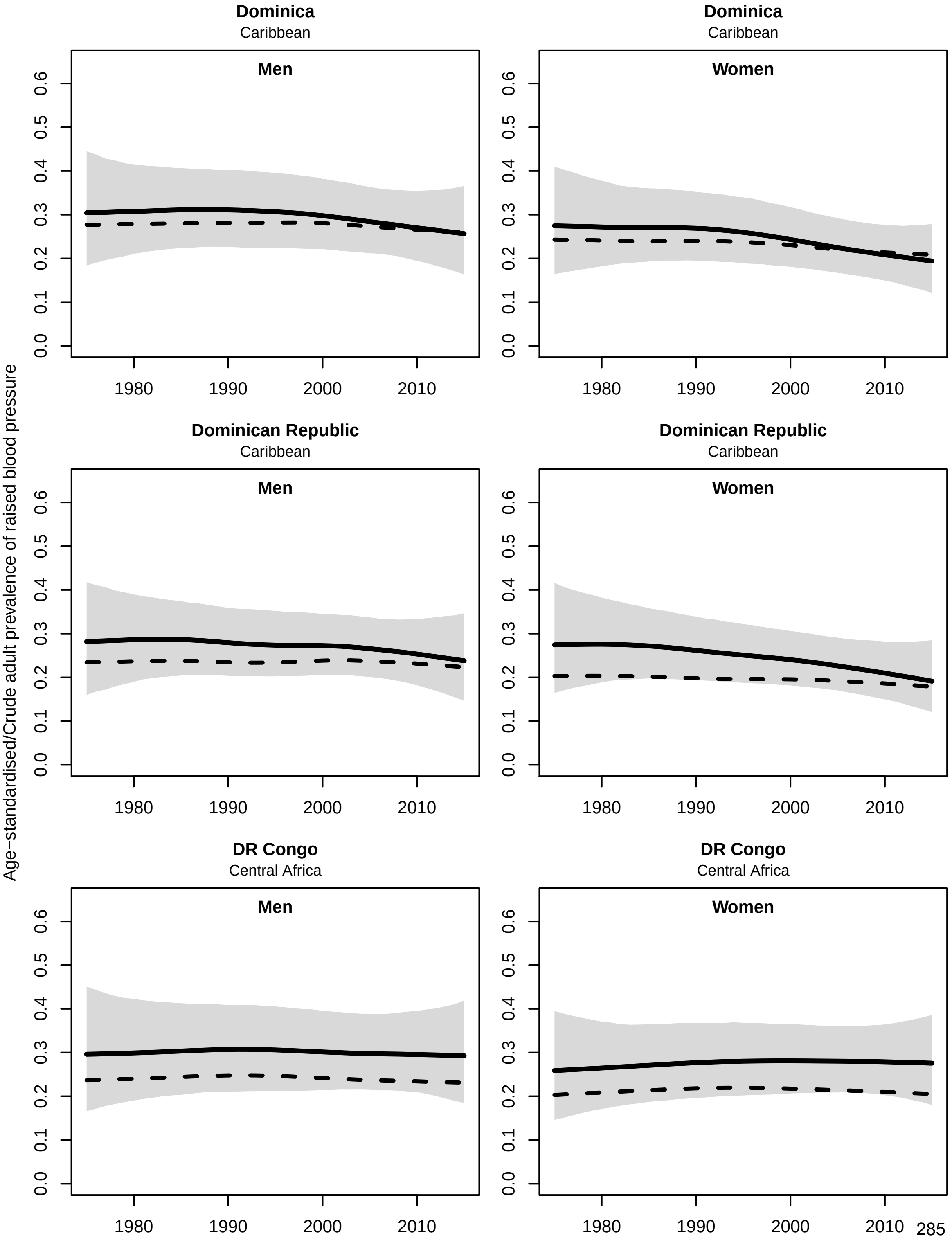
Dominica
Caribbean
Men
Dominica
Caribbean
Women
Dominican Republic
Caribbean
Men
Dominican Republic
Caribbean
Women
DR Congo
Central Africa
Men
DR Congo
Central Africa
Women
Age−standardised/Crude adult prevalence of raised blood pressure
0.0
0.1
0.2
0.3
0.4
0.5
0.6
1980
1990
2000
2010

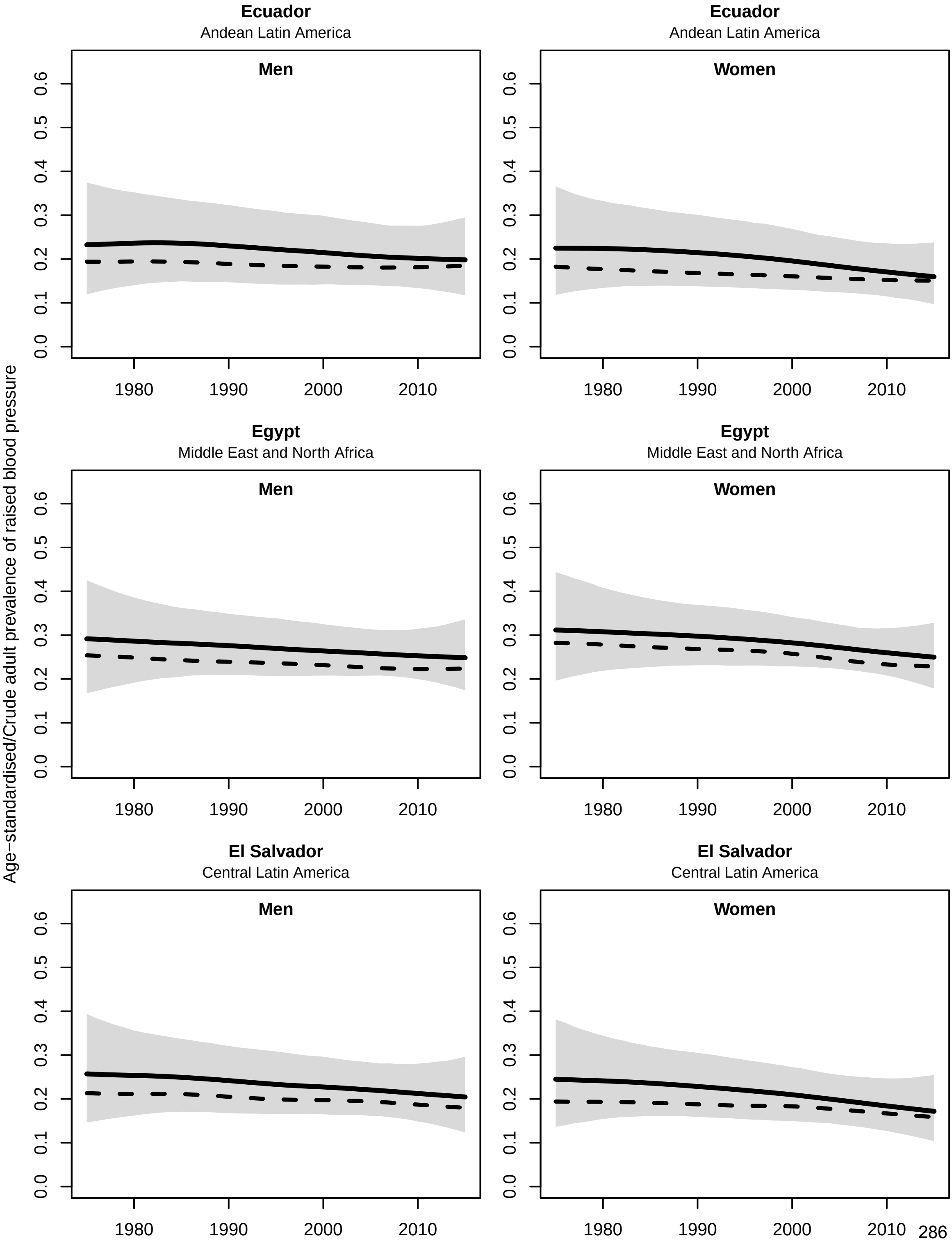
Ecuador
Andean Latin America
Men
Ecuador
Andean Latin America
Women
Egypt
Middle East and North Africa
Men
Egypt
Middle East and North Africa
Women
El Salvador
Central Latin America
Men
El Salvador
Central Latin America
Women
Age−standardised/Crude adult prevalence of raised blood pressure
0.0
0.1
0.2
0.3
0.4
0.5
0.6
1980
1990
2000
2010

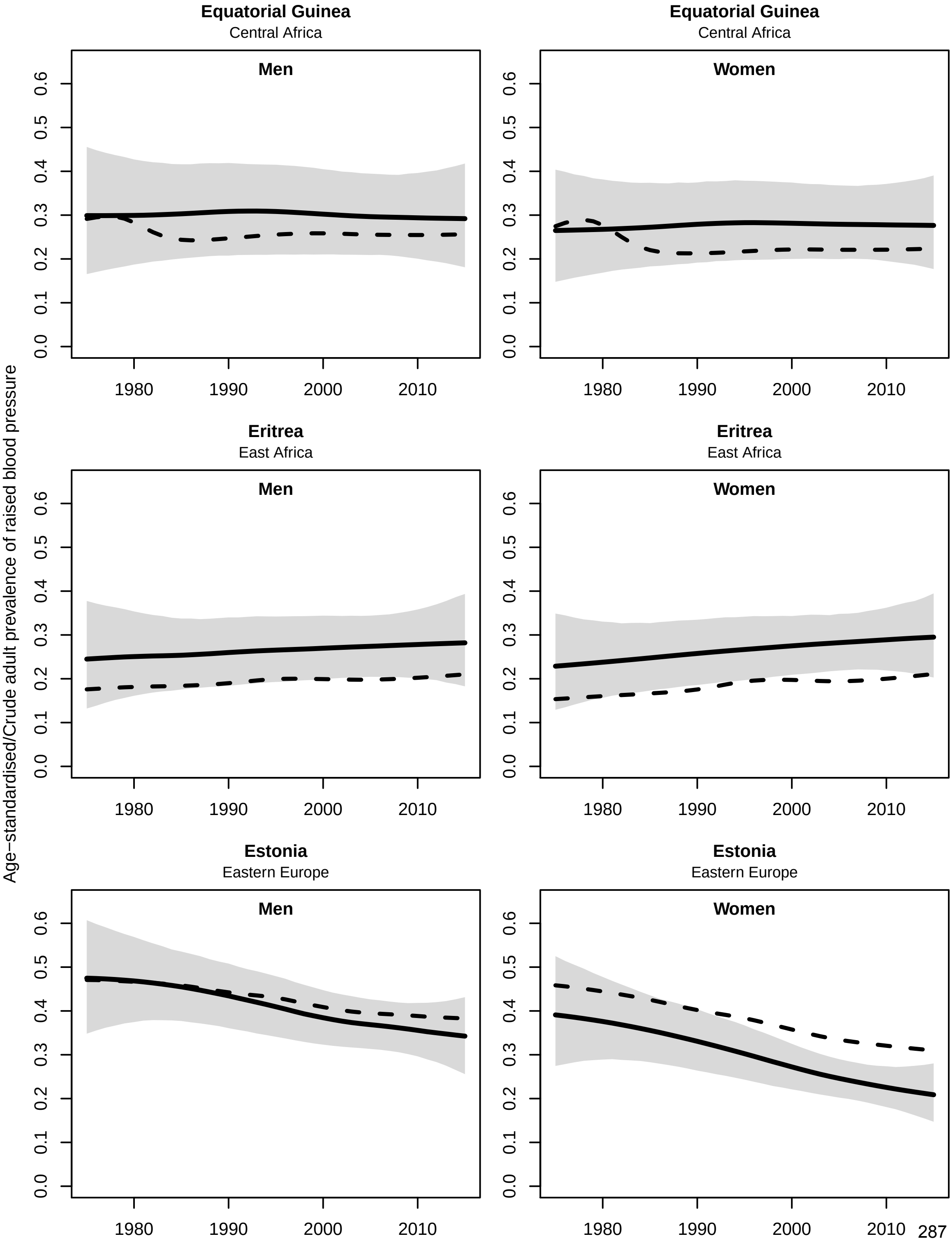
Equatorial Guinea
Central Africa
Men
Women
Eritrea
East Africa
Men
Women
Estonia
Eastern Europe
Men
Women
Age−standardised/Crude adult prevalence of raised blood pressure
0.0
0.1
0.2
0.3
0.4
0.5
0.6
1980
1990
2000
2010

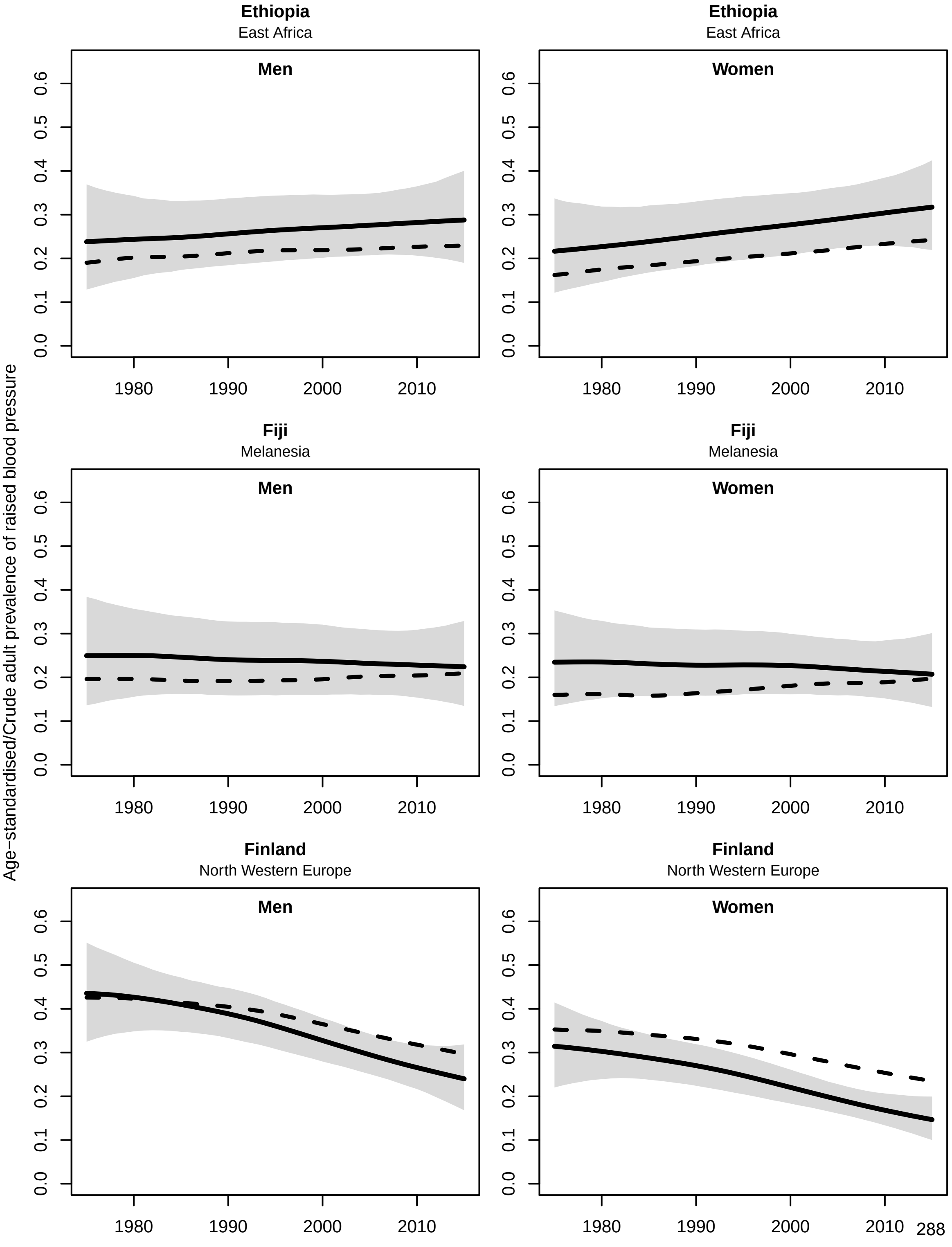
Ethiopia
East Africa
Men
Women
Fiji
Melanesia
Men
Women
Finland
North Western Europe
Men
Women
Age−standardised/Crude adult prevalence of raised blood pressure
0.0
0.1
0.2
0.3
0.4
0.5
0.6
1980
1990
2000
2010

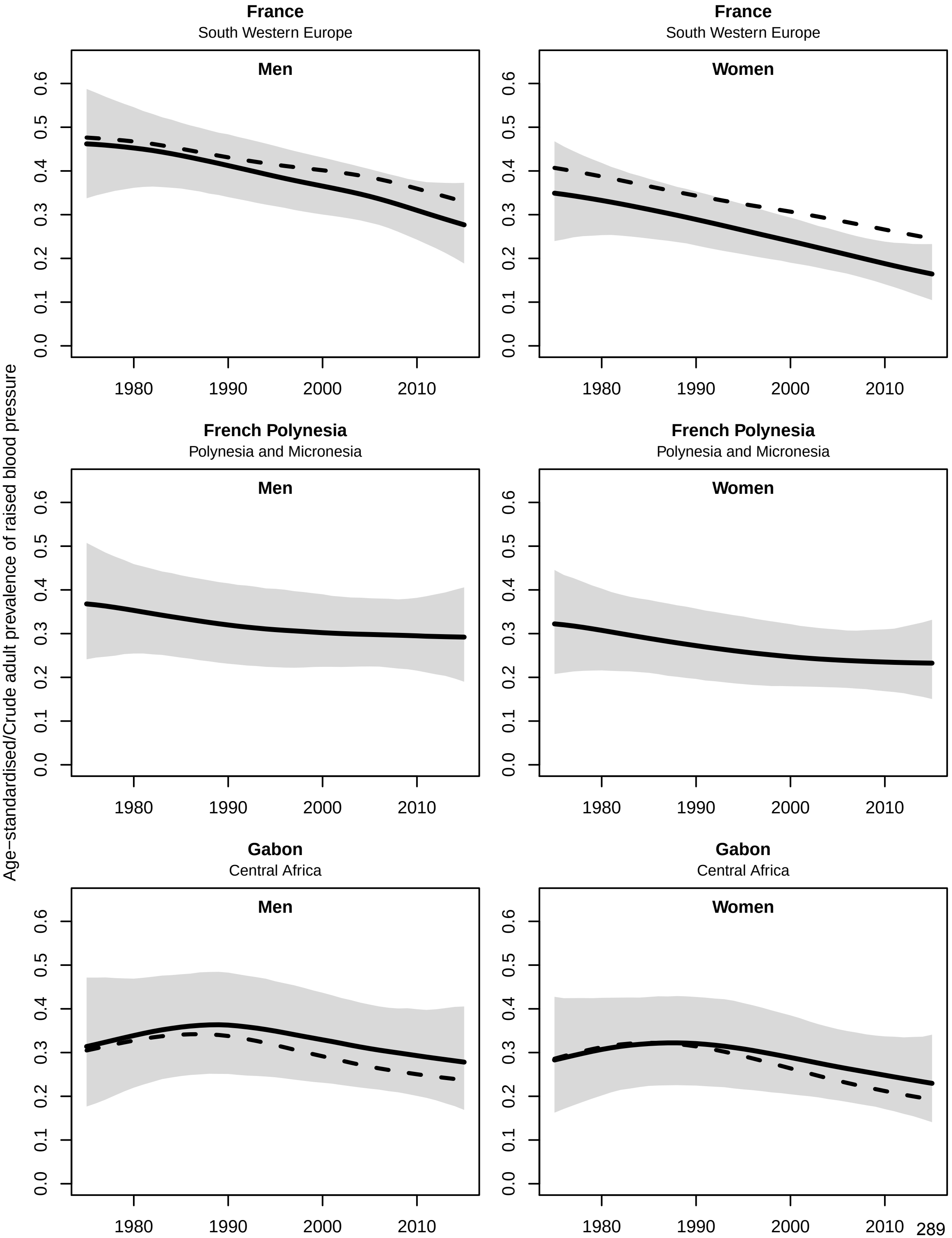
Age−standardised/Crude adult prevalence of raised blood pressure
France
South Western Europe
Men
France
South Western Europe
Women
French Polynesia
Polynesia and Micronesia
Men
French Polynesia
Polynesia and Micronesia
Women
Gabon
Central Africa
Men
Gabon
Central Africa
Women
0.0
0.1
0.2
0.3
0.4
0.5
0.6
1980
1990
2000
2010

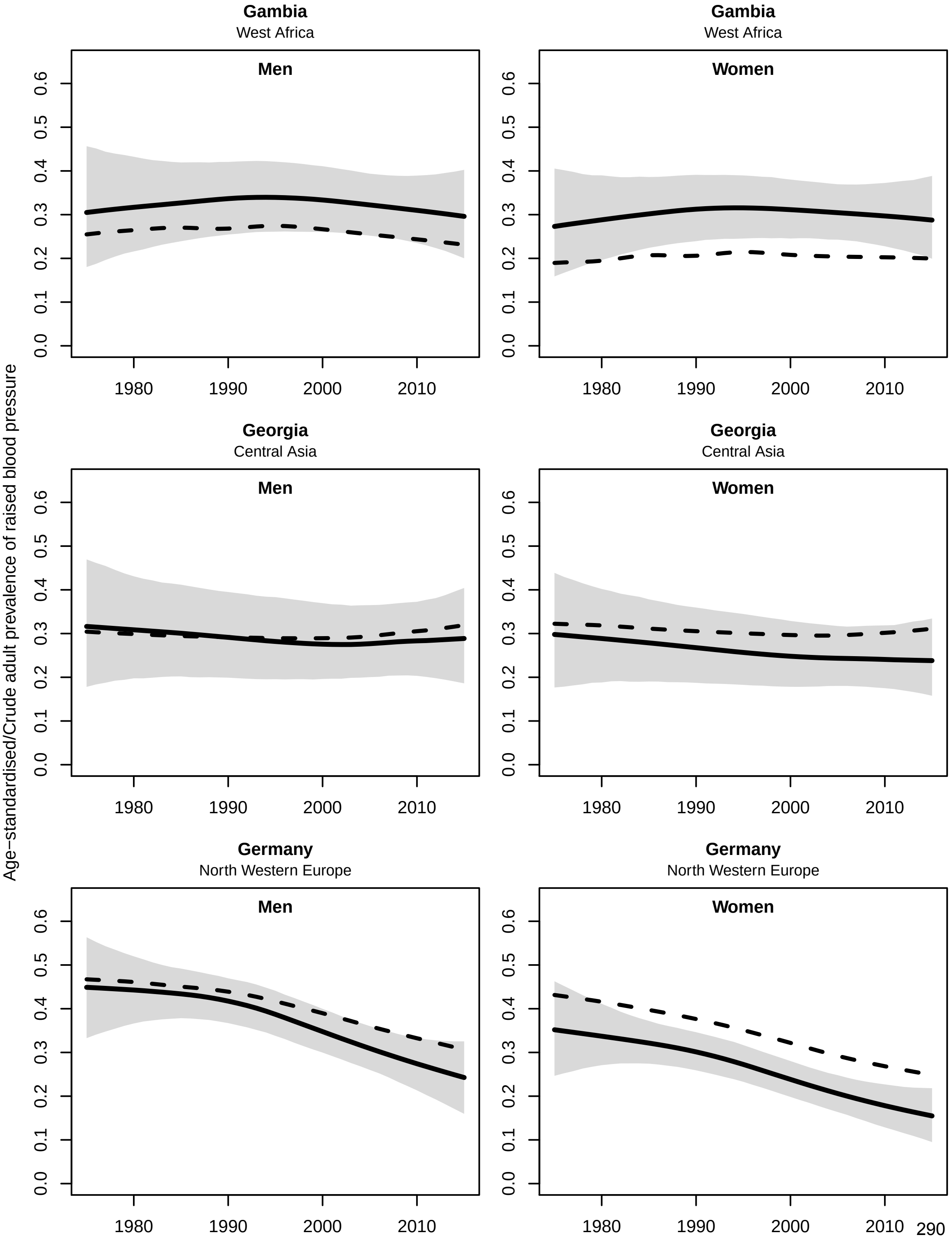
Gambia
West Africa
Men
Gambia
West Africa
Women
Georgia
Central Asia
Men
Georgia
Central Asia
Women
Germany
North Western Europe
Men
Germany
North Western Europe
Women
Age−standardised/Crude adult prevalence of raised blood pressure
0.0
0.1
0.2
0.3
0.4
0.5
0.6
1980
1990
2000
2010

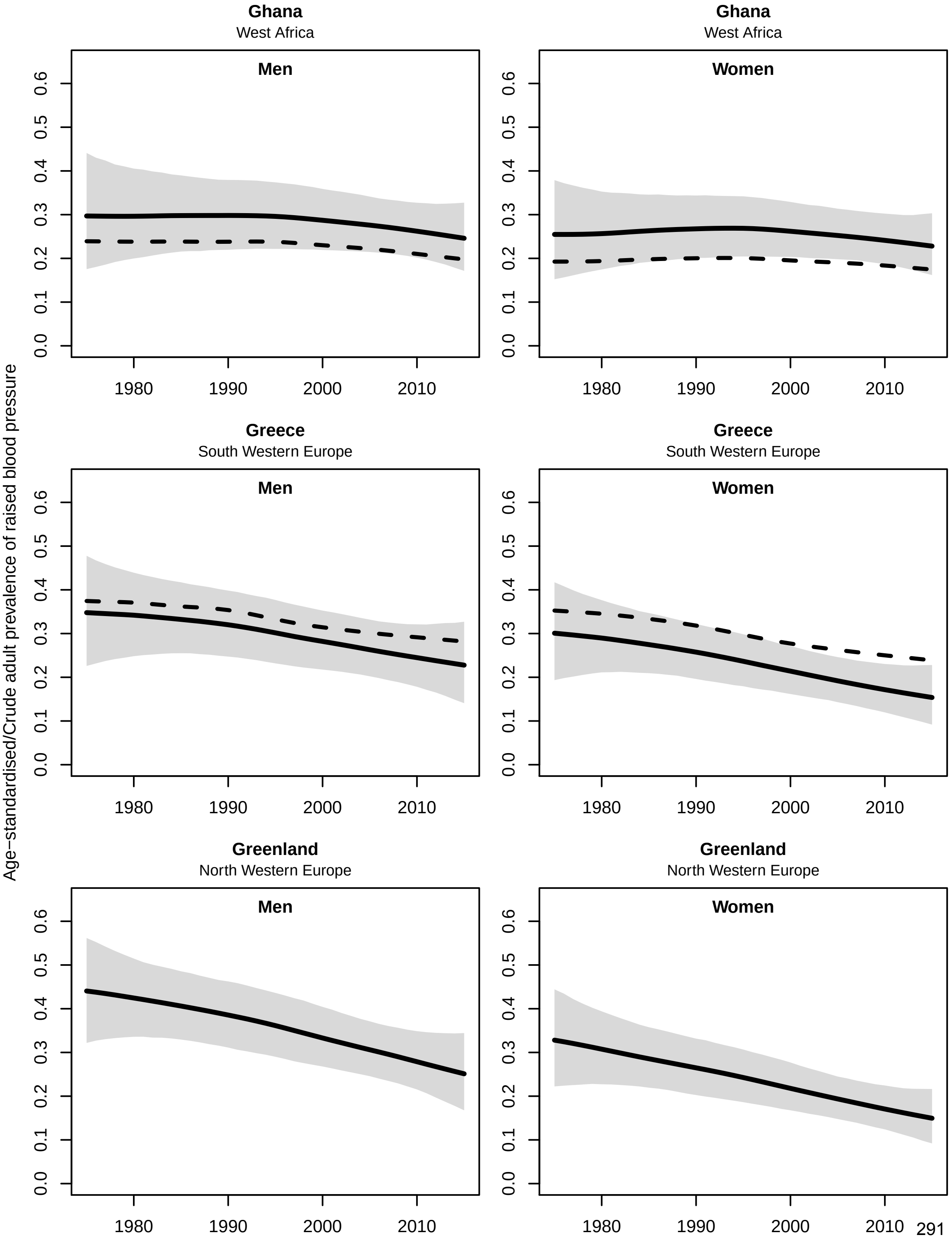
Ghana
West Africa
Men
Ghana
West Africa
Women
Greece
South Western Europe
Men
Greece
South Western Europe
Women
Greenland
North Western Europe
Men
Greenland
North Western Europe
Women
Age−standardised/Crude adult prevalence of raised blood pressure
0.0
0.1
0.2
0.3
0.4
0.5
0.6
1980
1990
2000
2010

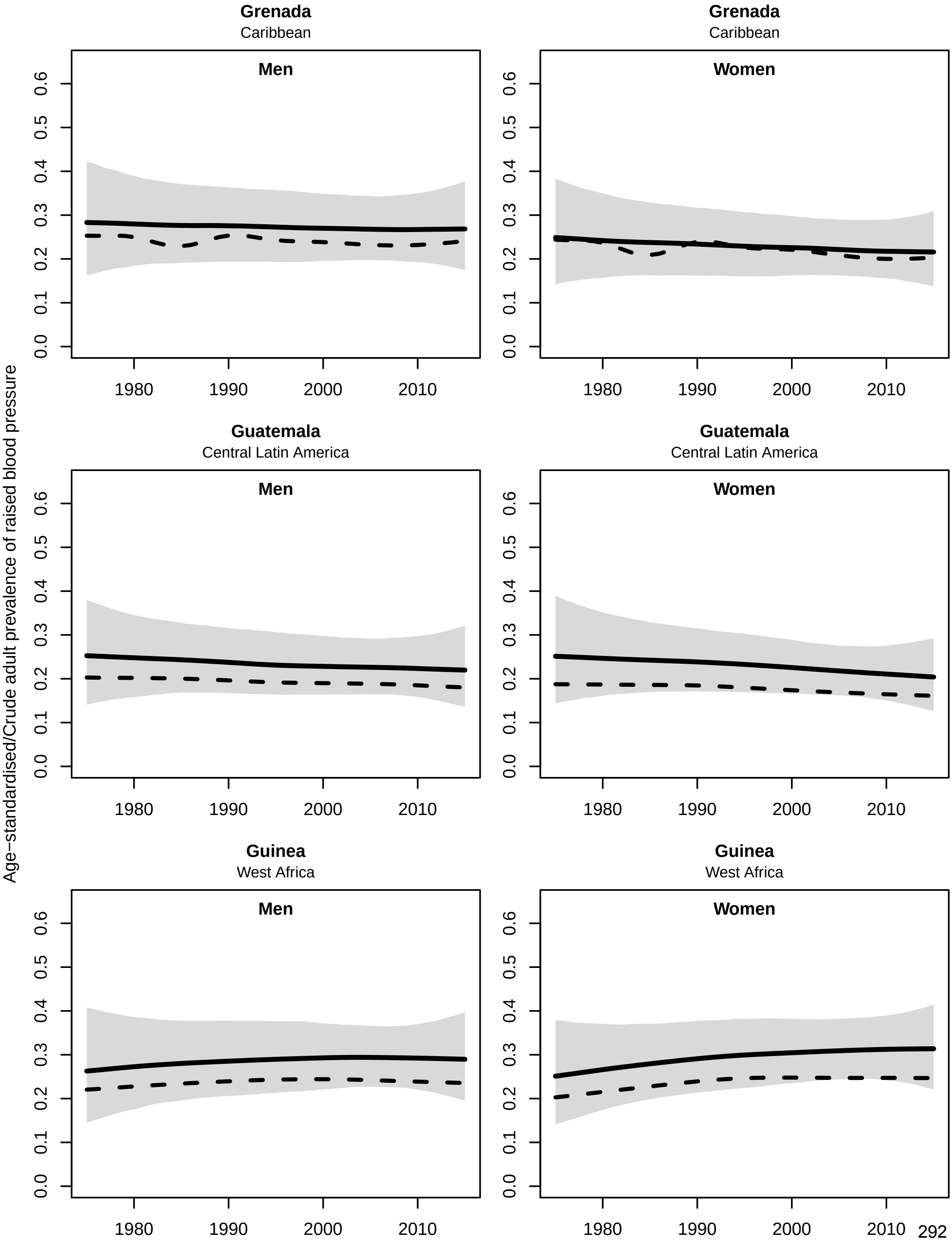
Age−standardised/Crude adult prevalence of raised blood pressure
Grenada
Caribbean
Men
Women
Guatemala
Central Latin America
Guinea
West Africa
0.0
0.1
0.2
0.3
0.4
0.5
0.6
1980
1990
2000
2010

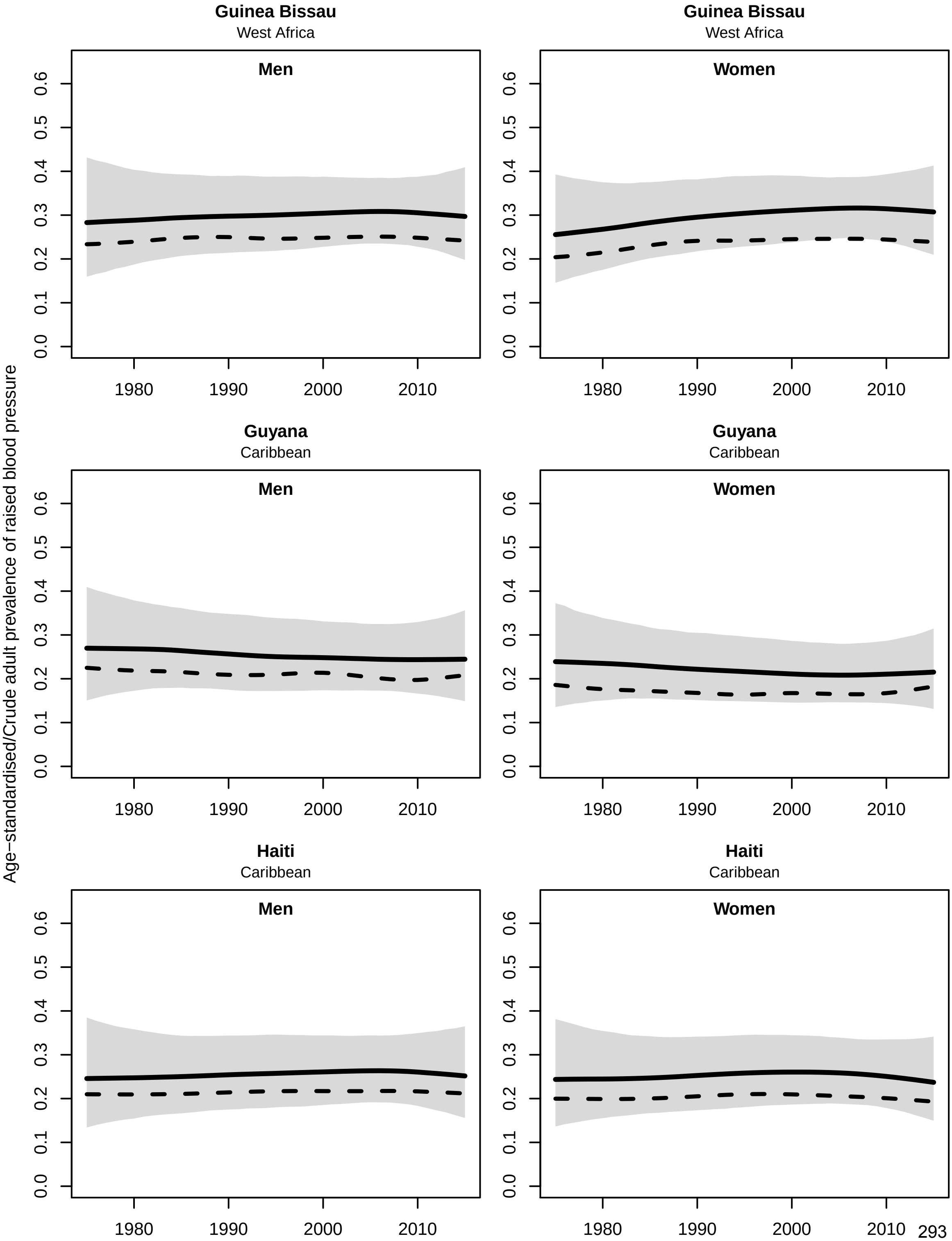
Guinea Bissau
West Africa
Men
Guinea Bissau
West Africa
Women
Guyana
Caribbean
Men
Guyana
Caribbean
Women
Haiti
Caribbean
Men
Haiti
Caribbean
Women
Age−standardised/Crude adult prevalence of raised blood pressure
0.0
0.1
0.2
0.3
0.4
0.5
0.6
1980
1990
2000
2010

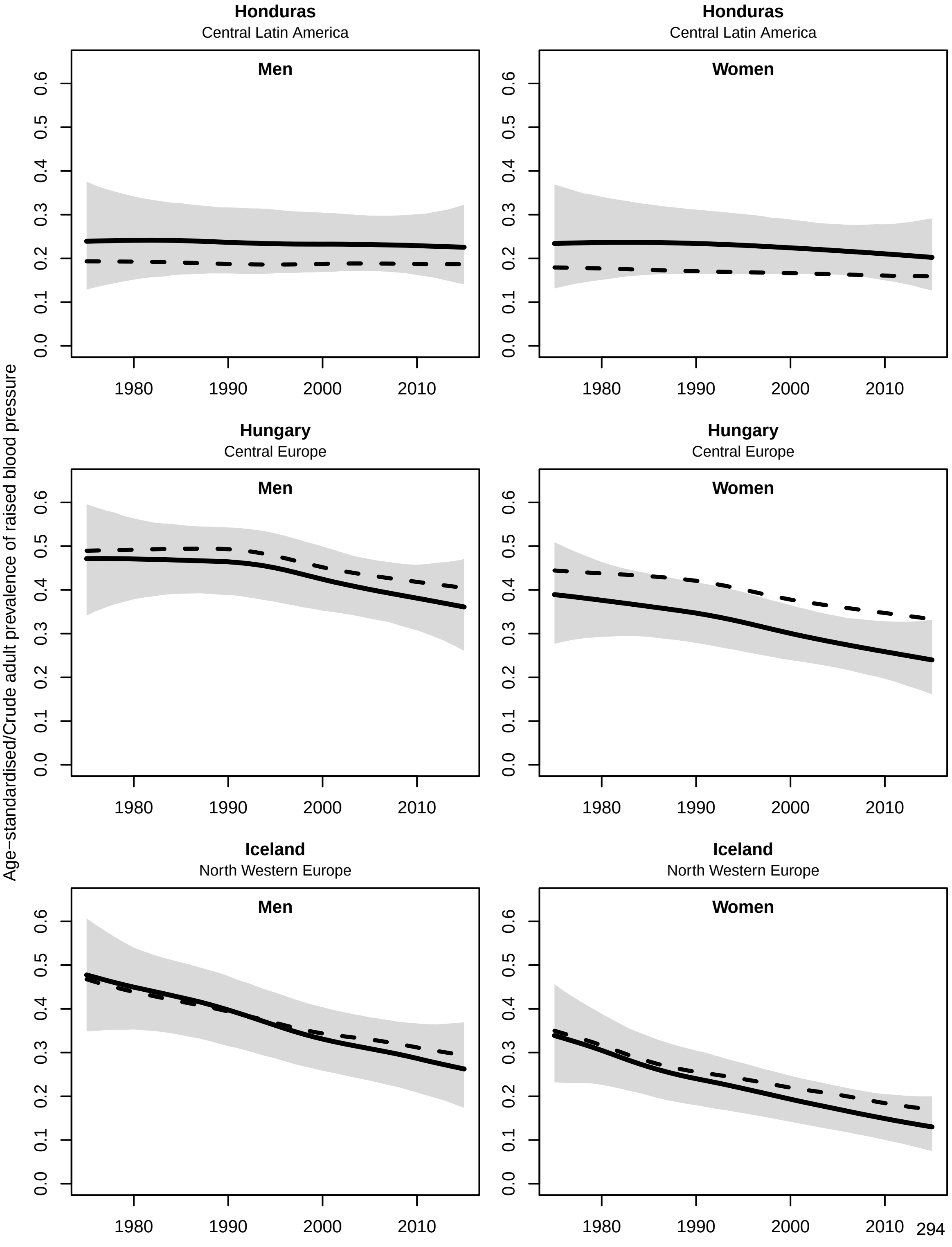
Honduras
Central Latin America
Men
Honduras
Central Latin America
Women
Hungary
Central Europe
Men
Hungary
Central Europe
Women
Iceland
North Western Europe
Men
Iceland
North Western Europe
Women
Age−standardised/Crude adult prevalence of raised blood pressure
0.0
0.1
0.2
0.3
0.4
0.5
0.6
1980
1990
2000
2010

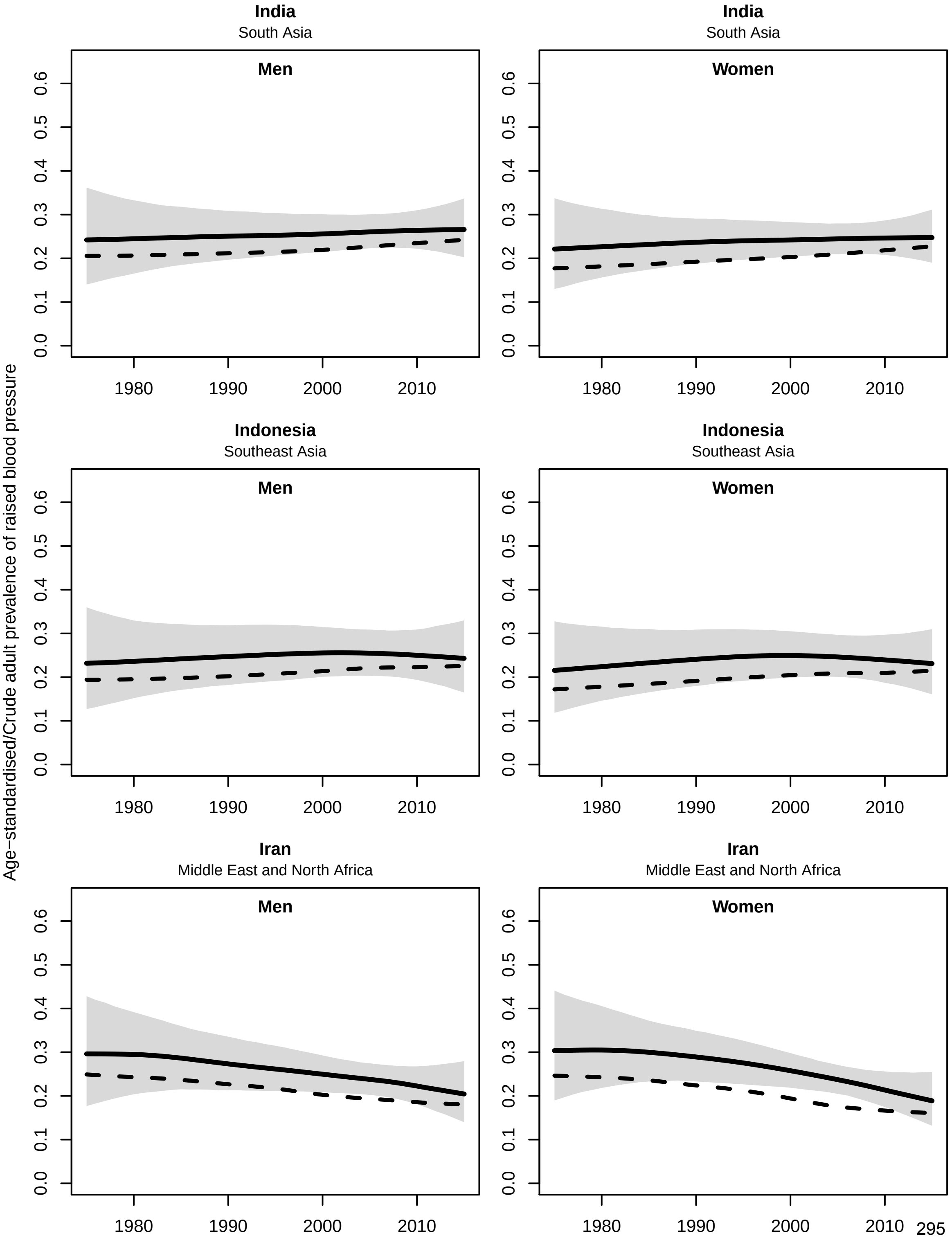

India
South Asia
Men
Women
Indonesia
Southeast Asia
Iran
Middle East and North Africa
Age−standardised/Crude adult prevalence of raised blood pressure
0.0
0.1
0.2
0.3
0.4
0.5
0.6
1980
1990
2000
2010

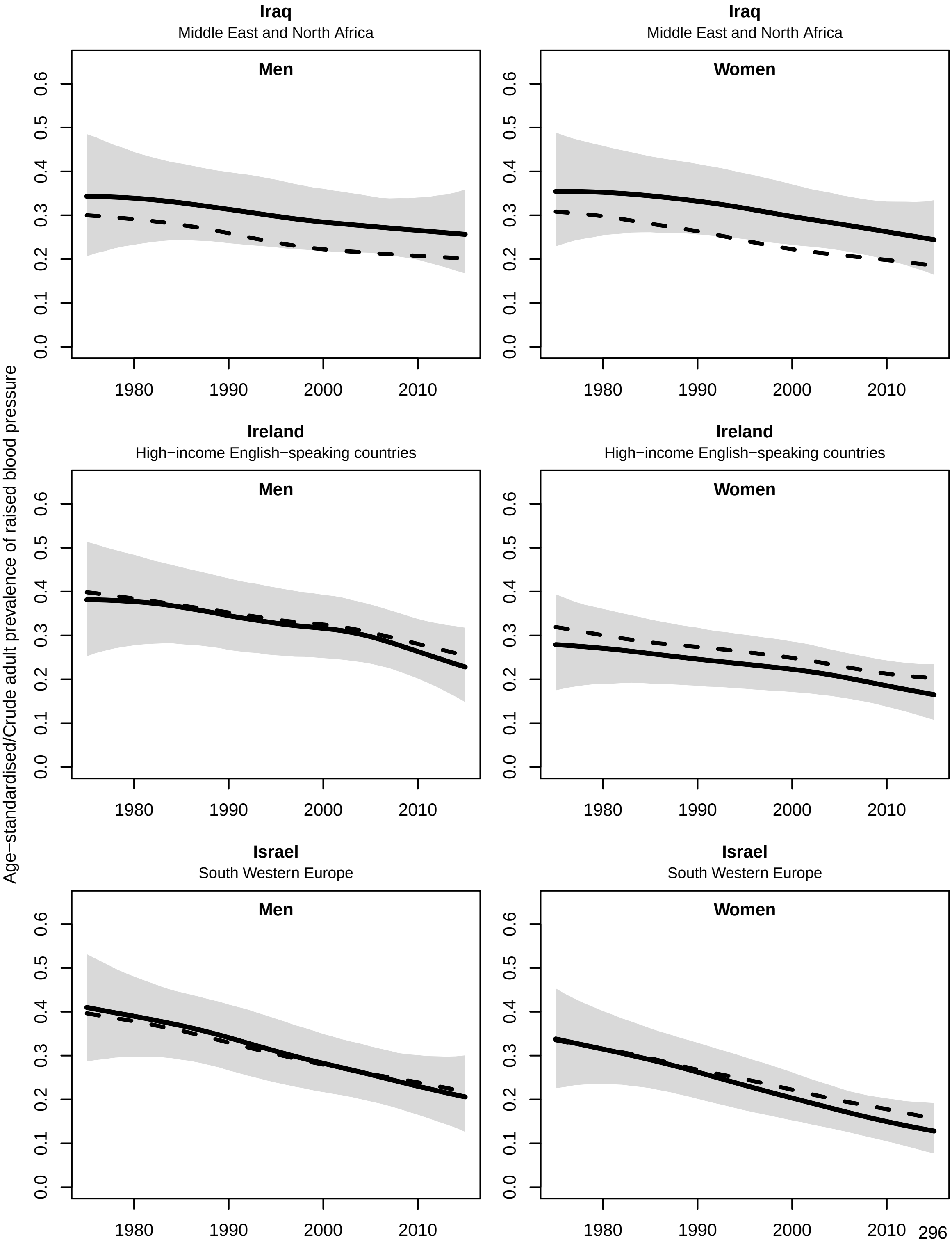
Iraq
Middle East and North Africa
Men
Iraq
Middle East and North Africa
Women
Ireland
High−income English−speaking countries
Men
Ireland
High−income English−speaking countries
Women
Israel
South Western Europe
Men
Israel
South Western Europe
Women
Age−standardised/Crude adult prevalence of raised blood pressure
0.0
0.1
0.2
0.3
0.4
0.5
0.6
1980
1990
2000
2010

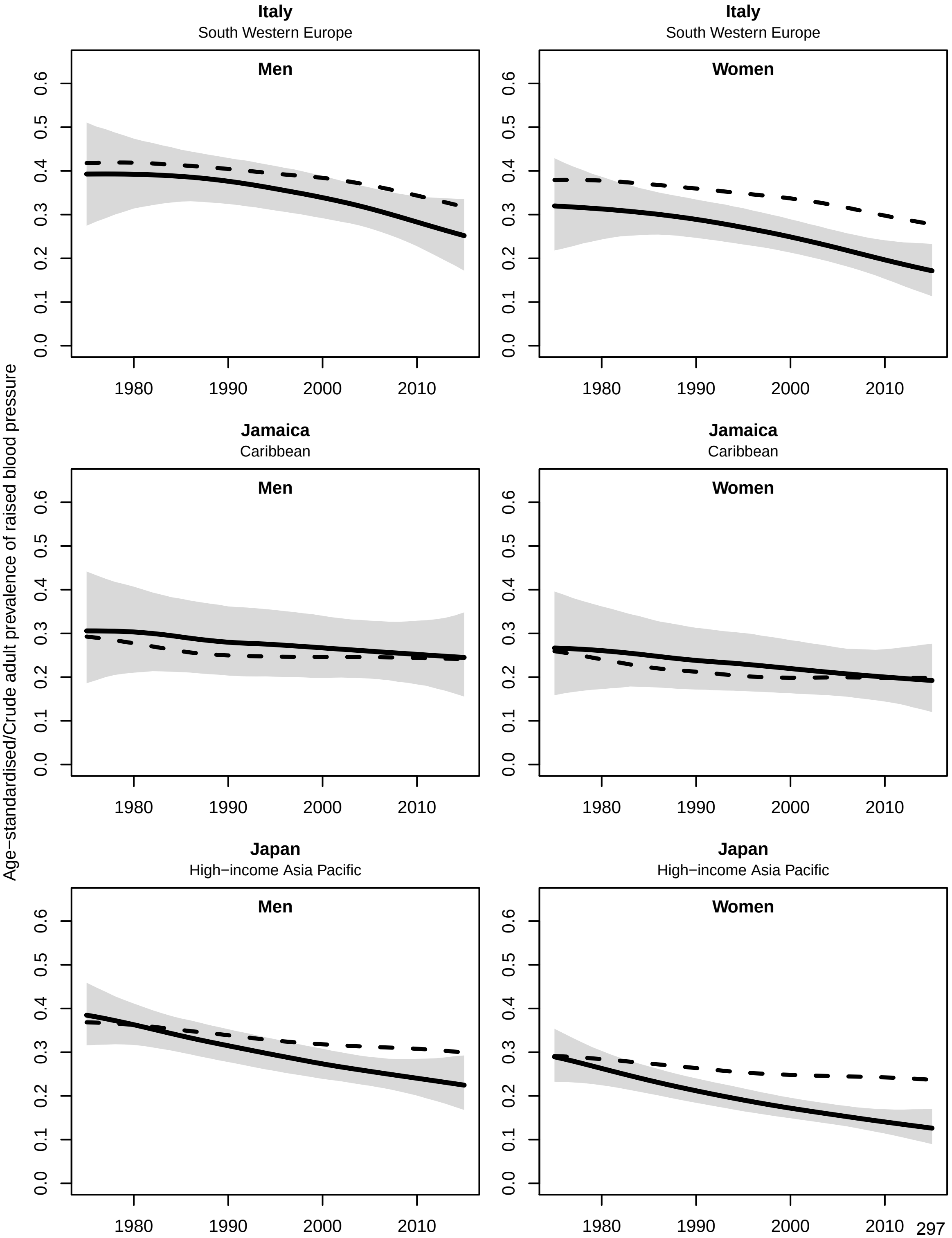
Italy
South Western Europe
Men
Italy
South Western Europe
Women
Jamaica
Caribbean
Men
Jamaica
Caribbean
Women
Japan
High−income Asia Pacific
Men
Japan
High−income Asia Pacific
Women
Age−standardised/Crude adult prevalence of raised blood pressure
0.0
0.1
0.2
0.3
0.4
0.5
0.6
1980
1990
2000
2010

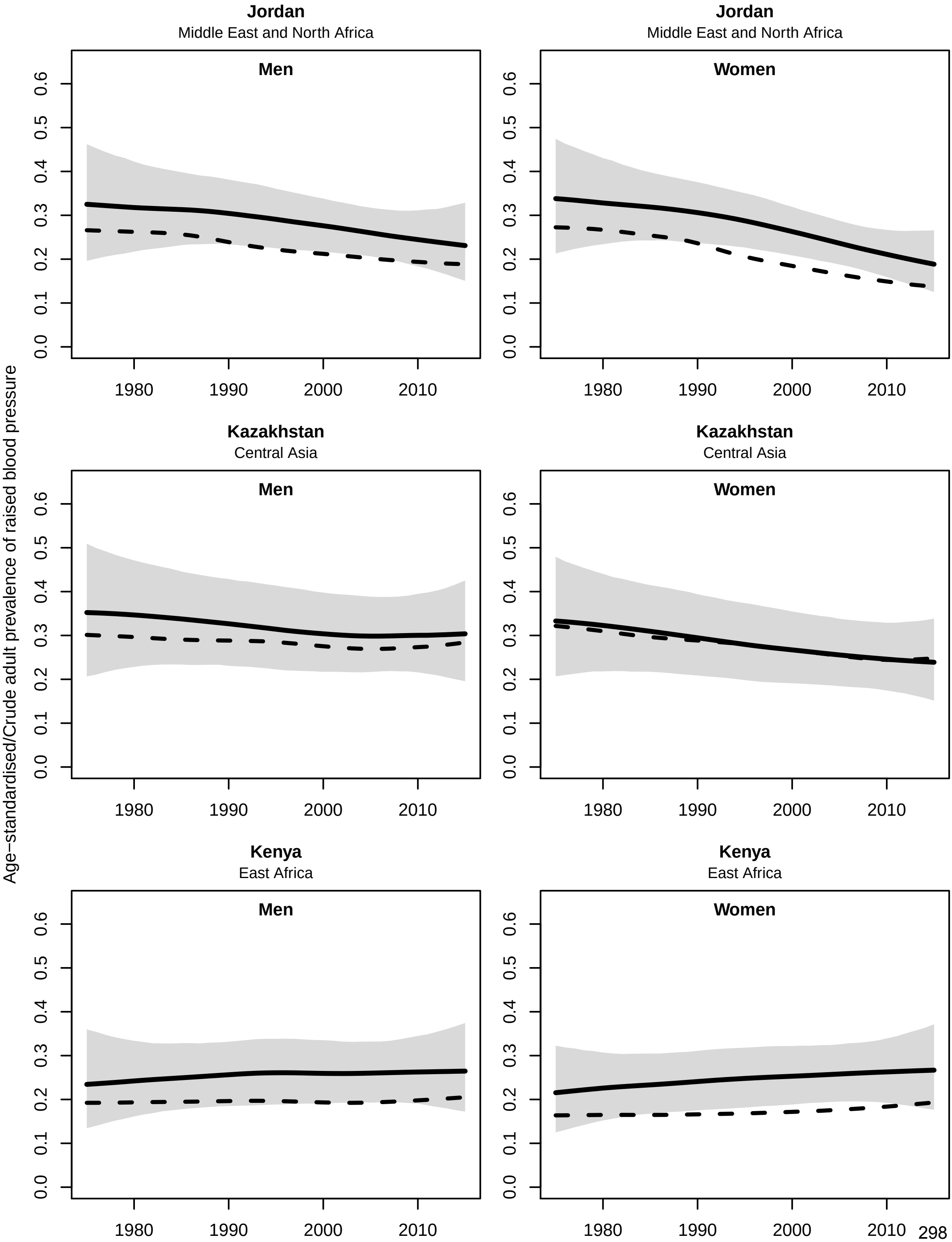
Jordan
Middle East and North Africa
Men
Jordan
Middle East and North Africa
Women
Kazakhstan
Central Asia
Men
Kazakhstan
Central Asia
Women
Kenya
East Africa
Men
Kenya
East Africa
Women
Age−standardised/Crude adult prevalence of raised blood pressure
0.0
0.1
0.2
0.3
0.4
0.5
0.6
1980
1990
2000
2010

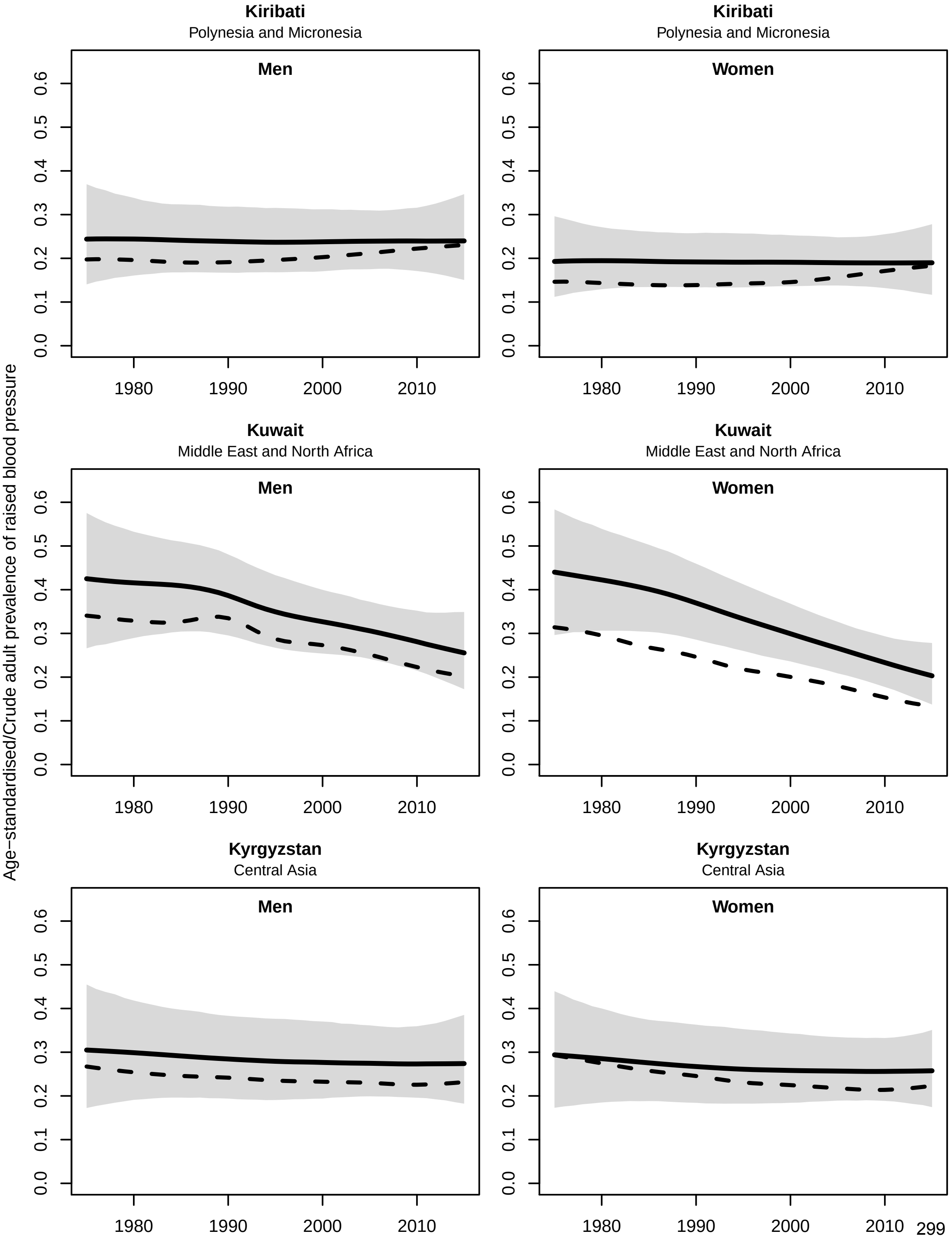
Kiribati
Polynesia and Micronesia
Men
Women
Kuwait
Middle East and North Africa
Kyrgyzstan
Central Asia
Age−standardised/Crude adult prevalence of raised blood pressure
0.0
0.1
0.2
0.3
0.4
0.5
0.6
1980
1990
2000
2010

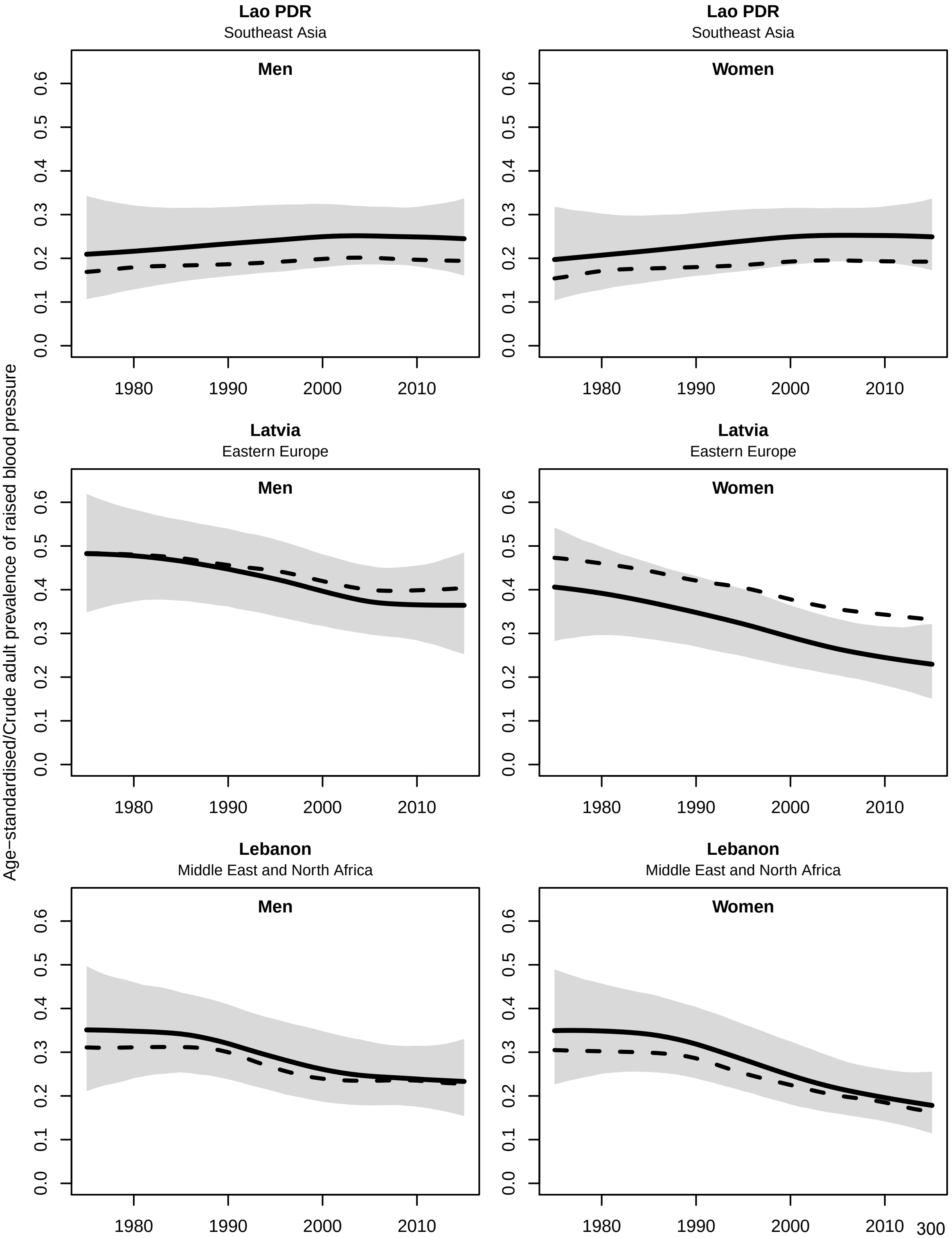
Lao PDR
Southeast Asia
Men
Lao PDR
Southeast Asia
Women
Latvia
Eastern Europe
Men
Latvia
Eastern Europe
Women
Lebanon
Middle East and North Africa
Men
Lebanon
Middle East and North Africa
Women
Age−standardised/Crude adult prevalence of raised blood pressure
0.0
0.1
0.2
0.3
0.4
0.5
0.6
1980
1990
2000
2010

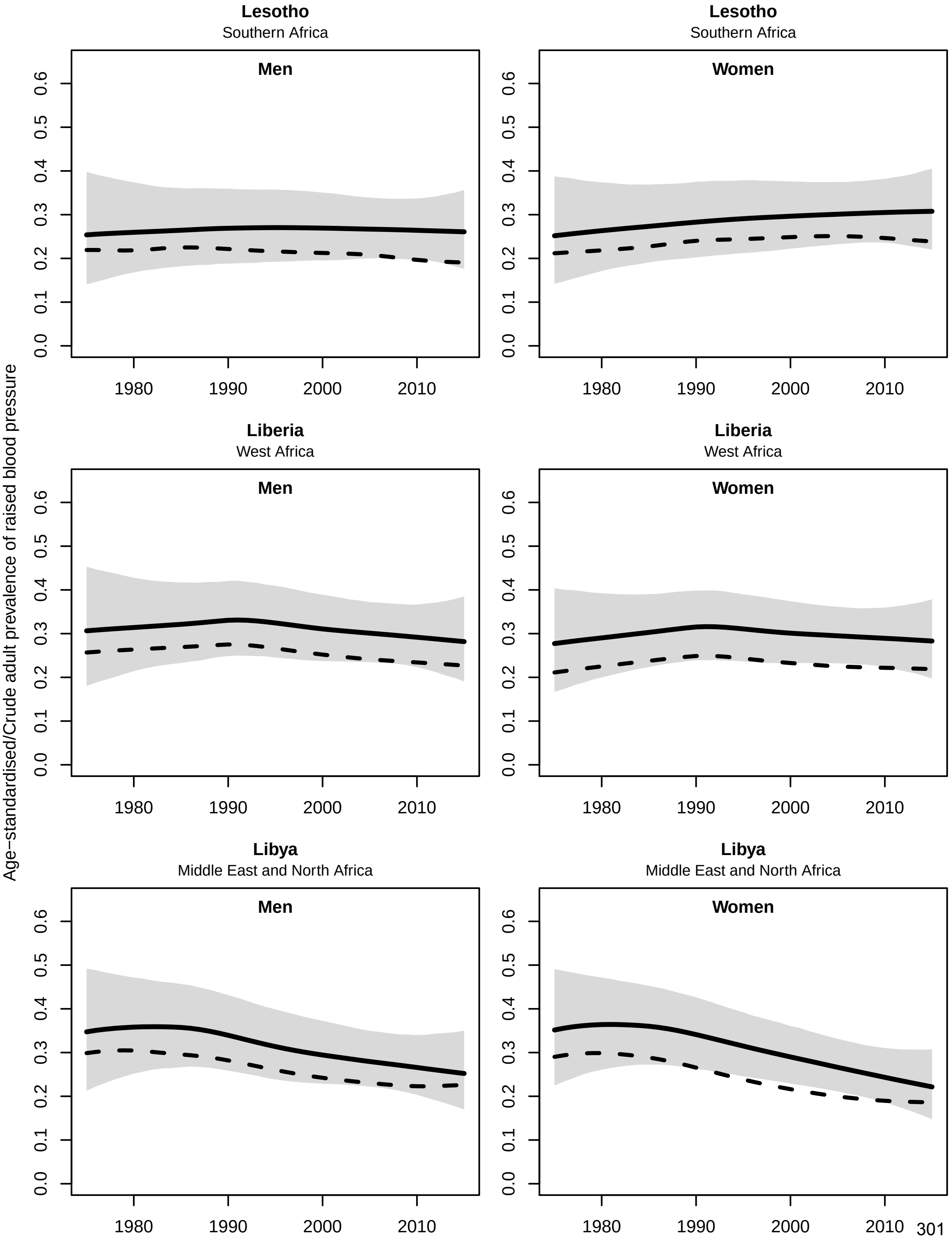
Lesotho
Southern Africa
Men
Lesotho
Southern Africa
Women
Liberia
West Africa
Men
Liberia
West Africa
Women
Libya
Middle East and North Africa
Men
Libya
Middle East and North Africa
Women
Age−standardised/Crude adult prevalence of raised blood pressure
0.0
0.1
0.2
0.3
0.4
0.5
0.6
1980
1990
2000
2010

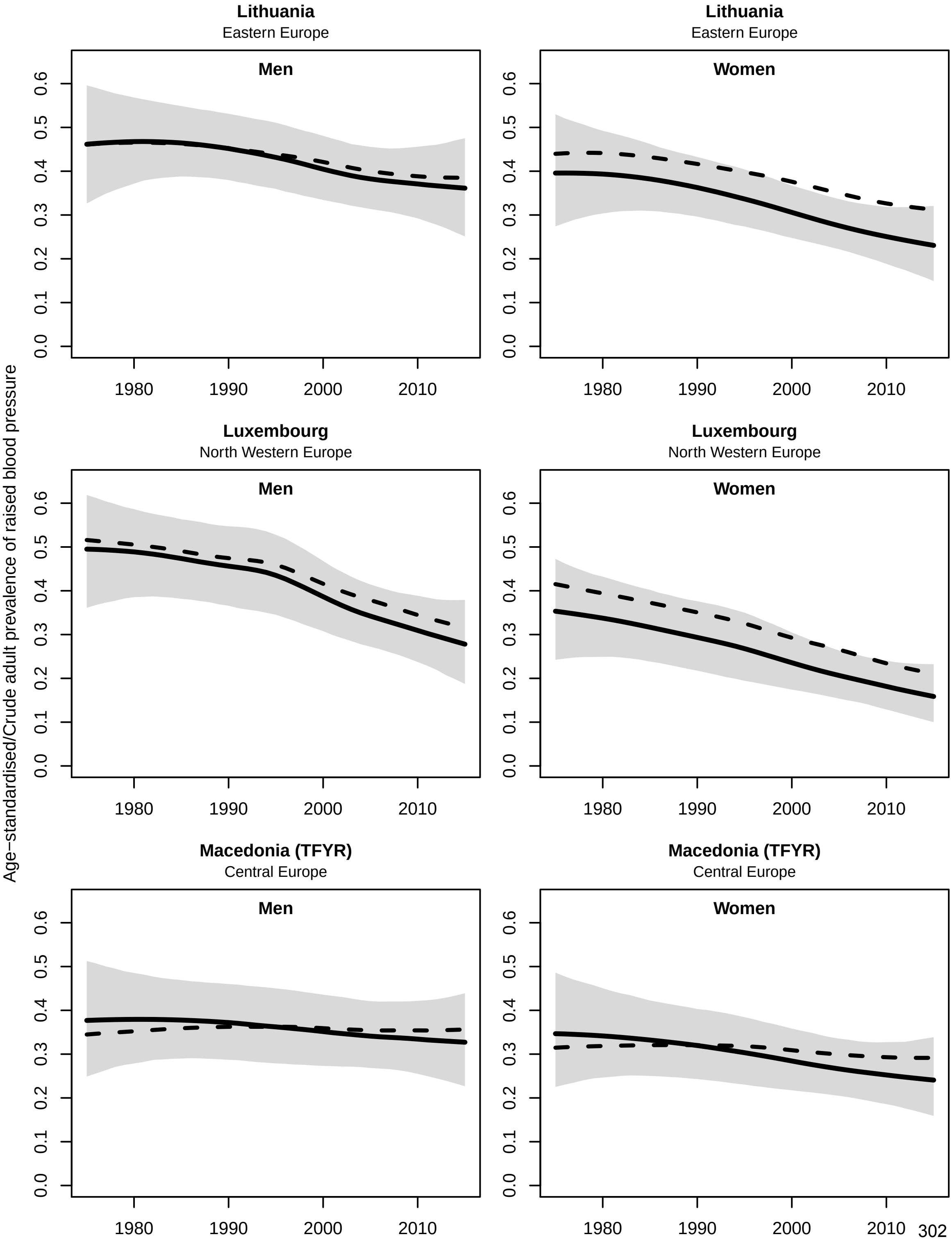
Lithuania
Eastern Europe
Men
Women
Luxembourg
North Western Europe
Men
Women
Macedonia (TFYR)
Central Europe
Men
Women
Age−standardised/Crude adult prevalence of raised blood pressure
0.0
0.1
0.2
0.3
0.4
0.5
0.6
1980
1990
2000
2010

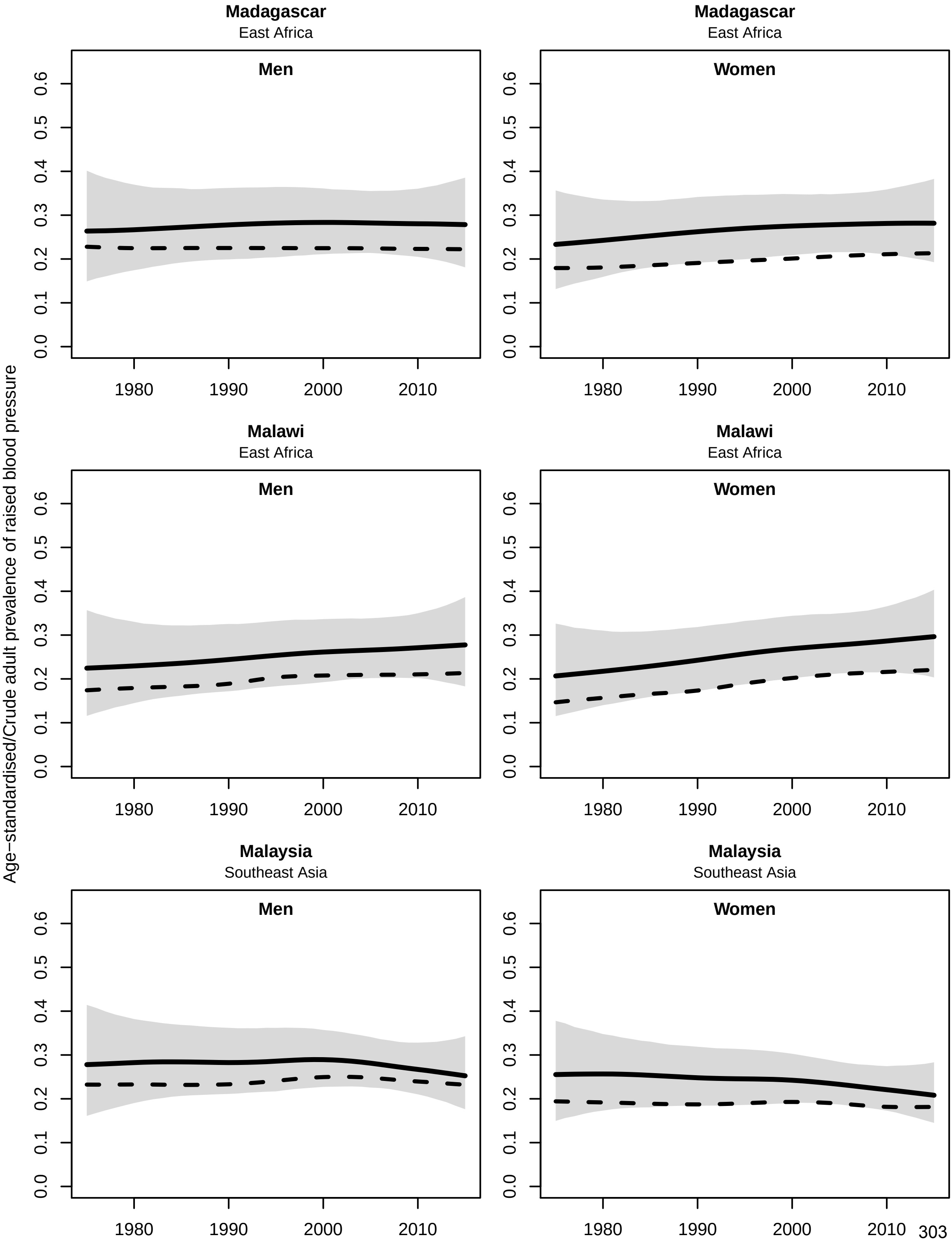
Age−standardised/Crude adult prevalence of raised blood pressure
Madagascar
East Africa
Men
Women
Malawi
East Africa
Malaysia
Southeast Asia
0.0
0.1
0.2
0.3
0.4
0.5
0.6
1980
1990
2000
2010

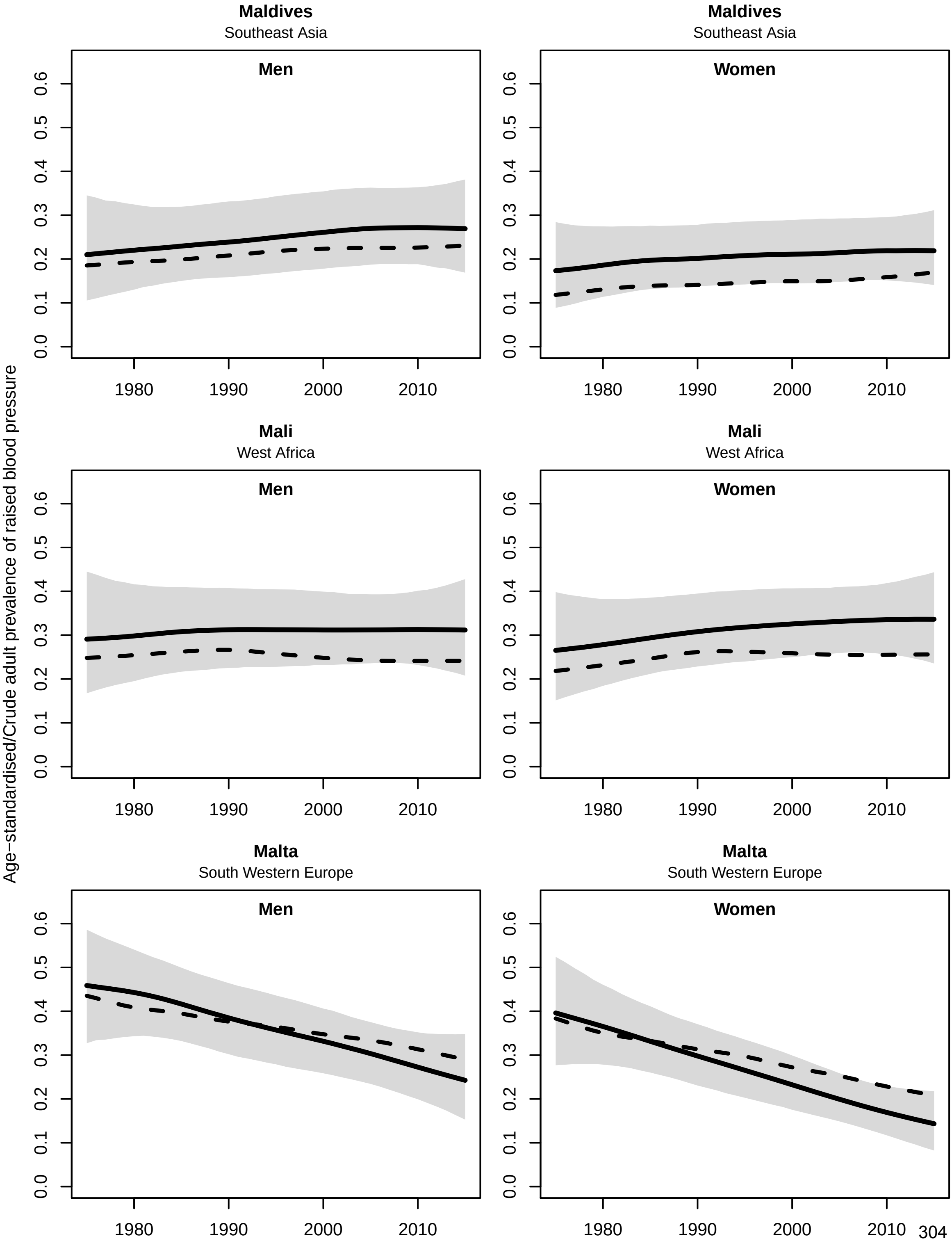
Age−standardised/Crude adult prevalence of raised blood pressure
Maldives
Southeast Asia
Men
Maldives
Southeast Asia
Women
Mali
West Africa
Men
Mali
West Africa
Women
Malta
South Western Europe
Men
Malta
South Western Europe
Women
0.0
0.1
0.2
0.3
0.4
0.5
0.6
1980
1990
2000
2010

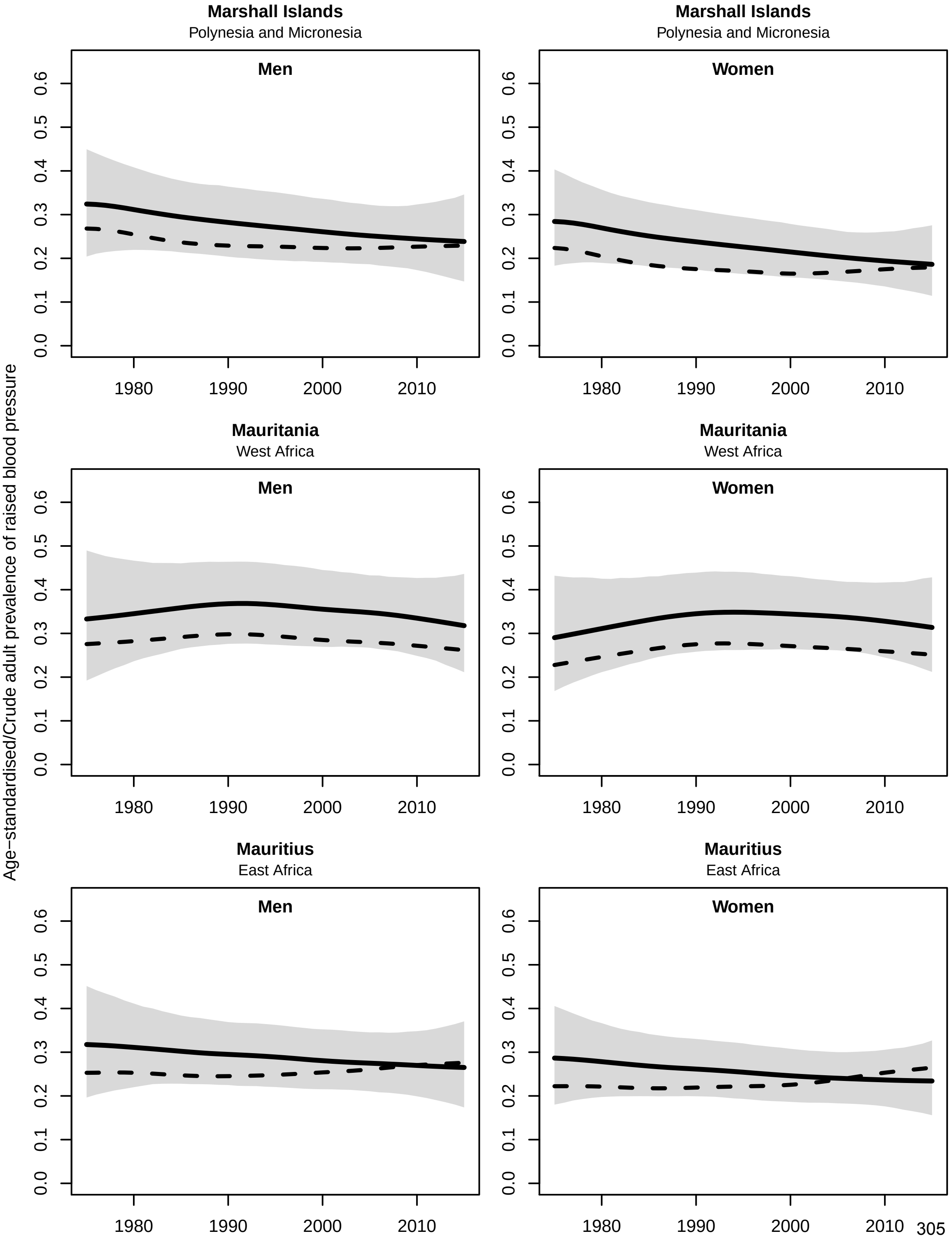
Marshall Islands
Polynesia and Micronesia
Men
Women
Mauritania
West Africa
Mauritius
East Africa
Age−standardised/Crude adult prevalence of raised blood pressure
0.0
0.1
0.2
0.3
0.4
0.5
0.6
1980
1990
2000
2010

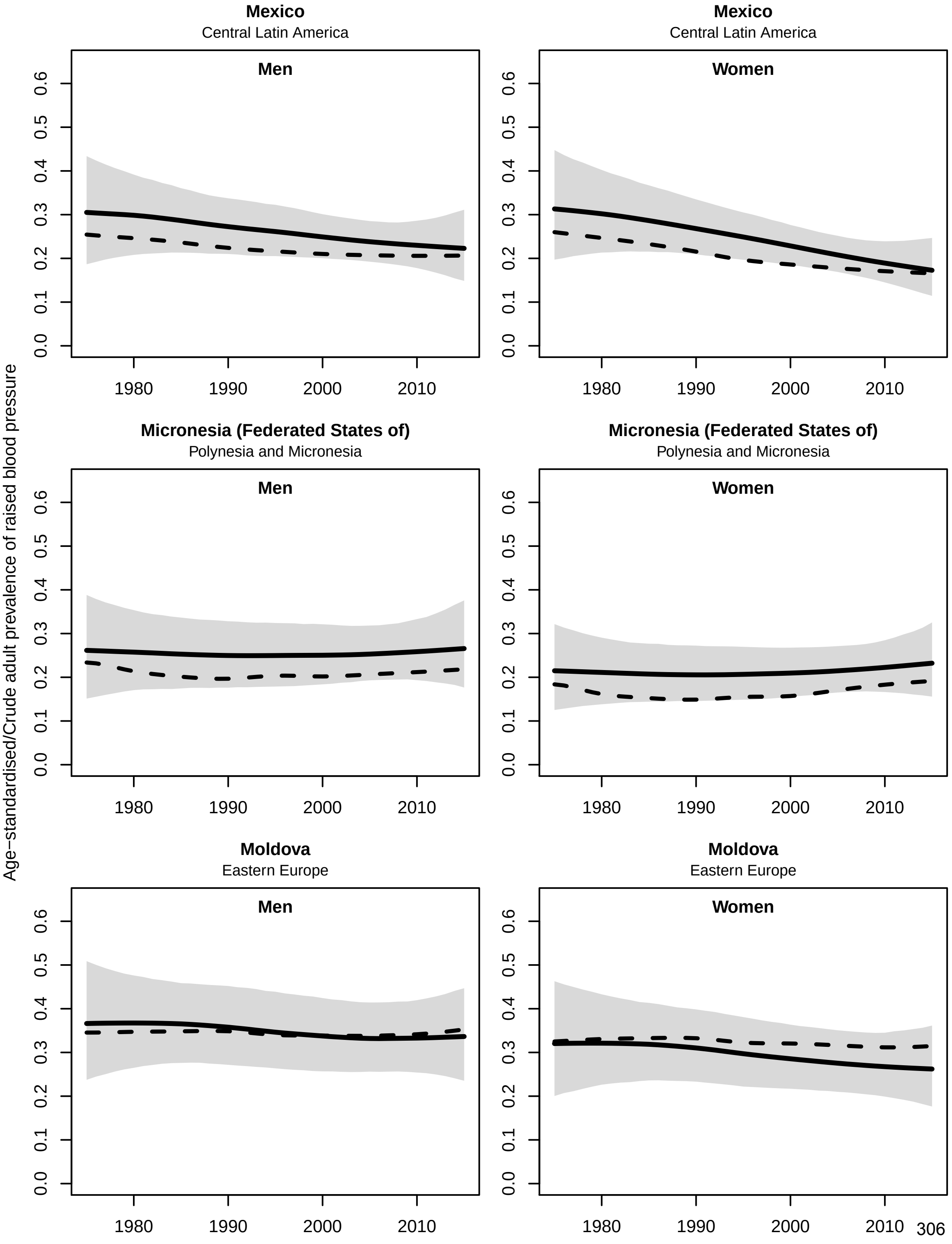
Mexico
Central Latin America
Men
Mexico
Central Latin America
Women
Micronesia (Federated States of)
Polynesia and Micronesia
Men
Micronesia (Federated States of)
Polynesia and Micronesia
Women
Moldova
Eastern Europe
Men
Moldova
Eastern Europe
Women
Age−standardised/Crude adult prevalence of raised blood pressure
0.0
0.1
0.2
0.3
0.4
0.5
0.6
1980
1990
2000
2010

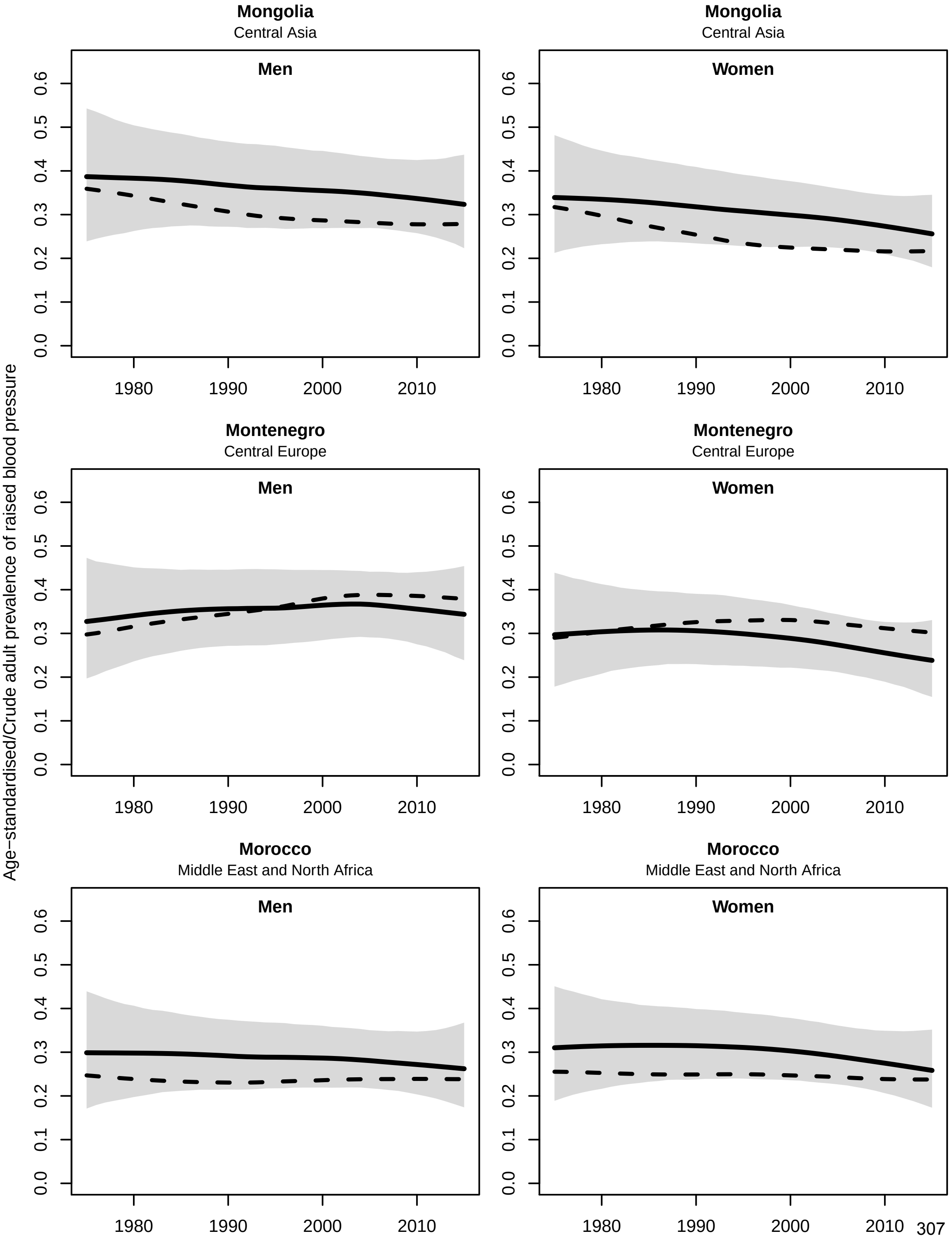
Mongolia
Central Asia
Men
Mongolia
Central Asia
Women
Montenegro
Central Europe
Men
Montenegro
Central Europe
Women
Morocco
Middle East and North Africa
Men
Morocco
Middle East and North Africa
Women
Age−standardised/Crude adult prevalence of raised blood pressure
0.0
0.1
0.2
0.3
0.4
0.5
0.6
1980
1990
2000
2010

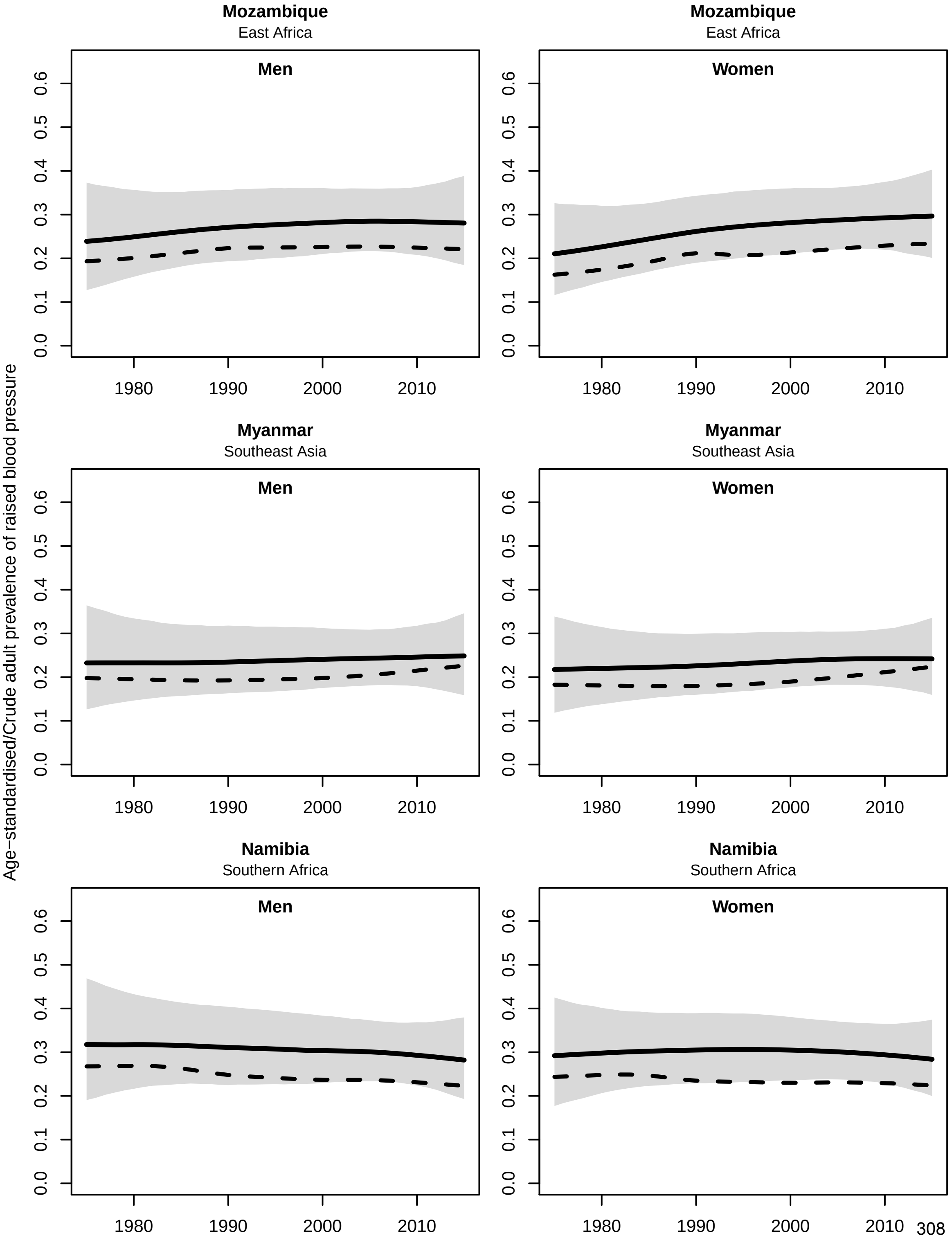
Mozambique
East Africa
Men
Mozambique
East Africa
Women
Myanmar
Southeast Asia
Men
Myanmar
Southeast Asia
Women
Namibia
Southern Africa
Men
Namibia
Southern Africa
Women
Age−standardised/Crude adult prevalence of raised blood pressure
0.0
0.1
0.2
0.3
0.4
0.5
0.6
1980
1990
2000
2010

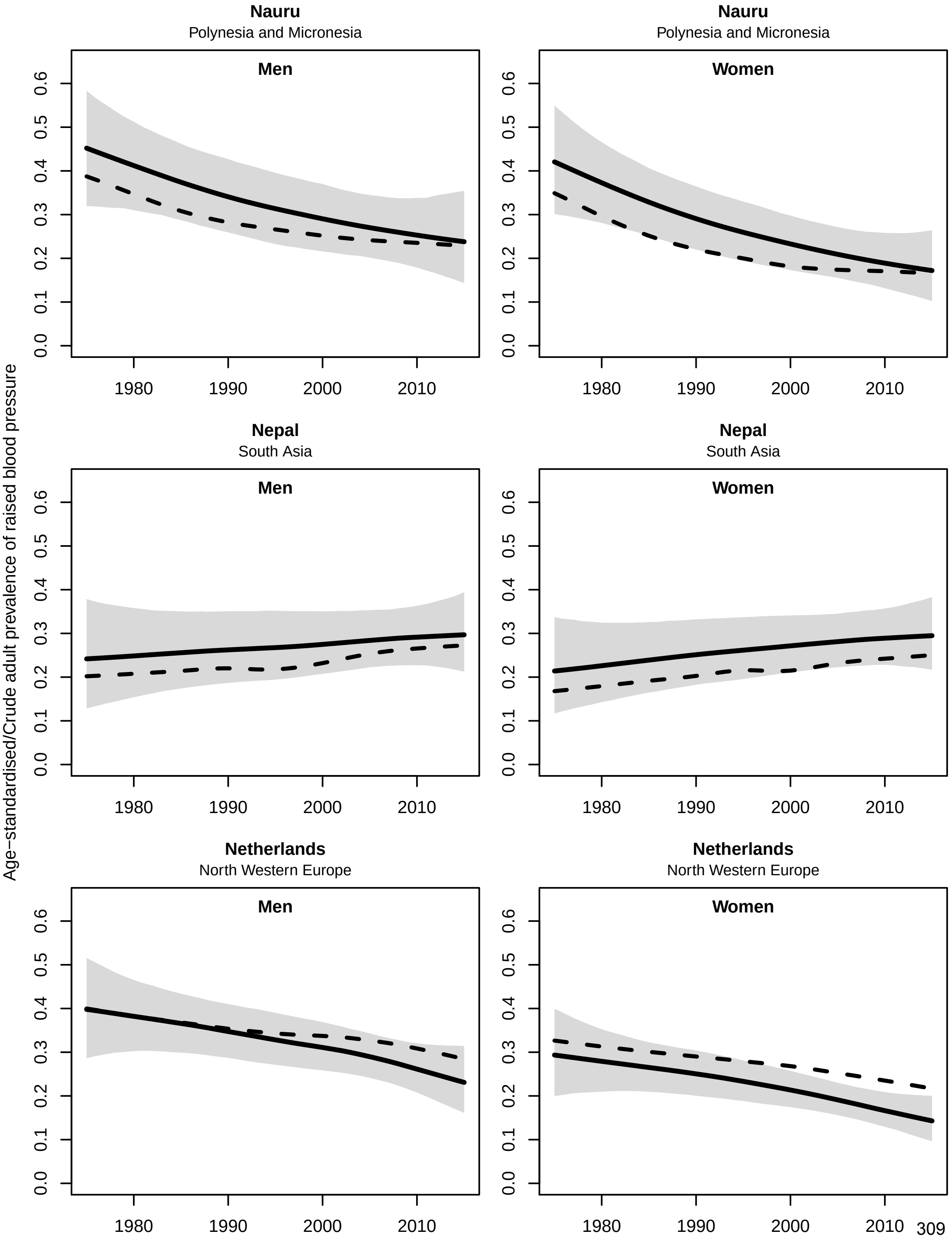
Nauru
Polynesia and Micronesia
Men
Nauru
Polynesia and Micronesia
Women
Nepal
South Asia
Men
Nepal
South Asia
Women
Netherlands
North Western Europe
Men
Netherlands
North Western Europe
Women
Age−standardised/Crude adult prevalence of raised blood pressure
0.0
0.1
0.2
0.3
0.4
0.5
0.6
1980
1990
2000
2010

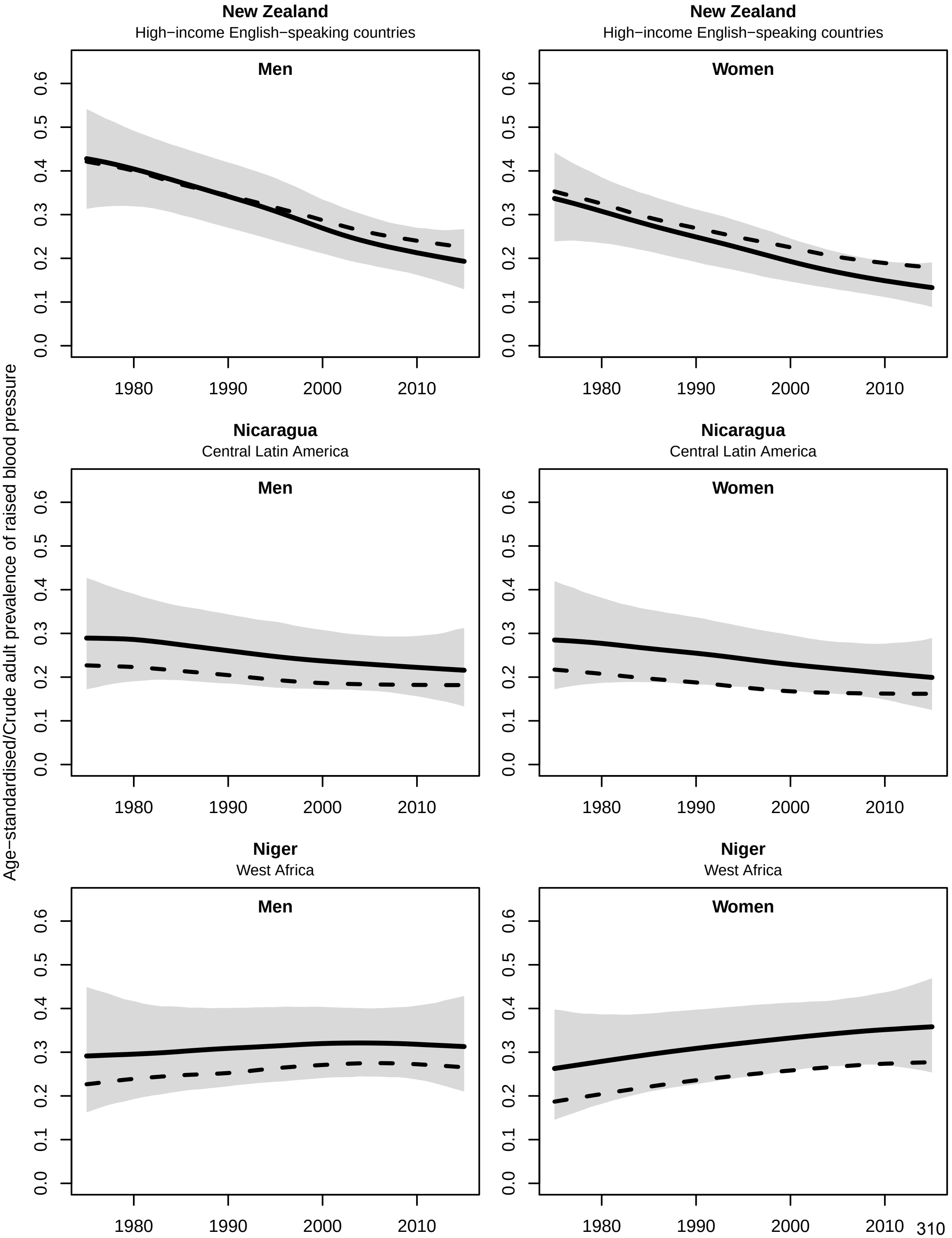
New Zealand
High−income English−speaking countries
Men
Women
Nicaragua
Central Latin America
Niger
West Africa
Age−standardised/Crude adult prevalence of raised blood pressure
0.0
0.1
0.2
0.3
0.4
0.5
0.6
1980
1990
2000
2010

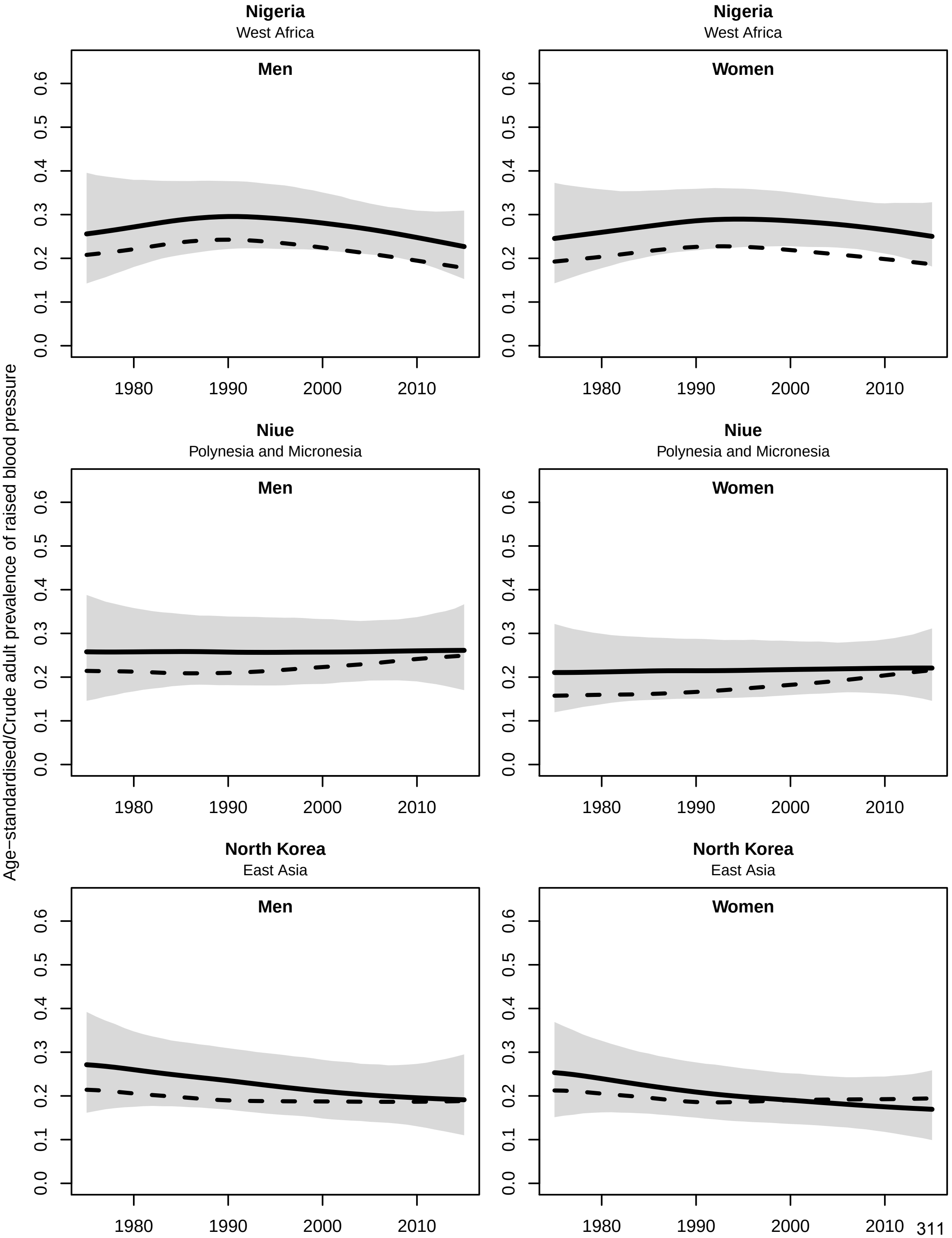
Nigeria
West Africa
Men
Nigeria
West Africa
Women
Niue
Polynesia and Micronesia
Men
Niue
Polynesia and Micronesia
Women
North Korea
East Asia
Men
North Korea
East Asia
Women
Age−standardised/Crude adult prevalence of raised blood pressure
0.0
0.1
0.2
0.3
0.4
0.5
0.6
1980
1990
2000
2010

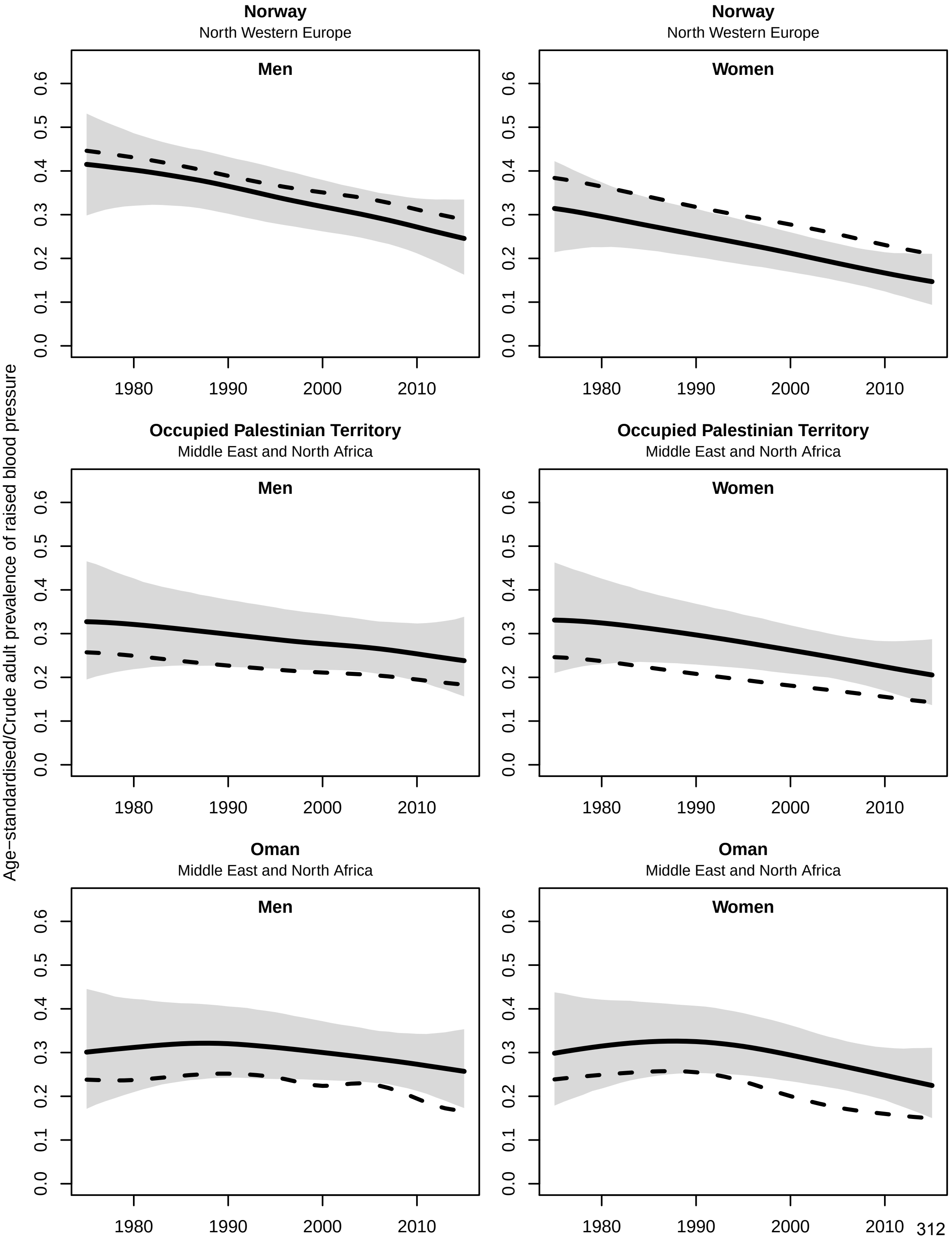
Norway
North Western Europe
Men
Norway
North Western Europe
Women
Occupied Palestinian Territory
Middle East and North Africa
Men
Occupied Palestinian Territory
Middle East and North Africa
Women
Oman
Middle East and North Africa
Men
Oman
Middle East and North Africa
Women
Age−standardised/Crude adult prevalence of raised blood pressure
0.0
0.1
0.2
0.3
0.4
0.5
0.6
1980
1990
2000
2010

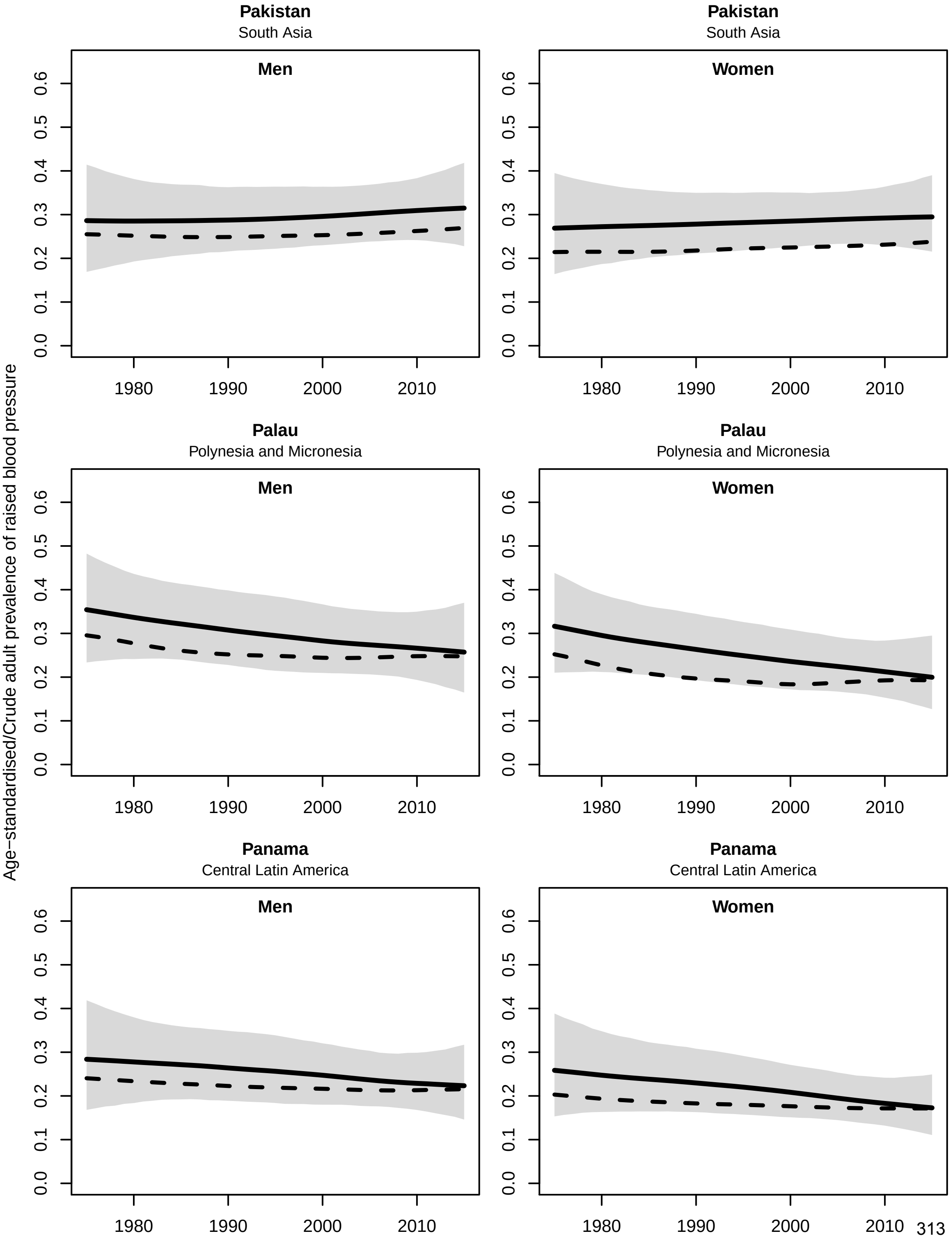
Pakistan
South Asia
Men
Pakistan
South Asia
Women
Palau
Polynesia and Micronesia
Men
Palau
Polynesia and Micronesia
Women
Panama
Central Latin America
Men
Panama
Central Latin America
Women
Age−standardised/Crude adult prevalence of raised blood pressure
0.0
0.1
0.2
0.3
0.4
0.5
0.6
1980
1990
2000
2010

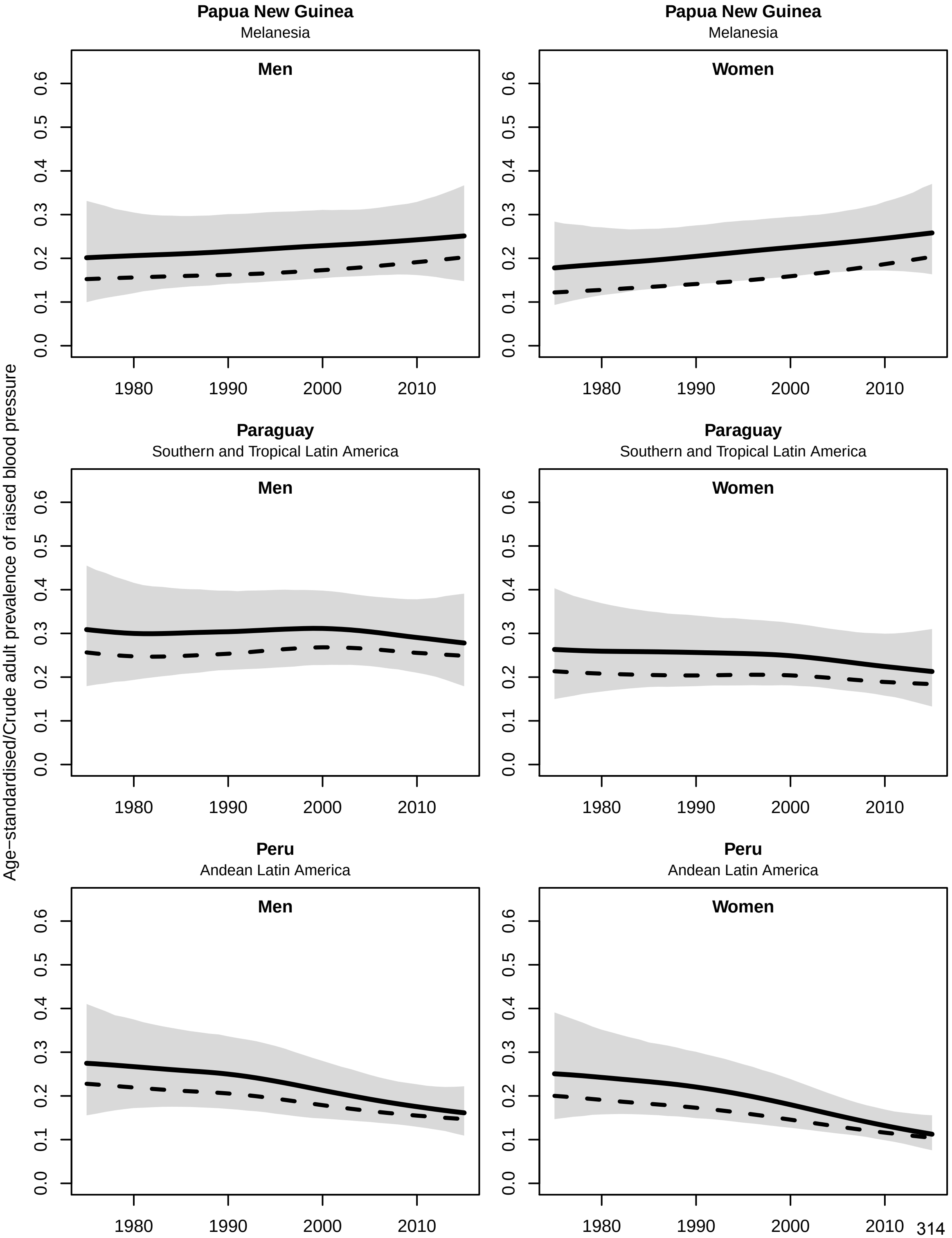
Papua New Guinea
Melanesia
Men
Women
Paraguay
Southern and Tropical Latin America
Men
Women
Peru
Andean Latin America
Men
Women
Age−standardised/Crude adult prevalence of raised blood pressure
0.0
0.1
0.2
0.3
0.4
0.5
0.6
1980
1990
2000
2010

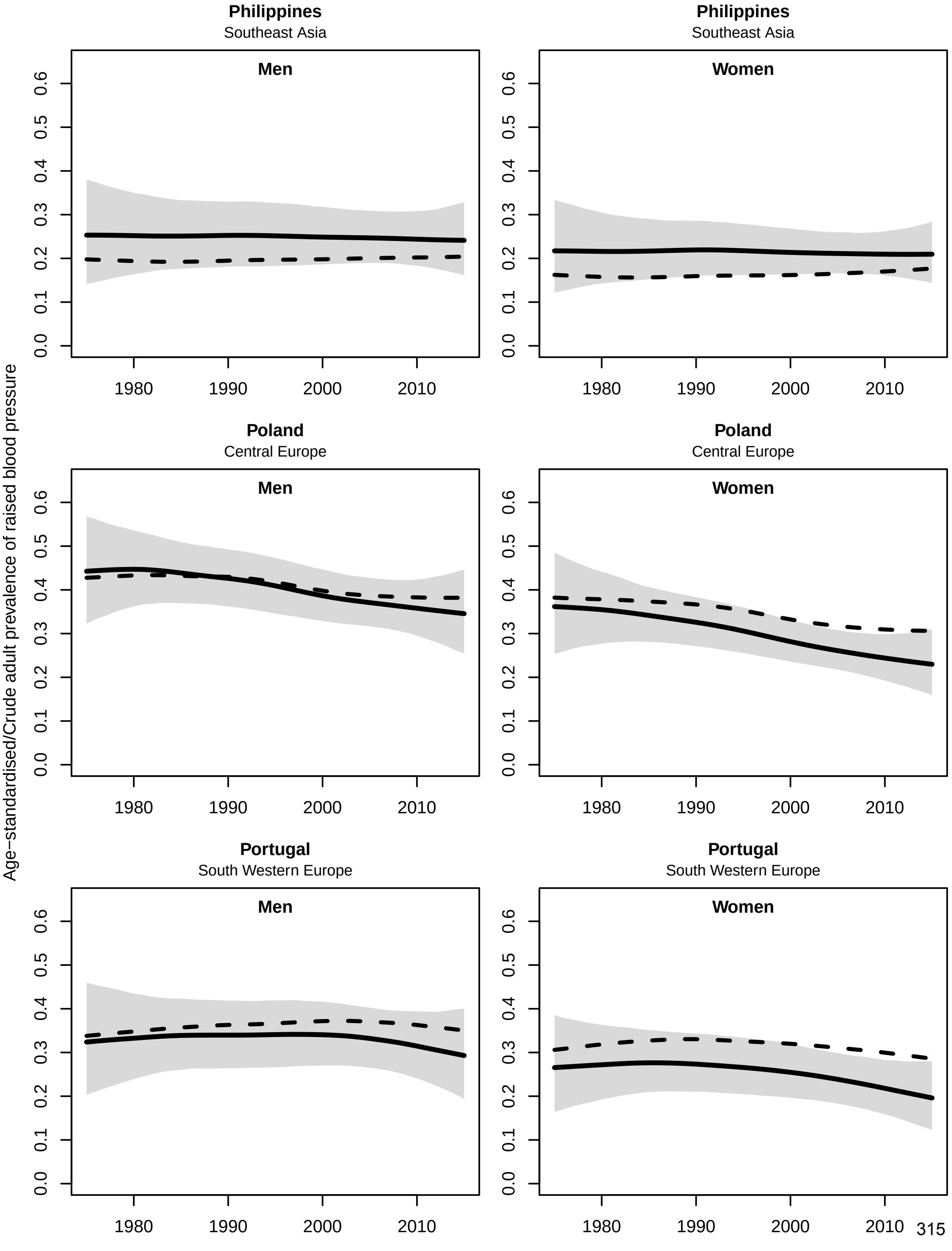

Philippines
Southeast Asia
Men
Women
Poland
Central Europe
Portugal
South Western Europe
Age−standardised/Crude adult prevalence of raised blood pressure
0.0
0.1
0.2
0.3
0.4
0.5
0.6
1980
1990
2000
2010

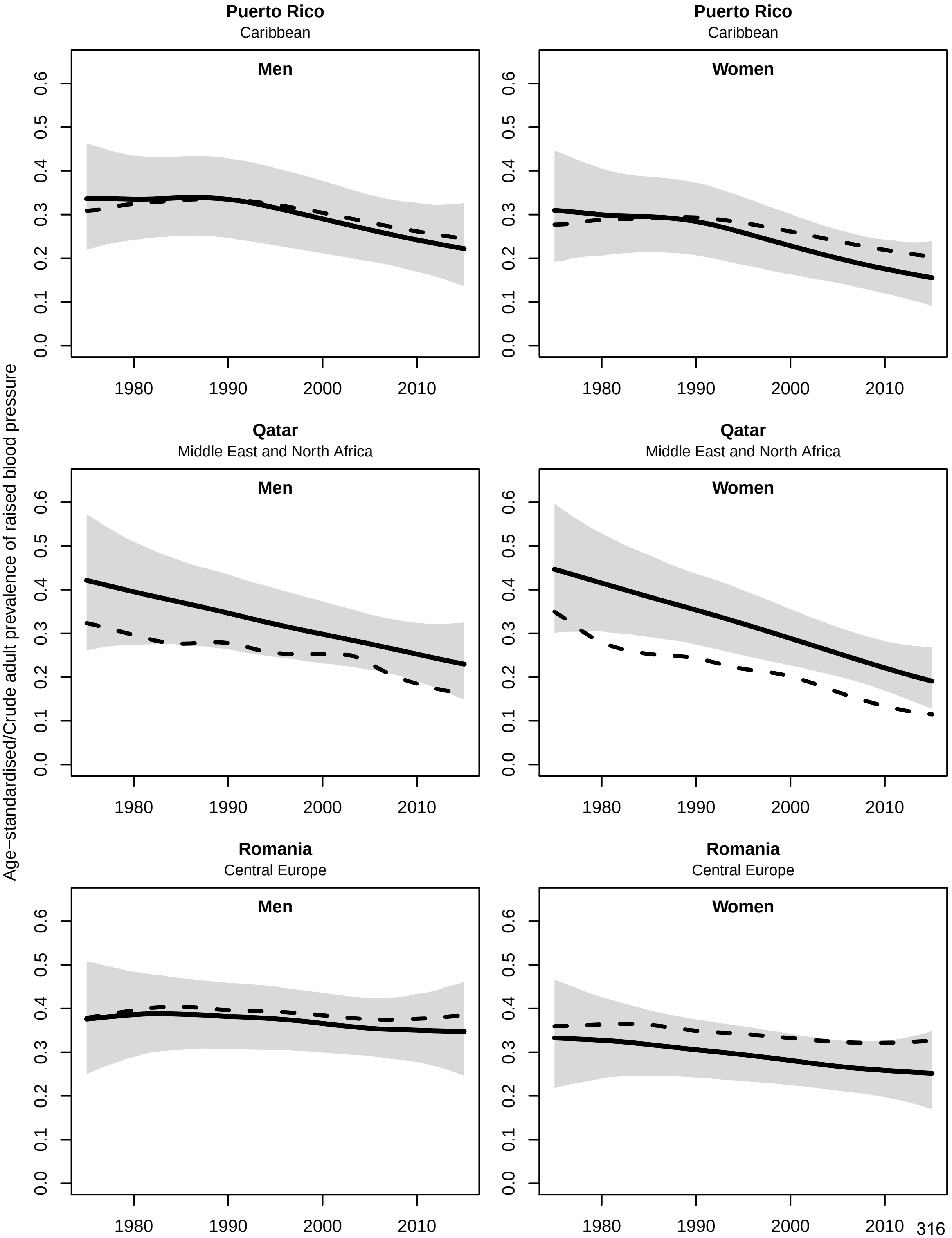
Puerto Rico
Caribbean
Men
Women
Qatar
Middle East and North Africa
Romania
Central Europe
Age−standardised/Crude adult prevalence of raised blood pressure
0.0
0.1
0.2
0.3
0.4
0.5
0.6
1980
1990
2000
2010

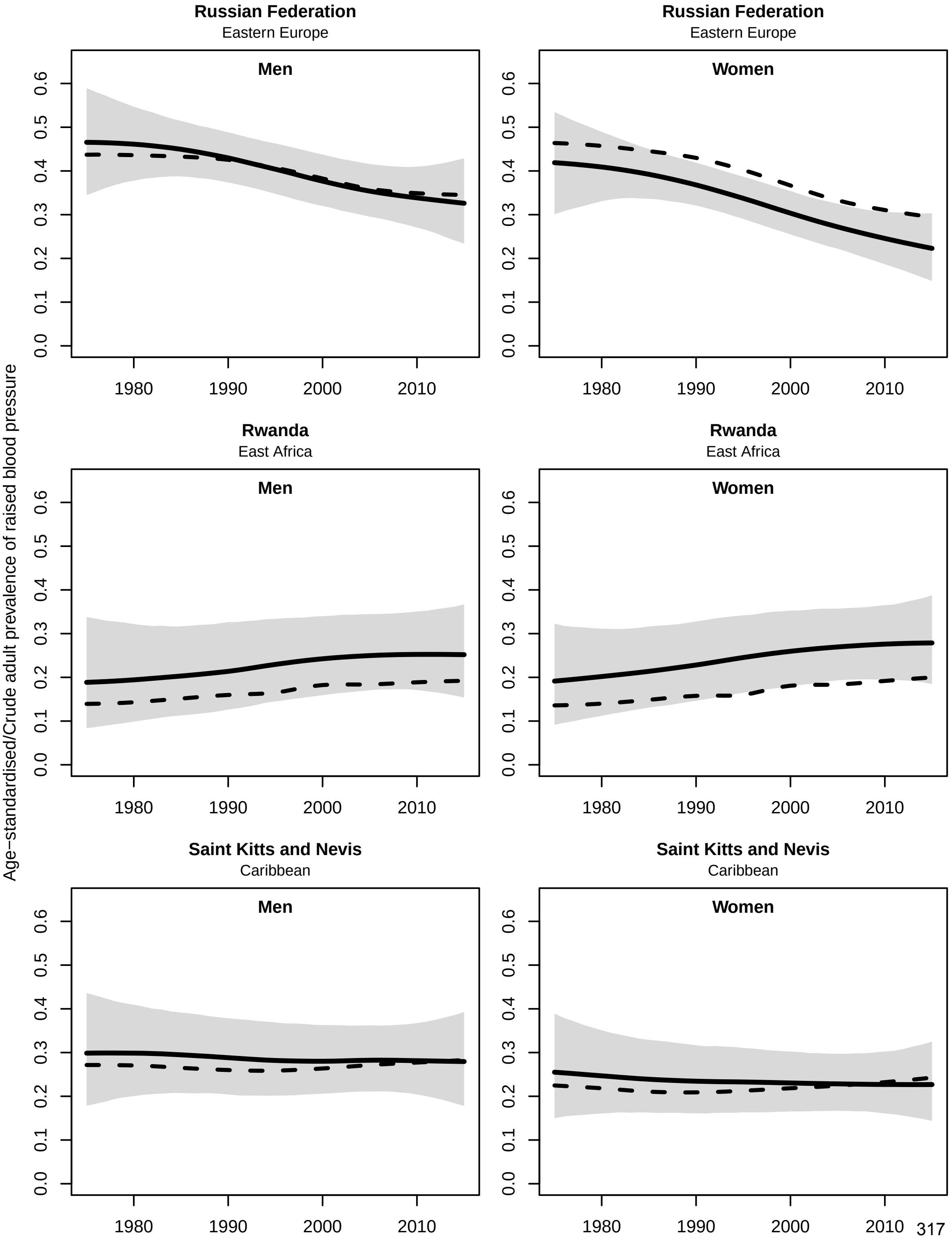
Russian Federation
Eastern Europe
Men
Russian Federation
Eastern Europe
Women
Rwanda
East Africa
Men
Rwanda
East Africa
Women
Saint Kitts and Nevis
Caribbean
Men
Saint Kitts and Nevis
Caribbean
Women
Age−standardised/Crude adult prevalence of raised blood pressure
0.0
0.1
0.2
0.3
0.4
0.5
0.6
1980
1990
2000
2010

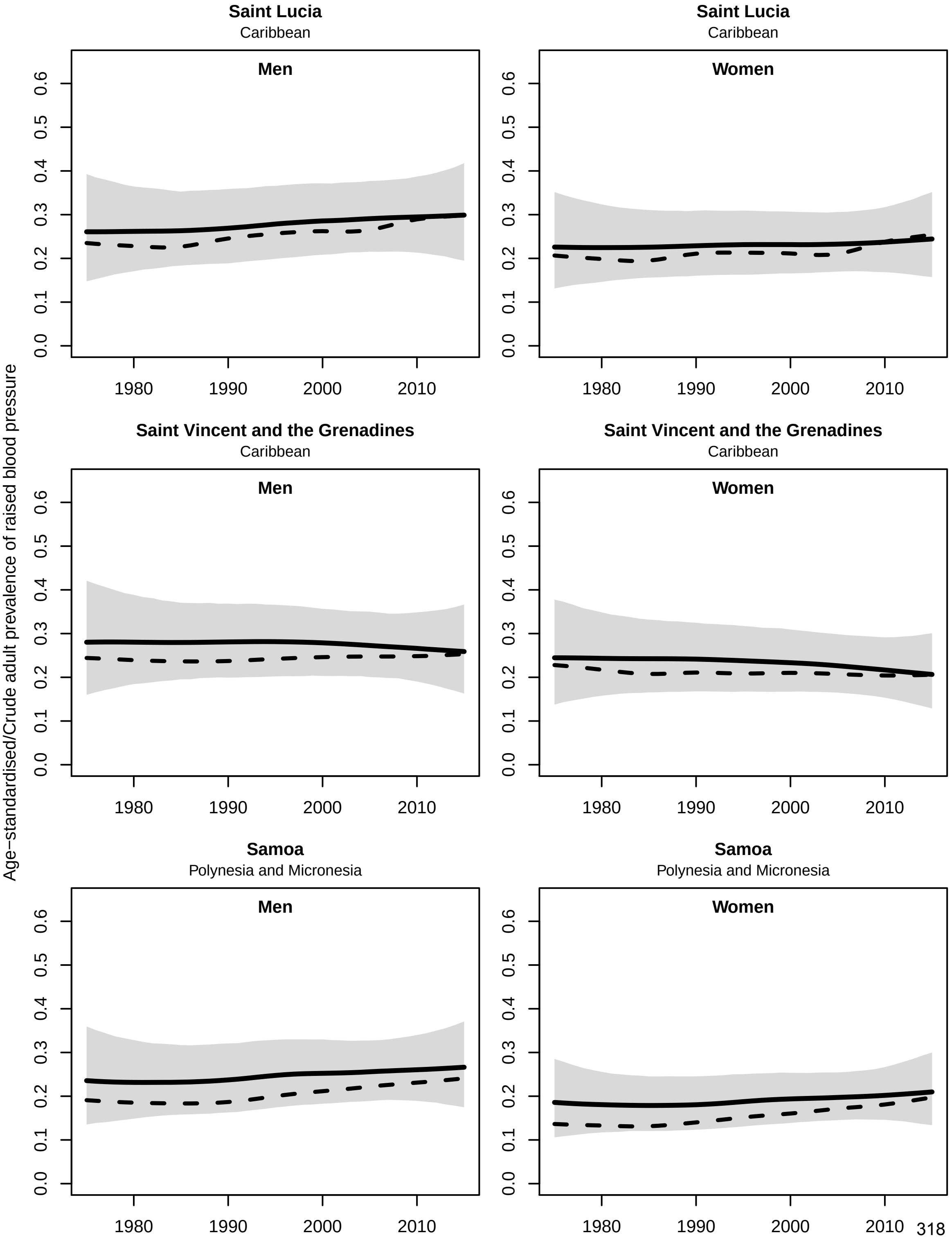
Age−standardised/Crude adult prevalence of raised blood pressure
Saint Lucia
Caribbean
Men
Saint Lucia
Caribbean
Women
Saint Vincent and the Grenadines
Caribbean
Men
Saint Vincent and the Grenadines
Caribbean
Women
Samoa
Polynesia and Micronesia
Men
Samoa
Polynesia and Micronesia
Women
0.0
0.1
0.2
0.3
0.4
0.5
0.6
1980
1990
2000
2010

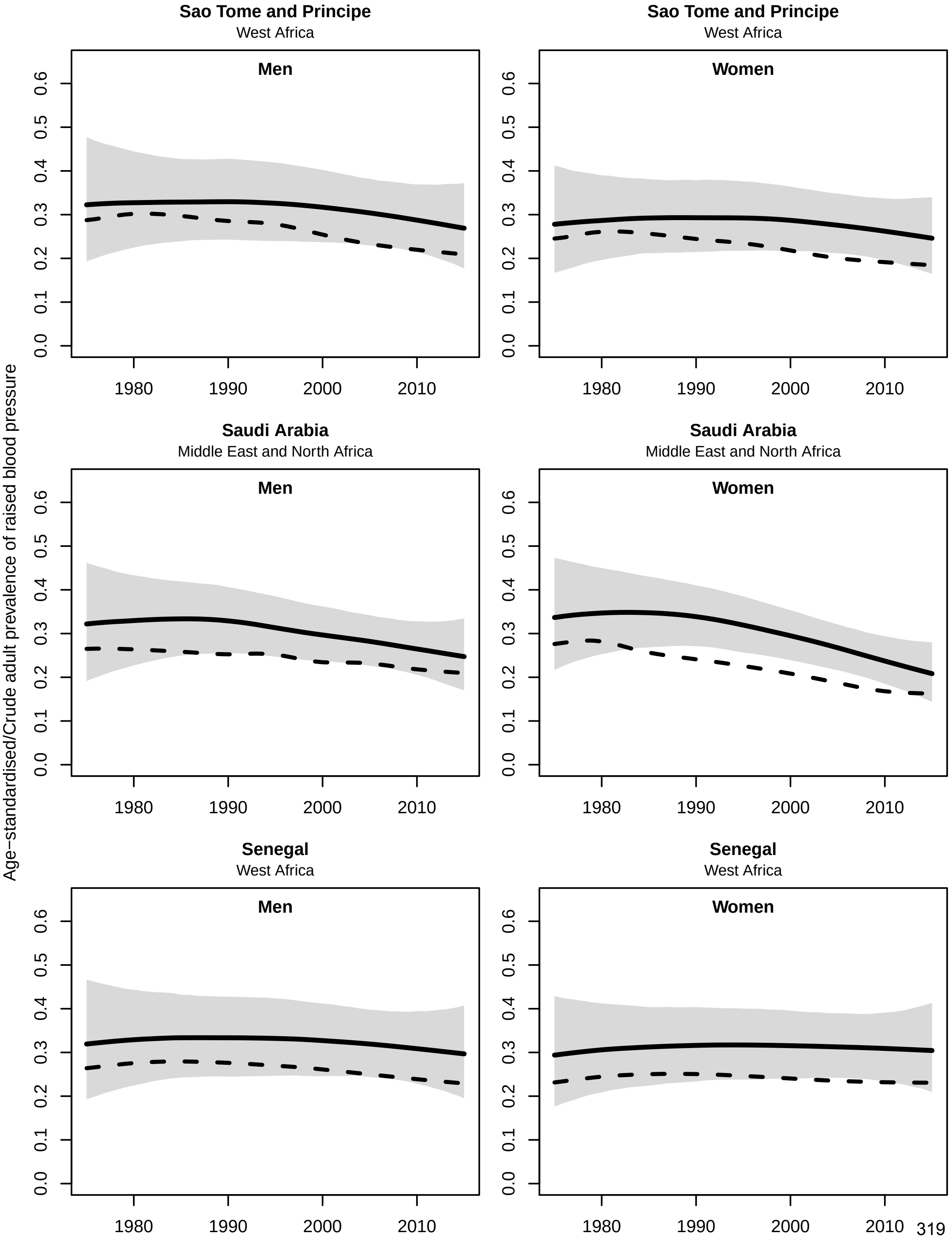
Sao Tome and Principe
West Africa
Men
Sao Tome and Principe
West Africa
Women
Saudi Arabia
Middle East and North Africa
Men
Saudi Arabia
Middle East and North Africa
Women
Senegal
West Africa
Men
Senegal
West Africa
Women
Age−standardised/Crude adult prevalence of raised blood pressure
0.0
0.1
0.2
0.3
0.4
0.5
0.6
1980
1990
2000
2010

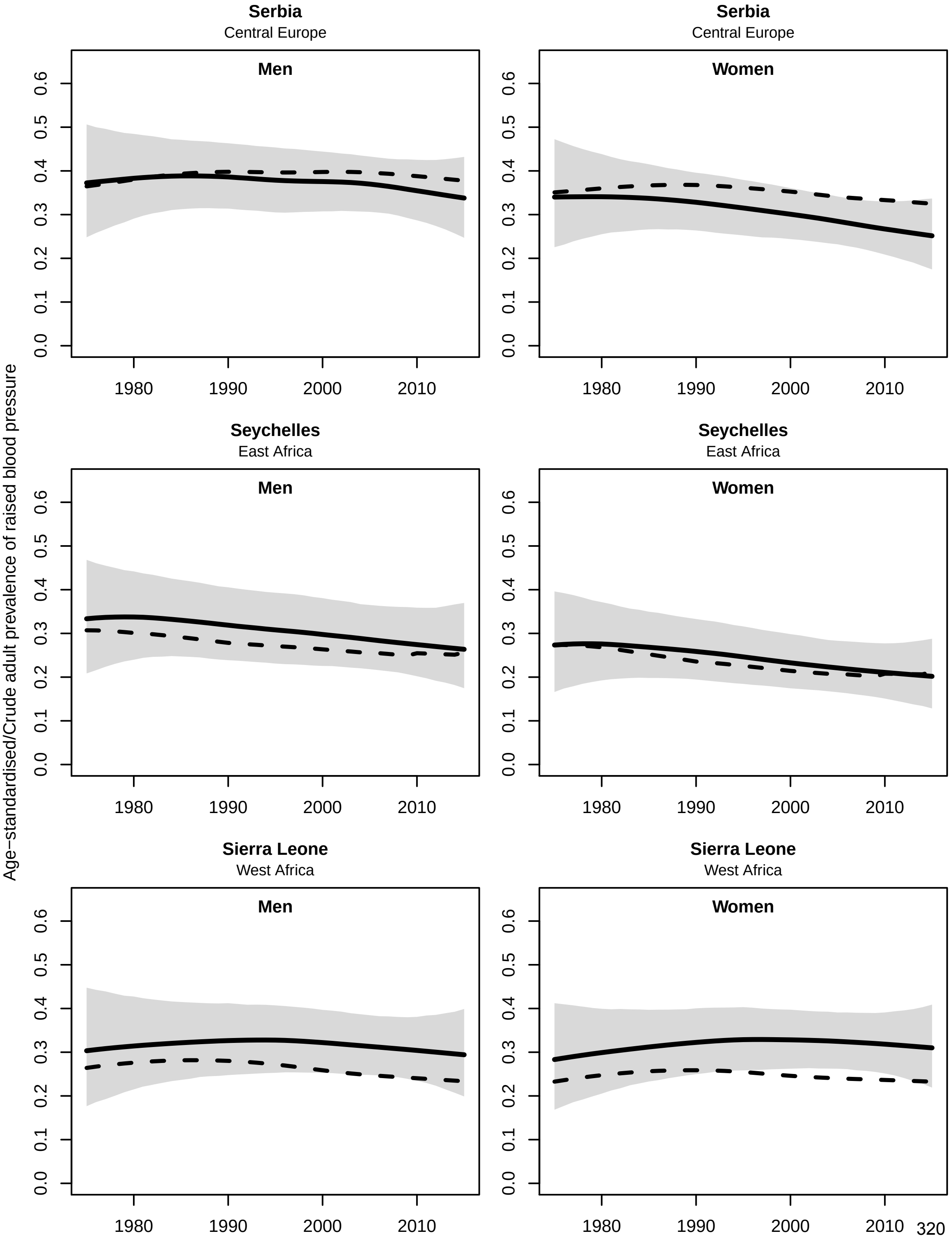
Serbia
Central Europe
Men
Women
Seychelles
East Africa
Sierra Leone
West Africa
Age−standardised/Crude adult prevalence of raised blood pressure
0.0
0.1
0.2
0.3
0.4
0.5
0.6
1980
1990
2000
2010

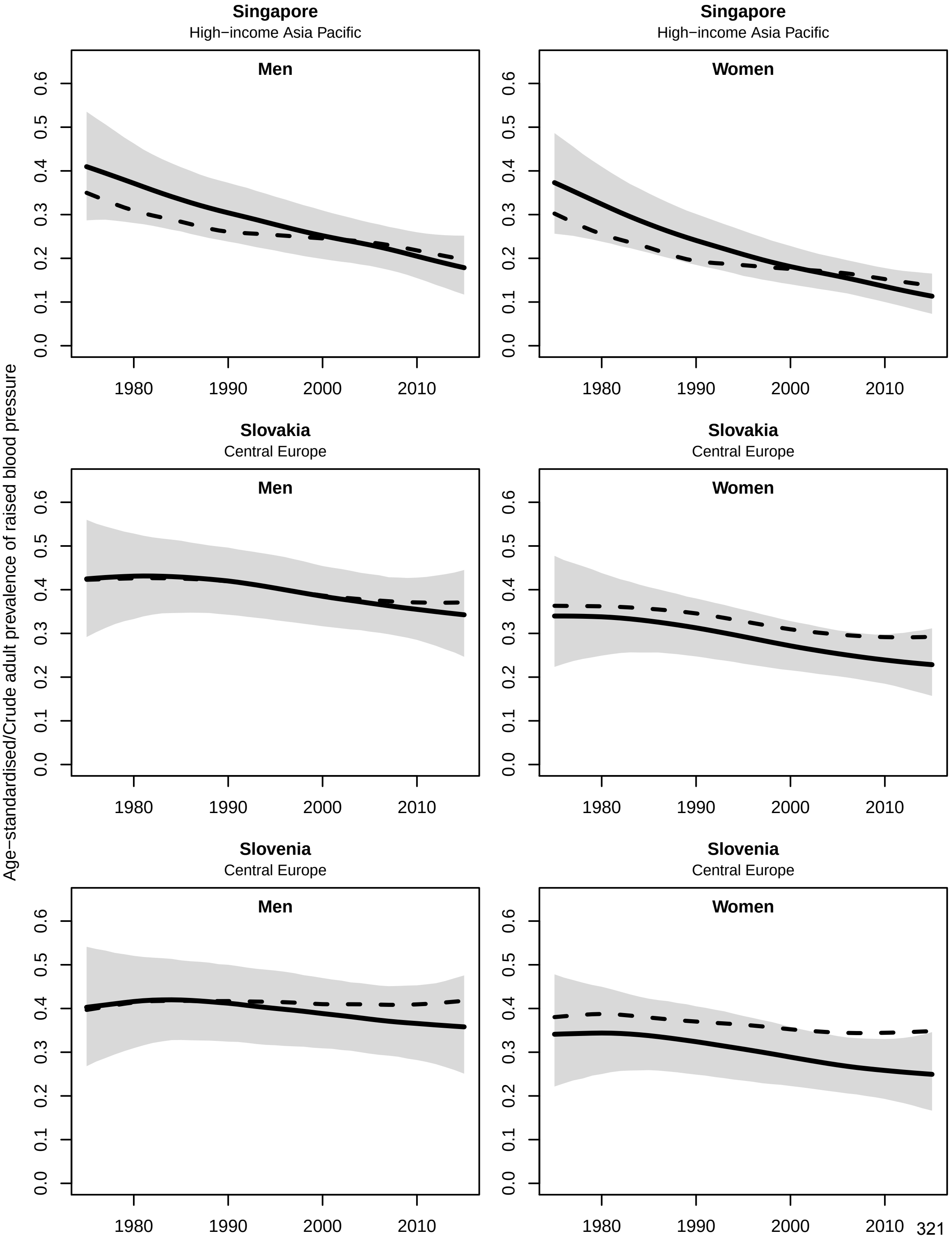
Singapore
High−income Asia Pacific
Men
Women
Slovakia
Central Europe
Slovenia
Central Europe
Age−standardised/Crude adult prevalence of raised blood pressure
0.0
0.1
0.2
0.3
0.4
0.5
0.6
1980
1990
2000
2010

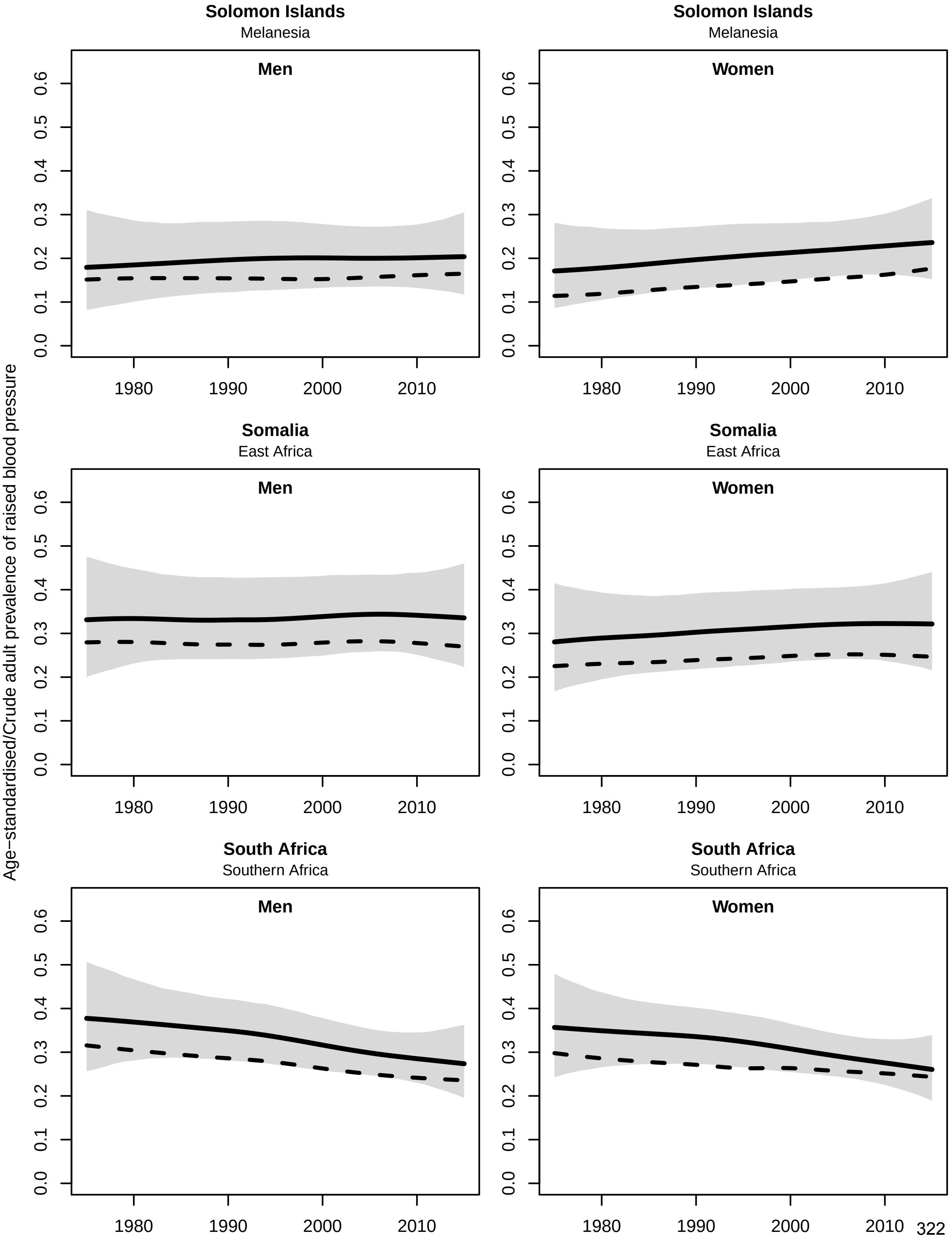
Solomon Islands
Melanesia
Men
Solomon Islands
Melanesia
Women
Somalia
East Africa
Men
Somalia
East Africa
Women
South Africa
Southern Africa
Men
South Africa
Southern Africa
Women
Age−standardised/Crude adult prevalence of raised blood pressure
0.0
0.1
0.2
0.3
0.4
0.5
0.6
1980
1990
2000
2010

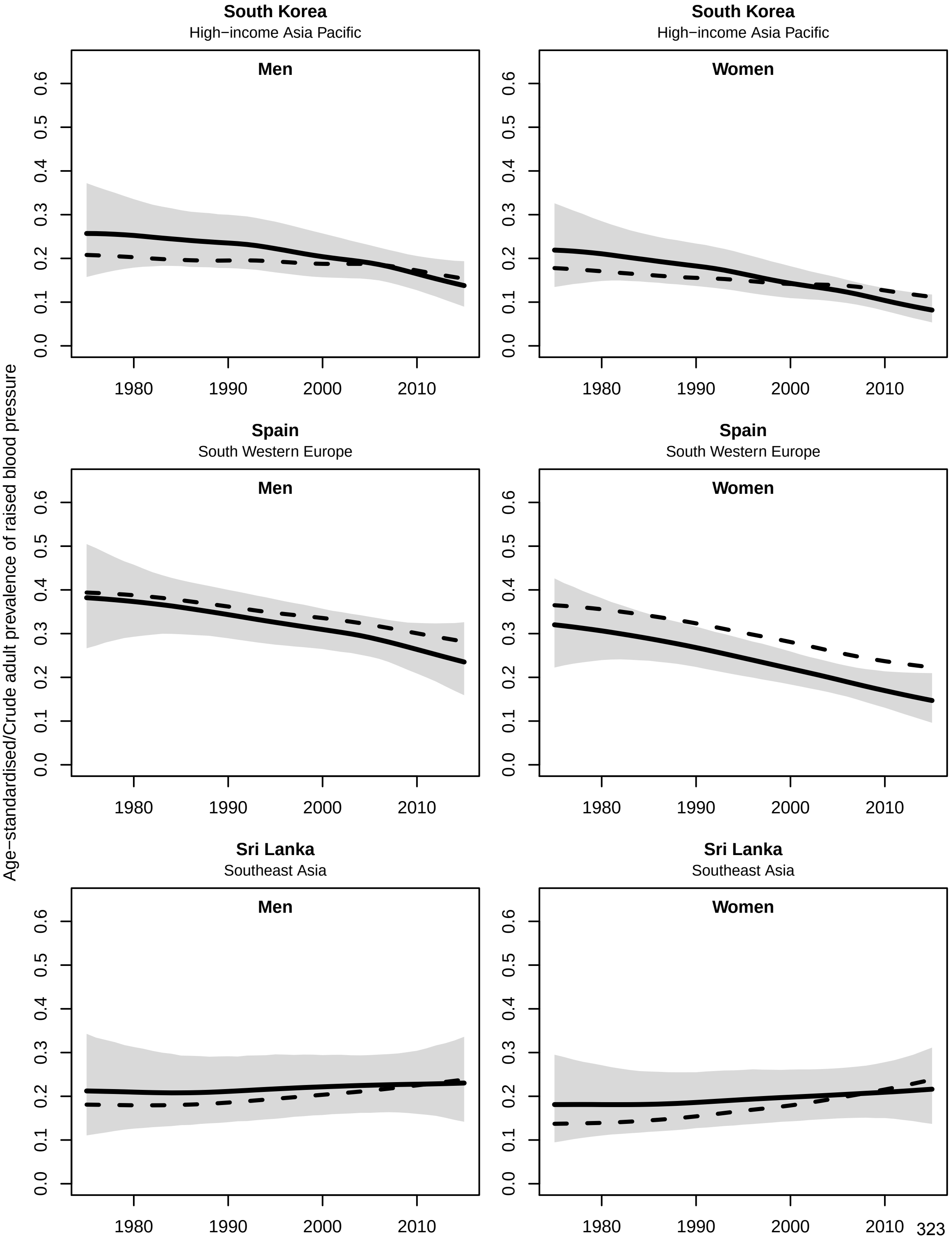

South Korea
High−income Asia Pacific
Men
South Korea
High−income Asia Pacific
Women
Spain
South Western Europe
Men
Spain
South Western Europe
Women
Sri Lanka
Southeast Asia
Men
Sri Lanka
Southeast Asia
Women
Age−standardised/Crude adult prevalence of raised blood pressure
0.0
0.1
0.2
0.3
0.4
0.5
0.6
1980
1990
2000
2010

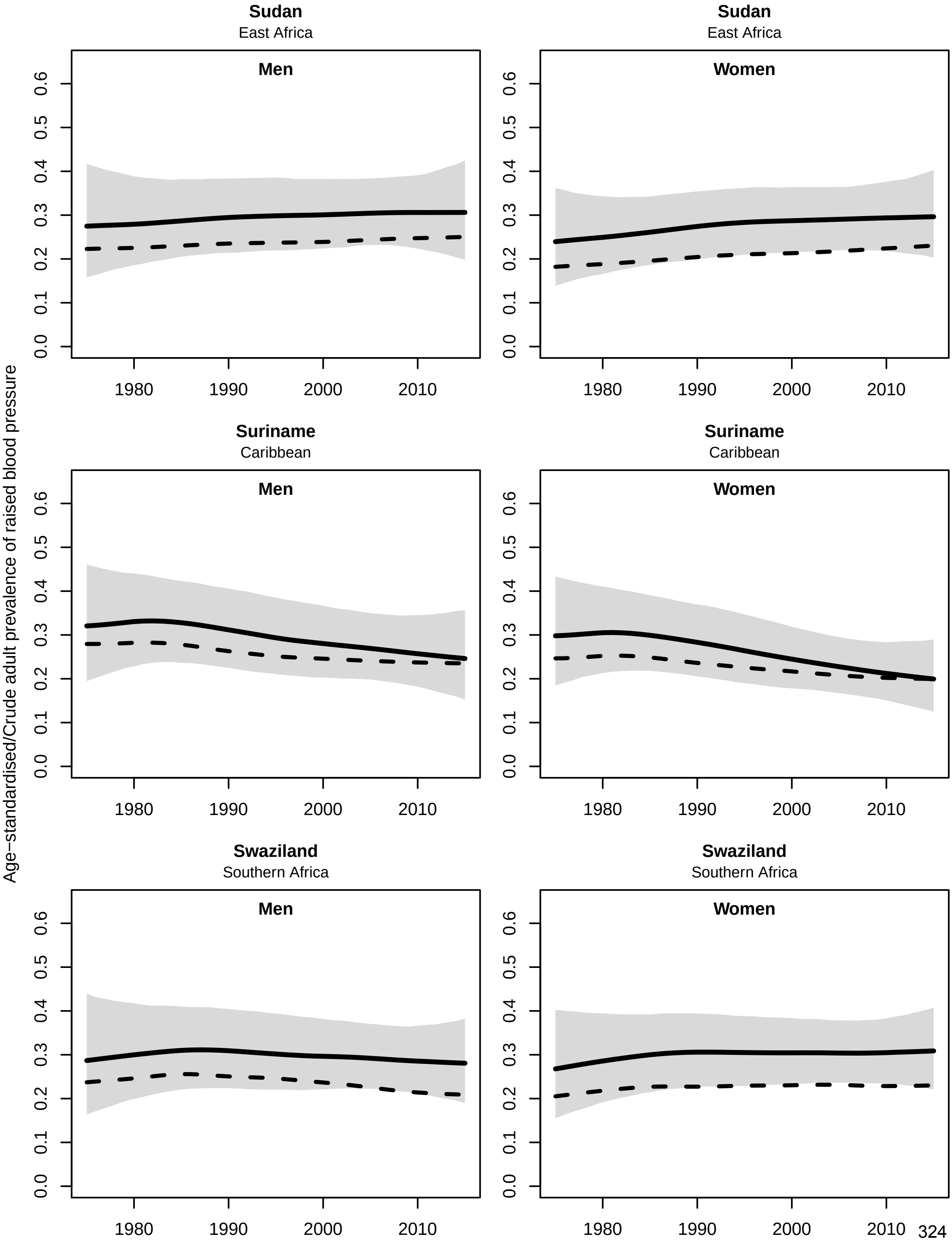
Sudan
East Africa
Men
Sudan
East Africa
Women
Suriname
Caribbean
Men
Suriname
Caribbean
Women
Swaziland
Southern Africa
Men
Swaziland
Southern Africa
Women
Age−standardised/Crude adult prevalence of raised blood pressure
0.0
0.1
0.2
0.3
0.4
0.5
0.6
1980
1990
2000
2010

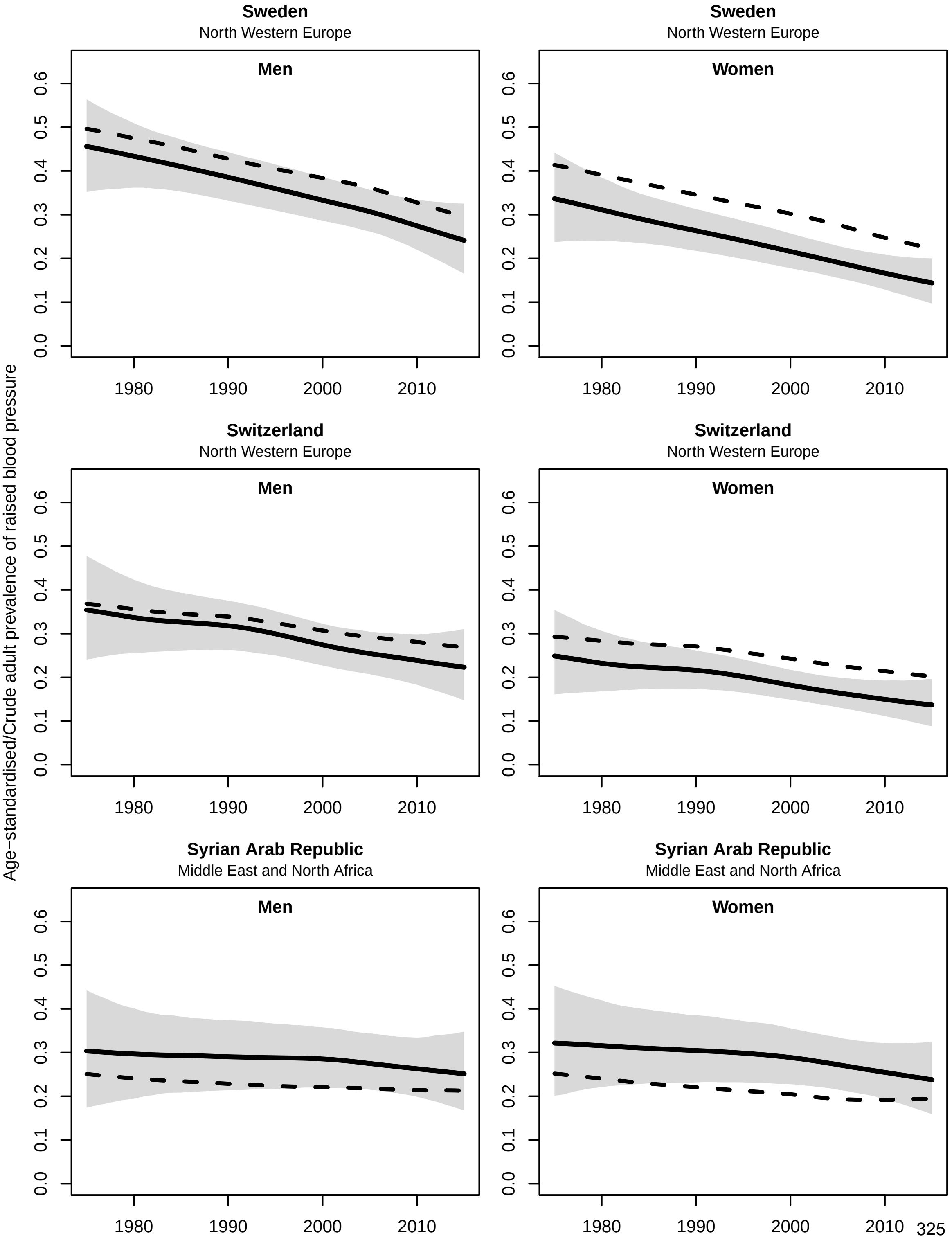
Age−standardised/Crude adult prevalence of raised blood pressure
Sweden
North Western Europe
Men
Women
Switzerland
North Western Europe
Men
Women
Syrian Arab Republic
Middle East and North Africa
Men
Women
0.0
0.1
0.2
0.3
0.4
0.5
0.6
1980
1990
2000
2010

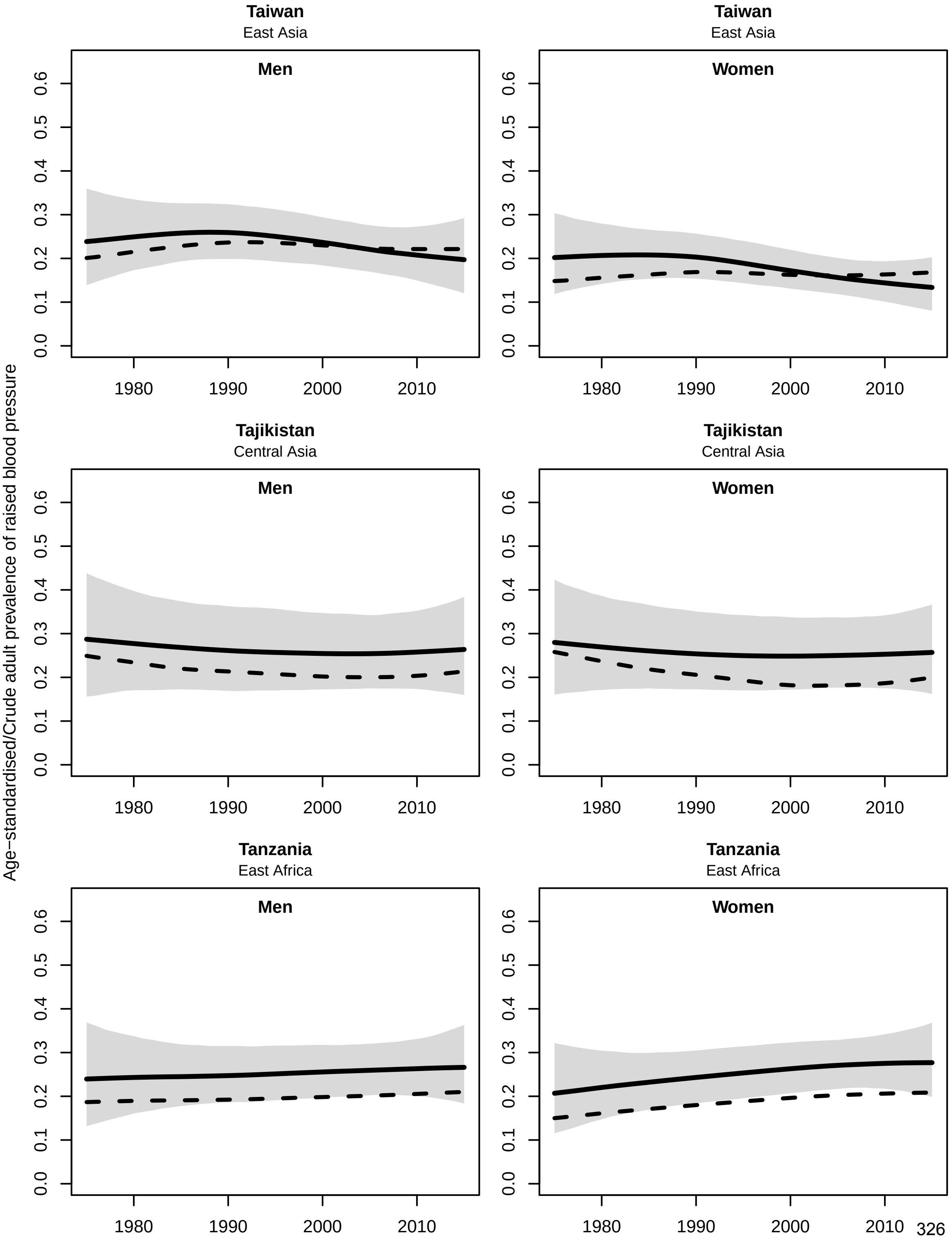
Taiwan
East Asia
Men
Women
Tajikistan
Central Asia
Tanzania
East Africa
Age−standardised/Crude adult prevalence of raised blood pressure
0.0
0.1
0.2
0.3
0.4
0.5
0.6
1980
1990
2000
2010

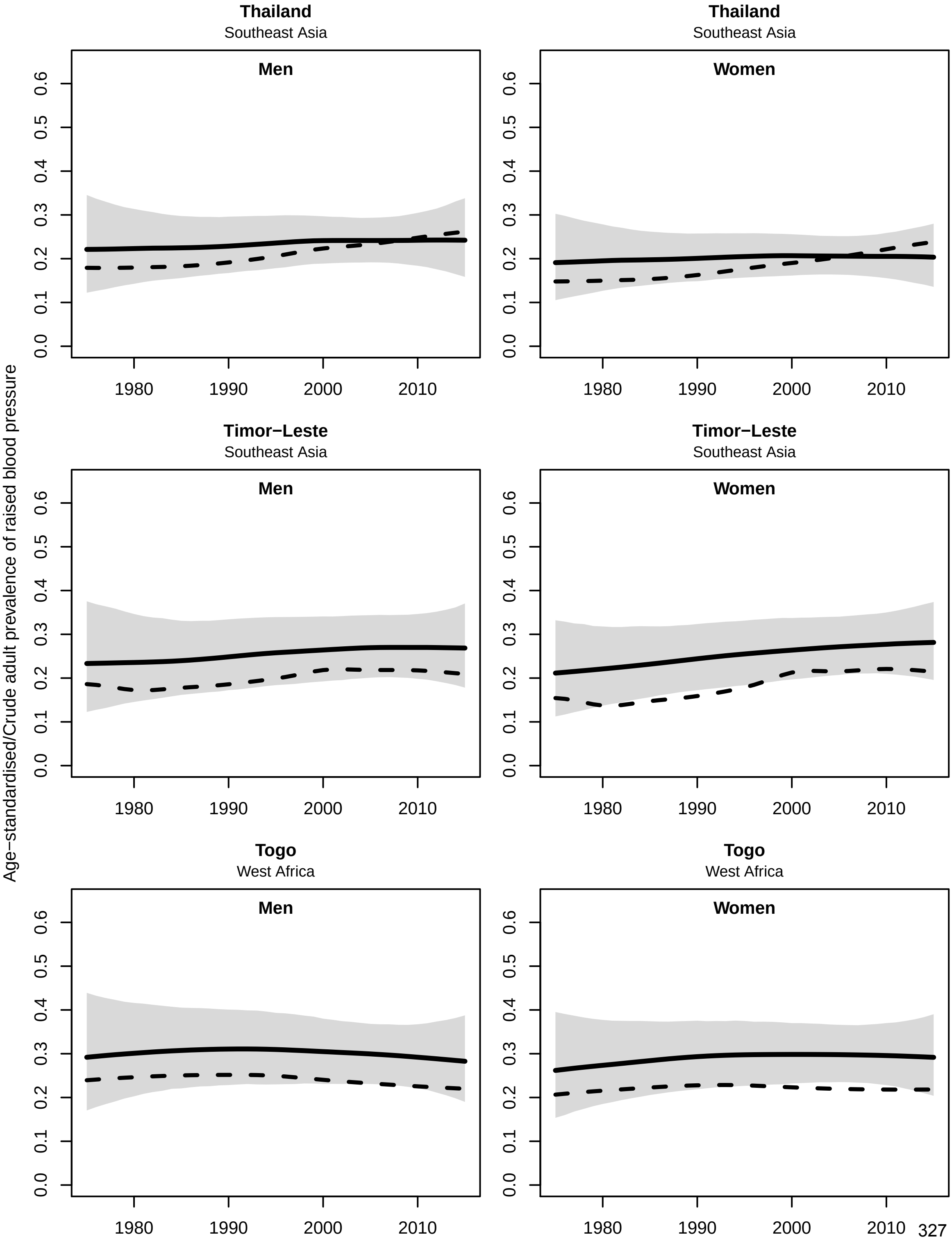
Thailand
Southeast Asia
Men
Thailand
Southeast Asia
Women
Timor−Leste
Southeast Asia
Men
Timor−Leste
Southeast Asia
Women
Togo
West Africa
Men
Togo
West Africa
Women
Age−standardised/Crude adult prevalence of raised blood pressure
0.0
0.1
0.2
0.3
0.4
0.5
0.6
1980
1990
2000
2010

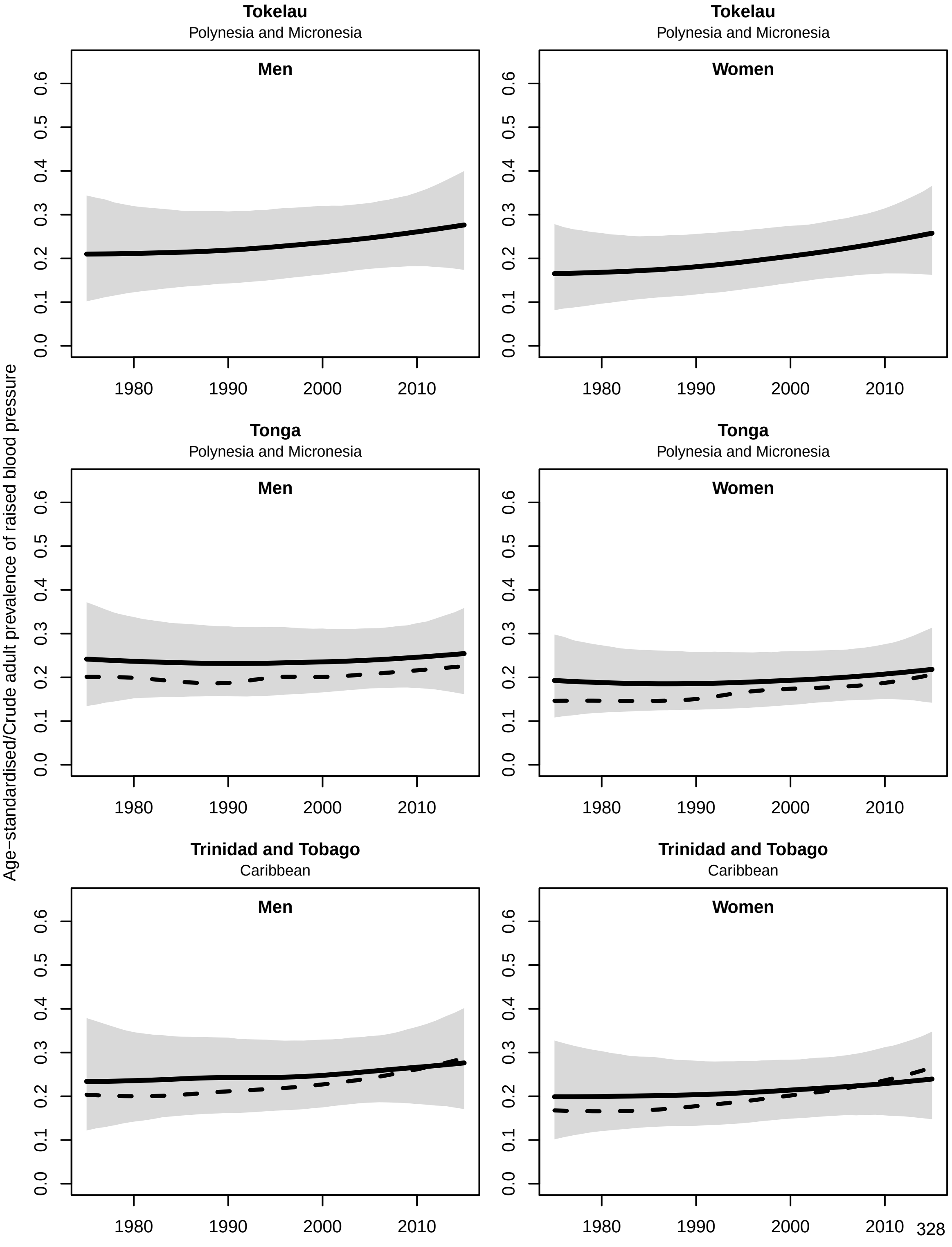
Age−standardised/Crude adult prevalence of raised blood pressure
Tokelau
Polynesia and Micronesia
Men
Women
Tonga
Polynesia and Micronesia
Men
Women
Trinidad and Tobago
Caribbean
Men
Women
0.0
0.1
0.2
0.3
0.4
0.5
0.6
1980
1990
2000
2010

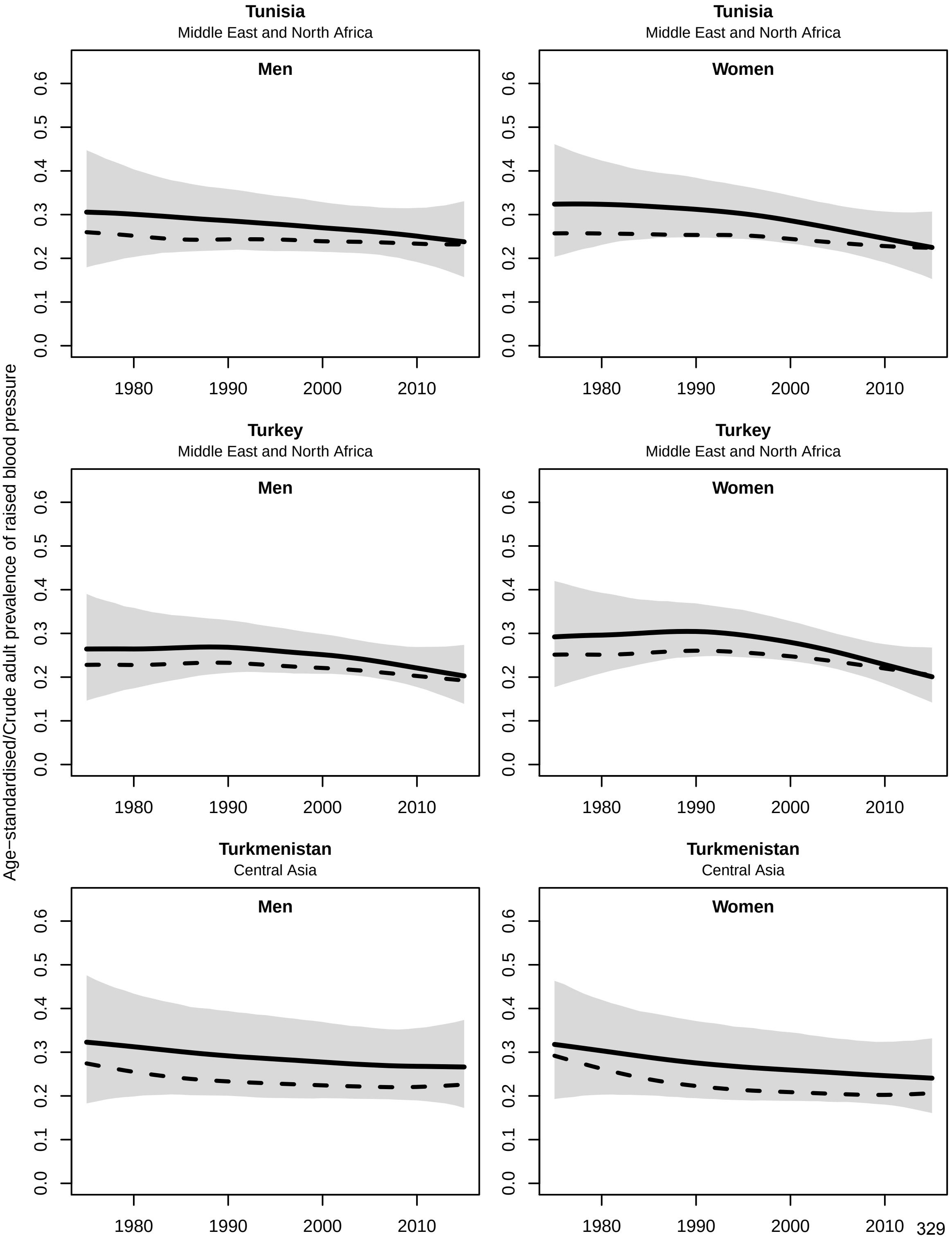
Tunisia
Middle East and North Africa
Men
Tunisia
Middle East and North Africa
Women
Turkey
Middle East and North Africa
Men
Turkey
Middle East and North Africa
Women
Turkmenistan
Central Asia
Men
Turkmenistan
Central Asia
Women
Age−standardised/Crude adult prevalence of raised blood pressure
0.0
0.1
0.2
0.3
0.4
0.5
0.6
1980
1990
2000
2010

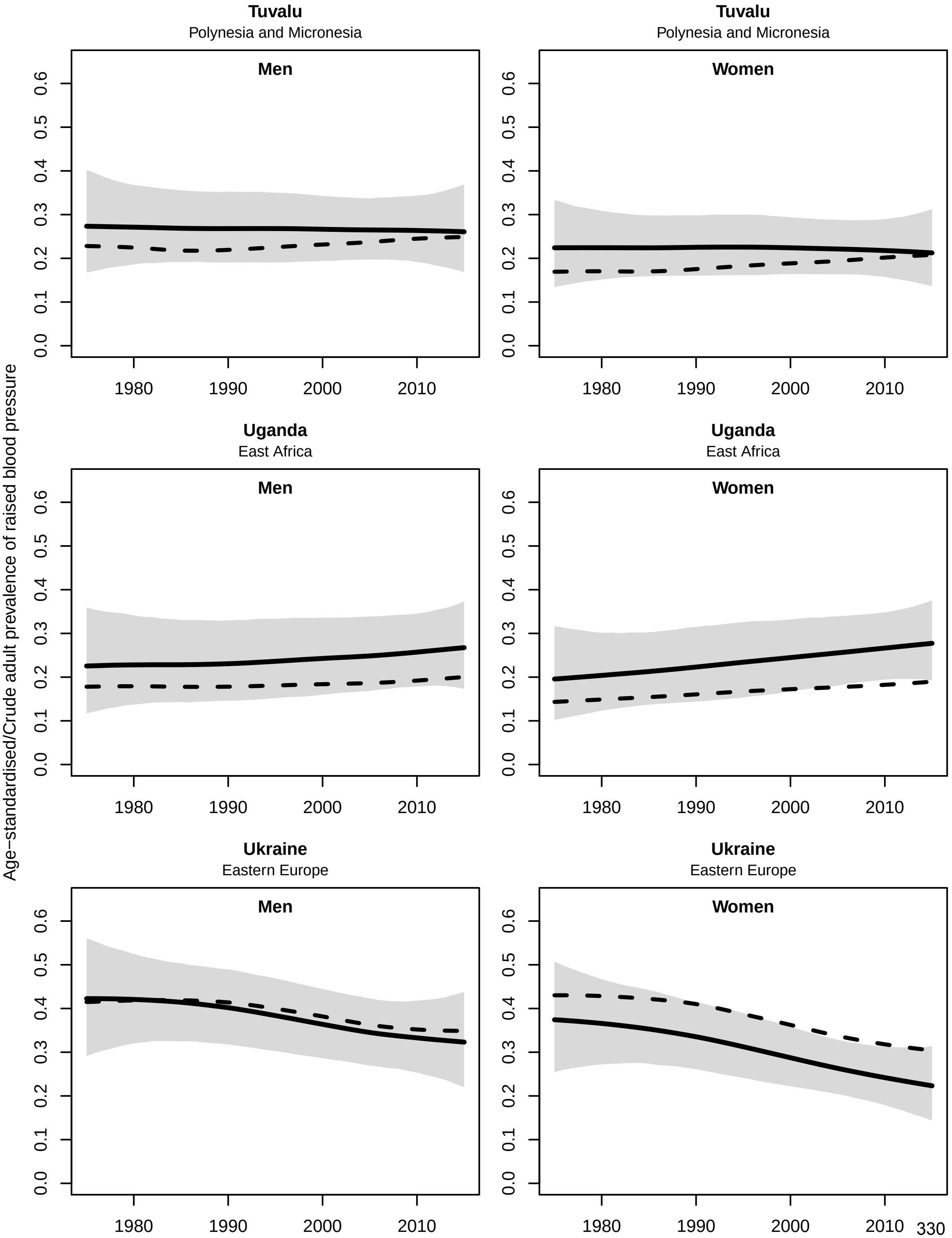

Tuvalu
Polynesia and Micronesia
Men
Women
Uganda
East Africa
Ukraine
Eastern Europe
Age−standardised/Crude adult prevalence of raised blood pressure
0.0
0.1
0.2
0.3
0.4
0.5
0.6
1980
1990
2000
2010

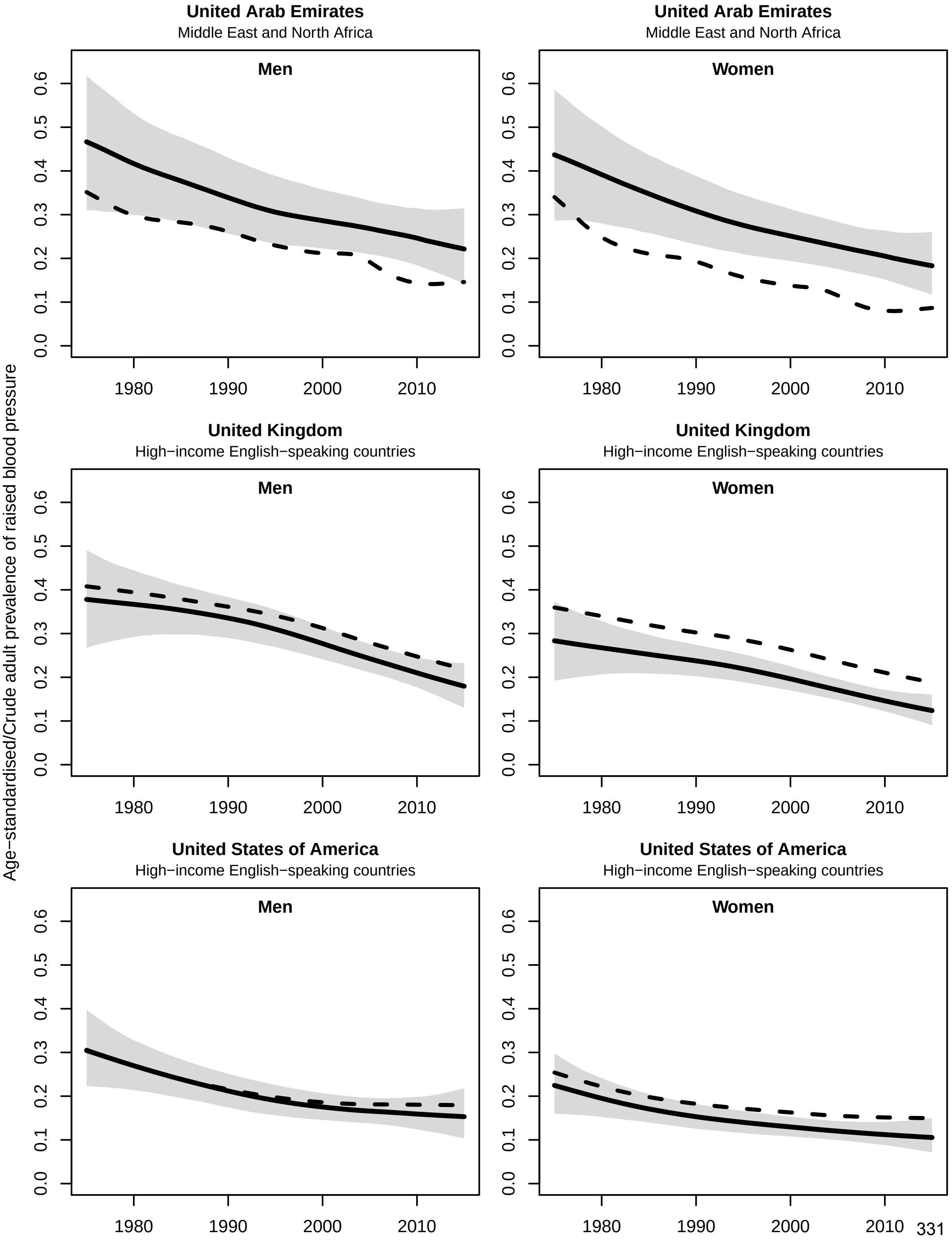
United Arab Emirates
Middle East and North Africa
Men
Women
United Kingdom
High−income English−speaking countries
United States of America
Age−standardised/Crude adult prevalence of raised blood pressure
0.0
0.1
0.2
0.3
0.4
0.5
0.6
1980
1990
2000
2010

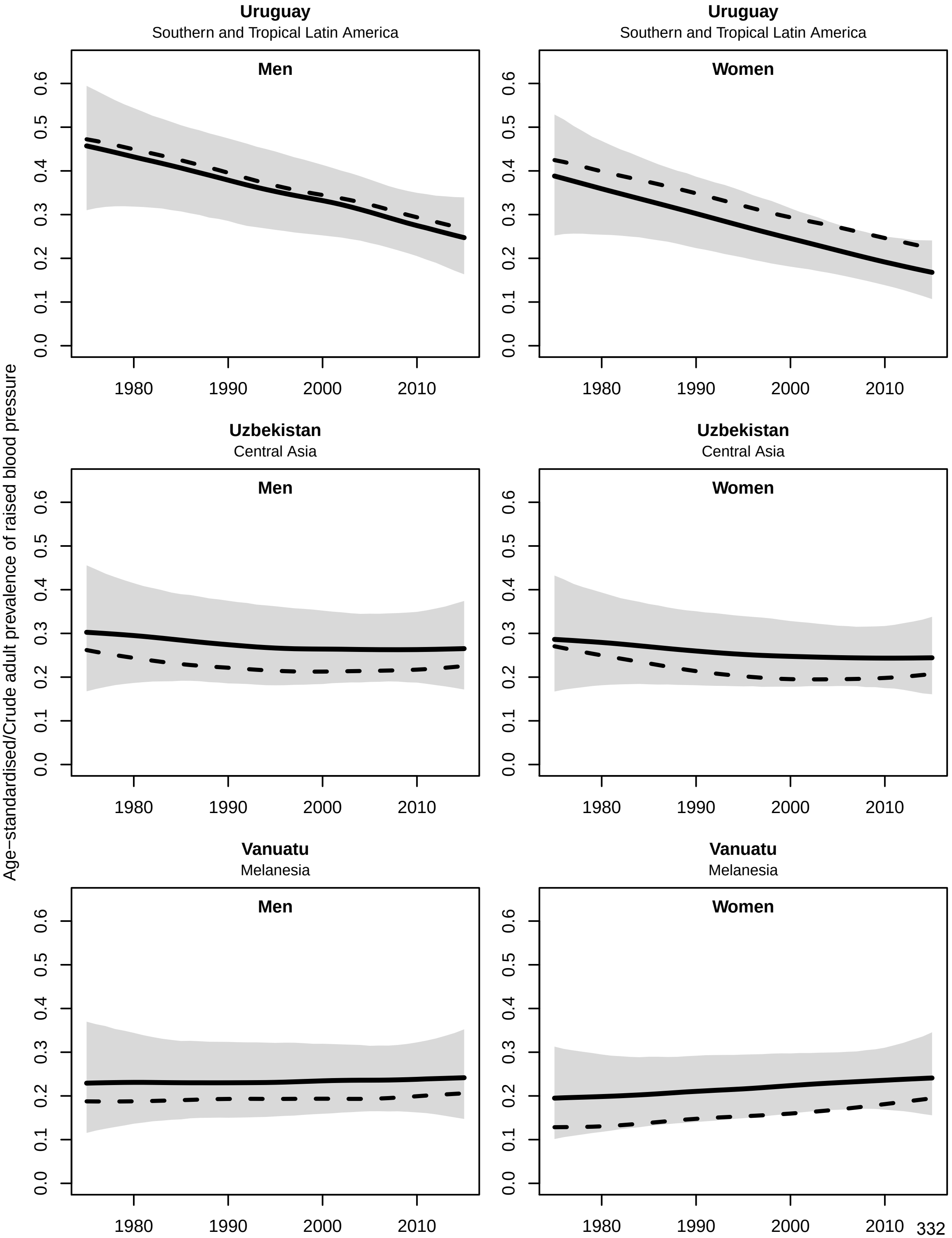
Age−standardised/Crude adult prevalence of raised blood pressure
Uruguay
Southern and Tropical Latin America
Men
Women
Uzbekistan
Central Asia
Men
Women
Vanuatu
Melanesia
Men
Women
0.0
0.1
0.2
0.3
0.4
0.5
0.6
1980
1990
2000
2010

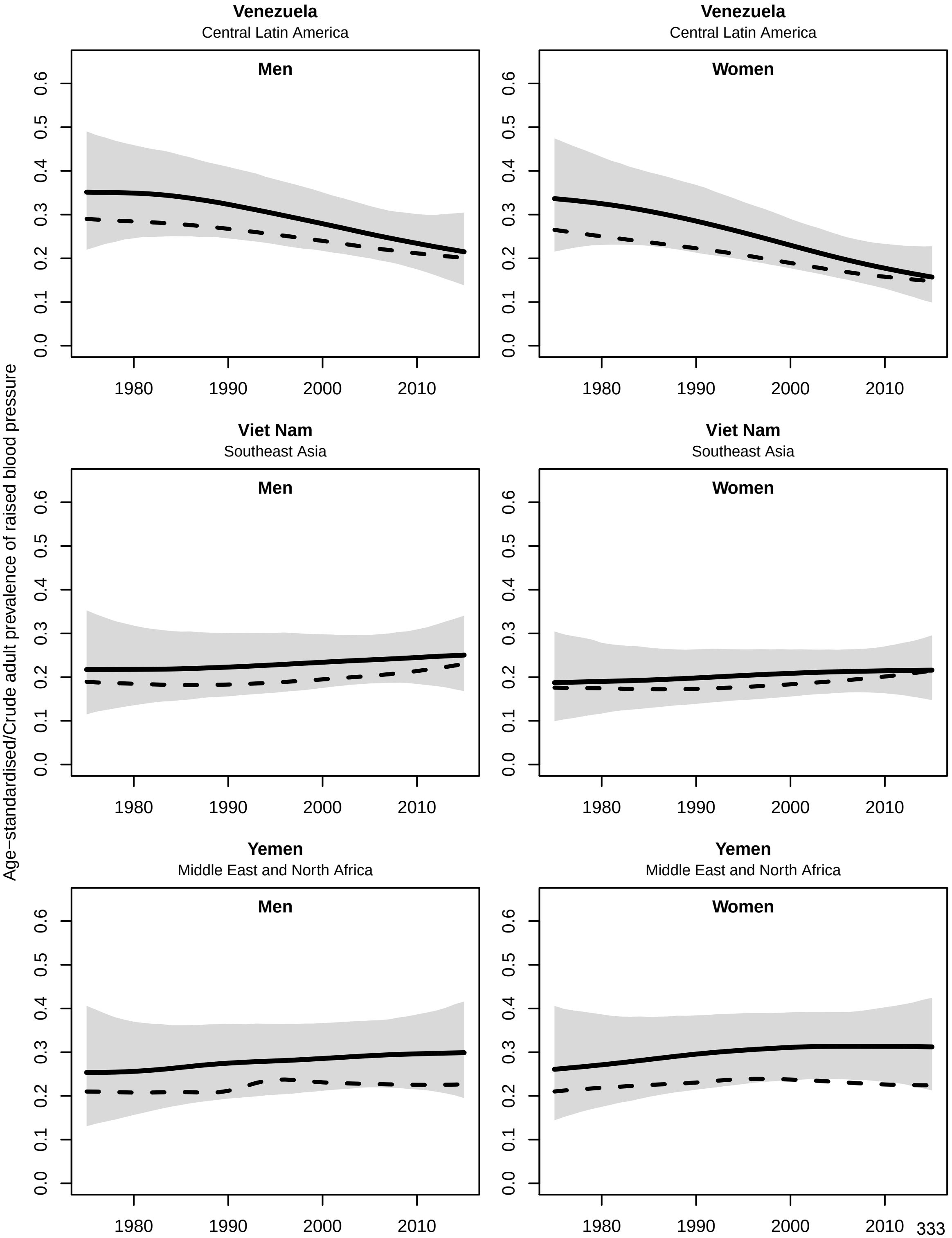
Venezuela
Central Latin America
Men
Venezuela
Central Latin America
Women
Viet Nam
Southeast Asia
Men
Viet Nam
Southeast Asia
Women
Yemen
Middle East and North Africa
Men
Yemen
Middle East and North Africa
Women
Age−standardised/Crude adult prevalence of raised blood pressure
0.0
0.1
0.2
0.3
0.4
0.5
0.6
1980
1990
2000
2010

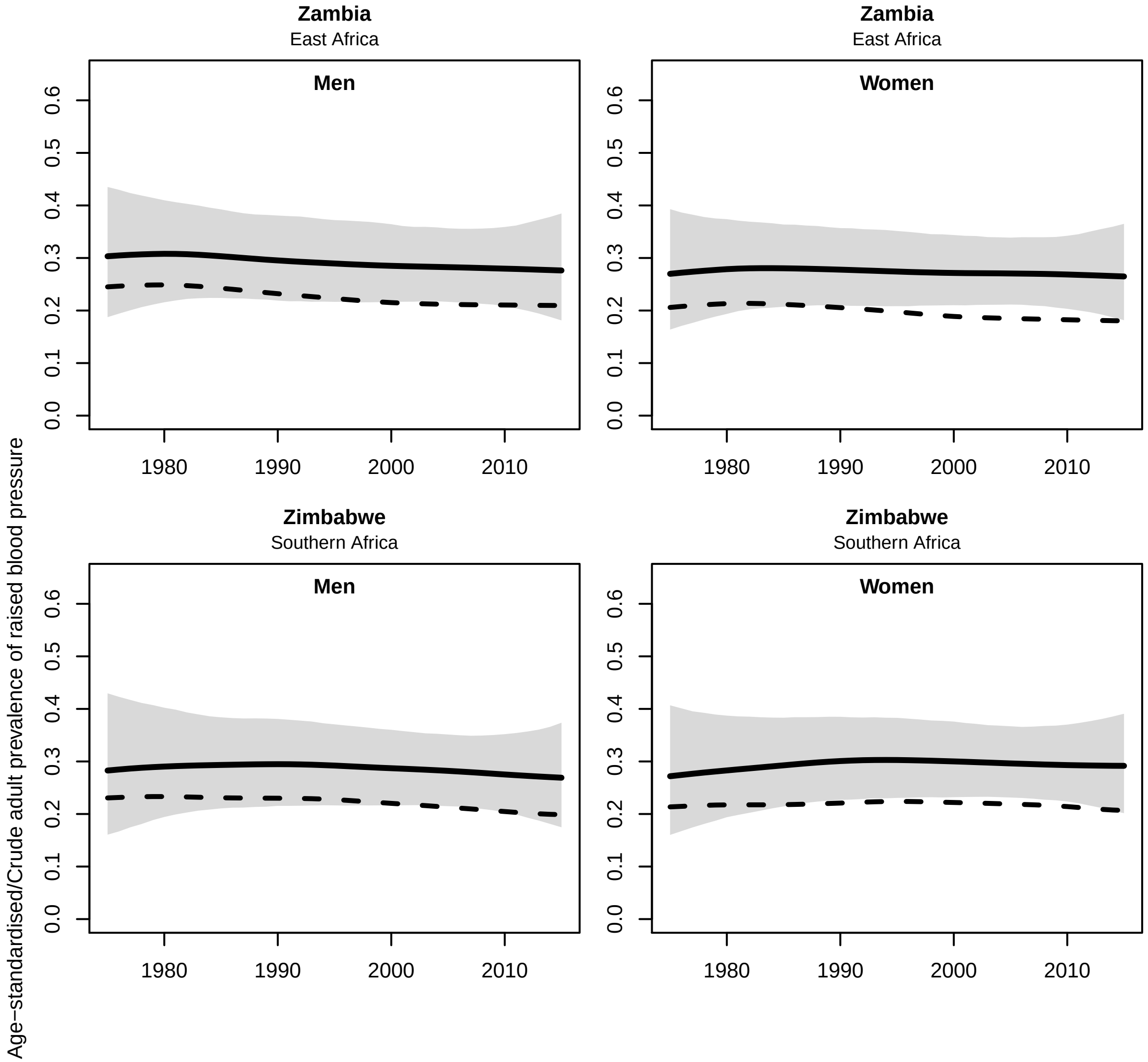
Zambia
East Africa
Men
Zambia
East Africa
Women
Zimbabwe
Southern Africa
Men
Zimbabwe
Southern Africa
Women
Age−standardised/Crude adult prevalence of raised blood pressure
0.0
0.1
0.2
0.3
0.4
0.5
0.6
1980
1990
2000
2010

References

1. Finucane MM, Stevens GA, Cowan MJ, et al. National, regional, and global trends in body-mass index since 1980: systematic analysis of health examination surveys and epidemiological studies with 960 country-years and 9.1 million participants. *Lancet* 2011; **377**(9765): 557-67.
2. Danaei G, Finucane MM, Lin JK, et al. National, regional, and global trends in systolic blood pressure since 1980: systematic analysis of health examination surveys and epidemiological studies with 786 country-years and 5·4 million participants. *Lancet* 2011; **377**(9765): 568-77.
3. Danaei G, Finucane MM, Lu Y, et al. National, regional, and global trends in fasting plasma glucose and diabetes prevalence since 1980: systematic analysis of health examination surveys and epidemiological studies with 370 country-years and 2.7 million participants. *Lancet* 2011; **378**(9785): 31-40.
4. Farzadfar F, Finucane MM, Danaei G, et al. National, regional, and global trends in serum total cholesterol since 1980: systematic analysis of health examination surveys and epidemiological studies with 321 country-years and 3.0 million participants. *Lancet* 2011; **377**(9765): 578-86.
5. NCD Risk Factor Collaboration. Trends in adult body-mass index in 200 countries from 1975 to 2014: a pooled analysis of 1698 population-based measurement studies with 19.2 million participants. *Lancet* 2016; **387**(10026): 1377-96.
6. NCD Risk Factor Collaboration. A century of trends in adult human height. *eLife* 2016; **5**: e13410.
7. NCD Risk Factor Collaboration. Worldwide trends in diabetes since 1980: a pooled analysis of 751 population-based studies with 4.4 million participants. *Lancet* 2016; **387**(10027): 1513-30.
8. Schwarz G. Estimating Dimension of a Model. *Ann Stat* 1978; **6**(2): 461-4.
9. Danaei G, Singh GM, Paciorek CJ, et al. The global cardiovascular risk transition: associations of four metabolic risk factors with national income, urbanization, and Western diet in 1980 and 2008. *Circulation* 2013; **127**(14): 1493-502, 502e1-8.
10. Ezzati M, Riboli E. Behavioral and dietary risk factors for noncommunicable diseases. *N Engl J Med* 2013; **369**(10): 954-64.
11. Nakagawa S, Schielzeth H. A general and simple method for obtaining R2 from generalized linear mixed-effects models. *Methods Ecol Evol* 2013; **4**(2): 133-42.
12. McFadden D. Conditional Logit Analysis of Qualitative Choice Behavior. In: Zarembka P, ed. Frontiers in econometrics: Academic Press; 1974: 105-42.
